# Identification of Differential Expression Genes between Volume and Pressure Overloaded Hearts Based on Bioinformatics Analysis

**DOI:** 10.3390/genes13071276

**Published:** 2022-07-19

**Authors:** Yuanfeng Fu, Di Zhao, Yufei Zhou, Jing Lu, Le Kang, Xueli Jiang, Ran Xu, Zhiwen Ding, Yunzeng Zou

**Affiliations:** 1 Shanghai Institute of Cardiovascular Diseases, Zhongshan Hospital, Fudan University, Shanghai 200032, China; shanjianhongye@126.com (Y.F.); 21111210059@m.fudan.edu.cn (D.Z.); 21111210061@m.fudan.edu.cn (Y.Z.); 19111510063@fudan.edu.cn (J.L.); kang.le@zs-hospital.sh.cn (L.K.); 19111210047@fudan.edu.cn (X.J.); xu.ran@zs-hospital.sh.cn (R.X.); 2 Institutes of Biomedical Sciences, Fudan University, Shanghai 200032, China

**Keywords:** volume overload, microarray datasets, deferentially expressed genes, biomarkers, bioinformatics analysis

## Abstract

Volume overload (VO) and pressure overload (PO) are two common pathophysiological conditions associated with cardiac disease. VO, in particular, often occurs in a number of diseases, and no clinically meaningful molecular marker has yet been established. We intend to find the main differential gene expression using bioinformatics analysis. GSE97363 and GSE52796 are the two gene expression array datasets related with VO and PO, respectively. The LIMMA algorithm was used to identify differentially expressed genes (DEGs) of VO and PO. The DEGs were divided into three groups and subjected to functional enrichment analysis, which comprised GO analysis, KEGG analysis, and the protein–protein interaction (PPI) network. To validate the sequencing data, cardiomyocytes from AR and TAC mouse models were used to extract RNA for qRT-PCR. The three genes with random absolute values of LogFC and indicators of heart failure (natriuretic peptide B, *NPPB*) were detected: carboxylesterase 1D (*CES1D*), whirlin (*WHRN*), and WNK lysine deficient protein kinase 2 (*WNK2*). The DEGs in VO and PO were determined to be 2761 and 1093, respectively, in this study. Following the intersection, 305 genes were obtained, 255 of which expressed the opposing regulation and 50 of which expressed the same regulation. According to the GO and pathway enrichment studies, DEGs with opposing regulation are mostly common in fatty acid degradation, propanoate metabolism, and other signaling pathways. Finally, we used Cytoscape’s three techniques to identify six hub genes by intersecting 255 with the opposite expression and constructing a PPI network. Peroxisome proliferator-activated receptor (*PPARα*), acyl-CoA dehydrogenase medium chain (*ACADM*), patatin-like phospholipase domain containing 2 (*PNPLA2*), isocitrate dehydrogenase 3 (*IDH3*), heat shock protein family D member 1 (*HSPD1*), and dihydrolipoamide S-acetyltransferase (*DLAT*) were identified as six potential genes. Furthermore, we predict that the hub genes *PPARα*, *ACADM*, and *PNPLA2* regulate VO myocardial changes via fatty acid metabolism and acyl-Coa dehydrogenase activity, and that these genes could be employed as basic biomarkers for VO diagnosis and treatment.

## 1. Introduction

Changes in social work pressure and nutritional structure aggravate heart failure (HF), which is a prevalent illness [1]. Volume overload (VO) is one of the most common causes of HF [2,3]. Anemia, hyperthyroidism, pregnancy-induced hypertension, and chronic renal failure can all increase cardiac preload and hence cause VO [4,5,6,7,8]. Although treatment of the underlying illness can postpone the onset of heart failure, how to appropriately preserve the heart and improve myocardial remodeling remains a critical concern when VO develops [7,9].

The production of VO is quite complex, and it is widely assumed that it is directly tied to the permanent activation of neurohumoral renin–angiotensin–aldosterone system (RAAS) [2,3,10]. In patients with HF, those heart failure with perserved ejection fraction (HFpEF: EF > 50%; also includes diastolic heart failure) or heart failure with mid-range ejection fraction (HFmrEF: EF 40–49%) ejection fraction frequently have VO [10,11,12]. This is in contrast to heart failure with a low or decreased ejection fraction (HFrEF: EF 40%; also known as systolic heart failure) [12]. Because VO and pressure overload(PO) mediate various types of HF, research have revealed that they differ in inflammatory response, oxidative stress, endothelial dysfunction, and other processes [13,14,15,16,17,18,19]. Several studies have found that the alterations in cardiomyocytes generated by VO and PO are distinct [20,21,22], although it has yet to be discovered which genes regulate these changes.

There is no identified biomarker to discriminate between VO and PO in extant fundamental research. Although ultrasound scanning can validate the presence of VO in clinical diagnosis and animal experiments [23,24], gene and protein biomarkers are still a more practical way of validation in cell research [25]. As a result, we attempted to use bioinformatics tools to assess the genetic information of existing VO and PO models in order to identify relevant indicators to guide VO detection.

## 2. Materials and Methods

### 2.1. Microarray Data Collection and Preprocessing

The gene expression profiles were screened and downloaded from the National Center for Biotechnology Information Gene Expression Omnibus (GEO; https://www.ncbi.nlm.nih.gov/geo/ (accessed on 4 November 2021)). In GSE97363, there were 10 mice in the control group and 4 mice receiving pulmonary insufficiency and stenosis (PSPI). In GSE52796, there were 3 mice in the control group and 5 mice receiving transverse aortic constriction (TAC) (showed in Table 1). In order to explore the difference between VO and PO, the right ventricular (RV) dilation and failure of mouse and the TAC datasets were included [26,27]. The datasets using transgenic mouse and suckling mouse were excluded, and only the datasets of wild-type mouse that underwent surgical treatment were kept. The TAC datasets in which the hypertrophic genes natriuretic peptide A (*NPPA*) and natriuretic peptide B (*NPPB*) remained unchanged were excluded from the analysis. Datasets with less than 3 samples per group were also excluded.

### 2.2. Study Design and Differentially Expressed Gene Screening

GSE97363 and GSE52796 were downloaded from GEO database through GEOquery package. The GEO dataset with low quality and low reads was eliminated, while the remainder of the expression set was changed to a logarithmic scale on a base-2 scale. The LIMMA package provides an integrated solution for analyzing data from gene expression experiments, containing rich features for information borrowing to overcome the problem of small sample sizes [28]. Furthermore, before completing studies, gene expression levels were standardized by averaging the treatments. By clustering samples using the correlation measure, a broad assessment of statistical implementation may be produced. Outlying samples can be identified using dendrograms based on the correlation measure [29]. Samples with an irregular distribution of noise intensities may provide a significant problem. This can be balanced by using non-normalized data to generate a box plot of log intensities before using absolute signal intensities, which results in a more equitable portrayal of data [30]. Then, the datasets were removed for the probes corresponding to multiple molecules for one probe; when encountering the probes corresponding to the same molecule, only the probe with the largest signal value was retained, and the filtered data used the combatting batch effect (ComBat) function of the SVA package to remove the inter-batch difference (different datasets are regarded as the inter-batch difference) [31]. To acquire their respective differential expression values, the DEGs in each database were computed and processed, respectively.

**Figure 1:**
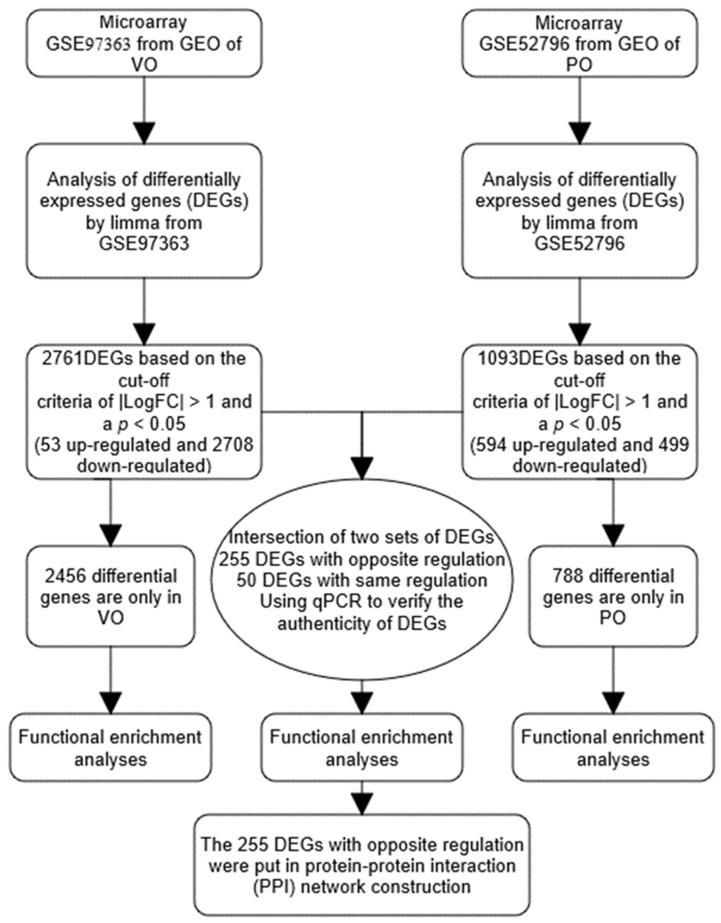
Flowchart of data analysis. VO: volume overload; PO: pressure overload.

The cut-off used to select DEGs was defined as *p*-value < 0.05, and |log fold−change (FC)| > 0.5 between each model category using student t-test for additional review. The heatmap function in the ggplot2 package and Complex Heatmap package was used in the study to produce heatmap plots of DEGs. To allow for comparison of different data findings, the logFC transformation equation was used to normalize the expression values for each data point in each expression data condition [32]. The dataset was downloaded through the GEOquery package (2.54.1 version), using the surrogate variable analysis (SVA) package (3.34.0 version), LIMMA package (3.42.2 version), umap package (0.2.7.0 version) (UMAP analysis) [33], ggplot2 package (3.3.3 version) and Complex Heatmap package (2.2.0 version) to organize and analyze datasets. The data analysis process is shown in Figure 1.

### 2.3. Functional Enrichment Analysis

The online tool Database for Annotation, Visualization and Integrated Discovery (DAVID; https://david.ncifcrf.gov/ (accessed on 7 November 2021)) [34] was used to annotate the Gene Ontology (GO) enrichment analysis (http://amp.pharm.mssm.edu/Enrichr/ (accessed on 7 November 2021)) [35] of identified DEGs. The Kyoto Encyclopedia of Genes and Genomes (KEGG) Orthology-Based Annotation System (KOBAS; http://kobas.cbi.pku.edu.cn/kobas3 (accessed on 7 November 2021)) web-server was used to annotate and identify KEGG-enriched pathways [36]. Significant enrichment thresholds for GO and KEGG analyses were adjusted *p*-value < 0.05 and count ≥ 2.

### 2.4. Protein–Protein Interaction (PPI) Network Construction

The DEGs with opposite regulation obtained previously were mapped into Search Tool for the Retrieval of Interacting Genes/Proteins(STRING; www.string-db.org (accessed on 17 November 2021)) v11.5 [37]. A combined score of ±0.4 of PPI pairs was considered significant [38,39]. CytoScape (www.cytoscape.org/ (accessed on 17 November 2021); Institute for Systems Biology, Seattle, WA) was used to construct and visualize the network of DEGs with opposite regulation. “CytoHubba“ (a plugin of CytoScape) was used to identify the hub genes of the PPI network using three algorithms—Degree, Closeness Centrality, and Betweenness Centrality [38,40]. A Venn diagram was constructed and consisted of genes ranked in the top 20 of each method. There were 6 hub genes in all the three gene sets. A PPI network was constructed by CytoScape using the genes whose rank sum ranked in the top 20 of all and the most related genes in the STRING.

### 2.5. Mice and Surgery

C57BL/6J male mice (12–15 wk old, 24.0–34.0 g) for surgery were purchased from the Shanghai Branch of the National Rodent Laboratory Animal Resources (Shanghai, China). Animals were settled at 24 ± 2 °C under 12:12-h dark–light cycles. Performing animal experiments followed the National Institutes of Health Guide for the Care and Use of Laboratory Animals (no. 85-23, Revised 1996). The experimental protocol was ratified by the Animal Care and Use Committee of Zhongshan Hospital, Fudan University.

Transverse aortic constriction (TAC). Pressure overload was induced by TAC in 9 mice, according to methods we have previously described [41]. Mice were anesthetized by intraperitoneal injection of a mixture of ketamine (150 mg/kg) and xylazine (10 mg/kg), endotracheally intubated, and ventilated (type 7025, Harvard Apparatus, March-Hugstetten, Germany). After opening the chest cavity and isolating the transverse aorta, it was tied with a blunted 27-gauge needle between the innominate artery and left common carotid artery. Aortic constriction was yielded by removing the needle, followed by ligation with 6-0 silk. Subcutaneous meloxicam (0.13 mg each) was injected for pain relief. The corresponding sham-operated mice (sham; *n* = 6) underwent the same surgery without aortic constriction.

Aortic regurgitation (AR). VO was induced by AR, a developed mouse model for volume overload study. Under the guidance of ultrasound imaging, AR surgery was performed in 9 mice, according to methods we have previously reported [42,43]. As described above, after mice were anesthetized, a plastic catheter with wire was intercalated in the right common carotid artery. Next, the wire was pushed through the catheter to prick the aortic valves ended at significant diastolic retrograde flow in the aortic arch showed on the Doppler ultrasound. The catheter and the wire were withdrawn, followed by ligation with right common carotid artery. Meloxicam (0.13 mg each) was injected subcutaneously for analgesia. The corresponding sham-operated mice (sham; *n* = 6) underwent the same process without spoilage of the aortic valves.

### 2.6. RNA Isolation and Quantitative PCR Analysis

Total RNA was extracted from mouse cardiomyocytes using the TRIzol reagent (Ambion, #257401). Following the manual, PrimeScript TM RT Reagent Kit with gDNA Eraser (Takara, #RR047A) was applied to synthesize cDNA. On a Bio-Rad IQ5 multicolor detection system, quantitative real-time polymerase chain reaction (qRT-PCR) was executed by ChamQ Universal SYBR qPCR Master Mix (Vazyme, #Q711-02). The detection procedure was as follows: 5 min at 95 °C followed by 40 cycles of 20 s at 95 °C and 30 s at 60 °C. The results were analyzed in 2^−^^△△Ct^ method. The primer sequences are listed in Supplementary Table S8. For all analyses, a *p*-value < 0.05 was considered significant. All qRT-PCR data were expressed as the mean ± standard error of mean (SEM). Statistical analyses were performed using Graph Pad Prism (version 9.0.0).

## 3. Results

### 3.1. Identification of DEGs and Verification of qRT-PCR

**Figure 2:**
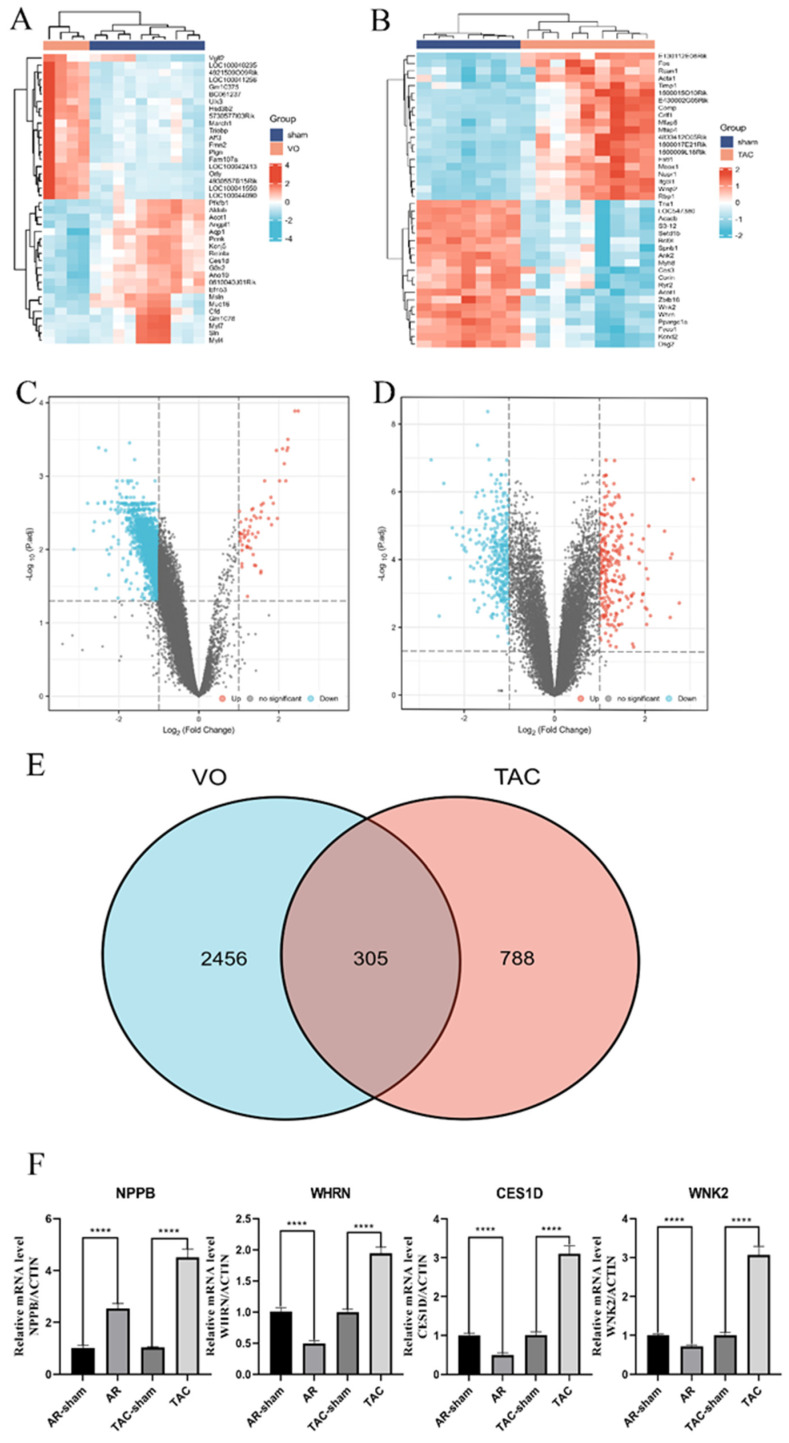
The volcano plot, heatmap, and qPCR of DEGs. The gradient color from blue to red represents the gene expression value ((**A**): VO group/sham group; (**B**): TAC group/sham group) from down-regulation to up-regulation, respectively. DEGs: differentially expressed genes. The volcano plot of DEGs: red and blue dots represent up-regulated and down-regulated genes, respectively. (**C**) is VO group vs. sham group; (**D**) is TAC group vs. sham group. (**E**) is the Venn analysis of VO and TAC. (**F**) is qPCR results of RNA from mice that received AR or TAC. ****: *p*-value < 0.0001.

**Figure 3:**
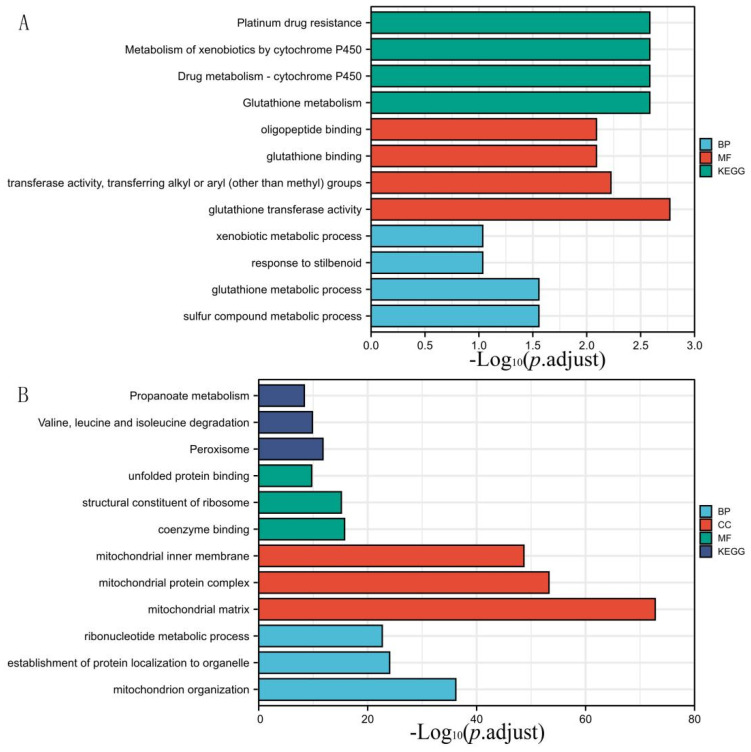
GO and pathway analysis of DEGs in VO group. (**A**) is an up-regulated differential gene, and (**B**) is a down-regulated differential gene. DEGs were divided into KEGG pathway and 3 functional groups, including BP, CC, and MF. KEGG: Kyoto Encyclopedia of Genes and Genomes; GO: Gene Ontology; BP: biological process; CC: cellular component; MF: molecular function; DEGs: differentially expressed genes.

In the VO group, compared with the control group, 2761 DEGs were identified, including 53 up- and 2708 down-regulated genes (Supplementary Sheet S1). A total of 40 DEGs were identified between the TAC group and the control group, including 594 up- and 499 down-regulated genes (Supplementary Sheet S2). Volcano plots and heatmaps of the identified DEGs can be observed in Figure 2 and Figure 3, respectively. To verify the authenticity of the sequencing data, cardiomyocytes from AR and TAC mouse models were used to extract RNA for qRT-PCR. Carboxylesterase 1D (*CES1D*), whirlin (*WHRN*), and WNK lysine deficient protein kinase 2 (*WNK2*) were the three genes with random absolute values of LogFC and indicators of heart failure (natriuretic peptide B, *NPPB*) detected. The gene expression of *CES1D*, *WHRN*, and *WNK2* decreased in the AR group and increased in the TAC group, which was consistent with the results in the database.

### 3.2. DEGs Co-Expression Results and Functional Enrichment Analysis

**Figure 4:**
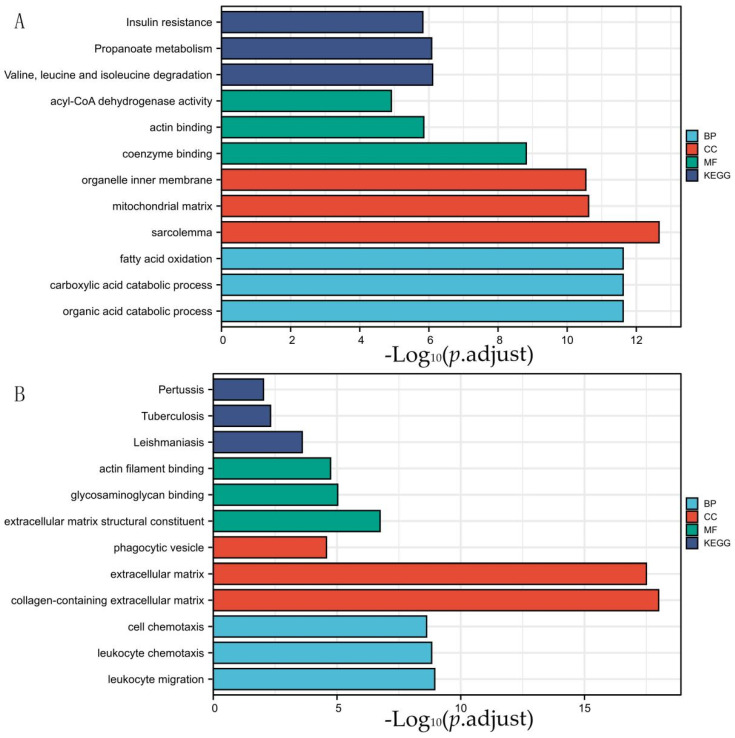
GO and pathway analysis of DEGs in PO group. (**A**) is the figure of up-regulated differential gene, and (**B**) is the figure of down-regulated differential gene. DEGs were divided into KEGG pathway and 3 functional groups, including BP, CC, and MF. KEGG: Kyoto Encyclopedia of Genes and Genomes; GO: Gene Ontology; BP: biological process; CC: cellular component; MF: molecular function; DEGs: differentially expressed genes.

Through Venn analysis, the DEGs in the VO and TAC groups discovered 305 genes. The down-regulated DEGs in VO were predominantly involved in cell component (CC) ontology, such as the mitochondrial matrix, mitochondrial inner membrane, mitochondrial protein complex, organelle inner membrane, and organellar ribosome, according to functional enrichment analysis. In terms of the biological process (BP), the down-regulated DEGs were significantly enriched in coenzyme binding. The molecular function (MF) analysis also showed that the down-regulated DEGs were primarily enriched in the fatty acid oxidation monocarboxylic acid catabolic process, sulfur compound metabolic process, cellular respiration, and energy derivation by oxidation of the organic compounds. Additionally, the KEGG pathway analysis of the up-regulated DEGs was found to be enriched in carbon metabolism, the citrate cycle (TCA cycle), peroxisome, propanoate metabolism, and valine, leucine, and isoleucine degradation (Figure 3 and Supplementary Tables S1 and S2). Compared with the VO group, in the TAC group, the up-regulated DEGs were primarily enriched in nine GO terms, including three BP terms (fatty acid oxidation, carboxylic acid, and organic acid catabolic process), three CC terms (organelle inner membrane, mitochondrial matrix, and sarcolemma), and three MF term (acyl-CoA dehydrogenase activity, actin binding, and coenzyme binding; Figure 4 and Supplementary Tables S3 and S4). Furthermore, the DEGs that were elevated were significantly abundant in five KEGG pathways, including fatty acid degradation, dilated cardiomyopathy, hypertrophic cardiomyopathy, propanoate metabolism, and valine, leucine, and isoleucine degradation.

**Figure 5:**
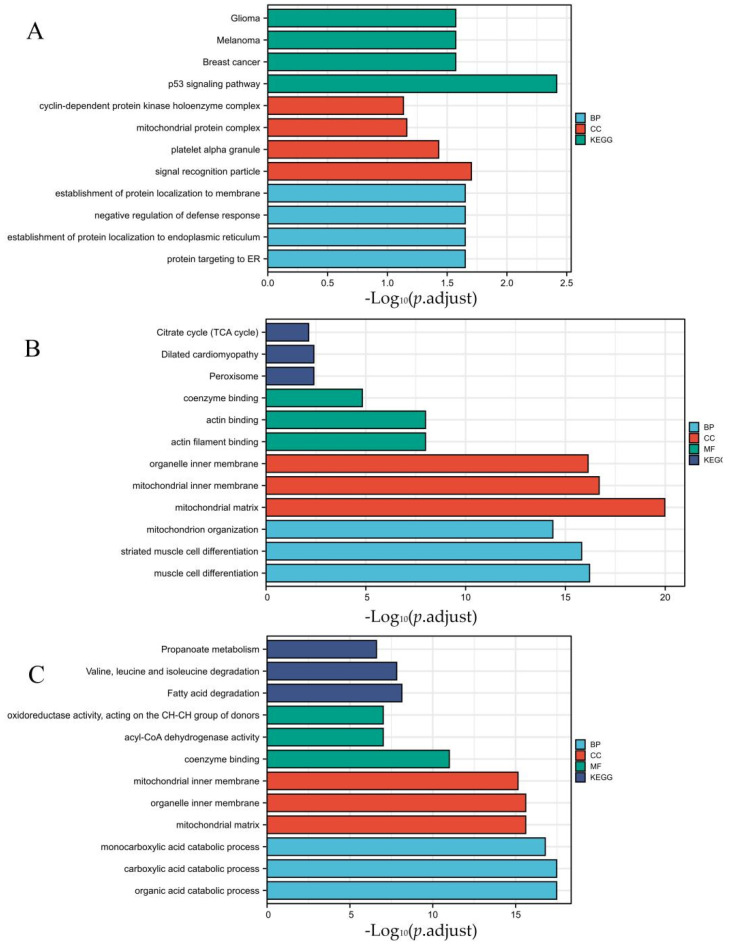
GO and pathway analysis of DEGs in VO and TAC groups with same regulation, opposite regulation, and no interaction. DEGs were divided into two functional groups, including BP and CC. GO: Gene Ontology; BP: biological process; CC: cellular component; KEGG: Kyoto Encyclopedia of Genes and Genomes; DEGs: differentially expressed genes. (**A**) is GO analysis of DEGs with same regulation. (**B**) is GO analysis of DEGs with opposite regulation. (**C**) is GO analysis of DEGs with no interaction.

Then, the 305 DEGs obtained in the Venn analysis were divided into two groups for enrichment analysis. Among them, genes with the same gene expression changes were grouped into one group (Figure 5A), and genes with opposite gene expression changes were grouped into another group (Figure 5B). In Figure 5A, the DEGs were enriched in 12 GO terms, including 4 BP terms (p53 signaling pathway and melanoma), 4 CC terms (signal recognition particle and platelet α granule), and 4 KEGG pathways (negative regulation of defense response and the establishment of protein localization to the endoplasmic reticulum; Figure 5A and Supplementary Table S5). In Figure 5B, the DEGs with opposite changes were significantly enriched in three KEGG pathways, including fatty acid degradation, propanoate metabolism, and valine, leucine, and isoleucine degradation. Moreover, the DEGs were enriched in nine GO terms, including three BP terms (acyl-CoA dehydrogenase activity and oxidoreductase activity, acting on the CH-CH group of donors), three CC terms (mitochondrial and organelle inner membrane), and three MF term (monocarboxylic acid, carboxylic acid, and organic acid catabolic process; Figure 5B and Supplementary Table S6). Finally, DEGs with no intersection between VO and TAC were used for enrichment analysis in Figure 5C (Supplementary Table S7).

### 3.3. Protein–Protein Interaction (PPI) Network

**Figure 6:**
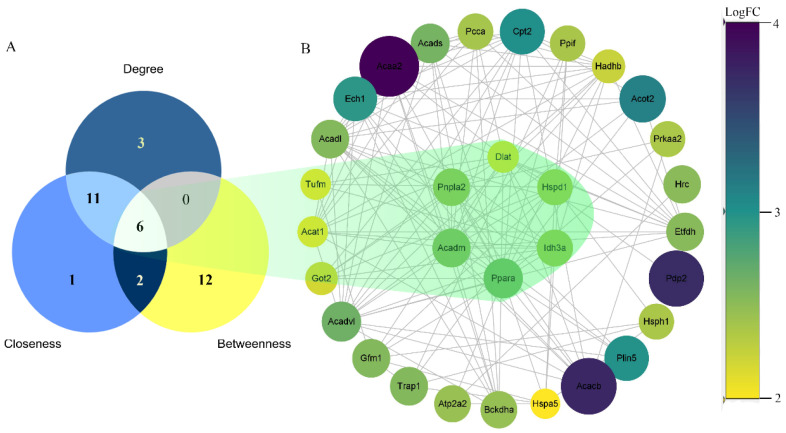
PPI network. (**A**) is Venn analysis with three algorithms. (**B**) is PPI network construction. Each circle represents a gene node. The transition from yellow to purple and the changes in the diameter of the circle indicate an increase in the sum of the absolute values of logFC. The genes with the 6 circles in the center represent the 6 genes with the intersection of the three algorithms.

CytoScape software was used to build a PPI network in order to identify key genes. As the logFC of the gene grew, the hue of the concentric circles darkened from yellow to purple. Figure 6A depicts the Venn diagram of the three algorithms. Figure 6B depicts the top six gene nodes, which include peroxisome proliferator-activated receptor α (*PPARα*), acyl-CoA dehydrogenase medium chain (*ACADM*), patatin-like phospholipase domain containing 2 (*PNPLA2*), isocitrate dehydrogenase 3α (*IDH3α*), heat shock protein family D member 1 (*HSPD1*), and dihydrolipoamide S-acetyltransferase (*DLAT*). When combined with the results of the GO analysis, these genes are mostly involved in controlling the body’s fatty acid metabolism, potassium ion transport, and cell proliferation.

## 4. Discussion

We employed bioinformatics tools to examine and combine two publicly available microarray data sets in this study. To uncover distinct genes, we compared the VO and PO model data with their respective control groups. The two groups of differential genes were then compared again, and it was discovered that the expression of 53 genes in the VO group was up-regulated compared to the PO model data, while the expression of 2708 genes was down-regulated. Among them, there are significant differences in the expression of *PPARα*, *ACADM*, *PNPLA2*, *IDH3α*, *HSPD1*, and *DLAT*, and the function research of these genes in VO has not been paid attention to.

*PPARα* is expressed in the heart, kidney, and liver [44,45], with differing protein and mRNA expression patterns in humans and other animals [46]. *PPARα* is implicated in vascular damage, cardiac disease, hypertension, and lipid disorders [47]. Among them, *PPARα* was identified as a transcriptional regulator of the production and activity of endogenous vasoconstrictors and their receptors, which may induce them to attenuate the vasoconstriction response to major endogenous vasoconstrictors such as angiotensin II (Ang II) [48,49]. Studies have reported that *PPARα* can protect the heart by resetting the renin–angiotensin system (RAS) to control blood pressure [49,50]. In addition, *PPARα* participates in mitochondrial-mediated energy metabolism [51] and can also regulate the synthesis of very low-density lipoproteins to improve blood lipids [52]. In many clinical studies, *PPARα* has also been linked to the start of cardiovascular disease, atherosclerotic alterations, and hypertension in numerous clinical trials [53,54,55,56]. In the current research, the exploration and clinical intervention of *PPARα* also focuses on PO. The expression of *PPARα* is observed to be down-regulated in the VO model, and the heart tissue loses its ability to regulate blood pressure and blood vessel damage under the condition of VO, which is also consistent with previous research reports.

*PNPLA2* is a critical gene in the process of energy metabolism that encodes a protein that is required for intracellular triglyceride (TG) breakdown. *PNPLA2* mutations can induce severe lipodystrophy, which can lead to severe cardiomyopathy due to an abnormal energy source [57,58,59]. In mouse experiments, if there is a homozygous missense mutation of *PNPLA2* (c.245G> A, p.G82D), arrhythmia and obvious cardiac dysfunction will occur [60]. Pathological analysis of the mice revealed that the fat in the myocardial cells of the animals had increased, as had the fibrous alterations in the myocardium. We hypothesize that the persistent expression of *PNPLA2* aids myocardial cells in hydrolyzing TG, hence lowering fat formation and alleviating cardiac fibrosis.

*HSPD1* is a gene encoding mitochondrial protein, which is involved in the folding and assembly of newly imported proteins in mitochondria [61]. This gene has been associated to atherosclerosis and has been linked to inflammatory response [62,63]. According to research, anti-heat-shock protein 60 or the down-regulation of this gene can worsen atherosclerosis [62,64]. It has also been linked to the aggravation of HF by inducing cell death via the toll-like receptor (TLR)-4 [42]. The foregoing findings are likewise compatible with the down-regulation state of *HSPD1* as shown by our VO data. When VO occurs, the down-regulation of *HSPD1* causes the immune system to worsen vascular injury and promotes cardiomyocyte death in the state of HF.

*ACADM* is linked to the etiology of medium chain acyl-CoA dehydrogenase deficit (*MCADD*) [65,66,67] and is primarily engaged in the mitochondrial fatty acid-oxidation pathway, which affects the body’s energy metabolism. In the VO model, we hypothesize that the gene modulates mitochondrial activity, which then affects cell energy supply, resulting in a sequence of pathological alterations. *IDH3* is highly expressed in the heart tissue in available human data [68,69], although the existing research results are associated to malignancies and retinopathy [70,71,72]. Our research discovered that *IDH3* has a considerable down-regulation state in the VO model, providing us with a viable research target; nevertheless, additional trials are required to validate the statistical data. *DLAT* (also known as E2; PBC; PDCE2; PDC-E2) is also a gene that is highly expressed in heart tissue but has not been paid attention to in heart disease research. The protein product of *DLAT*, dihydrolipoamide acetyltransferase, accepts the acetyl group formed by the oxidative decarboxylation of pyruvate and transfers it to coenzyme A. Studies have reported that it is closely related to the pathogenesis of primary biliary cirrhosis (PBC), an autoimmune liver disease [73]. However, combined with the results of its tumor research [74,75], we speculate that this gene affects the heart’s adaptive changes to VO by participating in the regulation of cell proliferation, and basic experimental data are still needed to support our conjecture.

Recently reported findings from human myocardial samples revealed that angiotensin converting enzyme 2 (ACE2) was considerably up-regulated in PO but not in VO [76]. However, in our original dataset, there are no significant changes in ACE2 in either the TAC or PSPI groups. Previous rat hypertension models revealed that ACE2 was significantly reduced at both the gene and protein levels, therefore whether ACE2 is raised in the PO state remains debatable [77]. On the one hand, the data which reported ACE2 up-regulated in PO come from severe aortic stenosis (AS). Whether all the other pressure load conditions also lead to increased ACE2 expression is unknown. On the other hand, we assume that due to changes in sample sources, mouse samples did not reveal substantial differences [78].

By degrading Ang II to angiotensin (1-7), ACE2 performs a crucial anti-inflammatory and anti-fibrotic role in RAAS [79], which can ease the process of cardiac insufficiency and cardiac hypertrophy [80]. Previous research has also discovered that ACE2 is a functional host receptor for COVID-19 [81,82]. COVID-19 reduces the expression of p53 after entering the host cells, causing a homeostasis imbalance [83]. Our KEGG enrichment analysis revealed that the expression of differential genes in the p53 signaling pathway was similar among the genes altered in the same direction by PO and VO, providing a good reference for our treatment. The differential expression genes in our pathway analysis were cyclin G1, cyclin dependent kinase 4, growth arrest and DNA damage inducible α, and insulin like growth factor 1, all of which were down-regulated to varying degrees and were linked to cell cycle and DNA damage [84,85]. Activation of p53 has been shown in vivo and in vitro to up-regulate the RAS [86,87], yet, down-regulation of the p53 signaling pathway in cardiovascular illness can disrupt the angiotensin I converting enzyme (ACE)/ACE2 balance. ACE2 exerts a core regulatory role in RAAS. When the p53 signaling pathway is suppressed to down-regulate RAS and ACE is blocked, RAAS is down-regulated by ACE2 [79]. Consider that identical gene expression alterations were observed in both PO and VO, which may explain why individuals with a history of cardiovascular disease have a higher risk of death when infected with COVID-19 [88].

However, there are certain limitations to this study. The first is that the sample’s source is limited, and there are no human-sourced data accessible for comparison in the existing studies. Second, because VO and PO have distinct detection time points, existing study data cannot give more detection time points, and we cannot analyze how the differential genes of the two will evolve over time. Finally, in vivo and in vitro trials are required to validate our findings when paired with existing experimental study results.

## 5. Conclusions

In summary, the expression of *PPARα*, *ACADM*, *PNPLA2*, *IDH3α*, *HSPD1*, and *DLAT* in the VO model differs considerably from that in the PO model and could be employed as a biomarker for the diagnosis and treatment of VO. Additionally, *PPARα*, *ACADM* and *PNPLA2* may also have a role in the regulation of the development and prognosis of VO via the fatty acid metabolism pathway and acyl-Coa dehydrogenase activity.

## Figures and Tables

**Table 1:** Basic information of GEO datasets used in the study.

GSE Series	Type	Sample Size			Platform
		Control	Pulmonary Insufficiency and Stenosis	Transverse Aortic Constriction	
GSE97363	mRNA	10	4		GPL13912
GSE52796	mRNA	3		5	GPL6887

## Data Availability

Research data can be obtained by contacting the corresponding author.

## Supplementary Materials

The following supporting information can be downloaded at: https://www.mdpi.com/article/10.3390/genes13071276/s1, Table S1: GO analysis of DEGs in VO group of up-regulated differential gene; Table S2: GO analysis of DEGs in VO group of down-regulated differential gene; Table S3: GO analysis of DEGs in TAC group of up-regulated differential gene; Table S4: GO analysis of DEGs in TAC group of down-regulated differential gene; Table S5: GO analysis of DEGs in VO and TAC groups with same regulation; Table S6: GO analysis of DEGs in VO and TAC groups with opposite regulation; Table S7: GO analysis of DEGs in VO and TAC groups with no interaction; Table S8: Primer sequences; Sheet S1: The differentially expressed genes (DEGs) of volume overload (VO); Sheet S2: The differentially expressed genes (DEGs) of pressure overload (PO).
