# Supplementary material for: Identification of Differential Expression Genes between Volume and Pressure Overloaded Hearts Based on Bioinformatics Analysis
Source: Genes (Basel). 2022 Jul 19;13(7):1276. doi: 10.3390/genes13071276 (PMC9318830; doi:10.3390/genes13071276)
Supplement: Supplementary file 1 [file genes-13-01276-s001.zip › genes-1739099-supplementary.pdf]

Sheet S1. The differentially expressed genes (DEGs) of volume overload (VO)

ID	LogFC	AveExpr	t	p.Value	adj.p.Val	B	Gene.Symbol
28963	2.485618	7.134974	10.84387	7.23E-09	0.000129	10.22246	Gm10375
34207	2.40279	7.250885	10.48338	1.17E-08	0.000129	9.808189	LOC100041256
40268	2.2274	6.949047	9.566585	4.29E-08	0.000315	8.68359	BC061237
34310	2.226851	7.230612	8.928314	1.11E-07	0.000409	7.836293	LOC100041550
11774	2.203637	12.72265	8.597987	1.86E-07	0.000445	7.37574	Hsd3b2
7715	2.175901	7.122512	7.586846	9.72E-07	0.001159	5.867157	LOC100044090
13532	2.137786	7.070263	8.170703	3.68E-07	0.000676	6.756772	Orly
59366	2.094507	7.287153	8.806436	1.34E-07	0.000423	7.668158	LOC100040235
51382	2.019994	9.844755	7.657062	8.63E-07	0.001159	5.976878	Pign
22100	1.965515	10.29	5.656941	3.32E-05	0.003769	2.552725	Ulk3
58659	1.862676	8.458082	6.46711	7.09E-06	0.002346	4.014119	Fmn2
19567	1.74471	10.67976	5.656931	3.32E-05	0.003769	2.552707	Triobp
12857	1.612315	6.875448	6.467791	7.09E-06	0.002346	4.015304	LOC100042413
19886	1.563811	8.841558	5.90946	2.03E-05	0.003048	3.018753	Aff3
46281	1.563493	10.08237	6.335107	9.07E-06	0.002463	3.7828	Fam107a
39015	1.554225	8.520828	3.5088	0.002839	0.019696	-1.68939	Gsta1
39268	1.548705	9.152994	3.451265	0.003209	0.020878	-1.80536	Gm10639
32308	1.538695	7.244491	5.731818	2.87E-05	0.003679	2.691876	Sly
8107	1.480945	7.905235	4.941357	0.00014	0.005859	1.184809	Slc35e2
61762	1.441614	7.27396	5.699028	3.06E-05	0.003721	2.631038	LOC380994
9509	1.424385	8.690114	3.659742	0.002058	0.016573	-1.38434	Csprs
34119	1.423561	9.606183	4.720611	0.00022	0.006777	0.750467	E330020D12Rik
43085	1.393177	7.086246	3.672428	0.002003	0.016353	-1.35866	Syt13
50954	1.383904	7.366297	5.775059	2.64E-05	0.003483	2.771868	Sfi1

25423	1.359101	6.942301	4.034354	0.000928	0.011717	-0.62558	LOC100042435
53536	1.354383	6.858607	6.201055	1.17E-05	0.002765	3.545143	Gm5800
50852	1.339991	7.043056	4.840153	0.000172	0.006133	0.986311	LOC100039675
38206	1.325504	6.954495	6.043731	1.57E-05	0.002865	3.262731	Olfr39
30502	1.302481	6.82542	5.528375	4.28E-05	0.00399	2.311948	LOC100040022
60376	1.295314	8.621765	4.281225	0.00055	0.009575	-0.12717	Pdik1l
22190	1.275895	7.237738	5.25486	7.39E-05	0.004874	1.792208	Pgbd1
30951	1.244864	8.520059	4.553125	0.000311	0.007598	0.417745	Slfn9
7386	1.236113	6.670749	5.050095	0.000112	0.005573	1.396813	LOC100041207
18820	1.224225	7.146517	4.846886	0.000169	0.00613	0.99955	Acot3
28124	1.217282	8.465396	2.858454	0.011219	0.043523	-2.98001	S100a9
55968	1.204884	11.67552	4.362106	0.000464	0.009	0.035457	Slc8a3
35366	1.200904	11.63511	4.322799	0.000504	0.009325	-0.04353	Lhx6
14514	1.183372	6.537453	5.629589	3.51E-05	0.003857	2.501696	LOC100040395
22853	1.159639	8.620832	4.769055	0.000199	0.006532	0.846215	Scd3
39210	1.136947	12.91978	5.076922	0.000106	0.005504	1.448905	Snap25
32149	1.136003	14.64699	4.69154	0.000233	0.006897	0.692898	H2-Gs10
19990	1.111557	7.13628	4.82526	0.000177	0.006166	0.957007	Tssk5
35051	1.087513	11.3312	4.40453	0.000425	0.008651	0.120589	Pdzrn3
61748	1.078852	7.451942	4.193316	0.000663	0.010247	-0.30434	Gm3161
4177	1.078389	7.443118	3.620173	0.002239	0.017286	-1.46439	Gsta2
8795	1.076142	12.24255	3.709064	0.001853	0.01565	-1.28449	Krt222
37573	1.047134	13.10303	4.835015	0.000174	0.006133	0.976205	Idua
8332	1.046578	6.668707	5.456602	4.94E-05	0.004211	2.176534	Gm9376
11084	1.041758	13.11104	4.537105	0.000322	0.007678	0.385791	Fgf23
28749	1.034209	6.640172	4.680853	0.000239	0.006939	0.671716	LOC100503215

57420	1.034086	12.96086	4.85714	0.000166	0.006078	1.019706	Slc17a5
20641	1.01854	6.750123	4.909848	0.000149	0.005914	1.123129	BC051408
45400	1.014512	6.748313	5.93048	1.95E-05	0.003017	3.057127	LOC677484
5489	-1.00004	10.41866	-3.11631	0.00653	0.031072	-2.47488	Rcor1
57339	-1.00007	12.1609	-3.7083	0.001856	0.01565	-1.28603	Pcbp2
61555	-1.0001	7.201149	-4.60336	0.00028	0.007327	0.517796	Spna1
48134	-1.0001	11.03788	-3.24265	0.004999	0.02661	-2.22372	Ube2j1
60793	-1.00016	12.25743	-3.07085	0.007188	0.032953	-2.56472	Clcn4-2
28010	-1.00019	11.20422	-2.99814	0.008376	0.036082	-2.70777	Wdr61
30512	-1.00042	8.62537	-3.66073	0.002054	0.016562	-1.38234	Ogfrl1
17268	-1.00044	8.758121	-3.73426	0.001756	0.015297	-1.23347	Rpia
33667	-1.00059	8.05251	-3.95049	0.001108	0.012525	-0.79537	Gfra2
27731	-1.00081	12.73294	-3.45692	0.00317	0.020734	-1.79398	Cops5
55774	-1.00082	11.36153	-4.18962	0.000668	0.010268	-0.31179	Prkag1
51711	-1.001	10.28609	-3.25757	0.004843	0.026079	-2.19394	Txndc9
9337	-1.00104	12.48171	-3.08341	0.007	0.032444	-2.53994	Camk2d
27524	-1.00123	14.83308	-3.69754	0.001899	0.015846	-1.30782	Gm15501
28424	-1.00124	11.58863	-3.71899	0.001814	0.015538	-1.26439	Mrps12
20294	-1.00143	10.28709	-3.46575	0.003111	0.020589	-1.77619	Rapgef2
24048	-1.00149	8.959143	-3.23458	0.005085	0.026878	-2.23983	Ptplb
42871	-1.00164	10.34749	-3.48103	0.003012	0.020295	-1.74539	Dhx16
4174	-1.00164	10.36094	-3.26533	0.004764	0.025854	-2.17845	Lrrc45
58551	-1.00167	11.7471	-3.59751	0.00235	0.017719	-1.51022	Nkiras2
30774	-1.00187	11.3931	-3.04519	0.007587	0.034024	-2.61532	Edc4
27885	-1.00222	12.75387	-3.89019	0.00126	0.013239	-0.91752	Dnm2
18185	-1.00228	12.22668	-3.29089	0.004512	0.025161	-2.12734	Irf3

16731	-1.00231	9.383377	-3.61015	0.002288	0.017493	-1.48467	Bcl2l12
58968	-1.00232	13.67185	-4.09334	0.000819	0.011164	-0.50626	Rps3
8959	-1.00233	11.19793	-2.95888	0.009095	0.038042	-2.78466	Taf9
37762	-1.00236	10.72341	-3.51105	0.002825	0.019649	-1.68485	Hmgxb3
55886	-1.00267	15.20648	-4.97069	0.000131	0.005815	1.24214	Brp44l
55875	-1.00276	10.71341	-2.84005	0.011657	0.044527	-3.01559	Unc50
49422	-1.00277	7.846938	-4.31406	0.000514	0.009341	-0.0611	Pkd2l2
12239	-1.00306	10.66587	-2.99769	0.008384	0.036087	-2.70867	Lsm3
41165	-1.00307	10.12392	-3.2625	0.004793	0.025958	-2.1841	Gtf2a1
8659	-1.00311	12.77165	-3.19584	0.00552	0.028147	-2.31702	Larp1
12403	-1.00321	13.23238	-2.75418	0.013932	0.049965	-3.18063	Wnk1
8297	-1.00342	9.309549	-3.69141	0.001924	0.015958	-1.32023	Phf23
26696	-1.00361	9.796461	-3.21661	0.005282	0.027481	-2.27566	Snrpa1
52119	-1.00364	10.64504	-3.43337	0.003333	0.02129	-1.8414	Orc3
4727	-1.00375	7.668743	-3.71499	0.00183	0.015573	-1.27249	Ccnjl
58616	-1.00387	8.927197	-2.80364	0.012574	0.046622	-3.08578	Snai2
26054	-1.00392	10.09862	-3.09832	0.006783	0.031869	-2.51046	Wdr55
24350	-1.00393	14.04556	-3.73867	0.00174	0.015236	-1.22453	Fem1a
11277	-1.00403	9.835679	-3.08101	0.007035	0.032533	-2.54467	Nup35
46484	-1.00419	13.78956	-4.53931	0.00032	0.007678	0.39018	Naca
15384	-1.0043	9.254276	-3.24733	0.004949	0.026398	-2.21439	Jarid2
52647	-1.00435	12.60636	-3.52751	0.002728	0.019251	-1.65162	Rpl5
54864	-1.00444	14.40843	-4.54216	0.000319	0.007663	0.395872	Prdx2
47690	-1.00478	11.46651	-3.36126	0.003886	0.02303	-1.98633	Tpd52l2
21131	-1.00478	13.03472	-3.6217	0.002232	0.017264	-1.46131	Pnpla2
42671	-1.00479	11.40141	-3.37859	0.003745	0.022702	-1.95153	Paics

49226	-1.00492	10.08394	-3.86079	0.001341	0.013636	-0.9771	Wiz
4224	-1.00493	11.01002	-3.58355	0.002421	0.018068	-1.53844	Esrrb
8129	-1.00496	9.282204	-3.15395	0.006031	0.029664	-2.40027	Brwd1
58633	-1.00504	8.10602	-4.9573	0.000135	0.005828	1.215976	Ybey
14113	-1.00519	7.96405	-4.07863	0.000845	0.011283	-0.53602	Atp10d
27405	-1.00537	9.206938	-3.62367	0.002223	0.017248	-1.45733	Ddr1
25152	-1.00549	8.671212	-4.34334	0.000483	0.00913	-0.00225	Rnaseh1
35967	-1.00552	10.3344	-3.28131	0.004605	0.025433	-2.14651	Sbno1
7519	-1.00567	9.822577	-2.99146	0.008494	0.036392	-2.72088	Zdhhc6
28206	-1.00568	10.38124	-2.87586	0.010819	0.042549	-2.94629	Caprin1
23341	-1.0057	8.818062	-3.74663	0.00171	0.015124	-1.20841	Mlx
2331	-1.0058	12.70405	-3.92284	0.001176	0.012879	-0.85138	Gm5614
50363	-1.00601	8.901024	-4.03941	0.000918	0.011676	-0.61536	Zmym5
34900	-1.00619	9.797914	-3.42128	0.00342	0.021617	-1.86572	Fuk
26172	-1.00622	8.579675	-3.78546	0.001575	0.014645	-1.12975	Sel1l
58513	-1.00633	8.573558	-3.13664	0.006256	0.030356	-2.4346	Runx1t1
27629	-1.00636	11.51649	-3.15566	0.006009	0.029609	-2.39687	Serbp1
22009	-1.00653	12.91794	-3.46099	0.003143	0.020649	-1.78578	Rnpepl1
18244	-1.0066	10.51754	-3.17757	0.005737	0.028836	-2.35335	Spata2
54747	-1.00668	9.279596	-3.8064	0.001506	0.014368	-1.08731	Slc38a3
11525	-1.00673	13.66329	-3.38283	0.003712	0.02261	-1.94301	Vps28
24940	-1.00678	8.313235	-3.92621	0.001167	0.012862	-0.84456	Rftn2
7872	-1.00679	12.61379	-2.94014	0.009459	0.038937	-2.82125	Sdf2
42334	-1.00687	9.464268	-3.35627	0.003927	0.023168	-1.99635	Zw10
40409	-1.00694	9.128788	-3.35017	0.003979	0.02337	-2.00858	Zfp217
61529	-1.00697	12.63195	-3.83859	0.001406	0.013923	-1.02208	Nedd8

42842	-1.00709	8.519064	-4.03237	0.000932	0.011726	-0.6296	Thrb
19581	-1.00709	11.48685	-3.4051	0.00354	0.021965	-1.89826	Eps15l1
52161	-1.00712	9.472674	-3.68182	0.001964	0.016154	-1.33964	Zfp64
32243	-1.00712	14.40907	-3.71375	0.001834	0.015584	-1.27501	Rps26
21986	-1.00724	10.10017	-3.64929	0.002105	0.016774	-1.40548	Isy1
37491	-1.00729	9.106517	-3.47892	0.003025	0.020334	-1.74964	Ints10
44161	-1.00732	8.728616	-3.78495	0.001576	0.014645	-1.13077	Zfp422
2820	-1.00753	10.77996	-3.60971	0.00229	0.017497	-1.48555	Sac3d1
58544	-1.00757	8.946141	-3.7413	0.00173	0.01522	-1.21919	Egln1
28937	-1.0076	8.131939	-3.92281	0.001176	0.012879	-0.85143	Hat1
2550	-1.00762	13.77817	-3.31739	0.004266	0.024348	-2.07429	Nudt3
42896	-1.00762	11.01517	-2.89718	0.010348	0.041204	-2.90491	Pcgf5
2025	-1.00769	9.161692	-3.89586	0.001245	0.013205	-0.90605	Unc119
57867	-1.00777	10.80721	-3.7908	0.001557	0.014594	-1.11892	Mylk3
55317	-1.00788	9.031705	-3.71838	0.001816	0.015541	-1.26563	Tmem136
17902	-1.00792	10.5555	-2.94178	0.009426	0.038863	-2.81805	Uchl4
46292	-1.0081	14.78367	-4.56504	0.000304	0.007532	0.441499	Gm10653
12374	-1.0082	10.38969	-2.98321	0.008642	0.036742	-2.73704	Klhl13
10015	-1.00828	8.995163	-3.9074	0.001215	0.013117	-0.88267	Mkln1
378	-1.00845	9.433612	-3.88918	0.001263	0.013239	-0.91957	Acvr1b
22774	-1.00846	10.94887	-3.58935	0.002391	0.017889	-1.52672	Raly
30392	-1.00847	12.66109	-4.03773	0.000921	0.011697	-0.61876	Bsg
26713	-1.00901	11.87592	-2.97997	0.008701	0.036914	-2.7434	Sec62
27561	-1.00908	10.59254	-3.68521	0.001949	0.016103	-1.33278	Pex13
22842	-1.00932	10.62904	-3.44408	0.003258	0.021052	-1.81983	Utp11l
23711	-1.0095	12.19936	-3.24758	0.004947	0.026397	-2.21388	Dbndd2

57166	-1.00953	13.62563	-4.02041	0.000956	0.011851	-0.6538	Gm16477
54489	-1.00985	10.58481	-3.95478	0.001098	0.012517	-0.78668	Ttc4
28381	-1.00991	10.00014	-3.20651	0.005397	0.027769	-2.29578	Snap29
58366	-1.00997	10.67759	-3.36805	0.00383	0.022899	-1.97269	Gm16515
26304	-1.01	8.037406	-4.42988	0.000403	0.00847	0.171407	Ppp2r3a
3696	-1.01002	9.504922	-3.39967	0.003581	0.02212	-1.90918	Gmnn
4111	-1.01007	13.80631	-4.44724	0.000388	0.008335	0.206176	Rhot2
20402	-1.0103	9.990238	-3.88002	0.001288	0.013363	-0.93813	Wrap53
7198	-1.01055	8.820898	-3.39433	0.003622	0.022279	-1.9199	D030016E14Rik
55381	-1.01072	10.36805	-3.16004	0.005954	0.029457	-2.38817	Fbxo38
30918	-1.01084	13.23826	-3.34691	0.004006	0.023439	-2.01512	Rps7
29394	-1.01087	10.29778	-4.47102	0.00037	0.00812	0.253767	Gm13141
14923	-1.01091	9.356912	-3.44641	0.003242	0.020997	-1.81514	Maml1
29343	-1.01103	9.669433	-3.11871	0.006497	0.031022	-2.47013	Cmtm7
11372	-1.01104	12.36923	-3.30152	0.004412	0.024829	-2.10608	Rps6kb1
16001	-1.01112	9.550411	-3.97018	0.001063	0.012344	-0.7555	Acot8
47219	-1.01138	8.884072	-3.76407	0.001648	0.014898	-1.17308	Sike1
31914	-1.01163	12.23663	-3.55762	0.002558	0.018579	-1.59084	Smpx
60033	-1.01165	8.994768	-5.03434	0.000115	0.005645	1.366188	Gpsm1
53014	-1.01179	10.5664	-3.07512	0.007123	0.032767	-2.5563	Cops2
50942	-1.0119	9.466914	-3.16757	0.00586	0.029223	-2.37322	Phactr1
12645	-1.01199	9.157721	-4.65435	0.000252	0.007106	0.619148	LOC100504758
31891	-1.01203	10.29994	-2.7784	0.01325	0.048261	-3.13425	Glis2
31844	-1.01204	9.074584	-4.28733	0.000543	0.009554	-0.11487	Mgat4b
40558	-1.01218	11.52301	-3.42874	0.003366	0.021421	-1.85071	Herc1
15976	-1.01226	12.4967	-3.36564	0.00385	0.022932	-1.97753	Fbxo6

28195	-1.01227	9.888155	-3.53283	0.002697	0.019083	-1.64088	Srek1ip1
23039	-1.0123	11.32023	-2.9106	0.010062	0.040504	-2.87881	Inpp5j
61214	-1.0125	10.9543	-3.3537	0.003949	0.023245	-2.00149	Trp53i13
31547	-1.01253	9.232569	-3.66793	0.002023	0.016409	-1.36778	Stk16
9351	-1.01259	10.39443	-3.78815	0.001566	0.014632	-1.1243	Lztr1
39002	-1.01271	10.01819	-3.32125	0.004231	0.024256	-2.06656	Mll3
46521	-1.01276	11.27271	-3.92274	0.001176	0.012879	-0.85158	Ppp2ca
53545	-1.01277	9.148525	-3.29213	0.004501	0.025137	-2.12486	Krit1
27484	-1.01279	9.262006	-3.7649	0.001645	0.014895	-1.1714	Pex5
14626	-1.01284	9.89107	-4.41583	0.000415	0.008576	0.143241	Gorasp1
16730	-1.01292	15.01651	-4.60543	0.000279	0.007327	0.521911	Pfkm
49805	-1.01305	8.599519	-3.81392	0.001482	0.014282	-1.07206	Zfp933
30293	-1.01313	9.778143	-3.20945	0.005363	0.027693	-2.28992	Cramp1l
15891	-1.0132	9.077275	-3.48163	0.003008	0.02028	-1.74419	Tmem201
22736	-1.01337	9.574267	-3.45654	0.003173	0.020745	-1.79474	Myst3
35796	-1.01345	10.87369	-3.1853	0.005644	0.028518	-2.33798	Emp2
60000	-1.01349	7.767039	-3.63129	0.002187	0.017104	-1.4419	Lrp4
19551	-1.01362	10.56468	-3.09774	0.006791	0.031887	-2.51161	Ralbp1
60028	-1.01375	9.432731	-4.38972	0.000438	0.008803	0.09089	Kcna7
5841	-1.01394	9.206268	-3.16716	0.005865	0.029235	-2.37404	Akap11
19760	-1.01409	9.634136	-3.85253	0.001365	0.013751	-0.99384	Fgf9
6851	-1.01447	12.62811	-3.61518	0.002263	0.017388	-1.47448	Gcsh
17574	-1.01447	10.36329	-3.46116	0.003142	0.020649	-1.78544	Zdhhc16
24855	-1.01457	12.36602	-3.72296	0.001799	0.015473	-1.25636	Tspan7
47885	-1.01462	10.03222	-3.46747	0.0031	0.020535	-1.77272	Sf3a1
10677	-1.01472	9.353273	-3.84631	0.001383	0.013852	-1.00644	Abcc10

9656	-1.01477	11.35984	-3.16043	0.005949	0.029452	-2.38741	Mib2
60585	-1.01484	12.26038	-4.16055	0.00071	0.010492	-0.37047	Stoml2
9541	-1.01489	11.77719	-3.2674	0.004743	0.025814	-2.1743	Lap3
48802	-1.01506	11.86639	-3.28898	0.004531	0.025185	-2.13116	Rer1
38334	-1.0151	9.987634	-3.47243	0.003067	0.020469	-1.76273	Zfp513
33726	-1.0151	11.78924	-3.22013	0.005243	0.02739	-2.26864	Lars
61247	-1.01522	8.943135	-3.29842	0.004441	0.02491	-2.11227	Gmcl1
62590	-1.0153	9.172898	-3.70421	0.001872	0.015712	-1.29432	Prkra
59658	-1.01531	11.91608	-3.07424	0.007136	0.032805	-2.55803	Fam40b
16042	-1.0154	12.51975	-3.43617	0.003314	0.021217	-1.83575	Numa1
1845	-1.01592	15.47211	-4.85415	0.000167	0.006078	1.013825	Oaz1
9573	-1.01618	9.346154	-3.2306	0.005128	0.027031	-2.24777	Kcnj8
43414	-1.0165	9.392396	-2.84217	0.011606	0.044422	-3.01149	Dok4
19946	-1.01659	10.18761	-3.26902	0.004727	0.025804	-2.17108	Rars2
30358	-1.0168	13.23067	-3.32853	0.004166	0.024009	-2.05196	Arl2bp
18175	-1.01681	10.50074	-3.55294	0.002584	0.018673	-1.60029	Rps19bp1
51679	-1.01681	9.561728	-3.95224	0.001104	0.01252	-0.79182	Ncoa6
42701	-1.01682	10.04317	-3.37816	0.003749	0.022716	-1.95239	Tmem128
25584	-1.01685	9.228166	-3.51713	0.002789	0.019531	-1.67258	Fam134c
60415	-1.01687	9.216395	-3.47003	0.003083	0.020504	-1.76755	C030046E11Rik
50386	-1.017	11.28592	-3.75466	0.001681	0.015038	-1.19213	Bphl
21680	-1.01711	16.27106	-5.99045	1.74E-05	0.002891	3.166244	Lpl
8523	-1.0172	11.21661	-3.15065	0.006073	0.029782	-2.40681	Prps1
1626	-1.01737	11.94778	-2.89807	0.010329	0.041142	-2.90318	Oxnad1
4582	-1.01745	11.98796	-2.78525	0.013063	0.047832	-3.12112	Arhgef12
30514	-1.01758	10.16666	-3.31307	0.004305	0.024473	-2.08294	Polr1c

34642	-1.01758	12.83739	-3.80896	0.001498	0.014345	-1.08213	Glud1
17596	-1.01761	9.587073	-3.21363	0.005316	0.02756	-2.28159	Kat2b
14329	-1.01772	15.122	-3.99468	0.001009	0.012068	-0.70589	Cox8a
30469	-1.01773	9.278538	-3.30031	0.004423	0.024855	-2.10848	Ddx19b
53125	-1.01778	11.20602	-3.43877	0.003295	0.021171	-1.83051	Rab24
35798	-1.01781	10.77225	-3.34907	0.003988	0.023388	-2.0108	Iars
28842	-1.01783	12.62593	-3.2387	0.005041	0.026724	-2.23162	R3hdm2
51099	-1.01794	11.87768	-3.09002	0.006903	0.032225	-2.52689	Pf4
6456	-1.01802	12.6764	-3.2824	0.004594	0.025403	-2.14432	Rnf6
53472	-1.01821	9.958279	-3.56021	0.002544	0.018513	-1.5856	Rnf38
14303	-1.01847	10.49939	-3.04984	0.007513	0.033886	-2.60615	Tmpo
15614	-1.01853	16.10772	-4.62226	0.00027	0.007254	0.555384	Rplp2
4139	-1.0186	9.464314	-5.30226	6.72E-05	0.004775	1.882989	Med25
30246	-1.01877	8.107277	-4.28446	0.000547	0.009563	-0.12065	Guf1
51042	-1.01877	9.729892	-3.67496	0.001992	0.01631	-1.35353	Btbd9
52519	-1.01878	9.649965	-3.83868	0.001406	0.013923	-1.02191	Repin1
2311	-1.01882	11.40388	-3.27973	0.004621	0.025459	-2.14967	Tmem9b
50822	-1.01903	8.365474	-3.74997	0.001698	0.015061	-1.20164	Vps26b
33921	-1.01909	10.54711	-3.40254	0.003559	0.022041	-1.90341	Actr8
32703	-1.01911	8.23726	-4.0325	0.000931	0.011726	-0.62934	L1td1
320	-1.01915	10.40804	-2.85557	0.011287	0.043702	-2.98559	Cryz
42514	-1.01962	15.38878	-5.17042	8.76E-05	0.005259	1.629775	Atp2a2
41521	-1.01966	9.530788	-3.69479	0.00191	0.015897	-1.31339	Blmh
30936	-1.01969	12.04487	-3.28127	0.004606	0.025433	-2.14659	Eif3m
32654	-1.01983	10.59051	-3.72216	0.001802	0.015482	-1.25797	Gm3258
28557	-1.01984	11.98496	-3.6007	0.002334	0.017655	-1.50377	Eif1b

30638	-1.02	11.35799	-3.40542	0.003538	0.021962	-1.89762	Afg3l2
11209	-1.02001	10.30563	-3.04967	0.007516	0.033886	-2.60649	Snx2
30157	-1.02021	12.30372	-3.5695	0.002495	0.018332	-1.56682	Tmem134
25893	-1.02023	8.945944	-3.27983	0.00462	0.025459	-2.14946	Cog3
5939	-1.02031	8.25222	-4.35761	0.000469	0.009046	0.026433	Dnd1
38200	-1.02048	11.64294	-2.84193	0.011612	0.04443	-3.01196	Nid1
25628	-1.02063	11.4292	-3.73669	0.001747	0.015272	-1.22853	Pin4
5624	-1.02089	9.281586	-3.03587	0.007737	0.034404	-2.63366	Tspan6
31252	-1.02092	9.505699	-3.17294	0.005794	0.029014	-2.36255	Ankrd27
56021	-1.021	11.01535	-3.52071	0.002768	0.019433	-1.66536	Irf1
50659	-1.02117	14.83866	-3.52377	0.00275	0.019343	-1.65919	Oaz2-ps
48724	-1.02125	12.63149	-3.57699	0.002455	0.018192	-1.55169	Rpl36al
48614	-1.02138	8.447975	-4.79569	0.000188	0.006286	0.898759	Siah2
56581	-1.02147	10.28812	-3.81473	0.001479	0.014273	-1.07043	Znrd1as
60119	-1.02155	8.28846	-3.70013	0.001888	0.015774	-1.30259	Purg
19032	-1.02161	14.10215	-4.1165	0.000779	0.010901	-0.45945	Psma2
8118	-1.02166	15.23422	-3.98961	0.00102	0.012136	-0.71615	Hsp90ab1
168	-1.02166	10.4735	-3.2842	0.004577	0.025351	-2.14072	Nck2
23634	-1.02168	9.090318	-4.06916	0.000862	0.011379	-0.55517	Mtcp1
27002	-1.02199	10.02216	-3.62234	0.002229	0.017254	-1.46001	Tm2d2
11243	-1.02202	9.745563	-2.85896	0.011207	0.043503	-2.97903	Acbd3
52420	-1.02224	12.26508	-3.77852	0.001598	0.014708	-1.1438	Tmx2
5651	-1.02228	9.973487	-3.02899	0.00785	0.034628	-2.64719	Exosc6
61887	-1.02245	9.227635	-3.82656	0.001443	0.014117	-1.04645	Tada2a
4115	-1.02268	10.1396	-3.59065	0.002385	0.01787	-1.52409	Gtf3c5
62775	-1.02276	9.256381	-3.56349	0.002527	0.018445	-1.57898	Snx15

20245	-1.02276	9.570017	-3.56115	0.002539	0.018512	-1.58371	Stard13
49572	-1.02281	10.13471	-3.47986	0.003019	0.020329	-1.74775	Epc1
5925	-1.02295	9.745414	-3.9769	0.001048	0.012318	-0.74189	Ap3s2
31293	-1.02308	14.88024	-3.89111	0.001258	0.01323	-0.91567	Rpl27
10825	-1.02308	9.790867	-3.2495	0.004927	0.026341	-2.21006	Pdgfra
60357	-1.02318	12.15064	-3.73521	0.001752	0.015284	-1.23153	Larp4b
7264	-1.0232	9.840879	-4.22977	0.000614	0.010087	-0.23082	Srpr
26144	-1.0233	9.521015	-3.50595	0.002856	0.019739	-1.69513	Ccdc61
32293	-1.02363	8.830601	-3.36334	0.003869	0.02299	-1.98215	Whsc2
61431	-1.02366	10.35879	-3.7816	0.001588	0.014679	-1.13757	Nup133
42268	-1.02371	10.50392	-3.2364	0.005065	0.026796	-2.23619	Cdc5l
19047	-1.02371	11.48338	-3.43182	0.003344	0.021333	-1.8445	Rcsd1
53892	-1.02373	9.034872	-3.55428	0.002577	0.018638	-1.59758	D230037D09Rik
46714	-1.02397	8.114663	-3.54386	0.002635	0.018815	-1.61861	Ccl7
16475	-1.02407	7.848861	-4.08892	0.000826	0.011211	-0.5152	Pgpep1
6804	-1.0242	13.24089	-4.55204	0.000312	0.007607	0.415573	Cd36
36673	-1.02422	9.599503	-3.18247	0.005678	0.028617	-2.34361	Casp6
6672	-1.02423	12.89253	-3.22368	0.005204	0.027282	-2.26156	Nedd4
50744	-1.02424	12.93698	-3.64586	0.00212	0.016831	-1.41243	Qk
15543	-1.02428	14.42485	-3.48579	0.002981	0.020149	-1.73578	Cdc37
6922	-1.02428	13.26982	-3.48381	0.002994	0.020224	-1.73979	Lgmn
50941	-1.02429	13.40058	-2.82926	0.011922	0.045157	-3.03642	Crispld2
21984	-1.02443	13.29699	-3.89385	0.00125	0.013205	-0.91011	Erp29
59210	-1.0245	9.920497	-3.54947	0.002603	0.018753	-1.6073	Tbcel
54397	-1.02452	10.8013	-3.19484	0.005531	0.028177	-2.319	Exoc3
48043	-1.02457	8.603325	-3.46321	0.003128	0.020609	-1.7813	Tada1

19443	-1.02477	8.977156	-4.10325	0.000802	0.011077	-0.48623	Usp38
18012	-1.02482	9.006265	-3.56765	0.002504	0.018361	-1.57056	Isg20l2
46626	-1.02518	10.33835	-3.74335	0.001722	0.015184	-1.21505	Ankrd17
44440	-1.02521	9.143984	-3.72767	0.001781	0.015384	-1.2468	Extl1
50434	-1.02547	9.766476	-3.49207	0.002942	0.019992	-1.72313	Zfp281
45916	-1.02554	14.78743	-3.44983	0.003219	0.020878	-1.80824	Rpl22
15708	-1.02567	8.95932	-4.21315	0.000635	0.0101	-0.26433	Surf2
41677	-1.02568	11.44365	-3.05598	0.007416	0.033562	-2.59405	Impdh2
1580	-1.02573	12.13716	-3.00273	0.008295	0.035868	-2.69877	Ebna1bp2
57918	-1.02577	10.67061	-3.81742	0.001471	0.014231	-1.06499	Ppp3cb
14342	-1.02597	13.54188	-4.00343	0.000991	0.012022	-0.68818	Gm6010
7534	-1.02609	13.61916	-3.88627	0.001271	0.013278	-0.92547	Srl
45281	-1.0261	12.10856	-3.85252	0.001365	0.013751	-0.99385	Robld3
30118	-1.02617	9.33272	-3.00665	0.008227	0.0357	-2.69109	Bcl3
50354	-1.0262	9.91755	-3.60215	0.002327	0.017641	-1.50083	Zfp386
35934	-1.02644	10.32299	-3.83315	0.001423	0.013978	-1.03312	Aamp
9684	-1.02661	12.28925	-3.51365	0.00281	0.019589	-1.67961	Pdcd6
22524	-1.02664	10.52654	-3.42475	0.003395	0.021507	-1.85874	Smarca5
4444	-1.02666	10.03205	-3.25162	0.004905	0.026261	-2.20583	Chfr
54233	-1.02677	9.340678	-3.46747	0.0031	0.020535	-1.77272	Gng12
41640	-1.02689	9.956696	-3.26067	0.004811	0.026008	-2.18775	Rab34
4752	-1.02709	9.662765	-3.28737	0.004546	0.025232	-2.13438	Sirt1
42574	-1.0271	8.774017	-4.08246	0.000838	0.011255	-0.52826	Pm20d1
30113	-1.02713	12.7743	-3.74498	0.001716	0.015149	-1.21174	Eif4h
59846	-1.02716	9.524391	-3.37025	0.003812	0.022879	-1.96828	Gramd4
50666	-1.02727	11.04239	-3.3765	0.003762	0.022767	-1.95573	Pmvk

44701	-1.0273	11.96244	-3.5811	0.002434	0.01812	-1.5434	Sharpin
11898	-1.02734	10.5685	-3.12429	0.006421	0.030812	-2.45908	Cd82
29688	-1.02742	11.27783	-3.59758	0.00235	0.017719	-1.51008	Cep63
31498	-1.02745	14.07969	-4.6071	0.000278	0.007325	0.525238	Prdx5
59034	-1.02745	11.14562	-3.58197	0.002429	0.018098	-1.54163	Oplah
32276	-1.02754	7.516242	-4.99308	0.000126	0.005707	1.285824	Slc9a2
24570	-1.02763	10.09492	-3.14495	0.006147	0.030006	-2.41813	D8Ertd82e
30629	-1.02764	9.522309	-3.53171	0.002704	0.019116	-1.64314	Zfml
9268	-1.02776	9.748956	-3.19546	0.005524	0.028163	-2.31776	Ctcf
22094	-1.02795	9.335519	-3.36901	0.003822	0.022879	-1.97076	Igdcc4
7205	-1.0281	9.61588	-3.2959	0.004465	0.024999	-2.11731	Lrig1
13962	-1.02811	9.152453	-3.64438	0.002127	0.016859	-1.41542	Extl2
27877	-1.02821	10.62792	-3.40258	0.003559	0.022041	-1.90333	Ctnnal1
16267	-1.02834	12.68478	-3.66856	0.00202	0.016408	-1.36648	Znhit1
16368	-1.02881	11.72186	-3.14879	0.006097	0.029849	-2.41051	Msl1
48117	-1.02892	9.04896	-4.05976	0.000879	0.011455	-0.57418	Nme4
54594	-1.02911	8.486901	-3.63552	0.002167	0.017052	-1.43336	Ei24
62770	-1.02918	11.22637	-3.00249	0.0083	0.03587	-2.69925	Rpap3
60121	-1.02922	9.193599	-3.94028	0.001133	0.012673	-0.81606	Vta1
5275	-1.02993	11.35373	-3.45923	0.003155	0.020669	-1.78932	Spin1
21564	-1.02994	9.425205	-3.26789	0.004738	0.025809	-2.17333	Fam13a
21229	-1.03004	9.877701	-3.39253	0.003636	0.022321	-1.92353	Ifrd2
35179	-1.03008	10.94219	-3.23352	0.005096	0.026909	-2.24195	Aagab
10428	-1.03012	13.52249	-3.17526	0.005765	0.028924	-2.35793	Eef1b2
33700	-1.03024	10.25165	-2.75421	0.013931	0.049965	-3.18058	Grasp
18467	-1.03041	9.00983	-3.89436	0.001249	0.013205	-0.90908	Cox18

46518	-1.03042	9.444193	-3.36968	0.003817	0.022879	-1.96943	Fancl
58090	-1.03092	11.31528	-3.75465	0.001681	0.015038	-1.19215	ORF19
38651	-1.03095	10.91071	-4.1047	0.000799	0.01107	-0.48331	Parl
11438	-1.03118	12.14094	-3.50509	0.002861	0.019749	-1.69687	Abcc8
21374	-1.0313	9.444468	-3.44128	0.003278	0.021098	-1.82546	Tmem33
55424	-1.03136	11.91085	-3.59993	0.002338	0.017671	-1.50532	F11r
35893	-1.03138	9.368383	-3.06098	0.007339	0.033348	-2.5842	Psmd14
5109	-1.03142	12.98059	-3.86981	0.001316	0.013489	-0.95882	Higd2a
28478	-1.03161	10.65189	-3.77013	0.001627	0.014847	-1.16079	Arfgap2
26016	-1.03168	10.69444	-3.33702	0.004091	0.023741	-2.03495	Tmem188
31030	-1.03176	11.41626	-3.89199	0.001255	0.013211	-0.91388	Anxa11
28369	-1.03179	13.21508	-2.99742	0.008388	0.036093	-2.70919	Eif3l
50508	-1.03179	11.73784	-2.89034	0.010497	0.041624	-2.9182	Airn
33138	-1.03181	11.95079	-3.5446	0.00263	0.018815	-1.61712	Glo1
26855	-1.0319	9.807032	-3.5602	0.002544	0.018513	-1.58562	Polg
50601	-1.0319	9.641766	-3.0916	0.00688	0.032145	-2.52375	Agps
10104	-1.03195	10.31193	-3.15564	0.00601	0.029609	-2.39691	Srp19
4426	-1.03199	9.728835	-3.0939	0.006847	0.032037	-2.5192	Aldh9a1
49095	-1.032	10.6038	-3.85454	0.001359	0.013742	-0.98976	Larp4
11358	-1.03229	8.422012	-4.23004	0.000613	0.010087	-0.23028	Gm1943
57542	-1.03229	10.146	-3.30898	0.004342	0.024603	-2.09114	Slc37a4
10806	-1.03234	13.8665	-2.98416	0.008625	0.036717	-2.73519	Itga7
13317	-1.03243	11.54527	-3.3679	0.003831	0.022899	-1.97299	Ppp3ca
51615	-1.03259	13.5869	-3.57357	0.002473	0.018272	-1.5586	Mrpl55
53891	-1.03299	10.00263	-3.55318	0.002583	0.018669	-1.5998	BC018507
8948	-1.03302	12.05259	-3.16751	0.005861	0.029223	-2.37335	Surf4

5455	-1.03308	12.51003	-3.69174	0.001923	0.015958	-1.31956	Apoa1bp
56633	-1.03312	9.964234	-3.41272	0.003483	0.02179	-1.88294	Luc7l
61820	-1.03314	14.27178	-3.70805	0.001857	0.01565	-1.28655	Got2
5758	-1.0334	10.61641	-4.12978	0.000758	0.010755	-0.43263	Prpf19
53611	-1.03355	8.526121	-3.67486	0.001993	0.01631	-1.35374	Zfp661
6616	-1.0336	10.02223	-3.5999	0.002338	0.017671	-1.50539	Crls1
2871	-1.03383	10.49078	-3.89559	0.001246	0.013205	-0.9066	Pfdn2
57213	-1.03384	12.03534	-3.42417	0.003399	0.021521	-1.85989	Carkd
40654	-1.03385	9.030945	-3.06002	0.007354	0.033392	-2.58609	Thap1
23500	-1.03387	13.36352	-3.86838	0.00132	0.013502	-0.96173	Dtnbp1
22675	-1.0339	9.823852	-3.36024	0.003894	0.023047	-1.98838	Cnksr3
26103	-1.03398	7.873714	-4.84344	0.000171	0.006133	0.992777	Lrrc39
56709	-1.03402	12.0772	-2.88259	0.010668	0.042106	-2.93323	Txn1
37840	-1.03407	12.23775	-3.11718	0.006518	0.031058	-2.47315	Prrc2b
11015	-1.03408	13.2264	-3.47641	0.003042	0.020394	-1.75471	Sumo1
31563	-1.03408	10.59934	-2.82582	0.012008	0.045351	-3.04305	Hexb
52893	-1.03412	12.79152	-3.31605	0.004278	0.024392	-2.07697	Bod1
61728	-1.03423	9.37916	-3.29903	0.004435	0.024891	-2.11105	Ercc2
22662	-1.03423	10.79185	-4.28886	0.000542	0.009554	-0.1118	Dnttip1
2194	-1.03423	8.47716	-3.79155	0.001554	0.014594	-1.11739	C330019G07Rik
61869	-1.03435	10.25286	-3.49258	0.002939	0.019989	-1.72209	Arhgap23
29550	-1.03436	9.46526	-3.84352	0.001392	0.013865	-1.0121	Ykt6
57629	-1.03446	11.3057	-3.25541	0.004865	0.026153	-2.19826	Kbtbd2
20594	-1.03447	11.85493	-3.36501	0.003855	0.022938	-1.97879	Copb2
43574	-1.03451	14.08467	-3.90186	0.001229	0.013182	-0.89388	Rpl23a-ps1
36519	-1.03472	8.906772	-3.09173	0.006878	0.032145	-2.5235	Ntsr2

60777	-1.03486	11.96292	-4.06143	0.000876	0.011455	-0.57081	Narfl
17347	-1.03494	9.567251	-3.36319	0.00387	0.02299	-1.98246	Fbxl17
22253	-1.03501	8.877124	-3.76553	0.001643	0.014895	-1.17013	Daam1
42090	-1.03513	11.60348	-3.15602	0.006005	0.0296	-2.39616	Ube2r2
10132	-1.03515	12.48777	-3.73102	0.001768	0.015357	-1.24002	Neurl2
54419	-1.03516	10.30326	-3.21847	0.005261	0.027407	-2.27194	Fkbp7
22079	-1.03522	8.003749	-3.86709	0.001324	0.01351	-0.96434	Tec
27055	-1.03527	10.00682	-3.19713	0.005505	0.028109	-2.31446	Pramef8
15441	-1.03547	7.763215	-4.42039	0.000411	0.008563	0.152393	Abcb7
21738	-1.03549	10.02066	-3.41477	0.003468	0.021743	-1.87882	Fam193b
35208	-1.03567	11.12294	-3.75838	0.001668	0.01499	-1.18461	Slc41a3
16291	-1.03572	12.4406	-4.6879	0.000235	0.006899	0.685676	Atp5c1
13537	-1.03574	10.93285	-3.53769	0.002669	0.018977	-1.63109	Exosc5
29854	-1.03576	9.433988	-3.55405	0.002578	0.01864	-1.59803	Igsf8
14167	-1.03577	13.82302	-3.1501	0.00608	0.029806	-2.4079	Sepp1
33026	-1.03586	9.3803	-3.70054	0.001887	0.015769	-1.30176	Prkaa1
18334	-1.03588	11.20779	-3.6995	0.001891	0.015786	-1.30385	Fam98c
1313	-1.03592	9.811206	-3.36196	0.00388	0.023027	-1.98492	Nup37
39522	-1.03598	9.680368	-3.32748	0.004175	0.02405	-2.05407	Morc2a
58203	-1.03599	11.54291	-3.36327	0.003869	0.02299	-1.9823	BC024659
55449	-1.03619	10.84099	-4.25337	0.000584	0.009828	-0.18327	Mrps35
28990	-1.03624	11.86736	-3.71767	0.001819	0.015552	-1.26706	Prkaa2
4959	-1.03626	11.6458	-3.3564	0.003926	0.023168	-1.99607	Nsmce4a
46053	-1.03626	9.818849	-3.56575	0.002515	0.0184	-1.57441	Samd4b
54311	-1.0364	11.44622	-3.54985	0.002601	0.018753	-1.60652	Tomm20
6020	-1.03645	13.95503	-3.69535	0.001908	0.01589	-1.31225	Mrps23

58656	-1.03656	9.204856	-3.90405	0.001223	0.01316	-0.88945	Gpatch1
52314	-1.03668	10.8397	-3.75278	0.001688	0.015038	-1.19594	B3gnt1
19498	-1.03673	8.493772	-3.95126	0.001107	0.012525	-0.79381	Pex10
7497	-1.03675	10.8533	-3.04205	0.007637	0.034182	-2.6215	Nr1h3
7547	-1.03679	9.748268	-3.71635	0.001824	0.015558	-1.26974	Crot
47224	-1.03679	13.36354	-3.43342	0.003333	0.02129	-1.84128	Ctsl
36040	-1.03682	11.35178	-4.01752	0.000961	0.011911	-0.65966	Ccdc12
62611	-1.03687	8.777385	-4.181	0.00068	0.010327	-0.3292	Zfp866
20922	-1.03691	11.9906	-3.75164	0.001692	0.015038	-1.19825	Usp28
4728	-1.03693	14.21225	-3.6599	0.002057	0.016573	-1.38402	Ecsit
3037	-1.03701	9.305733	-3.27	0.004717	0.025783	-2.16912	Trim55
8562	-1.03702	10.5033	-4.09123	0.000822	0.011187	-0.51054	Msh2
4432	-1.03712	9.805668	-3.31037	0.00433	0.024568	-2.08835	Bcdin3d
15466	-1.03726	9.529198	-3.36121	0.003886	0.02303	-1.98643	AI597468
33625	-1.03743	12.52254	-3.76379	0.001649	0.014901	-1.17365	Erh
7337	-1.03744	11.24658	-2.89684	0.010356	0.041204	-2.90557	Uba2
8213	-1.03749	11.61214	-3.7269	0.001784	0.015393	-1.24837	Trappc1
46403	-1.03768	11.00611	-3.25502	0.004869	0.026163	-2.19903	Fam98a
45095	-1.03775	11.27404	-3.45098	0.003211	0.020878	-1.80593	Mcoln1
6920	-1.0378	8.354348	-4.00252	0.000993	0.012032	-0.69002	Gadd45a
59280	-1.03785	11.20617	-3.24711	0.004952	0.026398	-2.21483	Cdadc1
21683	-1.03818	14.07548	-4.99292	0.000126	0.005707	1.28551	Slc25a3
17416	-1.0382	8.372456	-4.15571	0.000717	0.010555	-0.38025	Usp45
43171	-1.03843	10.07429	-3.89281	0.001253	0.013211	-0.91222	Atpaf2
32407	-1.0385	10.23207	-3.5728	0.002477	0.018289	-1.56017	Prps1l3
35105	-1.03852	8.258071	-3.83519	0.001416	0.013953	-1.02897	Cpne3

43286	-1.03856	9.930514	-3.6341	0.002174	0.017075	-1.43622	Gm3646
22857	-1.03857	9.038266	-3.64146	0.00214	0.01691	-1.42134	Hltf
13071	-1.03863	9.485809	-3.07202	0.00717	0.032919	-2.56243	Kat2a
39134	-1.03871	10.97624	-3.65679	0.002071	0.016636	-1.39032	Stk39
15279	-1.03877	9.169005	-3.57082	0.002487	0.018311	-1.56416	Ddx47
39618	-1.03898	8.900545	-3.38146	0.003723	0.022643	-1.94576	Klhl25
1817	-1.0392	10.58265	-3.46393	0.003123	0.020606	-1.77984	Tmem131
23421	-1.03932	8.660375	-3.95389	0.0011	0.01252	-0.78848	N6amt1
16674	-1.03943	10.13985	-3.35903	0.003904	0.023094	-1.99081	Rnf103
5549	-1.03944	11.68374	-2.81208	0.012356	0.046138	-3.06954	Samd1
33563	-1.03944	8.076753	-4.7458	0.000209	0.006636	0.800288	Bik
51222	-1.03979	9.723636	-3.49489	0.002924	0.019934	-1.71744	Kpna4
46156	-1.03999	8.819581	-4.00451	0.000988	0.01201	-0.68598	Upf2
37720	-1.04011	12.18355	-3.36471	0.003858	0.022944	-1.97941	Ezh1
58201	-1.04038	7.838694	-4.20646	0.000644	0.010117	-0.27782	Cdk7
9648	-1.04071	9.412634	-3.2742	0.004675	0.025631	-2.16071	Csnk2a2
16502	-1.04072	8.701075	-4.42177	0.00041	0.008558	0.155155	Cdk16
7564	-1.04073	11.8609	-3.18622	0.005633	0.028488	-2.33616	Rftn1
24104	-1.04091	10.16923	-3.61238	0.002277	0.017453	-1.48016	Ttc33
49949	-1.04106	10.77886	-3.43271	0.003338	0.021299	-1.84271	Sarnp
11943	-1.04112	8.082738	-4.56046	0.000307	0.007568	0.432372	Plxnb3
20974	-1.04132	13.50614	-3.68516	0.00195	0.016103	-1.33288	Snrpd2
40446	-1.04141	10.4947	-3.1913	0.005573	0.028314	-2.32604	Hdac7
57760	-1.04144	10.09358	-4.48273	0.000361	0.008021	0.277178	Neo1
43921	-1.04144	9.048326	-3.87951	0.001289	0.013363	-0.93916	Polr2h
37325	-1.04159	14.83585	-5.3131	6.57E-05	0.004733	1.903712	Atp5j

41396	-1.04163	10.87839	-3.03017	0.00783	0.034566	-2.64487	Gar1
43885	-1.04171	10.42421	-3.26382	0.004779	0.025911	-2.18145	Hsd17b11
61114	-1.04171	8.683839	-3.56438	0.002522	0.018434	-1.57717	Spice1
60131	-1.04181	9.230827	-3.88473	0.001275	0.013309	-0.92859	Gnb1l
43920	-1.04193	13.05048	-3.73893	0.001739	0.015236	-1.22399	Mrpl52
16236	-1.04195	12.0289	-3.34995	0.00398	0.023375	-2.00902	Tmem93
46157	-1.04197	10.17852	-3.75947	0.001664	0.014971	-1.1824	Frs2
33824	-1.04202	9.599309	-3.98776	0.001024	0.012161	-0.7199	Kti12
53535	-1.04205	11.17574	-3.55711	0.002561	0.018592	-1.59187	Timm8a1
10953	-1.04212	12.17036	-4.12529	0.000765	0.010796	-0.44169	Dohh
44195	-1.04225	13.05733	-3.29065	0.004515	0.025162	-2.12783	Tmed2
55447	-1.04252	10.87967	-3.15766	0.005984	0.029531	-2.3929	Zfp710
26173	-1.04265	7.80964	-3.60794	0.002298	0.017522	-1.48913	Lypd2
8907	-1.04316	8.660197	-4.57568	0.000297	0.007471	0.462687	Blcap
59067	-1.04341	10.71821	-3.49497	0.002924	0.019934	-1.71727	Cmtm6
34804	-1.0439	12.98127	-3.81156	0.00149	0.01431	-1.07686	Gm7555
23705	-1.04404	10.09026	-3.46048	0.003147	0.020649	-1.7868	Atf2
26062	-1.04405	9.706945	-3.5075	0.002847	0.019705	-1.69201	Mecp2
1530	-1.0441	11.04278	-3.51396	0.002808	0.019589	-1.67898	Arcn1
4192	-1.04419	11.14256	-3.18693	0.005625	0.028479	-2.33474	Ube2a
1266	-1.04437	10.69631	-3.07457	0.007132	0.032799	-2.5574	Nudt16l1
37800	-1.04439	10.92789	-3.80187	0.001521	0.014445	-1.0965	Gtf2a2
19688	-1.04454	10.48426	-3.39618	0.003608	0.022229	-1.91619	Srsf1
7018	-1.04464	9.938868	-3.87314	0.001307	0.013449	-0.95207	Add3
39721	-1.04471	9.601188	-3.78965	0.001561	0.014599	-1.12125	Ccdc28b
34332	-1.04478	11.81604	-2.7815	0.013165	0.04807	-3.12832	Ets2

60286	-1.0448	12.06741	-3.70877	0.001854	0.01565	-1.28509	Sf3b5
11590	-1.04483	9.509256	-3.51781	0.002785	0.019516	-1.67121	Kbtbd12
6170	-1.04513	9.97069	-3.04369	0.007611	0.034071	-2.61826	Fem1b
36726	-1.04519	10.31042	-3.07349	0.007148	0.032846	-2.55952	Chchd8
2149	-1.04524	11.57292	-3.20796	0.00538	0.027742	-2.29288	Fam40a
60052	-1.04535	11.94238	-3.66095	0.002053	0.016561	-1.3819	Slc35b1
23895	-1.04539	9.117554	-4.36652	0.00046	0.008954	0.044317	Rnmtl1
5352	-1.04559	10.45893	-3.27752	0.004642	0.025534	-2.15409	Ubn2
34958	-1.04563	11.44215	-3.58414	0.002418	0.018058	-1.53725	Rbfox2
46917	-1.04587	13.51283	-3.8823	0.001281	0.013352	-0.93351	Csda
38032	-1.04594	12.63701	-3.15551	0.006011	0.029609	-2.39716	Rbbp7
6555	-1.04606	7.836881	-4.4899	0.000355	0.007997	0.291519	G630090E17Rik
33394	-1.04624	9.193373	-3.28214	0.004597	0.025411	-2.14484	Gm3613
15274	-1.04627	9.763394	-3.44596	0.003245	0.021001	-1.81603	Ltbp1
18587	-1.04632	9.543046	-3.28934	0.004527	0.025178	-2.13044	Rpf1
41347	-1.04643	13.17946	-3.53212	0.002701	0.019106	-1.64233	Dnpep
43404	-1.04644	9.153437	-3.38812	0.00367	0.022463	-1.93239	Zfand6
24305	-1.04652	10.44218	-3.6542	0.002083	0.016684	-1.39555	Ankrd46
2184	-1.04655	9.856503	-4.09183	0.000821	0.01118	-0.50932	Btbd2
38578	-1.04671	9.843937	-3.44591	0.003246	0.021001	-1.81614	Setd6
55649	-1.04671	8.145666	-4.02263	0.000951	0.011806	-0.64932	Krt10
3817	-1.04693	11.92722	-3.29441	0.004479	0.025054	-2.1203	Son
25243	-1.04693	8.385345	-3.97134	0.00106	0.012341	-0.75315	Zfp341
28158	-1.04695	9.268738	-3.78615	0.001572	0.014645	-1.12834	Rcbtb2
59112	-1.047	9.629943	-3.57229	0.00248	0.018302	-1.5612	Gosr1
11814	-1.04708	9.345098	-4.73515	0.000213	0.006665	0.77922	Fam193a

7988	-1.04735	10.81851	-3.47803	0.003031	0.020348	-1.75144	Gm15431
16882	-1.04745	11.10777	-3.23902	0.005037	0.026723	-2.23098	Psmc6
46124	-1.04749	8.788125	-4.24808	0.00059	0.009909	-0.19392	Zfp213
59300	-1.0475	9.813294	-3.28513	0.004568	0.025314	-2.13886	Ncapd2
3737	-1.04756	9.598641	-4.0045	0.000988	0.01201	-0.68601	Snrpn
51266	-1.04794	11.12048	-3.43737	0.003305	0.02121	-1.83335	Araf
20282	-1.04799	10.11804	-3.89998	0.001234	0.013201	-0.8977	BC024814
54765	-1.04807	9.522157	-3.674	0.001997	0.016322	-1.35548	Bcor
45919	-1.0481	12.07402	-3.12217	0.00645	0.030891	-2.46328	Csrp2
49085	-1.04812	8.160141	-4.50036	0.000348	0.007904	0.312423	Gstm7
56493	-1.04815	11.53754	-3.44753	0.003234	0.020954	-1.81289	Cwc15
33474	-1.04834	8.442822	-4.18126	0.00068	0.010327	-0.32867	Abhd10
18716	-1.04836	11.76932	-4.59185	0.000287	0.007378	0.494905	Pnkd
60129	-1.04845	10.13449	-4.55759	0.000308	0.007581	0.426648	Pim3
32948	-1.04857	9.246777	-3.8359	0.001414	0.013953	-1.02754	Wdr83
16814	-1.04858	8.210207	-4.0294	0.000938	0.011752	-0.63561	Gmps
20838	-1.04881	9.709411	-3.7921	0.001553	0.01459	-1.11629	Exoc8
1305	-1.04886	10.27634	-3.095	0.006831	0.031983	-2.51704	Tmem129
12165	-1.04897	11.64611	-3.12004	0.006479	0.030969	-2.4675	Celf2
19245	-1.04902	11.81035	-3.14886	0.006097	0.029849	-2.41038	Fam136a
47478	-1.04921	15.82088	-5.49181	4.60E-05	0.004091	2.243052	Ndufv1
22984	-1.04923	11.90432	-3.65046	0.002099	0.016751	-1.40311	Rpa1
38468	-1.04929	13.83646	-4.03466	0.000927	0.011716	-0.62496	Fastk
53260	-1.04948	8.709536	-4.19358	0.000662	0.010247	-0.30381	Mrps5
49365	-1.0495	9.507781	-3.79033	0.001558	0.014596	-1.11987	Nup54
34888	-1.04967	9.586305	-3.0524	0.007472	0.033761	-2.60111	Ebpl

19442	-1.04968	9.598872	-4.89495	0.000153	0.005923	1.093927	Hemk1
49050	-1.0497	11.04939	-3.53096	0.002708	0.019129	-1.64467	Cept1
50558	-1.04986	10.56656	-3.23703	0.005059	0.026787	-2.23495	Ankra2
51353	-1.04986	11.81673	-3.64401	0.002128	0.016861	-1.41618	Armc1
17175	-1.04989	10.63354	-3.07828	0.007076	0.032645	-2.55006	Wtip
53830	-1.04995	8.877609	-4.06875	0.000862	0.011382	-0.556	Pnpt1
11066	-1.05006	12.35757	-3.65136	0.002095	0.016742	-1.4013	Asnsd1
59497	-1.05014	10.48515	-2.87223	0.010901	0.042788	-2.95333	Stag2
44719	-1.05014	9.286725	-3.70745	0.001859	0.01565	-1.28776	Sirt4
26368	-1.05017	13.86051	-3.74059	0.001733	0.015225	-1.22065	Nenf
61457	-1.05035	10.22034	-3.37312	0.003789	0.022849	-1.96252	Fkbp2
26222	-1.05056	13.77663	-3.26312	0.004786	0.025937	-2.18285	Psma3
18911	-1.05059	8.879345	-3.98133	0.001038	0.012262	-0.73292	Eif2b3
23683	-1.05062	12.4385	-3.56067	0.002542	0.018513	-1.58466	Hmg20b
15696	-1.05082	14.47531	-3.80127	0.001523	0.014454	-1.0977	Pgm2
18909	-1.0509	14.05216	-3.59399	0.002368	0.017794	-1.51734	Prdx6
44316	-1.05103	12.24148	-3.27852	0.004632	0.0255	-2.15208	Fam96a
4594	-1.05106	15.15141	-4.93294	0.000142	0.005859	1.16834	Ndufv2
10345	-1.05108	8.701855	-4.43455	0.000399	0.008456	0.180764	Nlrx1
35738	-1.05113	8.997851	-3.50831	0.002842	0.019701	-1.69037	Caprin2
39059	-1.05142	8.965595	-2.8517	0.011378	0.043924	-2.99307	Kcnip2
20304	-1.05151	11.7455	-4.00813	0.000981	0.011995	-0.67865	Mtx1
36235	-1.05151	9.063983	-3.53141	0.002705	0.019122	-1.64375	Dhx15
20936	-1.05162	11.54361	-3.12193	0.006453	0.030893	-2.46375	Ctnnd1
18885	-1.05164	12.51555	-3.76171	0.001656	0.014915	-1.17785	Eif2b4
1151	-1.05166	14.71004	-3.97475	0.001053	0.012334	-0.74623	Pkig

48879	-1.05167	13.06773	-3.6818	0.001964	0.016154	-1.3397	Arhgef7
23563	-1.05171	8.543863	-4.25671	0.00058	0.009789	-0.17654	Tmem82
4877	-1.05188	9.065845	-3.69354	0.001915	0.015915	-1.31593	Taf8
15075	-1.05217	10.26994	-3.42687	0.00338	0.021485	-1.85447	Ssna1
27290	-1.05242	12.63725	-3.2663	0.004754	0.025849	-2.17651	Murc
52793	-1.05245	9.796563	-4.05931	0.00088	0.011455	-0.57509	Nipa2
27458	-1.05249	9.638252	-3.63021	0.002192	0.017112	-1.4441	Dpcd
31265	-1.05253	10.96203	-3.70812	0.001857	0.01565	-1.2864	Apba3
42684	-1.05288	9.160943	-4.3565	0.00047	0.009047	0.024189	Cno
30945	-1.05312	14.08286	-3.98447	0.001031	0.012226	-0.72656	Pptc7
38440	-1.05332	8.407847	-4.22001	0.000626	0.0101	-0.2505	Cd1d1
13419	-1.05342	13.45091	-3.55608	0.002567	0.018615	-1.59395	Hspa9
26427	-1.05347	12.88821	-3.86233	0.001337	0.013616	-0.97397	Egln2
52881	-1.05348	14.55477	-3.99147	0.001016	0.012109	-0.71238	Mtap7d1
14214	-1.05363	8.458524	-3.89419	0.001249	0.013205	-0.90943	Ppp6r3
9686	-1.05382	12.251	-3.74803	0.001705	0.015099	-1.20557	Dhx30
417	-1.05382	12.26101	-3.1283	0.006367	0.030632	-2.45114	Arhgap21
54143	-1.05394	8.322028	-4.69511	0.000232	0.006885	0.699972	Dpyd
51299	-1.05396	9.982636	-3.2125	0.005328	0.027606	-2.28384	Fam63b
50603	-1.05418	11.11522	-3.43637	0.003312	0.021215	-1.83536	Osbpl2
7310	-1.05427	10.18931	-3.08724	0.006944	0.032298	-2.53238	Usp15
39068	-1.05445	13.83714	-4.7175	0.000221	0.006791	0.744319	Sgca
53262	-1.05491	10.4592	-3.39169	0.003643	0.022349	-1.92522	Nxt1
44188	-1.05499	10.09941	-3.80385	0.001514	0.014415	-1.09248	Kdm2b
17699	-1.05525	10.00212	-3.38423	0.003701	0.022568	-1.9402	Pdpr
3348	-1.0553	11.65727	-3.43634	0.003312	0.021215	-1.83541	Arl6ip5

27152	-1.05571	13.30389	-3.36982	0.003816	0.022879	-1.96914	Tmem63b
42910	-1.05578	9.35842	-3.54591	0.002623	0.018788	-1.61449	Ino80d
4257	-1.05582	11.44844	-3.38351	0.003706	0.022585	-1.94164	Ttc19
55331	-1.05582	10.57082	-3.87927	0.00129	0.013363	-0.93966	Tbc1d16
10610	-1.056	8.809012	-4.46253	0.000376	0.008192	0.236785	Vps33a
9040	-1.05612	13.48063	-3.87569	0.0013	0.013415	-0.9469	Bloc1s1
33806	-1.05614	11.23401	-3.35079	0.003973	0.023352	-2.00734	Tgfbr2
27843	-1.05632	9.857747	-3.64968	0.002103	0.016767	-1.40471	Gtf3a
34471	-1.0564	8.398396	-4.08232	0.000838	0.011255	-0.52855	Tcfcp2
40859	-1.05653	11.00116	-3.14733	0.006116	0.029914	-2.4134	C2cd2l
53921	-1.05657	10.72748	-3.15961	0.005959	0.029462	-2.38903	Ankrd16
18757	-1.05665	9.162853	-3.03947	0.007679	0.034276	-2.62658	E2f3
62630	-1.05689	14.79807	-3.40089	0.003572	0.022085	-1.90672	Ctnna1
12586	-1.05708	11.50214	-3.03074	0.007821	0.034553	-2.64376	Top2b
45232	-1.05718	8.919555	-4.42562	0.000406	0.008514	0.162867	Zfp868
8912	-1.05731	9.437485	-3.7561	0.001676	0.015024	-1.18921	Mzt1
31042	-1.05763	12.80959	-4.47949	0.000363	0.00805	0.2707	Map1lc3a
19852	-1.05802	12.85124	-3.84351	0.001392	0.013865	-1.01212	Ppdpf
2102	-1.05804	10.58567	-3.47902	0.003025	0.020334	-1.74944	Rnf111
29754	-1.05815	11.43167	-4.21447	0.000634	0.0101	-0.26167	Srp54c
23297	-1.05827	10.67187	-3.65164	0.002094	0.016739	-1.40074	Ola1
60353	-1.05831	8.969678	-4.08281	0.000837	0.011255	-0.52755	Clns1a
17312	-1.05856	9.837652	-3.2898	0.004523	0.025171	-2.12952	Haus4
57450	-1.05863	11.50871	-3.75297	0.001687	0.015038	-1.19557	LOC100046282
52859	-1.05867	10.64832	-3.42467	0.003396	0.021507	-1.8589	Tctex1d2
27465	-1.05872	10.44297	-3.06436	0.007287	0.033262	-2.57753	Kdm5b

38815	-1.05878	11.88729	-3.19057	0.005582	0.02835	-2.32749	U2af1
41939	-1.05908	8.865576	-3.99131	0.001016	0.012109	-0.71271	Helb
3151	-1.05913	13.43634	-3.40817	0.003517	0.021892	-1.89209	Creg1
22271	-1.05921	8.998454	-3.34728	0.004003	0.023433	-2.01438	Tgs1
4969	-1.0595	13.32032	-3.88717	0.001268	0.013271	-0.92365	Ndufa8
21713	-1.0596	9.46312	-4.60774	0.000278	0.007325	0.526507	Tm9sf1
60858	-1.05986	9.371274	-3.6477	0.002112	0.016819	-1.40871	Nxt2
32381	-1.05991	14.29667	-3.76226	0.001654	0.014915	-1.17674	Gm9457
26446	-1.06003	12.35765	-3.3132	0.004304	0.024473	-2.08269	Nxf1
47851	-1.06003	10.30607	-3.2293	0.005142	0.027066	-2.25035	Exoc2
31589	-1.06007	9.807557	-4.54619	0.000316	0.007649	0.403922	Rbfa
8814	-1.06017	14.52528	-4.08235	0.000838	0.011255	-0.52849	Txn2
39039	-1.06018	12.56537	-3.47947	0.003022	0.020329	-1.74853	Ccdc72
54303	-1.06021	9.538292	-4.30553	0.000523	0.009425	-0.07826	Zfp770
46902	-1.06021	8.331384	-3.50345	0.002871	0.01976	-1.70017	Mtap
16144	-1.06029	12.39161	-3.09554	0.006823	0.03196	-2.51596	Rbm39
39742	-1.06033	10.28516	-3.63485	0.00217	0.017058	-1.43472	Camsap1
26102	-1.06053	8.935403	-3.78725	0.001569	0.014645	-1.12611	Agk
44430	-1.06059	11.28682	-3.25754	0.004843	0.026079	-2.194	Nfe2l2
48095	-1.0606	10.39115	-4.11928	0.000775	0.010871	-0.45383	Denr
36212	-1.06061	12.21533	-4.26824	0.000566	0.009682	-0.1533	Ap1b1
18884	-1.06072	13.62242	-3.57694	0.002455	0.018192	-1.55179	Gm2099
27407	-1.06094	13.25202	-3.17003	0.00583	0.02914	-2.36834	Dnaja4
35280	-1.06132	15.94917	-3.59887	0.002343	0.017698	-1.50746	Hba-a1
36538	-1.06144	8.409051	-4.3666	0.00046	0.008954	0.044482	Map2k6
34962	-1.06148	11.57685	-4.05246	0.000893	0.01149	-0.58894	Ythdf1

4197	-1.06161	9.418505	-3.18989	0.00559	0.028359	-2.32885	Zfp238
33141	-1.06168	11.34006	-3.90808	0.001213	0.013108	-0.88129	Sms
60423	-1.06178	11.15228	-3.40063	0.003574	0.022088	-1.90725	Ociad1
17134	-1.06206	10.11702	-3.04507	0.007589	0.034024	-2.61554	Pigs
24254	-1.06209	8.625007	-3.43271	0.003338	0.021299	-1.84272	Tfdp1
24400	-1.06217	11.73444	-3.7798	0.001594	0.014702	-1.14121	Rab3a
41501	-1.06232	10.36881	-3.57113	0.002486	0.018311	-1.56355	Rnf181
12499	-1.06236	11.23911	-3.58283	0.002425	0.018084	-1.53989	Fam168b
24551	-1.0627	10.16605	-2.81897	0.01218	0.045755	-3.05626	Matn2
60830	-1.06272	10.51107	-3.91465	0.001196	0.013013	-0.86798	Ttc7
9931	-1.06275	11.87763	-3.62259	0.002228	0.017254	-1.45951	Gstp1
16221	-1.06293	11.13153	-3.6646	0.002037	0.016487	-1.37451	Taz
45532	-1.06314	10.68809	-4.12993	0.000758	0.010755	-0.43233	Rnf11
1249	-1.06321	10.92355	-3.94278	0.001127	0.012635	-0.81099	Gfm2
26481	-1.06345	12.50929	-2.83516	0.011777	0.044807	-3.02503	Jun
27881	-1.06352	9.054252	-3.44142	0.003277	0.021098	-1.82519	Pde1c
21583	-1.06352	10.74261	-3.33635	0.004097	0.023749	-2.0363	Osbpl5
28654	-1.06368	8.329126	-4.08136	0.00084	0.011264	-0.5305	Nop16
29350	-1.06379	14.02822	-4.22697	0.000617	0.010087	-0.23646	Rpl7a-ps5
14139	-1.06387	10.11287	-3.42262	0.003411	0.02158	-1.86302	AW549877
685	-1.06405	10.72353	-3.72823	0.001779	0.015379	-1.24566	Suv420h2
23992	-1.06416	12.67978	-3.49434	0.002928	0.019951	-1.71856	Ank1
38821	-1.06423	9.029213	-3.59226	0.002376	0.017839	-1.52083	Olfml2b
31606	-1.06444	11.97483	-3.09831	0.006783	0.031869	-2.51049	Hnrnpc
6293	-1.06454	10.78772	-3.44069	0.003282	0.021115	-1.82665	Sertad2
34912	-1.0648	9.056948	-3.48737	0.002971	0.0201	-1.73261	Fbxo11

517	-1.06492	10.37583	-3.63932	0.00215	0.016969	-1.42567	Tpp2
40623	-1.06505	8.923286	-4.20481	0.000647	0.010126	-0.28116	Nrip1
14699	-1.06519	8.195281	-4.65357	0.000253	0.007106	0.61758	Prkab1
9601	-1.06563	9.82775	-3.42497	0.003394	0.021507	-1.85829	Oxr1
34084	-1.06566	10.5178	-3.32381	0.004208	0.024181	-2.06142	Tgfbr3
49676	-1.06567	11.56169	-3.05601	0.007416	0.033562	-2.59399	Csnk1a1
38574	-1.06571	8.21672	-4.07936	0.000843	0.011277	-0.53454	C1qtnf4
38216	-1.06572	9.700291	-4.82573	0.000177	0.006166	0.957932	Gtf3c1
5153	-1.06626	11.9769	-3.26241	0.004794	0.025958	-2.18427	Klc2
45271	-1.06635	9.725705	-3.8264	0.001443	0.014117	-1.04678	Nipa1
42918	-1.06651	12.14831	-3.89556	0.001246	0.013205	-0.90665	Obsl1
10297	-1.06661	8.996438	-3.66817	0.002022	0.016409	-1.36729	Ccdc91
44866	-1.06674	12.57455	-3.67405	0.001996	0.016322	-1.35537	Fnta
58854	-1.06723	11.68556	-4.00151	0.000995	0.012039	-0.69206	Agpat6
34409	-1.06727	11.82739	-2.94469	0.009369	0.038726	-2.81237	Arpc5l
19262	-1.06743	10.36097	-3.12304	0.006438	0.030866	-2.46155	Jmjd1c
4704	-1.06752	11.04824	-2.79201	0.012881	0.047441	-3.10812	Thy1
58889	-1.06756	10.62165	-3.72863	0.001777	0.015378	-1.24486	Tsen15
31730	-1.06767	11.24743	-4.06008	0.000878	0.011455	-0.57353	Sh2b1
8530	-1.06774	13.67244	-4.21797	0.000629	0.0101	-0.25462	Cpt1b
14724	-1.06786	9.908949	-3.71626	0.001825	0.015558	-1.26992	Rasgrp2
59961	-1.06791	8.032194	-4.22019	0.000626	0.0101	-0.25014	Car2
6548	-1.06798	9.741455	-3.06141	0.007332	0.033344	-2.58336	Bdp1
21441	-1.06803	13.11982	-3.31496	0.004288	0.024443	-2.07916	Cdk2ap1
21992	-1.06806	9.932238	-4.14636	0.000732	0.010607	-0.39912	Rbm6
13629	-1.06807	9.892224	-3.73733	0.001745	0.015258	-1.22724	Commd8

52100	-1.06817	11.76819	-3.31137	0.00432	0.024535	-2.08636	Tsnax
51433	-1.06821	14.26295	-4.06747	0.000865	0.011399	-0.55858	Acox1
46419	-1.06873	11.63748	-4.34251	0.000484	0.00913	-0.00391	Gm6133
10602	-1.0688	9.12321	-3.90152	0.00123	0.013186	-0.89458	Hexim1
28166	-1.06883	9.074437	-4.18155	0.000679	0.010327	-0.32809	Med22
53563	-1.06892	13.5323	-4.10606	0.000797	0.011056	-0.48055	Gm4825
10386	-1.06931	10.11265	-3.74593	0.001713	0.015125	-1.20983	Fip1l1
10459	-1.06935	13.09754	-3.47423	0.003056	0.020437	-1.7591	Mrpl48
28075	-1.06993	10.33432	-4.30725	0.000521	0.009398	-0.07479	Tnfaip2
19941	-1.07015	10.85433	-2.9771	0.008754	0.037072	-2.749	Glt8d1
59289	-1.07023	9.851038	-3.14702	0.00612	0.029914	-2.41402	Rabgap1l
61999	-1.07039	14.48385	-4.7722	0.000198	0.006509	0.852431	Brp44
51201	-1.07044	9.457676	-3.78708	0.001569	0.014645	-1.12645	Gm8801
30479	-1.07069	9.059117	-3.85964	0.001345	0.013644	-0.97943	Zfp277
8618	-1.07074	8.624661	-3.30363	0.004392	0.024763	-2.10185	Stard3nl
34755	-1.07088	8.689563	-3.2654	0.004763	0.025854	-2.1783	Zmat1
15457	-1.07101	13.5676	-2.98829	0.008551	0.036518	-2.72709	Fabp5l2
4955	-1.07107	14.23205	-3.74054	0.001733	0.015225	-1.22075	Nisch
58036	-1.07125	11.63546	-3.35814	0.003912	0.023119	-1.99259	Guk1
41140	-1.07125	9.516794	-2.94834	0.009298	0.038591	-2.80525	Lrrk2
42113	-1.07146	10.73333	-3.51039	0.002829	0.019655	-1.68617	Cdkn2d
395	-1.07152	10.5036	-3.61016	0.002288	0.017493	-1.48464	Zscan21
40437	-1.07184	9.261153	-3.18578	0.005639	0.028495	-2.33702	Rassf3
48562	-1.07199	11.52226	-4.12975	0.000758	0.010755	-0.43268	Snrpd3
12363	-1.072	10.75708	-4.19397	0.000662	0.010247	-0.30302	Naa10
28832	-1.072	8.641635	-2.96022	0.009069	0.037987	-2.78204	Ccl24

47996	-1.07205	11.43674	-3.6245	0.002219	0.017241	-1.45564	Asb10
19324	-1.07209	11.92841	-3.33883	0.004076	0.023681	-2.03132	Zcchc24
20607	-1.07209	14.42306	-4.02646	0.000943	0.011762	-0.64156	LOC100504872
32830	-1.07213	9.545755	-3.72252	0.001801	0.015476	-1.25724	Snx14
44744	-1.07213	11.81308	-3.56086	0.002541	0.018512	-1.58428	Ccnl2
58665	-1.07226	10.86883	-4.00604	0.000985	0.01201	-0.68289	Unc45a
8	-1.07234	11.31514	-3.50772	0.002845	0.019705	-1.69157	C85492
4859	-1.07238	8.297334	-3.80118	0.001523	0.014454	-1.09789	Sec23a
44700	-1.07241	8.447666	-5.02857	0.000117	0.005645	1.35495	Mto1
16837	-1.07261	8.239242	-3.9328	0.001151	0.012818	-0.83121	Sema5a
51825	-1.07268	10.65121	-4.63219	0.000264	0.007219	0.575128	Mrpl38
42143	-1.07273	9.607839	-4.07336	0.000854	0.011339	-0.54667	Psmg3
12691	-1.07281	11.36395	-3.69588	0.001906	0.015885	-1.31119	Foxj3
12683	-1.07292	12.5152	-3.07743	0.007089	0.032663	-2.55174	Sipa1l2
25747	-1.07338	8.252068	-4.07268	0.000855	0.011339	-0.54805	Prepl
24270	-1.07341	8.799282	-3.79424	0.001545	0.014574	-1.11194	Zfp644
763	-1.07349	12.83134	-4.54262	0.000318	0.007663	0.396784	Asb11
20069	-1.07354	8.01477	-4.78989	0.000191	0.006332	0.88732	Zfp128
48705	-1.07376	9.577798	-3.41702	0.003451	0.021703	-1.87429	Hmg20a
32167	-1.07386	11.16474	-3.49346	0.002933	0.019964	-1.72033	Lmo4
42987	-1.07397	9.156413	-4.11454	0.000783	0.010932	-0.46343	Rhobtb3
14572	-1.07405	15.85145	-4.93921	0.00014	0.005859	1.180618	Hrc
17002	-1.0741	9.976017	-3.29935	0.004432	0.02488	-2.11042	Cdk2ap2
13635	-1.07417	11.9645	-3.10717	0.006658	0.0315	-2.49297	Bicc1
16088	-1.0742	10.42189	-3.63028	0.002192	0.017112	-1.44395	Aftph
43066	-1.07437	15.32129	-4.91852	0.000146	0.005905	1.140124	Got1

18818	-1.07446	12.42398	-3.25395	0.00488	0.026189	-2.20118	Snrpg
23439	-1.07449	10.0366	-3.13711	0.00625	0.030342	-2.43368	Casp9
18003	-1.07451	8.045783	-4.16281	0.000707	0.010472	-0.36591	Zfp11
40827	-1.07452	9.143818	-2.99569	0.008419	0.036197	-2.71259	Slc22a17
14301	-1.07458	10.26011	-3.53905	0.002662	0.018958	-1.62832	Nup107
9090	-1.07462	9.894332	-3.38777	0.003673	0.022467	-1.93308	Gfpt1
784	-1.07467	13.37918	-3.8998	0.001235	0.013201	-0.89806	Psmd12
36123	-1.07512	10.7214	-3.7095	0.001851	0.01565	-1.28361	P2ry2
19314	-1.07522	7.190695	-2.7863	0.013035	0.04776	-3.11909	Prss12
36413	-1.07562	9.782181	-3.61131	0.002282	0.017474	-1.48231	Colec11
42893	-1.07575	10.7735	-3.47679	0.003039	0.020389	-1.75393	Pcmt1
41627	-1.07582	11.65945	-3.03842	0.007696	0.034319	-2.62864	Pop7
14858	-1.07603	11.4425	-3.0229	0.007951	0.034927	-2.65915	Thrsp
37197	-1.07604	12.54342	-3.62434	0.002219	0.017241	-1.45596	Fech
14061	-1.07629	11.66931	-3.72987	0.001773	0.01537	-1.24234	Hyal2
53148	-1.07644	10.98586	-3.71849	0.001816	0.015541	-1.26541	Aven
16294	-1.07645	9.688393	-4.61068	0.000276	0.007325	0.532364	Gde1
4035	-1.07646	11.49323	-2.92979	0.009666	0.03948	-2.84144	Rad21
53820	-1.07647	10.22489	-4.28958	0.000541	0.009554	-0.11035	Mrpl9
32219	-1.07658	12.61651	-3.70646	0.001863	0.015673	-1.28976	Gps1
16451	-1.07665	13.60194	-3.4704	0.003081	0.020494	-1.76681	Calm2
33970	-1.07713	9.353899	-4.22509	0.00062	0.010087	-0.24026	Parp8
13096	-1.07717	13.35684	-2.90276	0.010228	0.040896	-2.89405	Lmod3
34125	-1.07738	13.81197	-4.17972	0.000682	0.010327	-0.33178	Mrpl12
51581	-1.07751	11.58463	-3.57179	0.002482	0.018311	-1.5622	Cd320
41434	-1.07754	9.792976	-3.6629	0.002044	0.016522	-1.37795	Txnrd3

6021	-1.07767	9.273609	-2.94124	0.009437	0.038872	-2.81911	Hlf
42173	-1.07771	11.51365	-4.18676	0.000672	0.01029	-0.31756	Mcat
44940	-1.07773	8.073975	-3.81877	0.001467	0.014216	-1.06225	Fam117a
38782	-1.07776	11.64815	-3.49842	0.002902	0.019868	-1.71033	Zwint
27455	-1.0778	8.531593	-4.1321	0.000754	0.010744	-0.42794	Rasd2
48399	-1.07782	12.30349	-3.6214	0.002233	0.017264	-1.4619	Ube2d3
4719	-1.07787	11.01713	-3.37208	0.003798	0.022874	-1.96461	Ube2e1
55328	-1.07792	9.408295	-4.45205	0.000385	0.008288	0.2158	Slc44a1
22490	-1.07793	10.56924	-3.29429	0.00448	0.025054	-2.12055	Dusp12
48734	-1.07798	11.86611	-4.07735	0.000847	0.011298	-0.53861	Lmf1
53320	-1.07804	12.44006	-4.75695	0.000204	0.006618	0.822306	Pink1
20567	-1.07814	8.169213	-3.78555	0.001574	0.014645	-1.12955	Ankrd34a
53474	-1.07818	14.85129	-5.0886	0.000103	0.0055	1.471556	Myoz2
26711	-1.07824	9.298011	-3.97485	0.001053	0.012334	-0.74605	Smcr8
18424	-1.07829	8.648314	-4.08488	0.000833	0.011248	-0.52337	Zfp961
50448	-1.07836	12.08639	-4.74041	0.000211	0.006665	0.789622	Dnajc4
36554	-1.07844	8.420488	-2.75603	0.013878	0.049847	-3.17709	Prr15
10508	-1.07845	8.093811	-3.2849	0.00457	0.02532	-2.13933	Col4a6
39785	-1.07861	9.878542	-3.93108	0.001155	0.012818	-0.83468	Synj2
41725	-1.07862	10.96742	-3.71532	0.001828	0.01557	-1.27181	Ndufaf1
31677	-1.07868	10.18796	-4.15011	0.000726	0.010601	-0.39155	Nme6
22130	-1.07878	15.83926	-3.99953	0.000999	0.012047	-0.69607	Rpl9
34232	-1.0788	15.98881	-5.5607	4.02E-05	0.003964	2.372697	Ndufb11
21620	-1.07889	11.89293	-3.672	0.002005	0.016353	-1.35953	Pcnt
58701	-1.07905	10.38373	-3.8118	0.001489	0.014309	-1.07637	Dnajc8
52439	-1.07963	11.83978	-2.85647	0.011266	0.04365	-2.98385	Zfp36l2

2521	-1.07964	12.43077	-3.32313	0.004214	0.02421	-2.0628	Atp6v0e
15645	-1.07966	10.19822	-4.14569	0.000733	0.010611	-0.40048	Tmem205
7949	-1.0797	11.26338	-3.08778	0.006936	0.032282	-2.5313	Parp6
37497	-1.0797	11.46215	-4.05268	0.000892	0.01149	-0.5885	Slc2a8
3526	-1.07975	13.56703	-3.40925	0.003509	0.021889	-1.88991	Spg7
60402	-1.07979	9.936489	-3.47101	0.003077	0.020486	-1.76559	Atf1
62918	-1.08012	9.814118	-3.48258	0.003002	0.020256	-1.74225	Srp72
48358	-1.0803	9.838467	-3.44878	0.003226	0.02091	-1.81036	Hrasls
9944	-1.08046	9.641715	-4.00746	0.000982	0.011995	-0.68001	Sphk2
16248	-1.08048	9.343608	-4.63539	0.000262	0.007219	0.581479	Gdpd5
6936	-1.08069	13.18294	-3.75636	0.001675	0.015024	-1.1887	Ppm1b
33063	-1.08088	11.2796	-3.50331	0.002872	0.01976	-1.70045	Sf3b3
10965	-1.08092	12.02458	-3.5863	0.002407	0.017981	-1.53289	Abhd11
32747	-1.08099	12.81246	-3.61069	0.002285	0.017485	-1.48358	Polr2i
10348	-1.081	9.645985	-4.23656	0.000605	0.010014	-0.21713	Gtpbp2
13938	-1.08103	15.44619	-4.53501	0.000323	0.007678	0.381611	Etfa
36393	-1.08124	12.95862	-3.77641	0.001605	0.014738	-1.14808	Selk
42420	-1.08133	9.368864	-3.5919	0.002378	0.01784	-1.52157	Rev3l
49574	-1.0815	11.51049	-3.327	0.004179	0.024066	-2.05505	Sec61a1
40342	-1.08151	8.661759	-3.94611	0.001119	0.012593	-0.80424	Kin
30528	-1.08152	13.4739	-3.85199	0.001367	0.013754	-0.99494	Rnf128
18622	-1.08152	11.4114	-3.07748	0.007088	0.032663	-2.55164	Rasa3
62171	-1.08171	11.22806	-3.38974	0.003658	0.022423	-1.92913	Cetn2
37517	-1.08176	9.984817	-3.62517	0.002216	0.017235	-1.45429	Mthfd1
38669	-1.08183	9.744654	-4.40603	0.000423	0.008651	0.123604	Dhx33
29951	-1.08189	10.723	-3.27562	0.004661	0.02559	-2.15789	Sbds

14442	-1.08194	13.77559	-4.94939	0.000137	0.005859	1.200512	Fam195a
11223	-1.08222	12.6548	-3.71336	0.001836	0.015584	-1.2758	Timm44
56059	-1.08223	10.72836	-3.52358	0.002751	0.019345	-1.65957	Rbm10
52532	-1.08223	8.216886	-3.97384	0.001055	0.012341	-0.74808	Adamts14
55871	-1.08254	12.85245	-3.37499	0.003774	0.022806	-1.95876	Hp1bp3
42669	-1.08267	10.48757	-4.20277	0.00065	0.010138	-0.28527	Glrx2
51637	-1.08305	11.44371	-4.14725	0.00073	0.010607	-0.39733	Opa3
31415	-1.08312	12.12018	-3.5932	0.002372	0.017815	-1.51894	Atp11a
19744	-1.08316	9.279676	-4.02248	0.000951	0.011806	-0.64962	Cyb5rl
44680	-1.08324	12.47778	-3.64676	0.002116	0.016831	-1.41062	Bri3bp
4379	-1.08331	9.907208	-3.34945	0.003985	0.023383	-2.01002	Usp3
60943	-1.08331	14.75989	-5.21382	8.02E-05	0.004967	1.71337	Uqcrfs1
10003	-1.08347	12.35651	-3.16247	0.005924	0.029382	-2.38335	Sirt3
18557	-1.08391	9.168037	-3.85544	0.001357	0.013729	-0.98793	Nrbf2
59881	-1.08391	10.73782	-4.38122	0.000446	0.008891	0.073837	Abcb10
56585	-1.08404	12.32622	-3.96387	0.001077	0.012408	-0.76827	Zfand5
3420	-1.08414	11.59486	-2.86209	0.011134	0.043339	-2.97296	Cxcr7
57825	-1.08439	8.5514	-3.55622	0.002566	0.018615	-1.59366	Nipal3
4887	-1.08441	13.23893	-3.85074	0.00137	0.013778	-0.99746	Casq2
12729	-1.08451	15.01243	-4.21105	0.000638	0.010115	-0.26858	Gm11810
1094	-1.08461	10.95065	-2.98889	0.00854	0.036489	-2.72591	Smarce1
61115	-1.08462	9.954361	-3.36742	0.003835	0.022914	-1.97396	Fbxo25
53905	-1.08493	12.06379	-3.49099	0.002949	0.020009	-1.72532	Tarbp2
61786	-1.08493	11.47759	-3.44127	0.003278	0.021098	-1.82549	Creld2
23227	-1.08496	13.01396	-3.53695	0.002674	0.018977	-1.63257	Ube2l3
47153	-1.08499	10.45018	-3.96459	0.001076	0.012402	-0.76682	Btf3l4

9737	-1.08499	10.78696	-4.50299	0.000346	0.007904	0.317671	Rfk
39193	-1.085	12.75192	-4.68445	0.000237	0.006925	0.678847	Mapkapk5
26996	-1.08504	8.4424	-4.20684	0.000644	0.010117	-0.27707	Lrrc57
12885	-1.08532	14.30697	-4.1481	0.000729	0.010606	-0.39562	Slc4a3
34673	-1.08541	8.575334	-3.82131	0.001459	0.014191	-1.05709	Tcta
28562	-1.0857	11.73122	-3.85974	0.001344	0.013644	-0.97922	Man2c1
696	-1.08579	11.20867	-3.81976	0.001464	0.014211	-1.06023	Grb2
53250	-1.0858	12.42615	-4.0081	0.000981	0.011995	-0.67872	Coq10a
38252	-1.08585	13.13739	-3.77098	0.001624	0.014835	-1.15907	Sub1
57768	-1.08607	14.93942	-4.27288	0.00056	0.009646	-0.14396	Cox7a2
14853	-1.08625	9.996734	-3.4372	0.003306	0.02121	-1.83369	Cbfa2t2
15150	-1.08638	10.67165	-3.72512	0.001791	0.015421	-1.25196	Ccdc75
4670	-1.08639	10.76149	-3.1921	0.005564	0.028305	-2.32445	Zfp322a
39443	-1.08653	11.35473	-3.88955	0.001262	0.013239	-0.91883	Mrps15
21385	-1.0866	11.69496	-3.38095	0.003727	0.022644	-1.94679	Fcf1
60343	-1.08664	13.7523	-3.02599	0.007899	0.034757	-2.65308	Vti1b
370	-1.08677	9.954997	-3.90337	0.001225	0.013165	-0.89082	Spen
22906	-1.08697	10.93195	-3.26506	0.004767	0.025862	-2.17898	Ost4
22468	-1.08701	9.433444	-3.00259	0.008298	0.03587	-2.69905	Cd200
60598	-1.08705	11.18615	-3.37023	0.003813	0.022879	-1.96831	Pdpk1
37746	-1.0871	10.80352	-3.55971	0.002547	0.018514	-1.5866	Chuk
62294	-1.08724	9.233032	-3.36786	0.003832	0.022899	-1.97308	Atg3
45160	-1.08727	10.17307	-3.25603	0.004859	0.02615	-2.19701	Dhx40
2390	-1.08731	9.845899	-3.18618	0.005634	0.028488	-2.33624	Bach1
26145	-1.08732	10.66783	-3.96208	0.001082	0.012434	-0.77189	Fry
37507	-1.0875	11.35546	-4.22096	0.000625	0.0101	-0.24859	Msl3

15399	-1.08763	8.781168	-3.63862	0.002153	0.016977	-1.42707	Dock5
46112	-1.08769	9.396086	-3.6948	0.00191	0.015897	-1.31338	Ctsc
50037	-1.08823	10.32248	-4.40609	0.000423	0.008651	0.123723	Rpap1
6289	-1.08824	11.95201	-3.30099	0.004417	0.024838	-2.10713	Bptf
43819	-1.08829	9.842462	-4.49985	0.000348	0.007904	0.311408	Lyrm4
60890	-1.08836	7.128596	-3.42645	0.003383	0.021485	-1.85531	Gm4544
19256	-1.08869	10.3255	-3.5144	0.002805	0.019583	-1.67808	Gm10767
44838	-1.08884	10.60798	-3.63534	0.002168	0.017052	-1.43371	Zc3h14
46176	-1.08887	12.61972	-3.62798	0.002202	0.017163	-1.44861	Nutf2
40811	-1.08902	10.21362	-3.80029	0.001526	0.014459	-1.09969	Mterfd2
4990	-1.08921	8.221118	-4.50056	0.000347	0.007904	0.312829	Slc19a1
33279	-1.0897	9.67047	-3.81894	0.001466	0.014216	-1.06191	Fam73a
62555	-1.08993	15.52932	-3.70311	0.001877	0.015731	-1.29654	Oxct1
58113	-1.09013	9.100058	-3.86143	0.00134	0.013623	-0.9758	Ube3c
2945	-1.09014	9.864141	-3.64599	0.002119	0.016831	-1.41217	Eif2c4
60642	-1.09015	13.65899	-3.20604	0.005402	0.027789	-2.2967	Taok1
56420	-1.09071	10.44711	-3.51358	0.00281	0.019589	-1.67974	Atp6ap1
35874	-1.09071	8.396786	-4.64954	0.000255	0.007124	0.609587	Klhl7
11094	-1.09071	11.25214	-3.60872	0.002295	0.017516	-1.48756	Cry2
8978	-1.09092	12.41561	-4.54233	0.000318	0.007663	0.396211	Rbm24
18384	-1.09096	11.00393	-4.05469	0.000889	0.01149	-0.58445	Plk1s1
51680	-1.09112	12.09545	-3.86201	0.001338	0.013616	-0.97462	Hnrnpm
21340	-1.09137	8.898192	-3.71419	0.001833	0.015584	-1.2741	Tmem185b
33444	-1.09144	9.674547	-3.77402	0.001613	0.014782	-1.15291	Pias2
21123	-1.09153	8.587118	-4.2455	0.000594	0.00994	-0.19913	Ercc8
42686	-1.09154	11.28931	-3.62314	0.002225	0.017254	-1.4584	G3bp1

40659	-1.09157	13.80164	-4.01422	0.000968	0.011934	-0.66633	Perp
42167	-1.09165	10.68351	-3.18369	0.005664	0.028582	-2.34118	Ggh
30300	-1.09198	7.845006	-5.57854	3.88E-05	0.003938	2.406167	Galm
3505	-1.09211	9.84467	-3.26698	0.004747	0.025827	-2.17515	Caml
28461	-1.09224	10.37154	-3.60617	0.002307	0.017546	-1.49272	Efr3a
27759	-1.09227	14.4164	-4.3184	0.000509	0.009336	-0.05237	Epas1
36410	-1.09243	7.577445	-3.54593	0.002623	0.018788	-1.61445	Dll1
24635	-1.09246	9.573962	-2.76679	0.013573	0.049086	-3.15651	P4ha1
44985	-1.09257	9.621454	-3.87161	0.001311	0.013481	-0.95517	Ptpn2
52918	-1.09259	8.520603	-4.06045	0.000878	0.011455	-0.57278	Prdm16
60321	-1.09276	9.496838	-3.96453	0.001076	0.012402	-0.76694	Nbeal1
27917	-1.09296	10.12793	-4.08315	0.000837	0.011255	-0.52688	Phf14
62714	-1.09299	9.255477	-3.86898	0.001318	0.0135	-0.96051	Ap3m1
39237	-1.09321	10.54924	-7.62893	9.05E-07	0.001159	5.933003	Ppp1r3b
19566	-1.09323	11.11516	-3.76573	0.001642	0.014895	-1.16972	Bcs1l
28687	-1.09334	9.853085	-3.6337	0.002176	0.017075	-1.43702	Pdcd2
25166	-1.09354	9.163644	-3.89597	0.001245	0.013205	-0.90581	Naaa
41898	-1.09409	8.622392	-4.08463	0.000834	0.011248	-0.52387	Nicn1
55965	-1.09418	9.754647	-3.40773	0.00352	0.021892	-1.89297	Fah
33447	-1.09422	9.118516	-3.68912	0.001933	0.016018	-1.32488	Nudt21
37199	-1.09427	9.855604	-3.87567	0.0013	0.013415	-0.94696	Plcl2
8714	-1.09433	9.931524	-3.89368	0.001251	0.013205	-0.91046	Rad17
57206	-1.09446	9.072019	-3.70849	0.001855	0.01565	-1.28566	Vps37a
58107	-1.09458	14.50421	-4.16667	0.000701	0.010424	-0.35811	Psmb6
49936	-1.09461	12.73114	-3.25873	0.004831	0.026053	-2.19163	Polr2g
32650	-1.09464	10.94194	-3.81954	0.001464	0.014211	-1.06068	Fgf1

28568	-1.09483	12.32647	-4.2079	0.000643	0.010117	-0.27492	Fbxw5
14120	-1.09516	12.50344	-3.91623	0.001192	0.013001	-0.86477	Arl6ip4
24583	-1.09524	9.899674	-3.59792	0.002348	0.017719	-1.5094	Plcb3
62855	-1.09524	12.91037	-4.57829	0.000295	0.007466	0.467888	Psap
35921	-1.09536	10.30666	-3.90903	0.001211	0.013091	-0.87935	Mvk
17423	-1.09582	10.95621	-4.06083	0.000877	0.011455	-0.57202	Wdr45
12544	-1.09591	9.938805	-3.42523	0.003392	0.021507	-1.85777	Fam110a
53740	-1.09602	11.28432	-3.89774	0.00124	0.013202	-0.90224	Bicd2
39897	-1.09608	10.30317	-3.85776	0.00135	0.013686	-0.98323	Dcun1d5
19732	-1.09609	8.984931	-3.34122	0.004055	0.023611	-2.02654	Acpl2
46142	-1.0961	9.47496	-3.73951	0.001737	0.015235	-1.22283	Dgka
32370	-1.09632	11.24501	-3.71884	0.001815	0.015538	-1.26469	Tbkbp1
49531	-1.09638	8.731441	-3.50682	0.002851	0.019722	-1.69337	Spon1
29492	-1.09674	11.28689	-3.21918	0.005254	0.027398	-2.27053	Arhgef25
24094	-1.09682	9.30205	-3.46931	0.003088	0.020516	-1.76901	Polb
45642	-1.09686	11.88389	-4.41052	0.000419	0.008624	0.132593	Polr2j
11799	-1.09698	8.283904	-4.60235	0.000281	0.007328	0.515793	Pts
7294	-1.09705	13.02084	-3.47946	0.003022	0.020329	-1.74855	Ppp1cb
33322	-1.09722	11.28691	-3.3659	0.003848	0.022932	-1.97702	Slc15a4
46816	-1.09733	8.848368	-3.9142	0.001197	0.013013	-0.86888	Clec2d
12399	-1.09742	10.53675	-3.59968	0.002339	0.017673	-1.50584	Appbp2
44295	-1.09757	13.88264	-4.50179	0.000347	0.007904	0.31528	Ube2b
6584	-1.09767	9.279088	-3.40862	0.003514	0.021892	-1.89117	Dlg3
47951	-1.09775	8.520205	-4.43018	0.000403	0.00847	0.172011	Pds5b
59393	-1.09804	13.16224	-3.96248	0.001081	0.01243	-0.7711	Ppp1cc
49003	-1.09814	8.829236	-3.07026	0.007197	0.032962	-2.5659	F830016B08Rik

25848	-1.09836	10.38437	-3.71586	0.001826	0.015558	-1.27073	Dnajc9
57626	-1.09849	14.86305	-4.96264	0.000134	0.005828	1.226414	Gm5786
49712	-1.09869	12.43485	-4.05373	0.00089	0.01149	-0.58639	Rbpms2
27974	-1.09908	8.580912	-3.9159	0.001193	0.013003	-0.86544	Rwdd4a
60332	-1.09913	8.353552	-4.59756	0.000284	0.007333	0.506261	Ube2w
34563	-1.09917	13.47451	-3.12318	0.006436	0.030866	-2.46128	Hspb8
49552	-1.09934	13.47509	-5.05764	0.00011	0.005572	1.411468	Ckm
8884	-1.09936	10.99117	-4.41106	0.000419	0.008622	0.133692	Mtr
19327	-1.09977	8.50599	-4.41793	0.000413	0.008563	0.147451	Aldh1l1
15467	-1.09978	11.90573	-3.36168	0.003882	0.023027	-1.98548	Mesdc2
44020	-1.1	13.78997	-5.23246	7.73E-05	0.004951	1.749204	Macrod1
51920	-1.10006	10.3978	-3.63221	0.002183	0.017094	-1.44004	Fam173b
51492	-1.10024	10.23146	-3.16871	0.005846	0.029198	-2.37095	Nr1d2
31947	-1.10031	14.70976	-3.02076	0.007987	0.035026	-2.66337	Sh3bgr
42662	-1.10038	10.87674	-4.05256	0.000893	0.01149	-0.58874	Krba1
44725	-1.1005	12.19916	-3.56408	0.002524	0.018434	-1.57779	Rom1
24664	-1.10052	10.17478	-3.83157	0.001427	0.014006	-1.0363	Golph3
17288	-1.10059	9.384957	-3.75619	0.001676	0.015024	-1.18904	Sox7
61735	-1.1006	11.89724	-3.42619	0.003385	0.021485	-1.85583	Arhgef6
26077	-1.10061	7.986596	-4.45367	0.000383	0.008277	0.219036	Rg9mtd1
39846	-1.10062	11.02789	-3.96379	0.001078	0.012408	-0.76844	Ccdc90a
57559	-1.1007	10.52345	-3.60071	0.002334	0.017655	-1.50375	Snrpf
17964	-1.10087	8.696505	-6.6209	5.35E-06	0.002346	4.280202	Gabrr2
42387	-1.10109	10.56524	-3.74007	0.001734	0.015225	-1.22169	Lrba
28758	-1.10122	9.923812	-3.921	0.00118	0.012908	-0.8551	Cars2
54650	-1.10151	10.06792	-3.5821	0.002428	0.018098	-1.54137	Abhd14a

13078	-1.10155	10.24121	-3.37564	0.003769	0.022795	-1.95746	Rai1
59504	-1.10182	10.88506	-3.62774	0.002203	0.017165	-1.44909	Diablo
61416	-1.10184	10.54479	-3.93082	0.001156	0.012818	-0.83522	Mrrf
25175	-1.10193	10.77923	-3.48982	0.002956	0.020021	-1.72767	Zc3h18
52496	-1.10203	10.73101	-2.90843	0.010108	0.040629	-2.88303	Zfp579
61292	-1.10203	10.76607	-3.32871	0.004164	0.024006	-2.05162	Clk4
4345	-1.10209	12.04162	-4.1387	0.000744	0.010683	-0.4146	AV026068
2686	-1.1021	10.80689	-4.03361	0.000929	0.011722	-0.62709	Srpk3
32365	-1.10225	10.73224	-4.11842	0.000776	0.010884	-0.45558	Med1
52203	-1.10312	14.64257	-4.94125	0.00014	0.005859	1.184603	Mrpl14
14331	-1.10318	13.02757	-3.73225	0.001764	0.015332	-1.23753	Ube2j2
16259	-1.10327	9.587164	-4.0518	0.000894	0.011495	-0.59028	Usp21
14980	-1.1034	10.20752	-3.82467	0.001449	0.014138	-1.0503	Cmc1
17483	-1.10369	13.19879	-3.66255	0.002046	0.016528	-1.37865	Mrp63
32389	-1.10374	12.03953	-3.88897	0.001263	0.013239	-0.92001	Nhp2l1
18203	-1.10403	7.698765	-3.59701	0.002353	0.017724	-1.51124	Klra8
17329	-1.10404	11.45232	-3.52615	0.002736	0.01928	-1.65437	Poldip2
38470	-1.10405	9.039551	-4.1167	0.000779	0.010901	-0.45906	Wnt5a
44885	-1.10419	11.03032	-3.35608	0.003929	0.023171	-1.99672	Psmd8
51324	-1.10423	10.38096	-3.44309	0.003265	0.021078	-1.82182	Tmem206
13900	-1.10426	11.85657	-4.09943	0.000808	0.011101	-0.49397	St6galnac6
8023	-1.10439	13.13861	-3.95942	0.001088	0.012478	-0.77729	Mrps25
37261	-1.10443	9.098415	-4.17392	0.00069	0.010371	-0.34349	Med26
5299	-1.10446	14.55385	-4.43164	0.000401	0.008463	0.174935	Mrpl51
3904	-1.10459	11.66566	-3.38113	0.003725	0.022643	-1.94643	Cd47
15597	-1.10491	12.71613	-3.50323	0.002873	0.01976	-1.70062	Fam96b

5735	-1.10494	10.92821	-3.61407	0.002269	0.017417	-1.47674	Pdcd2l
33655	-1.10507	11.90827	-3.70061	0.001887	0.015769	-1.30161	Smc1a
25964	-1.10532	9.777168	-3.61216	0.002278	0.017455	-1.4806	Tomm70a
48878	-1.10537	11.15703	-4.48654	0.000358	0.008021	0.284791	Brd4
41542	-1.10542	13.04401	-3.8866	0.00127	0.013275	-0.9248	Sec24c
46235	-1.10557	10.51161	-3.2911	0.00451	0.025161	-2.12692	Unc13b
48940	-1.10573	10.88517	-4.11955	0.000774	0.010871	-0.4533	Aph1a
29824	-1.10585	9.346306	-3.67188	0.002006	0.016353	-1.35977	Coq10b
7835	-1.10621	12.90861	-4.56834	0.000302	0.007502	0.448077	Ldb3
36722	-1.10622	9.98242	-3.41374	0.003476	0.021767	-1.88089	Klhl17
43069	-1.1063	10.17817	-4.1407	0.000741	0.010661	-0.41055	Snrpc
9750	-1.10631	12.57579	-3.39976	0.00358	0.02212	-1.90899	Tmed10
52084	-1.10638	12.10294	-3.51522	0.0028	0.019574	-1.67643	Yap1
28840	-1.10663	12.67083	-3.44238	0.00327	0.021098	-1.82326	H2afz
44581	-1.10675	10.5481	-3.8497	0.001373	0.013796	-0.99957	Psmg2
28210	-1.10687	11.73049	-3.72175	0.001803	0.015483	-1.25881	Acbd4
40345	-1.10725	10.21123	-3.6327	0.00218	0.017088	-1.43906	Cnot2
11629	-1.10728	8.740244	-3.41974	0.003431	0.021638	-1.86881	Wdr41
32175	-1.10731	10.5099	-4.29255	0.000537	0.00953	-0.10438	Rai12
1357	-1.10738	11.46478	-3.82808	0.001438	0.01408	-1.04339	Dpf2
36547	-1.10746	8.972382	-3.47141	0.003074	0.020486	-1.76478	Bivm
37195	-1.10748	9.726483	-3.47117	0.003076	0.020486	-1.76525	Sgce
35470	-1.10775	11.42115	-3.78404	0.001579	0.014646	-1.13261	Klhl22
30989	-1.10789	11.56444	-3.94321	0.001126	0.012635	-0.81013	Dnajc30
28079	-1.10792	11.59613	-3.43941	0.003291	0.021151	-1.82924	Ilkap
36193	-1.10817	7.76484	-4.09015	0.000824	0.011193	-0.51273	Ccbe1

53812	-1.10818	10.57964	-4.02607	0.000944	0.011763	-0.64234	Thap11
21946	-1.10823	10.79977	-3.65087	0.002097	0.016742	-1.4023	Tmem42
58082	-1.10826	15.51874	-5.72282	2.92E-05	0.003693	2.675191	Ndufb4
58126	-1.10839	9.605551	-3.66654	0.002029	0.016446	-1.37059	Man2a2
19724	-1.1086	10.07601	-3.84649	0.001383	0.013852	-1.00608	Pex6
40747	-1.10864	9.235203	-3.90317	0.001226	0.013165	-0.89123	Homer2
4491	-1.10869	12.26017	-3.67343	0.001999	0.016329	-1.35663	Pde4a
28893	-1.10895	11.63655	-3.60528	0.002311	0.017572	-1.49452	Clasrp
4041	-1.10927	13.9101	-4.20775	0.000643	0.010117	-0.27523	Rps4x
33041	-1.10932	10.52837	-3.87078	0.001313	0.013488	-0.95686	Gkap1
60259	-1.1094	10.91459	-3.23876	0.00504	0.026724	-2.23149	Cnot8
4455	-1.10959	10.75448	-4.85974	0.000165	0.00607	1.024806	Adcy6
23537	-1.10973	10.70629	-3.74952	0.0017	0.01507	-1.20256	Cul4a
41742	-1.10984	8.975744	-4.21385	0.000635	0.0101	-0.26291	Slc2a12
17267	-1.11002	9.150104	-4.18029	0.000681	0.010327	-0.33062	Fbxo34
52672	-1.11013	12.86361	-4.04455	0.000908	0.011594	-0.60496	Mrpl43
13299	-1.11021	12.02151	-4.32001	0.000507	0.009336	-0.04913	Spsb3
41638	-1.11028	8.573468	-3.77824	0.001599	0.014708	-1.14437	Fam185a
21423	-1.11056	13.41174	-3.45314	0.003196	0.020827	-1.80158	Tsen34
22350	-1.11065	8.731474	-4.16834	0.000699	0.010416	-0.35474	Polr3d
11897	-1.1108	9.152842	-3.46577	0.003111	0.020589	-1.77614	Klhl8
43473	-1.11095	10.10198	-4.34108	0.000485	0.009134	-0.00679	Setd2
40833	-1.11096	11.13121	-3.40785	0.003519	0.021892	-1.89273	Lsm2
56186	-1.11105	12.10511	-2.87702	0.010793	0.042492	-2.94405	Btg1
58985	-1.1112	10.93946	-3.68246	0.001961	0.01615	-1.33835	Slc25a46
58445	-1.11121	10.75765	-3.61991	0.00224	0.017286	-1.46492	Klhl12

21275	-1.11131	8.394283	-3.82084	0.00146	0.014191	-1.05806	Ggcx
3664	-1.1114	10.11753	-3.54846	0.002609	0.018768	-1.60934	Chchd4
18770	-1.11151	10.58134	-4.66785	0.000245	0.007001	0.645919	Tmem164
45184	-1.11168	13.43939	-3.97084	0.001062	0.012344	-0.75415	Lsmd1
11574	-1.11245	8.905893	-4.63878	0.00026	0.007218	0.588213	Chchd5
23774	-1.11253	11.19561	-3.60676	0.002304	0.017541	-1.49153	Txnl4a
18179	-1.11275	10.4485	-3.64423	0.002127	0.016859	-1.41572	Fcgrt
33793	-1.11276	10.74478	-4.16916	0.000697	0.010411	-0.3531	Cpsf3
26015	-1.11282	12.2502	-3.90503	0.001221	0.013158	-0.88746	Bud31
28538	-1.11293	11.57908	-3.32019	0.00424	0.024286	-2.06868	Traf2
58144	-1.11295	9.630192	-3.78976	0.00156	0.014599	-1.12104	Aig1
28851	-1.11303	8.092597	-3.72659	0.001785	0.015397	-1.249	Rgs6
56343	-1.11305	10.58179	-3.94216	0.001128	0.012641	-0.81224	Ttc17
45578	-1.11312	8.637041	-4.35953	0.000467	0.009033	0.030289	Sh3kbp1
33296	-1.11349	14.2429	-6.19126	1.19E-05	0.002786	3.527661	Ndufa9
2219	-1.11378	8.374326	-3.84013	0.001402	0.01392	-1.01897	Fam82a1
45612	-1.11379	15.24986	-5.66216	3.29E-05	0.003769	2.562453	Slc25a11
34681	-1.1138	9.223411	-3.88044	0.001286	0.013363	-0.93729	Ppp1r9a
9489	-1.11392	12.0562	-3.70107	0.001885	0.015769	-1.30067	Mrpl34
38472	-1.114	8.361239	-4.29967	0.000529	0.009465	-0.09005	Papolg
61173	-1.11408	10.22041	-4.12949	0.000758	0.010755	-0.4332	Tomm40l
39460	-1.11411	9.373351	-3.82212	0.001456	0.014177	-1.05546	Rcc1
45148	-1.11429	11.5461	-3.6939	0.001914	0.015915	-1.31519	Znfx1
14855	-1.11433	11.63781	-3.67188	0.002006	0.016353	-1.35978	Gstz1
34996	-1.11455	10.82163	-3.61952	0.002242	0.017286	-1.46571	Setx
5422	-1.11475	10.32668	-3.60805	0.002298	0.017522	-1.48892	Lmcd1

19687	-1.11477	10.16469	-4.41618	0.000415	0.008576	0.143948	Cmtm8
43355	-1.11497	13.07079	-4.34624	0.00048	0.009113	0.003585	Ict1
17665	-1.11506	9.980084	-3.72141	0.001805	0.015483	-1.25949	Lifr
25694	-1.11509	11.09706	-3.53722	0.002672	0.018977	-1.63203	Gatad1
60240	-1.11529	12.09215	-2.94176	0.009427	0.038863	-2.8181	Tspan13
12517	-1.1153	11.87329	-3.92542	0.001169	0.012871	-0.84615	Yif1a
11548	-1.11534	12.02294	-3.87443	0.001303	0.013438	-0.94947	Asb8
17766	-1.11538	8.907147	-4.47456	0.000367	0.008089	0.260837	Slco2b1
5639	-1.11561	13.27376	-3.99908	0.001	0.012047	-0.69697	Mrpl11
6155	-1.11562	10.91102	-3.65559	0.002076	0.016665	-1.39273	Scfd2
8605	-1.11564	9.575998	-3.49003	0.002955	0.020021	-1.72725	Ddr2
26027	-1.1159	10.98532	-3.92428	0.001172	0.012876	-0.84846	Isyna1
35999	-1.11696	8.787867	-3.87062	0.001314	0.013488	-0.95719	Fam92a
46904	-1.11711	9.897544	-3.89851	0.001238	0.013202	-0.90068	Pycard
8625	-1.11713	13.17434	-3.37093	0.003807	0.022874	-1.96692	Inppl1
25084	-1.11718	8.664056	-4.46299	0.000376	0.008192	0.237704	Metap1d
62428	-1.11782	10.57495	-3.73292	0.001761	0.015317	-1.23618	Akap9
22750	-1.11785	12.46581	-3.58016	0.002438	0.018131	-1.54528	Msi2
26452	-1.11786	9.617	-3.6812	0.001966	0.01616	-1.34091	Hspb11
57153	-1.11808	10.89274	-4.06655	0.000866	0.011414	-0.56044	Nsd1
45345	-1.11823	9.639904	-3.97251	0.001058	0.012341	-0.75077	Cpox
17631	-1.11852	10.73113	-3.55559	0.00257	0.018628	-1.59493	Tars
22280	-1.11857	12.17939	-3.25499	0.00487	0.026163	-2.19909	Lamp2
52501	-1.11874	11.54776	-3.22042	0.00524	0.027385	-2.26807	Entpd2
47148	-1.11893	10.24026	-4.40407	0.000425	0.008651	0.119661	Fam126a
37077	-1.11903	11.42273	-3.76498	0.001645	0.014895	-1.17124	Metap2

22433	-1.11907	10.48162	-3.49608	0.002917	0.019915	-1.71504	Jagn1
9485	-1.11916	11.68292	-3.85541	0.001357	0.013729	-0.98799	Ppa1
11495	-1.11919	9.755139	-3.85319	0.001363	0.013751	-0.9925	Mccc2
24698	-1.11923	12.25819	-3.60791	0.002299	0.017522	-1.48919	Spns1
1008	-1.11924	7.467395	-4.29022	0.00054	0.009554	-0.10906	Akap5
41089	-1.11927	10.21347	-3.36858	0.003826	0.022894	-1.97163	Kpna3
57946	-1.11966	10.8322	-4.2853	0.000546	0.009554	-0.11896	Ndufaf2
11763	-1.11992	12.5292	-4.12592	0.000764	0.010788	-0.44043	Trim63
34022	-1.11995	10.2801	-3.66572	0.002032	0.016461	-1.37223	Rsrc2
40935	-1.12033	12.59293	-3.555	0.002573	0.018637	-1.59612	Rnf114
36012	-1.12034	9.407359	-4.99709	0.000125	0.005707	1.293638	Qrsl1
43686	-1.12048	8.802446	-4.17021	0.000696	0.010396	-0.35098	Crbn
31862	-1.12051	8.904264	-4.60464	0.00028	0.007327	0.520355	AW209491
48644	-1.1206	8.787438	-3.89235	0.001254	0.013211	-0.91314	Immp2l
51938	-1.12063	9.046255	-4.46479	0.000374	0.008178	0.241305	Spryd4
26241	-1.12076	12.3063	-4.05353	0.000891	0.01149	-0.58679	Zbtb20
20478	-1.12076	7.166392	-4.54827	0.000314	0.007633	0.408071	Pla2g4e
40233	-1.12103	7.4805	-3.4542	0.003189	0.020805	-1.79945	Hspa1a
59892	-1.12112	8.29568	-3.03195	0.007801	0.03451	-2.64137	Itga8
53232	-1.12114	12.43153	-3.55084	0.002596	0.018727	-1.60453	Hk2
38364	-1.1214	11.17056	-3.5605	0.002543	0.018513	-1.58501	Polr2f
15772	-1.12149	11.0372	-4.1011	0.000805	0.011093	-0.49058	Chmp7
19044	-1.12155	9.441578	-3.41704	0.003451	0.021703	-1.87425	Lrrc17
41531	-1.12162	9.646679	-3.9143	0.001197	0.013013	-0.86867	Nubpl
19549	-1.12203	9.145756	-3.87978	0.001288	0.013363	-0.93862	Akr1e1
43156	-1.12204	9.650059	-4.65258	0.000253	0.007106	0.61563	Mov10l1

10597	-1.12226	13.8311	-4.13975	0.000742	0.010675	-0.41248	Pam16
62888	-1.12235	11.88879	-3.45357	0.003193	0.020825	-1.80071	Commd3
8407	-1.12267	13.13229	-4.23051	0.000613	0.010087	-0.22933	Dcaf11
565	-1.12313	10.16065	-3.68281	0.001959	0.016149	-1.33765	Cand2
9998	-1.1237	10.14953	-3.34073	0.004059	0.023623	-2.0275	Nck1
54288	-1.1239	11.06784	-4.16638	0.000701	0.010424	-0.35871	Sobp
14764	-1.12393	11.19012	-4.01662	0.000963	0.011923	-0.66147	Rfng
15517	-1.12424	11.27121	-3.7846	0.001578	0.014645	-1.13148	Ethe1
39894	-1.12439	11.12006	-3.42032	0.003427	0.021619	-1.86764	Snrpb2
58949	-1.12452	11.17072	-3.76795	0.001634	0.014876	-1.16522	Sdhaf1
48523	-1.12453	12.75168	-4.36323	0.000463	0.008987	0.03772	Rab12
875	-1.12503	13.32055	-4.18087	0.00068	0.010327	-0.32946	Ahsa1
48592	-1.12505	12.23411	-3.39864	0.003589	0.022148	-1.91125	Map4k4
42994	-1.12506	12.07075	-3.56767	0.002504	0.018361	-1.57053	Elavl1
49301	-1.12519	11.55263	-4.10123	0.000805	0.011093	-0.49032	Tsc22d4
40538	-1.12521	13.73912	-4.42351	0.000408	0.008543	0.158641	Mrpl2
8822	-1.12534	9.4844	-3.46212	0.003136	0.020634	-1.7835	Dcakd
2975	-1.12536	13.28365	-3.61659	0.002256	0.017363	-1.47164	Urod
28708	-1.12536	9.931783	-4.05624	0.000886	0.01149	-0.58131	Dolpp1
50498	-1.12537	10.70049	-3.91018	0.001208	0.013079	-0.87703	Hic1
12404	-1.12566	10.45168	-3.24722	0.00495	0.026398	-2.2146	Crebzf
40477	-1.12577	9.692065	-4.82739	0.000176	0.006166	0.961194	E130311K13Rik
60186	-1.12603	12.03854	-2.77372	0.013379	0.048548	-3.14322	Rangrf
62203	-1.12605	10.47816	-4.97182	0.000131	0.005815	1.244331	H2-Ke2
57969	-1.12605	9.889935	-3.894	0.00125	0.013205	-0.90982	Mlst8
49996	-1.12608	10.28324	-3.12704	0.006384	0.030681	-2.45364	Jam3

12271	-1.12608	12.14272	-3.48262	0.003002	0.020256	-1.74219	Nono
21878	-1.12624	12.90432	-3.77591	0.001607	0.014738	-1.14909	Mrpl13
28876	-1.12633	9.523342	-4.13461	0.00075	0.010741	-0.42287	Tmem218
31078	-1.12642	11.00906	-3.94357	0.001125	0.012635	-0.80939	Dnlz
46760	-1.12642	9.400962	-4.49195	0.000354	0.007971	0.295611	Ankrd50
44455	-1.12647	16.26893	-6.57635	5.80E-06	0.002346	4.203511	Cs
44809	-1.12654	9.818454	-4.23624	0.000605	0.010014	-0.21779	Kdm4a
21511	-1.12654	10.34499	-4.19269	0.000664	0.010247	-0.30561	Tmem51
40101	-1.12725	9.702779	-3.93156	0.001154	0.012818	-0.83372	Nol11
31949	-1.12775	13.86104	-4.04576	0.000906	0.011576	-0.60251	Grinl1a
55388	-1.12779	12.3427	-3.67381	0.001997	0.016322	-1.35587	Elof1
35780	-1.1282	9.614975	-4.27162	0.000562	0.009657	-0.14651	Slc31a1
41160	-1.1282	9.701559	-3.41641	0.003456	0.021712	-1.87552	Mettl16
50052	-1.12826	10.65169	-3.73362	0.001758	0.015306	-1.23476	D030056L22Rik
55554	-1.12877	8.589701	-4.70667	0.000226	0.006827	0.722872	Gm2695
20839	-1.12884	10.04602	-4.24806	0.00059	0.009909	-0.19396	Kcnd3
46500	-1.12904	10.37839	-3.61397	0.002269	0.017417	-1.47695	Osbpl3
28148	-1.12924	9.348255	-4.17869	0.000683	0.010338	-0.33386	Dnajc12
22518	-1.12924	13.23731	-4.98028	0.000129	0.00578	1.260846	Cox6b1
47305	-1.12949	14.9387	-3.16269	0.005921	0.029382	-2.38291	Clu
15582	-1.12952	12.57557	-4.28053	0.000551	0.00958	-0.12857	Rab14
1124	-1.12959	10.14805	-3.52685	0.002732	0.019267	-1.65296	Zmym3
55393	-1.12963	14.51041	-4.60753	0.000278	0.007325	0.526088	Gm7065
39639	-1.12964	9.853893	-3.95949	0.001087	0.012478	-0.77715	Lats2
31004	-1.12972	12.0946	-4.09547	0.000815	0.011156	-0.50196	Rab1
37547	-1.12984	11.40645	-4.2135	0.000635	0.0101	-0.26364	Ptges2

4778	-1.12991	8.605907	-4.2355	0.000606	0.010014	-0.21926	Lig4
51974	-1.13	9.511279	-3.29654	0.004459	0.024985	-2.11605	Kctd3
6427	-1.1304	12.41602	-4.34661	0.00048	0.009113	0.004333	Slc25a12
3852	-1.13041	10.8108	-3.13752	0.006244	0.030326	-2.43286	Pcna
56648	-1.13042	11.47059	-3.93038	0.001157	0.012818	-0.83611	Cbr4
27432	-1.13045	7.368033	-5.9651	1.83E-05	0.002941	3.120178	Gm6416
33636	-1.13055	9.719693	-3.91831	0.001187	0.012963	-0.86055	Wrnip1
12158	-1.13058	9.748159	-3.83367	0.001421	0.013969	-1.03206	Snx16
22705	-1.13077	11.73232	-4.04956	0.000898	0.011523	-0.59482	Usp2
50018	-1.13081	10.03282	-3.71077	0.001846	0.015643	-1.28104	Baz2b
33612	-1.13096	13.51358	-3.5051	0.002861	0.019749	-1.69685	Mrps16
62544	-1.13106	12.53761	-3.76879	0.001632	0.014858	-1.16351	Ttc3
14867	-1.13108	9.664814	-3.79398	0.001546	0.014574	-1.11249	Zfp131
11699	-1.13111	13.80144	-5.24843	7.48E-05	0.004894	1.779871	Timm17a
13008	-1.13146	13.17018	-3.7709	0.001624	0.014835	-1.15923	Paip2
16255	-1.13148	12.07729	-2.8966	0.010361	0.041217	-2.90604	Rcn3
7998	-1.13178	10.9561	-3.45938	0.003154	0.020669	-1.78903	Rab2a
21824	-1.13179	11.12829	-3.34238	0.004045	0.023591	-2.02421	Tmem132a
11880	-1.13188	11.31554	-3.9249	0.00117	0.012871	-0.84722	Psmd6
47801	-1.13195	11.12139	-3.93068	0.001156	0.012818	-0.8355	Zhx3
27435	-1.13196	9.448412	-4.54648	0.000316	0.007649	0.404499	Nedd4l
20951	-1.13213	12.73132	-4.04211	0.000913	0.011622	-0.60989	Mrpl41
25326	-1.1322	14.1327	-3.73526	0.001752	0.015284	-1.23144	Chchd1
28785	-1.13234	10.78246	-3.82103	0.00146	0.014191	-1.05767	Fance
6240	-1.13275	9.571865	-4.00016	0.000998	0.012046	-0.6948	Tmem14a
5201	-1.13325	11.05521	-4.17233	0.000693	0.010378	-0.3467	Hagh

21519	-1.13354	8.593499	-3.96775	0.001069	0.01237	-0.76041	Ado
25335	-1.13391	12.80286	-4.05478	0.000888	0.01149	-0.58426	Serinc1
11424	-1.13427	13.40629	-3.11852	0.0065	0.031028	-2.4705	Nudt4
18928	-1.13446	8.590011	-4.52641	0.000329	0.007758	0.364452	Magohb
45746	-1.13461	10.72112	-3.50593	0.002856	0.019739	-1.69517	Srsf3
3563	-1.13524	9.469488	-3.70615	0.001864	0.015677	-1.2904	Ahcyl1
62173	-1.13525	10.49746	-3.90359	0.001225	0.013165	-0.89037	B4galt3
29779	-1.13533	9.953261	-3.27554	0.004662	0.02559	-2.15805	Sp1
4019	-1.13536	10.85986	-3.26794	0.004738	0.025809	-2.17323	Casc3
45600	-1.13539	11.89909	-4.83777	0.000173	0.006133	0.981631	Ptp4a3
19323	-1.13572	10.85354	-3.48939	0.002959	0.020032	-1.72853	Otud4
30611	-1.13585	12.48153	-4.06302	0.000873	0.011432	-0.56758	Tmem141
33810	-1.1359	11.19582	-4.16016	0.000711	0.010492	-0.37127	Coasy
60078	-1.13598	9.591716	-3.71487	0.00183	0.015573	-1.27274	Exosc8
16666	-1.13609	9.838808	-3.95445	0.001099	0.012519	-0.78736	Gpatch8
38160	-1.13614	8.244104	-4.43487	0.000399	0.008456	0.181405	Lcor
59082	-1.1364	10.00291	-5.28421	6.96E-05	0.004827	1.848457	Tln2
3292	-1.13644	15.776	-4.74835	0.000208	0.006636	0.805325	Ndufa1
41860	-1.13648	12.29509	-3.66786	0.002023	0.016409	-1.36791	Hibch
57960	-1.13655	8.413243	-4.12816	0.000761	0.010779	-0.43591	Trmt11
6446	-1.1366	11.95458	-3.43036	0.003355	0.021378	-1.84744	Jkamp
23478	-1.13695	14.26235	-4.28527	0.000546	0.009554	-0.11903	Tmem59
39940	-1.13701	13.40569	-4.374	0.000453	0.008944	0.059341	Aurkaip1
35491	-1.13708	10.13191	-4.45622	0.000381	0.008263	0.224156	Med4
34428	-1.13712	11.81297	-3.86805	0.001321	0.013502	-0.9624	Qdpr
3476	-1.13723	10.06329	-4.98403	0.000128	0.005764	1.268162	Csl

15908	-1.13729	11.5726	-4.07633	0.000849	0.011306	-0.54067	Echdc3
27475	-1.13738	9.755234	-3.52582	0.002738	0.01928	-1.65504	Uhrf2
28552	-1.13748	8.716685	-4.18379	0.000676	0.010327	-0.32356	Erlec1
14775	-1.13749	9.667095	-3.4018	0.003565	0.022063	-1.90489	Ano1
52975	-1.13761	7.889498	-3.48307	0.002999	0.020248	-1.74127	Rgs18
11991	-1.13768	10.94398	-4.11944	0.000775	0.010871	-0.45351	Mal
47859	-1.13783	13.29368	-3.75351	0.001685	0.015038	-1.19446	Acaa1a
6402	-1.13786	12.57841	-4.46595	0.000373	0.008171	0.243612	Entpd4
44880	-1.13786	8.415588	-3.78229	0.001585	0.014676	-1.13615	Tlcd2
24326	-1.13788	11.72713	-3.46039	0.003147	0.020649	-1.78697	Ebp
21335	-1.1379	10.01166	-3.71783	0.001819	0.015552	-1.26674	Nab2
34236	-1.13803	10.62949	-4.03197	0.000932	0.011729	-0.6304	Bcas2
41168	-1.13811	11.93216	-3.89796	0.001239	0.013202	-0.90179	Zfp703
27998	-1.13813	11.82431	-3.47414	0.003056	0.020437	-1.75927	Pten
33548	-1.13831	10.22022	-3.76178	0.001656	0.014915	-1.17771	Zfp1
29321	-1.13838	10.46164	-3.60066	0.002334	0.017655	-1.50386	Cyb5d2
17	-1.13843	10.94024	-4.01623	0.000964	0.011923	-0.66227	Cops3
49403	-1.13847	11.96901	-5.00043	0.000124	0.005707	1.300142	Sdr39u1
58657	-1.13865	12.89033	-3.37659	0.003761	0.022767	-1.95556	Dexi
56160	-1.13935	12.62042	-4.0795	0.000843	0.011277	-0.53425	Mrps7
48008	-1.13962	11.21868	-3.39438	0.003622	0.022279	-1.9198	Dynlt1c
25247	-1.13963	12.27894	-3.72321	0.001798	0.015472	-1.25583	Zfp46
55153	-1.14046	11.63958	-4.21829	0.000629	0.0101	-0.25397	Ppm1l
43381	-1.14068	12.23911	-3.52862	0.002722	0.019218	-1.6494	Me1
10216	-1.14079	14.43805	-4.2247	0.00062	0.010087	-0.24104	Hspa5
13633	-1.1412	8.726369	-4.30434	0.000524	0.009438	-0.08065	Nlrp10

35225	-1.14136	12.06438	-3.85509	0.001358	0.013732	-0.98865	Dus1l
14390	-1.14153	11.10086	-3.87319	0.001306	0.013449	-0.95197	Tcea3
48448	-1.14159	9.955289	-4.12995	0.000758	0.010755	-0.43227	Tsfm
56478	-1.14165	13.38801	-4.37459	0.000452	0.008944	0.060516	Coq6
14280	-1.14211	9.89694	-4.85346	0.000167	0.006078	1.012467	Rufy1
23722	-1.14218	12.92976	-3.41687	0.003453	0.021703	-1.87459	Spcs1
40742	-1.1422	11.00126	-3.19854	0.005488	0.028092	-2.31164	Fkbp10
60988	-1.14222	11.91507	-3.57129	0.002485	0.018311	-1.56322	Klhl9
20724	-1.14225	10.07537	-3.54748	0.002614	0.018768	-1.61132	Oxsr1
26652	-1.14234	14.06826	-4.2071	0.000644	0.010117	-0.27653	Pdcd5
59760	-1.14243	11.2843	-3.64586	0.00212	0.016831	-1.41243	Thap4
3515	-1.14249	9.141083	-4.3966	0.000432	0.008717	0.104692	Tiprl
39840	-1.14317	10.07808	-4.08504	0.000833	0.011248	-0.52305	Pqlc1
7917	-1.14323	9.457991	-3.75001	0.001698	0.015061	-1.20157	Ilf2
31489	-1.14345	9.812379	-4.01138	0.000974	0.011974	-0.67209	Msh3
22596	-1.1436	12.88362	-3.43274	0.003338	0.021299	-1.84265	Psma1
52068	-1.14365	10.84431	-4.07072	0.000859	0.011362	-0.55201	Insig2
38234	-1.14373	10.20699	-4.28684	0.000544	0.009554	-0.11587	Snrpb
60878	-1.144	12.17324	-3.94717	0.001116	0.012572	-0.80209	Fgfr1op2
58150	-1.14441	11.40259	-3.59009	0.002387	0.017879	-1.52522	Micall1
17502	-1.14444	11.13129	-3.41702	0.003451	0.021703	-1.87428	Commd7
15475	-1.14451	10.99671	-5.13785	9.35E-05	0.005355	1.566891	Fxn
61704	-1.14471	13.74161	-5.08907	0.000103	0.0055	1.472473	Hibadh
56954	-1.1453	8.445763	-3.90453	0.001222	0.013159	-0.88847	Prkar2b
33432	-1.14564	8.888049	-3.78097	0.00159	0.014684	-1.13883	Zfp326
6950	-1.14587	12.25896	-3.38013	0.003733	0.022676	-1.94844	Rgs5

58169	-1.14637	11.62524	-4.03869	0.000919	0.011683	-0.61681	Hmgb3
43006	-1.14638	9.188062	-5.12425	9.62E-05	0.00542	1.540604	Llgl2
52150	-1.14646	12.14976	-3.74294	0.001724	0.015191	-1.21587	Hbxip
2391	-1.14647	10.11723	-3.92474	0.001171	0.012871	-0.84753	Ralgapa2
7496	-1.14655	9.969894	-3.37603	0.003766	0.022782	-1.95668	Kcnj12
39448	-1.14658	8.390681	-5.37814	5.77E-05	0.004414	2.02771	Fastkd3
8088	-1.14659	11.3664	-3.96746	0.001069	0.01237	-0.761	Sypl
46096	-1.1466	10.5487	-3.28884	0.004532	0.025185	-2.13145	Ostc
19459	-1.14672	12.35483	-3.39408	0.003624	0.022284	-1.9204	Csnk1d
44322	-1.14678	12.27107	-4.1991	0.000655	0.010181	-0.29268	Ptpmt1
30712	-1.14682	9.124554	-4.43973	0.000395	0.008418	0.191127	Slc30a5
34102	-1.14686	14.79002	-5.11077	9.88E-05	0.005444	1.514512	Ndufb5
36092	-1.14704	12.91719	-4.19486	0.00066	0.010244	-0.30122	Btbd1
12462	-1.14704	8.847927	-3.54397	0.002634	0.018815	-1.61839	Ncapd3
32702	-1.14714	13.70208	-3.36464	0.003858	0.022944	-1.97954	Sdpr
2204	-1.14731	10.06739	-3.42649	0.003383	0.021485	-1.85524	Fbxw7
59149	-1.14759	11.87226	-3.57088	0.002487	0.018311	-1.56405	Rbms1
24986	-1.14762	10.96505	-3.98316	0.001034	0.012241	-0.72921	Mepce
6039	-1.14809	14.26551	-4.40394	0.000425	0.008651	0.119412	Hsd17b10
27467	-1.14809	8.650232	-4.98214	0.000128	0.005774	1.264486	Slc41a1
33553	-1.14813	13.48101	-4.50108	0.000347	0.007904	0.313868	Tufm
32848	-1.14833	8.91528	-3.95017	0.001109	0.012525	-0.79601	Sort1
47970	-1.14845	9.421246	-3.83533	0.001416	0.013953	-1.02869	Magt1
4846	-1.14853	11.89022	-2.83841	0.011697	0.04463	-3.01877	Psma6
24475	-1.14856	9.807697	-3.79911	0.00153	0.014474	-1.10208	Itsn2
2746	-1.1486	9.289917	-3.96608	0.001072	0.01238	-0.7638	Lzic

33313	-1.14887	10.117	-4.14883	0.000728	0.010604	-0.39414	Papd5
14469	-1.1495	9.696027	-4.42787	0.000404	0.008482	0.167369	Dot1l
34540	-1.14959	11.69756	-4.03024	0.000936	0.011742	-0.63392	Rhbdd3
46513	-1.14975	8.414828	-5.38207	5.73E-05	0.004414	2.035179	Nt5c3l
3374	-1.14984	12.92998	-3.45993	0.00315	0.020663	-1.78791	Map1lc3b
11787	-1.14993	9.698894	-3.35048	0.003976	0.023361	-2.00795	Kbtbd4
23780	-1.15005	11.38187	-2.96363	0.009005	0.03784	-2.77537	Trappc4
4245	-1.15011	9.339348	-4.01478	0.000967	0.011933	-0.6652	Cradd
37575	-1.15017	13.08728	-4.5614	0.000306	0.007568	0.434237	Anxa6
21584	-1.15017	11.78499	-3.57914	0.002444	0.018153	-1.54736	Rsl1d1
2517	-1.1502	13.29687	-4.30774	0.000521	0.009396	-0.0738	Grpel1
50529	-1.1506	10.30124	-3.76161	0.001657	0.014915	-1.17805	Pld2
49133	-1.1507	8.51927	-5.53912	4.19E-05	0.00398	2.332154	Mlxip
37591	-1.15103	15.53373	-5.10656	9.97E-05	0.005451	1.506348	Ak1
16405	-1.15106	11.88467	-4.49427	0.000352	0.007957	0.300259	Def8
44446	-1.15113	9.847999	-4.5159	0.000336	0.007874	0.343471	Gja1
42352	-1.15118	10.98615	-3.74007	0.001734	0.015225	-1.2217	Eed
56449	-1.15174	8.490952	-5.97565	1.79E-05	0.002925	3.13936	Pank1
3294	-1.15185	9.422303	-4.10717	0.000795	0.011053	-0.47832	Zfx
27072	-1.15192	12.25798	-3.80839	0.0015	0.014345	-1.08328	Lypla2
8347	-1.15202	10.05618	-3.9351	0.001145	0.012794	-0.82655	Dhodh
50082	-1.15213	12.50136	-3.22131	0.00523	0.027369	-2.2663	Dpm3
46074	-1.15223	9.817105	-3.7853	0.001575	0.014645	-1.13007	Slc31a2
1676	-1.15225	11.23359	-3.99617	0.001006	0.012068	-0.70286	Polr1a
59630	-1.15259	15.66535	-4.26908	0.000565	0.009682	-0.15162	Hint1
32652	-1.15261	12.10203	-4.39091	0.000437	0.008789	0.093272	Pank4

32443	-1.15262	11.00573	-5.02633	0.000117	0.005645	1.350601	Pin1-ps1
5212	-1.15287	12.67508	-4.59824	0.000283	0.007333	0.50762	Atad3a
11363	-1.15325	8.98925	-4.95915	0.000135	0.005828	1.219601	Klhl24
36656	-1.1535	11.40482	-3.79506	0.001543	0.014565	-1.11029	Pepd
16402	-1.1536	10.41897	-4.30924	0.000519	0.00939	-0.07079	Mapk9
18519	-1.15378	10.82208	-3.9297	0.001159	0.012818	-0.83748	Lars2
81	-1.15418	11.17786	-3.90257	0.001227	0.013169	-0.89244	Ckap5
119	-1.15439	9.665154	-3.99498	0.001009	0.012068	-0.70527	Tmem149
46343	-1.15531	13.5312	-3.79101	0.001556	0.014594	-1.11849	Tef
28536	-1.15538	8.926184	-4.16667	0.000701	0.010424	-0.35813	Zmym2
2206	-1.15561	12.37024	-4.08879	0.000827	0.011211	-0.51547	LOC100042049
56369	-1.15565	10.74736	-4.28665	0.000544	0.009554	-0.11624	Slc25a33
57391	-1.15573	10.98753	-4.00768	0.000982	0.011995	-0.67957	Ppp2r2a
44105	-1.15576	14.37612	-3.10079	0.006748	0.031748	-2.5056	Hbb-b2
18620	-1.15588	10.86186	-3.48662	0.002976	0.020126	-1.73412	Sec11a
2013	-1.1562	13.91097	-4.09786	0.000811	0.011114	-0.49713	Scp2
56348	-1.15624	8.796118	-4.63086	0.000265	0.007222	0.572476	Rif1
61345	-1.15625	10.68923	-4.37076	0.000456	0.008954	0.052825	D930015E06Rik
32860	-1.15644	11.73624	-4.4813	0.000362	0.008029	0.274315	Ttc12
41569	-1.15648	12.95895	-4.20554	0.000646	0.010122	-0.27969	Fars2
46850	-1.15649	12.10053	-2.93913	0.009479	0.038983	-2.82322	Zfp36l1
61864	-1.15658	8.763005	-4.23586	0.000606	0.010014	-0.21854	Dph2
9877	-1.1566	10.18643	-3.83871	0.001406	0.013923	-1.02185	Naa38
59608	-1.15666	10.9763	-4.59139	0.000287	0.007378	0.49397	Ptcd2
20084	-1.15682	10.75558	-4.05452	0.000889	0.01149	-0.58479	Chkb
46377	-1.15684	9.876899	-4.05594	0.000886	0.01149	-0.58191	Supv3l1

43809	-1.15702	10.10305	-4.05508	0.000888	0.01149	-0.58364	Polr2b
33445	-1.15748	10.17901	-4.00171	0.000994	0.012039	-0.69165	Iars2
698	-1.15769	10.76322	-4.18838	0.00067	0.010276	-0.31429	Mobkl3
28046	-1.15772	10.04977	-3.80356	0.001515	0.014418	-1.09307	Ssbp3
47036	-1.15782	8.039893	-3.77291	0.001617	0.014799	-1.15517	Gfra3
29566	-1.15789	9.682738	-3.81573	0.001476	0.014271	-1.06841	Rap2a
7480	-1.15792	9.907715	-3.46052	0.003146	0.020649	-1.78673	Ftsj1
54032	-1.15816	11.65244	-3.42279	0.003409	0.021579	-1.86268	Ppap2b
28727	-1.15836	9.076084	-3.89909	0.001236	0.013202	-0.89949	Cct6a
36531	-1.15887	8.866828	-3.91093	0.001206	0.013064	-0.87551	AK010878
33795	-1.15907	12.2975	-4.12186	0.000771	0.010847	-0.44863	Dnajb5
11550	-1.1592	11.63129	-4.21659	0.000631	0.0101	-0.2574	Raph1
13915	-1.15937	11.8697	-4.00148	0.000995	0.012039	-0.69211	Nop10
43816	-1.15945	11.89824	-3.77912	0.001596	0.014708	-1.14258	Cobra1
57221	-1.15947	13.44178	-3.53498	0.002685	0.01902	-1.63656	Pcbp1
32775	-1.15949	9.36481	-3.64865	0.002107	0.016791	-1.40679	Abhd5
24225	-1.15972	9.637757	-3.95508	0.001098	0.012515	-0.78607	Arhgef17
17729	-1.15991	10.26164	-4.02809	0.00094	0.011752	-0.63827	Man1a2
12251	-1.1601	8.124804	-4.43375	0.0004	0.008456	0.179158	Zfp809
13610	-1.1602	9.685486	-4.06382	0.000872	0.011419	-0.56597	Amy1
11874	-1.1605	10.51037	-5.25849	7.33E-05	0.004873	1.799165	Clpp
32915	-1.16059	9.397986	-4.16237	0.000707	0.010472	-0.3668	Rnf139
1100	-1.16063	11.73785	-3.6352	0.002169	0.017052	-1.43401	Spast
2943	-1.1608	8.144064	-4.0523	0.000893	0.01149	-0.58928	Ccdc117
6767	-1.16102	10.96257	-4.2332	0.000609	0.010041	-0.2239	Cux1
13506	-1.16107	7.871966	-6.47241	7.03E-06	0.002346	4.023353	D9Ertd402e

38899	-1.16126	11.09258	-3.76507	0.001645	0.014895	-1.17106	Gpbp1
19334	-1.16133	14.09858	-4.16685	0.000701	0.010424	-0.35775	Lrtm1
61280	-1.16135	9.128834	-4.14468	0.000734	0.010615	-0.40253	Cmpk1
5825	-1.16143	10.51693	-3.76785	0.001635	0.014876	-1.16543	Khk
51289	-1.16147	10.54741	-3.92468	0.001171	0.012871	-0.84766	Eif3e
48205	-1.16154	8.461856	-5.24537	7.53E-05	0.004894	1.77399	Slc22a5
48156	-1.16198	10.93203	-3.91531	0.001195	0.013013	-0.86663	Fbxo9
43359	-1.16218	10.72804	-4.27766	0.000555	0.009599	-0.13434	Ncoa1
37039	-1.16225	11.34734	-4.43823	0.000396	0.008424	0.188132	Amz2
19147	-1.16237	9.921157	-3.93215	0.001153	0.012818	-0.83251	Hectd1
56140	-1.16245	10.75758	-4.39898	0.00043	0.008694	0.109466	Fnip1
31846	-1.16251	11.66478	-3.50875	0.002839	0.019696	-1.68949	Fbxw11
645	-1.16259	11.08842	-2.86624	0.011038	0.043126	-2.96493	Capza2
8134	-1.16262	8.594672	-4.70679	0.000226	0.006827	0.723117	Smpd2
9812	-1.16289	9.071594	-3.59689	0.002353	0.017724	-1.51148	Slc6a9
58338	-1.16296	8.923098	-4.21515	0.000633	0.0101	-0.26031	Ebag9
29112	-1.16306	14.06114	-4.20433	0.000647	0.010126	-0.28211	Ndufc2
46604	-1.16326	9.415109	-4.34842	0.000478	0.009113	0.007971	Nit2
46048	-1.16366	10.05649	-4.18915	0.000668	0.010268	-0.31275	Gga2
46054	-1.16373	12.90398	-4.35328	0.000473	0.009089	0.017725	Akt2
57163	-1.16386	14.34121	-4.31956	0.000508	0.009336	-0.05003	Akr1b3
38424	-1.16388	13.81723	-4.45009	0.000386	0.008301	0.211877	Mrpl23
15974	-1.16396	9.609921	-4.16279	0.000707	0.010472	-0.36596	Zrsr1
13087	-1.16399	10.58287	-3.57134	0.002485	0.018311	-1.56312	Zfp655
32299	-1.16411	10.24499	-4.22855	0.000615	0.010087	-0.23329	Tti1
58578	-1.16445	10.90843	-4.40005	0.000429	0.008694	0.111601	Herc4

43755	-1.16448	10.71663	-3.76617	0.001641	0.014895	-1.16882	Pmpca
16566	-1.16479	11.20623	-4.15257	0.000722	0.010601	-0.38659	Anapc1
25107	-1.16493	11.65332	-4.21061	0.000639	0.010115	-0.26945	Smu1
62407	-1.16526	9.980844	-3.71039	0.001848	0.015649	-1.2818	Mina
6462	-1.1654	7.690099	-5.36991	5.87E-05	0.004449	2.012038	Slc25a16
13308	-1.16558	13.65936	-4.36804	0.000459	0.008954	0.047374	Mrpl30
4108	-1.16592	12.68351	-3.39786	0.003595	0.022168	-1.9128	Hmgn1
41549	-1.16658	11.85763	-4.28022	0.000552	0.00958	-0.12919	E2f6
30343	-1.16668	10.74685	-3.36154	0.003884	0.023027	-1.98577	Tsen54
16886	-1.16669	13.72166	-4.27859	0.000554	0.009598	-0.13247	Bola3
24010	-1.16687	12.32352	-4.24727	0.000591	0.009918	-0.19556	Mmaa
9597	-1.16695	13.43696	-4.22529	0.000619	0.010087	-0.23985	Fdx1
27398	-1.16769	13.01707	-5.43294	5.17E-05	0.004303	2.131741	Art1
46581	-1.16829	8.885463	-4.59583	0.000285	0.007333	0.502812	Ept1
31621	-1.16832	9.094488	-3.18281	0.005674	0.028611	-2.34293	Ecm2
42689	-1.16845	11.61092	-3.8308	0.00143	0.014017	-1.03786	Snrnp27
17619	-1.16882	9.467077	-3.99818	0.001002	0.012061	-0.6988	Taf12
3056	-1.16892	14.71218	-4.03054	0.000935	0.011742	-0.63331	Gm5623
60374	-1.16893	13.66175	-4.3578	0.000469	0.009046	0.026806	Mat2a
1843	-1.16894	13.35173	-4.00551	0.000986	0.01201	-0.68397	Fam54b
32755	-1.16894	12.32734	-4.65373	0.000253	0.007106	0.617907	Aarsd1
27414	-1.16911	11.1735	-4.93359	0.000142	0.005859	1.169625	Mavs
49406	-1.1692	13.05357	-4.2151	0.000633	0.0101	-0.2604	Cxx1b
29252	-1.16928	8.583435	-4.94179	0.000139	0.005859	1.185648	Ppil3
6910	-1.16928	11.73217	-5.42978	5.21E-05	0.004313	2.125747	Usf2
60474	-1.16952	8.751128	-3.91256	0.001202	0.013032	-0.8722	Gm7265

21608	-1.16952	11.23373	-3.92747	0.001164	0.012845	-0.84199	Dnajb2
58187	-1.16953	13.19407	-4.12705	0.000762	0.010783	-0.43814	Pmpcb
1315	-1.16992	9.361439	-4.26815	0.000566	0.009682	-0.15349	Mterfd1
27039	-1.16993	11.08612	-3.69719	0.0019	0.015852	-1.30854	Lass4
29076	-1.17007	10.42918	-3.66119	0.002052	0.016558	-1.3814	Acss2
58688	-1.17014	9.012396	-5.09751	0.000102	0.005481	1.488831	Gpam
41042	-1.17023	11.83123	-3.75336	0.001686	0.015038	-1.19478	Eif1ax
8569	-1.1703	12.48814	-3.63849	0.002153	0.016977	-1.42733	Trim35
36808	-1.17033	10.41193	-4.62543	0.000268	0.007229	0.561701	Vps25
28802	-1.17045	9.323118	-3.84375	0.001391	0.013865	-1.01163	Stard8
47571	-1.17075	11.49115	-5.54896	4.11E-05	0.00398	2.35066	Pla2g16
31046	-1.17077	10.97823	-4.27364	0.000559	0.009638	-0.14243	Rnf167
24725	-1.17083	10.07087	-3.73876	0.001739	0.015236	-1.22436	Farsb
20994	-1.1709	8.275347	-4.83762	0.000173	0.006133	0.981332	Cdk20
59102	-1.17131	11.52771	-3.68928	0.001933	0.016018	-1.32454	Pno1
33386	-1.17134	10.52384	-3.38797	0.003671	0.022464	-1.9327	Smchd1
49189	-1.17169	8.344476	-4.76182	0.000202	0.006581	0.831928	Lingo3
61796	-1.17185	9.115646	-4.94739	0.000138	0.005859	1.1966	Pdss2
15247	-1.17212	12.74052	-4.22467	0.00062	0.010087	-0.2411	Tceb1
33701	-1.17213	10.01957	-4.47388	0.000367	0.008089	0.259482	Eif2b1
48874	-1.17224	12.55909	-4.51792	0.000335	0.007855	0.347507	Map3k4
45910	-1.17225	12.85135	-4.07138	0.000858	0.011353	-0.55069	Mrps26
22410	-1.17252	8.836708	-4.15876	0.000713	0.010508	-0.37408	Fam149b
8504	-1.17277	10.10669	-3.40842	0.003515	0.021892	-1.89158	Med21
28080	-1.17277	9.650935	-4.28644	0.000544	0.009554	-0.11667	Mrps11
47261	-1.17308	15.58263	-6.56822	5.89E-06	0.002346	4.189478	Usmg5

46896	-1.1732	11.54249	-3.84061	0.0014	0.013919	-1.01799	Cdk4
2883	-1.17355	12.37982	-4.63495	0.000263	0.007219	0.580609	Vegfb
55108	-1.17357	10.55074	-3.5016	0.002883	0.019805	-1.7039	Emilin2
12743	-1.1737	10.23953	-3.56783	0.002503	0.018361	-1.57021	Snx13
8158	-1.17379	10.45841	-4.0247	0.000947	0.011777	-0.64513	Ccdc28a
30580	-1.1738	12.32836	-3.8619	0.001338	0.013616	-0.97484	Actr10
19530	-1.17383	10.93678	-3.59204	0.002378	0.01784	-1.52128	Sipa1
12201	-1.17391	9.255525	-4.19762	0.000657	0.010204	-0.29567	Chrm2
50276	-1.17393	8.592793	-4.45551	0.000382	0.008264	0.222723	Gin1
9288	-1.17394	10.70764	-2.81263	0.012341	0.0461	-3.06846	Serf1
47213	-1.1743	9.528931	-3.72906	0.001776	0.01537	-1.244	Taf2
34584	-1.17438	10.71281	-4.1903	0.000667	0.010268	-0.31044	R3hdm1
41215	-1.17444	9.494315	-4.90528	0.00015	0.005914	1.114172	Gm12824
22205	-1.17445	9.608435	-3.54895	0.002606	0.018758	-1.60835	Bcl10
46964	-1.17458	11.38221	-4.21757	0.00063	0.0101	-0.25542	Tfg
4007	-1.17474	11.10612	-3.21118	0.005343	0.02765	-2.28647	Cbfb
50944	-1.1749	10.11839	-3.9217	0.001178	0.012895	-0.85369	Kpna1
30278	-1.17502	10.83346	-4.32579	0.000501	0.009325	-0.03751	Surf1
18191	-1.17506	10.15078	-3.77403	0.001613	0.014782	-1.15289	Gls
35103	-1.17509	9.0428	-4.74969	0.000207	0.006636	0.807962	Gdpd1
56490	-1.17513	9.691762	-4.30077	0.000528	0.009458	-0.08782	Epm2aip1
6105	-1.17519	11.50974	-4.17452	0.000689	0.010365	-0.34226	Kri1
43241	-1.17535	12.05022	-4.16763	0.0007	0.010424	-0.35618	Bzw2
52989	-1.17542	11.87033	-4.93429	0.000142	0.005859	1.170992	Commd1
14562	-1.17562	12.15388	-3.64459	0.002126	0.016859	-1.415	Atp6v1e1
21355	-1.17579	10.14364	-4.22511	0.00062	0.010087	-0.24022	Arl1

34727	-1.17581	14.27573	-4.50783	0.000342	0.007904	0.327344	Eef1g
43407	-1.17587	7.562917	-3.5016	0.002883	0.019805	-1.70392	Kcnj3
37477	-1.17627	13.85426	-4.60514	0.000279	0.007327	0.521345	Mrpl4
40597	-1.17639	14.36158	-3.77371	0.001615	0.014783	-1.15354	Tesc
134	-1.1766	11.76652	-4.83762	0.000173	0.006133	0.981328	Mrpl28
14738	-1.1767	12.57346	-3.46378	0.003124	0.020606	-1.78015	Gm4130
53174	-1.1768	14.30132	-5.16142	8.92E-05	0.005274	1.612427	Sdhc
51014	-1.17731	12.68978	-3.67737	0.001982	0.016253	-1.34865	Gabarapl2
33203	-1.17746	10.87981	-4.03523	0.000926	0.011716	-0.62381	Spcs2
25294	-1.17757	10.37343	-4.28177	0.00055	0.009575	-0.12607	Wdr5
35942	-1.17765	10.46077	-4.93618	0.000141	0.005859	1.174692	Zfyve19
9206	-1.17776	10.69087	-4.32046	0.000507	0.009336	-0.04824	Dolk
32023	-1.1778	12.14252	-4.14232	0.000738	0.010638	-0.40729	Tbca
59798	-1.17781	10.84056	-3.81352	0.001483	0.014288	-1.07288	Wbp11
37314	-1.17801	10.66727	-3.8002	0.001526	0.014459	-1.09988	Rgl3
50027	-1.17811	10.25297	-3.84828	0.001378	0.013821	-1.00245	Slc30a9
61436	-1.1785	10.49937	-3.68489	0.001951	0.016103	-1.33344	Ppm1m
55062	-1.17879	11.9927	-3.82377	0.001451	0.01415	-1.05211	Zer1
46462	-1.17887	10.77847	-4.10723	0.000795	0.011053	-0.47818	Mapk1ip1
56684	-1.17937	11.15899	-3.2944	0.004479	0.025054	-2.12032	Rbm18
15022	-1.17952	12.90026	-4.57428	0.000298	0.007471	0.459901	Tmem143
52402	-1.17956	10.34548	-4.25899	0.000577	0.009765	-0.17194	Fam132a
26588	-1.18002	9.801111	-4.18032	0.000681	0.010327	-0.33057	Pex3
18966	-1.18053	9.541896	-3.42147	0.003419	0.021615	-1.86533	Lrrc33
2589	-1.18086	7.849409	-4.0725	0.000856	0.011339	-0.54842	Gm7120
16564	-1.18104	10.38146	-3.57611	0.00246	0.018215	-1.55349	Syne2

56634	-1.18147	10.54247	-4.36583	0.000461	0.008954	0.042935	Uqcc
36949	-1.18215	10.21807	-3.7022	0.00188	0.015749	-1.29838	Atg5
36529	-1.18216	12.42798	-3.82125	0.001459	0.014191	-1.05722	H3f3a
20591	-1.18257	9.738725	-2.77147	0.013442	0.0487	-3.14755	Folr2
41543	-1.18282	9.330915	-4.26289	0.000572	0.009722	-0.16409	C330018D20Rik
13830	-1.18304	11.99597	-4.21013	0.00064	0.010117	-0.27042	Spag9
43589	-1.18312	13.8541	-4.90639	0.00015	0.005914	1.11635	Acat1
30291	-1.18315	13.28527	-3.92356	0.001174	0.012879	-0.84992	Canx
36934	-1.18337	12.73289	-4.8657	0.000163	0.006036	1.03653	Samm50
61849	-1.18343	9.002632	-6.42493	7.67E-06	0.002382	3.940493	Crat
52019	-1.1836	11.97295	-3.4416	0.003276	0.021098	-1.82482	Ythdc1
17408	-1.18463	9.365702	-3.76902	0.001631	0.014858	-1.16306	Snapc3
17978	-1.18465	9.256586	-4.16041	0.00071	0.010492	-0.37077	Mtrf1l
11442	-1.18536	9.768775	-3.76882	0.001631	0.014858	-1.16346	Nhlrc1
42520	-1.18547	11.19174	-3.80682	0.001505	0.014364	-1.08645	Asxl1
47139	-1.18553	9.084787	-4.5732	0.000299	0.007471	0.45776	Prkrir
2708	-1.18578	8.952427	-4.66719	0.000246	0.007001	0.644622	Arl3
38074	-1.18581	10.6255	-4.21284	0.000636	0.0101	-0.26496	Hsd3b7
30268	-1.18591	12.4368	-4.2183	0.000629	0.0101	-0.25396	Mfn1
47152	-1.18625	11.29427	-3.74926	0.001701	0.015072	-1.20308	Hspa14
23953	-1.18629	9.428305	-3.73604	0.001749	0.015275	-1.22985	Cdk5
22012	-1.18675	9.52124	-4.16335	0.000706	0.010472	-0.36483	Lrfn4
23820	-1.18688	9.853088	-4.75493	0.000205	0.006627	0.818313	Sbk1
60244	-1.18706	9.739782	-3.70929	0.001852	0.01565	-1.28403	Rnd3
39386	-1.18712	12.34598	-4.0159	0.000965	0.011925	-0.66293	Trappc2l
2961	-1.18722	13.29484	-4.97759	0.00013	0.00578	1.255605	Psme1

16090	-1.18742	9.786946	-3.83399	0.00142	0.013969	-1.0314	Zbtb44
24821	-1.1877	9.461614	-3.94398	0.001124	0.012635	-0.80857	Zfp777
32835	-1.18822	9.165872	-3.49211	0.002942	0.019992	-1.72305	Fam43a
25998	-1.18827	14.54603	-4.97735	0.00013	0.00578	1.255129	Vdac3
57838	-1.1884	10.22885	-4.53794	0.000321	0.007678	0.387453	Fig4
14149	-1.18859	9.774806	-3.96914	0.001065	0.012358	-0.7576	Tnfaip8
12560	-1.18867	13.28008	-3.72957	0.001774	0.01537	-1.24297	Acad11
34497	-1.18876	10.86083	-3.65659	0.002072	0.016636	-1.39071	Gpank1
49641	-1.18882	13.83031	-4.24447	0.000595	0.009954	-0.20119	LOC675851
42641	-1.1889	9.757101	-4.09308	0.000819	0.011164	-0.50679	Zbtb40
10288	-1.18896	10.64771	-4.11947	0.000775	0.010871	-0.45346	Stard3
47408	-1.1892	12.72119	-4.534	0.000324	0.007686	0.379593	Rpl22l1
21492	-1.18937	10.0897	-4.6378	0.000261	0.007218	0.586279	Kif16b
1532	-1.18967	16.01514	-5.0981	0.000101	0.005481	1.489962	Acadvl
34933	-1.18994	10.33491	-4.28336	0.000548	0.009571	-0.12288	Cldn12
45606	-1.19007	13.95911	-4.74784	0.000208	0.006636	0.80432	Mrps21
22545	-1.19029	11.97905	-3.65515	0.002078	0.016675	-1.39363	Polr2k
372	-1.19034	13.30158	-4.39785	0.000431	0.008702	0.1072	Mrpl53
62928	-1.19066	8.751282	-4.31408	0.000514	0.009341	-0.06105	Nhsl1
21193	-1.19093	10.17866	-3.95363	0.001101	0.01252	-0.78901	Prpf18
39749	-1.19099	9.859871	-4.01099	0.000975	0.011974	-0.67286	Rab28
44678	-1.19103	9.043593	-4.4325	0.000401	0.008456	0.176641	Fbxo10
56132	-1.19111	9.336756	-4.70931	0.000225	0.006827	0.72809	Pex26
16350	-1.19143	8.416773	-4.49576	0.000351	0.007948	0.303224	Gtf2e1
44734	-1.19144	11.72215	-4.09941	0.000808	0.011101	-0.49399	Eif3f
54762	-1.19158	11.24122	-3.86817	0.00132	0.013502	-0.96214	Zfp260

50334	-1.19166	8.909154	-4.06575	0.000868	0.011414	-0.56207	Sec24d
45572	-1.19175	15.30358	-5.71035	2.99E-05	0.003707	2.652066	LOC100504871
44170	-1.19199	12.32403	-4.50116	0.000347	0.007904	0.314012	Tmem11
3654	-1.19222	9.710069	-4.82281	0.000178	0.006182	0.952179	Mrps2
14196	-1.1924	9.536499	-3.39557	0.003613	0.022245	-1.91742	Mex3c
15999	-1.19247	10.46306	-4.06505	0.000869	0.011417	-0.56347	Rcor3
2109	-1.19256	10.73024	-4.15162	0.000724	0.010601	-0.3885	Pex19
33397	-1.19277	7.359689	-2.97443	0.008803	0.037224	-2.75425	Gpm6a
47390	-1.19287	11.39364	-4.07172	0.000857	0.011351	-0.54999	Zcchc17
40356	-1.19294	7.326504	-3.77919	0.001596	0.014708	-1.14244	Clec18a
28929	-1.19299	11.46999	-3.7333	0.00176	0.01531	-1.23541	Cnot6l
617	-1.19326	9.49919	-3.83893	0.001405	0.013923	-1.02139	Zbtb5
57114	-1.19333	9.740429	-3.96655	0.001071	0.012374	-0.76284	H2afx
48784	-1.19333	12.50258	-4.3706	0.000456	0.008954	0.052516	Acot7
28365	-1.19397	8.918296	-4.41961	0.000412	0.008563	0.150819	Med20
7130	-1.19419	10.21037	-3.63531	0.002168	0.017052	-1.43378	Derl1
38288	-1.19423	12.29553	-3.81754	0.001471	0.014231	-1.06474	Rnf7
29906	-1.19435	15.55405	-4.81393	0.000181	0.006223	0.934702	Rpl14
29538	-1.19436	11.43963	-4.48472	0.000359	0.008021	0.281154	Mrpl40
12662	-1.19442	10.98043	-3.20205	0.005448	0.027933	-2.30466	Mlxipl
29692	-1.19448	9.986045	-3.4816	0.003008	0.02028	-1.74423	Rab3il1
6680	-1.19453	14.48796	-4.89767	0.000153	0.005922	1.099264	Anapc13
35392	-1.19462	8.78381	-4.8365	0.000173	0.006133	0.979127	Eif4e3
38248	-1.1948	9.298362	-3.85901	0.001346	0.013656	-0.9807	Zfp612
51056	-1.19524	11.21394	-4.38067	0.000447	0.008891	0.072722	Zswim7
12401	-1.19581	8.033606	-4.73814	0.000212	0.006665	0.785135	Ehhadh

48591	-1.19584	11.9888	-4.68752	0.000235	0.006899	0.684938	Kcnk3
20076	-1.1959	11.52714	-4.05399	0.00089	0.01149	-0.58586	Hsbp1
45646	-1.19593	13.1358	-4.61713	0.000272	0.007278	0.545189	Me3
37582	-1.19607	10.4123	-3.97795	0.001046	0.012301	-0.73976	Fbxl12
3764	-1.19636	10.63927	-4.18525	0.000674	0.010316	-0.32061	Slc27a1
26766	-1.19646	9.313212	-3.4436	0.003262	0.021061	-1.82079	Fgl2
9917	-1.19653	11.47308	-4.38344	0.000444	0.008871	0.078283	Map2k5
18648	-1.19661	11.86154	-3.80673	0.001505	0.014364	-1.08665	Csnk2a1
40523	-1.19683	10.93104	-3.89572	0.001245	0.013205	-0.90632	Cdk18
14920	-1.19687	9.861174	-3.91418	0.001197	0.013013	-0.86893	Trak1
43872	-1.19729	9.491039	-3.89862	0.001238	0.013202	-0.90046	Tmem111
48420	-1.19748	12.34913	-6.72599	4.41E-06	0.002346	4.459912	Asb2
5968	-1.19758	9.931832	-4.67364	0.000242	0.006971	0.657416	Matr3
30141	-1.19782	12.583	-3.80487	0.001511	0.014396	-1.09042	Cd164
7642	-1.1979	9.793817	-4.27706	0.000555	0.009599	-0.13556	AI314976
33652	-1.19805	10.37396	-4.55043	0.000313	0.007617	0.412365	Rpain
57833	-1.19816	10.229	-4.20965	0.00064	0.010117	-0.27139	Wdr82
26235	-1.19861	9.215944	-5.02652	0.000117	0.005645	1.350969	Calr3
29618	-1.19893	14.01915	-4.57152	0.0003	0.007478	0.454403	Timm23
12502	-1.19905	13.09749	-4.51499	0.000337	0.007877	0.341652	Higd1a
60448	-1.19939	13.60159	-5.09323	0.000102	0.005491	1.480533	Cmya5
49314	-1.19955	13.71977	-3.76551	0.001643	0.014895	-1.17016	Ran
20183	-1.19955	11.60166	-4.31583	0.000512	0.009337	-0.05754	Jtb
54487	-1.19956	10.45383	-5.244	7.55E-05	0.004894	1.77136	Abtb2
40603	-1.19956	11.58472	-4.22258	0.000623	0.0101	-0.24533	Sgpp1
61307	-1.19969	16.03233	-5.21927	7.94E-05	0.004967	1.72386	Etfb

20035	-1.19971	9.407489	-4.3698	0.000457	0.008954	0.050913	Zfp407
31605	-1.19987	8.878338	-3.44061	0.003282	0.021115	-1.82681	Mrvi1
9526	-1.20007	10.12484	-4.30348	0.000525	0.009438	-0.08238	Cav2
2137	-1.20014	10.75737	-3.74607	0.001712	0.015125	-1.20954	Metap1
21068	-1.20024	13.76683	-3.5157	0.002797	0.01956	-1.67547	Mapksp1
35081	-1.20027	10.5638	-4.94378	0.000139	0.005859	1.18954	Whrn
48547	-1.20069	12.52685	-5.11381	9.82E-05	0.005435	1.520384	Car14
56570	-1.20091	12.95958	-4.61425	0.000274	0.007296	0.539461	Trip10
43820	-1.20091	13.05885	-4.32318	0.000504	0.009325	-0.04276	Psmc2
41082	-1.20109	11.511	-3.96887	0.001066	0.012359	-0.75815	Bccip
30782	-1.20119	12.17335	-3.75814	0.001669	0.01499	-1.18509	Smpdl3a
23903	-1.20192	11.37332	-3.23004	0.005134	0.027049	-2.24889	Nacc2
35570	-1.20207	9.025091	-4.07825	0.000845	0.011283	-0.53678	Ap4s1
22916	-1.20222	10.89459	-3.68551	0.001948	0.016103	-1.33217	Nufip2
37336	-1.2026	11.02095	-4.14836	0.000729	0.010606	-0.3951	Dedd2
50608	-1.20263	10.25394	-3.65804	0.002066	0.016609	-1.38779	Zfp207
1526	-1.20271	12.62063	-5.23443	7.70E-05	0.004951	1.752986	Oxa1l
31183	-1.20275	10.80604	-3.72279	0.001799	0.015473	-1.25668	Syap1
10697	-1.2028	14.2825	-3.25871	0.004831	0.026053	-2.19168	Cpe
27350	-1.20288	8.726936	-4.65104	0.000254	0.007111	0.612565	Npc1
20296	-1.20299	9.567565	-4.06021	0.000878	0.011455	-0.57327	Timm9
50493	-1.20338	9.668092	-3.03694	0.00772	0.034366	-2.63156	Tmprss13
9697	-1.2034	11.07685	-3.72526	0.00179	0.015421	-1.25169	Rexo4
62338	-1.20343	13.24191	-3.91633	0.001192	0.013001	-0.86456	Prdx1
15349	-1.20362	8.806428	-3.54456	0.002631	0.018815	-1.61721	Cggbp1
54540	-1.2037	9.972492	-3.92027	0.001182	0.012921	-0.85658	Dido1

50862	-1.20387	9.815172	-4.45782	0.00038	0.008257	0.227351	Laptm4b
60731	-1.20437	9.815715	-4.13642	0.000747	0.010709	-0.4192	Seh1l
28379	-1.20439	10.25519	-3.82388	0.001451	0.01415	-1.0519	Lztfl1
35071	-1.20456	10.66159	-4.01204	0.000973	0.011974	-0.67075	Zfp191
26226	-1.20473	9.826754	-3.83372	0.001421	0.013969	-1.03195	Ssr2
26460	-1.20474	8.344197	-5.59168	3.78E-05	0.0039	2.430793	Frem2
42405	-1.20489	9.211943	-4.89211	0.000154	0.005923	1.088351	Fn3k
29132	-1.20502	11.56197	-4.74481	0.000209	0.00664	0.798334	Ednra
26861	-1.20541	9.620185	-4.58003	0.000294	0.007457	0.471351	Arl2
44353	-1.20566	12.76591	-4.54978	0.000313	0.007617	0.411067	Csnk2b
51457	-1.2057	12.44225	-3.7805	0.001591	0.014692	-1.13979	Agpat2
21070	-1.20573	11.74538	-4.16335	0.000706	0.010472	-0.36483	Dennd4b
42129	-1.20596	10.47866	-4.2031	0.000649	0.010138	-0.2846	Lrrc20
13480	-1.206	15.05279	-5.6441	3.41E-05	0.003805	2.528778	D10Jhu81e
14433	-1.20604	15.12089	-5.53801	4.20E-05	0.00398	2.330081	Ndufa7
62015	-1.20629	9.743243	-4.48668	0.000358	0.008021	0.285071	Sh3rf2
27272	-1.20655	9.845952	-4.03606	0.000924	0.011716	-0.62213	Cetn3
21727	-1.20667	12.21975	-3.74633	0.001711	0.015124	-1.20902	Slmo2
20424	-1.20682	13.36441	-4.06418	0.000871	0.011417	-0.56524	Ctbp1
48299	-1.20683	11.09616	-5.61101	3.64E-05	0.0039	2.466979	Anks1
3597	-1.20698	14.60064	-3.45346	0.003194	0.020825	-1.80095	Tomm5
8210	-1.20749	13.81012	-4.56476	0.000304	0.007532	0.440943	Ghitm
1496	-1.20769	12.93854	-4.36814	0.000458	0.008954	0.047581	Cops7a
16330	-1.20783	13.47113	-4.63319	0.000264	0.007219	0.577105	Trpc4ap
40720	-1.20785	8.496402	-5.72098	2.93E-05	0.003693	2.671785	Cacnb2
29630	-1.20805	12.11508	-3.4979	0.002906	0.019874	-1.71138	Nptn

30492	-1.20807	9.995363	-4.0698	0.000861	0.01137	-0.55386	Gm6616
9690	-1.20811	9.262722	-4.50971	0.000341	0.007904	0.331106	Pik3r4
53787	-1.20854	9.794639	-5.02931	0.000117	0.005645	1.356391	Hmbs
39321	-1.20879	11.17789	-3.79669	0.001537	0.014542	-1.10698	Smg6
57368	-1.20895	12.54062	-4.05719	0.000884	0.011479	-0.57939	Raf1
27701	-1.20905	12.06596	-3.94986	0.00111	0.012525	-0.79664	Slc20a2
57353	-1.20917	10.95541	-4.31625	0.000511	0.009337	-0.0567	Scrn2
27508	-1.20943	10.10705	-3.89361	0.001251	0.013205	-0.91059	Sec13
43702	-1.2096	9.227623	-4.39879	0.00043	0.008694	0.109074	Esrrg
48039	-1.20966	9.745275	-3.8075	0.001502	0.014362	-1.08507	Dnajb9
32505	-1.2098	12.71644	-4.18977	0.000668	0.010268	-0.31149	Ulk1
11468	-1.20985	8.977755	-3.97272	0.001057	0.012341	-0.75036	Tmem126b
59344	-1.21015	9.259878	-3.70749	0.001859	0.01565	-1.28768	Tcf21
27216	-1.21017	9.544127	-4.6694	0.000244	0.007001	0.649	Zfp672
50156	-1.21024	11.12667	-4.40327	0.000426	0.008651	0.118067	Lclat1
32914	-1.21038	9.098622	-4.12365	0.000768	0.010819	-0.44501	Nmb
12923	-1.21041	9.385372	-4.50354	0.000345	0.007904	0.318774	Slc39a3
24718	-1.21063	14.29221	-4.57482	0.000298	0.007471	0.460986	Alas1
59043	-1.21089	10.38185	-4.08613	0.000831	0.011247	-0.52085	Hcfc1
26360	-1.21097	8.790754	-4.2364	0.000605	0.010014	-0.21746	Gtf2h3
25478	-1.21102	14.53217	-5.0624	0.000109	0.005572	1.420725	Ndufs4
23246	-1.21117	10.56329	-4.15523	0.000718	0.010559	-0.38121	Ccrn4l
22308	-1.2112	11.67902	-3.95336	0.001102	0.01252	-0.78956	Pdgfa
44697	-1.21123	9.619174	-4.28658	0.000544	0.009554	-0.11638	Tnni3k
10774	-1.21158	10.61775	-3.89335	0.001252	0.013205	-0.91113	Yaf2
42700	-1.21169	12.1179	-4.14081	0.00074	0.010661	-0.41035	Zfand3

26447	-1.21216	13.23368	-4.50656	0.000343	0.007904	0.324807	C1qbp
17494	-1.21333	12.21365	-3.54916	0.002605	0.018756	-1.60793	Hnrpdl
16120	-1.21371	9.746747	-4.02775	0.000941	0.011752	-0.63894	Dapk1
17466	-1.21374	11.05451	-3.68159	0.001965	0.016156	-1.34012	Dhdh
40258	-1.21377	10.04084	-4.12677	0.000763	0.010783	-0.43871	Fundc1
27063	-1.21378	11.84587	-4.81186	0.000182	0.006231	0.930623	Timm17b
27263	-1.21386	10.12135	-5.02836	0.000117	0.005645	1.354541	Agap1
59723	-1.21394	10.74651	-4.18713	0.000671	0.01029	-0.31683	Clcc1
16985	-1.21429	8.940861	-3.9029	0.001226	0.013166	-0.89177	Eif2a
38970	-1.21467	7.513736	-5.15122	9.10E-05	0.005319	1.592717	Slc38a4
14549	-1.21469	8.735509	-4.1819	0.000679	0.010327	-0.32739	Hpdl
11819	-1.21486	11.00306	-3.81788	0.00147	0.014231	-1.06404	Rfwd3
9738	-1.21502	10.2278	-5.01387	0.00012	0.005671	1.326327	Irx4
18413	-1.21554	11.96331	-3.88663	0.00127	0.013275	-0.92475	Fam125b
50975	-1.21598	11.97991	-4.32457	0.000502	0.009325	-0.03996	Mrpl47
30908	-1.21605	12.20847	-4.01536	0.000966	0.01193	-0.66403	Slc25a13
42291	-1.21622	10.84167	-4.27736	0.000555	0.009599	-0.13495	Ezr
55742	-1.21662	10.85739	-4.43866	0.000395	0.008424	0.188983	Scnm1
5400	-1.21694	11.10411	-4.05121	0.000895	0.011496	-0.59148	Mrpl44
7209	-1.21733	13.19374	-4.01633	0.000964	0.011923	-0.66206	Htra1
28302	-1.21747	11.77734	-4.36866	0.000458	0.008954	0.048621	Qars
4383	-1.21751	14.13985	-4.46978	0.00037	0.008133	0.251286	Dnajc15
22798	-1.21759	11.17807	-5.21498	8.00E-05	0.004967	1.715606	Mrps9
6899	-1.21763	10.87088	-4.61642	0.000273	0.00728	0.543784	Cenpv
60885	-1.21796	11.25478	-3.78034	0.001592	0.014692	-1.1401	Golga7
22144	-1.21814	12.72385	-5.11771	9.74E-05	0.005435	1.527949	Phpt1

28859	-1.21824	10.48604	-3.74219	0.001727	0.015206	-1.2174	Las1l
34547	-1.21826	7.948455	-6.07062	1.49E-05	0.002865	3.311266	Adhfe1
23334	-1.21826	11.81654	-3.99674	0.001005	0.012068	-0.70173	Slc29a1
61438	-1.21862	7.9984	-4.78327	0.000193	0.006391	0.874276	Amph
13849	-1.21869	11.35123	-3.95995	0.001086	0.012477	-0.77621	Opa1
9190	-1.21878	11.66161	-4.6556	0.000252	0.007106	0.621627	Nudt19
42471	-1.219	13.00255	-4.17837	0.000684	0.010338	-0.3345	Glrx5
14551	-1.21914	12.11776	-4.221	0.000625	0.0101	-0.24851	Gm4535
7218	-1.21924	10.51682	-4.57929	0.000295	0.007459	0.46989	Ubiad1
20671	-1.21959	8.545656	-5.38972	5.64E-05	0.004397	2.049722	Fastkd1
26493	-1.21976	11.57896	-3.09785	0.00679	0.031887	-2.51141	Ammecr1l
10912	-1.21995	8.211941	-4.20634	0.000645	0.010117	-0.27807	Pigz
2799	-1.22007	9.073293	-3.78465	0.001577	0.014645	-1.13139	Bmper
21546	-1.22011	12.44515	-4.6991	0.00023	0.006871	0.707869	Hdac5
52063	-1.22037	9.582519	-5.49819	4.54E-05	0.004091	2.25509	Pvrl2
34666	-1.22051	13.02694	-3.8412	0.001398	0.013908	-1.01679	Txndc17
17787	-1.22064	8.24099	-5.16361	8.88E-05	0.005274	1.616641	Lysmd4
5421	-1.22071	10.32201	-2.93401	0.009581	0.039249	-2.83321	Bace2
253	-1.22108	8.561173	-4.13818	0.000745	0.010683	-0.41566	Mthfd2l
62588	-1.22135	10.4043	-4.69189	0.000233	0.006897	0.693591	Nap1l4
38363	-1.22142	11.1262	-3.51504	0.002801	0.019575	-1.6768	Itpa
5146	-1.22181	12.09667	-5.15018	9.12E-05	0.005319	1.590726	Rilp
15095	-1.22206	9.007806	-4.4429	0.000392	0.00837	0.197476	Slc19a2
25811	-1.2222	9.824271	-3.70318	0.001876	0.015731	-1.2964	Arid1a
37585	-1.22248	14.32597	-5.15105	9.11E-05	0.005319	1.592407	Phyh
18216	-1.22297	10.50836	-5.06004	0.00011	0.005572	1.416124	Pecr

9608	-1.22302	9.675833	-4.19737	0.000657	0.010204	-0.29617	Krr1
27259	-1.2233	14.07253	-4.39543	0.000433	0.00873	0.102348	Mrfap1
26515	-1.2235	8.546034	-4.65969	0.000249	0.007065	0.62974	Cox15
2051	-1.22364	10.36782	-4.85535	0.000166	0.006078	1.016182	Adck1
25610	-1.22367	15.5725	-5.85331	2.27E-05	0.003204	2.915937	Cyc1
21655	-1.22388	12.89913	-4.59722	0.000284	0.007333	0.505589	Nudt9
32366	-1.22389	12.7563	-6.43824	7.48E-06	0.002382	3.963754	Adck3
59903	-1.22399	10.2685	-4.70604	0.000227	0.006827	0.721622	Acvr2b
61111	-1.22421	10.31041	-4.84891	0.000169	0.006115	1.003535	Cct3
53455	-1.22441	11.23352	-3.9273	0.001164	0.012845	-0.84235	Gata6
14917	-1.22472	10.36151	-4.88058	0.000158	0.005925	1.065736	Ndufaf3
53673	-1.22516	10.83755	-3.93125	0.001155	0.012818	-0.83435	Zfp943
12245	-1.22524	12.06624	-3.7161	0.001825	0.015558	-1.27025	Txndc15
56424	-1.2254	10.81247	-3.98048	0.00104	0.012268	-0.73465	Cdc14b
14085	-1.2255	12.44662	-2.90788	0.01012	0.040653	-2.88411	Slc38a2
27582	-1.22551	14.42231	-4.89832	0.000152	0.005922	1.100528	Rps15a
4587	-1.22572	12.55341	-4.31215	0.000516	0.009357	-0.06494	Dlat
46264	-1.22576	11.15328	-4.35788	0.000468	0.009046	0.026962	Ublcp1
16633	-1.22577	12.55118	-4.03466	0.000927	0.011716	-0.62497	Clasp1
40259	-1.22637	11.78968	-4.32346	0.000504	0.009325	-0.04219	Mff
58319	-1.22643	11.21706	-3.83729	0.00141	0.013936	-1.02472	Khdrbs3
33145	-1.22647	12.39219	-3.77282	0.001618	0.014799	-1.15535	Tardbp
10802	-1.22709	11.25568	-4.18047	0.000681	0.010327	-0.33026	Gtpbp5
58619	-1.22746	10.58169	-3.75233	0.00169	0.015038	-1.19685	Ing4
27025	-1.2285	8.728327	-4.88308	0.000157	0.005925	1.070639	Mett5d1
35924	-1.22862	12.4028	-4.63236	0.000264	0.007219	0.575468	Deb1

28092	-1.22885	8.561885	-4.23852	0.000602	0.009996	-0.21318	Zfp82
62671	-1.22895	15.77381	-6.07583	1.48E-05	0.002865	3.320662	Cycs
55997	-1.22898	11.95896	-3.8041	0.001513	0.014414	-1.09198	Pgs1
57617	-1.2291	9.701585	-3.80873	0.001499	0.014345	-1.08258	Dnajc24
50333	-1.22985	7.977195	-3.67699	0.001984	0.01626	-1.34943	Gprasp2
7829	-1.23004	8.827308	-4.48341	0.00036	0.008021	0.278537	Zfp30
13774	-1.23009	10.06962	-4.25552	0.000581	0.009799	-0.17894	Mcart1
48423	-1.2302	11.77426	-4.31344	0.000514	0.009346	-0.06234	Rfwd2
11331	-1.23039	9.388392	-4.0645	0.00087	0.011417	-0.56459	Xpo1
56688	-1.23049	9.907091	-3.95283	0.001103	0.01252	-0.79064	Med30
22082	-1.2306	9.099645	-4.41839	0.000413	0.008563	0.148385	Rdh5
25323	-1.23073	12.30726	-4.59882	0.000283	0.007333	0.508763	Mat2b
7656	-1.23085	10.05939	-3.47682	0.003039	0.020389	-1.75387	Cdc42ep3
47890	-1.23151	9.810174	-4.81897	0.000179	0.006189	0.94462	Lsm14b
57664	-1.23151	12.96021	-4.16232	0.000707	0.010472	-0.3669	Cxx1c
14640	-1.23155	11.26888	-3.87372	0.001305	0.013449	-0.95089	Thbd
9747	-1.2316	11.23947	-4.12082	0.000772	0.010864	-0.45074	Pigo
28611	-1.23203	10.29792	-4.1723	0.000693	0.010378	-0.34675	Rreb1
41672	-1.23214	12.6419	-3.34941	0.003985	0.023383	-2.01011	Lamb1
33294	-1.23221	10.18053	-4.75069	0.000207	0.006636	0.80995	Med31
59266	-1.2327	10.8312	-3.39299	0.003632	0.022318	-1.92261	Trappc6b
29745	-1.23272	12.35213	-4.03149	0.000933	0.011731	-0.63139	Mlf2
36862	-1.23289	10.87359	-4.5126	0.000339	0.007886	0.336876	Slc25a19
24320	-1.23386	11.70612	-4.06791	0.000864	0.011395	-0.55769	Syvn1
19541	-1.23409	11.26572	-4.21234	0.000637	0.0101	-0.26596	Rgs3
13558	-1.23439	11.31012	-3.97453	0.001053	0.012334	-0.74668	Pgam5

41395	-1.23444	9.86904	-3.99695	0.001004	0.012068	-0.70129	Golph3l
21332	-1.23506	8.134153	-4.99288	0.000126	0.005707	1.285435	Plxnb1
31837	-1.23514	10.56155	-3.89868	0.001238	0.013202	-0.90033	Adnp
26833	-1.23528	11.64383	-4.40513	0.000424	0.008651	0.121798	Ccdc58
60196	-1.23545	10.71419	-4.90092	0.000152	0.005922	1.105639	Tmem14c
19633	-1.23571	9.685445	-4.0351	0.000926	0.011716	-0.62408	Plekhb2
51368	-1.23589	9.555357	-4.99702	0.000125	0.005707	1.293502	Ric8b
29614	-1.23591	11.54425	-3.75389	0.001684	0.015038	-1.1937	Vezf1
38676	-1.23593	8.683295	-4.35266	0.000474	0.009093	0.016486	Acrbp
25272	-1.23602	10.78305	-5.4674	4.83E-05	0.004208	2.196954	Get4
56485	-1.23622	12.14288	-3.81732	0.001471	0.014231	-1.06519	Ssr3
45564	-1.23661	12.45469	-5.06579	0.000108	0.005562	1.42729	H2-Ke6
60886	-1.23671	12.84573	-4.32351	0.000504	0.009325	-0.0421	Mpp6
56153	-1.23704	10.20745	-4.34011	0.000486	0.009137	-0.00872	Cdkal1
62443	-1.23719	11.71893	-4.31994	0.000507	0.009336	-0.04928	Naprt1
2894	-1.23749	9.626113	-4.82036	0.000179	0.006189	0.947366	Dusp28
4520	-1.23786	8.112787	-4.64011	0.00026	0.007218	0.590863	Dcaf12l1
41671	-1.238	8.567623	-4.13367	0.000752	0.010741	-0.42476	Asb4
7008	-1.23805	11.68786	-4.7134	0.000223	0.006803	0.736192	Nudt8
10495	-1.2383	10.53843	-3.51043	0.002829	0.019655	-1.68609	Epdr1
57714	-1.23837	11.46825	-4.75162	0.000206	0.006636	0.811779	Sirt5
31235	-1.2386	15.65057	-6.17526	1.22E-05	0.002798	3.499096	Sod2
13529	-1.23871	11.31231	-3.8455	0.001386	0.013862	-1.00809	Alkbh5
47235	-1.23925	11.79447	-4.20817	0.000642	0.010117	-0.27438	Strn3
34372	-1.23929	10.83939	-4.0932	0.000819	0.011164	-0.50656	Gba2
36850	-1.23951	10.71145	-4.43082	0.000402	0.00847	0.173274	Klhl21

33937	-1.23952	14.0593	-4.19027	0.000667	0.010268	-0.31048	B230118H07Rik
18257	-1.24005	9.230231	-4.31128	0.000517	0.009357	-0.06669	Pppde1
19555	-1.24019	11.71542	-3.87939	0.001289	0.013363	-0.93941	Tmbim4
230	-1.24059	9.320516	-4.90715	0.00015	0.005914	1.117842	Pknox2
49988	-1.24106	9.491673	-4.88688	0.000156	0.005925	1.078108	Inpp5e
22753	-1.24142	11.83243	-3.97068	0.001062	0.012344	-0.75447	LOC100039181
11866	-1.24154	11.58166	-4.05121	0.000895	0.011496	-0.59147	Tnk2
5710	-1.24188	9.371504	-3.84686	0.001382	0.013848	-1.00534	Cse1l
3356	-1.24199	9.338814	-3.92948	0.001159	0.012818	-0.83794	Slu7
38545	-1.24221	8.4587	-4.55393	0.000311	0.007593	0.419351	Zfp84
36433	-1.24254	8.99623	-4.72757	0.000217	0.006719	0.764242	Dok2
8497	-1.24255	10.57861	-3.7471	0.001709	0.015117	-1.20744	Tjp1
54728	-1.24257	11.03748	-5.0307	0.000116	0.005645	1.359091	Trpt1
6816	-1.24261	11.66986	-3.95582	0.001096	0.012502	-0.78457	Ank2
53280	-1.24265	12.5892	-4.5719	0.000299	0.007478	0.455157	Dctn6
62570	-1.24299	8.914672	-4.3752	0.000452	0.008944	0.061748	Trim13
41829	-1.24306	12.51891	-5.01842	0.000119	0.005671	1.335195	Lrpprc
25291	-1.24328	13.12368	-3.98353	0.001033	0.012238	-0.72847	Gtf2i
15911	-1.24338	8.857303	-4.90788	0.000149	0.005914	1.119269	Ccdc64
5091	-1.24374	11.10124	-4.76066	0.000202	0.006587	0.829646	Plcd1
30891	-1.2439	9.314821	-4.52975	0.000327	0.007724	0.371116	Hiat1
13192	-1.24408	9.854107	-4.13268	0.000753	0.010744	-0.42676	Rb1cc1
23124	-1.24417	9.552189	-3.04413	0.007604	0.03406	-2.61739	Scrn1
39192	-1.24426	10.36837	-4.71332	0.000223	0.006803	0.736035	Mrpl46
23401	-1.24428	10.00935	-3.98923	0.001021	0.012136	-0.71692	Smad3
33467	-1.24457	11.3209	-3.99545	0.001008	0.012068	-0.70434	Chtf8

11062	-1.24463	11.26161	-3.94265	0.001127	0.012635	-0.81124	Gstm5
7026	-1.24482	10.64222	-4.2624	0.000573	0.009725	-0.16506	Osbpl1a
41241	-1.24485	11.09695	-3.81275	0.001486	0.014299	-1.07445	Eya3
21522	-1.24501	12.08829	-4.5975	0.000284	0.007333	0.506138	Mrpl27
33807	-1.24544	12.64454	-4.94445	0.000139	0.005859	1.190864	Tmed1
6806	-1.24559	11.29436	-3.4907	0.00295	0.020009	-1.72589	Lamp3
60143	-1.24589	11.29316	-3.61368	0.00227	0.017417	-1.47752	Yipf4
32752	-1.24618	9.856606	-4.86249	0.000164	0.006056	1.030212	Hars2
35192	-1.24622	13.14499	-5.08016	0.000105	0.005504	1.455195	Fam165b
52413	-1.24626	11.06394	-3.75185	0.001691	0.015038	-1.19783	Acvr1
36059	-1.24645	8.360229	-4.2247	0.00062	0.010087	-0.24104	Slc22a23
51004	-1.24657	11.82679	-3.71884	0.001815	0.015538	-1.26469	St13
16486	-1.24665	14.48181	-4.1711	0.000694	0.010391	-0.34917	Rpl23a
51020	-1.24711	11.31135	-4.51458	0.000337	0.007877	0.340825	Hist1h2bm
56324	-1.24712	9.664511	-3.85286	0.001364	0.013751	-0.99317	Cops8
29937	-1.24731	10.51917	-3.85322	0.001363	0.013751	-0.99245	Smc3
9285	-1.24798	10.28136	-4.43799	0.000396	0.008424	0.187649	Art5
5138	-1.24805	10.98337	-5.04648	0.000113	0.005602	1.389787	Ttc35
37256	-1.2487	10.79043	-4.48264	0.000361	0.008021	0.277	Ppapdc3
10709	-1.24882	9.017724	-4.41885	0.000412	0.008563	0.149296	Ogt
16132	-1.24889	12.67243	-4.39395	0.000434	0.008749	0.099373	Sra1
5749	-1.24925	11.57609	-4.32491	0.000502	0.009325	-0.03929	Sik3
12402	-1.24931	7.458763	-4.73469	0.000214	0.006665	0.77831	Gpr22
16170	-1.24939	10.53587	-3.69181	0.001922	0.015958	-1.31944	LOC100504289
22469	-1.24943	11.10259	-4.60821	0.000278	0.007325	0.527453	Cox11
3978	-1.24952	8.342658	-4.63168	0.000264	0.007219	0.574109	Plekhb1

58288	-1.24997	10.99439	-4.61296	0.000275	0.007306	0.536903	Rnf166
337	-1.25028	11.81095	-4.71366	0.000223	0.006803	0.7367	Grb14
43979	-1.25061	10.32513	-3.97127	0.001061	0.012341	-0.75328	Rchy1
51597	-1.25103	10.41415	-4.55737	0.000309	0.007581	0.42621	Mmachc
33492	-1.25112	12.34383	-4.73529	0.000213	0.006665	0.779504	Phtf2
24960	-1.25128	9.060453	-4.8882	0.000156	0.005925	1.080694	Zdhhc13
27342	-1.25155	11.28636	-4.4852	0.000359	0.008021	0.282124	Gm5617
11219	-1.25175	9.675071	-3.92781	0.001163	0.012845	-0.84131	Ubfd1
37478	-1.25192	12.60828	-3.95851	0.00109	0.012479	-0.77913	Fundc2
40531	-1.25236	11.29767	-4.80144	0.000186	0.006267	0.91009	Tcp11l2
15602	-1.25252	12.13558	-5.05493	0.000111	0.005572	1.406198	Pomgnt1
24440	-1.25258	8.390294	-5.2365	7.66E-05	0.004951	1.756973	Gab1
19799	-1.25319	8.888651	-4.95899	0.000135	0.005828	1.219279	Kcnip3
1203	-1.25327	12.11114	-4.34194	0.000484	0.009132	-0.00506	Ssbp1
21030	-1.25337	11.99504	-4.04447	0.000908	0.011594	-0.60512	Ptgr2
11694	-1.25351	9.167662	-3.46494	0.003117	0.020606	-1.77781	Cyp4b1
29397	-1.25353	12.35566	-5.03521	0.000115	0.005645	1.367865	Mrpl22
8100	-1.25357	9.562758	-4.59116	0.000288	0.007378	0.493526	Capn7
19613	-1.25379	12.5574	-3.79118	0.001556	0.014594	-1.11814	Xbp1
22797	-1.25453	8.390465	-4.40325	0.000426	0.008651	0.118027	Rasl10a
57111	-1.25461	8.701208	-5.01687	0.00012	0.005671	1.332183	Fbxl5
24897	-1.25463	10.47007	-3.99917	0.001	0.012047	-0.6968	Smyd1
47232	-1.25508	8.843298	-4.32774	0.000499	0.009314	-0.0336	C430048L16Rik
24737	-1.25522	13.64736	-4.62881	0.000266	0.007229	0.568414	Gm6238
41294	-1.25532	9.588675	-4.76358	0.000201	0.006567	0.835409	Mettl5
18881	-1.25533	9.64914	-3.9779	0.001046	0.012301	-0.73986	Vbp1

12872	-1.25546	11.60704	-3.86767	0.001322	0.013506	-0.96316	Hmgn2
33247	-1.25549	9.727635	-3.51203	0.002819	0.019626	-1.68287	Gm3014
60427	-1.25565	11.56116	-3.90591	0.001219	0.01314	-0.88567	Med8
17802	-1.25567	11.67895	-3.5977	0.002349	0.017719	-1.50983	Tmco1
16060	-1.25611	7.788371	-4.17461	0.000689	0.010365	-0.34209	Ddc
49478	-1.25632	11.22423	-3.97327	0.001056	0.012341	-0.74923	Abl1
17071	-1.25633	9.232458	-5.19297	8.37E-05	0.005108	1.673243	Psmb2
49740	-1.25645	10.98082	-4.25436	0.000583	0.009815	-0.18127	Dgcr2
20860	-1.25667	8.616793	-4.30378	0.000525	0.009438	-0.08179	Ttc21b
4081	-1.25682	14.57044	-4.10545	0.000798	0.011061	-0.4818	Gng5
60876	-1.25708	12.33945	-4.05926	0.00088	0.011455	-0.57519	Gm10094
48075	-1.2571	11.80169	-4.86346	0.000164	0.006054	1.032117	Gm12693
30810	-1.25725	12.8939	-3.96548	0.001074	0.012389	-0.76501	Dnaja3
5264	-1.25778	11.81011	-4.86595	0.000163	0.006036	1.037012	Rhot1
15877	-1.25782	11.31608	-4.26074	0.000575	0.00974	-0.16842	LOC100134990
50307	-1.25793	11.28451	-4.28532	0.000546	0.009554	-0.11892	Adipor1
48086	-1.2581	13.91626	-6.1603	1.26E-05	0.002798	3.472353	Dlst
36454	-1.2587	12.05794	-4.09391	0.000818	0.011164	-0.50512	Psmb1
44067	-1.25875	12.88354	-4.19354	0.000662	0.010247	-0.3039	Mrpl3
51876	-1.25878	8.743366	-4.79878	0.000187	0.006267	0.904847	Dut
23437	-1.25923	12.46326	-3.99159	0.001016	0.012109	-0.71214	Josd1
15958	-1.26009	12.30219	-3.98199	0.001037	0.012257	-0.73159	Ak3
52652	-1.26009	15.08143	-3.12458	0.006417	0.0308	-2.4585	Hbb-b1
31880	-1.26024	10.75503	-4.50488	0.000344	0.007904	0.321454	Fam118b
44343	-1.26092	12.31054	-5.07674	0.000106	0.005504	1.448556	Hint3
14172	-1.26141	14.2852	-4.63926	0.00026	0.007218	0.589175	Sdha

6673	-1.26155	12.50005	-3.75127	0.001694	0.015044	-1.199	Cct2
3020	-1.26197	10.1576	-4.55803	0.000308	0.007581	0.427526	LOC100503238
56531	-1.26201	12.43388	-4.37671	0.00045	0.008933	0.064767	Echdc2
31479	-1.26225	11.8289	-3.61865	0.002246	0.017305	-1.46746	Tfpi
31311	-1.26228	12.58572	-4.29625	0.000533	0.009487	-0.09692	Tmem85
59996	-1.26316	12.09094	-3.65659	0.002072	0.016636	-1.39073	Ptplad1
4598	-1.26352	11.64903	-3.77766	0.001601	0.014716	-1.14555	Prr14
28967	-1.26361	11.50769	-4.14634	0.000732	0.010607	-0.39917	Pcnp
60027	-1.26446	13.97061	-6.34613	8.88E-06	0.002463	3.802227	Bckdha
61104	-1.26464	10.14952	-4.63538	0.000262	0.007219	0.581458	Mpped2
49210	-1.26477	9.604066	-4.73156	0.000215	0.006689	0.772119	Lsm1
55798	-1.26499	12.63272	-4.01311	0.00097	0.011955	-0.66858	Ogn
59934	-1.26502	10.34684	-4.65306	0.000253	0.007106	0.616583	Zfp68
58302	-1.26504	10.59209	-4.31158	0.000516	0.009357	-0.06609	Coq5
52252	-1.26512	9.703424	-3.98039	0.00104	0.012268	-0.73482	Actr6
4712	-1.26547	12.55751	-4.26054	0.000575	0.00974	-0.16881	Hsd17b4
14943	-1.26567	10.44011	-4.03923	0.000918	0.011676	-0.61571	Stmn1
9649	-1.26587	11.34979	-4.17604	0.000687	0.010365	-0.33921	Gm10731
61534	-1.26626	12.38163	-5.39397	5.59E-05	0.004397	2.057786	Snapin
9061	-1.26649	11.67269	-4.79916	0.000187	0.006267	0.905595	Sgcb
60445	-1.26673	10.45283	-3.87023	0.001315	0.013488	-0.95798	Cdk5rap1
50615	-1.26675	11.66382	-4.25007	0.000588	0.009882	-0.18991	Npepps
33619	-1.26683	12.60112	-4.35673	0.00047	0.009047	0.024664	Rbm5
26490	-1.26683	12.01038	-3.78626	0.001572	0.014645	-1.12811	Hax1
31704	-1.26789	12.38294	-4.79783	0.000187	0.006267	0.902983	Wnk2
11095	-1.2679	8.969765	-4.48468	0.000359	0.008021	0.281076	Pigv

31989	-1.2679	9.42029	-4.20844	0.000642	0.010117	-0.27384	Lgi4
28906	-1.26794	11.27695	-4.31205	0.000516	0.009357	-0.06515	Eif2s1
38866	-1.26807	11.35702	-3.71352	0.001835	0.015584	-1.27547	Ncoa4
29263	-1.26819	14.28472	-4.9919	0.000126	0.005707	1.283525	Gm5451
25040	-1.26836	8.812493	-4.84566	0.00017	0.006133	0.997142	D2hgdh
14451	-1.26872	11.46213	-4.39976	0.000429	0.008694	0.111014	Cct4
47946	-1.26895	13.274	-5.08506	0.000104	0.005504	1.464684	Chchd7
54180	-1.26908	12.47538	-4.07579	0.00085	0.011306	-0.54175	Ubac1
59162	-1.26913	10.21797	-4.00074	0.000996	0.012046	-0.69362	Banf1
1719	-1.26941	11.94555	-4.32494	0.000502	0.009325	-0.03922	Ndufaf4
51602	-1.26942	9.07863	-4.78859	0.000191	0.006332	0.884756	Capn3
46620	-1.26969	10.01337	-5.07464	0.000106	0.005514	1.444477	AI316807
39131	-1.2699	9.407345	-3.88119	0.001284	0.013363	-0.93576	Gprasp1
24179	-1.27	11.25502	-4.83331	0.000174	0.006133	0.972857	Ipo13
22568	-1.27015	9.815766	-4.10047	0.000806	0.011101	-0.49186	Tmem41a
52478	-1.27048	11.81711	-3.60117	0.002332	0.017655	-1.50283	Sh3bp5l
12080	-1.2708	11.41329	-4.36421	0.000462	0.008977	0.03968	Cacna2d1
952	-1.2709	9.118648	-4.61964	0.000271	0.007258	0.55019	Kifc2
32520	-1.27106	10.64882	-5.49172	4.60E-05	0.004091	2.242882	Pitrm1
11477	-1.27112	12.76443	-3.75319	0.001687	0.015038	-1.19512	Cnbp
51570	-1.27152	12.48597	-4.80383	0.000185	0.006267	0.914803	Cpeb3
57071	-1.27158	11.07125	-4.29108	0.000539	0.009544	-0.10733	Gm6578
43162	-1.27204	11.81126	-4.6081	0.000278	0.007325	0.527234	Osgep
36897	-1.27241	11.19566	-4.31894	0.000508	0.009336	-0.05128	Zfp637
28040	-1.27241	11.08021	-3.31296	0.004306	0.024473	-2.08317	Hsph1
27689	-1.27248	12.54683	-4.26415	0.000571	0.009719	-0.16154	Nfyc

10861	-1.27266	11.42177	-4.53171	0.000326	0.007714	0.375028	Taf6
44794	-1.27327	8.349893	-5.53713	4.21E-05	0.00398	2.328419	Ccdc46
34158	-1.27379	9.282167	-3.93709	0.00114	0.012753	-0.82252	Pde6d
18813	-1.27412	10.06137	-4.5805	0.000294	0.007457	0.472305	Yars2
13297	-1.27421	8.801099	-5.59834	3.73E-05	0.0039	2.443265	Lphn1
19574	-1.27457	11.00393	-3.75198	0.001691	0.015038	-1.19757	Ccdc53
54144	-1.27495	11.63924	-4.04308	0.000911	0.011608	-0.60793	Btg3
58983	-1.27551	10.47277	-4.53778	0.000321	0.007678	0.38714	Tmem60
16283	-1.27592	13.6024	-4.76645	0.0002	0.006541	0.841064	Vldlr
62516	-1.27608	10.73366	-4.37406	0.000453	0.008944	0.059465	Klhdc2
32010	-1.2763	11.0151	-3.70078	0.001886	0.015769	-1.30126	Abat
30131	-1.27666	9.376502	-4.1503	0.000726	0.010601	-0.39118	Rem1
31170	-1.27668	11.18904	-3.79192	0.001553	0.01459	-1.11664	Pygo2
48872	-1.27706	11.35241	-3.68111	0.001967	0.01616	-1.34108	Srp9
23165	-1.27738	12.02886	-4.31777	0.00051	0.009337	-0.05365	Fam73b
56516	-1.27827	11.93727	-3.97623	0.00105	0.012323	-0.74325	Rnaseh2c
37119	-1.27842	13.70379	-4.80099	0.000186	0.006267	0.909208	Grcc10
21164	-1.27843	11.6731	-4.15193	0.000723	0.010601	-0.38787	Lrrfip1
10237	-1.27852	12.18536	-4.0053	0.000987	0.01201	-0.68438	Asph
42508	-1.27872	11.90064	-4.31856	0.000509	0.009336	-0.05206	Nt5c3
47636	-1.27882	11.33892	-4.99639	0.000125	0.005707	1.292276	Gchfr
41910	-1.27882	10.00369	-4.238	0.000603	0.009999	-0.21424	Rarb
59312	-1.27916	9.124584	-4.41527	0.000415	0.008578	0.142115	Lypla1
21971	-1.2796	11.2837	-3.66924	0.002017	0.016395	-1.36512	Siva1
62085	-1.27963	10.77649	-3.0965	0.006809	0.031943	-2.51408	Fam102a
59667	-1.28005	9.923839	-4.62898	0.000266	0.007229	0.568747	Gm826

54533	-1.28013	10.30666	-4.10395	0.0008	0.011075	-0.48483	Ubl3
2870	-1.28045	11.96463	-4.09928	0.000808	0.011101	-0.49426	Eif5a
59938	-1.28095	12.89326	-4.4076	0.000422	0.008651	0.126744	Stard7
26108	-1.28115	10.30175	-4.52233	0.000332	0.007808	0.356302	Lactb
42963	-1.28121	9.914723	-3.85989	0.001344	0.013644	-0.97892	Rabggtb
39973	-1.28123	11.86197	-4.2156	0.000632	0.0101	-0.2594	Rilpl1
50827	-1.28127	12.7549	-3.82512	0.001447	0.014131	-1.04939	Phldb1
59195	-1.28137	14.07748	-4.6937	0.000232	0.006885	0.697171	Suclg1
57808	-1.28148	10.21287	-3.98424	0.001032	0.012226	-0.72702	Meaf6
26421	-1.28151	10.43555	-3.8123	0.001487	0.0143	-1.07536	Rarg
45203	-1.28236	10.60495	-4.37396	0.000453	0.008944	0.05926	Zfp187
50438	-1.28287	12.10332	-4.12597	0.000764	0.010788	-0.44032	Dctpp1
38460	-1.28346	12.78495	-3.76315	0.001651	0.014908	-1.17494	Pkdcc
51269	-1.2839	11.30255	-4.60308	0.000281	0.007327	0.517252	Flcn
32551	-1.28398	11.24539	-4.34651	0.00048	0.009113	0.004135	Eif4g2
3260	-1.28423	10.46561	-4.13004	0.000757	0.010755	-0.4321	Pxmp3
46265	-1.28444	10.02436	-3.34816	0.003996	0.023403	-2.01261	Enpp2
42073	-1.28454	11.72042	-4.66748	0.000245	0.007001	0.645196	Atxn2
61545	-1.28458	14.31294	-4.57744	0.000296	0.00747	0.466201	Timm8b
58390	-1.28521	11.86273	-4.62031	0.000271	0.007258	0.551504	Cox16
20659	-1.28563	12.12634	-4.17359	0.000691	0.010372	-0.34416	Thra
52255	-1.28576	8.577205	-5.29361	6.83E-05	0.004788	1.866448	Dcaf4
20461	-1.28587	15.10014	-5.40634	5.45E-05	0.004397	2.08129	Ndufs1
28746	-1.2859	8.685663	-3.54763	0.002614	0.018768	-1.61101	D0H4S114
50750	-1.28613	11.8328	-4.06017	0.000878	0.011455	-0.57336	Psmg1
49142	-1.28616	10.93105	-5.12782	9.55E-05	0.005402	1.547495	Scn4b

48842	-1.28628	12.34209	-5.05241	0.000111	0.005572	1.401311	Hmgcl
54520	-1.28641	12.10346	-3.95148	0.001106	0.012525	-0.79336	Atp5sl
4815	-1.28652	10.0648	-4.91914	0.000146	0.005905	1.14133	Mrps22
22749	-1.28669	11.71398	-4.55467	0.00031	0.007593	0.420834	Cobll1
56642	-1.28675	13.66692	-4.43273	0.0004	0.008456	0.177114	Mtch2
48649	-1.28687	13.8677	-5.06416	0.000109	0.005567	1.424136	Pdhb
56161	-1.28693	11.99544	-3.97782	0.001046	0.012301	-0.74003	Mettl9
23581	-1.28704	11.41282	-4.11725	0.000778	0.010901	-0.45795	Zadh2
39587	-1.28775	12.261	-4.37771	0.000449	0.00893	0.066779	Atp1a2
15882	-1.28807	14.1796	-4.7815	0.000194	0.006401	0.870781	Atp5f1
14425	-1.28837	12.58354	-4.00019	0.000997	0.012046	-0.69474	Hist1h2bc
61770	-1.28843	9.673272	-4.57016	0.0003	0.00749	0.451699	Mettl3
58030	-1.28856	11.69734	-4.61846	0.000272	0.007267	0.547834	N6amt2
51683	-1.28865	8.852743	-6.6434	5.13E-06	0.002346	4.318828	Wfdc1
4378	-1.28909	9.899919	-4.06511	0.000869	0.011417	-0.56337	Bmpr1a
2668	-1.28914	10.89441	-5.39512	5.58E-05	0.004397	2.059976	Pdf
34857	-1.28974	12.8712	-3.9589	0.001089	0.012479	-0.77834	Cyb5
1215	-1.28978	10.89371	-4.38437	0.000443	0.008862	0.080142	Kcnq1
42461	-1.2899	12.05526	-4.1229	0.000769	0.01083	-0.44653	Pigyl
59359	-1.29012	8.525561	-5.26917	7.18E-05	0.004837	1.819646	Rhbdl3
19594	-1.29055	11.14463	-4.34671	0.00048	0.009113	0.004527	Crybg3
17864	-1.29059	11.65213	-4.83303	0.000174	0.006133	0.972297	Endog
47802	-1.29063	11.88206	-3.3582	0.003911	0.023119	-1.99247	Rgs2
28373	-1.29073	10.60231	-4.20641	0.000645	0.010117	-0.27793	Cstf2t
35261	-1.29084	10.07589	-4.88029	0.000158	0.005925	1.065167	Arfgef1
10236	-1.29101	10.39434	-4.85515	0.000167	0.006078	1.015802	Cog6

60978	-1.29167	8.538095	-3.04917	0.007523	0.033915	-2.60748	Traf4
38085	-1.29179	11.42721	-4.40848	0.000421	0.008645	0.128517	Tcp1
22727	-1.29192	10.61821	-4.77118	0.000198	0.006513	0.850413	Ablim1
21292	-1.29206	11.6122	-4.83307	0.000174	0.006133	0.972375	Clpx
28525	-1.2924	11.69966	-4.9026	0.000151	0.005914	1.108914	Cd59a
31743	-1.29251	8.621162	-4.58971	0.000288	0.007384	0.49063	Bola1
29617	-1.29335	12.12729	-4.75883	0.000203	0.006602	0.826034	Itgb1bp2
26416	-1.2937	11.01681	-5.70519	3.02E-05	0.003713	2.642489	Cbr1
56252	-1.29397	8.717842	-4.61542	0.000273	0.007287	0.541788	Syde2
38167	-1.29415	12.23428	-4.34631	0.00048	0.009113	0.003735	Hist2h2aa1
4333	-1.29455	12.65316	-4.21367	0.000635	0.0101	-0.26328	Ddt
6948	-1.29486	13.48797	-4.10625	0.000797	0.011056	-0.48018	Phyhd1
33506	-1.29503	12.42751	-4.80301	0.000185	0.006267	0.913196	Rmnd1
47541	-1.29505	10.71936	-3.78677	0.00157	0.014645	-1.12709	Fam174a
32474	-1.29538	12.87218	-4.67846	0.00024	0.006956	0.66697	Fblim1
61274	-1.29541	10.12569	-4.02879	0.000939	0.011752	-0.63684	Rbm14
59629	-1.29569	9.571521	-4.14367	0.000736	0.010629	-0.40456	Sacm1l
33516	-1.2958	12.33261	-4.68329	0.000237	0.006925	0.676547	Fam158a
59064	-1.29591	10.3742	-4.89162	0.000155	0.005923	1.087397	Gm8120
7746	-1.29596	8.63976	-4.6825	0.000238	0.006925	0.67499	Hist2h3c2-ps
34817	-1.29602	11.86702	-4.70261	0.000228	0.006851	0.714821	Art3
61928	-1.29629	9.140441	-5.44473	5.05E-05	0.004235	2.154069	Abhd8
44526	-1.29655	8.478952	-4.39232	0.000436	0.008771	0.096108	Plp1
19445	-1.29665	13.59653	-4.17143	0.000694	0.010391	-0.3485	D4Wsu53e
14477	-1.29727	10.1408	-4.09376	0.000818	0.011164	-0.50542	Ppil1
53128	-1.29764	8.863183	-4.70597	0.000227	0.006827	0.721475	Mboat2

7839	-1.29804	12.25162	-4.96673	0.000132	0.005817	1.234403	Mrps24
62916	-1.2981	11.77345	-4.90408	0.000151	0.005914	1.111833	Grsf1
8722	-1.29831	12.15267	-4.27671	0.000556	0.009599	-0.13626	Arfrp1
17063	-1.29935	9.185093	-4.69969	0.00023	0.006871	0.709044	Zdhhc17
31218	-1.29938	13.47378	-4.51325	0.000338	0.007886	0.338167	Sumo2
23648	-1.29941	10.09518	-6.42353	7.69E-06	0.002382	3.938045	Pex11b
17505	-1.3	11.72795	-3.95703	0.001093	0.012483	-0.78212	Lman1
27108	-1.30034	13.61155	-5.00586	0.000122	0.005707	1.310728	Alkbh7
48183	-1.30038	9.653774	-4.96981	0.000132	0.005815	1.24042	Celsr1
34999	-1.30042	11.08955	-4.20909	0.000641	0.010117	-0.27253	Abcb9
4090	-1.30042	12.97883	-4.5997	0.000283	0.007333	0.510509	Acadl
17992	-1.30044	9.241154	-4.63716	0.000261	0.007218	0.585007	Ndufab1
55581	-1.3006	10.48033	-5.1435	9.25E-05	0.005346	1.577822	Cryba4
57134	-1.30064	10.50062	-5.1643	8.87E-05	0.005274	1.617966	Spnb1
36220	-1.30071	9.874423	-4.5122	0.000339	0.007886	0.336066	Cnst
14585	-1.30135	11.77027	-3.99332	0.001012	0.012077	-0.70865	Ctsh
5524	-1.3015	12.18498	-4.12495	0.000766	0.010796	-0.44238	Clip4
61011	-1.30178	10.73093	-5.2722	7.13E-05	0.004827	1.82544	Mterfd3
23412	-1.30217	13.19333	-4.33438	0.000492	0.009216	-0.02024	Mrps33
6280	-1.30252	10.30784	-5.62562	3.53E-05	0.003857	2.494281	Myocd
4559	-1.30253	10.91607	-4.21566	0.000632	0.0101	-0.25928	Zfand1
3322	-1.30298	11.16963	-4.36831	0.000458	0.008954	0.04792	Suox
9524	-1.30306	10.82422	-5.10427	0.0001	0.005451	1.501914	Dguok
20342	-1.30328	11.08561	-4.19305	0.000663	0.010247	-0.30487	Exosc7
52648	-1.30332	9.019143	-4.89568	0.000153	0.005923	1.095362	Adrbk2
17826	-1.30349	10.95512	-4.40624	0.000423	0.008651	0.124019	Srsf6

4441	-1.30354	12.05415	-4.05539	0.000887	0.01149	-0.58302	Med28
16076	-1.30453	11.22702	-4.29907	0.00053	0.009469	-0.09126	Efna1
61593	-1.30459	8.185837	-3.35095	0.003972	0.02335	-2.00702	Clec1b
1767	-1.30475	9.346805	-4.15204	0.000723	0.010601	-0.38767	Ppnr
38593	-1.30477	9.477721	-4.41409	0.000416	0.008591	0.13976	Mylip
30764	-1.30512	9.797008	-4.27247	0.000561	0.009647	-0.14479	Pdp1
2675	-1.30513	10.40795	-4.74057	0.000211	0.006665	0.789934	Zfp639
25549	-1.30536	9.640888	-3.79313	0.001549	0.014578	-1.1142	Taf4a
61608	-1.30567	11.97743	-4.50756	0.000342	0.007904	0.326811	Aldh5a1
23781	-1.30609	11.67696	-4.49249	0.000353	0.00797	0.296703	Uck1
34660	-1.30712	11.69829	-4.10175	0.000804	0.011092	-0.48928	Rhoq
34927	-1.30793	10.77986	-4.41348	0.000417	0.008594	0.138532	Gm15800
39808	-1.30826	11.15181	-3.32478	0.004199	0.024144	-2.05949	Pxdn
46130	-1.30828	10.17988	-5.22396	7.86E-05	0.004964	1.73288	Slc46a3
52860	-1.30859	10.17289	-4.23347	0.000609	0.010041	-0.22336	Fkrp
60170	-1.30902	9.867918	-3.50359	0.002871	0.01976	-1.6999	Scd2
4550	-1.30935	9.772962	-4.22361	0.000622	0.0101	-0.24323	Slc4a7
31609	-1.30939	9.432871	-3.98933	0.001021	0.012136	-0.71671	C1galt1c1
14641	-1.30967	12.2814	-6.20098	1.17E-05	0.002765	3.545005	Gm5048
20491	-1.31001	10.87885	-4.54296	0.000318	0.007663	0.397476	Mpst
20352	-1.31003	10.55904	-4.34998	0.000476	0.009113	0.011107	Irak1
32345	-1.31052	9.885104	-4.00481	0.000988	0.01201	-0.68539	Gt(ROSA)26Sor
46173	-1.31065	10.98482	-4.10709	0.000795	0.011053	-0.47848	Dynlt1e
2860	-1.31084	9.060545	-4.52732	0.000329	0.007752	0.366257	Aga
27810	-1.31131	11.07518	-4.0257	0.000945	0.011764	-0.64309	Mgst1
49514	-1.31171	9.606628	-4.21382	0.000635	0.0101	-0.26299	Phf8

27639	-1.31179	8.515713	-4.62688	0.000267	0.007229	0.564573	Zfp955a
40671	-1.31248	13.13423	-4.35632	0.00047	0.009047	0.023832	Tspan3
19712	-1.31277	9.022565	-4.69427	0.000232	0.006885	0.698304	LOC100502627
40022	-1.31374	8.735284	-4.87019	0.000161	0.006012	1.045344	Kcnn2
40682	-1.314	9.487674	-4.80176	0.000186	0.006267	0.910721	Ormdl1
23130	-1.31404	12.37988	-4.66857	0.000245	0.007001	0.647359	Mllt6
28292	-1.31408	10.71722	-3.87777	0.001294	0.013381	-0.9427	Zfp664
20451	-1.31424	10.83634	-4.50542	0.000344	0.007904	0.322536	Rnf150
44708	-1.31427	13.16827	-4.96698	0.000132	0.005817	1.234894	Pdrg1
29359	-1.31478	10.04972	-4.00809	0.000981	0.011995	-0.67875	Tmem123
55616	-1.31504	10.02951	-4.76711	0.0002	0.006541	0.842382	Pigy
59603	-1.3152	9.221223	-5.33455	6.30E-05	0.004625	1.944651	Lonrf1
61909	-1.31524	11.64415	-4.63868	0.000261	0.007218	0.588021	Neurl4
31317	-1.31566	11.21222	-4.07371	0.000853	0.011338	-0.54597	Smad5
19430	-1.3158	10.83468	-4.38153	0.000446	0.008891	0.074459	Mrpl32
61679	-1.31683	9.719764	-5.66929	3.24E-05	0.003769	2.575739	Aasdhppt
50305	-1.31713	10.78541	-3.52606	0.002736	0.01928	-1.65455	Mapre2
19593	-1.31728	10.62955	-4.9396	0.00014	0.005859	1.18138	Fbxo21
43588	-1.31765	11.27738	-4.06572	0.000868	0.011414	-0.56213	Magoh
59953	-1.31785	13.44353	-4.07426	0.000852	0.011331	-0.54485	Skp1a
28360	-1.31809	11.18383	-4.85177	0.000168	0.006089	1.009151	Tsc22d1
15109	-1.31856	11.099	-4.03313	0.00093	0.011726	-0.62805	Ift20
22003	-1.3189	12.7983	-4.58997	0.000288	0.007384	0.491145	Lama2
46767	-1.31919	9.340568	-5.03907	0.000114	0.005645	1.375381	Inadl
14919	-1.31959	12.4449	-3.83759	0.001409	0.013936	-1.02411	Nr3c1
20497	-1.3197	10.4939	-4.66188	0.000248	0.007051	0.634083	Tmem25

61062	-1.31992	10.17673	-5.08179	0.000105	0.005504	1.458349	Cdc26
16907	-1.32027	7.426025	-3.7074	0.001859	0.01565	-1.28786	Pcp4
20875	-1.32071	10.39807	-4.31981	0.000508	0.009336	-0.04954	Mdp1
46409	-1.32075	12.26657	-4.91886	0.000146	0.005905	1.140777	Sgk3
4440	-1.32091	11.21032	-4.22609	0.000618	0.010087	-0.23824	Rab9
20160	-1.32134	11.39531	-4.51576	0.000337	0.007874	0.343187	Aldh4a1
52623	-1.32177	9.801266	-4.56877	0.000301	0.007502	0.448934	Tmem41b
19776	-1.32179	12.8731	-4.82249	0.000178	0.006182	0.95156	Wbp1
33595	-1.32188	11.00023	-4.50242	0.000346	0.007904	0.316539	Stat5b
45020	-1.32199	10.6072	-4.54274	0.000318	0.007663	0.39704	Tcf15
36039	-1.32229	12.18009	-4.47892	0.000363	0.008051	0.269569	Mtx2
41513	-1.32261	13.22222	-5.36744	5.89E-05	0.004449	2.007344	Ppa2
43722	-1.32354	8.925707	-4.98649	0.000127	0.005759	1.272964	Six5
53504	-1.32359	10.89061	-4.24053	0.0006	0.009984	-0.20913	Fbxo8
4501	-1.324	8.212507	-5.63397	3.48E-05	0.003851	2.509884	Egf
41106	-1.3243	10.51275	-4.10594	0.000797	0.011056	-0.48079	Dtymk
16171	-1.32446	8.894023	-5.00676	0.000122	0.005707	1.312488	Parp1
61479	-1.32447	11.74485	-4.10695	0.000795	0.011053	-0.47875	Pigp
17993	-1.32452	11.18307	-4.14461	0.000734	0.010615	-0.40266	Meis1
41621	-1.32461	12.35903	-4.66898	0.000245	0.007001	0.648173	Aifm1
58293	-1.32514	11.06602	-4.2226	0.000623	0.0101	-0.24528	Rab18
38228	-1.32522	10.84282	-4.85516	0.000167	0.006078	1.015814	Ggct
3097	-1.3255	10.69088	-3.17196	0.005806	0.029053	-2.36449	Dusp1
29036	-1.32632	8.903485	-4.08664	0.00083	0.011241	-0.51982	Cc2d2a
62913	-1.32643	11.63141	-4.85437	0.000167	0.006078	1.014265	Mrpl50
58207	-1.32647	10.49073	-4.53737	0.000322	0.007678	0.386325	Ctage5

41278	-1.32657	9.025494	-3.89961	0.001235	0.013201	-0.89845	Eef1e1
28932	-1.32728	11.52845	-4.48302	0.00036	0.008021	0.277753	LOC676974
17765	-1.32806	11.8871	-4.31597	0.000512	0.009337	-0.05726	Prosc
24127	-1.32815	8.554612	-5.47082	4.80E-05	0.004197	2.203415	Tmc7
57505	-1.32832	10.97018	-4.02549	0.000945	0.011764	-0.64352	Mogs
43502	-1.32897	12.92848	-4.71042	0.000225	0.006825	0.730294	Mmadhc
35993	-1.32906	11.77611	-4.7267	0.000217	0.006719	0.76251	Fam82b
49570	-1.32983	11.54994	-5.07983	0.000105	0.005504	1.454553	Immp1l
60646	-1.33041	11.21697	-4.74718	0.000208	0.006636	0.803017	Pin1
1914	-1.33066	9.387305	-3.75631	0.001675	0.015024	-1.1888	Tfrc
50284	-1.33077	12.76082	-4.57382	0.000298	0.007471	0.458981	Armc2
50078	-1.33107	11.41605	-4.6986	0.00023	0.006871	0.706891	Tmem37
18209	-1.33107	12.98857	-5.01519	0.00012	0.005671	1.328915	D16H22S680E
46935	-1.33187	8.20446	-4.54998	0.000313	0.007617	0.411474	Hmgcs2
26975	-1.33273	12.26314	-3.89412	0.00125	0.013205	-0.90957	Txnip
24895	-1.33313	9.832786	-4.49942	0.000348	0.007904	0.310553	Mzt2
45648	-1.33336	8.694328	-5.55338	4.08E-05	0.00398	2.358955	Apbb1
28917	-1.33382	11.68612	-4.55444	0.00031	0.007593	0.420366	Rasl2-9-ps
28795	-1.33405	10.51503	-4.42224	0.000409	0.008558	0.156093	Rbm4
36691	-1.33413	10.18972	-3.8764	0.001298	0.013407	-0.94548	Pja1
14618	-1.33416	9.908832	-4.02636	0.000944	0.011762	-0.64175	Ccdc45
42647	-1.33423	12.35997	-4.90529	0.00015	0.005914	1.114205	Sgcg
5869	-1.33431	8.50779	-3.55476	0.002574	0.018637	-1.59661	Cdon
10321	-1.3345	11.41873	-4.10358	0.000801	0.011076	-0.48558	l7Rn6
36577	-1.33454	11.13871	-4.83218	0.000175	0.006133	0.970618	Ccdc47
32073	-1.33486	12.83263	-5.13992	9.31E-05	0.005346	1.570897	Rxrg

27400	-1.33537	9.616389	-6.323	9.28E-06	0.002463	3.761457	Leng1
14351	-1.33543	11.25891	-3.83245	0.001425	0.01399	-1.03453	Ppp2r5c
38757	-1.33585	11.37792	-4.34762	0.000479	0.009113	0.006355	Ccnk
23800	-1.33619	9.898291	-4.88792	0.000156	0.005925	1.080144	Mapkapk3
27279	-1.3364	11.84933	-3.9668	0.001071	0.012374	-0.76234	Ppid
30592	-1.33671	14.49962	-5.21435	8.01E-05	0.004967	1.7144	Sdhd
15624	-1.33687	9.297134	-3.06349	0.0073	0.033285	-2.57924	Phkg1
17798	-1.33691	11.35597	-4.44372	0.000391	0.008366	0.199118	Mrpl45
1090	-1.33739	10.83653	-5.3248	6.42E-05	0.004669	1.926054	Il10rb
20549	-1.33774	8.519897	-3.01471	0.008089	0.035309	-2.67526	Rbp4
31666	-1.33833	13.07789	-4.92449	0.000144	0.005905	1.151807	Ptcd3
43274	-1.33846	8.804181	-5.45099	4.99E-05	0.004215	2.16592	Gcdh
435	-1.3397	10.77676	-3.65433	0.002082	0.016684	-1.39529	Pcsk6
38519	-1.33974	9.574478	-5.10407	0.0001	0.005451	1.501527	Mreg
14821	-1.34077	9.438196	-4.7088	0.000225	0.006827	0.72708	Synpo2
45836	-1.34121	10.80869	-3.49238	0.00294	0.019991	-1.72251	Anp32a
26171	-1.34123	12.91906	-5.67295	3.22E-05	0.003769	2.582539	Tecr
36006	-1.34189	8.924722	-5.20276	8.20E-05	0.005036	1.692104	Epb4.1l3
55820	-1.34296	11.15044	-5.53758	4.20E-05	0.00398	2.329261	Antxr2
2397	-1.34296	11.02561	-4.27385	0.000559	0.009638	-0.14201	Ttll1
25740	-1.34337	13.22247	-3.04839	0.007536	0.033928	-2.609	Myl1
33592	-1.34364	12.68751	-3.81044	0.001493	0.014332	-1.07913	Pdia6
22435	-1.34374	9.134068	-5.47262	4.78E-05	0.004197	2.20682	Dhrs11
24721	-1.34414	10.83928	-4.17469	0.000689	0.010365	-0.34193	Fam53c
37759	-1.34439	9.808112	-4.54431	0.000317	0.007663	0.400157	Triap1
12960	-1.34498	10.75778	-6.43127	7.58E-06	0.002382	3.951588	Acads

39782	-1.34503	9.946135	-4.47181	0.000369	0.008115	0.255338	Sdhaf2
26955	-1.34504	10.32776	-4.13014	0.000757	0.010755	-0.4319	Ift81
22641	-1.34516	11.06083	-4.90638	0.00015	0.005914	1.116336	Prickle1
12250	-1.34533	10.80718	-4.19087	0.000666	0.010268	-0.30928	Top1
52358	-1.34567	12.70517	-5.49946	4.53E-05	0.004091	2.257475	Mrps36
54824	-1.34604	7.79767	-3.9729	0.001057	0.012341	-0.74998	Acsm5
12000	-1.34754	11.4266	-4.95823	0.000135	0.005828	1.217786	Naa20
17127	-1.34764	9.320873	-5.81849	2.43E-05	0.00335	2.851931	Fdft1
21051	-1.34813	8.912735	-5.16422	8.87E-05	0.005274	1.617816	Rrs1
24574	-1.34828	9.82536	-4.38078	0.000446	0.008891	0.072941	Slc5a6
46317	-1.3486	12.31149	-3.36588	0.003848	0.022932	-1.97705	Serpinh1
62956	-1.34923	8.773159	-4.88609	0.000156	0.005925	1.07654	Asap3
55600	-1.34949	12.20887	-3.03314	0.007782	0.034451	-2.63902	Klf2
16557	-1.34952	9.897292	-4.58134	0.000294	0.007457	0.473966	Isoc1
58182	-1.34985	9.833498	-6.15514	1.27E-05	0.002798	3.463114	Ptov1
33740	-1.35012	12.17619	-4.18911	0.000669	0.010268	-0.31282	Ngrn
34199	-1.35027	13.26207	-4.62134	0.00027	0.007258	0.553567	Selenbp1
61862	-1.35114	9.290657	-3.79361	0.001548	0.014574	-1.11322	Igf1
14866	-1.35114	10.34661	-4.21378	0.000635	0.0101	-0.26306	Emg1
27603	-1.35134	11.88993	-4.63736	0.000261	0.007218	0.585403	Thoc7
26477	-1.35134	13.18805	-4.15067	0.000725	0.010601	-0.39042	Sspn
13982	-1.35142	13.69581	-4.6878	0.000235	0.006899	0.685483	Prpf8
22713	-1.35172	14.01442	-4.93034	0.000143	0.005878	1.163253	Dbi
59285	-1.35218	8.090381	-6.13836	1.31E-05	0.002798	3.433046	Klhdc1
44251	-1.35232	10.68909	-3.78396	0.00158	0.014646	-1.13278	Osbpl8
56336	-1.35242	14.86947	-5.26575	7.23E-05	0.004856	1.81308	Sucla2

61612	-1.35293	9.918187	-4.23421	0.000608	0.010034	-0.22186	Sfrs18
59384	-1.35294	8.595409	-4.99897	0.000124	0.005707	1.297309	Magix
55157	-1.35344	10.47049	-4.34848	0.000478	0.009113	0.008078	Manf
56194	-1.35357	10.01306	-4.83175	0.000175	0.006133	0.969787	Mipep
54782	-1.35405	11.93421	-4.8122	0.000182	0.006231	0.931284	Mad2l1bp
16534	-1.35415	11.76525	-4.96997	0.000132	0.005815	1.240723	Pxmp2
11834	-1.35424	9.490344	-5.27265	7.13E-05	0.004827	1.826306	Lpcat3
25680	-1.35536	10.29773	-4.45179	0.000385	0.008288	0.215272	Dram2
31697	-1.35593	12.17781	-4.11308	0.000785	0.010955	-0.46636	Kctd2
34141	-1.35652	9.102791	-3.71322	0.001837	0.015584	-1.27608	Nr2f1
26352	-1.35675	11.76153	-4.92957	0.000143	0.005878	1.161756	Hnrnpa2b1
8788	-1.35707	10.65962	-4.39871	0.00043	0.008694	0.10892	Tarsl2
18649	-1.35738	9.768788	-4.88126	0.000158	0.005925	1.067078	Fastkd5
57973	-1.35769	13.24405	-5.26422	7.25E-05	0.004856	1.81016	Tmem223
56826	-1.35777	13.40865	-5.49795	4.55E-05	0.004091	2.254639	Acacb
49190	-1.35822	9.80229	-4.47709	0.000365	0.008073	0.265903	Snn
38743	-1.35865	9.386396	-4.83437	0.000174	0.006133	0.974937	Gpt2
9680	-1.36103	11.43244	-4.92561	0.000144	0.005904	1.154005	Zfp629
3102	-1.36118	9.909597	-4.7555	0.000205	0.006627	0.819452	Svip
61811	-1.36227	9.722623	-2.82783	0.011958	0.04524	-3.03918	Cx3cr1
39994	-1.36322	8.288033	-4.1796	0.000682	0.010327	-0.33201	Tppp
21278	-1.3636	13.25377	-4.50063	0.000347	0.007904	0.31296	H2afv
43100	-1.36375	10.92453	-4.58298	0.000293	0.007445	0.477226	Eif4e
10703	-1.36446	9.121128	-4.57664	0.000296	0.007471	0.464608	Chadl
13726	-1.36553	9.905463	-4.98404	0.000128	0.005764	1.268188	Nmnat3
60693	-1.36591	9.241848	-5.96606	1.82E-05	0.002941	3.121936	Paqr9

36535	-1.36607	8.750261	-4.50167	0.000347	0.007904	0.315043	Tmtc4
13722	-1.36634	11.4278	-5.09079	0.000103	0.0055	1.475792	Mrps30
26205	-1.36686	10.02459	-4.28161	0.00055	0.009575	-0.1264	Gm6195
17418	-1.36718	13.1905	-4.84058	0.000172	0.006133	0.987158	Gnpat
41235	-1.36765	7.861538	-2.79185	0.012886	0.04745	-3.10844	Ptgfr
52425	-1.36873	9.54928	-3.68784	0.001939	0.016051	-1.32747	Tppp3
53113	-1.36903	12.75956	-5.98567	1.76E-05	0.002891	3.15756	Ivd
50729	-1.36936	12.71807	-4.25695	0.000579	0.009789	-0.17604	Imp3
22188	-1.36999	11.47559	-3.99803	0.001002	0.012061	-0.69911	Tra2a
19229	-1.37047	14.10246	-5.45599	4.94E-05	0.004211	2.17538	Echs1
61086	-1.37201	12.90233	-5.29757	6.78E-05	0.004788	1.874018	Prdx3
17940	-1.37246	12.12436	-4.22624	0.000618	0.010087	-0.23795	Mrpl36
13815	-1.37252	9.210762	-5.62638	3.53E-05	0.003857	2.495701	Tmem117
50770	-1.37255	13.30577	-4.45094	0.000385	0.008295	0.213587	Kpna2
54561	-1.37256	11.37448	-4.49453	0.000352	0.007957	0.300762	Ibtk
61801	-1.37283	13.07588	-4.6322	0.000264	0.007219	0.575155	Tmem66
62511	-1.37311	10.24045	-5.08764	0.000104	0.0055	1.469696	Ankrd24
21983	-1.37329	9.028897	-4.96216	0.000134	0.005828	1.225464	Scara5
51525	-1.37415	10.26162	-4.87974	0.000158	0.005925	1.064083	Cdkn1c
28938	-1.37491	12.20316	-4.11297	0.000785	0.010955	-0.46659	Amd2
14468	-1.37558	13.95792	-5.27939	7.03E-05	0.004827	1.839218	Mgrn1
13208	-1.37618	10.90748	-4.28286	0.000549	0.009571	-0.12387	Lonp2
3546	-1.3763	11.51853	-4.83283	0.000174	0.006133	0.971897	Foxred1
14769	-1.37891	14.3345	-5.27826	7.05E-05	0.004827	1.837064	Ybx1
6505	-1.37982	12.18499	-4.04713	0.000903	0.011556	-0.59972	Sh3glb1
48646	-1.38011	12.2556	-4.72051	0.00022	0.006777	0.750276	Pygb

9001	-1.38012	9.116814	-4.45606	0.000381	0.008263	0.223827	Dsc2
23672	-1.38057	10.40637	-4.83249	0.000174	0.006133	0.971245	Ythdf3
14004	-1.38134	10.37996	-4.36621	0.00046	0.008954	0.043707	Mtfr1
9076	-1.38272	9.807531	-3.80594	0.001507	0.014374	-1.08824	Clec10a
27771	-1.38308	15.70328	-6.96176	2.89E-06	0.001944	4.856819	Fhl2
7066	-1.38392	11.11824	-4.41689	0.000414	0.008573	0.145361	Tmod1
31638	-1.38446	8.417672	-4.79058	0.00019	0.006332	0.888693	Hnmt
21770	-1.3853	11.52008	-4.67647	0.000241	0.006957	0.66303	Nsmaf
3128	-1.38586	11.14194	-4.67478	0.000242	0.006964	0.65968	Mtg1
21024	-1.38626	11.79875	-4.90785	0.000149	0.005914	1.11921	Mrps31
22887	-1.38646	8.563193	-4.74641	0.000208	0.006636	0.801481	Slc47a1
24663	-1.38657	10.11521	-5.03163	0.000116	0.005645	1.360904	Uhrf1bp1
6294	-1.38789	12.11615	-4.99476	0.000125	0.005707	1.2891	Mrpl18
32887	-1.38791	9.82995	-4.00734	0.000982	0.011995	-0.68026	Golm1
61650	-1.38809	13.37887	-4.6082	0.000278	0.007325	0.527425	Eif4a2
32248	-1.38825	11.49829	-5.40169	5.51E-05	0.004397	2.072451	Pstk
40613	-1.38887	12.40011	-3.92533	0.001169	0.012871	-0.84633	Mrps6
50522	-1.38921	14.04896	-6.40978	7.89E-06	0.002382	3.913985	Idh3a
37515	-1.38922	10.27958	-4.45853	0.000379	0.008253	0.228766	Metrnl
56092	-1.38943	15.83345	-6.79244	3.91E-06	0.002278	4.572657	Hadhb
43452	-1.38953	11.90861	-4.93602	0.000141	0.005859	1.17436	Dnajc19
35206	-1.39026	9.309153	-4.44901	0.000387	0.008312	0.209719	Paip2b
27964	-1.3909	11.00273	-4.53586	0.000323	0.007678	0.383308	Lims1
18603	-1.39329	10.79236	-4.68923	0.000235	0.006899	0.68832	Trap1
55700	-1.39398	13.01608	-5.76272	2.70E-05	0.003547	2.749066	D13Mgi7
32239	-1.39411	9.939437	-4.90007	0.000152	0.005922	1.103963	Ubr3

44829	-1.39434	9.817534	-5.39512	5.58E-05	0.004397	2.059988	Rnf5
60173	-1.3949	10.62463	-3.84553	0.001386	0.013862	-1.00803	Cxadr
15554	-1.39505	12.40602	-5.23033	7.76E-05	0.004955	1.74511	Mrpl39
62843	-1.3952	9.8384	-4.26305	0.000572	0.009722	-0.16377	Vtn
35750	-1.3958	14.42254	-5.0175	0.000119	0.005671	1.333401	Rps2
47908	-1.39605	9.976987	-6.39347	8.13E-06	0.002422	3.885407	L2hgdh
54896	-1.39609	11.09211	-5.01587	0.00012	0.005671	1.330227	Mcee
30840	-1.39618	10.73606	-4.20328	0.000649	0.010138	-0.28425	Snx3
27106	-1.39624	11.9057	-6.05645	1.54E-05	0.002865	3.285701	Apip
44577	-1.39719	10.40334	-4.29134	0.000539	0.009544	-0.10681	Carns1
27821	-1.39758	7.52224	-5.27177	7.14E-05	0.004827	1.82462	Cngb3
30828	-1.39819	8.271544	-5.3979	5.55E-05	0.004397	2.065252	Macrod2
37811	-1.39893	12.0819	-4.65161	0.000254	0.007111	0.613706	Trmt2b
31003	-1.39948	10.54881	-5.25308	7.41E-05	0.004877	1.788786	Chrac1
6853	-1.40038	12.0931	-6.355	8.74E-06	0.002463	3.817824	Hadha
11122	-1.4006	11.93546	-6.12554	1.35E-05	0.002798	3.410056	Ppara
51494	-1.40139	11.83629	-4.90277	0.000151	0.005914	1.109252	Mrps17
44450	-1.4015	13.19075	-5.47961	4.72E-05	0.004174	2.220024	Glrx3
7968	-1.40202	10.4833	-4.74824	0.000208	0.006636	0.805102	Pex7
7841	-1.40372	10.42715	-4.99593	0.000125	0.005707	1.291382	Pigu
28752	-1.40414	9.234751	-4.89384	0.000154	0.005923	1.091746	Dusp23
13025	-1.40433	10.85332	-4.66578	0.000246	0.007012	0.641818	Dtd1
24247	-1.4048	9.138931	-2.91572	0.009955	0.040234	-2.86885	Aldh1b1
9532	-1.40491	11.59287	-6.13737	1.32E-05	0.002798	3.431271	Alad
34488	-1.40622	11.03271	-4.92109	0.000145	0.005905	1.145143	Hscb
41341	-1.40677	10.38896	-4.24088	0.000599	0.009984	-0.20843	Phf10

10584	-1.40784	13.29751	-4.67663	0.000241	0.006957	0.663342	Ndufs2
57113	-1.40801	9.177669	-5.09531	0.000102	0.005481	1.484556	Tnfrsf25
39003	-1.40817	9.164782	-4.31449	0.000513	0.009341	-0.06023	Sned1
20987	-1.4082	12.35518	-3.97968	0.001042	0.012279	-0.73625	Dhrs7
33408	-1.40859	10.00555	-4.34582	0.000481	0.009114	0.002734	F3
2802	-1.40936	10.58659	-4.49378	0.000352	0.007957	0.299281	Pfn2
18933	-1.4099	11.9095	-4.48275	0.000361	0.008021	0.277213	Churc1
9904	-1.41045	8.537886	-4.07559	0.00085	0.011306	-0.54215	Cd209f
4079	-1.41058	9.375603	-4.31824	0.000509	0.009336	-0.0527	Fndc4
26239	-1.41079	9.557391	-4.79811	0.000187	0.006267	0.903523	Adsl
30529	-1.41141	13.80947	-6.33996	8.99E-06	0.002463	3.791349	Chchd3
32431	-1.41212	11.82437	-4.81006	0.000183	0.006244	0.927074	Zfp706
62541	-1.41218	10.86812	-4.51824	0.000335	0.007855	0.348128	Nqo2
38378	-1.41225	8.954387	-5.18935	8.43E-05	0.005132	1.666268	Hdac9
52849	-1.41243	10.28523	-5.6548	3.34E-05	0.003769	2.548727	Ppp1r3a
57197	-1.41286	9.92007	-4.58913	0.000289	0.007384	0.489472	Rasl12
28077	-1.4132	15.13935	-6.74113	4.29E-06	0.002346	4.485666	Cox7c
8742	-1.41329	9.827552	-4.82686	0.000177	0.006166	0.960149	Mccc1
50673	-1.41347	9.713534	-5.11371	9.82E-05	0.005435	1.520193	Tmem135
46731	-1.41438	10.36086	-4.92758	0.000144	0.005891	1.157852	Eif3i
33025	-1.41442	9.743751	-4.68031	0.000239	0.006939	0.670643	Cenpa
30160	-1.41535	11.56369	-4.64212	0.000259	0.007218	0.594852	Rdh14
21682	-1.41652	10.41311	-5.33993	6.23E-05	0.004597	1.954931	Herc3
36966	-1.41777	12.42235	-4.28831	0.000542	0.009554	-0.1129	Ptpla
11174	-1.41781	15.8413	-5.814	2.45E-05	0.00335	2.843681	Atp5g2
52243	-1.41804	9.508723	-4.97921	0.000129	0.00578	1.258773	Stat5a

19649	-1.41825	11.98181	-4.91253	0.000148	0.005914	1.128388	Nup62
62797	-1.42053	11.40697	-4.6001	0.000282	0.007333	0.511312	Nod1
6654	-1.42122	11.1704	-4.5621	0.000306	0.007566	0.435645	Gcom1
59258	-1.42237	11.85838	-5.1399	9.32E-05	0.005346	1.570853	Nfs1
22629	-1.42314	10.97959	-5.37942	5.76E-05	0.004414	2.030126	Isca2
49781	-1.4234	10.52414	-5.17209	8.73E-05	0.005256	1.633002	Asb14
56748	-1.42355	10.24974	-4.34706	0.000479	0.009113	0.005242	Tmx4
12710	-1.42386	10.53029	-4.14619	0.000732	0.010607	-0.39947	Wif1
46027	-1.42584	11.42183	-3.91705	0.00119	0.012991	-0.86311	Laptm4a
15528	-1.4259	13.45937	-5.01841	0.000119	0.005671	1.335179	Txlnb
15765	-1.42762	8.577275	-5.27291	7.12E-05	0.004827	1.826806	Rnls
27924	-1.42902	10.2753	-4.74043	0.000211	0.006665	0.789668	Snapc5
23775	-1.42924	10.23622	-2.84603	0.011513	0.044222	-3.00403	Angptl4
11323	-1.42945	11.29736	-4.68252	0.000238	0.006925	0.675013	Tmem159
51254	-1.42957	9.298009	-4.72069	0.00022	0.006777	0.750616	Alox5
14973	-1.42991	8.218263	-4.58489	0.000291	0.007427	0.481048	Tmem35
13162	-1.43043	11.82194	-4.64117	0.000259	0.007218	0.592973	Larp1b
52154	-1.43047	12.4519	-4.91827	0.000146	0.005905	1.139627	Tmem106b
42146	-1.43096	11.16625	-5.11431	9.81E-05	0.005435	1.521369	Ube2e3
3299	-1.43236	13.1374	-5.59495	3.75E-05	0.0039	2.43691	Adk
15778	-1.43237	10.52954	-4.88607	0.000156	0.005925	1.076508	Akap1
62595	-1.43243	9.126505	-4.09823	0.00081	0.011112	-0.49638	Fign
28278	-1.43248	12.1479	-5.21376	8.02E-05	0.004967	1.713267	Ccbl1
57890	-1.43267	10.37766	-4.24068	0.0006	0.009984	-0.20884	Ccdc90b
50295	-1.43435	10.52782	-4.93363	0.000142	0.005859	1.169687	Glrx
21383	-1.43448	14.71661	-6.79194	3.92E-06	0.002278	4.571807	Nnt

61900	-1.43485	10.19916	-5.03099	0.000116	0.005645	1.359668	Pxmp4
2743	-1.43555	10.14682	-3.9007	0.001232	0.013196	-0.89624	Hist3h2a
37140	-1.43734	9.229171	-5.37658	5.79E-05	0.004414	2.024736	As3mt
49901	-1.44041	9.911902	-5.54449	4.15E-05	0.00398	2.342247	Ppif
16226	-1.44106	12.30367	-5.25147	7.44E-05	0.004878	1.785696	Spop
11622	-1.44177	11.39294	-3.97832	0.001045	0.012301	-0.73901	Trim11
25029	-1.44234	10.35324	-5.29578	6.80E-05	0.004788	1.870587	Hand2
9173	-1.4429	10.06372	-4.33841	0.000488	0.009154	-0.01214	Ube2n
2731	-1.44363	8.435335	-4.76623	0.0002	0.006541	0.840639	Ntf3
3729	-1.44405	14.0276	-5.27271	7.13E-05	0.004827	1.826422	Ndufb6
37566	-1.44446	10.24464	-4.69522	0.000232	0.006885	0.700183	Ccl27a
29419	-1.44486	12.69344	-4.80912	0.000183	0.006246	0.925234	Phb2
412	-1.4511	11.22656	-4.03497	0.000926	0.011716	-0.62433	Pdlim4
24851	-1.45175	12.5417	-6.01279	1.67E-05	0.002891	3.206757	Immt
9881	-1.45292	9.509397	-5.17374	8.70E-05	0.005252	1.636187	Chac2
1911	-1.45327	14.05982	-5.32303	6.44E-05	0.00467	1.92268	Mtfp1
56254	-1.4539	11.5273	-5.75127	2.76E-05	0.003584	2.727892	Ank
45735	-1.45543	12.05205	-4.68957	0.000234	0.006899	0.688987	Cish
2563	-1.45649	9.39128	-5.1634	8.88E-05	0.005274	1.616236	Ttc30b
51236	-1.45721	10.67688	-5.19362	8.36E-05	0.005108	1.674496	Mapk10
59775	-1.45798	8.409523	-3.79599	0.00154	0.014558	-1.1084	Ppl
35237	-1.45832	12.3425	-4.9562	0.000135	0.005828	1.213828	Mrpl15
17799	-1.45902	13.95474	-5.65268	3.35E-05	0.003769	2.544774	Dgat2
41696	-1.45919	11.09495	-5.3915	5.62E-05	0.004397	2.053104	Rtn2
62615	-1.46043	12.21253	-4.94898	0.000137	0.005859	1.199707	Isca1
26864	-1.4609	11.36241	-5.13428	9.42E-05	0.005379	1.560002	Oat

26573	-1.46398	9.595879	-5.38577	5.68E-05	0.00441	2.042216	Mrpl37
38104	-1.46592	12.21456	-5.57456	3.91E-05	0.003951	2.398714	Mut
14013	-1.46621	10.43516	-5.20287	8.20E-05	0.005036	1.692309	Bcap29
26584	-1.46707	11.47464	-4.83496	0.000174	0.006133	0.97609	Atad1
51352	-1.4677	10.11195	-4.5562	0.000309	0.007591	0.423866	Polr2d
42269	-1.46961	10.53663	-4.5354	0.000323	0.007678	0.382398	Rpa3
47434	-1.47222	10.72047	-5.00195	0.000123	0.005707	1.303121	Plekhh3
32475	-1.47323	12.05161	-4.50165	0.000347	0.007904	0.315008	Itpr2
48689	-1.47339	11.21806	-5.02749	0.000117	0.005645	1.352848	Acp6
54571	-1.47488	11.10022	-4.59617	0.000285	0.007333	0.503495	Sar1b
104	-1.47521	10.75973	-4.53579	0.000323	0.007678	0.383167	Tm2d1
19771	-1.47712	11.77916	-4.62511	0.000268	0.007229	0.561063	Chpt1
21702	-1.47772	9.554251	-5.31021	6.61E-05	0.004733	1.898181	Tom1l1
39457	-1.47787	13.39206	-5.41586	5.35E-05	0.004377	2.099349	Agpat3
25693	-1.47817	8.711781	-3.88005	0.001288	0.013363	-0.93807	Mnda
59299	-1.47967	12.29946	-4.95767	0.000135	0.005828	1.216703	Coq2
62933	-1.48092	11.5634	-6.17295	1.23E-05	0.002798	3.494976	Abcb8
10842	-1.48094	11.26864	-4.91194	0.000148	0.005914	1.12722	Frmd5
424	-1.48136	11.09862	-4.14333	0.000736	0.010629	-0.40525	Aldh2
40525	-1.48377	9.422393	-4.33891	0.000488	0.009152	-0.01115	Heyl
20740	-1.48417	11.34982	-5.00149	0.000123	0.005707	1.30222	Nudt6
62664	-1.48433	12.28273	-4.63183	0.000264	0.007219	0.574403	Igsf1
62112	-1.48442	9.896378	-4.32403	0.000503	0.009325	-0.04105	Gm889
20929	-1.48455	9.860341	-5.47701	4.74E-05	0.004178	2.215114	AI464131
31166	-1.48528	8.578467	-5.6024	3.70E-05	0.0039	2.450863	Oma1
42504	-1.48555	10.78867	-5.53406	4.23E-05	0.003987	2.322653	Ldhd

183	-1.48666	10.73022	-4.57997	0.000294	0.007457	0.471243	Csf1
1487	-1.48863	9.329255	-5.99434	1.73E-05	0.002891	3.173295	Slc25a29
56157	-1.4892	12.27279	-5.18183	8.56E-05	0.005182	1.651781	Agl
61737	-1.49001	13.47168	-6.02417	1.63E-05	0.002891	3.227363	Fhod3
18759	-1.49037	12.88091	-5.52521	4.31E-05	0.00399	2.305995	Gm561
7866	-1.49249	10.8618	-4.58464	0.000292	0.007427	0.480541	Irs1
5861	-1.49253	12.97224	-4.36848	0.000458	0.008954	0.048252	Fam36a
37373	-1.49267	8.565414	-5.59271	3.77E-05	0.0039	2.432732	Dok7
5663	-1.49272	12.32409	-5.0524	0.000111	0.005572	1.401292	Dld
2762	-1.49311	12.68582	-5.55922	4.03E-05	0.003964	2.369927	Msrb2
14604	-1.49355	12.25804	-4.96396	0.000133	0.005828	1.228994	Sqrdl
5834	-1.49389	9.622373	-4.06471	0.00087	0.011417	-0.56416	Art4
53634	-1.4974	12.89805	-6.62389	5.32E-06	0.002346	4.285346	Aldh6a1
60278	-1.49861	10.95829	-4.87013	0.000161	0.006012	1.04523	Elmod3
5317	-1.49895	13.54437	-5.60398	3.69E-05	0.0039	2.453815	Ndufa10
29603	-1.49898	9.679488	-4.81612	0.00018	0.006205	0.939019	Reep1
36019	-1.49972	14.71431	-5.78604	2.58E-05	0.00346	2.792141	Idh3b
18073	-1.49999	7.786052	-4.34019	0.000486	0.009137	-0.00856	Aqp4
35654	-1.50026	12.52679	-4.8061	0.000184	0.006267	0.919278	Pop5
28211	-1.50032	9.742293	-5.27558	7.09E-05	0.004827	1.831926	Tfb1m
14671	-1.50121	14.2592	-5.9942	1.73E-05	0.002891	3.173036	Acss1
62354	-1.50124	13.2853	-6.12684	1.34E-05	0.002798	3.412379	Mrps28
34312	-1.50384	11.76304	-5.56531	3.98E-05	0.003964	2.381348	Coq9
22558	-1.50651	9.578643	-5.25643	7.36E-05	0.004873	1.795221	Rpusd4
13538	-1.50745	14.42257	-6.15411	1.27E-05	0.002798	3.461262	Fndc5
39482	-1.50813	9.97464	-6.27185	1.02E-05	0.002528	3.670993	Agtr1a

30074	-1.50882	13.58949	-5.53812	4.20E-05	0.00398	2.330282	Hopx
13984	-1.5095	13.02193	-5.64266	3.42E-05	0.003805	2.526094	Ccng1
18362	-1.51005	9.794941	-6.12069	1.36E-05	0.002798	3.401352	Dusp7
17120	-1.51044	11.75696	-5.0592	0.00011	0.005572	1.414511	Ghr
43962	-1.51065	10.49204	-6.35794	8.69E-06	0.002463	3.823006	Acadsb
35616	-1.51139	9.640669	-5.39384	5.59E-05	0.004397	2.057549	Pdp2
3456	-1.51317	11.46723	-5.36869	5.88E-05	0.004449	2.009716	Klhl30
58991	-1.51333	8.663875	-6.1325	1.33E-05	0.002798	3.422534	Prrg1
44566	-1.51341	12.93376	-4.84311	0.000171	0.006133	0.99212	Abcd3
35282	-1.51374	11.0825	-5.69705	3.07E-05	0.003721	2.627368	Kptn
56975	-1.51441	11.60796	-4.72868	0.000216	0.006719	0.766431	Clcn3
26485	-1.51594	11.49391	-5.32808	6.38E-05	0.004669	1.932309	Hhatl
4992	-1.51697	7.804497	-4.21274	0.000636	0.0101	-0.26515	Kcna1
50308	-1.51922	12.03281	-5.5619	4.01E-05	0.003964	2.374957	Mrpl42
1698	-1.52033	12.94525	-5.36481	5.93E-05	0.004457	2.002325	Fkbp3
60434	-1.52103	10.76165	-4.86629	0.000163	0.006036	1.037686	Dip2c
54100	-1.52132	13.07983	-6.05087	1.55E-05	0.002865	3.275632	Ndufs8
62669	-1.52236	7.420659	-3.02658	0.00789	0.03472	-2.65192	Upk3b
51362	-1.52274	10.19716	-4.88284	0.000157	0.005925	1.070167	Zfp467
44891	-1.52442	9.18991	-5.53129	4.26E-05	0.00399	2.317431	Kcnh2
34422	-1.52467	9.852186	-5.04095	0.000114	0.00564	1.379045	Mmgt1
46587	-1.52555	10.12692	-5.25779	7.34E-05	0.004873	1.797821	Gzmm
59750	-1.52571	10.28941	-4.88029	0.000158	0.005925	1.065176	Lnpep
35735	-1.52926	11.47719	-5.80133	2.51E-05	0.003391	2.820331	Ccbl2
4807	-1.53023	9.71162	-5.49267	4.59E-05	0.004091	2.244677	Chordc1
8622	-1.53112	12.96376	-4.78106	0.000194	0.006401	0.869914	Apoo

49340	-1.53217	11.25448	-5.40196	5.50E-05	0.004397	2.072977	Tmtc1
41407	-1.53232	13.23306	-6.69193	4.70E-06	0.002346	4.401865	Mgst3
37837	-1.53288	9.796742	-5.14754	9.17E-05	0.00532	1.585619	Gmpr
42208	-1.53371	9.40227	-4.56034	0.000307	0.007568	0.432133	Blnk
23831	-1.53491	10.10685	-5.42019	5.31E-05	0.004364	2.107567	Efcab2
48725	-1.5365	13.228	-4.13843	0.000744	0.010683	-0.41515	Dbp
1732	-1.53795	10.46347	-4.08522	0.000833	0.011248	-0.52268	Ddo
28588	-1.5387	11.24534	-5.35177	6.08E-05	0.004544	1.977497	Tmem50b
47913	-1.53896	13.17682	-5.6578	3.32E-05	0.003769	2.554325	Hspe1
6513	-1.53992	10.60514	-4.35446	0.000472	0.009074	0.020109	Hsd11b1
48624	-1.5409	11.10645	-5.16101	8.93E-05	0.005274	1.611625	Osgepl1
28693	-1.54112	10.37393	-5.8108	2.46E-05	0.00335	2.837787	Pcca
39012	-1.54427	8.945415	-4.7998	0.000187	0.006267	0.906866	Tbc1d10c
44048	-1.54462	11.20974	-4.89898	0.000152	0.005922	1.101819	Slc25a38
38196	-1.54496	11.05239	-5.24401	7.55E-05	0.004894	1.771389	Maob
3168	-1.54561	12.40313	-4.57302	0.000299	0.007471	0.457405	Camk2g
49854	-1.54584	10.52771	-5.81332	2.45E-05	0.00335	2.842428	Bcl2l13
44846	-1.54663	14.72346	-7.417	1.30E-06	0.001253	5.598617	Fh1
31565	-1.5469	11.55127	-5.40055	5.52E-05	0.004397	2.070294	Akr1b10
41401	-1.54692	9.998018	-5.34917	6.11E-05	0.004552	1.972549	Wdr33
41655	-1.54698	8.441338	-5.10622	9.97E-05	0.005451	1.50569	Adamts15
61597	-1.54826	12.03098	-5.10204	0.000101	0.00546	1.497592	Bves
23339	-1.54832	11.02035	-6.28911	9.89E-06	0.002476	3.701567	Atp2a1
17142	-1.54856	13.48962	-4.65845	0.00025	0.007074	0.62727	Hspd1
21594	-1.54955	7.512729	-3.99077	0.001018	0.012116	-0.7138	Ccl11
52072	-1.5505	12.5042	-5.01372	0.00012	0.005671	1.326047	Rdm1

45795	-1.55068	11.64267	-5.99049	1.74E-05	0.002891	3.166306	Tmem70
14719	-1.5533	9.840662	-4.51198	0.000339	0.007886	0.335643	Pcmtd2
30508	-1.55378	12.37867	-5.93577	1.93E-05	0.003017	3.066768	Ephx2
37771	-1.55454	8.53777	-3.44383	0.00326	0.021057	-1.82033	Efemp1
32184	-1.5583	9.483774	-5.9917	1.74E-05	0.002891	3.168501	Tmx1
60714	-1.55936	10.43302	-5.46337	4.87E-05	0.004209	2.189336	Tfam
38721	-1.56475	9.559228	-6.29209	9.83E-06	0.002476	3.706846	Ankrd32
2218	-1.5648	11.37052	-4.88299	0.000157	0.005925	1.070463	Lrrc14b
15668	-1.57138	11.99299	-4.71912	0.00022	0.006778	0.747512	Dcun1d2
15946	-1.57487	9.471999	-4.28255	0.000549	0.009571	-0.1245	Lamb3
20006	-1.57528	12.73443	-5.57946	3.87E-05	0.003938	2.407905	Pdha1
57620	-1.57985	11.61117	-4.89464	0.000154	0.005923	1.093314	Mapk14
19663	-1.58032	13.34705	-5.49365	4.59E-05	0.004091	2.246526	Peci
11560	-1.58086	14.3113	-5.50721	4.46E-05	0.004091	2.272096	Acsl1
28304	-1.58095	15.37405	-6.5997	5.56E-06	0.002346	4.243747	Acadm
45071	-1.58355	13.48359	-5.74288	2.81E-05	0.003622	2.712357	Rit1
28266	-1.58419	12.08947	-5.56248	4.00E-05	0.003964	2.376036	Ifngr2
32268	-1.58481	8.751371	-5.22774	7.80E-05	0.004955	1.740148	Meig1
52198	-1.58817	11.89584	-4.69621	0.000231	0.006885	0.702158	Cacybp
5257	-1.5884	11.26476	-5.92596	1.97E-05	0.003017	3.048889	Gpt
8375	-1.59457	11.48897	-5.27281	7.13E-05	0.004827	1.826624	Ppip5k2
6808	-1.59471	10.72269	-4.27501	0.000558	0.009625	-0.13967	Gstt1
50000	-1.5967	10.86787	-5.1306	9.49E-05	0.005402	1.55288	Tbc1d4
21446	-1.60583	9.858634	-5.32638	6.40E-05	0.004669	1.929061	Unc45b
4489	-1.60645	10.21174	-8.30361	2.97E-07	0.000595	6.952157	Actr3b
6504	-1.61057	10.62167	-4.46563	0.000374	0.008171	0.24297	Pla2g5

44626	-1.61108	12.84586	-6.26086	1.04E-05	0.002553	3.651506	Ryr2
49578	-1.61222	10.67876	-6.06873	1.50E-05	0.002865	3.307856	Cited4
27254	-1.61237	13.28823	-5.75761	2.73E-05	0.003561	2.739615	Gfm1
45427	-1.61586	8.261361	-6.14671	1.29E-05	0.002798	3.44802	Ccdc69
599	-1.6167	10.51077	-5.38902	5.65E-05	0.004397	2.048391	Zfyve21
62133	-1.61716	10.82157	-4.60441	0.00028	0.007327	0.519883	Rcor2
23035	-1.61916	10.30078	-5.09613	0.000102	0.005481	1.486154	Gcat
33789	-1.62198	9.385721	-6.9571	2.91E-06	0.001944	4.84905	Schip1
36458	-1.62339	9.759183	-6.04495	1.57E-05	0.002865	3.264942	Fam78a
27814	-1.62442	13.29098	-6.46435	7.13E-06	0.002346	4.009309	Auh
50765	-1.62543	12.56899	-5.14061	9.30E-05	0.005346	1.572223	Entpd5
49685	-1.62757	13.06643	-6.0969	1.42E-05	0.002822	3.358595	Pcp4l1
41081	-1.62867	10.50866	-7.30369	1.58E-06	0.001432	5.416995	Tmod4
39732	-1.62933	7.96266	-5.1476	9.17E-05	0.00532	1.585735	Fbp2
1818	-1.63005	10.52091	-6.05567	1.54E-05	0.002865	3.284301	Pkia
32774	-1.63198	9.099509	-4.49994	0.000348	0.007904	0.311591	Erbb4
19521	-1.63365	14.02885	-5.92589	1.97E-05	0.003017	3.048753	Plin5
38740	-1.63382	9.824129	-6.03819	1.59E-05	0.002871	3.252711	Ttc32
18714	-1.63406	12.22717	-5.78159	2.61E-05	0.00346	2.783917	Fam174b
60765	-1.63605	11.56545	-5.89304	2.10E-05	0.003091	2.988737	Nudt7
33070	-1.63911	11.4178	-4.81642	0.00018	0.006205	0.939598	Gypc
26956	-1.64168	8.758386	-6.68902	4.72E-06	0.002346	4.396885	Acot2
27391	-1.64493	10.02857	-4.71543	0.000222	0.006801	0.740218	Mmd
8825	-1.64558	10.6057	-4.90925	0.000149	0.005914	1.121953	Uros
2016	-1.64634	11.58627	-5.21901	7.94E-05	0.004967	1.723367	Tmem65
57306	-1.6476	14.75804	-6.41071	7.88E-06	0.002382	3.915616	Eci1

25369	-1.64827	9.704746	-5.87381	2.18E-05	0.00314	2.95352	Dhrs4
38643	-1.64978	13.34869	-4.29309	0.000537	0.009527	-0.10329	Pdk4
43108	-1.65128	8.595737	-2.77187	0.013431	0.048675	-3.14677	Apoc1
56237	-1.65438	8.793869	-5.82661	2.39E-05	0.003341	2.866874	LOC100502705
4792	-1.6564	15.21039	-6.09957	1.41E-05	0.002822	3.363391	Decr1
60495	-1.65666	11.70956	-6.35971	8.66E-06	0.002463	3.826115	Amacr
61508	-1.65926	11.59778	-5.82501	2.40E-05	0.003341	2.863941	Pex11a
32400	-1.65953	11.06317	-5.05575	0.00011	0.005572	1.4078	Mrm1
5030	-1.66336	13.12915	-5.65363	3.35E-05	0.003769	2.546546	Mlycd
43464	-1.66427	10.53045	-4.9534	0.000136	0.005848	1.208364	Tspan18
45727	-1.67089	7.894218	-4.50275	0.000346	0.007904	0.317198	Slc38a5
21092	-1.67165	11.23305	-5.40202	5.50E-05	0.004397	2.073093	Tmem126a
12430	-1.67238	14.07433	-5.99817	1.72E-05	0.002891	3.180244	Etfdh
47474	-1.67251	12.27016	-5.98815	1.75E-05	0.002891	3.162067	Slc25a42
53146	-1.67361	9.369377	-6.50974	6.56E-06	0.002346	4.088253	Atg10
4085	-1.67371	11.81808	-5.07713	0.000106	0.005504	1.449315	Yipf7
5530	-1.67579	13.31883	-4.9686	0.000132	0.005817	1.238044	Cpt2
51528	-1.67733	9.412687	-5.4553	4.95E-05	0.004211	2.174062	Adrb1
47289	-1.68062	10.38454	-6.30189	9.65E-06	0.002476	3.724166	Agpat9
25436	-1.68222	8.86688	-6.58194	5.74E-06	0.002346	4.213148	Il15
48325	-1.68235	11.5673	-5.94979	1.88E-05	0.002985	3.092317	Nampt
44094	-1.68278	9.750779	-5.21462	8.01E-05	0.004967	1.714914	Arl4d
22207	-1.69242	9.908921	-3.17435	0.005777	0.028954	-2.35975	Plagl1
5364	-1.69706	8.532781	-5.25715	7.35E-05	0.004873	1.796602	Aldh1a2
5680	-1.7007	10.48002	-4.0489	0.0009	0.011533	-0.59615	Psd3
60658	-1.70341	11.0853	-5.01354	0.00012	0.005671	1.325701	Leprel4

35668	-1.70498	11.24023	-4.41951	0.000412	0.008563	0.150613	Sox4
36071	-1.70602	9.131936	-4.14665	0.000731	0.010607	-0.39855	Casq1
61718	-1.70634	10.02511	-7.0443	2.49E-06	0.001942	4.993703	Apobec2
30311	-1.71162	13.46802	-5.86862	2.20E-05	0.003151	2.944012	Slc25a34
4718	-1.71204	9.471522	-4.08155	0.000839	0.011264	-0.53011	Itm2a
25006	-1.715	8.262396	-5.37794	5.77E-05	0.004414	2.027326	Cacna1g
49579	-1.71698	10.10268	-5.72269	2.92E-05	0.003693	2.674952	Lpin1
47223	-1.71959	12.97694	-5.29295	6.84E-05	0.004788	1.865173	Plbd1
57143	-1.72194	9.668839	-5.61769	3.59E-05	0.003898	2.479467	Ppm1k
3690	-1.72518	9.91569	-4.81949	0.000179	0.006189	0.94564	Ppp1r3d
33128	-1.72639	10.42278	-6.0445	1.57E-05	0.002865	3.264128	Retsat
36941	-1.73049	8.069063	-3.03398	0.007768	0.034426	-2.63737	Cyp2s1
1871	-1.73186	8.869191	-9.29633	6.39E-08	0.000352	8.331531	Ctf1
62074	-1.73786	15.65045	-7.51172	1.10E-06	0.001159	5.748933	Acaa2
46874	-1.73983	12.59787	-5.65378	3.34E-05	0.003769	2.546835	Dnaja1
12417	-1.74176	11.20282	-5.12212	9.66E-05	0.005429	1.536482	Reep5
40017	-1.74334	9.184212	-5.90645	2.05E-05	0.003048	3.013247	Foxo6
24047	-1.75101	12.60602	-6.31602	9.40E-06	0.002466	3.749124	Bckdhb
57010	-1.75241	10.46499	-5.12771	9.55E-05	0.005402	1.547295	Dnmt3a
33769	-1.75303	11.99214	-5.93638	1.93E-05	0.003017	3.06789	Mlf1
62069	-1.75594	11.10961	-5.60361	3.69E-05	0.0039	2.453126	Lyrm5
62293	-1.75772	9.87856	-6.13424	1.32E-05	0.002798	3.425654	Slc25a20
38934	-1.76424	11.40946	-5.8888	2.12E-05	0.003091	2.980975	Fam131a
17337	-1.76647	12.88513	-7.4137	1.31E-06	0.001253	5.593362	Gm5595
1238	-1.76842	9.842412	-5.23365	7.71E-05	0.004951	1.751491	Abhd1
8230	-1.77132	9.788115	-3.79045	0.001558	0.014596	-1.11964	Tbx5

51685	-1.77264	8.098608	-3.68494	0.001951	0.016103	-1.33333	Rspo1
13384	-1.77386	10.51329	-5.87708	2.17E-05	0.00314	2.959511	Smtnl2
33250	-1.78735	11.8957	-5.53898	4.19E-05	0.00398	2.331898	Rnf207
49863	-1.78842	10.2521	-7.65912	8.60E-07	0.001159	5.98008	Fkbp4
10368	-1.7901	8.954502	-5.70382	3.03E-05	0.003713	2.639939	Gm14492
7121	-1.79455	11.07483	-7.03286	2.54E-06	0.001942	4.974798	Mif4gd
20404	-1.79586	8.389867	-5.18523	8.50E-05	0.00516	1.658331	Kcnv2
46423	-1.80407	10.49223	-6.32646	9.22E-06	0.002463	3.767555	Slc25a26
61709	-1.80527	8.532154	-3.26562	0.004761	0.025854	-2.17786	Fos
7341	-1.81129	9.925557	-5.7139	2.97E-05	0.003707	2.658656	Lrrc3b
36833	-1.81202	10.54078	-5.58973	3.79E-05	0.0039	2.427143	Mkks
28236	-1.81628	10.34827	-6.53204	6.29E-06	0.002346	4.126913	Suclg2
12976	-1.81841	11.79867	-6.72764	4.40E-06	0.002346	4.462729	Cmbl
18720	-1.81905	14.44299	-6.96484	2.87E-06	0.001944	4.861936	Pln
1796	-1.82582	7.997714	-3.22055	0.005238	0.027383	-2.26781	Nkain4
18895	-1.85008	10.20442	-6.51341	6.51E-06	0.002346	4.094616	Slc36a2
60864	-1.8501	11.10869	-6.60532	5.50E-06	0.002346	4.253425	Crhr2
60310	-1.85166	8.678187	-5.99654	1.72E-05	0.002891	3.177281	Itgb6
41816	-1.85702	9.730978	-6.37705	8.39E-06	0.002463	3.856598	Impa2
62427	-1.86269	10.08486	-5.88863	2.12E-05	0.003091	2.980672	Hfe2
56701	-1.86281	11.64012	-6.47032	7.05E-06	0.002346	4.019705	Fyco1
48055	-1.89115	9.412857	-3.89352	0.001251	0.013205	-0.91078	Ppbp
36154	-1.90057	12.18781	-6.49399	6.75E-06	0.002346	4.060895	Hsdl2
24508	-1.90988	14.16924	-7.58385	9.77E-07	0.001159	5.862455	Ech1
16365	-1.91022	12.89577	-6.62027	5.35E-06	0.002346	4.279123	Mid1ip1
1520	-1.91029	9.890778	-5.4149	5.36E-05	0.004377	2.097532	Slc26a6

6069	-1.91524	10.33247	-3.53843	0.002665	0.018966	-1.62959	Dpt
3285	-1.93699	10.34612	-6.7145	4.51E-06	0.002346	4.440356	P2ry1
26549	-1.94198	12.26315	-6.10939	1.39E-05	0.002822	3.381051	Mitf
50713	-1.96966	12.14898	-5.91234	2.02E-05	0.003048	3.024022	Gstk1
13753	-1.97364	12.43893	-7.1488	2.07E-06	0.001757	5.165488	Rpl3l
1890	-1.98332	9.635574	-6.55505	6.03E-06	0.002346	4.16672	Asb5
56206	-2.01009	10.4205	-4.57312	0.000299	0.007471	0.457601	Dbh
29393	-2.01067	10.24062	-4.44361	0.000391	0.008366	0.198911	Inmt
61703	-2.01589	12.50746	-6.24313	1.08E-05	0.00261	3.620039	Trdn
46564	-2.02017	8.748218	-2.81542	0.01227	0.045912	-3.06309	Car3
57091	-2.03619	13.65286	-6.79021	3.93E-06	0.002278	4.568884	Dhrs7c
44954	-2.03716	12.16862	-5.99041	1.74E-05	0.002891	3.166168	Dnajb1
36866	-2.04616	12.10173	-7.54931	1.04E-06	0.001159	5.808195	Rbfox1
38860	-2.06155	11.63643	-7.2881	1.62E-06	0.001432	5.391849	Fitm1
58134	-2.08948	8.907501	-6.07995	1.47E-05	0.002865	3.328076	Epha4
60277	-2.13559	10.74043	-4.73512	0.000213	0.006665	0.779177	Lgals4
29187	-2.18148	10.19913	-6.85022	3.53E-06	0.002239	4.670132	Kcnd2
62323	-2.19028	9.479667	-4.30833	0.00052	0.009396	-0.07263	Slc22a1
14790	-2.20163	11.00686	-6.10598	1.40E-05	0.002822	3.374926	Kcnj5
5591	-2.23418	11.00894	-5.49816	4.55E-05	0.004091	2.255027	Penk
39377	-2.24455	8.938597	-4.0468	0.000904	0.011557	-0.60039	Aldob
18419	-2.25368	8.298277	-3.39622	0.003608	0.022229	-1.9161	Muc16
2337	-2.29512	10.64599	-4.22815	0.000616	0.010087	-0.23409	Pfkfb1
56598	-2.3298	11.17527	-8.54539	2.02E-07	0.000445	7.300984	Efnb3
42075	-2.35523	11.7021	-6.49371	6.75E-06	0.002346	4.060403	Ano10
62398	-2.38258	10.27594	-5.42228	5.28E-05	0.004362	2.111538	Angpt1

16156	-2.50344	10.53236	-8.93006	1.11E-07	0.000409	7.83869	G0s2
19496	-2.52166	10.81939	-6.62346	5.32E-06	0.002346	4.284608	Ces1d
18121	-2.56884	9.083365	-3.03901	0.007686	0.034297	-2.62748	Cfd
14967	-2.6053	12.27879	-5.46395	4.86E-05	0.004209	2.190438	Aqp1
10748	-2.63345	9.588391	-5.06972	0.000107	0.005555	1.43492	Acot1
54909	-2.78008	9.710497	-6.6354	5.21E-06	0.002346	4.305108	Retnla
11380	-3.12552	9.036671	-4.24688	0.000592	0.009918	-0.19635	Msln

Sheet S2 The differentially expressed genes (DEGs) of pressure overload (PO)

ID	adj.*P*.Val	*P*.Value	t	B	logFC	Gene.symbol
ILMN_2625893	4.56E-05	1.55E-07	13.6958	8.099738	3.499986	Ces1d
ILMN_1214402	6.44E-06	5.69E-10	25.22372	13.03971	3.149162	Wnk2
ILMN_3139875	6.29E-05	3.39E-07	12.55281	7.333634	3.102932	Acot1
ILMN_2476329	1.98E-05	1.70E-08	17.46218	10.17938	3.019201	Whrn
ILMN_2644229	5.68E-06	3.00E-10	27.02079	13.52021	2.61044	Kcnd2
ILMN_2421890	0.000159	2.22E-06	10.14752	5.447442	2.55568	Acacb
ILMN_2588249	8.21E-05	6.22E-07	11.72701	6.731469	2.513091	Plin4
ILMN_2750912	0.000149	1.95E-06	10.29749	5.577587	2.4777	Fyco1
ILMN_2909238	0.002617	1.60E-04	6.039556	1.004214	2.472191	Sptb
ILMN_2710139	0.000252	5.02E-06	9.232003	4.610976	2.459956	Ppargc1a
ILMN_3134598	3.81E-05	9.92E-08	14.39073	8.53121	2.371887	Pdp2
ILMN_1229315	0.00174	9.23E-05	6.483259	1.580675	2.305932	Tns1
ILMN_2853957	0.000308	7.15E-06	8.856527	4.245647	2.30006	Dag1
ILMN_1235374	9.73E-06	2.67E-09	21.35305	11.79911	2.29782	Decr1
ILMN_1258086	0.000108	1.11E-06	10.98001	6.147368	2.275773	Setd1b
ILMN_2724545	0.000565	1.80E-05	7.937377	3.290734	2.274738	Sbk1
ILMN_2606973	3.87E-05	1.05E-07	14.29744	8.474696	2.244942	Tmem201
ILMN_2688415	3.78E-05	9.44E-08	14.46873	8.578142	2.233924	Vwa8
ILMN_1250469	0.003232	2.16E-04	5.804546	0.687756	2.203809	Bcl9l
ILMN_2634905	0.000272	5.71E-06	9.093842	4.47813	2.203534	Fbp2
ILMN_2665095	7.03E-05	4.24E-07	12.24306	7.112844	2.187733	Casz1
ILMN_2475156	0.02285	3.01E-03	3.961912	-2.06541	2.168751	Xist
ILMN_1236136	2.97E-05	5.88E-08	15.24314	9.028547	2.153289	Corin
ILMN_2978040	0.003241	2.17E-04	5.799873	0.681384	2.145563	Myh8
ILMN_2855423	2.87E-05	5.04E-08	15.5035	9.173904	2.143011	Ube3b
ILMN_2427454	9.73E-06	2.42E-09	21.58112	11.88123	2.105052	Npc1
ILMN_2769795	0.0001	9.34E-07	11.19894	6.322664	2.058932	Phkg1
ILMN_2698757	2.79E-05	4.00E-08	15.90145	9.390474	2.053724	Ppm1l
ILMN_2716085	3.78E-05	9.63E-08	14.43645	8.558757	2.012081	Pex6
ILMN_2964785	0.000179	2.73E-06	9.908762	5.236368	2.005951	Zfhx2
ILMN_2710419	0.005484	4.52E-04	5.252232	-0.08713	1.984573	Plec

ILMN_2672624	0.000157	2.17E-06	10.1716	5.468464	1.980454	Hcn2
ILMN_2617265	0.000198	3.31E-06	9.6882	5.037043	1.977809	Ipo13
ILMN_2835423	0.022688	2.98E-03	3.968202	-2.05525	1.977421	Cfd
ILMN_1259322	0.001067	4.63E-05	7.071086	2.30372	1.964091	Pdk4
ILMN_2591440	3.99E-05	1.19E-07	14.0971	8.35188	1.955014	Noct
ILMN_2858399	5.86E-05	2.82E-07	12.81367	7.515061	1.953973	Fitm2
ILMN_2628174	6.61E-05	3.72E-07	12.42161	7.240835	1.941483	Acss1
ILMN_2906855	9.7E-05	8.84E-07	11.26912	6.37812	1.930391	Ky
ILMN_1247614	0.000166	2.44E-06	10.03698	5.35032	1.884305	Dusp18
ILMN_2672221	5.68E-05	2.64E-07	12.90991	7.580978	1.884042	Fhod3
ILMN_3128792	0.000118	1.34E-06	10.75373	5.962452	1.88344	Ttn
ILMN_2601705	0.000719	2.58E-05	7.595583	2.911971	1.868931	Pirt
ILMN_2868013	2.54E-05	3.01E-08	16.40744	9.656466	1.863118	Pank1
ILMN_2637661	3.8E-05	9.82E-08	14.40542	8.540076	1.857681	Prpf8
ILMN_2675785	0.008946	8.62E-04	4.794488	-0.76274	1.842043	Myo18b
ILMN_1215246	9.03E-05	7.65E-07	11.45604	6.524126	1.83291	Mccc1
ILMN_2788984	0.000238	4.63E-06	9.319363	4.694047	1.829773	Acaa2
ILMN_1222261	0.000111	1.18E-06	10.90384	6.085555	1.826972	Gon4l
ILMN_1241229	6.84E-05	4.00E-07	12.32132	7.169191	1.818258	Prrc2c
ILMN_2750431	9.73E-06	1.62E-09	22.52915	12.20956	1.804552	Dst
ILMN_2701815	4.88E-05	1.75E-07	13.51714	7.984784	1.803122	Plxna2
ILMN_2689187	0.003061	2.01E-04	5.859715	0.762749	1.802159	Ahdc1
ILMN_3151642	8.1E-05	6.06E-07	11.76065	6.756862	1.80189	Myocd
ILMN_2874554	0.000159	2.28E-06	10.1164	5.4202	1.797148	Aldh4a1
ILMN_1227398	0.000288	6.40E-06	8.97262	4.360064	1.797044	Agt
ILMN_2713622	0.002859	1.82E-04	5.936978	0.867047	1.792968	Ldb3

ILMN_2723474	2.97E-05	5.93E-08	15.22943	9.02081	1.785262	Adcy9
ILMN_2691157	0.005024	4.02E-04	5.338637	0.037003	1.771737	Dctn1
ILMN_3005873	0.000545	1.69E-05	7.993291	3.351426	1.758297	Sort1
ILMN_2700107	0.000138	1.69E-06	10.4671	5.722588	1.755247	Polr1a
ILMN_2481117	0.00033	7.95E-06	8.747069	4.136545	1.746601	Man2a2
ILMN_1254104	0.000313	7.31E-06	8.833558	4.222852	1.741676	Prepl
ILMN_1241965	3.53E-05	8.10E-08	14.71566	8.724782	1.720165	Mitf
ILMN_3125920	0.002462	1.47E-04	6.106208	1.092548	1.714946	Mast2
ILMN_2997002	4.53E-05	1.48E-07	13.76305	8.142576	1.714916	Pigo
ILMN_1236539	0.000207	3.59E-06	9.599693	4.955857	1.712113	Pfkl
ILMN_1253692	0.000663	2.26E-05	7.719013	3.05031	1.707176	Glg1
ILMN_2635784	0.000149	1.96E-06	10.29517	5.575592	1.704032	Gpc1
ILMN_2766727	0.000206	3.55E-06	9.610816	4.966098	1.695791	Chrm2
ILMN_2670472	7.81E-05	5.30E-07	11.93833	6.889743	1.691595	AI464131
ILMN_2662191	0.000124	1.44E-06	10.65981	5.884562	1.691379	Oplah
ILMN_1226447	0.000583	1.87E-05	7.8972	3.246906	1.688153	Sema4b
ILMN_1233987	0.002452	1.46E-04	6.111291	1.099259	1.682742	Hipk2
ILMN_2855590	0.000159	2.28E-06	10.11753	5.421194	1.682209	Map7d1
ILMN_2782634	0.000279	5.96E-06	9.04818	4.433824	1.68078	Cobll1
ILMN_1218058	0.000186	2.99E-06	9.80498	5.143106	1.675331	Atp1a1
ILMN_3004553	0.00031	7.23E-06	8.845198	4.23441	1.674219	Pfkm
ILMN_2476341	0.00314	2.08E-04	5.833221	0.726789	1.668173	Ago2
ILMN_2752030	3.99E-05	1.17E-07	14.13358	8.374393	1.66526	Cacna2d1
ILMN_2670713	7.81E-05	5.34E-07	11.9292	6.882961	1.663183	Cyfip2
ILMN_1229216	0.001433	7.02E-05	6.711323	1.866573	1.662729	Zbtb16
ILMN_2707207	0.00026	5.26E-06	9.181283	4.562418	1.660953	Hhatl

ILMN_1240274	0.000539	1.67E-05	8.007697	3.367007	1.660495	Prrc2b
ILMN_3131197	0.000204	3.45E-06	9.641804	4.994571	1.656746	Repin1
ILMN_2784078	0.002397	1.42E-04	6.135247	1.130839	1.652211	Mmp15
ILMN_2459899	0.000353	8.96E-06	8.623744	4.012174	1.650326	Adamtsl4
ILMN_2687745	0.004422	3.33E-04	5.476932	0.233434	1.649074	Pnpla2
ILMN_2601453	0.000105	1.01E-06	11.09805	6.242318	1.648951	Fam78a
ILMN_2745889	7.93E-05	5.59E-07	11.86827	6.837593	1.647661	Acot2
ILMN_2842338	0.000586	1.90E-05	7.883615	3.232046	1.647289	Tbc1d10c
ILMN_1222829	0.000745	2.73E-05	7.544697	2.854418	1.641107	Ints1
ILMN_1238309	5.93E-05	3.02E-07	12.71473	7.446728	1.63327	Rbfox1
ILMN_2713060	0.000106	1.05E-06	11.05383	6.206868	1.629204	Fam131a
ILMN_1236742	0.002639	1.62E-04	6.030526	0.992199	1.626129	Alpk2
ILMN_1221142	0.000769	2.85E-05	7.504514	2.808753	1.62498	Ppard
ILMN_1236008	4.2E-05	1.28E-07	13.99436	8.288114	1.6239	Isoc2a
ILMN_1259206	0.000108	1.11E-06	10.97979	6.147188	1.620509	Hrc
ILMN_1228497	0.000944	3.85E-05	7.233534	2.495714	1.620293	Esrrb
ILMN_2604227	5.13E-05	2.05E-07	13.27472	7.82604	1.615955	Sugp2
ILMN_1244134	2.79E-05	4.27E-08	15.78834	9.329595	1.615777	Lrp4
ILMN_2923615	0.00033	7.97E-06	8.744081	4.13355	1.614287	Ppm1k
ILMN_2664660	9.73E-06	2.55E-09	21.45983	11.83771	1.613507	Aldh5a1
ILMN_1253791	2.97E-05	6.23E-08	15.14692	8.974071	1.611626	Kcnv2
ILMN_2594899	6.42E-05	3.54E-07	12.49293	7.291411	1.611124	Med26
ILMN_1220498	0.003704	2.60E-04	5.663466	0.494004	1.610212	Pygm
ILMN_2450031	0.007989	7.44E-04	4.897239	-0.60847	1.609738	Atp2a2
ILMN_2623591	6.42E-05	3.56E-07	12.48588	7.286427	1.608274	Apbb1
ILMN_2756704	0.00189	1.03E-04	6.393624	1.466399	1.604433	Parm1

ILMN_1243455	1.12E-05	3.69E-09	20.61387	11.52411	1.603713	Celsr2
ILMN_2709240	8.07E-05	5.88E-07	11.80076	6.78704	1.602214	Cpt1b
ILMN_2832641	0.00057	1.82E-05	7.926458	3.278841	1.599795	Ndst1
ILMN_1222609	0.000336	8.30E-06	8.702883	4.092162	1.593185	Igfals
ILMN_1216617	0.006509	5.71E-04	5.083904	-0.33207	1.593121	Kmt2d
ILMN_2723881	5.48E-05	2.45E-07	13.01598	7.653012	1.587701	Hcfc1
ILMN_2825080	2.97E-05	6.00E-08	15.20907	9.009303	1.585666	Ldhd
ILMN_1214500	0.000115	1.27E-06	10.81244	6.0108	1.581055	Rgma
ILMN_1218956	0.029453	4.18E-03	3.753131	-2.40518	1.577877	Tead1
ILMN_2765759	0.000392	1.04E-05	8.471875	3.856871	1.57608	Asb2
ILMN_1232261	0.000272	5.72E-06	9.092641	4.476968	1.575023	Ctnnal1
ILMN_2717777	0.000126	1.49E-06	10.61663	5.848523	1.572084	Polrmt
ILMN_2771709	0.00296	1.91E-04	5.899653	0.816767	1.571027	Ppargc1b
ILMN_2757232	0.00056	1.77E-05	7.953976	3.308788	1.567318	Aqp4
ILMN_2686721	0.000179	2.75E-06	9.897338	5.226147	1.56467	Tap2
ILMN_1249691	6.92E-05	4.14E-07	12.27507	7.13594	1.564006	Mlycd
ILMN_2524251	1.54E-05	7.83E-09	19.00137	10.87354	1.563018	Tnik
ILMN_2610156	0.000417	1.14E-05	8.38539	3.767355	1.561065	Neo1
ILMN_3159149	0.000681	2.37E-05	7.676229	3.00256	1.556875	Rusc2
ILMN_2658566	0.004019	2.89E-04	5.582286	0.381221	1.55609	Midn
ILMN_2690203	0.000169	2.52E-06	10.00037	5.31792	1.550523	R3hdm2
ILMN_1251989	9.73E-06	1.74E-09	22.36165	12.15304	1.549986	Ryr2
ILMN_1249578	0.000945	3.86E-05	7.23066	2.492347	1.545504	Sorl1
ILMN_1228568	3.57E-05	8.29E-08	14.67875	8.703047	1.543964	Irf2bp1
ILMN_2914416	3.53E-05	8.07E-08	14.72255	8.728831	1.543906	Ncstn
ILMN_1244402	0.00033	7.98E-06	8.742996	4.132462	1.539939	Kcnh2

ILMN_1252290	4.53E-05	1.51E-07	13.74009	8.12798	1.53352	Chd6
ILMN_1254199	0.008761	8.37E-04	4.81471	-0.73226	1.531487	Gaa
ILMN_3136056	0.000708	2.51E-05	7.622306	2.942075	1.531326	Atxn2
ILMN_1240471	0.000193	3.20E-06	9.727754	5.073102	1.528667	Retsat
ILMN_2990420	0.000121	1.39E-06	10.70616	5.923088	1.527964	Kdm4b
ILMN_3158565	3.77E-05	9.01E-08	14.54385	8.623055	1.527583	Plin5
ILMN_2666190	0.000845	3.28E-05	7.378534	2.66433	1.526567	Rnpepl1
ILMN_2698801	0.000912	3.68E-05	7.273752	2.542741	1.525416	Atg9a
ILMN_2445548	0.000562	1.78E-05	7.948531	3.302869	1.516718	C330006A16Rik
ILMN_2689887	0.000778	2.90E-05	7.489258	2.791365	1.515164	Mark2
ILMN_2688728	0.000129	1.54E-06	10.58375	5.820981	1.513891	Pcyt1a
ILMN_2955047	8.33E-05	6.50E-07	11.66858	6.687183	1.505148	Dsg2
ILMN_2615855	0.000216	3.84E-06	9.522772	4.884729	1.503073	Hnrnpa2b1
ILMN_2803138	0.002642	1.62E-04	6.028871	0.989995	1.501713	Prkaa2
ILMN_2736847	2.79E-05	4.20E-08	15.81718	9.345171	1.501327	Csdc2
ILMN_2628603	0.002042	1.14E-04	6.311583	1.360846	1.498897	Limch1
ILMN_1256171	0.00058	1.86E-05	7.903876	3.254202	1.495142	Tmem63b
ILMN_1213862	0.000418	1.15E-05	8.37427	3.755787	1.493815	Usp19
ILMN_2702704	8.02E-05	5.77E-07	11.82532	6.805473	1.493074	Ndufv1
ILMN_1242794	0.000752	2.77E-05	7.532504	2.840582	1.492997	Insr
ILMN_2832524	0.000299	6.78E-06	8.911672	4.300162	1.491526	Gpt
ILMN_2639972	0.000112	1.19E-06	10.89563	6.078862	1.489174	Ppp1r3b
ILMN_3160507	0.000109	1.13E-06	10.96175	6.132587	1.487622	Pm20d2
ILMN_2681474	0.009107	8.85E-04	4.776413	-0.79003	1.486206	Lmtk2
ILMN_1242039	0.000459	1.33E-05	8.229396	3.603882	1.485925	Med14
ILMN_1226195	0.001931	1.06E-04	6.368753	1.434496	1.48591	Bag3

ILMN_2690122	6.03E-05	3.10E-07	12.67688	7.42043	1.482031	Slc27a1
ILMN_2971314	0.000106	1.07E-06	11.03378	6.190748	1.478551	Dhdh
ILMN_2747507	0.008441	7.98E-04	4.847779	-0.68254	1.477656	Pitpnm2
ILMN_1225348	5.13E-05	2.11E-07	13.23673	7.800872	1.475571	Fam102a
ILMN_2795359	0.000117	1.31E-06	10.77392	5.979113	1.474451	Hdlbp
ILMN_2724235	0.002556	1.55E-04	6.066061	1.039415	1.474334	Myo18a
ILMN_2949021	4.56E-05	1.57E-07	13.67508	8.086498	1.472361	Nceh1
ILMN_2674351	1.56E-05	9.07E-09	18.70017	10.74367	1.469203	Ednra
ILMN_2460692	9.71E-05	8.91E-07	11.259	6.370146	1.468799	Bves
ILMN_1247682	0.003482	2.39E-04	5.727165	0.581838	1.468265	Atp6v0a1
ILMN_1247765	0.004492	3.40E-04	5.461309	0.211382	1.462052	Mfhas1
ILMN_1224079	0.03068	4.43E-03	3.716523	-2.4652	1.460728	Wfs1
ILMN_2992239	0.00027	5.60E-06	9.115082	4.498672	1.458597	Amd2
ILMN_2956932	0.000593	1.94E-05	7.865255	3.211928	1.458193	Acadvl
ILMN_2957987	0.000146	1.88E-06	10.34071	5.614757	1.457423	Perm1
ILMN_1257445	0.000358	9.14E-06	8.604099	3.992219	1.45736	Agl
ILMN_1229082	0.000498	1.49E-05	8.116572	3.48401	1.457171	Klhl21
ILMN_2694687	0.000272	5.71E-06	9.094364	4.478636	1.456556	Mypn
ILMN_3095624	0.001181	5.32E-05	6.949753	2.15815	1.455778	Kdm6b
ILMN_1243678	0.000547	1.71E-05	7.983689	3.341029	1.449147	Gtf3c1
ILMN_3135566	0.005897	4.99E-04	5.180315	-0.19128	1.447608	Tsc2
ILMN_1255832	0.00164	8.51E-05	6.550599	1.665814	1.446021	Irgq
ILMN_2777696	0.000149	1.97E-06	10.28875	5.570055	1.445902	Gcat
ILMN_2788836	0.026237	3.60E-03	3.846328	-2.25296	1.443716	Myh6
ILMN_2484322	0.000105	1.02E-06	11.08749	6.233865	1.442581	Ank3
ILMN_2782061	0.001695	8.86E-05	6.516997	1.623407	1.441771	Slc4a3

ILMN_2806676	0.000615	2.04E-05	7.818242	3.16024	1.440961	Acads
ILMN_1224093	2.87E-05	4.86E-08	15.56626	9.208503	1.440797	Llgl2
ILMN_2992232	0.001166	5.24E-05	6.962386	2.173394	1.439498	Mlxipl
ILMN_3004864	0.000423	1.17E-05	8.355739	3.736482	1.439199	Sun2
ILMN_1226540	0.000273	5.78E-06	9.080166	4.464881	1.438044	Agpat3
ILMN_2633212	9.03E-05	7.72E-07	11.44287	6.513914	1.431758	Cox10
ILMN_2864834	1.77E-05	1.31E-08	17.96396	10.41421	1.430868	Abcb8
ILMN_2618364	0.01462	1.67E-03	4.346248	-1.45289	1.429889	Flnc
ILMN_2674184	0.000365	9.40E-06	8.575734	3.963336	1.429645	Kcnb1
ILMN_2685985	0.003782	2.68E-04	5.641138	0.463079	1.428548	Megf8
ILMN_2765936	8.1E-05	6.02E-07	11.76861	6.762862	1.42648	Fam73b
ILMN_2622217	0.000919	3.73E-05	7.262129	2.529171	1.426204	Myh11
ILMN_2695900	1.26E-05	4.43E-09	20.20978	11.36783	1.425726	Kcnj12
ILMN_1238640	0.000272	5.74E-06	9.088294	4.472758	1.424105	Faap100
ILMN_2783519	0.000882	3.49E-05	7.322051	2.598954	1.422125	Acadl
ILMN_1224336	0.000634	2.14E-05	7.773289	3.110579	1.420751	Spsb1
ILMN_2966826	0.004796	3.76E-04	5.387733	0.107054	1.41621	Ttll1
ILMN_2706640	0.038384	5.94E-03	3.53264	-2.76846	1.413101	Cmya5
ILMN_2518744	0.00023	4.40E-06	9.374043	4.745679	1.412721	Wnk4
ILMN_3092653	0.044562	7.20E-03	3.413787	-2.96582	1.410293	Palld
ILMN_2826709	0.000104	9.76E-07	11.14364	6.278719	1.408941	Atad3a
ILMN_2628258	0.001351	6.41E-05	6.788786	1.962114	1.408837	Syvn1
ILMN_2927172	0.000104	9.84E-07	11.13385	6.270916	1.408458	Ppara
ILMN_2832620	0.002584	1.57E-04	6.052257	1.021094	1.405671	Rfx1
ILMN_2813547	0.001149	5.13E-05	6.980895	2.195691	1.402893	Scn4b
ILMN_2733179	5.48E-05	2.43E-07	13.02805	7.661167	1.401523	Aldh2

ILMN_1228288	0.00519	4.20E-04	5.306148	-0.00955	1.400023	Zbtb20
ILMN_2885532	0.000375	9.71E-06	8.542943	3.929843	1.398484	Cpt2
ILMN_2845214	0.000365	9.39E-06	8.576764	3.964387	1.398332	Lrpprc
ILMN_1254671	0.000244	4.79E-06	9.281674	4.658296	1.39777	BC034090
ILMN_2719069	0.000381	9.96E-06	8.516841	3.903103	1.390992	Maob
ILMN_2966104	2.63E-05	3.71E-08	16.03346	9.460867	1.388626	Htra1
ILMN_1222599	0.000127	1.51E-06	10.60382	5.837801	1.387183	Ubr4
ILMN_2798674	0.000219	3.95E-06	9.494039	4.858023	1.384871	Proser1
ILMN_1245489	0.003868	2.76E-04	5.617909	0.430828	1.384353	Tmem94
ILMN_1245815	0.000273	5.78E-06	9.08159	4.466262	1.383906	Ube2o
ILMN_2755727	0.000115	1.26E-06	10.82692	6.022685	1.383059	Filip1
ILMN_2509327	0.005315	4.33E-04	5.284448	-0.04072	1.382565	Wipf3
ILMN_2993221	0.000813	3.09E-05	7.432111	2.725984	1.375974	Slc4a4
ILMN_1250904	0.000445	1.26E-05	8.278761	3.655896	1.374187	Rtn2
ILMN_1257579	3.78E-05	9.62E-08	14.43888	8.560217	1.374186	Nup210
ILMN_2727329	8.08E-05	5.90E-07	11.79547	6.783071	1.373285	Bckdha
ILMN_1235186	7.63E-05	5.00E-07	12.01705	6.947944	1.372697	Cox15
ILMN_1232596	0.000137	1.67E-06	10.48462	5.737432	1.369721	Pom121
ILMN_1259606	0.00149	7.43E-05	6.664221	1.808093	1.369563	Abhd18
ILMN_1232621	0.000151	2.01E-06	10.26042	5.54559	1.369502	Ank
ILMN_2789294	2.87E-05	4.99E-08	15.52269	9.1845	1.368792	Tmem25
ILMN_1248181	0.003409	2.32E-04	5.750217	0.613481	1.36696	Zbtb7a
ILMN_2487847	0.000229	4.34E-06	9.388275	4.759073	1.366664	Uhrf1bp1
ILMN_2628488	3.84E-05	1.01E-07	14.36422	8.515196	1.365234	Pick1
ILMN_1237821	0.000333	8.16E-06	8.720356	4.109736	1.365118	Hectd1
ILMN_1257253	3.35E-05	7.26E-08	14.89395	8.828873	1.361806	Iqsec2

ILMN_3094043	0.000155	2.13E-06	10.19263	5.486787	1.359707	Paqr9
ILMN_3158250	0.003045	1.99E-04	5.866941	0.772539	1.358692	Cxcl12
ILMN_1257876	9.73E-06	2.02E-09	22.00202	12.02955	1.357472	B4gat1
ILMN_2760064	1.56E-05	8.55E-09	18.81984	10.7956	1.357274	Focad
ILMN_2630730	7.81E-05	5.32E-07	11.93466	6.887018	1.357073	Sf3a1
ILMN_2645526	0.000115	1.27E-06	10.81438	6.012395	1.355772	Abcc8
ILMN_2652518	0.000116	1.29E-06	10.79469	5.996212	1.354708	Atg2b
ILMN_1246992	0.000946	3.87E-05	7.229657	2.491171	1.353191	Hmgxb3
ILMN_1232237	0.001905	1.04E-04	6.386013	1.456644	1.35065	Ciapin1
ILMN_2685329	0.001139	5.07E-05	6.99085	2.207666	1.350323	Hspg2
ILMN_1216689	0.002388	1.41E-04	6.139088	1.135896	1.349319	Aplp2
ILMN_2664040	0.000201	3.38E-06	9.667139	5.017788	1.348129	Alas1
ILMN_3104271	0.000159	2.26E-06	10.12568	5.428331	1.347446	Abcc9
ILMN_2694659	7.38E-05	4.73E-07	12.09314	7.003826	1.345902	Ckap5
ILMN_2489448	0.000337	8.33E-06	8.698289	4.087537	1.343871	Sobp
ILMN_2879534	0.001076	4.71E-05	7.056054	2.285786	1.342357	Extl1
ILMN_2670778	0.001843	9.95E-05	6.421426	1.50196	1.341666	Kif1b
ILMN_2661005	9.03E-05	7.76E-07	11.43673	6.509156	1.340952	Arel1
ILMN_2486863	0.003287	2.22E-04	5.784144	0.659914	1.332046	Fam160b2
ILMN_2422333	1.51E-05	7.34E-09	19.13413	10.92991	1.330795	Trap1
ILMN_1216308	0.000455	1.31E-05	8.245894	3.621294	1.33049	Zfp574
ILMN_2684316	0.000484	1.43E-05	8.154911	3.5249	1.329181	P2ry1
ILMN_1250303	7.37E-05	4.65E-07	12.11551	7.020181	1.327607	C730029A08Rik
ILMN_2593496	0.00014	1.72E-06	10.44587	5.704561	1.325121	Got2
ILMN_1229556	0.000193	3.16E-06	9.743201	5.087145	1.325085	Ppp1r12b
ILMN_2501267	0.001222	5.57E-05	6.909618	2.109583	1.324885	Tmc7

ILMN_3053158	0.000231	4.42E-06	9.370675	4.742507	1.323844	Dyrk1b
ILMN_2511768	0.000155	2.13E-06	10.19224	5.486441	1.321957	Ttc17
ILMN_2595637	0.000111	1.17E-06	10.92105	6.09956	1.320006	Dnajc16
ILMN_3153940	0.000659	2.25E-05	7.725293	3.057301	1.31858	Unc45b
ILMN_1239535	0.001057	4.55E-05	7.085549	2.320947	1.31609	Plekhm2
ILMN_1250017	0.000586	1.90E-05	7.886143	3.234813	1.314884	Rptor
ILMN_2705991	0.000107	1.09E-06	11.001	6.164332	1.313431	Tpp1
ILMN_1244906	0.000355	9.03E-06	8.615808	4.004117	1.311639	Ap1b1
ILMN_1249888	0.046369	7.57E-03	3.383079	-3.01696	1.305566	Adcy6
ILMN_2633333	0.005875	4.97E-04	5.183829	-0.18617	1.304152	Brd4
ILMN_1254488	0.000193	3.19E-06	9.732173	5.077121	1.303751	Mgrn1
ILMN_2644161	9.96E-06	3.08E-09	21.02242	11.6778	1.299503	Acad12
ILMN_2517170	0.000636	2.15E-05	7.768279	3.105031	1.297405	Tuba4a
ILMN_1234471	0.000176	2.64E-06	9.945726	5.269361	1.29541	Cux2
ILMN_3129198	0.004666	3.61E-04	5.417689	0.149626	1.292901	Pgm5
ILMN_2694381	0.001535	7.75E-05	6.628242	1.763226	1.290993	Dnajc7
ILMN_1257020	4.38E-05	1.35E-07	13.90362	8.231351	1.289017	Aldh1b1
ILMN_2501357	2.44E-05	2.64E-08	16.64093	9.775804	1.28871	Dyrk1a
ILMN_1219915	0.000819	3.13E-05	7.421197	2.713453	1.288351	Dgat2
ILMN_2668261	0.000104	9.76E-07	11.14419	6.279153	1.284833	Slc26a6
ILMN_1234702	0.000697	2.44E-05	7.647195	2.970035	1.284105	Gpt2
ILMN_2933112	0.000162	2.36E-06	10.07627	5.384956	1.280907	Oat
ILMN_2641278	0.000462	1.34E-05	8.22229	3.596373	1.280242	Mfn2
ILMN_3107156	0.001221	5.56E-05	6.910432	2.110571	1.27963	Anxa11
ILMN_2679386	0.048469	8.01E-03	3.348285	-3.07497	1.276508	Gsn
ILMN_2680673	0.00134	6.35E-05	6.797529	1.972847	1.275524	Fign

ILMN_2475585	5.01E-05	1.82E-07	13.45105	7.941819	1.275196	Vdac1
ILMN_2859032	0.000454	1.30E-05	8.251642	3.627354	1.274682	Gfod1
ILMN_2716098	0.000123	1.43E-06	10.67083	5.893733	1.273816	Hsph1
ILMN_2718330	0.001695	8.86E-05	6.516637	1.622952	1.272991	Cish
ILMN_1251909	6.16E-05	3.18E-07	12.64146	7.395744	1.271922	Vars2
ILMN_3136542	0.000276	5.89E-06	9.061271	4.446547	1.27186	Gcdh
ILMN_2529395	0.000205	3.52E-06	9.621599	4.976016	1.270585	Fbxo10
ILMN_2993804	0.006483	5.68E-04	5.087802	-0.32635	1.267045	Ift81
ILMN_1249698	0.000433	1.21E-05	8.324814	3.704184	1.267018	Wbp2
ILMN_2881272	0.000288	6.39E-06	8.975207	4.362598	1.265819	Aifm1
ILMN_2669487	0.023422	3.11E-03	3.941144	-2.09901	1.265809	Kcnk3
ILMN_3036708	0.000522	1.60E-05	8.050652	3.413325	1.263877	Gatsl2
ILMN_2606667	0.002536	1.53E-04	6.074751	1.050936	1.263735	Ddx6
ILMN_3114641	0.002414	1.43E-04	6.127274	1.120338	1.262638	Pik3r1
ILMN_1258601	0.000782	2.92E-05	7.483442	2.784729	1.257856	Ywhag
ILMN_2757569	0.001381	6.63E-05	6.759751	1.926395	1.256793	Eno3
ILMN_1237323	8.62E-05	6.95E-07	11.58046	6.619954	1.25631	Lman2l
ILMN_2723639	0.000231	4.44E-06	9.364911	4.737075	1.255933	Pacs1
ILMN_2595091	5.13E-05	2.11E-07	13.23635	7.800618	1.254524	Nhlrc1
ILMN_2996683	0.000179	2.73E-06	9.906708	5.234531	1.254005	Nectin2
ILMN_2689274	0.024166	3.23E-03	3.915393	-2.14072	1.252684	Dync1h1
ILMN_1238982	0.035074	5.29E-03	3.604497	-2.64962	1.250601	Myom2
ILMN_1225056	9.73E-06	2.79E-09	21.24528	11.75986	1.246905	Slc5a6
ILMN_2763674	0.000285	6.24E-06	8.998956	4.385836	1.24476	Fto
ILMN_1237034	0.000217	3.89E-06	9.509384	4.872295	1.244566	AU040320
ILMN_2670517	0.000155	2.13E-06	10.19404	5.48801	1.243983	Fntb

ILMN_1246734	0.000155	2.08E-06	10.22012	5.510672	1.242155	Osbp2
ILMN_1260244	0.000396	1.05E-05	8.4591	3.843698	1.24046	Zfp777
ILMN_2936199	0.000176	2.64E-06	9.944222	5.268022	1.239721	Trim32
ILMN_2651035	0.000546	1.70E-05	7.991262	3.349229	1.238861	Kremen1
ILMN_2697499	0.000216	3.84E-06	9.523652	4.885546	1.237994	Igdcc4
ILMN_2666447	0.000267	5.50E-06	9.1345	4.517413	1.236038	Intu
ILMN_2963792	3.98E-05	1.14E-07	14.17561	8.400249	1.230902	Trmt11
ILMN_2669958	0.000587	1.91E-05	7.879454	3.22749	1.230209	Bcas3
ILMN_1231930	0.000104	9.97E-07	11.11655	6.257111	1.22947	Tesk1
ILMN_2698046	0.013349	1.47E-03	4.428464	-1.32426	1.229405	Stat3
ILMN_2948649	0.001578	8.07E-05	6.594801	1.721369	1.22829	Helz
ILMN_1223862	0.000304	6.97E-06	8.883013	4.271868	1.227787	Cux1
ILMN_2712685	0.000236	4.57E-06	9.333765	4.707673	1.226479	Phka1
ILMN_1255611	0.000243	4.74E-06	9.294599	4.670571	1.225534	Ppp1r3d
ILMN_1217353	0.000307	7.08E-06	8.867332	4.256352	1.224269	Rfx2
ILMN_1219208	0.000329	7.91E-06	8.751894	4.14138	1.223829	Nol6
ILMN_1229829	0.000102	9.58E-07	11.16688	6.297217	1.221185	Arid1a
ILMN_1237752	0.0001	9.35E-07	11.19793	6.321868	1.219024	Ubr2
ILMN_2517171	0.000488	1.44E-05	8.147658	3.517176	1.21888	Stk16
ILMN_2715201	0.000893	3.57E-05	7.301105	2.57461	1.216407	Golga2
ILMN_2659960	0.000807	3.05E-05	7.443557	2.73911	1.2155	Lrba
ILMN_2697174	2.63E-05	3.68E-08	16.0477	9.468413	1.215191	Trrap
ILMN_2491654	0.020802	2.66E-03	4.041063	-1.93783	1.214727	Ubap2
ILMN_2547737	0.000114	1.25E-06	10.83385	6.028365	1.214425	Daam1
ILMN_2678336	0.002532	1.53E-04	6.076267	1.052943	1.214347	Ctf1
ILMN_2740134	6.29E-05	3.34E-07	12.57359	7.348234	1.212278	Idh3a

ILMN_1250265	0.003409	2.32E-04	5.749678	0.612742	1.211253	Scn5a
ILMN_2686252	0.009107	8.85E-04	4.776395	-0.79006	1.211231	Ppip5k1
ILMN_2790246	6.29E-05	3.38E-07	12.55668	7.336357	1.210974	Herpud1
ILMN_2893321	5.13E-05	2.10E-07	13.23909	7.802432	1.210602	Fam185a
ILMN_2822915	0.000285	6.25E-06	8.997297	4.384214	1.208091	Lnpep
ILMN_1254786	0.00014	1.75E-06	10.42829	5.689612	1.20687	Ech1
ILMN_2609052	0.006541	5.75E-04	5.079614	-0.33837	1.205916	Dynll2
ILMN_2820948	0.00027	5.65E-06	9.10487	4.488801	1.204964	Dbt
ILMN_3161263	0.000709	2.52E-05	7.617865	2.937078	1.204964	Fzd5
ILMN_1257501	0.00014	1.76E-06	10.41982	5.682404	1.204636	Gatb
ILMN_2466164	0.001	4.18E-05	7.160205	2.409456	1.203156	Wfdc1
ILMN_2962958	2.97E-05	6.09E-08	15.18555	8.995995	1.20068	Nudc
ILMN_2737296	0.00011	1.15E-06	10.9374	6.112844	1.198547	Lars2
ILMN_2748384	0.000909	3.66E-05	7.27989	2.549901	1.197967	Zfp710
ILMN_2710678	0.001471	7.28E-05	6.680485	1.828319	1.19791	Nfic
ILMN_2926155	0.004248	3.15E-04	5.519094	0.292769	1.193862	Rbm38
ILMN_3158444	0.000107	1.08E-06	11.01253	6.173632	1.192697	Ank2
ILMN_1230048	0.000269	5.56E-06	9.121433	4.504805	1.192213	Fxr2
ILMN_2602204	0.000281	6.05E-06	9.033036	4.419085	1.192106	Tsc22d2
ILMN_2457727	7.71E-05	5.11E-07	11.98949	6.927611	1.190924	Adrb1
ILMN_1248465	0.000392	1.04E-05	8.476179	3.861306	1.190646	Zcchc3
ILMN_2750089	0.001195	5.39E-05	6.93711	2.142872	1.189924	Slc38a3
ILMN_2605453	0.000157	2.18E-06	10.16937	5.466514	1.189898	Ranbp10
ILMN_1227459	0.000681	2.37E-05	7.676103	3.002419	1.18958	Gga2
ILMN_2589525	0.000157	2.18E-06	10.1655	5.463143	1.189579	Cpeb3
ILMN_3153225	0.002138	1.21E-04	6.261102	1.295438	1.187317	Pomgnt2

ILMN_2742599	0.046182	7.53E-03	3.386217	-3.01173	1.187071	Nfix
ILMN_2987339	0.000288	6.35E-06	8.981538	4.368798	1.186908	Mybbp1a
ILMN_1219898	6.84E-05	4.02E-07	12.31633	7.165607	1.186221	Dnajc28
ILMN_2748837	0.002174	1.24E-04	6.240741	1.268959	1.185422	Aco2
ILMN_2667814	0.000396	1.06E-05	8.456495	3.84101	1.185005	Iars2
ILMN_2484679	0.001757	9.38E-05	6.470049	1.563902	1.181355	Gpcpd1
ILMN_1224664	0.012354	1.33E-03	4.497157	-1.21748	1.17992	Kdm2a
ILMN_3163481	8.62E-05	6.98E-07	11.57407	6.615057	1.178471	Trappc9
ILMN_1222009	8.1E-05	6.08E-07	11.75647	6.753711	1.174712	Gab1
ILMN_2780205	0.002503	1.50E-04	6.088295	1.068869	1.172291	Ctnna1
ILMN_2894355	0.002414	1.43E-04	6.127731	1.12094	1.171128	Prox1
ILMN_1255897	3.87E-05	1.05E-07	14.30623	8.480039	1.17086	Dip2c
ILMN_2950837	6.23E-05	3.26E-07	12.60964	7.373509	1.170067	Mfn1
ILMN_1216883	0.005153	4.15E-04	5.314052	0.001791	1.167278	Raver1
ILMN_3009685	0.000955	3.92E-05	7.218005	2.477504	1.166562	Ptprs
ILMN_2442914	6.42E-05	3.54E-07	12.49118	7.290172	1.165749	Lsm14b
ILMN_2931096	0.010345	1.04E-03	4.664048	-0.96073	1.162615	Igf2r
ILMN_1244272	0.00141	6.83E-05	6.734653	1.89543	1.159303	Epb41l3
ILMN_2424912	0.000852	3.31E-05	7.368836	2.653132	1.159083	Vamp2
ILMN_1255216	0.00019	3.07E-06	9.773133	5.1143	1.158358	Hsf1
ILMN_2833376	2.97E-05	6.00E-08	15.21049	9.010108	1.157735	Prkaca
ILMN_2624713	0.003264	2.20E-04	5.791718	0.670256	1.157244	Mast4
ILMN_2626359	0.00027	5.64E-06	9.106299	4.490183	1.156596	Cds2
ILMN_2760430	0.002248	1.30E-04	6.201156	1.217312	1.156504	Lrp10
ILMN_2494747	5.68E-05	2.65E-07	12.90387	7.57686	1.156221	Huwe1
ILMN_2810473	0.001149	5.13E-05	6.980478	2.195189	1.156072	Acadm

ILMN_2655025	0.006113	5.24E-04	5.145283	-0.24228	1.152179	Dennd4b
ILMN_1248384	7.92E-05	5.56E-07	11.87454	6.84228	1.151157	Ylpm1
ILMN_2524489	0.019115	2.38E-03	4.114252	-1.82049	1.150039	Afg3l2
ILMN_3150313	0.001544	7.83E-05	6.620276	1.753269	1.150003	Strip2
ILMN_2835461	0.000312	7.27E-06	8.839584	4.228837	1.147784	Noa1
ILMN_3148489	0.002802	1.77E-04	5.961153	0.899508	1.147645	Furin
ILMN_1242264	0.001924	1.06E-04	6.372552	1.439375	1.147317	Supt6
ILMN_2934533	0.013246	1.46E-03	4.436073	-1.3124	1.146887	Prpf19
ILMN_1241756	0.000155	2.13E-06	10.19354	5.487578	1.145569	Nfib
ILMN_2967622	0.000288	6.33E-06	8.985082	4.372267	1.144012	Gpsm1
ILMN_2447456	0.017086	2.07E-03	4.204873	-1.6761	1.143673	Kdm6a
ILMN_2657190	0.000705	2.49E-05	7.63164	2.952568	1.142855	Fbxo42
ILMN_2590321	0.002961	1.91E-04	5.89904	0.81594	1.142612	Nhlrc2
ILMN_1258158	0.002051	1.14E-04	6.306803	1.354667	1.141524	Aldh6a1
ILMN_2421751	0.000192	3.14E-06	9.747705	5.091237	1.14108	Kif1c
ILMN_2424605	0.000332	8.10E-06	8.727524	4.116937	1.140485	Txnrd2
ILMN_1223875	0.001068	4.63E-05	7.069596	2.301943	1.135161	Rasd2
ILMN_1222333	0.008931	8.60E-04	4.796188	-0.76017	1.13476	Slc2a4
ILMN_2562146	0.00377	2.67E-04	5.643981	0.46702	1.134483	Ankhd1
ILMN_2958484	0.000298	6.76E-06	8.915917	4.304345	1.132276	Nlrx1
ILMN_2421179	0.014743	1.69E-03	4.338965	-1.46432	1.13113	Rhobtb1
ILMN_2675674	0.00647	5.67E-04	5.089726	-0.32353	1.13037	Acsf2
ILMN_2466601	0.000277	5.91E-06	9.057321	4.44271	1.129803	Wdr7
ILMN_2722716	0.015329	1.78E-03	4.303093	-1.52075	1.129583	Atp1a2
ILMN_1217519	0.000302	6.90E-06	8.894653	4.283369	1.128586	Myh14
ILMN_1233008	0.000105	1.02E-06	11.09164	6.237189	1.128215	Dhx30

ILMN_2636666	0.001547	7.85E-05	6.617746	1.750104	1.127881	Prodh
ILMN_2796472	0.001073	4.67E-05	7.062521	2.293505	1.126716	Vldlr
ILMN_2792924	0.000514	1.55E-05	8.076944	3.441575	1.124952	Gstk1
ILMN_1252110	0.000391	1.04E-05	8.477695	3.862867	1.124883	Foxj2
ILMN_1255551	0.040619	6.39E-03	3.487218	-2.84377	1.123855	Sorbs1
ILMN_2599719	0.000691	2.42E-05	7.65701	2.981041	1.121007	Slc44a2
ILMN_1245678	0.00492	3.90E-04	5.360462	0.068186	1.120831	Abhd1
ILMN_2621901	0.000113	1.22E-06	10.86688	6.055398	1.120675	N4bp1
ILMN_2743780	0.000512	1.55E-05	8.080656	3.445557	1.120231	Msl2
ILMN_1230087	0.00073	2.64E-05	7.574922	2.888641	1.118315	Psme4
ILMN_2681824	4.78E-05	1.69E-07	13.56798	8.017668	1.118194	Fbxw7
ILMN_3023962	0.000347	8.74E-06	8.64941	4.038185	1.117997	Tnni3k
ILMN_1223268	0.000178	2.71E-06	9.916076	5.242905	1.117436	Bcl2l13
ILMN_1237234	5.12E-05	1.93E-07	13.36566	7.885964	1.116184	Tarsl2
ILMN_2597769	0.001049	4.51E-05	7.0943	2.331359	1.114865	Igf2
ILMN_2819530	0.00074	2.71E-05	7.553019	2.863852	1.11429	Rpap3
ILMN_3142789	0.000105	1.02E-06	11.08895	6.235038	1.113321	Abcb10
ILMN_2474111	4.53E-05	1.50E-07	13.74623	8.131883	1.113147	Arfgef2
ILMN_3002181	2.23E-05	2.06E-08	17.10518	10.00697	1.112657	Ppif
ILMN_1248254	0.000218	3.94E-06	9.496406	4.860226	1.112316	Gfm1
ILMN_2419138	0.000417	1.14E-05	8.382633	3.764488	1.11156	Vcl
ILMN_1247131	0.000211	3.71E-06	9.562924	4.921923	1.111111	Emc10
ILMN_2590896	0.000562	1.78E-05	7.94815	3.302455	1.110502	Slc25a13
ILMN_1217815	0.001709	8.97E-05	6.506762	1.610459	1.109169	Cacnb2
ILMN_1225300	0.000709	2.53E-05	7.616168	2.935168	1.107897	Slc25a29
ILMN_2844097	0.002738	1.71E-04	5.987554	0.934862	1.107809	Foxn3

ILMN_3064283	0.011398	1.19E-03	4.575493	-1.09649	1.107571	Pde4dip
ILMN_2783852	0.000185	2.92E-06	9.830901	5.166487	1.107336	Adgrl1
ILMN_1238648	8.07E-05	5.88E-07	11.80155	6.787638	1.107318	Tbc1d4
ILMN_2713898	0.012001	1.28E-03	4.524221	-1.17559	1.106979	Ddr1
ILMN_2433547	0.000117	1.32E-06	10.76658	5.973059	1.106771	Trp53bp1
ILMN_2679456	0.004536	3.46E-04	5.449171	0.194225	1.106769	Crat
ILMN_2698443	0.000304	6.96E-06	8.885194	4.274024	1.105892	Efnb1
ILMN_2503651	0.000317	7.48E-06	8.80931	4.19873	1.103465	Usp9x
ILMN_2663230	0.004291	3.20E-04	5.507284	0.276176	1.101447	Slco3a1
ILMN_3005211	0.028026	3.92E-03	3.7931	-2.33979	1.100861	Akap6
ILMN_2631591	0.000138	1.69E-06	10.47122	5.726079	1.100504	Nckap5
ILMN_1254154	0.024854	3.36E-03	3.891531	-2.17944	1.100028	Prrc2a
ILMN_2875938	9.13E-05	8.10E-07	11.38124	6.465996	1.097334	Pccb
ILMN_2491232	0.000537	1.66E-05	8.014117	3.373942	1.096534	Usp36
ILMN_1228970	5.48E-05	2.48E-07	12.99717	7.640287	1.095884	Rhobtb2
ILMN_2721874	0.000204	3.49E-06	9.630581	4.984269	1.094796	Arhgap20
ILMN_2804523	0.010406	1.05E-03	4.658374	-0.96939	1.094059	Dusp7
ILMN_1222492	0.000155	2.10E-06	10.20946	5.501416	1.091809	Mipep
ILMN_1252601	0.000745	2.73E-05	7.545663	2.855514	1.089941	Bcl7a
ILMN_2596499	0.001485	7.38E-05	6.669589	1.814773	1.086177	Ldoc1l
ILMN_1231066	0.002881	1.84E-04	5.927416	0.854185	1.086104	Hist2h2be
ILMN_2954474	0.000724	2.62E-05	7.583249	2.89805	1.086045	Enpp2
ILMN_2608174	0.001218	5.54E-05	6.914157	2.115086	1.085986	Tmem143
ILMN_1255644	0.033062	4.88E-03	3.655498	-2.56553	1.08589	Atn1
ILMN_2805372	0.000293	6.57E-06	8.944559	4.332529	1.084741	Itgb6
ILMN_2971721	0.006035	5.15E-04	5.158703	-0.22272	1.082261	Fbrsl1

ILMN_2627205	0.000225	4.25E-06	9.41308	4.782372	1.081051	Zmynd8
ILMN_1232447	5.13E-05	2.14E-07	13.21577	7.786946	1.080651	Por
ILMN_1226228	0.004445	3.36E-04	5.47132	0.225517	1.078697	Urgcp
ILMN_3115213	0.000255	5.10E-06	9.214611	4.594353	1.078602	Irx2
ILMN_1226688	0.000225	4.24E-06	9.416153	4.785254	1.078183	Ephx2
ILMN_2799351	0.000869	3.40E-05	7.345452	2.626086	1.075225	Acot11
ILMN_3124235	6.42E-05	3.56E-07	12.48619	7.286648	1.075126	Sall4
ILMN_2740020	0.027825	3.88E-03	3.799367	-2.32956	1.074457	Mapt
ILMN_2600465	0.00207	1.16E-04	6.29711	1.342129	1.072761	Phactr4
ILMN_1256518	0.000159	2.24E-06	10.13537	5.436817	1.070078	Akr7a5
ILMN_2970436	0.001372	6.56E-05	6.768755	1.937484	1.069763	Dnajb5
ILMN_2462448	0.000508	1.53E-05	8.092275	3.458012	1.069234	Tufm
ILMN_1256369	0.00014	1.75E-06	10.42433	5.686243	1.068773	Lynx1
ILMN_2666990	0.000412	1.11E-05	8.408825	3.791689	1.068227	Eef1a2
ILMN_2608666	0.000868	3.39E-05	7.347302	2.628229	1.067273	Dcaf6
ILMN_1225191	0.00047	1.38E-05	8.19302	3.565386	1.065081	Naprt
ILMN_2458442	0.00028	6.01E-06	9.039296	4.42518	1.064476	Usp15
ILMN_1212875	0.002654	1.63E-04	6.023218	0.982466	1.064134	Arhgef19
ILMN_2772582	0.007164	6.49E-04	4.992791	-0.46636	1.064	Smad3
ILMN_2973156	0.000208	3.61E-06	9.592175	4.948929	1.063303	Esrrg
ILMN_2624328	0.000636	2.15E-05	7.766345	3.102888	1.062624	Adamts7
ILMN_1218761	0.007458	6.82E-04	4.958104	-0.51779	1.062588	Lzts2
ILMN_2611022	0.000157	2.16E-06	10.17716	5.473308	1.062114	Bcl11b
ILMN_2791578	8.81E-05	7.18E-07	11.53827	6.587576	1.060948	Gspt1
ILMN_2846255	0.004624	3.56E-04	5.427468	0.163494	1.060293	Pgpep1
ILMN_2898319	0.00011	1.14E-06	10.9437	6.117954	1.059688	Aldh1l2

ILMN_2800687	0.003656	2.56E-04	5.676057	0.511412	1.058712	Hr
ILMN_2869524	0.000235	4.51E-06	9.34665	4.719848	1.058241	Coa7
ILMN_3156010	0.002085	1.17E-04	6.2894	1.332147	1.058054	Pdzrn3
ILMN_2699488	0.046377	7.58E-03	3.382799	-3.01743	1.057314	Lamb2
ILMN_2680406	0.003684	2.58E-04	5.668559	0.501048	1.055478	Rnf44
ILMN_1246777	0.009296	9.07E-04	4.758874	-0.81656	1.055434	Ptprm
ILMN_2658266	0.030735	4.44E-03	3.71494	-2.4678	1.05469	Gpr22
ILMN_2722081	0.002885	1.85E-04	5.925157	0.851145	1.054284	Clcn3
ILMN_2420353	9.03E-05	7.76E-07	11.4376	6.509831	1.05354	C230098O21Rik
ILMN_2596479	0.001425	6.97E-05	6.71817	1.87505	1.052256	Camk2a
ILMN_2757993	2.87E-05	5.07E-08	15.49424	9.168784	1.052224	Dlat
ILMN_1254409	0.026928	3.72E-03	3.825769	-2.28647	1.051952	Atxn1
ILMN_1227693	5.83E-05	2.78E-07	12.83652	7.530764	1.051396	Ints5
ILMN_2804622	0.005692	4.77E-04	5.214033	-0.14236	1.050926	Med25
ILMN_3107059	5.68E-05	2.61E-07	12.92644	7.592252	1.050917	Espn
ILMN_2876712	0.002107	1.19E-04	6.276517	1.315448	1.050881	Krba1
ILMN_2669146	4.61E-05	1.61E-07	13.63992	8.063967	1.049457	Synrg
ILMN_1227412	0.005753	4.84E-04	5.20358	-0.15751	1.048271	Ppip5k2
ILMN_1231586	0.000245	4.81E-06	9.277326	4.654163	1.048262	Sertad2
ILMN_2509052	3.32E-05	7.09E-08	14.9341	8.852114	1.047882	Zfp629
ILMN_2636200	0.003631	2.53E-04	5.683922	0.522274	1.046373	Syne1
ILMN_2509817	0.000219	3.97E-06	9.487394	4.851837	1.045466	Atp8a1
ILMN_2623844	0.00076	2.81E-05	7.518947	2.825177	1.045367	Pkp4
ILMN_1256673	0.000324	7.77E-06	8.770928	4.160429	1.04476	Uqcrc1
ILMN_3122427	0.002992	1.94E-04	5.885962	0.798275	1.044331	Suclg2
ILMN_2616709	0.029894	4.27E-03	3.739756	-2.4271	1.044193	Nisch

ILMN_2986051	4.46E-05	1.43E-07	13.8214	8.179549	1.043803	Kyat1
ILMN_1257880	0.048851	8.10E-03	3.341865	-3.08568	1.043234	Itga7
ILMN_1248190	5.1E-05	1.91E-07	13.37924	7.894878	1.043201	Mfsd7c
ILMN_1220834	0.009164	8.91E-04	4.77113	-0.79801	1.042659	Camta2
ILMN_3154640	0.000453	1.29E-05	8.256722	3.632707	1.04187	Aff1
ILMN_2466316	0.00141	6.83E-05	6.734713	1.895504	1.041086	Usp2
ILMN_2608933	0.000908	3.65E-05	7.281261	2.5515	1.040918	Tcof1
ILMN_2465338	0.006525	5.73E-04	5.08194	-0.33495	1.040458	Pigt
ILMN_1212672	0.000217	3.91E-06	9.503685	4.866997	1.040379	Elac2
ILMN_2643977	0.000179	2.73E-06	9.905984	5.233883	1.040234	Acat1
ILMN_1221298	7.81E-05	5.31E-07	11.938	6.889497	1.03924	Tuba8
ILMN_1251595	0.001471	7.29E-05	6.680398	1.828211	1.038173	Bhlhb9
ILMN_2764727	0.001761	9.41E-05	6.466857	1.559846	1.037327	Actn2
ILMN_2594768	0.000257	5.16E-06	9.202332	4.5826	1.036134	Tha1
ILMN_1244995	0.00045	1.28E-05	8.265463	3.641911	1.035856	LOC432459
ILMN_1221828	0.00762	7.01E-04	4.938877	-0.54638	1.034555	Wiz
ILMN_2756286	0.000219	3.96E-06	9.48932	4.85363	1.033987	Larp4b
ILMN_2728379	0.000262	5.32E-06	9.168245	4.549897	1.033724	Ivd
ILMN_1232998	0.000515	1.56E-05	8.072839	3.437169	1.033617	Zak
ILMN_2829935	7.81E-05	5.28E-07	11.94536	6.894958	1.033015	Dpysl4
ILMN_2762514	0.00094	3.83E-05	7.237744	2.500647	1.03249	Mier2
ILMN_1253596	0.003058	2.01E-04	5.860844	0.764279	1.032393	Pi4ka
ILMN_1255561	0.000307	7.11E-06	8.863388	4.252446	1.031978	Hspa5
ILMN_2669804	0.001062	4.59E-05	7.078371	2.3124	1.031887	Etfdh
ILMN_1245081	0.000193	3.21E-06	9.725352	5.070916	1.030901	Otud4
ILMN_1239726	0.001384	6.67E-05	6.755529	1.921191	1.03086	Snai3

ILMN_3146196	0.000614	2.03E-05	7.822021	3.164404	1.029301	Akap1
ILMN_2868133	0.000334	8.21E-06	8.713879	4.103226	1.028963	Gata6
ILMN_2627299	0.000493	1.47E-05	8.13247	3.500986	1.028611	Akap9
ILMN_2921698	0.00021	3.67E-06	9.57494	4.933026	1.028504	Pcca
ILMN_2672943	8.61E-05	6.92E-07	11.58533	6.623683	1.027215	Cntn2
ILMN_2447042	0.0003	6.82E-06	8.906656	4.295215	1.027103	Tnpo3
ILMN_2881681	0.00475	3.70E-04	5.399989	0.124487	1.02562	Tnrc6c
ILMN_1226555	0.000143	1.82E-06	10.3789	5.647473	1.025506	Inafm2
ILMN_2817864	0.00056	1.77E-05	7.953831	3.308631	1.025022	Ckmt2
ILMN_2675464	0.000091	7.94E-07	11.40757	6.486497	1.024173	Ankrd17
ILMN_2594855	0.001522	7.65E-05	6.639335	1.777078	1.024025	Pdpk1
ILMN_2939652	0.000142	1.79E-06	10.39788	5.663693	1.023859	Selenbp2
ILMN_2942669	0.00032	7.64E-06	8.788465	4.177948	1.023463	Atp13a1
ILMN_2654068	0.000283	6.19E-06	9.00823	4.394895	1.023048	Med23
ILMN_1230454	3.87E-05	1.03E-07	14.32809	8.49331	1.023045	Hadhb
ILMN_1222137	0.000107	1.09E-06	10.99956	6.163169	1.021848	Kmt2a
ILMN_2484527	0.001156	5.18E-05	6.972481	2.18556	1.020606	Vegfa
ILMN_2687032	0.001289	6.00E-05	6.845792	2.031922	1.019135	Fbxo31
ILMN_2943165	0.004087	2.96E-04	5.564882	0.356918	1.017888	Aqp7
ILMN_3010109	0.000224	4.16E-06	9.436287	4.804118	1.017149	Slc25a11
ILMN_3008068	0.014484	1.65E-03	4.353584	-1.44137	1.017006	Scara5
ILMN_2443129	0.000115	1.28E-06	10.80937	6.008283	1.015861	Usp10
ILMN_1227039	0.003252	2.19E-04	5.795959	0.676045	1.015456	Dlc1
ILMN_2509623	0.000587	1.91E-05	7.876988	3.224789	1.013015	Bptf
ILMN_2557496	0.000225	4.21E-06	9.422322	4.791038	1.012578	Ehd2
ILMN_2845080	0.019792	2.50E-03	4.081839	-1.87238	1.012515	Psap

ILMN_2991912	5.59E-05	2.56E-07	12.95435	7.611237	1.011229	Rab3a
ILMN_2797239	0.000159	2.26E-06	10.12501	5.427747	1.010848	Slc12a3
ILMN_2682574	0.000155	2.10E-06	10.2138	5.50519	1.009212	Pla2g5
ILMN_2447538	0.000552	1.73E-05	7.972471	3.328867	1.00889	Emsy
ILMN_3104414	0.001652	8.59E-05	6.542838	1.656032	1.008546	Etfb
ILMN_2800297	8.49E-05	6.79E-07	11.61078	6.643147	1.008426	Sart3
ILMN_2713841	4.46E-05	1.42E-07	13.83141	8.185877	1.00646	Hspd1
ILMN_1250816	3.87E-05	1.06E-07	14.2829	8.465849	1.005475	Apeh
ILMN_1230395	0.00012	1.37E-06	10.72115	5.935506	1.004239	C2cd2l
ILMN_2960407	0.002398	1.42E-04	6.134257	1.129536	1.003498	Mthfd1
ILMN_2616164	0.000283	6.17E-06	9.011252	4.397845	1.002263	Itga3
ILMN_1253259	0.00149	7.42E-05	6.664506	1.808448	1.002262	Snx27
ILMN_1237432	0.000659	2.25E-05	7.724648	3.056583	1.00172	Atg2a
ILMN_2913989	0.00014	1.76E-06	10.42251	5.68469	1.001679	Myzap
ILMN_1230422	0.000204	3.46E-06	9.638624	4.991653	1.001589	Ice1
ILMN_2416424	7.14E-05	4.36E-07	12.20257	7.08354	1.001383	Wbp1l
ILMN_2605201	0.002719	1.69E-04	5.995644	0.945676	1.000821	Klhl30
ILMN_3093150	0.000377	9.79E-06	-8.53472	3.921427	-1.00041	Npm3
ILMN_1229745	0.000282	6.09E-06	-9.02603	4.412259	-1.00133	Sertad4
ILMN_2777319	0.017086	2.07E-03	-4.20478	-1.67625	-1.00174	Stc1
ILMN_2654554	0.011465	1.20E-03	-4.56905	-1.10641	-1.00208	Fgl2
ILMN_1216042	0.00176	9.40E-05	-6.4679	1.561177	-1.00297	Apoe
ILMN_2739760	0.011764	1.24E-03	-4.54378	-1.14537	-1.00437	Prelp
ILMN_2515784	0.000434	1.22E-05	-8.31717	3.696181	-1.00468	Tspan6
ILMN_2501489	6.42E-05	3.50E-07	-12.5071	7.301415	-1.00653	Was
ILMN_2866327	0.033492	4.96E-03	-3.64545	-2.58209	-1.0071	Pkd2

ILMN_1220996	0.00099	4.12E-05	-7.17324	2.424838	-1.00725	Ptpn6
ILMN_2712075	0.03351	4.97E-03	-3.64436	-2.58387	-1.00808	Lcn2
ILMN_2805945	0.000111	1.18E-06	-10.9113	6.091626	-1.00828	Serp1
ILMN_2721357	0.004004	2.88E-04	-5.58585	0.386191	-1.00842	Lhfpl2
ILMN_1219213	0.010434	1.06E-03	-4.65448	-0.97534	-1.00948	Mfsd11
ILMN_2699898	0.014128	1.59E-03	-4.37635	-1.40569	-1.00985	Itgae
ILMN_2594971	0.00463	3.57E-04	-5.42588	0.161237	-1.01043	Rpl38
ILMN_1238314	0.004723	3.67E-04	-5.40515	0.131822	-1.01101	Serpinb1c
ILMN_1217143	0.041833	6.65E-03	-3.46326	-2.88355	-1.01312	Mbip
ILMN_1245733	0.001115	4.94E-05	-7.01406	2.235536	-1.01325	Ddx49
ILMN_1260262	0.001034	4.41E-05	-7.114	2.354764	-1.01391	Mrpl30
ILMN_1249498	0.01978	2.49E-03	-4.08278	-1.87087	-1.01528	Plek
ILMN_2719202	0.006669	5.90E-04	-5.06063	-0.36625	-1.01674	Hist1h2bf
ILMN_3161639	5.13E-05	1.96E-07	-13.3406	7.869479	-1.01681	Ssna1
ILMN_1256735	0.003232	2.16E-04	-5.80382	0.686771	-1.01735	Gusb
ILMN_2873822	0.010853	1.11E-03	-4.61929	-1.02921	-1.01889	Aebp1
ILMN_2724294	0.000397	1.06E-05	-8.45351	3.837933	-1.02035	Gpx7
ILMN_2596834	0.000684	2.39E-05	-7.66969	2.995238	-1.02038	Llph
ILMN_2694955	0.022941	3.02E-03	-3.9583	-2.07126	-1.02181	Igfbp4
ILMN_2654682	5.13E-05	2.22E-07	-13.1604	7.750036	-1.02184	Hspa1l
ILMN_2660263	0.014966	1.72E-03	-4.32496	-1.48634	-1.02219	Bcl2a1b
ILMN_1259294	0.004205	3.10E-04	-5.52955	0.307445	-1.02252	Tmem126b
ILMN_1247592	0.004792	3.75E-04	-5.3887	0.108424	-1.02341	Casp1
ILMN_2611295	0.012487	1.35E-03	-4.48724	-1.23285	-1.02423	Dusp26
ILMN_2755008	7.64E-05	5.03E-07	-12.0109	6.943424	-1.0256	Nfkbiz
ILMN_3161936	0.000883	3.50E-05	-7.31996	2.596525	-1.02564	Mpp1

ILMN_1232458	0.000403	1.08E-05	-8.43369	3.817445	-1.02609	C1qtnf2
ILMN_2633805	0.000204	3.48E-06	-9.63472	4.988067	-1.02773	Mrpl43
ILMN_1248099	0.005705	4.78E-04	-5.21175	-0.14567	-1.02807	Col16a1
ILMN_2853508	0.003477	2.38E-04	-5.7299	0.585602	-1.02835	Tmem208
ILMN_1222453	0.00857	8.14E-04	-4.83442	-0.7026	-1.03044	Apold1
ILMN_3001076	0.003808	2.70E-04	-5.63379	0.452886	-1.03159	Kmt5a
ILMN_1221274	0.002617	1.60E-04	-6.03926	1.003814	-1.0318	Rab32
ILMN_2636764	0.005574	4.63E-04	-5.23517	-0.11177	-1.03329	C1qtnf5
ILMN_1219786	0.010032	1.00E-03	-4.69038	-0.92057	-1.03463	Morn2
ILMN_2663585	0.027851	3.89E-03	-3.7983	-2.33129	-1.03537	Jdp2
ILMN_1242508	0.001039	4.44E-05	-7.10742	2.346951	-1.03681	Pin4
ILMN_2463181	0.019771	2.49E-03	-4.08334	-1.86998	-1.0372	Tnc
ILMN_2933618	0.000275	5.83E-06	-9.07066	4.455662	-1.03733	Gemin7
ILMN_2636116	0.005641	4.71E-04	-5.22315	-0.12915	-1.03734	Pdcd5
ILMN_2849359	0.000179	2.75E-06	-9.89725	5.22607	-1.03752	Fam96b
ILMN_2756665	0.000826	3.17E-05	-7.40771	2.697949	-1.03874	Cbr2
ILMN_2749052	0.000168	2.49E-06	-10.0153	5.331175	-1.03985	Ramp3
ILMN_2663130	0.001293	6.03E-05	-6.8419	2.027168	-1.04008	P2ry6
ILMN_2614486	0.000606	1.99E-05	-7.84173	3.186101	-1.0408	Slc50a1
ILMN_1377920	5.48E-05	2.48E-07	-13.0017	7.643334	-1.04116	Eef1a1
ILMN_2641678	0.000317	7.50E-06	-8.80752	4.196944	-1.04204	Bdh2
ILMN_2631663	0.000546	1.70E-05	-7.98986	3.347713	-1.04232	BC022687
ILMN_1254553	0.000565	1.79E-05	-7.94001	3.293603	-1.0426	Pole3
ILMN_3125606	8.26E-05	6.33E-07	-11.7029	6.713189	-1.04407	Ifi27
ILMN_1259470	0.00359	2.49E-04	-5.69613	0.539118	-1.04486	Tmem176b
ILMN_3136561	0.012604	1.37E-03	-4.47903	-1.2456	-1.04552	Sparc

ILMN_2834379	8.26E-05	6.33E-07	-11.7036	6.713771	-1.04586	Tgfbi
ILMN_2999762	0.00955	9.39E-04	-4.73534	-0.85222	-1.04614	Fmod
ILMN_2710185	0.001016	4.29E-05	-7.13719	2.382248	-1.04724	Myl12b
ILMN_2629455	0.000282	6.09E-06	-9.02499	4.411243	-1.04847	Ptprn
ILMN_2711267	0.010427	1.05E-03	-4.65521	-0.97423	-1.04995	Krt18
ILMN_1249366	0.00151	7.56E-05	-6.64978	1.790104	-1.05098	Bcl2
ILMN_2724942	0.00895	8.63E-04	-4.79381	-0.76376	-1.05123	Ptgis
ILMN_1252204	0.00014	1.72E-06	-10.4464	5.705054	-1.05209	Sepw1
ILMN_2452717	0.004655	3.59E-04	-5.42071	0.153908	-1.05235	Rab26os
ILMN_3091641	2.23E-05	2.16E-08	-17.0153	9.962833	-1.0526	Dab2
ILMN_2690976	0.000137	1.66E-06	-10.488	5.740316	-1.05303	Cdk4
ILMN_1213858	0.000799	3.00E-05	-7.45715	2.75468	-1.05419	Itgb3bp
ILMN_1215085	0.017345	2.10E-03	-4.19346	-1.69423	-1.05443	Fkbp10
ILMN_2424060	0.014627	1.67E-03	-4.34579	-1.4536	-1.05565	Ace
ILMN_3001616	0.004106	2.98E-04	-5.55899	0.348676	-1.05656	Plp2
ILMN_2766580	0.000458	1.32E-05	-8.23398	3.60872	-1.05681	Emc8
ILMN_2683831	0.001075	4.69E-05	-7.05932	2.289686	-1.05755	Mafk
ILMN_1232601	0.004822	3.79E-04	-5.38192	0.098778	-1.05841	Cyb561
ILMN_2702233	0.000913	3.69E-05	-7.27261	2.541405	-1.0621	Ccng1
ILMN_3066673	0.032191	4.72E-03	-3.67669	-2.53065	-1.06324	Gm14325
ILMN_2669766	0.040738	6.42E-03	-3.48474	-2.84788	-1.06362	Eid1
ILMN_2602406	0.000167	2.46E-06	-10.0267	5.341259	-1.06403	Ptpmt1
ILMN_2707616	0.003808	2.70E-04	-5.63422	0.453478	-1.06449	Col22a1
ILMN_1227972	0.001214	5.51E-05	-6.91933	2.121356	-1.06453	Rps16
ILMN_2759563	0.00039	1.03E-05	-8.4838	3.869153	-1.06492	Gatsl3
ILMN_2655595	0.001255	5.78E-05	-6.87747	2.070533	-1.06534	Crcp

ILMN_2694275	0.004143	3.02E-04	-5.54921	0.334989	-1.06537	Lxn
ILMN_1241496	0.000546	1.70E-05	-7.98867	3.346418	-1.06577	Egfr
ILMN_2681186	0.000498	1.49E-05	-8.11597	3.483363	-1.06626	Rab29
ILMN_1224855	0.000492	1.46E-05	-8.13567	3.504396	-1.06787	Samd9l
ILMN_1248811	0.041637	6.61E-03	-3.46694	-2.87744	-1.06812	Ctla2b
ILMN_2679077	0.001597	8.20E-05	-6.58099	1.704044	-1.06908	Nhp2
ILMN_2735961	1.56E-05	9.30E-09	-18.6491	10.72136	-1.06956	Tlr13
ILMN_1260348	0.000584	1.89E-05	-7.89097	3.240097	-1.07001	Srp9
ILMN_2910596	0.000155	2.09E-06	-10.2184	5.509151	-1.07158	Pold4
ILMN_1221936	0.000223	4.10E-06	-9.45224	4.819041	-1.07187	Cox19
ILMN_2661185	0.000206	3.53E-06	-9.61625	4.9711	-1.072	Scml4
ILMN_2608622	0.00016	2.30E-06	-10.1059	5.411009	-1.07205	B3gnt9
ILMN_1260512	0.002263	1.32E-04	-6.19459	1.20872	-1.07272	Mif
ILMN_1233840	0.014873	1.71E-03	-4.33012	-1.47822	-1.073	Kif1a
ILMN_2624385	0.001301	6.09E-05	-6.83318	2.016519	-1.07317	Use1
ILMN_2966632	0.006312	5.48E-04	-5.11393	-0.28807	-1.07364	Cyb5a
ILMN_2694884	0.012612	1.37E-03	-4.47832	-1.24671	-1.07364	Dnaic1
ILMN_2464381	0.000362	9.27E-06	-8.58986	3.977726	-1.07478	Ttc1
ILMN_2895000	0.000171	2.55E-06	-9.98345	5.30291	-1.07635	Slc35c2
ILMN_1215901	0.000219	3.97E-06	-9.48854	4.852902	-1.0768	Agpat2
ILMN_2861493	0.000819	3.12E-05	-7.42285	2.715349	-1.07767	Tceal8
ILMN_2840818	0.000467	1.36E-05	-8.20535	3.57845	-1.07817	Cmpk1
ILMN_1233188	0.000952	3.90E-05	-7.22289	2.483234	-1.07823	Snord123
ILMN_2959291	0.00576	4.85E-04	-5.20184	-0.16003	-1.07957	Upp1
ILMN_1239102	0.004611	3.54E-04	-5.43142	0.1691	-1.07998	H2-Eb1
ILMN_3089584	0.006554	5.77E-04	-5.07662	-0.34277	-1.08087	Cd74

ILMN_1247343	0.011679	1.23E-03	-4.5511	-1.13408	-1.08115	Lmcd1
ILMN_2950721	0.000335	8.27E-06	-8.70685	4.096157	-1.0828	Smim15
ILMN_2611510	0.002866	1.83E-04	-5.9335	0.862377	-1.08285	Med21
ILMN_2879588	2.92E-05	5.41E-08	-15.3854	9.108339	-1.08322	Lhfp
ILMN_1238868	5.13E-05	2.20E-07	-13.1718	7.75769	-1.08334	Gm5434
ILMN_3085992	0.009284	9.06E-04	-4.75996	-0.81492	-1.08434	Ifi202b
ILMN_2688681	0.003656	2.56E-04	-5.6754	0.510504	-1.0854	Npm3-ps1
ILMN_3103689	0.000709	2.53E-05	-7.6145	2.933295	-1.087	Spata24
ILMN_1243525	8.51E-05	6.83E-07	-11.6038	6.637833	-1.08915	Bloc1s6
ILMN_2675669	0.000269	5.56E-06	-9.12224	4.505583	-1.09064	Cd72
ILMN_1250771	0.000738	2.69E-05	-7.55721	2.868594	-1.09123	Prorsd1
ILMN_2669062	0.003457	2.37E-04	-5.73519	0.592866	-1.09369	Pi16
ILMN_1260378	0.000104	1.00E-06	-11.1091	6.251153	-1.09378	Csrp1
ILMN_2878071	9.66E-05	8.79E-07	-11.277	6.38436	-1.09471	Lyz1
ILMN_2742075	0.000458	1.32E-05	-8.23456	3.609333	-1.09545	Cd14
ILMN_3003130	0.000386	1.02E-05	-8.49701	3.882745	-1.09575	Rarres2
ILMN_2725414	0.001104	4.86E-05	-7.02767	2.251848	-1.09584	Cd9
ILMN_2681604	0.00419	3.09E-04	-5.53289	0.312134	-1.09662	Svbp
ILMN_2816137	0.00014	1.72E-06	-10.4475	5.705911	-1.09709	Ppp1r11
ILMN_2799596	0.000104	9.98E-07	-11.1155	6.256294	-1.09713	Vmac
ILMN_1230882	0.017141	2.08E-03	-4.20219	-1.68037	-1.09716	Pramel4
ILMN_2701991	0.021571	2.79E-03	-4.00992	-1.98795	-1.09723	Rcan1
ILMN_2890357	0.002928	1.88E-04	-5.91052	0.831429	-1.0976	Eva1b
ILMN_2866267	0.000531	1.63E-05	-8.03	3.391086	-1.09867	F2rl1
ILMN_2933914	0.001112	4.91E-05	-7.0188	2.241223	-1.09874	Psmd14
ILMN_2501191	9.03E-05	7.71E-07	-11.4449	6.515468	-1.09892	Sulf1

ILMN_2718189	4.65E-05	1.63E-07	-13.616	8.048604	-1.10002	Chn2
ILMN_2883392	0.001347	6.39E-05	-6.79201	1.966068	-1.10054	S100a11
ILMN_2776521	0.000231	4.42E-06	-9.36925	4.74116	-1.10237	Dpy30
ILMN_2614644	0.001783	9.56E-05	-6.45416	1.543702	-1.106	Pnisr
ILMN_2651643	0.000266	5.46E-06	-9.14165	4.524309	-1.10694	Naca
ILMN_2541190	8.1E-05	6.02E-07	-11.7691	6.763266	-1.11068	Dda1
ILMN_1232849	3.78E-05	9.68E-08	-14.4284	8.553914	-1.11125	Cd99
ILMN_1232901	0.000125	1.46E-06	-10.6428	5.87042	-1.11268	Vamp5
ILMN_2497657	0.00174	9.23E-05	-6.48319	1.580589	-1.11285	Xirp2
ILMN_2883164	0.000531	1.63E-05	-8.02961	3.390657	-1.11286	Serpine2
ILMN_2624280	8.1E-05	6.00E-07	-11.7747	6.767453	-1.114	Rps27l
ILMN_2971816	2.87E-05	4.88E-08	-15.5584	9.204165	-1.11471	Gltp
ILMN_2670727	0.000296	6.68E-06	-8.92823	4.316474	-1.11474	Gtf3a
ILMN_2712120	0.000223	4.12E-06	-9.44637	4.813553	-1.11711	S100a6
ILMN_2775030	0.000996	4.15E-05	-7.1665	2.416891	-1.11739	Folr2
ILMN_1248860	6.8E-05	3.92E-07	-12.3498	7.18963	-1.11795	Dpm3
ILMN_1247267	0.002268	1.32E-04	-6.1923	1.205733	-1.118	Smox
ILMN_2642517	0.000295	6.63E-06	-8.93599	4.324102	-1.11838	Medag
ILMN_1228535	0.001202	5.44E-05	-6.92934	2.133476	-1.11848	Pmp22
ILMN_1254516	0.001576	8.04E-05	-6.59806	1.725453	-1.12061	Ntan1
ILMN_2821676	0.003602	2.50E-04	-5.69241	0.533993	-1.12212	Nostrin
ILMN_2810882	0.000305	7.02E-06	-8.87579	4.264722	-1.12344	Ppic
ILMN_2971946	0.015457	1.80E-03	-4.29466	-1.53404	-1.12546	Lsm5
ILMN_2672190	0.006978	6.27E-04	-5.01804	-0.42902	-1.12618	Id1
ILMN_2850937	0.003278	2.21E-04	-5.78753	0.664532	-1.12831	Tmed3
ILMN_2790097	0.000731	2.65E-05	-7.57105	2.884264	-1.12892	Rhoc

ILMN_1219253	0.000632	2.13E-05	-7.77721	3.114917	-1.12935	Olfml1
ILMN_2647435	0.001383	6.65E-05	-6.75737	1.923456	-1.13107	Rassf1
ILMN_3070389	0.001605	8.27E-05	-6.57449	1.695875	-1.13115	Gm5796
ILMN_2769884	0.002218	1.28E-04	-6.21644	1.237281	-1.13155	Igf1
ILMN_3129497	0.000265	5.40E-06	-9.15405	4.536248	-1.1322	Nsmf
ILMN_2903379	0.003287	2.22E-04	-5.78381	0.65945	-1.13251	Memo1
ILMN_2944666	0.000466	1.36E-05	-8.20925	3.582578	-1.13346	Ifit3
ILMN_2822141	0.000514	1.56E-05	-8.07505	3.439547	-1.13387	Lyrm5
ILMN_1242221	0.000136	1.64E-06	-10.5065	5.755901	-1.13536	Ppp1r14b
ILMN_2792809	5.83E-05	2.76E-07	-12.8465	7.537585	-1.1356	Arpc5
ILMN_1247990	0.025682	3.51E-03	-3.86372	-2.22466	-1.13865	Purb
ILMN_3076439	0.001015	4.28E-05	-7.13913	2.384537	-1.13877	Slc1a2
ILMN_1249976	7.81E-05	5.43E-07	-11.9073	6.866679	-1.13938	Cfl2
ILMN_2634317	0.00033	8.02E-06	-8.73775	4.127201	-1.13958	Slc1a3
ILMN_2956942	0.000219	3.95E-06	-9.49265	4.856727	-1.13979	Prkcdbp
ILMN_1248108	0.002151	1.22E-04	-6.25222	1.283898	-1.14027	Lyplal1
ILMN_1253854	0.000606	1.99E-05	-7.84009	3.184289	-1.14083	Tgif1
ILMN_3135801	0.000396	1.06E-05	-8.45867	3.843251	-1.14085	Tmem237
ILMN_2929572	3.35E-05	7.33E-08	-14.8789	8.820132	-1.14099	Ccdc68
ILMN_1233809	0.004622	3.55E-04	-5.42875	0.165309	-1.14173	Cmtm7
ILMN_2715585	0.000257	5.14E-06	-9.20586	4.585975	-1.14227	Cyp20a1
ILMN_1258594	0.000622	2.07E-05	-7.80147	3.141738	-1.14325	Zfp560
ILMN_2642922	0.000382	9.99E-06	-8.51431	3.900503	-1.14343	Mtmr11
ILMN_2704641	4.56E-05	1.54E-07	-13.7103	8.109003	-1.14423	Dph3
ILMN_1236010	0.001478	7.33E-05	-6.67479	1.821236	-1.14556	Bicc1
ILMN_1226525	0.005398	4.42E-04	-5.26847	-0.06372	-1.14672	H2-Ab1

ILMN_1213989	0.007416	6.76E-04	-4.96411	-0.50888	-1.14723	Pmepa1
ILMN_2649199	0.016958	2.04E-03	-4.21208	-1.66466	-1.15054	Hpgd
ILMN_3163288	0.000193	3.17E-06	-9.73741	5.081887	-1.15552	Egfl7
ILMN_2617468	0.009555	9.40E-04	-4.73451	-0.85348	-1.15627	Chac1
ILMN_2629486	0.000143	1.84E-06	-10.3702	5.640072	-1.15685	Srpx
ILMN_1250752	0.028862	4.07E-03	-3.76924	-2.37881	-1.15804	Kif23
ILMN_2620804	0.00136	6.48E-05	-6.77947	1.950668	-1.15934	Iah1
ILMN_2901227	3.32E-05	7.11E-08	-14.9292	8.849264	-1.16144	Tmem5
ILMN_2619528	0.000455	1.31E-05	-8.24691	3.622363	-1.16153	Klhl6
ILMN_2914507	0.000912	3.68E-05	-7.27508	2.544287	-1.16187	Ndufb6
ILMN_2858106	0.018193	2.23E-03	-4.15413	-1.75683	-1.16215	Tpt1
ILMN_2751492	9.03E-05	7.69E-07	-11.4483	6.518096	-1.16302	Pgap2
ILMN_3130403	0.000141	1.77E-06	-10.4147	5.678075	-1.16374	Smagp
ILMN_2596611	1.77E-05	1.30E-08	-17.9809	10.42199	-1.16409	Clic1
ILMN_1243826	0.000335	8.22E-06	-8.71224	4.101578	-1.16595	Plekha4
ILMN_2454788	0.000628	2.11E-05	-7.78682	3.125552	-1.16671	Wbp1
ILMN_2429025	9.13E-05	8.21E-07	-11.3649	6.453214	-1.16763	Wbp5
ILMN_2705673	0.000157	2.17E-06	-10.1747	5.47117	-1.16784	Spg21
ILMN_3001540	0.001451	7.15E-05	-6.69641	1.848083	-1.16993	Lum
ILMN_2654754	0.000468	1.37E-05	-8.19957	3.572322	-1.17015	Pdpn
ILMN_2827780	0.000799	3.01E-05	-7.45657	2.754013	-1.17112	Phf11d
ILMN_3101732	7.21E-05	4.46E-07	-12.1734	7.062378	-1.17374	Syngr1
ILMN_2863837	0.000319	7.59E-06	-8.79448	4.183946	-1.17499	Alox5ap
ILMN_1233017	0.041096	6.50E-03	-3.47726	-2.86031	-1.17575	Klra9
ILMN_2484707	0.000826	3.18E-05	-7.40675	2.696838	-1.17587	Tyms
ILMN_2609323	0.000527	1.62E-05	-8.03915	3.400942	-1.17707	Lst1

ILMN_2478202	0.000147	1.91E-06	-10.3249	5.601204	-1.17809	Camta1
ILMN_1249934	0.004162	3.06E-04	-5.54156	0.324288	-1.17849	Gna13
ILMN_1256702	0.00324	2.17E-04	-5.80172	0.683901	-1.18221	S100a10
ILMN_2515363	0.000091	7.93E-07	-11.4092	6.487749	-1.185	Tpm4
ILMN_2684667	0.000122	1.41E-06	-10.6914	5.910845	-1.19035	Slirp
ILMN_2625451	0.001295	6.04E-05	-6.84051	2.02547	-1.19077	Ankrd1
ILMN_2752873	0.000209	3.65E-06	-9.5816	4.93917	-1.19213	Lgals9
ILMN_1250438	0.000616	2.05E-05	-7.81368	3.155209	-1.19319	Marcksl1
ILMN_2726837	0.003628	2.53E-04	-5.68515	0.523966	-1.1934	Nppb
ILMN_2733733	0.000251	4.97E-06	-9.24138	4.619925	-1.19373	Tlr2
ILMN_3002745	3.78E-05	9.19E-08	-14.5112	8.603566	-1.19573	Vkorc1
ILMN_2670508	5.99E-05	3.07E-07	-12.6926	7.431362	-1.19678	H2-M3
ILMN_2723718	0.000157	2.16E-06	-10.1789	5.474788	-1.1973	Rcn3
ILMN_2773624	0.000104	9.79E-07	-11.1405	6.276185	-1.19834	Hexa
ILMN_2757445	0.000301	6.87E-06	-8.89812	4.286792	-1.19848	Cep83
ILMN_2860750	0.004087	2.96E-04	-5.56474	0.356713	-1.20272	Snrnp27
ILMN_1259252	0.006121	5.25E-04	-5.14397	-0.24419	-1.20293	Anxa1
ILMN_2684874	0.00047	1.38E-05	-8.1924	3.564726	-1.20469	Timm10b
ILMN_2886618	0.000397	1.06E-05	-8.45517	3.839641	-1.20549	Nbl1
ILMN_3145814	2.87E-05	4.92E-08	-15.5457	9.1972	-1.2059	Ogfrl1
ILMN_2914938	7.16E-05	4.40E-07	-12.1926	7.076331	-1.20614	F13a1
ILMN_2939681	0.011679	1.23E-03	-4.55111	-1.13406	-1.20719	Lyz2
ILMN_2665008	5.68E-05	2.64E-07	-12.9075	7.579306	-1.20942	Rab31
ILMN_2787172	0.011842	1.26E-03	-4.53632	-1.15689	-1.20972	Tpm2
ILMN_2909248	9.13E-05	8.04E-07	-11.3914	6.473929	-1.20977	Vgll2
ILMN_1234223	0.000159	2.25E-06	-10.1287	5.430975	-1.21087	Pld4

ILMN_2516348	0.000844	3.26E-05	-7.38342	2.669963	-1.21109	Vamp4
ILMN_2711461	0.001478	7.33E-05	-6.67477	1.821211	-1.21132	Tgfb1
ILMN_2748966	0.000666	2.28E-05	-7.71093	3.041304	-1.2146	Tgfb3
ILMN_2730005	0.001019	4.31E-05	-7.13417	2.378674	-1.21531	Rpl29
ILMN_2942989	5.13E-05	2.19E-07	-13.1816	7.764214	-1.21661	Mtpn
ILMN_1225261	0.000206	3.55E-06	-9.60983	4.96519	-1.22078	Uchl1
ILMN_2513826	0.000676	2.34E-05	-7.68708	3.01469	-1.22325	Tnfrsf11b
ILMN_2644719	0.002475	1.48E-04	-6.09979	1.084065	-1.2273	Hmgn3
ILMN_1249242	0.00028	5.99E-06	-9.04339	4.429164	-1.23229	Dnajc2
ILMN_3160770	0.000207	3.57E-06	-9.60482	4.96058	-1.23292	Cd200
ILMN_1256285	0.007458	6.82E-04	-4.95804	-0.51789	-1.23369	St6galnac2
ILMN_1242700	0.001367	6.53E-05	-6.77297	1.94267	-1.23549	Spcs3
ILMN_2964042	0.025315	3.44E-03	-3.87608	-2.20456	-1.23763	Bgn
ILMN_1251341	0.025504	3.47E-03	-3.8699	-2.21461	-1.24221	Ndufc2
ILMN_1214488	0.00126	5.81E-05	-6.87309	2.065205	-1.2424	Pebp1
ILMN_2675811	0.002317	1.35E-04	-6.1711	1.177957	-1.24424	Slc11a1
ILMN_2638114	0.00305	2.00E-04	-5.86486	0.76972	-1.24677	Ptn
ILMN_2695228	0.000446	1.27E-05	-8.27598	3.652971	-1.24745	Fam220a
ILMN_1239380	0.000396	1.06E-05	-8.45865	3.843231	-1.24758	P3h3
ILMN_2592823	0.000466	1.36E-05	-8.21001	3.583387	-1.24896	Cdc42ep5
ILMN_2776431	0.000149	1.99E-06	-10.2739	5.557277	-1.25079	C1qa
ILMN_1227814	0.000859	3.35E-05	-7.35929	2.642101	-1.25154	Srr
ILMN_1246861	0.027623	3.85E-03	-3.80497	-2.32041	-1.25229	Ctss
ILMN_2422982	0.010139	1.02E-03	-4.68042	-0.93574	-1.25484	Synpo
ILMN_2777270	0.004237	3.14E-04	-5.52192	0.296741	-1.25712	Pfdn4
ILMN_2798138	0.000357	9.09E-06	-8.60907	3.997272	-1.25971	Gipc2

ILMN_2822000	0.004252	3.16E-04	-5.51702	0.289857	-1.26059	Slc10a6
ILMN_2685516	0.000587	1.91E-05	-7.87662	3.224391	-1.26274	Hcls1
ILMN_1249289	0.000196	3.26E-06	-9.70757	5.054715	-1.26316	Tspan12
ILMN_2660555	0.002189	1.26E-04	-6.23077	1.255976	-1.26522	Bcl2a1d
ILMN_1232716	0.001578	8.06E-05	-6.5959	1.72275	-1.26692	Cyp1b1
ILMN_1216133	0.000988	4.11E-05	-7.17558	2.427602	-1.2692	Igsf1
ILMN_2604282	0.030238	4.34E-03	-3.72944	-2.44402	-1.26929	Sfrp1
ILMN_2749669	0.039277	6.12E-03	-3.51407	-2.79923	-1.27123	Nr1d1
ILMN_2688966	0.000742	2.71E-05	-7.55065	2.861167	-1.27184	Col8a2
ILMN_2806700	0.000148	1.93E-06	-10.3094	5.58786	-1.27357	Cyba
ILMN_2999439	0.000434	1.22E-05	-8.31634	3.695317	-1.27454	Klf4
ILMN_1233545	0.013349	1.48E-03	-4.42838	-1.32439	-1.27469	Lbh
ILMN_2947568	0.000777	2.89E-05	-7.49393	2.796695	-1.27494	Gadd45a
ILMN_3107690	0.000353	8.97E-06	-8.62292	4.011335	-1.27508	Fam177a
ILMN_2706730	0.025182	3.41E-03	-3.88098	-2.19659	-1.27699	Ptprr
ILMN_1245307	0.005749	4.83E-04	-5.20428	-0.15649	-1.27817	Fbln2
ILMN_1219002	0.002522	1.52E-04	-6.08097	1.059172	-1.28066	Usmg5
ILMN_2986458	0.000871	3.42E-05	-7.34091	2.620829	-1.28665	Terf2ip
ILMN_2543929	0.00091	3.66E-05	-7.27826	2.547996	-1.28679	Cenpw
ILMN_1257631	6.23E-05	3.26E-07	-12.6058	7.370835	-1.28722	Apobec1
ILMN_2998976	0.00016	2.30E-06	-10.1048	5.409988	-1.28849	Lpar1
ILMN_1259400	4.56E-05	1.56E-07	-13.6853	8.093049	-1.29051	Prr33
ILMN_2710819	5.48E-05	2.46E-07	-13.0115	7.649972	-1.29121	Csf1r
ILMN_2794645	0.003617	2.52E-04	-5.68832	0.528348	-1.29214	Cyr61
ILMN_2757224	0.005951	5.05E-04	-5.17207	-0.20327	-1.29216	Srp19
ILMN_2636212	0.001693	8.84E-05	-6.51827	1.625016	-1.29356	Sh3bgrl

ILMN_3049559	0.000598	1.96E-05	-7.854	3.199572	-1.2948	C4b
ILMN_2904686	0.006146	5.29E-04	-5.13956	-0.25062	-1.29673	Cyb5r3
ILMN_2757428	7.92E-05	5.55E-07	-11.8773	6.8443	-1.2999	Bloc1s2
ILMN_1260025	0.001374	6.58E-05	-6.76631	1.934476	-1.30018	Map3k7cl
ILMN_2672597	4.93E-05	1.79E-07	-13.4828	7.96252	-1.30063	Gng10
ILMN_2443330	0.000569	1.81E-05	-7.92884	3.28144	-1.30112	Ttr
ILMN_2748875	5.48E-05	2.44E-07	-13.0206	7.656139	-1.30114	Fcer1g
ILMN_1229340	0.010584	1.08E-03	-4.64173	-0.99484	-1.30193	Triqk
ILMN_2811737	0.000321	7.66E-06	-8.78514	4.174628	-1.30666	Casp4
ILMN_2743244	0.003172	2.11E-04	-5.82282	0.712644	-1.30676	Cd86
ILMN_2642913	0.000251	4.98E-06	-9.24067	4.619247	-1.30901	Emp1
ILMN_2700848	0.000113	1.22E-06	-10.8689	6.057084	-1.31499	Arrb2
ILMN_2803399	0.000112	1.19E-06	-10.8977	6.080536	-1.31816	Mcm6
ILMN_2946088	0.001975	1.09E-04	-6.34563	1.404758	-1.31835	Panx1
ILMN_2921303	0.000431	1.20E-05	-8.3309	3.710548	-1.31955	Tyms-ps
ILMN_2795078	4.56E-05	1.55E-07	-13.7009	8.103026	-1.32261	Unc93b1
ILMN_2675874	0.001661	8.64E-05	-6.53798	1.649905	-1.32587	Alas2
ILMN_2598775	4.46E-05	1.42E-07	-13.8281	8.183788	-1.32614	Stard3nl
ILMN_2451022	0.002967	1.92E-04	-5.89602	0.811859	-1.32858	Vim
ILMN_1249032	0.000225	4.20E-06	-9.42613	4.794602	-1.33399	Mzt1
ILMN_3117381	0.000345	8.66E-06	-8.65944	4.048336	-1.33845	Fhl1
ILMN_2771979	0.001376	6.60E-05	-6.76471	1.932499	-1.34888	Dio2
ILMN_2543417	0.000807	3.05E-05	-7.44361	2.739172	-1.35125	Auh
ILMN_2771237	0.001757	9.37E-05	-6.47037	1.56431	-1.36091	Lbp
ILMN_2974343	5.83E-05	2.78E-07	-12.8346	7.529435	-1.36313	Pla1a
ILMN_2687403	5.13E-05	2.17E-07	-13.1957	7.773596	-1.36605	Fcgr3

ILMN_2625125	0.000185	2.93E-06	-9.82822	5.164074	-1.36714	Cx3cr1
ILMN_2707834	7.25E-05	4.55E-07	-12.1467	7.042949	-1.36735	Rpl13a
ILMN_2667463	0.000467	1.37E-05	-8.20327	3.576252	-1.36843	Ocel1
ILMN_2854157	0.000327	7.87E-06	-8.75699	4.146482	-1.36999	Aqp8
ILMN_2720083	0.000119	1.35E-06	-10.738	5.949435	-1.37111	Bace2
ILMN_2718204	9.03E-05	7.66E-07	-11.4534	6.522077	-1.37143	Clec11a
ILMN_1247377	0.002065	1.15E-04	-6.30006	1.345948	-1.38238	Mpeg1
ILMN_2444316	0.000414	1.12E-05	-8.39681	3.779217	-1.38284	Tpm3
ILMN_2657478	0.012049	1.28E-03	-4.52124	-1.18019	-1.38454	Cd53
ILMN_1253741	0.016764	2.02E-03	-4.22146	-1.64979	-1.38503	Pcolce
ILMN_2987984	0.000731	2.66E-05	-7.56857	2.88146	-1.38882	S100a13
ILMN_1215252	5.87E-05	2.98E-07	-12.7343	7.460254	-1.38953	Bmp4
ILMN_1258770	0.000468	1.37E-05	-8.2016	3.574481	-1.39167	Lrrc51
ILMN_2806159	0.000494	1.47E-05	-8.12767	3.495867	-1.39711	Tmsb4x
ILMN_2792601	0.001457	7.19E-05	-6.69171	1.842259	-1.39823	Lpar6
ILMN_2693403	0.000665	2.27E-05	-7.71445	3.045224	-1.39907	Cela1
ILMN_2950622	3.99E-05	1.18E-07	-14.1164	8.363813	-1.39912	Arhgdig
ILMN_2544890	0.000851	3.31E-05	-7.36941	2.653793	-1.39976	Pde4b
ILMN_2787257	0.000179	2.72E-06	-9.91083	5.238216	-1.40099	Coro1a
ILMN_1227907	0.006081	5.20E-04	-5.15061	-0.23452	-1.40116	Gmfg
ILMN_1251071	0.000301	6.86E-06	-8.89992	4.288574	-1.402	Ypel5
ILMN_2885277	0.000688	2.41E-05	-7.66225	2.986911	-1.40405	Nnmt
ILMN_2795412	0.000179	2.75E-06	-9.90078	5.229231	-1.40603	Tmem176a
ILMN_1217235	0.001543	7.81E-05	-6.62247	1.756009	-1.40752	Fam114a1
ILMN_2915232	0.000334	8.17E-06	-8.71871	4.108085	-1.4105	Cotl1
ILMN_2857595	9.42E-05	8.49E-07	-11.321	6.418898	-1.41486	AI413582

ILMN_2681913	0.028864	4.07E-03	-3.76903	-2.37915	-1.42446	Crct1
ILMN_2440530	0.025175	3.41E-03	-3.88147	-2.19579	-1.42519	Vat1
ILMN_2993314	0.000313	7.30E-06	-8.8357	4.224984	-1.427	Clec4n
ILMN_1245354	0.002617	1.60E-04	-6.03907	1.003565	-1.42861	Tlr7
ILMN_2671923	0.005252	4.26E-04	-5.29506	-0.02547	-1.42896	Ly86
ILMN_2493826	0.000121	1.39E-06	-10.7082	5.924804	-1.42939	Ugt1a10
ILMN_2712892	0.030384	4.37E-03	-3.72506	-2.4512	-1.42952	Mir99ahg
ILMN_2829330	0.000229	4.34E-06	-9.38878	4.759546	-1.43079	Hexb
ILMN_2741117	0.000167	2.48E-06	-10.0196	5.334968	-1.43193	Ankrd23
ILMN_1239806	0.000777	2.89E-05	-7.49332	2.795994	-1.43239	Rpa3
ILMN_2984744	0.000314	7.36E-06	-8.82682	4.216155	-1.43291	Emp3
ILMN_2788121	7.71E-05	5.14E-07	-11.9818	6.921921	-1.43412	Dynll1
ILMN_2687169	8.36E-05	6.56E-07	-11.6567	6.678154	-1.44183	Id3
ILMN_1250213	0.000109	1.14E-06	-10.952	6.124693	-1.44271	Sh3glb1
ILMN_1258691	0.00029	6.45E-06	-8.96536	4.352949	-1.44459	Pdzd3
ILMN_2677712	8.49E-05	6.75E-07	-11.6192	6.649611	-1.44841	Arf5
ILMN_2847144	0.002189	1.26E-04	-6.23145	1.256853	-1.46081	Hist1h2ak
ILMN_1256676	0.021509	2.78E-03	-4.01242	-1.98392	-1.46455	Ddah1
ILMN_2631518	0.002107	1.19E-04	-6.27707	1.31616	-1.46623	Serf1
ILMN_1258629	0.00193	1.06E-04	-6.36937	1.435283	-1.46696	Col3a1
ILMN_1228631	0.000638	2.16E-05	-7.76384	3.100112	-1.46782	Nkd2
ILMN_2479977	7.98E-05	5.69E-07	-11.8442	6.819585	-1.46802	Enho
ILMN_2795040	0.001075	4.69E-05	-7.05996	2.290448	-1.47405	Hist1h2ad
ILMN_2904641	1.43E-05	6.15E-09	-19.5055	11.08481	-1.47678	Snora65
ILMN_2650115	0.001423	6.94E-05	-6.72146	1.879124	-1.47715	Adamtsl2
ILMN_2970879	1.77E-05	1.30E-08	-17.9863	10.42446	-1.47876	Uchl3

ILMN_2621021	0.001536	7.76E-05	-6.62756	1.762371	-1.48053	Ptpn14
ILMN_2867147	0.00019	3.09E-06	-9.76686	5.10862	-1.48177	Tyrobp
ILMN_2860480	3.98E-05	1.14E-07	-14.1658	8.394205	-1.48347	Arpc1b
ILMN_2588051	0.003852	2.74E-04	-5.62223	0.436835	-1.48481	Tubb2b
ILMN_2733356	7.38E-05	4.74E-07	-12.0901	7.001575	-1.48523	Endod1
ILMN_2903926	5.87E-05	2.89E-07	-12.7764	7.489391	-1.48541	Pdgfrl
ILMN_2650739	0.000526	1.61E-05	-8.04218	3.404203	-1.48669	Arhgap9
ILMN_2838317	0.000236	4.57E-06	-9.3329	4.706854	-1.49411	Pqlc3
ILMN_1246173	0.00021	3.69E-06	-9.5696	4.928089	-1.49496	Msx1
ILMN_1239430	0.000152	2.04E-06	-10.2423	5.529936	-1.49646	Mrc1
ILMN_2880906	8.36E-05	6.55E-07	-11.6587	6.679685	-1.49733	Pdlim2
ILMN_2702303	0.002675	1.65E-04	-6.01372	0.969812	-1.49858	Ch25h
ILMN_2864309	0.0108	1.10E-03	-4.62377	-1.02234	-1.50501	Wfdc17
ILMN_2687586	8.45E-05	6.65E-07	-11.6387	6.664484	-1.50536	Cxcl16
ILMN_2789948	2.97E-05	6.21E-08	-15.1515	8.976676	-1.5156	Arhgdib
ILMN_1246056	0.00035	8.83E-06	-8.63926	4.027903	-1.51563	Gngt2
ILMN_2609614	6.66E-05	3.83E-07	-12.3842	7.214214	-1.51571	Snx10
ILMN_1239181	0.001156	5.18E-05	-6.97299	2.186178	-1.51662	Nmral1
ILMN_2898924	0.001022	4.33E-05	-7.12832	2.371738	-1.51739	Kcnj14
ILMN_1241695	0.00039	1.03E-05	-8.4837	3.869053	-1.52018	Ms4a6d
ILMN_1229683	0.009107	8.85E-04	-4.77624	-0.79029	-1.52176	Gm9961
ILMN_1234812	0.000429	1.19E-05	-8.33543	3.715281	-1.52269	Dap
ILMN_2730329	0.001789	9.59E-05	-6.45094	1.539602	-1.53094	Hist1h2ah
ILMN_2609813	0.00149	7.43E-05	-6.66434	1.808238	-1.53126	Chil1
ILMN_2879382	0.000291	6.51E-06	-8.95543	4.343206	-1.53937	Med10
ILMN_1231506	7.38E-05	4.72E-07	-12.0966	7.006379	-1.54099	Cnrip1

ILMN_2757966	0.000262	5.31E-06	-9.17153	4.553055	-1.5549	Pf4
ILMN_1214602	0.000183	2.84E-06	-9.86073	5.193323	-1.55836	Sfrp2
ILMN_1252506	2.63E-05	3.59E-08	-16.0935	9.492648	-1.55944	Fbn1
ILMN_1252757	0.000918	3.72E-05	-7.26402	2.531376	-1.55997	Mllt3
ILMN_2609809	0.000257	5.15E-06	-9.204	4.584195	-1.56246	Sec61b
ILMN_2504823	0.001744	9.27E-05	-6.47912	1.575416	-1.56271	Trim47
ILMN_1251669	0.003052	2.00E-04	-5.86381	0.768299	-1.56642	Evi2a
ILMN_2847787	0.000835	3.21E-05	-7.39601	2.684477	-1.57416	Adgre1
ILMN_2630605	0.032077	4.69E-03	-3.6797	-2.5257	-1.57483	Fscn1
ILMN_2877069	0.000248	4.88E-06	-9.26315	4.640675	-1.58198	Tspo
ILMN_2880536	0.000546	1.70E-05	-7.9891	3.346889	-1.59426	Uck2
ILMN_2762944	0.001747	9.30E-05	-6.47696	1.572679	-1.59715	Ifi27l2a
ILMN_1247691	4.93E-05	1.78E-07	-13.4859	7.964529	-1.60878	Hes1
ILMN_2983624	2.23E-05	2.06E-08	-17.0973	10.00313	-1.61051	Ackr4
ILMN_2609998	3.87E-05	1.07E-07	-14.2723	8.459411	-1.6125	Fxyd6
ILMN_1246800	0.037895	5.85E-03	-3.54273	-2.75174	-1.61991	Serpina3n
ILMN_2821460	0.00338	2.30E-04	-5.75775	0.623806	-1.62144	Ptpn18
ILMN_1219574	0.003606	2.51E-04	-5.69136	0.532545	-1.62621	Hist1h2af
ILMN_2909150	0.007959	7.40E-04	-4.90059	-0.60346	-1.62865	Ctgf
ILMN_2946466	0.000106	1.04E-06	-11.0613	6.212842	-1.6299	Bin1
ILMN_2771766	0.013759	1.53E-03	-4.40185	-1.3658	-1.63199	Ccl12
ILMN_2923607	0.000225	4.22E-06	-9.41919	4.788102	-1.63372	Phlda3
ILMN_2804487	0.000159	2.25E-06	-10.1331	5.434812	-1.64083	Aif1
ILMN_3091003	0.001165	5.23E-05	-6.96425	2.175644	-1.64225	Ms4a7
ILMN_2625351	6.29E-05	3.37E-07	-12.5611	7.339498	-1.64682	Sh3bgrl3
ILMN_2699531	0.000929	3.77E-05	-7.25138	2.516605	-1.64992	Rgs10

ILMN_2992709	2.54E-05	2.93E-08	-16.4567	9.681815	-1.66219	Trem2
ILMN_2666018	0.000139	1.70E-06	-10.4628	5.71892	-1.67826	Mgp
ILMN_2837802	0.00037	9.58E-06	-8.55611	3.943304	-1.69343	BC028528
ILMN_1225035	2.97E-05	5.92E-08	-15.2322	9.022347	-1.69863	Carhsp1
ILMN_1248830	0.001242	5.69E-05	-6.89121	2.087238	-1.70139	Hist1h2an
ILMN_2910934	0.00087	3.41E-05	-7.34196	2.622044	-1.70786	Cd52
ILMN_3145331	0.000182	2.82E-06	-9.86906	5.200804	-1.70894	Tmsb10
ILMN_2836654	0.002378	1.40E-04	-6.14442	1.142917	-1.71016	Hist1h2ap
ILMN_2869110	0.000264	5.36E-06	-9.16051	4.542459	-1.71471	Comp
ILMN_1237364	2.23E-05	2.23E-08	-16.9514	9.931298	-1.71979	Mmp23
ILMN_2702193	3.49E-05	7.86E-08	-14.7653	8.75391	-1.72206	Mxra7
ILMN_1232884	0.000118	1.34E-06	-10.7517	5.960802	-1.74041	Sphk1
ILMN_1238276	0.000892	3.55E-05	-7.3067	2.581123	-1.74974	Hist1h2ai
ILMN_1213286	3.78E-05	9.49E-08	-14.4609	8.573452	-1.75313	Ccl21a
ILMN_2715840	6.66E-05	3.81E-07	-12.3894	7.217897	-1.77499	C1qc
ILMN_2899599	0.030784	4.45E-03	-3.71347	-2.47021	-1.78245	Ddx3y
ILMN_2538422	0.002047	1.14E-04	-6.30858	1.356969	-1.78247	Adap1
ILMN_1241350	0.00027	5.61E-06	-9.11239	4.496066	-1.79714	Vsig4
ILMN_1254082	0.000178	2.71E-06	-9.91617	5.242986	-1.80037	Chodl
ILMN_2689790	0.000629	2.11E-05	-7.78538	3.123957	-1.80328	Igfbp6
ILMN_2831436	3.99E-05	1.20E-07	-14.092	8.348703	-1.81391	Fcrls
ILMN_2918838	0.001868	1.01E-04	-6.40669	1.483119	-1.81462	Ptms
ILMN_2619620	3.67E-05	8.59E-08	-14.6202	8.668405	-1.83123	C1qb
ILMN_1220034	0.004495	3.41E-04	-5.45998	0.209499	-1.8399	Junb
ILMN_2839569	2.23E-05	2.11E-08	-17.0522	9.980988	-1.84331	Ctsz
ILMN_2782964	5.13E-05	2.21E-07	-13.1664	7.75408	-1.84491	Enpp1

ILMN_2927638	0.001616	8.35E-05	-6.5658	1.684951	-1.84566	Mxra8
ILMN_2623983	0.014373	1.63E-03	-4.36088	-1.42993	-1.84945	Egr2
ILMN_2738825	0.004589	3.52E-04	-5.4355	0.174873	-1.85726	Acta1
ILMN_2691567	2.79E-05	4.44E-08	-15.7236	9.29451	-1.85903	Hyi
ILMN_2602139	1.77E-05	1.27E-08	-18.0251	10.44222	-1.85949	LOC100041504
ILMN_2711163	0.000146	1.90E-06	-10.3317	5.607	-1.86331	Ctsk
ILMN_1233455	4.61E-05	1.61E-07	-13.6416	8.065037	-1.87389	Olfml3
ILMN_2543688	0.000207	3.57E-06	-9.6044	4.960189	-1.88017	Snhg18
ILMN_2658501	8.26E-05	6.40E-07	-11.6885	6.702327	-1.88333	Ifitm3
ILMN_1223317	0.000145	1.87E-06	-10.3508	5.623448	-1.89301	Lgals3
ILMN_2691951	0.001195	5.40E-05	-6.93583	2.141319	-1.8966	Svep1
ILMN_3127595	0.00016	2.29E-06	-10.1125	5.416779	-1.90172	Lman1l
ILMN_1226016	0.000285	6.26E-06	-8.9969	4.383824	-1.90649	Scx
ILMN_2967266	0.000115	1.27E-06	-10.811	6.009621	-1.91127	Fxyd5
ILMN_2639239	4.46E-05	1.40E-07	-13.8464	8.195317	-1.91279	Serpinf1
ILMN_3161547	0.00324	2.17E-04	-5.80152	0.683631	-1.94507	Nppa
ILMN_2817714	8.49E-05	6.72E-07	-11.6233	6.652736	-1.95617	Tspan17
ILMN_2969845	3.77E-05	9.07E-08	-14.5324	8.616208	-1.99371	Ccl21c
ILMN_1246153	0.000217	3.87E-06	-9.51648	4.878885	-2.01231	Erdr1
ILMN_2870864	2.63E-05	3.61E-08	-16.0851	9.488222	-2.0316	Pamr1
ILMN_1247811	5.13E-05	2.04E-07	-13.2843	7.832386	-2.06244	Ass1
ILMN_2852957	0.000105	1.02E-06	-11.0868	6.233349	-2.07019	Dkk3
ILMN_1238886	0.000118	1.34E-06	-10.7526	5.961527	-2.11169	Ccl8
ILMN_2690603	7.99E-05	5.73E-07	-11.8345	6.812341	-2.13109	Spp1
ILMN_2735350	0.011574	1.21E-03	-4.56078	-1.11915	-2.14944	Gdf15
ILMN_2615412	0.000149	1.95E-06	-10.2986	5.57858	-2.14989	Gng13

ILMN_2646052	0.000223	4.11E-06	-9.44858	4.815621	-2.15327	C4a
ILMN_2833163	0.000489	1.45E-05	-8.14369	3.512952	-2.16459	Fam180a
ILMN_2773113	5.87E-05	2.93E-07	-12.76	7.478056	-2.18199	Capg
ILMN_2663249	6.84E-05	3.98E-07	-12.3297	7.175203	-2.19108	Slamf9
ILMN_2750515	0.000106	1.05E-06	-11.0551	6.207885	-2.192	Fos
ILMN_2997494	0.002953	1.91E-04	-5.90184	0.819724	-2.25563	Lox
ILMN_2818294	0.000243	4.75E-06	-9.29143	4.667559	-2.2673	Srpx2
ILMN_2724880	0.035326	5.35E-03	-3.59762	-2.66099	-2.30384	Nmrk2
ILMN_2734683	0.000379	9.84E-06	-8.52902	3.915591	-2.3201	Fstl1
ILMN_2806065	0.000144	1.85E-06	-10.3609	5.632039	-2.32045	Ankrd2
ILMN_2698499	5.33E-05	2.34E-07	-13.0812	7.696952	-2.36598	Nox4
ILMN_1213886	6.42E-05	3.59E-07	-12.474	7.277991	-2.48595	Meox1
ILMN_3103896	0.005086	4.08E-04	-5.3268	0.020066	-2.56184	Timp1
ILMN_1238433	1.56E-05	8.70E-09	-18.7837	10.77995	-2.58965	Rbp1
ILMN_1225835	2.79E-05	4.37E-08	-15.7513	9.309521	-2.63529	Mfap5
ILMN_2633350	0.000138	1.69E-06	-10.4661	5.721736	-2.67376	Mfap4
ILMN_2747811	0.00033	8.00E-06	-8.74039	4.129849	-2.76013	Crlf1
ILMN_2742042	5.13E-05	2.05E-07	-13.2756	7.826629	-2.84461	Nupr1
ILMN_2503553	2.54E-05	3.06E-08	-16.3766	9.640541	-3.03408	Col8a1
ILMN_2486906	8.9E-05	7.39E-07	-11.5004	6.558389	-3.28501	Wisp2
ILMN_2636424	5.68E-06	3.76E-10	-26.3675	13.35165	-3.37448	Itgbl1
ILMN_1227100	4.26E-06	9.41E-11	-30.593	14.33415	-3.6075	Uba6

Table S1. GO analysis of DEGs in VO group of up-regulated differential gene.

Ontology	ID	Description	GeneRatio	BgRatio	pvalue	p.adjust	qvalue
BP	GO:0006790	sulfur compound metabolic process	5/35	272/23210	5.19e-05	0.028	0.024
BP	GO:0006749	glutathione metabolic process	3/35	59/23210	9.64e-05	0.028	0.024
BP	GO:0035634	response to stilbenoid	2/35	22/23210	5.01e-04	0.092	0.079
BP	GO:0006805	xenobiotic metabolic process	3/35	112/23210	6.40e-04	0.092	0.079
MF	GO:0004364	glutathione transferase activity	3/35	32/22710	1.61e-05	0.002	0.001
MF	GO:0016765	transferase activity, transferring alkyl or aryl (other than methyl) groups	3/35	61/22710	1.14e-04	0.006	0.004
MF	GO:0043295	glutathione binding	2/35	17/22710	3.09e-04	0.008	0.006
MF	GO:1900750	oligopeptide binding	2/35	17/22710	3.09e-04	0.008	0.006
KEGG	mmu00480	Glutathione metabolism	3/14	71/8910	1.66e-04	0.003	0.002
KEGG	mmu00982	Drug metabolism - cytochrome P450	3/14	71/8910	1.66e-04	0.003	0.002
KEGG	mmu00980	Metabolism of xenobiotics by cytochrome P450	3/14	73/8910	1.80e-04	0.003	0.002
KEGG	mmu01524	Platinum drug resistance	3/14	80/8910	2.36e-04	0.003	0.002

Table S2. GO analysis of DEGs in VO group of down-regulated differential gene.

Ontology	ID	Description	GeneRatio	BgRatio	pvalue	p.adjust	qvalue
BP	GO:0007005	mitochondrion organization	151/2381	459/23210	1.13e-40	6.61e-37	5.09e-37
BP	GO:0072594	establishment of protein localization to organelle	115/2381	374/23210	3.15e-28	9.23e-25	7.10e-25
BP	GO:0009259	ribonucleotide metabolic process	123/2381	433/23210	1.39e-26	2.10e-23	1.62e-23
CC	GO:0005759	mitochondrial matrix	148/2416	264/23436	2.22e-76	1.63e-73	1.18e-73
CC	GO:0098798	mitochondrial protein complex	128/2416	263/23436	1.42e-56	5.24e-54	3.80e-54
CC	GO:0005743	mitochondrial inner membrane	162/2416	433/23436	8.50e-52	2.08e-49	1.51e-49
MF	GO:0050662	coenzyme binding	85/2363	288/22710	1.58e-19	1.73e-16	1.29e-16

MF	GO:0003735	structural constituent of ribosome	63/2363	182/22710	1.23e-18	6.76e-16	5.05e-16
MF	GO:0051082	unfolded protein binding	37/2363	97/22710	5.17e-13	1.89e-10	1.41e-10
KEGG	mmu04146	Peroxisome	41/1165	86/8910	5.07e-15	1.59e-12	1.31e-12
KEGG	mmu00280	Valine, leucine and isoleucine degradation	30/1165	57/8910	8.93e-13	1.40e-10	1.15e-10
KEGG	mmu00640	Propanoate metabolism	21/1165	34/8910	4.03e-11	4.22e-09	3.46e-09

Table S3. GO analysis of DEGs in TAC group of up-regulated differential gene.

Ontology	ID	Description	GeneRatio	BgRatio	pvalue	p.adjust	qvalue
BP	GO:0016054	organic acid catabolic process	31/564	213/23210	1.21e-15	2.40e-12	1.77e-12
BP	GO:0046395	carboxylic acid catabolic process	31/564	213/23210	1.21e-15	2.40e-12	1.77e-12
BP	GO:0019395	fatty acid oxidation	22/564	98/23210	1.62e-15	2.40e-12	1.77e-12
CC	GO:0042383	sarcolemma	28/571	163/23436	4.01e-16	2.20e-13	1.59e-13
CC	GO:0005759	mitochondrial matrix	32/571	264/23436	8.73e-14	2.40e-11	1.74e-11
CC	GO:0019866	organelle inner membrane	43/571	475/23436	1.58e-13	2.88e-11	2.09e-11
MF	GO:0050662	coenzyme binding	32/568	288/22710	1.92e-12	1.53e-09	1.14e-09
MF	GO:0003779	actin binding	34/568	428/22710	3.54e-09	1.41e-06	1.05e-06
MF	GO:0003995	acyl-CoA dehydrogenase activity	6/568	10/22710	4.60e-08	1.22e-05	9.10e-06
KEGG	mmu00280	Valine, leucine and isoleucine degradation	14/294	57/8910	2.86e-09	7.81e-07	6.23e-07
KEGG	mmu00640	Propanoate metabolism	11/294	34/8910	6.10e-09	8.32e-07	6.64e-07
KEGG	mmu04931	Insulin resistance	18/294	110/8910	1.64e-08	1.50e-06	1.19e-06

Table S4. GO analysis of DEGs in TAC group of down-regulated differential gene.

Ontology	ID	Description	GeneRatio	BgRatio	pvalue	p.adjust	qvalue
BP	GO:0050900	leukocyte migration	32/455	340/23210	2.60e-13	1.12e-09	7.69e-10

BP	GO:0030595	leukocyte chemotaxis	25/455	211/23210	6.93e-13	1.50e-09	1.02e-09
BP	GO:0060326	cell chemotaxis	29/455	299/23210	1.66e-12	2.39e-09	1.64e-09
CC	GO:0062023	collagen-containing extracellular matrix	43/463	359/23436	2.32e-21	1.01e-18	8.11e-19
CC	GO:0031012	extracellular matrix	48/463	475/23436	1.45e-20	3.16e-18	2.54e-18
CC	GO:0045335	phagocytic vesicle	12/463	89/23436	1.84e-07	2.66e-05	2.14e-05
MF	GO:0005201	extracellular matrix structural constituent	18/442	140/22710	2.75e-10	1.82e-07	1.55e-07
MF	GO:0005539	glycosaminoglycan binding	19/442	208/22710	2.83e-08	9.38e-06	7.98e-06
MF	GO:0051015	actin filament binding	18/442	200/22710	8.25e-08	1.82e-05	1.55e-05
KEGG	mmu05140	Leishmaniasis	11/221	70/8910	1.02e-06	2.54e-04	2.21e-04
KEGG	mmu05152	Tuberculosis	15/221	180/8910	3.90e-05	0.005	0.004
KEGG	mmu05133	Pertussis	9/221	77/8910	1.13e-04	0.009	0.008

Table S5. GO analysis of DEGs in VO and TAC groups with same regulation.

ONTOLOGY	ID	Description	GeneRatio	BgRatio	pvalue	p.adjust	qvalue
BP	GO:0045047	protein targeting to ER	3/46	33/23210	3.8126E-05	0.02231621	0.01652736
BP	GO:0072599	establishment of protein localization to endoplasmic reticulum	3/46	37/23210	5.3996E-05	0.02231621	0.01652736
BP	GO:0031348	negative regulation of defense response	5/46	220/23210	7.3033E-05	0.02231621	0.01652736
BP	GO:0090150	establishment of protein localization to membrane	5/46	223/23210	7.7855E-05	0.02231621	0.01652736
BP	GO:0010257	NADH dehydrogenase complex assembly	3/46	45/23210	9.7522E-05	0.02231621	0.01652736
BP	GO:0032981	mitochondrial respiratory chain complex I assembly	3/46	45/23210	9.7522E-05	0.02231621	0.01652736
BP	GO:2001233	regulation of apoptotic signaling pathway	6/46	414/23210	0.00015912	0.02937918	0.02175818
BP	GO:1903749	positive regulation of establishment of protein localization to mitochondrion	2/46	10/23210	0.00017118	0.02937918	0.02175818
BP	GO:0150079	negative regulation of neuroinflammatory response	2/46	11/23210	0.00020896	0.03008583	0.02228153
BP	GO:0070972	protein localization to endoplasmic reticulum	3/46	59/23210	0.00021912	0.03008583	0.02228153
BP	GO:0008654	phospholipid biosynthetic process	4/46	158/23210	0.00027002	0.03083803	0.02283861
BP	GO:00070	mitochondrion organization	6/46	459/2321	0.000277	0.030838	0.022838

	05			0	47	03	61
	GO:01500				0.000291	0.030838	0.022838
BP	76	neuroinflammatory response	3/46	65/23210	98	03	61
	GO:00331				0.000480	0.035691	0.026433
BP	08	mitochondrial respiratory chain complex assembly	3/46	77/23210	92	44	03
	GO:00066				0.000512	0.035691	0.026433
BP	14	SRP-dependent cotranslational protein targeting to membrane	2/46	17/23210	8	44	03
	GO:00708				0.000512	0.035691	0.026433
BP	86	positive regulation of calcineurin-NFAT signaling cascade	2/46	17/23210	8	44	03
	GO:01060				0.000512	0.035691	0.026433
BP	58	positive regulation of calcineurin-mediated signaling	2/46	17/23210	8	44	03
	GO:00380				0.000518	0.035691	0.026433
BP	34	signal transduction in absence of ligand	3/46	79/23210	46	44	03
	GO:00971				0.000518	0.035691	0.026433
BP	92	extrinsic apoptotic signaling pathway in absence of ligand	3/46	79/23210	46	44	03
	GO:00321			336/2321	0.000519	0.035691	0.026433
BP	02	negative regulation of response to external stimulus	5/46	0	9	44	03
	GO:00066				0.000576	0.037670	0.027898
BP	13	cotranslational protein targeting to membrane	2/46	18/23210	17	51	73
	GO:20003			200/2321	0.000657	0.041051	0.030403
BP	77	regulation of reactive oxygen species metabolic process	4/46	0	79	95	03
	GO:00061				0.000787	0.045070	0.033379
BP	20	mitochondrial electron transport, NADH to ubiquinone	2/46	21/23210	83	58	22
	GO:19012				0.000787	0.045070	0.033379
BP	23	negative regulation of NIK/NF-kappaB signaling	2/46	21/23210	83	58	22
BP	GO:00725	establishment of protein localization to organelle	5/46	374/2321	0.000842	0.045706	0.033850

	94			0	68	27	01
	GO:00326				0.000865	0.045706	0.033850
BP	91	negative regulation of interleukin-1 beta production	2/46	22/23210	52	27	01
	GO:20012				0.000971	0.048228	0.035718
BP	37	negative regulation of extrinsic apoptotic signaling pathway	3/46	98/23210	31	96	31
	GO:00506				0.001031	0.048228	0.035718
BP	87	negative regulation of defense response to virus	2/46	24/23210	53	96	31
	GO:01500				0.001119	0.048228	0.035718
BP	77	regulation of neuroinflammatory response	2/46	25/23210	81	96	31
	GO:19037				0.001119	0.048228	0.035718
BP	47	regulation of establishment of protein localization to mitochondrion	2/46	25/23210	81	96	31
	GO:19034			103/2321	0.001121	0.048228	0.035718
BP	26	regulation of reactive oxygen species biosynthetic process	3/46	0	62	96	31
	GO:20012			231/2321	0.001124	0.048228	0.035718
BP	34	negative regulation of apoptotic signaling pathway	4/46	0	05	96	31
	GO:00464			106/2321	0.001218	0.050695	0.037545
BP	74	glycerophospholipid biosynthetic process	3/46	0	47	68	16
	GO:00066			109/2321	0.001320	0.053322	0.039490
BP	12	protein targeting to membrane	3/46	0	44	58	63
	GO:00086			111/2321	0.001391	0.054579	0.040421
BP	37	apoptotic mitochondrial changes	3/46	0	32	56	55
	GO:00326				0.001613	0.060849	0.045064
BP	92	negative regulation of interleukin-1 production	2/46	30/23210	52	01	7
	GO:00066			256/2321	0.001639	0.060849	0.045064
BP	05	protein targeting	4/46	0	78	01	7
BP	GO:19034	reactive oxygen species biosynthetic process	3/46	121/2321	0.001781	0.064370	0.047672

	09			0	56	62	8
	GO:20011				0.001835	0.064606	0.047847
BP	71	positive regulation of ATP biosynthetic process	2/46	32/23210	15	86	76
	GO:19010				0.001951	0.065337	0.048389
BP	99	negative regulation of signal transduction in absence of ligand	2/46	33/23210	09	79	09
	GO:20012	negative regulation of extrinsic apoptotic signaling pathway in absence			0.001951	0.065337	0.048389
BP	40	of ligand	2/46	33/23210	09	79	09
	GO:00198				0.002193	0.071694	0.053097
BP	35	cytolysis	2/46	35/23210	14	85	11
	GO:00026			473/2321	0.002371	0.073472	0.054413
BP	83	negative regulation of immune system process	5/46	0	11	38	55
	GO:00308				0.002448	0.073472	0.054413
BP	10	positive regulation of nucleotide biosynthetic process	2/46	37/23210	67	38	55
	GO:19003				0.002448	0.073472	0.054413
BP	73	positive regulation of purine nucleotide biosynthetic process	2/46	37/23210	67	38	55
	GO:00108			136/2321	0.002484	0.073472	0.054413
BP	21	regulation of mitochondrion organization	3/46	0	7	38	55
	GO:00725			288/2321	0.002515	0.073472	0.054413
BP	93	reactive oxygen species metabolic process	4/46	0	08	38	55
	GO:00450			141/2321	0.002752	0.075435	0.055867
BP	17	glycerolipid biosynthetic process	3/46	0	1	78	65
	GO:00073			493/2321	0.002834	0.075435	0.055867
BP	46	regulation of mitotic cell cycle	5/46	0	77	78	65
	GO:00091			298/2321	0.002843	0.075435	0.055867
BP	41	nucleoside triphosphate metabolic process	4/46	0	88	78	65
BP	GO:00708	regulation of calcineurin-NFAT signaling cascade	2/46	40/23210	0.002857	0.075435	0.055867

	84					78	65
	GO:01060					0.075435	0.055867
BP	56	regulation of calcineurin-mediated signaling	2/46	40/23210	0.002857	78	65
	GO:00028				0.002999	0.077710	0.057552
BP	32	negative regulation of response to biotic stimulus	2/46	41/23210	73	11	01
	GO:00105				0.003145	0.079983	0.059235
BP	59	regulation of glycoprotein biosynthetic process	2/46	42/23210	76	86	95
	GO:00507			149/2321	0.003215	0.080276	0.059452
BP	28	negative regulation of inflammatory response	3/46	0	72	03	33
	GO:00434				0.003295	0.080787	0.059831
BP	03	skeletal muscle tissue regeneration	2/46	43/23210	06	89	41
	GO:00149				0.003447	0.083045	0.061503
BP	04	myotube cell development	2/46	44/23210	63	56	44
	GO:00518			155/2321	0.003593	0.083336	0.061719
BP	96	regulation of protein kinase B signaling	3/46	0	07	72	07
	GO:19035				0.003603	0.083336	0.061719
BP	80	positive regulation of ATP metabolic process	2/46	45/23210	45	72	07
	GO:00901				0.003924	0.083336	0.061719
BP	99	regulation of release of cytochrome c from mitochondria	2/46	47/23210	79	72	07
	GO:19030				0.003924	0.083336	0.061719
BP	18	regulation of glycoprotein metabolic process	2/46	47/23210	79	72	07
	GO:20012				0.003924	0.083336	0.061719
BP	39	regulation of extrinsic apoptotic signaling pathway in absence of ligand	2/46	47/23210	79	72	07
	GO:00091			160/2321	0.003927	0.083336	0.061719
BP	42	nucleoside triphosphate biosynthetic process	3/46	0	4	72	07
BP	GO:20012	regulation of extrinsic apoptotic signaling pathway	3/46	161/2321	0.003996	0.083336	0.061719

	36			0	46	72	07
	GO:00504				0.004090	0.083336	0.061719
BP	35	amyloid-beta metabolic process	2/46	48/23210	29	72	07
	GO:00508				0.004090	0.083336	0.061719
BP	50	positive regulation of calcium-mediated signaling	2/46	48/23210	29	72	07
	GO:00326			164/2321	0.004208	0.083336	0.061719
BP	80	regulation of tumor necrosis factor production	3/46	0	09	72	07
	GO:00331				0.004258	0.083336	0.061719
BP	73	calcineurin-NFAT signaling cascade	2/46	49/23210	98	72	07
	GO:00439			165/2321	0.004280	0.083336	0.061719
BP	01	negative regulation of multi-organism process	3/46	0	12	72	07
	GO:19035			166/2321	0.004352	0.083336	0.061719
BP	55	regulation of tumor necrosis factor superfamily cytokine production	3/46	0	9	72	07
	GO:00326			167/2321	0.004426	0.083336	0.061719
BP	40	tumor necrosis factor production	3/46	0	43	72	07
	GO:00459				0.004430	0.083336	0.061719
BP	81	positive regulation of nucleotide metabolic process	2/46	50/23210	87	72	07
	GO:19005				0.004430	0.083336	0.061719
BP	44	positive regulation of purine nucleotide metabolic process	2/46	50/23210	87	72	07
	GO:00604				0.004605	0.085154	0.063065
BP	28	lung epithelium development	2/46	51/23210	93	21	1
	GO:00717			170/2321	0.004651	0.085154	0.063065
BP	06	tumor necrosis factor superfamily cytokine production	3/46	0	54	21	1
	GO:00977				0.004784	0.086429	0.064009
BP	20	calcineurin-mediated signaling	2/46	52/23210	15	38	49
BP	GO:00066	phospholipid metabolic process	4/46	348/2321	0.004936	0.088031	0.065195

	44			0	93	24	83
	GO:00507			354/2321	0.005242	0.092282	0.068344
BP	27	regulation of inflammatory response	4/46	0	56	57	36
	GO:19055				0.005528	0.096082	0.071158
BP	17	macrophage migration	2/46	56/23210	39	09	28
	GO:00427				0.005722	0.096995	0.071834
BP	75	mitochondrial ATP synthesis coupled electron transport	2/46	57/23210	23	42	68
	GO:00480				0.005722	0.096995	0.071834
BP	16	inositol phosphate-mediated signaling	2/46	57/23210	23	42	68
	GO:00106				0.005919	0.098147	0.072687
BP	56	negative regulation of muscle cell apoptotic process	2/46	58/23210	16	3	77
	GO:00434			186/2321	0.005968	0.098147	0.072687
BP	91	protein kinase B signaling	3/46	0	92	3	77
	GO:19012			368/2321	0.006004	0.098147	0.072687
BP	14	regulation of neuron death	4/46	0	64	3	77
	GO:00485				0.000167	0.019813	0.012902
CC	00	signal recognition particle	2/46	10/23436	91	75	8
	GO:00310				0.000630	0.037224	0.024240
CC	91	platelet alpha granule	2/46	19/23436	93	76	92
	GO:00987			263/2343	0.001746	0.068712	0.044745
CC	98	mitochondrial protein complex	4/46	6	93	52	89
	GO:00003				0.003086	0.073300	0.047733
CC	07	cyclin-dependent protein kinase holoenzyme complex	2/46	42/23436	88	8	8
	GO:00057				0.004348	0.073300	0.047733
CC	47	mitochondrial respiratory chain complex I	2/46	50/23436	35	8	8
CC	GO:00309	NADH dehydrogenase complex	2/46	50/23436	0.004348	0.073300	0.047733

	64				35	8	8
	GO:00452				0.004348	0.073300	0.047733
CC	71	respiratory chain complex I	2/46	50/23436	35	8	8
	GO:00003				0.006205	0.078733	0.051271
CC	15	organellar large ribosomal subunit	2/46	60/23436	24	38	51
	GO:00057				0.006205	0.078733	0.051271
CC	62	mitochondrial large ribosomal subunit	2/46	60/23436	24	38	51
	GO:00301			383/2343	0.006672	0.078733	0.051271
CC	41	secretory granule	4/46	6	32	38	51
	GO:00444			216/2343	0.008757	0.093949	0.061180
CC	55	mitochondrial membrane part	3/46	6	99	37	23
	GO:00057				0.010043	0.095487	0.062181
CC	46	mitochondrial respiratory chain	2/46	77/23436	6	1	61
	GO:00988				0.011066	0.095487	0.062181
CC	03	respiratory chain complex	2/46	81/23436	37	1	61
	GO:19025				0.011328	0.095487	0.062181
CC	54	serine/threonine protein kinase complex	2/46	82/23436	98	1	61
	GO:00704				0.013243	0.097897	0.063751
CC	69	respiratory chain	2/46	89/23436	32	82	48
	GO:00003				0.014103	0.097897	0.063751
CC	13	organellar ribosome	2/46	92/23436	92	82	48
	GO:00057				0.014103	0.097897	0.063751
CC	61	mitochondrial ribosome	2/46	92/23436	92	82	48
	GO:00057			264/2343	0.015020	0.098465	0.064121
CC	59	mitochondrial matrix	3/46	6	22	87	4
KEGG	mmu0411	p53 signaling pathway	4/23	72/8910	3.0905E-	0.003832	0.003090

	5				05	2	48
KEGG	mmu05224	Breast cancer	4/23	147/8910	0.00049333	0.02679496	0.02160884
KEGG	mmu05218	Melanoma	3/23	72/8910	0.00079791	0.02679496	0.02160884
KEGG	mmu05214	Glioma	3/23	74/8910	0.00086435	0.02679496	0.02160884
KEGG	mmu01522	Endocrine resistance	3/23	93/8910	0.00167586	0.04156128	0.03351716
KEGG	mmu03060	Protein export	2/23	29/8910	0.00248058	0.0512654	0.04134306

Table S6. GO analysis of DEGs in VO and TAC groups with opposite regulation.

ONTOLOGY	ID	Description	GeneRatio	BgRatio	pvalue	p.adjust	qvalue
BP	GO:0016054	organic acid catabolic process	27/245	213/23210	2.1827E-21	3.3243E-18	2.5963E-18
BP	GO:0046395	carboxylic acid catabolic process	27/245	213/23210	2.1827E-21	3.3243E-18	2.5963E-18
BP	GO:0072329	monocarboxylic acid catabolic process	21/245	112/23210	1.5989E-20	1.6235E-17	1.2679E-17
BP	GO:0006631	fatty acid metabolic process	32/245	376/23210	7.8118E-20	5.9487E-17	4.646E-17
BP	GO:0009062	fatty acid catabolic process	19/245	93/23210	1.986E-19	1.2099E-16	9.4492E-17
BP	GO:0044	small molecule catabolic process	29/245	311/232	3.8449E-	1.9519E-	1.5245E-

	282			10	19	16	16
	GO:0019			98/2321	5.712E-1	2.4855E-	1.9412E-
BP	395	fatty acid oxidation	19/245	0	9	16	16
	GO:0034			100/232	8.5656E-	3.2614E-	2.5471E-
BP	440	lipid oxidation	19/245	10	19	16	16
	GO:0006			67/2321	1.0107E-	3.4206E-	2.6715E-
BP	635	fatty acid beta-oxidation	16/245	0	17	15	15
	GO:0044			199/232	2.4431E-	7.4418E-	5.8121E-
BP	242	cellular lipid catabolic process	22/245	10	16	14	14
	GO:0030			218/232	1.9368E-	5.3633E-	4.1888E-
BP	258	lipid modification	21/245	10	14	12	12
	GO:0033			11/2321	2.2075E-	5.6034E-	4.3763E-
BP	539	fatty acid beta-oxidation using acyl-CoA dehydrogenase	8/245	0	14	12	12
	GO:0006			447/232		1.0591E-	8.2714E-
BP	163	purine nucleotide metabolic process	28/245	10	4.52E-14	11	12
	GO:0009			423/232	8.4215E-	1.8323E-	1.431E-1
BP	150	purine ribonucleotide metabolic process	27/245	10	14	11	1
	GO:0009			433/232	1.4725E-	2.9901E-	2.3353E-
BP	259	ribonucleotide metabolic process	27/245	10	13	11	11
	GO:0016			306/232	2.169E-1	4.1293E-	3.225E-1
BP	042	lipid catabolic process	23/245	10	3	11	1
	GO:0019			450/232	3.6764E-	6.5872E-	5.1446E-
BP	693	ribose phosphate metabolic process	27/245	10	13	11	11
	GO:0072			490/232	4.3035E-	7.2824E-	5.6876E-
BP	521	purine-containing compound metabolic process	28/245	10	13	11	11
BP	GO:0033	nucleoside bisphosphate metabolic process	14/245	99/2321	2.5196E-	3.6547E-	2.8543E-

	865			0	12	10	10
BP	GO:0033875	ribonucleoside bisphosphate metabolic process	14/245	99/23210	2.5196E-12	3.6547E-10	2.8543E-10
BP	GO:0034032	purine nucleoside bisphosphate metabolic process	14/245	99/23210	2.5196E-12	3.6547E-10	2.8543E-10
BP	GO:0006637	acyl-CoA metabolic process	13/245	82/23210	3.4363E-12	4.5509E-10	3.5543E-10
BP	GO:0035383	thioester metabolic process	13/245	82/23210	3.4363E-12	4.5509E-10	3.5543E-10
BP	GO:0006732	coenzyme metabolic process	22/245	331/23210	8.5163E-12	1.0809E-09	8.4416E-10
BP	GO:0006091	generation of precursor metabolites and energy	23/245	379/23210	1.7838E-11	2.1734E-09	1.6975E-09
BP	GO:0006164	purine nucleotide biosynthetic process	18/245	240/23210	1.0044E-10	1.1767E-08	9.1904E-09
BP	GO:0072522	purine-containing compound biosynthetic process	18/245	245/23210	1.4085E-10	1.589E-08	1.241E-08
BP	GO:0009152	purine ribonucleotide biosynthetic process	17/245	223/23210	2.6202E-10	2.8504E-08	2.2262E-08
BP	GO:0009260	ribonucleotide biosynthetic process	17/245	232/23210	4.8387E-10	5.0823E-08	3.9693E-08
BP	GO:0046390	ribose phosphate biosynthetic process	17/245	239/23210	7.6492E-10	7.7665E-08	6.0657E-08
BP	GO:0044262	cellular carbohydrate metabolic process	18/245	284/23210	1.5284E-09	1.4935E-07	1.1664E-07
BP	GO:0009	nucleotide biosynthetic process	18/245	285/232	1.6163E-	1.4935E-	1.1664E-

	165			10	09	07	07
BP	GO:0051188	cofactor biosynthetic process	17/245	251/23210	1.618E-09	1.4935E-07	1.1664E-07
BP	GO:0015980	energy derivation by oxidation of organic compounds	17/245	254/23210	1.9383E-09	1.7365E-07	1.3562E-07
BP	GO:1901293	nucleoside phosphate biosynthetic process	18/245	291/23210	2.2493E-09	1.9269E-07	1.5049E-07
BP	GO:0009108	coenzyme biosynthetic process	15/245	194/23210	2.4985E-09	1.9269E-07	1.5049E-07
BP	GO:0033866	nucleoside bisphosphate biosynthetic process	8/245	35/23210	2.5304E-09	1.9269E-07	1.5049E-07
BP	GO:0034030	ribonucleoside bisphosphate biosynthetic process	8/245	35/23210	2.5304E-09	1.9269E-07	1.5049E-07
BP	GO:0034033	purine nucleoside bisphosphate biosynthetic process	8/245	35/23210	2.5304E-09	1.9269E-07	1.5049E-07
BP	GO:0046320	regulation of fatty acid oxidation	8/245	35/23210	2.5304E-09	1.9269E-07	1.5049E-07
BP	GO:0006790	sulfur compound metabolic process	17/245	272/23210	5.4442E-09	3.8814E-07	3.0314E-07
BP	GO:0035384	thioester biosynthetic process	7/245	25/23210	5.4794E-09	3.8814E-07	3.0314E-07
BP	GO:0071616	acyl-CoA biosynthetic process	7/245	25/23210	5.4794E-09	3.8814E-07	3.0314E-07
BP	GO:0003012	muscle system process	20/245	392/23210	7.7288E-09	5.3505E-07	4.1788E-07
BP	GO:0006	acetyl-CoA metabolic process	7/245	33/2321	4.5315E-	3.0673E-	2.3956E-

	084			0	08	06	06
BP	GO:0008016	regulation of heart contraction	13/245	177/23210	5.4394E-08	3.6018E-06	2.8131E-06
BP	GO:0062012	regulation of small molecule metabolic process	18/245	359/23210	5.7884E-08	3.7514E-06	2.9298E-06
BP	GO:0005996	monosaccharide metabolic process	15/245	253/23210	8.8216E-08	5.5981E-06	4.3721E-06
BP	GO:0086001	cardiac muscle cell action potential	8/245	54/23210	9.411E-08	5.8502E-06	4.569E-06
BP	GO:0006109	regulation of carbohydrate metabolic process	13/245	186/23210	9.7474E-08	5.9381E-06	4.6377E-06
BP	GO:0019318	hexose metabolic process	14/245	223/23210	1.1858E-07	7.0825E-06	5.5314E-06
BP	GO:0005977	glycogen metabolic process	9/245	78/23210	1.3596E-07	7.6691E-06	5.9896E-06
BP	GO:0006073	cellular glucan metabolic process	9/245	78/23210	1.3596E-07	7.6691E-06	5.9896E-06
BP	GO:0044042	glucan metabolic process	9/245	78/23210	1.3596E-07	7.6691E-06	5.9896E-06
BP	GO:0046322	negative regulation of fatty acid oxidation	5/245	13/23210	1.5112E-07	8.3695E-06	6.5366E-06
BP	GO:0044264	cellular polysaccharide metabolic process	10/245	104/23210	1.5655E-07	8.5117E-06	6.6477E-06
BP	GO:0006006	glucose metabolic process	13/245	194/23210	1.5928E-07	8.5117E-06	6.6477E-06
BP	GO:0086	cardiac muscle cell contraction	8/245	62/2321	2.8438E-	1.4935E-	1.1664E-

	003			0	07	05	05
	GO:0016			142/232	3.4241E-	1.7678E-	1.3806E-
BP	052	carbohydrate catabolic process	11/245	10	07	05	05
	GO:0006			88/2321	3.8869E-	1.9409E-	1.5159E-
BP	112	energy reserve metabolic process	9/245	0	07	05	05
	GO:0019			88/2321	3.8869E-	1.9409E-	1.5159E-
BP	217	regulation of fatty acid metabolic process	9/245	0	07	05	05
	GO:0005			115/232	4.0401E-	1.9849E-	1.5502E-
BP	976	polysaccharide metabolic process	10/245	10	07	05	05
	GO:0060			211/232	4.1917E-	2.0266E-	1.5828E-
BP	047	heart contraction	13/245	10	07	05	05
	GO:0009			90/2321	4.7175E-	2.2452E-	1.7536E-
BP	063	cellular amino acid catabolic process	9/245	0	07	05	05
	GO:0002			93/2321	6.2507E-	2.9292E-	2.2877E-
BP	027	regulation of heart rate	9/245	0	07	05	05
	GO:0003			221/232	7.0953E-	3.2746E-	2.5575E-
BP	015	heart process	13/245	10	07	05	05
	GO:0006			261/232	8.0112E-	3.6421E-	2.8445E-
BP	520	cellular amino acid metabolic process	14/245	10	07	05	05
	GO:0060			124/232	8.141E-0	3.6467E-	2.8481E-
BP	048	cardiac muscle contraction	10/245	10	7	05	05
	GO:0005			18/2321	9.6356E-	4.1338E-	3.2285E-
BP	980	glycogen catabolic process	5/245	0	07	05	05
	GO:0006			18/2321	9.6356E-	4.1338E-	3.2285E-
BP	085	acetyl-CoA biosynthetic process	5/245	0	07	05	05
BP	GO:0009	glucan catabolic process	5/245	18/2321	9.6356E-	4.1338E-	3.2285E-

	251			0	07	05	05
BP	GO:0008015	blood circulation	19/245	484/23210	1.0609E-06	4.4881E-05	3.5053E-05
BP	GO:0034764	positive regulation of transmembrane transport	13/245	233/23210	1.286E-06	5.3658E-05	4.1907E-05
BP	GO:1904064	positive regulation of cation transmembrane transport	11/245	163/23210	1.3547E-06	5.5762E-05	4.355E-05
BP	GO:0003013	circulatory system process	19/245	495/23210	1.4789E-06	5.9655E-05	4.6591E-05
BP	GO:0051289	protein homotetramerization	9/245	103/23210	1.4884E-06	5.9655E-05	4.6591E-05
BP	GO:0046394	carboxylic acid biosynthetic process	16/245	362/23210	1.6969E-06	6.3659E-05	4.9718E-05
BP	GO:0034637	cellular carbohydrate biosynthetic process	8/245	78/23210	1.7056E-06	6.3659E-05	4.9718E-05
BP	GO:0090257	regulation of muscle system process	13/245	239/23210	1.7074E-06	6.3659E-05	4.9718E-05
BP	GO:1903522	regulation of blood circulation	13/245	239/23210	1.7074E-06	6.3659E-05	4.9718E-05
BP	GO:0044247	cellular polysaccharide catabolic process	5/245	20/23210	1.7137E-06	6.3659E-05	4.9718E-05
BP	GO:0051481	negative regulation of cytosolic calcium ion concentration	5/245	20/23210	1.7137E-06	6.3659E-05	4.9718E-05
BP	GO:0016053	organic acid biosynthetic process	16/245	363/23210	1.7586E-06	6.4063E-05	5.0034E-05
BP	GO:0043	regulation of carbohydrate catabolic process	7/245	55/2321	1.7667E-	6.4063E-	5.0034E-

	470			0	06	05	05
	GO:0044			79/2321	1.8806E-	6.7393E-	5.2634E-
BP	272	sulfur compound biosynthetic process	8/245	0	06	05	05
	GO:0030			108/232	2.2161E-	7.849E-0	6.1301E-
BP	048	actin filament-based movement	9/245	10	06	5	05
	GO:0086			37/2321	2.2999E-	8.0522E-	6.2888E-
BP	065	cell communication involved in cardiac conduction	6/245	0	06	05	05
	GO:0061			58/2321	2.5482E-	8.8202E-	6.8886E-
BP	337	cardiac conduction	7/245	0	06	05	05
	GO:0034			179/232	3.3777E-	0.000115	9.0284E-
BP	767	positive regulation of ion transmembrane transport	11/245	10	06	6	05
	GO:0019			338/232	3.4269E-	0.000115	9.0583E-
BP	216	regulation of lipid metabolic process	15/245	10	06	98	05
	GO:0043			86/2321	3.5897E-		9.372E-0
BP	648	dicarboxylic acid metabolic process	8/245	0	06	0.00012	5
	GO:0000			23/2321	3.6244E-		9.372E-0
BP	272	polysaccharide catabolic process	5/245	0	06	0.00012	5
	GO:0086			11/2321	3.7719E-	0.000122	9.546E-0
BP	016	AV node cell action potential	4/245	0	06	23	5
	GO:0086			11/2321	3.7719E-	0.000122	9.546E-0
BP	027	AV node cell to bundle of His cell signaling	4/245	0	06	23	5
	GO:0070			87/2321	3.9176E-	0.000125	9.8102E-
BP	252	actin-mediated cell contraction	8/245	0	06	61	05
	GO:0045			24/2321	4.5389E-	0.000144	0.000112
BP	922	negative regulation of fatty acid metabolic process	5/245	0	06	01	48
BP	GO:0072	monocarboxylic acid biosynthetic process	13/245	264/232	5.093E-0	0.000159	0.000124

	330			10	6	93	91
BP	GO:0010675	regulation of cellular carbohydrate metabolic process	10/245	152/23210	5.1703E-06	0.0001607	0.00012551
BP	GO:0009437	carnitine metabolic process	4/245	12/23210	5.611E-06	0.00016754	0.00013085
BP	GO:0086019	cell-cell signaling involved in cardiac conduction	5/245	25/23210	5.6249E-06	0.00016754	0.00013085
BP	GO:0010906	regulation of glucose metabolic process	9/245	121/23210	5.6836E-06	0.00016754	0.00013085
BP	GO:0005978	glycogen biosynthetic process	6/245	43/23210	5.7202E-06	0.00016754	0.00013085
BP	GO:0009250	glucan biosynthetic process	6/245	43/23210	5.7202E-06	0.00016754	0.00013085
BP	GO:0086002	cardiac muscle cell action potential involved in contraction	6/245	43/23210	5.7202E-06	0.00016754	0.00013085
BP	GO:0033692	cellular polysaccharide biosynthetic process	7/245	67/23210	6.7992E-06	0.00019724	0.00015405
BP	GO:0006941	striated muscle contraction	10/245	158/23210	7.2912E-06	0.00020796	0.00016242
BP	GO:0016051	carbohydrate biosynthetic process	11/245	194/23210	7.3051E-06	0.00020796	0.00016242
BP	GO:0086067	AV node cell to bundle of His cell communication	4/245	13/23210	8.0376E-06	0.00022669	0.00017705
BP	GO:0010565	regulation of cellular ketone metabolic process	9/245	127/23210	8.4411E-06	0.00023374	0.00018255
BP	GO:0043	regulation of generation of precursor metabolites and energy	9/245	127/232	8.4411E-	0.000233	0.000182

	467			10	06	74	55
	GO:0043			322/232	9.1958E-	0.000252	0.000197
BP	270	positive regulation of ion transport	14/245	10	06	35	08
	GO:0006			14/2321	1.1159E-	0.000300	0.000234
BP	577	amino-acid betaine metabolic process	4/245	0	05	81	94
	GO:0046			14/2321	1.1159E-	0.000300	0.000234
BP	459	short-chain fatty acid metabolic process	4/245	0	05	81	94
	GO:0003			101/232	1.1936E-	0.000318	0.000249
BP	300	cardiac muscle hypertrophy	8/245	10	05	92	08
	GO:0006			134/232	1.3034E-	0.000345	0.000269
BP	633	fatty acid biosynthetic process	9/245	10	05	24	64
	GO:0050			169/232	1.317E-0	0.000345	0.000270
BP	821	protein stabilization	10/245	10	5	84	1
	GO:0006			289/232	1.344E-0	0.000349	0.000273
BP	936	muscle contraction	13/245	10	5	89	27
	GO:0034			478/232	1.4424E-	0.000369	0.000288
BP	765	regulation of ion transmembrane transport	17/245	10	05	76	79
	GO:0000			75/2321	1.4446E-	0.000369	0.000288
BP	271	polysaccharide biosynthetic process	7/245	0	05	76	79
	GO:0009			15/2321	1.5091E-	0.000383	0.000299
BP	083	branched-chain amino acid catabolic process	4/245	0	05	07	18
	GO:1901			76/2321	1.577E-0	0.000396	0.000309
BP	606	alpha-amino acid catabolic process	7/245	0	5	53	69
	GO:0014			105/232	1.5882E-	0.000396	0.000309
BP	897	striated muscle hypertrophy	8/245	10	05	53	69
BP	GO:0048	multicellular organismal homeostasis	17/245	486/232	1.7834E-	0.000441	0.000344

	871			10	05	65	93
	GO:0014			107/232	1.8234E-	0.000447	0.000349
BP	896	muscle hypertrophy	8/245	10	05	9	81
	GO:0009			16/2321	1.9955E-	0.000486	0.000379
BP	081	branched-chain amino acid metabolic process	4/245	0	05	27	78
	GO:0042			395/232	2.1843E-	0.000528	0.000412
BP	692	muscle cell differentiation	15/245	10	05	04	41
	GO:1904			80/2321	2.2109E-	0.000530	0.000414
BP	427	positive regulation of calcium ion transmembrane transport	7/245	0	05	27	15
	GO:1904			349/232	2.2584E-	0.000537	0.000419
BP	062	regulation of cation transmembrane transport	14/245	10	05	44	74
	GO:0086			33/2321	2.3454E-	0.000553	0.000432
BP	091	regulation of heart rate by cardiac conduction	5/245	0	05	8	52
	GO:0051			183/232	2.6219E-	0.000614	0.000479
BP	262	protein tetramerization	10/245	10	05	34	81
	GO:0051			56/2321	2.7168E-	0.000629	0.000491
BP	196	regulation of coenzyme metabolic process	6/245	0	05	1	33
	GO:0044			34/2321	2.7262E-	0.000629	0.000491
BP	275	cellular carbohydrate catabolic process	5/245	0	05	1	33
	GO:0032			114/232	2.8898E-	0.000661	0.000516
BP	414	positive regulation of ion transmembrane transporter activity	8/245	10	05	83	9
	GO:0015			455/232	2.9625E-	0.000673	0.000525
BP	672	monovalent inorganic cation transport	16/245	10	05	42	94
	GO:0045			84/2321	3.0416E-	0.000686	0.000535
BP	913	positive regulation of carbohydrate metabolic process	7/245	0	05	28	99
BP	GO:0009	purine ribonucleoside triphosphate metabolic process	12/245	272/232	3.4929E-	0.000782	0.000610

	205			10	05	31	99
BP	GO:0006090	pyruvate metabolic process	8/245	118/23210	3.7048E-05	0.00082372	0.00064333
BP	GO:0071804	cellular potassium ion transport	10/245	191/23210	3.7776E-05	0.00082781	0.00064652
BP	GO:0071805	potassium ion transmembrane transport	10/245	191/23210	3.7776E-05	0.00082781	0.00064652
BP	GO:0009199	ribonucleoside triphosphate metabolic process	12/245	276/23210	4.025E-05	0.00087574	0.00068396
BP	GO:1901605	alpha-amino acid metabolic process	10/245	193/23210	4.1266E-05	0.00088898	0.0006943
BP	GO:0062013	positive regulation of small molecule metabolic process	9/245	155/23210	4.1443E-05	0.00088898	0.0006943
BP	GO:0010876	lipid localization	14/245	370/23210	4.2741E-05	0.00091042	0.00071105
BP	GO:0032411	positive regulation of transporter activity	8/245	121/23210	4.435E-05	0.00093813	0.00073268
BP	GO:0010959	regulation of metal ion transport	15/245	421/23210	4.5361E-05	0.0009529	0.00074422
BP	GO:0009144	purine nucleoside triphosphate metabolic process	12/245	281/23210	4.788E-05	0.00099893	0.00078017
BP	GO:0045821	positive regulation of glycolytic process	4/245	20/23210	5.1386E-05	0.00105759	0.00082598
BP	GO:0060307	regulation of ventricular cardiac muscle cell membrane repolarization	4/245	20/23210	5.1386E-05	0.00105759	0.00082598
BP	GO:0008	cholesterol metabolic process	8/245	126/232	5.9173E-	0.001209	0.000944

	203			10	05	68	77
BP	GO:0009409	response to cold	5/245	40/23210	6.1228E-05	0.0012351	0.00096462
BP	GO:0086004	regulation of cardiac muscle cell contraction	5/245	40/23210	6.1228E-05	0.0012351	0.00096462
BP	GO:0030813	positive regulation of nucleotide catabolic process	4/245	21/23210	6.2953E-05	0.00125329	0.00097883
BP	GO:0051197	positive regulation of coenzyme metabolic process	4/245	21/23210	6.2953E-05	0.00125329	0.00097883
BP	GO:0001666	response to hypoxia	10/245	204/23210	6.5793E-05	0.00130133	0.00101635
BP	GO:0046034	ATP metabolic process	11/245	247/23210	6.8012E-05	0.00133655	0.00104386
BP	GO:0099625	ventricular cardiac muscle cell membrane repolarization	4/245	22/23210	7.6306E-05	0.00148993	0.00116365
BP	GO:0097164	ammonium ion metabolic process	9/245	168/23210	7.7435E-05	0.00150191	0.001173
BP	GO:1902652	secondary alcohol metabolic process	8/245	131/23210	7.7906E-05	0.00150191	0.001173
BP	GO:0001508	action potential	8/245	132/23210	8.2187E-05	0.00157448	0.00122968
BP	GO:0009141	nucleoside triphosphate metabolic process	12/245	298/23210	8.3948E-05	0.00159816	0.00124818
BP	GO:0032412	regulation of ion transmembrane transporter activity	11/245	254/23210	8.7267E-05	0.00163405	0.0012762
BP	GO:1901	positive regulation of potassium ion transmembrane transport	5/245	43/2321	8.7292E-	0.001634	0.001276

	381			0	05	05	2
	GO:0062			99/2321	8.7443E-	0.001634	0.001276
BP	014	negative regulation of small molecule metabolic process	7/245	0	05	05	2
	GO:0055			69/2321	8.9478E-	0.001661	0.001297
BP	117	regulation of cardiac muscle contraction	6/245	0	05	9	95
	GO:0016			135/232	9.6218E-	0.001776	0.001387
BP	125	sterol metabolic process	8/245	10	05	25	26
	GO:0009			136/232	0.000101	0.001848	0.001443
BP	166	nucleotide catabolic process	8/245	10	31	11	39
	GO:0006			174/232	0.000101	0.001848	0.001443
BP	575	cellular modified amino acid metabolic process	9/245	10	32	11	39
	GO:0006			137/232	0.000106	0.001933	0.001509
BP	754	ATP biosynthetic process	8/245	10	63	33	94
	GO:0099			24/2321	0.000109	0.001964	0.001534
BP	623	regulation of cardiac muscle cell membrane repolarization	4/245	0	02	97	66
	GO:0043			103/232	0.000112	0.002000	0.001562
BP	255	regulation of carbohydrate biosynthetic process	7/245	10	3	42	34
	GO:1900			103/232	0.000112	0.002000	0.001562
BP	542	regulation of purine nucleotide metabolic process	7/245	10	3	42	34
	GO:0022			262/232	0.000114	0.002033	0.001587
BP	898	regulation of transmembrane transporter activity	11/245	10	81	2	95
	GO:0006			46/2321	0.000121	0.002120	0.001656
BP	110	regulation of glycolytic process	5/245	0	15	74	31
	GO:0030			46/2321	0.000121	0.002120	0.001656
BP	811	regulation of nucleotide catabolic process	5/245	0	15	74	31
BP	GO:0035	multicellular organismal signaling	8/245	141/232	0.000130	0.002254	0.001760

	637			10	26	46	75
	GO:0055			141/232	0.000130	0.002254	0.001760
BP	088	lipid homeostasis	8/245	10	26	46	75
	GO:0051			10/2321	0.000131	0.002271	0.001773
BP	791	medium-chain fatty acid metabolic process	3/245	0	99	42	99
	GO:0030			75/2321	0.000142	0.002400	0.001874
BP	808	regulation of nucleotide biosynthetic process	6/245	0	59	15	53
	GO:0051			75/2321	0.000142	0.002400	0.001874
BP	193	regulation of cofactor metabolic process	6/245	0	59	15	53
	GO:1900			75/2321	0.000142	0.002400	0.001874
BP	371	regulation of purine nucleotide biosynthetic process	6/245	0	59	15	53
	GO:0006			107/232	0.000142	0.002400	0.001874
BP	140	regulation of nucleotide metabolic process	7/245	10	62	15	53
	GO:0055			143/232	0.000143	0.002403	0.001877
BP	007	cardiac muscle cell differentiation	8/245	10	61	49	14
	GO:0051			316/232	0.000145	0.002423	0.001892
BP	146	striated muscle cell differentiation	12/245	10	57	02	39
	GO:1903			48/2321	0.000148	0.002462	0.001923
BP	115	regulation of actin filament-based movement	5/245	0	76	59	3
	GO:0090			26/2321	0.000150	0.002483	0.001939
BP	208	positive regulation of triglyceride metabolic process	4/245	0	86	95	99
	GO:0009			272/232	0.000159	0.002596	0.002027
BP	167	purine ribonucleoside monophosphate metabolic process	11/245	10	38	06	54
	GO:0032			272/232	0.000159	0.002596	0.002027
BP	409	regulation of transporter activity	11/245	10	38	06	54
BP	GO:0009	purine nucleoside monophosphate metabolic process	11/245	273/232	0.000164	0.002666	0.002082

	126			10	55	04	2
	GO:0051			322/232	0.000173	0.002784	0.002174
BP	235	maintenance of location	12/245	10	29	56	76
	GO:0009			147/232	0.000173	0.002784	0.002174
BP	206	purine ribonucleoside triphosphate biosynthetic process	8/245	10	69	56	76
	GO:0036			230/232	0.000176	0.002816	0.002199
BP	293	response to decreased oxygen levels	10/245	10	59	16	44
	GO:0009			276/232	0.000180	0.002857	0.002231
BP	161	ribonucleoside monophosphate metabolic process	11/245	10	93	14	44
	GO:0009			148/232	0.000181	0.002857	0.002231
BP	145	purine nucleoside triphosphate biosynthetic process	8/245	10	97	14	44
	GO:1901			148/232	0.000181	0.002857	0.002231
BP	292	nucleoside phosphate catabolic process	8/245	10	97	14	44
	GO:0009			150/232	0.000199	0.003116	0.002433
BP	201	ribonucleoside triphosphate biosynthetic process	8/245	10	5	35	89
	GO:0006			234/232	0.000202	0.003142	0.002454
BP	813	potassium ion transport	10/245	10	98	23	1
	GO:0051			28/2321	0.000203	0.003142	0.002454
BP	194	positive regulation of cofactor metabolic process	4/245	0	22	23	1
	GO:0043			52/2321	0.000218	0.003356	0.002621
BP	268	positive regulation of potassium ion transport	5/245	0	18	41	38
	GO:0006			29/2321	0.000233	0.003512	0.002742
BP	536	glutamate metabolic process	4/245	0	8	03	92
	GO:0070			29/2321	0.000233	0.003512	0.002742
BP	296	sarcoplasmic reticulum calcium ion transport	4/245	0	8	03	92
BP	GO:0090	regulation of cholesterol metabolic process	4/245	29/2321	0.000233	0.003512	0.002742

	181			0	8	03	92
	GO:0099			29/2321	0.000233	0.003512	0.002742
BP	622	cardiac muscle cell membrane repolarization	4/245	0	8	03	92
	GO:0051			383/232	0.000234	0.003512	0.002742
BP	260	protein homooligomerization	13/245	10	06	03	92
	GO:0010			12/2321	0.000238	0.003515	0.002745
BP	889	regulation of sequestering of triglyceride	3/245	0	22	24	43
	GO:0009			285/232	0.000238	0.003515	0.002745
BP	123	nucleoside monophosphate metabolic process	11/245	10	61	24	43
	GO:0006			154/232	0.000238	0.003515	0.002745
BP	937	regulation of muscle contraction	8/245	10	74	24	43
	GO:0042			195/232	0.000238	0.003515	0.002745
BP	180	cellular ketone metabolic process	9/245	10	89	24	43
	GO:0043			117/232	0.000248	0.003631	0.002836
BP	500	muscle adaptation	7/245	10	15	67	36
	GO:0006			83/2321	0.000249	0.003631	0.002836
BP	942	regulation of striated muscle contraction	6/245	0	19	67	36
	GO:0045			157/232	0.000272	0.003947	0.003082
BP	834	positive regulation of lipid metabolic process	8/245	10	14	32	89
	GO:0009			158/232	0.000284	0.004062	0.003172
BP	127	purine nucleoside monophosphate biosynthetic process	8/245	10	09	57	9
	GO:0009			158/232	0.000284	0.004062	0.003172
BP	168	purine ribonucleoside monophosphate biosynthetic process	8/245	10	09	57	9
	GO:0046			158/232	0.000284	0.004062	0.003172
BP	496	nicotinamide nucleotide metabolic process	8/245	10	09	57	9
BP	GO:0002	regulation of the force of heart contraction	4/245	31/2321	0.000304	0.004316	0.003370

	026			0	65	06	87
	GO:0086			31/2321	0.000304	0.004316	0.003370
BP	010	membrane depolarization during action potential	4/245	0	65	06	87
	GO:0009			160/232	0.000309	0.004321	0.003374
BP	142	nucleoside triphosphate biosynthetic process	8/245	10	27	23	91
	GO:0019			160/232	0.000309	0.004321	0.003374
BP	362	pyridine nucleotide metabolic process	8/245	10	27	23	91
	GO:1903			160/232	0.000309	0.004321	0.003374
BP	169	regulation of calcium ion transmembrane transport	8/245	10	27	23	91
	GO:0009			162/232	0.000336	0.004676	0.003652
BP	156	ribonucleoside monophosphate biosynthetic process	8/245	10	24	67	51
	GO:0060			32/2321	0.000345	0.004759	0.003716
BP	306	regulation of membrane repolarization	4/245	0	3	2	97
	GO:2001			32/2321	0.000345	0.004759	0.003716
BP	171	positive regulation of ATP biosynthetic process	4/245	0	3	2	97
	GO:0045			163/232	0.000350	0.004808	0.003755
BP	333	cellular respiration	8/245	10	43	16	2
	GO:0055			207/232	0.000370	0.005064	0.003955
BP	001	muscle cell development	9/245	10	78	51	42
	GO:0072			165/232	0.000380	0.005171	0.004038
BP	524	pyridine-containing compound metabolic process	8/245	10	27	04	62
	GO:0006			90/2321	0.000386	0.005206	0.004066
BP	096	glycolytic process	6/245	0	61	93	65
	GO:0045			90/2321	0.000386	0.005206	0.004066
BP	833	negative regulation of lipid metabolic process	6/245	0	61	93	65
BP	GO:0010	regulation of cardiac muscle contraction by calcium ion signaling	3/245	14/2321	0.000388	0.005206	0.004066

	882			0	04	93	65
	GO:0006			91/2321	0.000410	0.005453	0.004258
BP	757	ATP generation from ADP	6/245	0	3	18	97
	GO:1903			91/2321	0.000410	0.005453	0.004258
BP	578	regulation of ATP metabolic process	6/245	0	3	18	97
	GO:0046			210/232	0.000411	0.005453	0.004258
BP	434	organophosphate catabolic process	9/245	10	76	18	97
	GO:0009			168/232	0.000428	0.005655	0.004416
BP	124	nucleoside monophosphate biosynthetic process	8/245	10	89	4	91
	GO:0097			61/2321	0.000462	0.006040	0.004717
BP	006	regulation of plasma lipoprotein particle levels	5/245	0	4	18	42
	GO:0046			170/232	0.000464	0.006040	0.004717
BP	890	regulation of lipid biosynthetic process	8/245	10	02	18	42
	GO:0060			170/232	0.000464	0.006040	0.004717
BP	401	cytosolic calcium ion transport	8/245	10	02	18	42
	GO:0030			15/2321	0.000481	0.006108	0.004770
BP	730	sequestering of triglyceride	3/245	0	28	23	57
	GO:0035			15/2321	0.000481	0.006108	0.004770
BP	337	fatty-acyl-CoA metabolic process	3/245	0	28	23	57
	GO:0055			15/2321	0.000481	0.006108	0.004770
BP	089	fatty acid homeostasis	3/245	0	28	23	57
	GO:0086			15/2321	0.000481	0.006108	0.004770
BP	014	atrial cardiac muscle cell action potential	3/245	0	28	23	57
	GO:0086			15/2321	0.000481	0.006108	0.004770
BP	026	atrial cardiac muscle cell to AV node cell signaling	3/245	0	28	23	57
BP	GO:0086	atrial cardiac muscle cell to AV node cell communication	3/245	15/2321	0.000481	0.006108	0.004770

	066			0	28	23	57
	GO:0042			94/2321	0.000488	0.006170	0.004819
BP	866	pyruvate biosynthetic process	6/245	0	21	53	23
	GO:0034			172/232	0.000501	0.006311	0.004929
BP	404	nucleobase-containing small molecule biosynthetic process	8/245	10	45	62	42
	GO:2001			63/2321	0.000537	0.006732	0.005257
BP	169	regulation of ATP biosynthetic process	5/245	0	06	08	8
	GO:0006			314/232	0.000540	0.006749	0.005271
BP	066	alcohol metabolic process	11/245	10	67	53	43
	GO:0006			36/2321	0.000547	0.006775	0.005292
BP	458	'de novo' protein folding	4/245	0	24	93	05
	GO:0071			36/2321	0.000547	0.006775	0.005292
BP	398	cellular response to fatty acid	4/245	0	24	93	05
	GO:0051			134/232	0.000564	0.006963	0.005438
BP	928	positive regulation of calcium ion transport	7/245	10	66	41	47
	GO:0043			97/2321	0.000577	0.007089	0.005536
BP	502	regulation of muscle adaptation	6/245	0	19	18	7
	GO:0006			16/2321	0.000587		0.005592
BP	103	2-oxoglutarate metabolic process	3/245	0	74	0.007161	79
	GO:1903			16/2321	0.000587		0.005592
BP	779	regulation of cardiac conduction	3/245	0	74	0.007161	79
	GO:0006			177/232	0.000605	0.007309	0.005708
BP	733	oxidoreduction coenzyme metabolic process	8/245	10	82	01	39
	GO:0030			37/2321	0.000608	0.007309	0.005708
BP	810	positive regulation of nucleotide biosynthetic process	4/245	0	52	01	39
BP	GO:1900	positive regulation of purine nucleotide biosynthetic process	4/245	37/2321	0.000608	0.007309	0.005708

	373			0	52	01	39
	GO:0046			98/2321	0.000609	0.007309	0.005708
BP	031	ADP metabolic process	6/245	0	48	01	39
	GO:0001			179/232	0.000652	0.007760	0.006060
BP	659	temperature homeostasis	8/245	10	19	02	63
	GO:0006			179/232	0.000652	0.007760	0.006060
BP	839	mitochondrial transport	8/245	10	19	02	63
	GO:0051			272/232	0.000664	0.007871	0.006147
BP	924	regulation of calcium ion transport	10/245	10	17	86	97
	GO:0010			17/2321	0.000708	0.008296	0.006479
BP	867	positive regulation of triglyceride biosynthetic process	3/245	0	14	09	31
	GO:0042			17/2321	0.000708	0.008296	0.006479
BP	135	neurotransmitter catabolic process	3/245	0	14	09	31
	GO:0086			17/2321	0.000708	0.008296	0.006479
BP	012	membrane depolarization during cardiac muscle cell action potential	3/245	0	14	09	31
	GO:0046			325/232	0.000718	0.008382	0.006546
BP	486	glycerolipid metabolic process	11/245	10	22	01	41
	GO:0031			275/232	0.000722	0.008399	0.006560
BP	647	regulation of protein stability	10/245	10	51	87	36
	GO:0035			182/232	0.000727	0.008420	0.006576
BP	051	cardiocyte differentiation	8/245	10	08	82	72
	GO:1901			39/2321	0.000745	0.008600	0.006717
BP	021	positive regulation of calcium ion transmembrane transporter activity	4/245	0	44	75	25
	GO:0006			103/232	0.000792	0.009041	0.007061
BP	165	nucleoside diphosphate phosphorylation	6/245	10	54	51	48
BP	GO:0009	purine nucleoside diphosphate metabolic process	6/245	103/232	0.000792	0.009041	0.007061

	135			10	54	51	48
	GO:0009			103/232	0.000792	0.009041	0.007061
BP	179	purine ribonucleoside diphosphate metabolic process	6/245	10	54	51	48
	GO:0072			279/232	0.000806	0.009169	0.007161
BP	659	protein localization to plasma membrane	10/245	10	81	91	77
	GO:0070			280/232	0.000829	0.009388	0.007332
BP	588	calcium ion transmembrane transport	10/245	10	1	26	29
	GO:0046			18/2321	0.000843	0.009512	0.007429
BP	321	positive regulation of fatty acid oxidation	3/245	0	16	14	05
	GO:0019			70/2321	0.000871	0.009780	0.007638
BP	915	lipid storage	5/245	0	13	77	85
	GO:0070			282/232	0.000875	0.009780	0.007638
BP	482	response to oxygen levels	10/245	10	21	77	85
	GO:0046			105/232	0.000876	0.009780	0.007638
BP	939	nucleotide phosphorylation	6/245	10	61	77	85
	GO:0042			41/2321	0.000902	0.009999	0.007809
BP	304	regulation of fatty acid biosynthetic process	4/245	0	79	65	8
	GO:0086			41/2321	0.000902	0.009999	0.007809
BP	009	membrane repolarization	4/245	0	79	65	8
	GO:0019			106/232	0.000921	0.010165	0.007939
BP	359	nicotinamide nucleotide biosynthetic process	6/245	10	14	88	62
	GO:0009			107/232	0.000967	0.010637	0.008308
BP	185	ribonucleoside diphosphate metabolic process	6/245	10	39	74	15
	GO:0010			42/2321	0.000989	0.010804	0.008438
BP	883	regulation of lipid storage	4/245	0	63	37	29
BP	GO:0045	positive regulation of fatty acid metabolic process	4/245	42/2321	0.000989	0.010804	0.008438

	923			0	63	37	29
	GO:0031			19/2321	0.000993	0.010807	0.008440
BP	998	regulation of fatty acid beta-oxidation	3/245	0	49	7	89
	GO:0014			449/232	0.001035	0.011227	0.008768
BP	706	striated muscle tissue development	13/245	10	75	37	66
	GO:0019			109/232	0.001065	0.011505	0.008986
BP	363	pyridine nucleotide biosynthetic process	6/245	10	22	91	2
	GO:0015			150/232	0.001097	0.011817	0.009229
BP	718	monocarboxylic acid transport	7/245	10	95	53	57
	GO:0032			75/2321	0.001190	0.012767	0.009971
BP	543	mitochondrial translation	5/245	0	44	9	82
	GO:0006			153/232	0.001231	0.013163	0.010280
BP	457	protein folding	7/245	10	62	24	58
	GO:0072			113/232	0.001283	0.013592	0.010615
BP	525	pyridine-containing compound biosynthetic process	6/245	10	53	69	99
	GO:0090			45/2321	0.001285	0.013592	0.010615
BP	207	regulation of triglyceride metabolic process	4/245	0	19	69	99
	GO:1903			45/2321	0.001285	0.013592	0.010615
BP	580	positive regulation of ATP metabolic process	4/245	0	19	69	99
	GO:2001			77/2321	0.001339	0.014101	0.011013
BP	259	positive regulation of cation channel activity	5/245	0	75	25	18
	GO:0010			21/2321	0.001342	0.014101	0.011013
BP	866	regulation of triglyceride biosynthetic process	3/245	0	54	25	18
	GO:0044			463/232	0.001364	0.014277	0.011151
BP	270	cellular nitrogen compound catabolic process	13/245	10	03	81	07
BP	GO:0046	carboxylic acid transport	10/245	301/232	0.001427	0.014893	0.011631

	942			10	72	3	77
	GO:0015			302/232	0.001463	0.015211	0.011880
BP	849	organic acid transport	10/245	10	25	78	51
	GO:0007			357/232	0.001528	0.015788	0.012331
BP	178	transmembrane receptor protein serine/threonine kinase signaling pathway	11/245	10	92	71	09
	GO:0046			469/232	0.001529	0.015788	0.012331
BP	700	heterocycle catabolic process	13/245	10	11	71	09
	GO:0007			254/232	0.001579	0.016253	0.012694
BP	626	locomotory behavior	9/245	10	46	51	1
	GO:0060			472/232	0.001617	0.016590	0.012957
BP	537	muscle tissue development	13/245	10	67	69	45
	GO:0010			48/2321	0.001637	0.016737	0.013072
BP	107	potassium ion import	4/245	0	48	42	04
	GO:0006			473/232	0.001648	0.016789	0.013113
BP	874	cellular calcium ion homeostasis	13/245	10	12	93	05
	GO:0014			49/2321	0.001768	0.017925	0.013999
BP	888	striated muscle adaptation	4/245	0	43	28	77
	GO:0009			163/232	0.001771	0.017925	0.013999
BP	266	response to temperature stimulus	7/245	10	34	28	77
	GO:0009			121/232	0.001821	0.018368	0.014345
BP	132	nucleoside diphosphate metabolic process	6/245	10	15	28	76
	GO:0048			260/232	0.001852	0.018620	0.014542
BP	738	cardiac muscle tissue development	9/245	10	25	3	58
	GO:1901			83/2321	0.001870	0.018745	
BP	019	regulation of calcium ion transmembrane transporter activity	5/245	0	81	03	0.01464
BP	GO:0006	calcium ion transport	12/245	423/232	0.001898	0.018881	0.014746

	816			10	7	9	9
	GO:0045			50/2321	0.001906	0.018881	0.014746
BP	981	positive regulation of nucleotide metabolic process	4/245	0	47	9	9
	GO:1900			50/2321	0.001906	0.018881	0.014746
BP	544	positive regulation of purine nucleotide metabolic process	4/245	0	47	9	9
	GO:1901			481/232	0.001909	0.018881	0.014746
BP	615	organic hydroxy compound metabolic process	13/245	10	27	9	9
	GO:1901			84/2321	0.001972	0.019442	0.015185
BP	379	regulation of potassium ion transmembrane transport	5/245	0	38	95	08
	GO:0009			24/2321	0.001995	0.019611	0.015317
BP	065	glutamine family amino acid catabolic process	3/245	0	96	95	07
	GO:0070			51/2321	0.002051	0.020095	0.015694
BP	542	response to fatty acid	4/245	0	8	74	92
	GO:0070			86/2321	0.002187	0.021318	0.016649
BP	509	calcium ion import	5/245	0	52	13	61
	GO:0051			374/232	0.002199	0.021318	0.016649
BP	480	regulation of cytosolic calcium ion concentration	11/245	10	09	13	61
	GO:0055			489/232	0.002203	0.021318	0.016649
BP	074	calcium ion homeostasis	13/245	10	71	13	61
	GO:0061			52/2321	0.002204	0.021318	0.016649
BP	077	chaperone-mediated protein folding	4/245	0	6	13	61
	GO:0001			170/232	0.002247	0.021489	0.016783
BP	890	placenta development	7/245	10	22	94	79
	GO:1901			25/2321	0.002250	0.021489	0.016783
BP	032	negative regulation of response to reactive oxygen species	3/245	0	59	94	79
BP	GO:1903	negative regulation of hydrogen peroxide-induced cell death	3/245	25/2321	0.002250	0.021489	0.016783

	206			0	59	94	79
	GO:2001			25/2321	0.002250	0.021489	0.016783
BP	039	negative regulation of cellular response to drug	3/245	0	59	94	79
	GO:0043			171/232	0.002322	0.022107	0.017266
BP	271	negative regulation of ion transport	7/245	10	55	75	31
	GO:0043			324/232	0.002445	0.023205	0.018123
BP	434	response to peptide hormone	10/245	10	48	42	6
	GO:0003			26/2321	0.002524	0.023537	0.018382
BP	299	muscle hypertrophy in response to stress	3/245	0	46	55	99
	GO:0014			26/2321	0.002524	0.023537	0.018382
BP	898	cardiac muscle hypertrophy in response to stress	3/245	0	46	55	99
	GO:0045			26/2321	0.002524	0.023537	0.018382
BP	117	azole transport	3/245	0	46	55	99
	GO:0098			26/2321	0.002524	0.023537	0.018382
BP	901	regulation of cardiac muscle cell action potential	3/245	0	46	55	99
	GO:1902			26/2321	0.002524	0.023537	0.018382
BP	175	regulation of oxidative stress-induced intrinsic apoptotic signaling pathway	3/245	0	46	55	99
	GO:0046			54/2321	0.002533	0.023537	0.018382
BP	503	glycerolipid catabolic process	4/245	0	4	55	99
	GO:0072			497/232	0.002534	0.023537	0.018382
BP	503	cellular divalent inorganic cation homeostasis	13/245	10	57	55	99
	GO:0042			439/232	0.002574	0.023835	0.018615
BP	391	regulation of membrane potential	12/245	10	5	63	8
	GO:1901			384/232	0.002693	0.024862	0.019418
BP	652	response to peptide	11/245	10	63	98	16
BP	GO:0010	regulation of cardiac muscle cell apoptotic process	4/245	55/2321	0.002709	0.024936	0.019475

	665			0	77	44	53
	GO:0016			176/232	0.002728	0.025036	0.019553
BP	202	regulation of striated muscle tissue development	7/245	10	83	17	43
	GO:0006			330/232	0.002789	0.025515	0.019927
BP	869	lipid transport	10/245	10	48	76	99
	GO:0014			27/2321	0.002818	0.025623	0.020012
BP	887	cardiac muscle adaptation	3/245	0	07	38	04
	GO:1901			27/2321	0.002818	0.025623	0.020012
BP	018	positive regulation of potassium ion transmembrane transporter activity	3/245	0	07	38	04
	GO:0065			56/2321	0.002894	0.026238	0.020492
BP	002	intracellular protein transmembrane transport	4/245	0	38	91	78
	GO:1901			179/232	0.002997	0.027093	0.021160
BP	861	regulation of muscle tissue development	7/245	10	51	25	02
	GO:0001			93/2321	0.003076	0.027533	0.021503
BP	676	long-chain fatty acid metabolic process	5/245	0	16	61	94
	GO:0046			93/2321	0.003076	0.027533	0.021503
BP	889	positive regulation of lipid biosynthetic process	5/245	0	16	61	94
	GO:0050			57/2321	0.003087	0.027533	0.021503
BP	994	regulation of lipid catabolic process	4/245	0	4	61	94
	GO:0071			57/2321	0.003087	0.027533	0.021503
BP	806	protein transmembrane transport	4/245	0	4	61	94
	GO:0048			180/232	0.003091	0.027533	0.021503
BP	634	regulation of muscle organ development	7/245	10	43	61	94
	GO:0005			28/2321	0.003131	0.027731	0.021658
BP	979	regulation of glycogen biosynthetic process	3/245	0	85	43	44
BP	GO:0010	regulation of glucan biosynthetic process	3/245	28/2321	0.003131	0.027731	0.021658

	962			0	85	43	44
	GO:0010			58/2321	0.003289	0.028954	0.022613
BP	662	regulation of striated muscle cell apoptotic process	4/245	0	03	91	99
	GO:0098			58/2321	0.003289	0.028954	0.022613
BP	900	regulation of action potential	4/245	0	03	91	99
	GO:0010			59/2321	0.003499	0.030718	0.023991
BP	659	cardiac muscle cell apoptotic process	4/245	0	45	51	37
	GO:0071			96/2321	0.003527	0.030783	0.024042
BP	456	cellular response to hypoxia	5/245	0	08	63	23
	GO:0120			96/2321	0.003527	0.030783	0.024042
BP	162	positive regulation of cold-induced thermogenesis	5/245	0	08	63	23
	GO:1990			344/232	0.003743	0.032580	0.025445
BP	778	protein localization to cell periphery	10/245	10	62	15	33
	GO:0042			30/2321	0.003821	0.033070	0.025828
BP	026	protein refolding	3/245	0	65	3	14
	GO:0051			30/2321	0.003821	0.033070	0.025828
BP	085	chaperone cofactor-dependent protein refolding	3/245	0	65	3	14
	GO:0045			141/232	0.003903	0.033680	0.026304
BP	017	glycerolipid biosynthetic process	6/245	10	24	63	81
	GO:1905			61/2321	0.003947	0.033965	0.026526
BP	207	regulation of cardiocyte differentiation	4/245	0	35	05	94
	GO:0071			142/232	0.004040	0.034472	0.026923
BP	331	cellular response to hexose stimulus	6/245	10	3	68	4
	GO:0106			142/232	0.004040	0.034472	0.026923
BP	106	cold-induced thermogenesis	6/245	10	3	68	4
BP	GO:0120	regulation of cold-induced thermogenesis	6/245	142/232	0.004040	0.034472	0.026923

	161			10	3	68	4
	GO:0071			143/232	0.004180	0.035143	0.027447
BP	326	cellular response to monosaccharide stimulus	6/245	10	89	47	3
	GO:0010			62/2321	0.004185	0.035143	0.027447
BP	658	striated muscle cell apoptotic process	4/245	0	19	47	3
	GO:0001			31/2321	0.004198	0.035143	0.027447
BP	832	blastocyst growth	3/245	0	47	47	3
	GO:0006			31/2321	0.004198	0.035143	0.027447
BP	099	tricarboxylic acid cycle	3/245	0	47	47	3
	GO:0010			31/2321	0.004198	0.035143	0.027447
BP	614	negative regulation of cardiac muscle hypertrophy	3/245	0	47	47	3
	GO:0090			31/2321	0.004198	0.035143	0.027447
BP	075	relaxation of muscle	3/245	0	47	47	3
	GO:0055			100/232	0.004199	0.035143	0.027447
BP	013	cardiac muscle cell development	5/245	10	68	47	3
	GO:2000			101/232	0.004381	0.036561	0.028555
BP	300	regulation of synaptic vesicle exocytosis	5/245	10	18	86	07
	GO:0006			297/232	0.004473	0.037233	0.029079
BP	836	neurotransmitter transport	9/245	10	91	7	78
	GO:0055			193/232	0.004528	0.037583	0.029353
BP	002	striated muscle cell development	7/245	10	33	95	33
	GO:0010			102/232	0.004568	0.037811	0.029531
BP	522	regulation of calcium ion transport into cytosol	5/245	10	21	83	31
	GO:0070			472/232	0.004589	0.037845	0.029557
BP	838	divalent metal ion transport	12/245	10	29	12	3
BP	GO:0006	citrate metabolic process	3/245	32/2321	0.004597	0.037845	0.029557

	101			0	08	12	3
	GO:0031			355/232	0.004661	0.038257	0.029879
BP	331	positive regulation of cellular catabolic process	10/245	10	37	29	21
	GO:0010			64/2321	0.004689	0.038257	0.029879
BP	676	positive regulation of cellular carbohydrate metabolic process	4/245	0	51	29	21
	GO:0006			10/2321	0.004722	0.038257	0.029879
BP	560	proline metabolic process	2/245	0	5	29	21
	GO:0015			10/2321	0.004722	0.038257	0.029879
BP	937	coenzyme A biosynthetic process	2/245	0	5	29	21
	GO:0019			10/2321	0.004722	0.038257	0.029879
BP	374	galactolipid metabolic process	2/245	0	5	29	21
	GO:0035			10/2321	0.004722	0.038257	0.029879
BP	811	negative regulation of urine volume	2/245	0	5	29	21
	GO:0017			103/232	0.004760	0.038363	0.029962
BP	015	regulation of transforming growth factor beta receptor signaling pathway	5/245	10	84	82	41
	GO:0140			103/232	0.004760	0.038363	0.029962
BP	053	mitochondrial gene expression	5/245	10	84	82	41
	GO:0015			415/232	0.004821	0.038652	0.030187
BP	711	organic anion transport	11/245	10	79	43	82
	GO:0072			475/232	0.004822	0.038652	0.030187
BP	511	divalent inorganic cation transport	12/245	10	04	43	82
	GO:0009			65/2321	0.004956	0.039624	0.030947
BP	064	glutamine family amino acid metabolic process	4/245	0	34	68	15
	GO:0010			33/2321	0.005017	0.039699	0.031005
BP	667	negative regulation of cardiac muscle cell apoptotic process	3/245	0	81	39	5
BP	GO:0014	negative regulation of muscle hypertrophy	3/245	33/2321	0.005017	0.039699	0.031005

	741			0	81	39	5
	GO:0021			33/2321	0.005017	0.039699	0.031005
BP	680	cerebellar Purkinje cell layer development	3/245	0	81	39	5
	GO:1903			33/2321	0.005017	0.039699	0.031005
BP	205	regulation of hydrogen peroxide-induced cell death	3/245	0	81	39	5
	GO:0043			105/232	0.005163	0.040639	0.031739
BP	266	regulation of potassium ion transport	5/245	10	31	37	63
	GO:1903			105/232	0.005163	0.040639	0.031739
BP	844	regulation of cellular response to transforming growth factor beta stimulus	5/245	10	31	37	63
	GO:0071			150/232	0.005268	0.041359	0.032302
BP	322	cellular response to carbohydrate stimulus	6/245	10	41	76	26
	GO:0055			106/232	0.005373	0.041966	0.032776
BP	006	cardiac cell development	5/245	10	32	97	5
	GO:1902			106/232	0.005373	0.041966	0.032776
BP	803	regulation of synaptic vesicle transport	5/245	10	32	97	5
	GO:0002			34/2321	0.005461	0.042434	0.033141
BP	931	response to ischemia	3/245	0	02	36	53
	GO:0071			34/2321	0.005461	0.042434	0.033141
BP	634	regulation of transforming growth factor beta production	3/245	0	02	36	53
	GO:0019			483/232	0.005489	0.042544	0.033227
BP	439	aromatic compound catabolic process	12/245	10	12	17	29
	GO:0006			67/2321	0.005520	0.042676	0.033330
BP	940	regulation of smooth muscle contraction	4/245	0	14	04	29
	GO:0003			107/232	0.005589	0.043101	0.033662
BP	014	renal system process	5/245	10	29	23	36
BP	GO:0007	muscle organ development	11/245	424/232	0.005641	0.043391	0.033888

	517			10	13	15	79
	GO:0006			11/2321	0.005731	0.043590	0.034044
BP	002	fructose 6-phosphate metabolic process	2/245	0	93	2	25
	GO:0016			11/2321	0.005731	0.043590	0.034044
BP	188	synaptic vesicle maturation	2/245	0	93	2	25
	GO:0071			11/2321	0.005731	0.043590	0.034044
BP	415	cellular response to purine-containing compound	2/245	0	93	2	25
	GO:1902	regulation of calcium ion transmembrane transport via high voltage-gated		11/2321	0.005731	0.043590	0.034044
BP	514	calcium channel	2/245	0	93	2	25
	GO:0034			425/232	0.005738	0.043590	0.034044
BP	655	nucleobase-containing compound catabolic process	11/245	10	57	2	25
	GO:0019			35/2321	0.005927	0.044249	0.034558
BP	432	triglyceride biosynthetic process	3/245	0	01	2	94
	GO:0034			35/2321	0.005927	0.044249	0.034558
BP	381	plasma lipoprotein particle clearance	3/245	0	01	2	94
	GO:0046			35/2321	0.005927	0.044249	0.034558
BP	461	neutral lipid catabolic process	3/245	0	01	2	94
	GO:0046			35/2321	0.005927	0.044249	0.034558
BP	464	acylglycerol catabolic process	3/245	0	01	2	94
	GO:0051			35/2321	0.005927	0.044249	0.034558
BP	084	'de novo' posttranslational protein folding	3/245	0	01	2	94
	GO:0070			35/2321	0.005927	0.044249	0.034558
BP	873	regulation of glycogen metabolic process	3/245	0	01	2	94
	GO:2001			35/2321	0.005927	0.044249	0.034558
BP	038	regulation of cellular response to drug	3/245	0	01	2	94
BP	GO:0071	cellular response to transforming growth factor beta stimulus	7/245	204/232	0.006096	0.045402	0.035459

	560			10	43	72	84
	GO:0007			69/2321	0.006125	0.045506	0.035540
BP	492	endoderm development	4/245	0	25	09	58
	GO:1990			156/232	0.006354	0.047094	0.036780
BP	845	adaptive thermogenesis	6/245	10	46	12	84
	GO:0010			36/2321	0.006416	0.047110	0.036793
BP	664	negative regulation of striated muscle cell apoptotic process	3/245	0	08	57	68
	GO:0071			36/2321	0.006416	0.047110	0.036793
BP	604	transforming growth factor beta production	3/245	0	08	57	68
	GO:1905			36/2321	0.006416	0.047110	0.036793
BP	209	positive regulation of cardiocyte differentiation	3/245	0	08	57	68
	GO:0001			372/232	0.006418	0.047110	0.036793
BP	505	regulation of neurotransmitter levels	10/245	10	54	57	68
	GO:0060			157/232	0.006550	0.047961	0.037457
BP	402	calcium ion transport into cytosol	6/245	10	16	03	9
	GO:0071			208/232	0.006756	0.048386	0.037790
BP	559	response to transforming growth factor beta	7/245	10	78	58	26
	GO:0001			112/232	0.006761	0.048386	0.037790
BP	892	embryonic placenta development	5/245	10	77	58	26
	GO:0006			112/232	0.006761	0.048386	0.037790
BP	939	smooth muscle contraction	5/245	10	77	58	26
	GO:0010			71/2321	0.006772	0.048386	0.037790
BP	611	regulation of cardiac muscle hypertrophy	4/245	0	9	58	26
	GO:0000			12/2321	0.006830	0.048386	0.037790
BP	083	regulation of transcription involved in G1/S transition of mitotic cell cycle	2/245	0	67	58	26
BP	GO:0003	renal water homeostasis	2/245	12/2321	0.006830	0.048386	0.037790

	091			0	67	58	26
	GO:0006			12/2321	0.006830	0.048386	0.037790
BP	086	acetyl-CoA biosynthetic process from pyruvate	2/245	0	67	58	26
	GO:0006			12/2321	0.006830	0.048386	0.037790
BP	654	phosphatidic acid biosynthetic process	2/245	0	67	58	26
	GO:0009			12/2321	0.006830	0.048386	0.037790
BP	068	aspartate family amino acid catabolic process	2/245	0	67	58	26
	GO:0030			12/2321	0.006830	0.048386	0.037790
BP	388	fructose 1,6-bisphosphate metabolic process	2/245	0	67	58	26
	GO:0043			12/2321	0.006830	0.048386	0.037790
BP	471	regulation of cellular carbohydrate catabolic process	2/245	0	67	58	26
	GO:0045			12/2321	0.006830	0.048386	0.037790
BP	717	negative regulation of fatty acid biosynthetic process	2/245	0	67	58	26
	GO:0061			12/2321	0.006830	0.048386	0.037790
BP	577	calcium ion transmembrane transport via high voltage-gated calcium channel	2/245	0	67	58	26
	GO:1901			12/2321	0.006830	0.048386	0.037790
BP	298	regulation of hydrogen peroxide-mediated programmed cell death	2/245	0	67	58	26
	GO:0032			37/2321	0.006928	0.048739	0.038065
BP	885	regulation of polysaccharide biosynthetic process	3/245	0	51	61	97
	GO:0036			37/2321	0.006928	0.048739	0.038065
BP	474	cell death in response to hydrogen peroxide	3/245	0	51	61	97
	GO:0072			37/2321	0.006928	0.048739	0.038065
BP	350	tricarboxylic acid metabolic process	3/245	0	51	61	97
	GO:0001			159/232	0.006954	0.048810	0.038121
BP	678	cellular glucose homeostasis	6/245	10	64	69	49
BP	GO:0051	protein heterooligomerization	6/245	160/232	0.007163	0.050161	0.039176

	291			10	53	17	23
BP	GO:0060419	heart growth	5/245	114/23210	0.0072756	0.05082905	0.03969784
BP	GO:0042593	glucose homeostasis	8/245	265/23210	0.00735615	0.05127421	0.04004552
BP	GO:0035690	cellular response to drug	10/245	380/23210	0.00740591	0.05132523	0.04008536
BP	GO:0006094	gluconeogenesis	4/245	73/23210	0.00746433	0.05132523	0.04008536
BP	GO:0060191	regulation of lipase activity	4/245	73/23210	0.00746433	0.05132523	0.04008536
BP	GO:1990542	mitochondrial transmembrane transport	4/245	73/23210	0.00746433	0.05132523	0.04008536
BP	GO:0046460	neutral lipid biosynthetic process	3/245	38/23210	0.00746457	0.05132523	0.04008536
BP	GO:0046463	acylglycerol biosynthetic process	3/245	38/23210	0.00746457	0.05132523	0.04008536
BP	GO:0033500	carbohydrate homeostasis	8/245	266/23210	0.00751722	0.05157086	0.04027721
BP	GO:0007193	adenylate cyclase-inhibiting G protein-coupled receptor signaling pathway	4/245	74/23210	0.00782683	0.05333451	0.04165463
BP	GO:0014743	regulation of muscle hypertrophy	4/245	74/23210	0.00782683	0.05333451	0.04165463
BP	GO:1903201	regulation of oxidative stress-induced cell death	4/245	74/23210	0.00782683	0.05333451	0.04165463
BP	GO:0015	basic amino acid transport	2/245	13/2321	0.008016	0.053957	0.042140

	802			0	74	21	96
	GO:0034			13/2321	0.008016	0.053957	0.042140
BP	982	mitochondrial protein processing	2/245	0	74	21	96
	GO:0050			13/2321	0.008016	0.053957	0.042140
BP	746	regulation of lipoprotein metabolic process	2/245	0	74	21	96
	GO:0071			13/2321	0.008016	0.053957	0.042140
BP	380	cellular response to prostaglandin E stimulus	2/245	0	74	21	96
	GO:0097			39/2321	0.008024	0.053957	0.042140
BP	009	energy homeostasis	3/245	0	5	21	96
	GO:2001			39/2321	0.008024	0.053957	0.042140
BP	024	negative regulation of response to drug	3/245	0	5	21	96
	GO:0007			165/232	0.008276	0.055529	0.043368
BP	179	transforming growth factor beta receptor signaling pathway	6/245	10	57	58	99
	GO:0019			76/2321	0.008586	0.057479	0.044892
BP	319	hexose biosynthetic process	4/245	0	11	79	12
	GO:0042			40/2321	0.008608	0.057503	0.044910
BP	398	cellular modified amino acid biosynthetic process	3/245	0	53	45	6
	GO:0003			41/2321	0.009216	0.059940	0.046813
BP	298	physiological muscle hypertrophy	3/245	0	87	34	83
	GO:0003			41/2321	0.009216	0.059940	0.046813
BP	301	physiological cardiac muscle hypertrophy	3/245	0	87	34	83
	GO:0006			41/2321	0.009216	0.059940	0.046813
BP	418	tRNA aminoacylation for protein translation	3/245	0	87	34	83
	GO:0009			41/2321	0.009216	0.059940	0.046813
BP	066	aspartate family amino acid metabolic process	3/245	0	87	34	83
BP	GO:0061	cell growth involved in cardiac muscle cell development	3/245	41/2321	0.009216	0.059940	0.046813

	049			0	87	34	83
	GO:0036			121/232	0.009288	0.059940	0.046813
BP	294	cellular response to decreased oxygen levels	5/245	10	1	34	83
	GO:0010			14/2321	0.009288	0.059940	0.046813
BP	421	hydrogen peroxide-mediated programmed cell death	2/245	0	19	34	83
	GO:0010			14/2321	0.009288	0.059940	0.046813
BP	896	regulation of triglyceride catabolic process	2/245	0	19	34	83
	GO:0015			14/2321	0.009288	0.059940	0.046813
BP	936	coenzyme A metabolic process	2/245	0	19	34	83
	GO:0043			14/2321	0.009288	0.059940	0.046813
BP	650	dicarboxylic acid biosynthetic process	2/245	0	19	34	83
	GO:0046			14/2321	0.009288	0.059940	0.046813
BP	473	phosphatidic acid metabolic process	2/245	0	19	34	83
	GO:0086			14/2321	0.009288	0.059940	0.046813
BP	069	bundle of His cell to Purkinje myocyte communication	2/245	0	19	34	83
	GO:0090			14/2321	0.009288	0.059940	0.046813
BP	136	epithelial cell-cell adhesion	2/245	0	19	34	83
	GO:0097			14/2321	0.009288	0.059940	0.046813
BP	468	programmed cell death in response to reactive oxygen species	2/245	0	19	34	83
	GO:1901			14/2321	0.009288	0.059940	0.046813
BP	387	positive regulation of voltage-gated calcium channel activity	2/245	0	19	34	83
	GO:1901			14/2321	0.009288	0.059940	0.046813
BP	841	regulation of high voltage-gated calcium channel activity	2/245	0	19	34	83
	GO:0006			122/232	0.009603	0.061846	0.048302
BP	639	acylglycerol metabolic process	5/245	10	88	54	58
BP	GO:0051	regulation of release of sequestered calcium ion into cytosol	4/245	79/2321	0.009812	0.063058	0.049249

	279			0	81	67	26
	GO:0006			225/232		0.065073	0.050822
BP	650	glycerophospholipid metabolic process	7/245	10	0.010169	03	49
	GO:0032			225/232		0.065073	0.050822
BP	868	response to insulin	7/245	10	0.010169	03	49
	GO:0006			124/232	0.010257	0.065501	0.051157
BP	638	neutral lipid metabolic process	5/245	10	46	49	12
	GO:0006			400/232	0.010389	0.066205	0.051706
BP	979	response to oxidative stress	10/245	10	44	51	96
	GO:0008			43/2321	0.010507	0.066400	0.051859
BP	631	intrinsic apoptotic signaling pathway in response to oxidative stress	3/245	0	31	94	6
	GO:0010			43/2321	0.010507	0.066400	0.051859
BP	907	positive regulation of glucose metabolic process	3/245	0	31	94	6
	GO:1901			43/2321	0.010507	0.066400	0.051859
BP	031	regulation of response to reactive oxygen species	3/245	0	31	94	6
	GO:1990			43/2321	0.010507	0.066400	0.051859
BP	573	potassium ion import across plasma membrane	3/245	0	31	94	6
	GO:0009			401/232	0.010559	0.066592	0.052009
BP	991	response to extracellular stimulus	10/245	10	56	98	59
	GO:0021			15/2321	0.010643	0.066705	0.052097
BP	694	cerebellar Purkinje cell layer formation	2/245	0	1	55	5
	GO:0039			15/2321	0.010643	0.066705	0.052097
BP	535	regulation of RIG-I signaling pathway	2/245	0	1	55	5
	GO:1902			15/2321	0.010643	0.066705	0.052097
BP	894	negative regulation of pri-miRNA transcription by RNA polymerase II	2/245	0	1	55	5
BP	GO:0022	respiratory electron transport chain	4/245	81/2321	0.010690	0.066865	0.052222

	904			0	65	93	76
	GO:0046			82/2321	0.011147	0.069348	0.054161
BP	364	monosaccharide biosynthetic process	4/245	0	95	41	59
	GO:0051			82/2321	0.011147	0.069348	0.054161
BP	899	membrane depolarization	4/245	0	95	41	59
	GO:0032			44/2321	0.011189	0.069348	0.054161
BP	881	regulation of polysaccharide metabolic process	3/245	0	75	41	59
	GO:0043			44/2321	0.011189	0.069348	0.054161
BP	039	tRNA aminoacylation	3/245	0	75	41	59
	GO:0009			344/232	0.011201	0.069348	0.054161
BP	636	response to toxic substance	9/245	10	38	41	59
	GO:0051			127/232		0.069779	0.054498
BP	592	response to calcium ion	5/245	10	0.011294	99	66
	GO:0051			178/232	0.011744	0.072409	0.056552
BP	588	regulation of neurotransmitter transport	6/245	10	36	51	34
	GO:0043			45/2321	0.011897	0.072409	0.056552
BP	038	amino acid activation	3/245	0	21	51	34
	GO:0060			45/2321	0.011897	0.072409	0.056552
BP	421	positive regulation of heart growth	3/245	0	21	51	34
	GO:1902			45/2321	0.011897	0.072409	0.056552
BP	893	regulation of pri-miRNA transcription by RNA polymerase II	3/245	0	21	51	34
	GO:0006			348/232	0.012007	0.072409	0.056552
BP	644	phospholipid metabolic process	9/245	10	89	51	34
	GO:0014			16/2321	0.012079	0.072409	0.056552
BP	733	regulation of skeletal muscle adaptation	2/245	0	59	51	34
BP	GO:0031	response to caffeine	2/245	16/2321	0.012079	0.072409	0.056552

	000			0	59	51	34
	GO:0036			16/2321	0.012079	0.072409	0.056552
BP	270	response to diuretic	2/245	0	59	51	34
	GO:0043			16/2321	0.012079	0.072409	0.056552
BP	084	penile erection	2/245	0	59	51	34
	GO:0060			16/2321	0.012079	0.072409	0.056552
BP	192	negative regulation of lipase activity	2/245	0	59	51	34
	GO:0070			16/2321	0.012079	0.072409	0.056552
BP	885	negative regulation of calcineurin-NFAT signaling cascade	2/245	0	59	51	34
	GO:0071			16/2321	0.012079	0.072409	0.056552
BP	379	cellular response to prostaglandin stimulus	2/245	0	59	51	34
	GO:0106			16/2321	0.012079	0.072409	0.056552
BP	057	negative regulation of calcineurin-mediated signaling	2/245	0	59	51	34
	GO:0045			84/2321	0.012099	0.072409	0.056552
BP	844	positive regulation of striated muscle tissue development	4/245	0	95	51	34
	GO:0048			84/2321	0.012099	0.072409	0.056552
BP	636	positive regulation of muscle organ development	4/245	0	95	51	34
	GO:0060			84/2321	0.012099	0.072409	0.056552
BP	420	regulation of heart growth	4/245	0	95	51	34
	GO:0090	regulation of transmembrane receptor protein serine/threonine kinase signaling		233/232	0.012149	0.072560	0.056670
BP	092	pathway	7/245	10	02	61	34
	GO:0022			85/2321	0.012594	0.074493	0.058179
BP	900	electron transport chain	4/245	0	86	11	64
	GO:0098			85/2321	0.012594	0.074493	0.058179
BP	659	inorganic cation import across plasma membrane	4/245	0	86	11	64
BP	GO:0099	inorganic ion import across plasma membrane	4/245	85/2321	0.012594	0.074493	0.058179

	587			0	86	11	64
	GO:1900			85/2321	0.012594	0.074493	0.058179
BP	407	regulation of cellular response to oxidative stress	4/245	0	86	11	64
	GO:1901			85/2321	0.012594	0.074493	0.058179
BP	863	positive regulation of muscle tissue development	4/245	0	86	11	64
	GO:0098			181/232	0.012670	0.074797	0.058417
BP	656	anion transmembrane transport	6/245	10	92	73	55
	GO:0015			86/2321	0.013102		0.060290
BP	908	fatty acid transport	4/245	0	54	0.077196	62
	GO:0016			133/232	0.013576	0.079031	0.061724
BP	999	antibiotic metabolic process	5/245	10	75	95	51
	GO:0046			133/232	0.013576	0.079031	0.061724
BP	928	regulation of neurotransmitter secretion	5/245	10	75	95	51
	GO:0006			17/2321	0.013595	0.079031	0.061724
BP	677	glycosylceramide metabolic process	2/245	0	78	95	51
	GO:0010			17/2321	0.013595	0.079031	0.061724
BP	888	negative regulation of lipid storage	2/245	0	78	95	51
	GO:0015			17/2321	0.013595	0.079031	0.061724
BP	701	bicarbonate transport	2/245	0	78	95	51
	GO:0040			17/2321	0.013595	0.079031	0.061724
BP	015	negative regulation of multicellular organism growth	2/245	0	78	95	51
	GO:1902	negative regulation of oxidative stress-induced intrinsic apoptotic signaling		17/2321	0.013595	0.079031	0.061724
BP	176	pathway	2/245	0	78	95	51
	GO:0009			184/232	0.013647	0.079182	0.061842
BP	746	response to hexose	6/245	10	66	44	04
BP	GO:0034	response to monosaccharide	6/245	185/232	0.013984	0.080983	0.063248

	284			10	62	15	41
	GO:1903			88/2321	0.014156	0.081752	0.063849
BP	035	negative regulation of response to wounding	4/245	0	6	48	26
	GO:2000			48/2321	0.014171	0.081752	0.063849
BP	725	regulation of cardiac muscle cell differentiation	3/245	0	15	48	26
	GO:1903			186/232	0.014327	0.082497	0.064430
BP	305	regulation of regulated secretory pathway	6/245	10	33	28	95
	GO:0071			135/232	0.014401	0.082770	
BP	453	cellular response to oxygen levels	5/245	10	88	06	0.064644
	GO:0009			422/232	0.014645	0.084009	0.065611
BP	896	positive regulation of catabolic process	10/245	10	04	03	64
	GO:0070			89/2321	0.014703	0.084184	0.065748
BP	301	cellular response to hydrogen peroxide	4/245	0	18	01	3
	GO:0017			136/232	0.014826	0.084573	0.066052
BP	158	regulation of calcium ion-dependent exocytosis	5/245	10	8	85	77
	GO:0097			136/232	0.014826	0.084573	0.066052
BP	553	calcium ion transmembrane import into cytosol	5/245	10	8	85	77
	GO:0008			301/232	0.015009	0.084992	0.066379
BP	202	steroid metabolic process	8/245	10	78	53	76
	GO:0001			18/2321	0.015189	0.084992	0.066379
BP	833	inner cell mass cell proliferation	2/245	0	86	53	76
	GO:0010			18/2321	0.015189	0.084992	0.066379
BP	002	cardioblast differentiation	2/245	0	86	53	76
	GO:0034			18/2321	0.015189	0.084992	0.066379
BP	389	lipid droplet organization	2/245	0	86	53	76
BP	GO:0034	response to prostaglandin E	2/245	18/2321	0.015189	0.084992	0.066379

	695			0	86	53	76
	GO:0039			18/2321	0.015189	0.084992	0.066379
BP	529	RIG-I signaling pathway	2/245	0	86	53	76
	GO:0039	regulation of viral-induced cytoplasmic pattern recognition receptor signaling		18/2321	0.015189	0.084992	0.066379
BP	531	pathway	2/245	0	86	53	76
	GO:0045			18/2321	0.015189	0.084992	0.066379
BP	722	positive regulation of gluconeogenesis	2/245	0	86	53	76
	GO:0060			18/2321	0.015189	0.084992	0.066379
BP	947	cardiac vascular smooth muscle cell differentiation	2/245	0	86	53	76
	GO:0086			18/2321	0.015189	0.084992	0.066379
BP	011	membrane repolarization during action potential	2/245	0	86	53	76
	GO:0002			137/232	0.015260	0.084992	0.066379
BP	221	pattern recognition receptor signaling pathway	5/245	10	04	53	76
	GO:0072			90/2321	0.015262	0.084992	0.066379
BP	655	establishment of protein localization to mitochondrion	4/245	0	94	53	76
	GO:1902			90/2321	0.015262	0.084992	0.066379
BP	600	proton transmembrane transport	4/245	0	94	53	76
	GO:0007			138/232	0.015701	0.087117	0.068039
BP	050	cell cycle arrest	5/245	10	67	11	08
	GO:0016			138/232	0.015701	0.087117	0.068039
BP	079	synaptic vesicle exocytosis	5/245	10	67	11	08
	GO:0019			427/232	0.015771	0.087345	0.068217
BP	932	second-messenger-mediated signaling	10/245	10	58	89	75
	GO:0051			50/2321	0.015814	0.087424	0.068279
BP	055	negative regulation of lipid biosynthetic process	3/245	0	5	62	24
BP	GO:0002	innate immune response-activating signal transduction	5/245	139/232	0.016151	0.089127	0.069609

	758			10	75	25	01
	GO:0071			140/232	0.016610	0.091492	0.071455
BP	333	cellular response to glucose stimulus	5/245	10	35	09	96
	GO:0006			51/2321	0.016674	0.091515	0.071474
BP	111	regulation of gluconeogenesis	3/245	0	67	39	16
	GO:2000			51/2321	0.016674	0.091515	0.071474
BP	649	regulation of sodium ion transmembrane transporter activity	3/245	0	67	39	16
	GO:0010			19/2321	0.016860	0.091870	0.071751
BP	766	negative regulation of sodium ion transport	2/245	0	02	5	51
	GO:0010	regulation of release of sequestered calcium ion into cytosol by sarcoplasmic		19/2321	0.016860	0.091870	0.071751
BP	880	reticulum	2/245	0	02	5	51
	GO:0021			19/2321	0.016860	0.091870	0.071751
BP	692	cerebellar Purkinje cell layer morphogenesis	2/245	0	02	5	51
	GO:0034			19/2321	0.016860	0.091870	0.071751
BP	063	stress granule assembly	2/245	0	02	5	51
	GO:0036			93/2321	0.017022	0.092588	0.072312
BP	473	cell death in response to oxidative stress	4/245	0	17	47	24
	GO:0000			194/232	0.017282	0.093834	0.073285
BP	209	protein polyubiquitination	6/245	10	11	78	62
	GO:0031			371/232	0.017512	0.094006	0.073419
BP	667	response to nutrient levels	9/245	10	24	26	55
	GO:0001			52/2321	0.017560	0.094006	0.073419
BP	895	retina homeostasis	3/245	0	59	26	55
	GO:0021			52/2321	0.017560	0.094006	0.073419
BP	587	cerebellum morphogenesis	3/245	0	59	26	55
BP	GO:0046	pigment biosynthetic process	3/245	52/2321	0.017560	0.094006	0.073419

	148			0	59	26	55
	GO:0061			52/2321	0.017560	0.094006	0.073419
BP	614	pri-miRNA transcription by RNA polymerase II	3/245	0	59	26	55
	GO:1900			52/2321	0.017560	0.094006	0.073419
BP	271	regulation of long-term synaptic potentiation	3/245	0	59	26	55
	GO:1901			52/2321	0.017560	0.094006	0.073419
BP	570	fatty acid derivative biosynthetic process	3/245	0	59	26	55
	GO:1903			52/2321	0.017560	0.094006	0.073419
BP	202	negative regulation of oxidative stress-induced cell death	3/245	0	59	26	55
	GO:1902			94/2321	0.017635	0.094241	0.073603
BP	882	regulation of response to oxidative stress	4/245	0	54	82	53
	GO:0019			143/232	0.018037	0.096222	0.075150
BP	233	sensory perception of pain	5/245	10	84	89	75
	GO:0070			95/2321	0.018262	0.097251	0.075953
BP	585	protein localization to mitochondrion	4/245	0	51	06	76
	GO:0072			374/232	0.018346	0.097529	0.076171
BP	594	establishment of protein localization to organelle	9/245	10	83	57	28
	GO:1900			53/2321	0.018472	0.097872	0.076438
BP	408	negative regulation of cellular response to oxidative stress	3/245	0	32	34	98
	GO:0032			438/232	0.018475	0.097872	0.076438
BP	386	regulation of intracellular transport	10/245	10	57	34	98
	GO:0098			144/232	0.018531	0.097996	0.076535
BP	693	regulation of synaptic vesicle cycle	5/245	10	11	11	65
	GO:0009			198/232	0.018906	0.099805	0.077949
BP	743	response to carbohydrate	6/245	10	12	96	16
CC	GO:0005	mitochondrial matrix	27/248	264/234	6.8557E-	2.4447E-	2.0762E-

	759			36	19	16	16
	GO:0019			475/234	1.2766E-	2.4447E-	2.0762E-
CC	866	organelle inner membrane	34/248	36	18	16	16
	GO:0005			433/234	5.6109E-	7.1632E-	6.0834E-
CC	743	mitochondrial inner membrane	32/248	36	18	16	16
	GO:0042			163/234	2.2826E-	2.1856E-	1.8562E-
CC	383	sarcolemma	15/248	36	10	08	08
	GO:0030			70/2343	3.3393E-	2.5579E-	2.1723E-
CC	315	T-tubule	10/248	6	09	07	07
	GO:0009			47/2343	9.8691E-	0.000539	0.000458
CC	295	nucleoid	6/248	6	06	98	58
	GO:0042			47/2343	9.8691E-	0.000539	0.000458
CC	645	mitochondrial nucleoid	6/248	6	06	98	58
	GO:0016			75/2343	1.4689E-	0.000703	0.000597
CC	529	sarcoplasmic reticulum	7/248	6	05	23	22
	GO:0005			79/2343	2.0696E-	0.000880	0.000747
CC	811	lipid droplet	7/248	6	05	75	97
	GO:0016			84/2343	3.0921E-	0.001184	0.001005
CC	528	sarcoplasm	7/248	6	05	28	75
	GO:0098			263/234	0.000121	0.004230	0.003592
CC	798	mitochondrial protein complex	11/248	36	5	38	66
	GO:1902			308/234	0.000470	0.014862	0.012622
CC	495	transmembrane transporter complex	11/248	36	62	86	3
	GO:0014			62/2343	0.000504	0.014862	0.012622
CC	704	intercalated disc	5/248	6	48	86	3
CC	GO:1990	transporter complex	11/248	315/234	0.000567	0.015520	0.013180

	351			36	32	13	48
	GO:0019			185/234	0.000822	0.017931	0.015228
CC	867	outer membrane	8/248	36	36	16	05
	GO:0031			185/234	0.000822	0.017931	0.015228
CC	968	organelle outer membrane	8/248	36	36	16	05
	GO:0005			143/234	0.000842	0.017931	0.015228
CC	777	peroxisome	7/248	36	72	16	05
	GO:0042			143/234	0.000842	0.017931	0.015228
CC	579	microbody	7/248	36	72	16	05
	GO:0031			249/234	0.001402	0.026940	0.022879
CC	300	intrinsic component of organelle membrane	9/248	36	48	61	34
	GO:0005			407/234	0.001406	0.026940	0.022879
CC	635	nuclear envelope	12/248	36	82	61	34
	GO:0044			83/2343	0.001891	0.033475	0.028429
CC	291	cell-cell contact zone	5/248	6	67	9	45
	GO:0044			123/234	0.002004	0.033475	0.028429
CC	853	plasma membrane raft	6/248	36	37	9	45
	GO:0033			24/2343	0.002010	0.033475	0.028429
CC	017	sarcoplasmic reticulum membrane	3/248	6	3	9	45
	GO:0005			168/234	0.002132	0.034035	0.028904
CC	741	mitochondrial outer membrane	7/248	36	75	2	43
	GO:0034			219/234	0.002394	0.036680	0.031151
CC	703	cation channel complex	8/248	36	32	94	33
	GO:0031			222/234	0.002603	0.038351	0.032569
CC	301	integral component of organelle membrane	8/248	36	48	22	82
CC	GO:0005	caveola	5/248	94/2343	0.003256	0.046196	0.039232

	901			6	67	44	38
	GO:0034			293/234	0.004167	0.057001	0.048408
CC	702	ion channel complex	9/248	36	24	94	95
	GO:0045			364/234	0.005635	0.068160	0.057885
CC	121	membrane raft	10/248	36	74	54	41
	GO:0098			365/234	0.005742	0.068160	0.057885
CC	857	membrane microdomain	10/248	36	18	54	41
	GO:0090			11/2343	0.005759	0.068160	0.057885
CC	533	cation-transporting ATPase complex	2/248	6	79	54	41
	GO:1990			11/2343	0.005759	0.068160	0.057885
CC	454	L-type voltage-gated calcium channel complex	2/248	6	79	54	41
	GO:1990			108/234	0.005872	0.068160	0.057885
CC	204	oxidoreductase complex	5/248	36	84	54	41
	GO:0045			370/234	0.006298	0.070697	0.060040
CC	211	postsynaptic membrane	10/248	36	15	61	02
	GO:0005			36/2343	0.006460	0.070697	0.060040
CC	790	smooth endoplasmic reticulum	3/248	6	62	61	02
	GO:0032			71/2343	0.006832	0.071049	0.060338
CC	592	integral component of mitochondrial membrane	4/248	6	45	04	47
	GO:0030			12/2343	0.006863	0.071049	0.060338
CC	061	mitochondrial crista	2/248	6	75	04	47
	GO:0098			377/234	0.007145	0.072023	0.061166
CC	589	membrane region	10/248	36	94	59	11
	GO:0098			73/2343	0.007529	0.073944	0.062797
CC	573	intrinsic component of mitochondrial membrane	4/248	6	64	94	82
CC	GO:0043	myelin sheath	7/248	213/234	0.007758	0.074284	0.063086

	209			36	19	7	36
	GO:0098			13/2343	0.008055	0.075249	0.063905
CC	533	ATPase dependent transmembrane transport complex	2/248	6	42	37	61
	GO:0044			216/234	0.008345	0.076052	0.064587
CC	455	mitochondrial membrane part	7/248	36	24	66	8
	GO:0012			332/234	0.009168	0.076052	0.064587
CC	506	vesicle membrane	9/248	36	51	66	8
	GO:0042			14/2343	0.009332	0.076052	0.064587
CC	581	specific granule	2/248	6	83	66	8
	GO:0045			14/2343	0.009332	0.076052	0.064587
CC	239	tricarboxylic acid cycle enzyme complex	2/248	6	83	66	8
	GO:0071			14/2343	0.009332	0.076052	0.064587
CC	006	U2-type catalytic step 1 spliceosome	2/248	6	83	66	8
	GO:0071			14/2343	0.009332	0.076052	0.064587
CC	012	catalytic step 1 spliceosome	2/248	6	83	66	8
	GO:0071			84/2343	0.012202	0.096192	0.081691
CC	013	catalytic step 2 spliceosome	4/248	6	99	49	58
	GO:0031			233/234	0.012306	0.096192	0.081691
CC	965	nuclear membrane	7/248	36	61	49	58
	GO:0050			288/227	1.7754E-	1.0031E-	8.3165E-
MF	662	coenzyme binding	24/251	10	14	11	12
	GO:0003			10/2271	3.4751E-	9.7603E-	8.0919E-
MF	995	acyl-CoA dehydrogenase activity	6/251	0	10	08	08
	GO:0016			56/2271	5.1825E-	9.7603E-	8.0919E-
MF	627	oxidoreductase activity, acting on the CH-CH group of donors	10/251	0	10	08	08
MF	GO:0016	oxidoreductase activity, acting on the aldehyde or oxo group of donors	8/251	58/2271	2.3695E-	3.0928E-	2.5641E-

	903			0	07	05	05
	GO:0050			82/2271	3.0958E-	3.0928E-	2.5641E-
MF	660	flavin adenine dinucleotide binding	9/251	0	07	05	05
	GO:0000			26/2271	3.2844E-	3.0928E-	2.5641E-
MF	062	fatty-acyl-CoA binding	6/251	0	07	05	05
	GO:0016			146/227	7.1095E-	5.7384E-	4.7575E-
MF	874	ligase activity	11/251	10	07	05	05
	GO:0016	oxidoreductase activity, acting on the aldehyde or oxo group of donors, NAD or		48/2271	9.2713E-	6.5479E-	5.4286E-
MF	620	NADP as acceptor	7/251	0	07	05	05
	GO:0016			267/227	1.7983E-	0.000109	9.085E-0
MF	746	transferase activity, transferring acyl groups	14/251	10	06	58	5
	GO:1901			35/2271	2.1304E-	0.000109	9.085E-0
MF	567	fatty acid derivative binding	6/251	0	06	58	5
	GO:0016			233/227	2.1334E-	0.000109	9.085E-0
MF	747	transferase activity, transferring acyl groups other than amino-acyl groups	13/251	10	06	58	5
	GO:0008			43/2271	7.4314E-	0.000349	0.000290
MF	374	O-acyltransferase activity	6/251	0	06	9	09
	GO:0030			51/2271	2.039E-0	0.000886	0.000734
MF	170	pyridoxal phosphate binding	6/251	0	5	19	71
	GO:0070			52/2271	2.2838E-	0.000921	0.000764
MF	279	vitamin B6 binding	6/251	0	05	69	14
	GO:0052			156/227	6.2026E-	0.002225	0.001844
MF	689	carboxylic ester hydrolase activity	9/251	10	05	26	88
	GO:0022			414/227	6.3016E-	0.002225	0.001844
MF	838	substrate-specific channel activity	15/251	10	05	26	88
MF	GO:0033	amide binding	14/251	371/227	7.1947E-	0.002360	0.001956

	218			10	05	43	95
	GO:0016			21/2271		0.002360	0.001956
MF	408	C-acyltransferase activity	4/251	0	7.52E-05	43	95
	GO:0008			22/2271	9.1115E-	0.002709	0.002246
MF	483	transaminase activity	4/251	0	05	48	33
	GO:0015			438/227	0.000117	0.003168	0.002626
MF	267	channel activity	15/251	10	76	28	71
	GO:0022			438/227	0.000117	0.003168	0.002626
MF	803	passive transmembrane transporter activity	15/251	10	76	28	71
	GO:0016			24/2271	0.000130	0.003340	0.002769
MF	769	transferase activity, transferring nitrogenous groups	4/251	0	08	63	59
	GO:0005			402/227	0.000166	0.004097	0.003397
MF	216	ion channel activity	14/251	10	82	91	43
	GO:0016			27/2271	0.000209	0.004927	0.004085
MF	645	oxidoreductase activity, acting on the CH-NH group of donors	4/251	0	32	65	33
	GO:0005			192/227	0.000298	0.006482	0.005374
MF	244	voltage-gated ion channel activity	9/251	10	31	53	43
	GO:0022			192/227	0.000298	0.006482	0.005374
MF	832	voltage-gated channel activity	9/251	10	31	53	43
	GO:0022			331/227	0.000338	0.007082	0.005871
MF	836	gated channel activity	12/251	10	46	56	89
	GO:0044			13/2271	0.000351	0.007093	0.005880
MF	548	S100 protein binding	3/251	0	52	14	66
	GO:1901			250/227	0.000492	0.009596	0.007955
MF	681	sulfur compound binding	10/251	10	54	03	72
MF	GO:0016	acylglycerol O-acyltransferase activity	3/251	15/2271	0.000550	0.010361	0.008590

	411			0	16	33	2
	GO:0051			62/2271	0.000613	0.011130	0.009228
MF	287	NAD binding	5/251	0	74	92	24
	GO:0022			355/227	0.000630	0.011130	0.009228
MF	804	active transmembrane transporter activity	12/251	10	42	92	24
	GO:0019			132/227	0.000676	0.011583	0.009603
MF	842	vitamin binding	7/251	10	54	11	14
	GO:0051			97/2271	0.000733	0.012026	0.009971
MF	082	unfolded protein binding	6/251	0	24	89	06
	GO:0005			312/227	0.000748	0.012026	0.009971
MF	261	cation channel activity	11/251	10	22	89	06
	GO:0004			17/2271	0.000808	0.012026	0.009971
MF	029	aldehyde dehydrogenase (NAD) activity	3/251	0	89	89	06
	GO:0004			17/2271	0.000808	0.012026	0.009971
MF	724	magnesium-dependent protein serine/threonine phosphatase activity	3/251	0	89	89	06
	GO:0034			17/2271	0.000808	0.012026	0.009971
MF	185	apolipoprotein binding	3/251	0	89	89	06
	GO:0022			319/227	0.000896	0.012987	0.010767
MF	839	ion gated channel activity	11/251	10	5	78	7
	GO:0046			426/227	0.000975	0.013777	0.011422
MF	873	metal ion transmembrane transporter activity	13/251	10	42	76	64
	GO:0022			143/227	0.001083	0.014928	0.012376
MF	843	voltage-gated cation channel activity	7/251	10	3	35	55
	GO:0097			71/2271	0.001139	0.015328	0.012707
MF	110	scaffold protein binding	5/251	0	43	08	96
MF	GO:0008	protein C-terminus binding	9/251	234/227	0.001229	0.016158	0.013396

	022			10	74	18	16
	GO:0015			76/2271	0.001546	0.019855	0.016461
MF	297	antiporter activity	5/251	0	29	74	68
	GO:0016			46/2271	0.001651	0.020735	0.017190
MF	790	thiolester hydrolase activity	4/251	0	48	22	82
	GO:0034			22/2271	0.001758	0.021295	0.017655
MF	237	protein kinase A regulatory subunit binding	3/251	0	69	12	01
	GO:0016			115/227	0.001771	0.021295	0.017655
MF	407	acetyltransferase activity	6/251	10	45	12	01
	GO:0015			157/227	0.001854	0.021827	0.018096
MF	079	potassium ion transmembrane transporter activity	7/251	10	36	34	26
	GO:0005			121/227	0.002290	0.026379	0.021870
MF	267	potassium channel activity	6/251	10	99	93	65
	GO:0015			360/227	0.002334	0.026379	0.021870
MF	077	monovalent inorganic cation transmembrane transporter activity	11/251	10	51	93	65
	GO:0005			85/2271	0.002533	0.027737	0.022996
MF	249	voltage-gated potassium channel activity	5/251	0	97	63	27
	GO:0047			25/2271	0.002563	0.027737	0.022996
MF	617	acyl-CoA hydrolase activity	3/251	0	27	63	27
	GO:0051			52/2271	0.002601	0.027737	0.022996
MF	018	protein kinase A binding	4/251	0	94	63	27
	GO:0016			26/2271	0.002874	0.030072	0.024931
MF	878	acid-thiol ligase activity	3/251	0	15	1	69
	GO:0015			27/2271	0.003207	0.032358	0.026827
MF	301	anion:anion antiporter activity	3/251	0	25	88	58
MF	GO:0016	CoA hydrolase activity	3/251	27/2271	0.003207	0.032358	0.026827

	289			0	25	88	58
	GO:0060			225/227	0.003673	0.036409	0.030185
MF	090	molecular adaptor activity	8/251	10	15	26	6
	GO:0042			282/227	0.004303	0.041923	0.034756
MF	623	ATPase activity, coupled	9/251	10	61	13	95
	GO:0005			148/227	0.006145	0.055316	0.045860
MF	342	organic acid transmembrane transporter activity	6/251	10	6	46	87
	GO:0046			148/227	0.006145	0.055316	0.045860
MF	943	carboxylic acid transmembrane transporter activity	6/251	10	6	46	87
	GO:0005			34/2271	0.006199	0.055316	0.045860
MF	310	dicarboxylic acid transmembrane transporter activity	3/251	0	45	46	87
	GO:0003			11/2271	0.006265	0.055316	0.045860
MF	841	1-acylglycerol-3-phosphate O-acyltransferase activity	2/251	0	94	46	87
	GO:0015			11/2271	0.006265	0.055316	0.045860
MF	174	basic amino acid transmembrane transporter activity	2/251	0	94	46	87
	GO:0102			11/2271	0.006265	0.055316	0.045860
MF	991	myristoyl-CoA hydrolase activity	2/251	0	94	46	87
	GO:0005			108/227	0.007022	0.061044	0.050610
MF	496	steroid binding	5/251	10	87	92	13
	GO:0042			12/2271	0.007464	0.063901	0.052978
MF	171	lysophosphatidic acid acyltransferase activity	2/251	0	6	48	41
	GO:0008			310/227	0.007844	0.065281	0.054122
MF	509	anion transmembrane transporter activity	9/251	10	94	39	44
	GO:0016			37/2271	0.007856	0.065281	0.054122
MF	877	ligase activity, forming carbon-sulfur bonds	3/251	0	88	39	44
MF	GO:0042	ATPase activity, coupled to transmembrane movement of substances	5/251	113/227	0.008461	0.068834	0.057068

	626			10	44	68	34
	GO:0002			13/2271	0.008757	0.068834	0.057068
MF	161	aminoacyl-tRNA editing activity	2/251	0	89	68	34
	GO:0015			13/2271	0.008757	0.068834	0.057068
MF	556	C4-dicarboxylate transmembrane transporter activity	2/251	0	89	68	34
	GO:0043			114/227	0.008771	0.068834	0.057068
MF	492	ATPase activity, coupled to movement of substances	5/251	10	85	68	34
	GO:0030			116/227	0.009416	0.072878	0.060420
MF	165	PDZ domain binding	5/251	10	13	27	74
	GO:0016			265/227	0.009551	0.072930	0.060463
MF	791	phosphatase activity	8/251	10	9	07	68
	GO:0071			14/2271	0.010143	0.075641	0.062711
MF	617	lysophospholipid acyltransferase activity	2/251	0	58	43	58
	GO:0004			41/2271	0.010436	0.075641	0.062711
MF	812	aminoacyl-tRNA ligase activity	3/251	0	86	43	58
	GO:0016			41/2271	0.010436	0.075641	0.062711
MF	875	ligase activity, forming carbon-oxygen bonds	3/251	0	86	43	58
	GO:0015			119/227	0.010442	0.075641	0.062711
MF	405	P-P-bond-hydrolysis-driven transmembrane transporter activity	5/251	10	53	43	58
	GO:0015			121/227	0.011167	0.079871	0.066218
MF	399	primary active transmembrane transporter activity	5/251	10	83	22	34
	GO:0016			122/227	0.011543	0.081522	0.067587
MF	298	lipase activity	5/251	10	06	83	63
	GO:0004			171/227	0.012047	0.084031	0.069667
MF	721	phosphoprotein phosphatase activity	6/251	10	03	73	67
MF	GO:0016	palmitoyl-CoA hydrolase activity	2/251	16/2271	0.013183	0.089742	0.074402

	290			0	47	89	58
MF	GO:0070006	metalloaminopeptidase activity	2/251	16/22710	0.01318347	0.08974289	0.07440258
MF	GO:0016879	ligase activity, forming carbon-nitrogen bonds	3/251	46/22710	0.01427609	0.0960237	0.07960977
MF	GO:0016646	oxidoreductase activity, acting on the CH-NH group of donors, NAD or NADP as acceptor	2/251	17/22710	0.01483341	0.09812017	0.08134788
MF	GO:0015459	potassium channel regulator activity	3/251	47/22710	0.01512744	0.09812017	0.08134788
MF	GO:0015238	drug transmembrane transporter activity	4/251	86/22710	0.01526616	0.09812017	0.08134788
MF	GO:0015291	secondary active transmembrane transporter activity	7/251	233/22710	0.01528243	0.09812017	0.08134788
KEGG	mmu00071	Fatty acid degradation	12/151	51/8910	3.2525E-11	7.3181E-09	6.2311E-09
KEGG	mmu00280	Valine, leucine and isoleucine degradation	12/151	57/8910	1.3281E-10	1.4941E-08	1.2721E-08
KEGG	mmu00640	Propanoate metabolism	9/151	34/8910	3.3185E-09	2.4888E-07	2.1192E-07
KEGG	mmu01200	Carbon metabolism	13/151	124/8910	1.5429E-07	8.679E-06	7.3898E-06
KEGG	mmu01212	Fatty acid metabolism	9/151	62/8910	8.5682E-07	3.8557E-05	3.283E-05
KEGG	mmu00620	Pyruvate metabolism	7/151	39/8910	3.4099E-06	0.00012787	0.00010888
KEGG	mmu0493	Insulin resistance	10/151	110/891	1.5958E-	0.000512	0.000436

	1			0	05	95	76
KEGG	mmu00330	Arginine and proline metabolism	7/151	54/8910	3.1743E-05	0.00089277	0.00076016
KEGG	mmu04975	Fat digestion and absorption	6/151	43/8910	7.793E-05	0.00194824	0.00165885
KEGG	mmu03320	PPAR signaling pathway	8/151	89/8910	0.00012509	0.00281447	0.00239641
KEGG	mmu00410	beta-Alanine metabolism	5/151	31/8910	0.00015575	0.00318575	0.00271254
KEGG	mmu05414	Dilated cardiomyopathy	8/151	94/8910	0.00018359	0.00344228	0.00293097
KEGG	mmu01210	2-Oxocarboxylic acid metabolism	4/151	20/8910	0.00031085	0.00500517	0.00426171
KEGG	mmu05412	Arrhythmogenic right ventricular cardiomyopathy	7/151	77/8910	0.00031143	0.00500517	0.00426171
KEGG	mmu00250	Alanine, aspartate and glutamate metabolism	5/151	39/8910	0.00047326	0.00709883	0.00604438
KEGG	mmu00561	Glycerolipid metabolism	6/151	62/8910	0.00060326	0.00848334	0.00722324
KEGG	mmu05410	Hypertrophic cardiomyopathy	7/151	91/8910	0.00086064	0.01139078	0.00969882
KEGG	mmu00010	Glycolysis / Gluconeogenesis	6/151	67/8910	0.00091369	0.01142107	0.00972461
KEGG	mmu00062	Fatty acid elongation	4/151	29/8910	0.00135361	0.01602954	0.01364855
KEGG	mmu0038	Tryptophan metabolism	5/151	52/8910	0.001790	0.020144	0.017152

	0				63	56	33
KEGG	mmu04922	Glucagon signaling pathway	7/151	104/8910	0.00188182	0.02016236	0.01716748
KEGG	mmu04146	Peroxisome	6/151	86/8910	0.00330995	0.03385172	0.02882345
KEGG	mmu04260	Cardiac muscle contraction	6/151	87/8910	0.00350644	0.03430211	0.02920695
KEGG	mmu04261	Adrenergic signaling in cardiomyocytes	8/151	152/8910	0.00424239	0.03809484	0.03243631
KEGG	mmu00220	Arginine biosynthesis	3/151	20/8910	0.00440207	0.03809484	0.03243631
KEGG	mmu00770	Pantothenate and CoA biosynthesis	3/151	20/8910	0.00440207	0.03809484	0.03243631
KEGG	mmu04152	AMPK signaling pathway	7/151	126/8910	0.00549726	0.04581047	0.03900588
KEGG	mmu04910	Insulin signaling pathway	7/151	139/8910	0.00926733	0.07233207	0.06158801
KEGG	mmu00340	Histidine metabolism	3/151	26/8910	0.0093228	0.07233207	0.06158801
KEGG	mmu01230	Biosynthesis of amino acids	5/151	79/8910	0.01079229	0.08094214	0.06891916
KEGG	mmu00650	Butanoate metabolism	3/151	28/8910	0.01146109	0.08318534	0.07082916

Table S7. GO analysis of DEGs in VO and TAC groups with no interaction.

ID	Description	GeneR	BgRatio	pvalue	p.adjust	qvalue	Count

		atio					
GO:004		87/171	395/23	1.0512E	6.1411E	4.1948E	
2692	muscle cell differentiation	0	210	-20	-17	-17	87
GO:005		75/171	316/23	5.2563E	1.5354E	1.0488E	
1146	striated muscle cell differentiation	0	210	-20	-16	-16	75
GO:000		91/171	459/23	2.1786E	4.2426E	2.898E-	
7005	mitochondrion organization	0	210	-18	-15	15	91
GO:000		77/171	379/23	2.2827E	3.3339E	2.2773E	
6091	generation of precursor metabolites and energy	0	210	-16	-13	-13	77
GO:001		60/171	254/23	3.6082E	4.2158E	2.8797E	
5980	energy derivation by oxidation of organic compounds	0	210	-16	-13	-13	60
GO:003		76/171	393/23	5.8637E	5.7093E	3.8999E	
2970	regulation of actin filament-based process	0	210	-15	-12	-12	76
GO:000		79/171	447/23	2.6493E	2.211E-	1.5103E	
6163	purine nucleotide metabolic process	0	210	-13	10	-10	79
GO:000		77/171	433/23	3.8391E	2.5432E	1.7372E	
9259	ribonucleotide metabolic process	0	210	-13	-10	-10	77
GO:000		73/171	400/23	4.2174E	2.5432E	1.7372E	
7015	actin filament organization	0	210	-13	-10	-10	73
GO:004		42/171	163/23	4.3532E	2.5432E	1.7372E	
5333	cellular respiration	0	210	-13	-10	-10	42
GO:000		60/171	298/23	7.0948E	3.768E-	2.5738E	
9141	nucleoside triphosphate metabolic process	0	210	-13	10	-10	60
GO:000		75/171	423/23	9.0621E	4.4118E	3.0136E	
9150	purine ribonucleotide metabolic process	0	210	-13	-10	-10	75
GO:001	ribose phosphate metabolic process	78/171	450/23	1.0553E	4.7422E	3.2393E	78

9693		0	210	-12	-10	-10	
GO:003		65/171	344/23	1.6348E	6.8217E	4.6597E	
2956	regulation of actin cytoskeleton organization	0	210	-12	-10	-10	65
GO:006		80/171	472/23	1.7934E	6.8709E	4.6934E	
0537	muscle tissue development	0	210	-12	-10	-10	80
GO:007		82/171	490/23	1.8818E	6.8709E	4.6934E	
2521	purine-containing compound metabolic process	0	210	-12	-10	-10	82
GO:005		47/171	207/23	2.624E-	9.0173E	6.1595E	
5001	muscle cell development	0	210	12	-10	-10	47
GO:005		44/171	193/23	1.1237E	3.6472E	2.4913E	
5002	striated muscle cell development	0	210	-11	-09	-09	44
GO:001		75/171	449/23	1.8346E	5.641E-	3.8532E	
4706	striated muscle tissue development	0	210	-11	09	-09	75
GO:005		39/171	161/23	2.3243E	6.7856E	4.6351E	
1147	regulation of muscle cell differentiation	0	210	-11	-09	-09	39
GO:190		63/171	348/23	2.4392E	6.7856E	4.6351E	
2903	regulation of supramolecular fiber organization	0	210	-11	-09	-09	63
GO:011		51/171	255/23	4.8199E	1.2799E	8.7427E	
0053	regulation of actin filament organization	0	210	-11	-08	-09	51
GO:000		54/171	281/23	6.4317E	1.6336E	1.1159E	
9144	purine nucleoside triphosphate metabolic process	0	210	-11	-08	-08	54
GO:000		52/171	272/23	1.761E-	4.2865E	2.928E-	
9205	purine ribonucleoside triphosphate metabolic process	0	210	10	-08	08	52
GO:002		26/171	85/232	2.0975E	4.9014E	3.348E-	
2900	electron transport chain	0	10	-10	-08	08	26
GO:190	response to peptide	65/171	384/23	2.3048E	4.9309E	3.3682E	65

1652		0	210	-10	-08	-08	
GO:005		52/171	274/23	2.3206E	4.9309E	3.3682E	
1258	protein polymerization	0	210	-10	-08	-08	52
GO:004		58/171	324/23	2.3633E	4.9309E	3.3682E	
3434	response to peptide hormone	0	210	-10	-08	-08	58
GO:005		49/171	251/23	2.7739E	5.588E-	3.817E-	
1188	cofactor biosynthetic process	0	210	-10	08	08	49
GO:000		52/171	276/23	3.0478E	5.935E-	4.0541E	
9199	ribonucleoside triphosphate metabolic process	0	210	-10	08	-08	52
GO:200		52/171	281/23	5.9377E	1.119E-	7.6433E	
0146	negative regulation of cell motility	0	210	-10	07	-08	52
GO:005		31/171	123/23	8.9227E	1.629E-	1.1127E	
1153	regulation of striated muscle cell differentiation	0	210	-10	07	-07	31
GO:000		52/171	285/23	9.9775E	1.7663E	1.2065E	
9123	nucleoside monophosphate metabolic process	0	210	-10	-07	-07	52
GO:000		64/171	392/23	1.4347E	2.4652E	1.6839E	
3012	muscle system process	0	210	-09	-07	-07	64
GO:000		60/171	357/23	1.4998E	2.495E-	1.7042E	
7178	transmembrane receptor protein serine/threonine kinase signaling pathway	0	210	-09	07	-07	60
GO:004		47/171	247/23	1.5375E	2.495E-	1.7042E	
6034	ATP metabolic process	0	210	-09	07	-07	47
GO:005		55/171	316/23	1.9798E	3.126E-	2.1353E	
1271	negative regulation of cellular component movement	0	210	-09	07	-07	55
GO:002		24/171	81/232	2.1216E	3.2617E	2.228E-	
2904	respiratory electron transport chain	0	10	-09	-07	07	24
GO:005	positive regulation of cytoskeleton organization	43/171	218/23	2.4309E	3.6414E	2.4873E	43

1495		0	210	-09	-07	-07	
GO:000		50/171	276/23	2.6755E	3.9076E	2.6692E	
9161	ribonucleoside monophosphate metabolic process	0	210	-09	-07	-07	50
GO:001		29/171	116/23	3.7776E	5.3826E	3.6767E	
0927	cellular component assembly involved in morphogenesis	0	210	-09	-07	-07	29
GO:005		55/171	322/23	3.9716E	5.5244E	3.7736E	
1235	maintenance of location	0	210	-09	-07	-07	55
GO:000		56/171	331/23	4.1024E	5.5735E	3.8071E	
6732	coenzyme metabolic process	0	210	-09	-07	-07	56
GO:004		56/171	332/23	4.5878E	6.0487E	4.1317E	
0013	negative regulation of locomotion	0	210	-09	-07	-07	56
GO:000		49/171	272/23	4.6592E	6.0487E	4.1317E	
9167	purine ribonucleoside monophosphate metabolic process	0	210	-09	-07	-07	49
GO:000		49/171	273/23	5.2796E	6.7051E	4.5801E	
9126	purine nucleoside monophosphate metabolic process	0	210	-09	-07	-07	49
GO:003		48/171	268/23	8.0981E	1.0066E	6.8757E	
0336	negative regulation of cell migration	0	210	-09	-06	-07	48
GO:007		45/171	244/23	9.3343E	1.1361E	7.7602E	
1375	cellular response to peptide hormone stimulus	0	210	-09	-06	-07	45
GO:190		50/171	287/23	1.0279E	1.2091E	8.2592E	
1653	cellular response to peptide	0	210	-08	-06	-07	50
GO:003		40/171	204/23	1.0348E	1.2091E	8.2592E	
1032	actomyosin structure organization	0	210	-08	-06	-07	40
GO:005		24/171	88/232	1.3107E	1.5014E	1.0255E	
1149	positive regulation of muscle cell differentiation	0	10	-08	-06	-06	24
GO:190	nucleoside phosphate biosynthetic process	50/171	291/23	1.6399E	1.8423E	1.2585E	50

1293		0	210	-08	-06	-06	
GO:000		49/171	285/23	2.2276E	2.4428E	1.6686E	
9165	nucleotide biosynthetic process	0	210	-08	-06	-06	49
GO:007		59/171	374/23	2.258E-	2.4428E	1.6686E	
2594	establishment of protein localization to organelle	0	210	08	-06	-06	59
GO:190		37/171	186/23	2.4531E	2.6056E	1.7798E	
5475	regulation of protein localization to membrane	0	210	-08	-06	-06	37
GO:000		59/171	376/23	2.7471E	2.8658E	1.9575E	
6631	fatty acid metabolic process	0	210	-08	-06	-06	59
GO:190		50/171	297/23	3.2354E	3.2798E	2.2404E	
3829	positive regulation of cellular protein localization	0	210	-08	-06	-06	50
GO:000		64/171	424/23	3.2562E	3.2798E	2.2404E	
7517	muscle organ development	0	210	-08	-06	-06	64
GO:000		47/171	272/23	3.6589E	3.6229E	2.4747E	
6790	sulfur compound metabolic process	0	210	-08	-06	-06	47
GO:004		19/171	61/232	4.0419E	3.9354E	2.6882E	
2773	ATP synthesis coupled electron transport	0	10	-08	-06	-06	19
GO:004		15/171	39/232	4.4468E	4.2587E	2.909E-	
3001	Golgi to plasma membrane protein transport	0	10	-08	-06	06	15
GO:003		40/171	215/23	4.7604E	4.4856E	3.064E-	
2271	regulation of protein polymerization	0	210	-08	-06	06	40
GO:003		42/171	233/23	5.6562E	5.245E-	3.5827E	
4764	positive regulation of transmembrane transport	0	210	-08	06	-06	42
GO:001		63/171	421/23	5.7635E	5.261E-	3.5937E	
0959	regulation of metal ion transport	0	210	-08	06	-06	63
GO:000	actin polymerization or depolymerization	38/171	201/23	6.598E-	5.9301E	4.0507E	38

8154		0	210	08	-06	-06	
GO:200		26/171	109/23	6.7018E	5.9321E	4.0521E	
0379	positive regulation of reactive oxygen species metabolic process	0	210	-08	-06	-06	26
GO:004		22/171	82/232	7.2227E	6.2065E	4.2395E	
8747	muscle fiber development	0	10	-08	-06	-06	22
GO:004		18/171	57/232	7.2243E	6.2065E	4.2395E	
2775	mitochondrial ATP synthesis coupled electron transport	0	10	-08	-06	-06	18
GO:007		47/171	279/23	8.123E-	6.8774E	4.6978E	
2659	protein localization to plasma membrane	0	210	08	-06	-06	47
GO:190		37/171	195/23	8.8724E	7.4047E	5.0579E	
2905	positive regulation of supramolecular fiber organization	0	210	-08	-06	-06	37
GO:004		56/171	362/23	1.0075E	8.2901E	5.6628E	
6394	carboxylic acid biosynthetic process	0	210	-07	-06	-06	56
GO:199		54/171	344/23	1.0401E	8.4308E	5.7589E	
0778	protein localization to cell periphery	0	210	-07	-06	-06	54
GO:000		70/171	495/23	1.0535E	8.4308E	5.7589E	
9611	response to wounding	0	210	-07	-06	-06	70
GO:001		56/171	363/23	1.1076E	8.7441E	5.9729E	
6053	organic acid biosynthetic process	0	210	-07	-06	-06	56
GO:003		40/171	222/23	1.1753E	9.0881E	6.2079E	
0323	respiratory tube development	0	210	-07	-06	-06	40
GO:009		42/171	239/23	1.1823E	9.0881E	6.2079E	
0257	regulation of muscle system process	0	210	-07	-06	-06	42
GO:003		34/171	174/23	1.4026E	1.057E-	7.2203E	
0041	actin filament polymerization	0	210	-07	05	-06	34
GO:001	regulation of lipid metabolic process	53/171	338/23	1.4113E	1.057E-	7.2203E	53

9216		0	210	-07	05	-06	
GO:004		65/171	451/23	1.4821E	1.0868E	7.4239E	
3254	regulation of protein complex assembly	0	210	-07	-05	-06	65
GO:005		30/171	143/23	1.4883E	1.0868E	7.4239E	
5007	cardiac muscle cell differentiation	0	210	-07	-05	-06	30
GO:009	regulation of transmembrane receptor protein serine/threonine kinase signaling	41/171	233/23	1.6039E	1.1568E	7.9019E	
0092	pathway	0	210	-07	-05	-06	41
GO:000		34/171	176/23	1.8668E	1.33E-0	9.085E-	
8064	regulation of actin polymerization or depolymerization	0	210	-07	5	06	34
GO:003		59/171	397/23	1.9192E	1.3429E	9.1727E	
2535	regulation of cellular component size	0	210	-07	-05	-06	59
GO:005		20/171	73/232	1.9308E	1.3429E	9.1727E	
1155	positive regulation of striated muscle cell differentiation	0	10	-07	-05	-06	20
GO:000		34/171	177/23	2.1494E	1.4773E	1.0091E	
6733	oxidoreduction coenzyme metabolic process	0	210	-07	-05	-05	34
GO:003		39/171	219/23	2.3119E	1.5563E	1.0631E	
0324	lung development	0	210	-07	-05	-05	39
GO:003		45/171	271/23	2.3298E	1.5563E	1.0631E	
0198	extracellular matrix organization	0	210	-07	-05	-05	45
GO:000		36/171	194/23	2.3443E	1.5563E	1.0631E	
9108	coenzyme biosynthetic process	0	210	-07	-05	-05	36
GO:003		34/171	179/23	2.838E-	1.8629E	1.2725E	
0832	regulation of actin filament length	0	210	07	-05	-05	34
GO:000		39/171	221/23	2.9514E	1.9158E	1.3086E	
3015	heart process	0	210	-07	-05	-05	39
GO:004	muscle adaptation	26/171	117/23	3.0247E	1.9418E	1.3264E	26

3500		0	210	-07	-05	-05	
GO:014		24/171	103/23	3.3809E	2.1469E	1.4665E	
0053	mitochondrial gene expression	0	210	-07	-05	-05	24
GO:200		18/171	63/232	3.9357E	2.4723E	1.6888E	
1169	regulation of ATP biosynthetic process	0	10	-07	-05	-05	18
GO:003		34/171	182/23	4.2644E	2.627E-	1.7944E	
5051	cardiocyte differentiation	0	210	-07	05	-05	34
GO:004		50/171	322/23	4.2719E	2.627E-	1.7944E	
3270	positive regulation of ion transport	0	210	-07	05	-05	50
GO:004		54/171	360/23	4.6421E	2.8249E	1.9296E	
2060	wound healing	0	210	-07	-05	-05	54
GO:003		39/171	225/23	4.7554E	2.8596E	1.9533E	
2868	response to insulin	0	210	-07	-05	-05	39
GO:004		43/171	260/23	4.7969E	2.8596E	1.9533E	
8738	cardiac muscle tissue development	0	210	-07	-05	-05	43
GO:200		36/171	200/23	5.0784E	2.9967E	2.047E-	
0377	regulation of reactive oxygen species metabolic process	0	210	-07	-05	05	36
GO:004		48/171	307/23	5.8848E	3.4379E	2.3483E	
5765	regulation of angiogenesis	0	210	-07	-05	-05	48
GO:003		56/171	382/23	6.1094E	3.5338E	2.4139E	
4248	regulation of cellular amide metabolic process	0	210	-07	-05	-05	56
GO:003		37/171	211/23	6.9148E	3.922E-	2.679E-	
0595	leukocyte chemotaxis	0	210	-07	05	05	37
GO:006		37/171	211/23	6.9148E	3.922E-	2.679E-	
0047	heart contraction	0	210	-07	05	05	37
GO:000	urogenital system development	52/171	346/23	7.0885E	3.9819E	2.7199E	52

1655		0	210	-07	-05	-05	
GO:004		40/171	238/23	7.7814E	4.3294E	2.9573E	
5444	fat cell differentiation	0	210	-07	-05	-05	40
GO:007		36/171	204/23	8.3181E	4.5844E	3.1315E	
1560	cellular response to transforming growth factor beta stimulus	0	210	-07	-05	-05	36
GO:190		33/171	179/23	8.561E-	4.6741E	3.1928E	
3034	regulation of response to wounding	0	210	07	-05	-05	33
GO:190		63/171	454/23	8.7646E	4.741E-	3.2385E	
4951	positive regulation of establishment of protein localization	0	210	-07	05	-05	63
GO:000		58/171	406/23	9.1684E	4.9139E	3.3566E	
1667	ameboidal-type cell migration	0	210	-07	-05	-05	58
GO:001		56/171	387/23	9.3154E	4.9473E	3.3794E	
0721	negative regulation of cell development	0	210	-07	-05	-05	56
GO:000		40/171	240/23	9.7006E	5.0788E	3.4692E	
6164	purine nucleotide biosynthetic process	0	210	-07	-05	-05	40
GO:006		53/171	359/23	9.7368E	5.0788E	3.4692E	
2012	regulation of small molecule metabolic process	0	210	-07	-05	-05	53
GO:001		26/171	124/23	9.9527E	5.1454E	3.5147E	
4902	myotube differentiation	0	210	-07	-05	-05	26
GO:190		51/171	341/23	1.0475E	5.3682E	3.6669E	
1342	regulation of vasculature development	0	210	-06	-05	-05	51
GO:000		39/171	232/23	1.0579E	5.3741E	3.6709E	
9260	ribonucleotide biosynthetic process	0	210	-06	-05	-05	39
GO:004		49/171	323/23	1.1144E	5.5642E	3.8007E	
2063	gliogenesis	0	210	-06	-05	-05	49
GO:007	ERK1 and ERK2 cascade	49/171	323/23	1.1144E	5.5642E	3.8007E	49

0371		0	210	-06	-05	-05	
GO:006		55/171	380/23	1.153E-	5.7082E	3.8991E	
0562	epithelial tube morphogenesis	0	210	06	-05	-05	55
GO:009		36/171	207/23	1.1912E	5.848E-	3.9946E	
7529	myeloid leukocyte migration	0	210	-06	05	-05	36
GO:007		47/171	306/23	1.2854E	6.2108E	4.2424E	
0372	regulation of ERK1 and ERK2 cascade	0	210	-06	-05	-05	47
GO:001		38/171	225/23	1.2864E	6.2108E	4.2424E	
0632	regulation of epithelial cell migration	0	210	-06	-05	-05	38
GO:004		16/171	55/232	1.3237E	6.3387E	4.3298E	
3470	regulation of carbohydrate catabolic process	0	10	-06	-05	-05	16
GO:007		36/171	208/23	1.34E-0	6.3644E	4.3474E	
1559	response to transforming growth factor beta	0	210	6	-05	-05	36
GO:200		12/171	32/232	1.3831E	6.5161E	4.451E-	
1171	positive regulation of ATP biosynthetic process	0	10	-06	-05	05	12
GO:003		19/171	75/232	1.442E-	6.7394E	4.6035E	
2543	mitochondrial translation	0	10	06	-05	-05	19
GO:006		17/171	62/232	1.5566E	7.2172E	4.9299E	
1951	establishment of protein localization to plasma membrane	0	10	-06	-05	-05	17
GO:004		26/171	127/23	1.6052E	7.3461E	5.0179E	
3467	regulation of generation of precursor metabolites and energy	0	210	-06	-05	-05	26
GO:003		30/171	159/23	1.6095E	7.3461E	5.0179E	
0833	regulation of actin filament polymerization	0	210	-06	-05	-05	30
GO:007		40/171	245/23	1.6594E	7.5148E	5.1332E	
2522	purine-containing compound biosynthetic process	0	210	-06	-05	-05	40
GO:003	cellular response to insulin stimulus	34/171	193/23	1.7291E	7.7654E	5.3044E	34

2869		0	210	-06	-05	-05	
GO:005		46/171	300/23	1.7413E	7.7654E	5.3044E	
1604	protein maturation	0	210	-06	-05	-05	46
GO:001		48/171	319/23	1.8075E	7.986E-	5.455E-	
8108	peptidyl-tyrosine phosphorylation	0	210	-06	05	05	48
GO:000		30/171	160/23	1.8433E	7.986E-	5.455E-	
9142	nucleoside triphosphate biosynthetic process	0	210	-06	05	05	30
GO:001		30/171	160/23	1.8433E	7.986E-	5.455E-	
9362	pyridine nucleotide metabolic process	0	210	-06	05	05	30
GO:007		42/171	264/23	1.8454E	7.986E-	5.455E-	
2330	monocarboxylic acid biosynthetic process	0	210	-06	05	05	42
GO:005		52/171	358/23	2.0103E	8.5867E	5.8654E	
0678	regulation of epithelial cell proliferation	0	210	-06	-05	-05	52
GO:001		22/171	98/232	2.0137E	8.5867E	5.8654E	
9395	fatty acid oxidation	0	10	-06	-05	-05	22
GO:000		33/171	186/23	2.0776E	8.795E-	6.0076E	
6109	regulation of carbohydrate metabolic process	0	210	-06	05	-05	33
GO:005		34/171	195/23	2.1976E	9.2364E	6.3091E	
0731	positive regulation of peptidyl-tyrosine phosphorylation	0	210	-06	-05	-05	34
GO:004		39/171	239/23	2.256E-	9.414E-	6.4305E	
6390	ribose phosphate biosynthetic process	0	210	06	05	-05	39
GO:001		48/171	322/23	2.3628E	9.7899E	6.6872E	
8212	peptidyl-tyrosine modification	0	210	-06	-05	-05	48
GO:000		37/171	223/23	2.7201E	0.00011	7.6442E	
9152	purine ribonucleotide biosynthetic process	0	210	-06	191	-05	37
GO:190	positive regulation of cation transmembrane transport	30/171	163/23	2.7451E	0.00011	7.6605E	30

4064		0	210	-06	215	-05	
GO:005		29/171	155/23	2.8444E	0.00011	7.8824E	
1896	regulation of protein kinase B signaling	0	210	-06	54	-05	29
GO:003		22/171	100/23	2.8777E	0.00011	7.9196E	
4440	lipid oxidation	0	210	-06	594	-05	22
GO:006		40/171	251/23	3.0793E	0.00012	8.4165E	
0541	respiratory system development	0	210	-06	322	-05	40
GO:000		17/171	65/232	3.1803E	0.00012	8.5556E	
6893	Golgi to plasma membrane transport	0	10	-06	525	-05	17
GO:015		17/171	65/232	3.1803E	0.00012	8.5556E	
0076	neuroinflammatory response	0	10	-06	525	-05	17
GO:007		44/171	288/23	3.1945E	0.00012	8.5556E	
2593	reactive oxygen species metabolic process	0	210	-06	525	-05	44
GO:004		61/171	452/23	3.2651E	0.00012	8.6863E	
2326	negative regulation of phosphorylation	0	210	-06	716	-05	61
GO:000		14/171	46/232	3.3396E	0.00012	8.7677E	
6110	regulation of glycolytic process	0	10	-06	836	-05	14
GO:003		14/171	46/232	3.3396E	0.00012	8.7677E	
0811	regulation of nucleotide catabolic process	0	10	-06	836	-05	14
GO:001		54/171	384/23	3.4768E	0.00013	9.0682E	
0256	endomembrane system organization	0	210	-06	276	-05	54
GO:000		30/171	165/23	3.5555E	0.00013	9.1537E	
7179	transforming growth factor beta receptor signaling pathway	0	210	-06	401	-05	30
GO:007		30/171	165/23	3.5555E	0.00013	9.1537E	
2524	pyridine-containing compound metabolic process	0	210	-06	401	-05	30
GO:004	phosphatidylinositol-mediated signaling	29/171	157/23	3.7111E	0.00013	9.4931E	29

8015		0	210	-06	898	-05	
GO:003		58/171	425/23	3.9871E	0.00014	0.00010	
4655	nucleobase-containing compound catabolic process	0	210	-06	742	07	58
GO:005		58/171	425/23	3.9871E	0.00014	0.00010	
0673	epithelial cell proliferation	0	210	-06	742	07	58
GO:003		42/171	272/23	4.0291E	0.00014	0.00010	
4504	protein localization to nucleus	0	210	-06	804	112	42
GO:005		22/171	102/23	4.067E-	0.00014	0.00010	
1897	positive regulation of protein kinase B signaling	0	210	06	843	139	22
GO:005		41/171	263/23		0.00014	0.00010	
0730	regulation of peptidyl-tyrosine phosphorylation	0	210	4.1E-06	843	139	41
GO:000		18/171	73/232	4.1159E	0.00014	0.00010	
9060	aerobic respiration	0	10	-06	843	139	18
GO:004		29/171	158/23	4.2299E	0.00015	0.00010	
6496	nicotinamide nucleotide metabolic process	0	210	-06	16	355	29
GO:004		64/171	486/23	4.3066E	0.00015	0.00010	
8871	multicellular organismal homeostasis	0	210	-06	341	479	64
GO:000		28/171	150/23	4.39E-0	0.00015	0.00010	
8360	regulation of cell shape	0	210	6	543	617	28
GO:190		26/171	134/23	4.5727E	0.00016	0.00010	
4375	regulation of protein localization to cell periphery	0	210	-06	024	946	26
GO:003		40/171	255/23	4.5808E	0.00016	0.00010	
4330	cell junction organization	0	210	-06	024	946	40
GO:190		50/171	349/23	4.6604E	0.00016	0.00011	
4062	regulation of cation transmembrane transport	0	210	-06	206	07	50
GO:000	endothelial cell proliferation	27/171	143/23	5.1724E	0.00017	0.00012	27

1935		0	210	-06	832	181	
GO:000		30/171	168/23	5.1891E	0.00017	0.00012	
9124	nucleoside monophosphate biosynthetic process	0	210	-06	832	181	30
GO:004		26/171	135/23	5.2716E	0.00018	0.00012	
5598	regulation of fat cell differentiation	0	210	-06	01	302	26
GO:003		42/171	275/23	5.3401E	0.00018	0.00012	
1647	regulation of protein stability	0	210	-06	138	389	42
GO:004		29/171	160/23	5.4721E	0.00018	0.00012	
8017	inositol lipid-mediated signaling	0	210	-06	479	622	29
GO:000		31/171	177/23	5.5099E	0.00018	0.00012	
8016	regulation of heart contraction	0	210	-06	499	636	31
GO:005		51/171	361/23	5.6314E	0.00018	0.00012	
1961	negative regulation of nervous system development	0	210	-06	799	841	51
GO:004		22/171	104/23	5.6872E	0.00018	0.00012	
2136	neurotransmitter biosynthetic process	0	210	-06	878	895	22
GO:004		32/171	186/23	5.775E-	0.00019	0.00013	
3491	protein kinase B signaling	0	210	06	061	02	32
GO:003		18/171	75/232	6.2122E	0.00020	0.00013	
0808	regulation of nucleotide biosynthetic process	0	10	-06	051	696	18
GO:004		18/171	75/232	6.2122E	0.00020	0.00013	
6323	glucose import	0	10	-06	051	696	18
GO:004		18/171	75/232	6.2122E	0.00020	0.00013	
6626	regulation of insulin receptor signaling pathway	0	10	-06	051	696	18
GO:190		18/171	75/232	6.2122E	0.00020	0.00013	
0371	regulation of purine nucleotide biosynthetic process	0	10	-06	051	696	18
GO:000	oxidative phosphorylation	21/171	97/232	6.2817E	0.00020	0.00013	21

6119		0	10	-06	053	698	
GO:004		21/171	97/232	6.2817E	0.00020	0.00013	
3502	regulation of muscle adaptation	0	10	-06	053	698	21
GO:000		28/171	153/23	6.5182E	0.00020	0.00014	
6457	protein folding	0	210	-06	695	136	28
GO:190			12/232	6.6253E	0.00020	0.00014	
0272	negative regulation of long-term synaptic potentiation	7/1710	10	-06	841	236	7
GO:004		46/171	315/23	6.6355E	0.00020	0.00014	
3062	extracellular structure organization	0	210	-06	841	236	46
GO:000		26/171	137/23	6.9698E	0.00021	0.00014	
6754	ATP biosynthetic process	0	210	-06	537	712	26
GO:007		31/171	179/23	6.9868E	0.00021	0.00014	
1772	response to BMP	0	210	-06	537	712	31
GO:007		31/171	179/23	6.9868E	0.00021	0.00014	
1773	cellular response to BMP stimulus	0	210	-06	537	712	31
GO:005		55/171	403/23	7.0069E	0.00021	0.00014	
1098	regulation of binding	0	210	-06	537	712	55
GO:004		61/171	463/23	7.0607E	0.00021	0.00014	
4270	cellular nitrogen compound catabolic process	0	210	-06	537	712	61
GO:000		25/171	129/23	7.0783E	0.00021	0.00014	
1936	regulation of endothelial cell proliferation	0	210	-06	537	712	25
GO:000		11/171	31/232	7.1554E	0.00021	0.00014	
6099	tricarboxylic acid cycle	0	10	-06	659	795	11
GO:000		21/171	98/232	7.4457E	0.00022	0.00015	
7006	mitochondrial membrane organization	0	10	-06	303	235	21
GO:001	regulation of muscle cell apoptotic process	21/171	98/232	7.4457E	0.00022	0.00015	21

0660		0	10	-06	303	235	
GO:000		33/171	197/23	7.4827E	0.00022	0.00015	
7519	skeletal muscle tissue development	0	210	-06	303	235	33
GO:200		56/171	414/23	7.5907E	0.00022	0.00015	
1233	regulation of apoptotic signaling pathway	0	210	-06	51	376	56
GO:001		22/171	106/23	7.8726E	0.00023	0.00015	
4812	muscle cell migration	0	210	-06	212	855	22
GO:000		29/171	163/23	7.9704E	0.00023	0.00015	
9266	response to temperature stimulus	0	210	-06	212	855	29
GO:003		12/171	37/232	8.0259E	0.00023	0.00015	
0810	positive regulation of nucleotide biosynthetic process	0	10	-06	212	855	12
GO:007		12/171	37/232	8.0259E	0.00023	0.00015	
2350	tricarboxylic acid metabolic process	0	10	-06	212	855	12
GO:190		12/171	37/232	8.0259E	0.00023	0.00015	
0373	positive regulation of purine nucleotide biosynthetic process	0	10	-06	212	855	12
GO:000			21/232	8.153E-	0.00023	0.00015	
6120	mitochondrial electron transport, NADH to ubiquinone	9/1710	10	06	287	907	9
GO:190		20/171	91/232	8.154E-	0.00023	0.00015	
3578	regulation of ATP metabolic process	0	10	06	287	907	20
GO:005		58/171	435/23	8.1855E	0.00023	0.00015	
1222	positive regulation of protein transport	0	210	-06	287	907	58
GO:004		45/171	308/23	8.2115E	0.00023	0.00015	
5927	positive regulation of growth	0	210	-06	287	907	45
GO:005		48/171	337/23	8.4027E	0.00023	0.00016	
0768	negative regulation of neurogenesis	0	210	-06	702	19	48
GO:003	nucleobase-containing small molecule biosynthetic process	30/171	172/23	8.439E-	0.00023	0.00016	30

4404		0	210	06	702	19	
GO:009		41/171	271/23	8.6928E	0.00024	0.00016	
0287	regulation of cellular response to growth factor stimulus	0	210	-06	21	537	41
GO:005		15/171	56/232	8.7025E	0.00024	0.00016	
1196	regulation of coenzyme metabolic process	0	10	-06	21	537	15
GO:200		19/171	84/232	8.7699E	0.00024	0.00016	
1057	reactive nitrogen species metabolic process	0	10	-06	281	586	19
GO:000		23/171	115/23	9.4518E	0.00026	0.00017	
5976	polysaccharide metabolic process	0	210	-06	046	791	23
GO:005		41/171	272/23	9.5206E	0.00026	0.00017	
1924	regulation of calcium ion transport	0	210	-06	112	837	41
GO:001		20/171	92/232	9.7048E	0.00026	0.00018	
4909	smooth muscle cell migration	0	10	-06	493	097	20
GO:000		27/171	148/23	1.0045E	0.00027	0.00018	
9145	purine nucleoside triphosphate biosynthetic process	0	210	-05	169	559	27
GO:190		27/171	148/23	1.0045E	0.00027	0.00018	
1292	nucleoside phosphate catabolic process	0	210	-05	169	559	27
GO:005		29/171	165/23	1.0174E	0.00027	0.00018	
1017	actin filament bundle assembly	0	210	-05	278	633	29
GO:000		11/171	32/232	1.0179E	0.00027	0.00018	
6101	citrate metabolic process	0	10	-05	278	633	11
GO:005		21/171	100/23	1.0376E	0.00027	0.00018	
5013	cardiac muscle cell development	0	210	-05	679	907	21
GO:190		34/171	209/23	1.0497E	0.00027	0.00019	
4950	negative regulation of establishment of protein localization	0	210	-05	875	041	34
GO:004	heterocycle catabolic process	61/171	469/23	1.0575E	0.00027	0.00019	61

6700		0	210	-05	955	095	
GO:001		28/171	157/23	1.0821E	0.00028	0.00019	
0594	regulation of endothelial cell migration	0	210	-05	477	452	28
GO:005		63/171	490/23	1.1022E	0.00028	0.00019	
0708	regulation of protein secretion	0	210	-05	874	723	63
GO:000		18/171	78/232	1.1171E	0.00028	0.00019	
5977	glycogen metabolic process	0	10	-05	877	725	18
GO:000		18/171	78/232	1.1171E	0.00028	0.00019	
6073	cellular glucan metabolic process	0	10	-05	877	725	18
GO:004		18/171	78/232	1.1171E	0.00028	0.00019	
4042	glucan metabolic process	0	10	-05	877	725	18
GO:004			17/232	1.1354E	0.00029	0.00019	
8739	cardiac muscle fiber development	8/1710	10	-05	219	959	8
GO:003		21/171	101/23	1.22E-0	0.00030	0.00021	
1532	actin cytoskeleton reorganization	0	210	5	958	146	21
GO:000		47/171	332/23	1.2216E	0.00030	0.00021	
6417	regulation of translation	0	210	-05	958	146	47
GO:000		28/171	158/23	1.2241E	0.00030	0.00021	
6941	striated muscle contraction	0	210	-05	958	146	28
GO:000		28/171	158/23	1.2241E	0.00030	0.00021	
9755	hormone-mediated signaling pathway	0	210	-05	958	146	28
GO:004		54/171	401/23	1.2465E	0.00031	0.00021	
8638	regulation of developmental growth	0	210	-05	17	291	54
GO:000		43/171	294/23	1.2577E	0.00031	0.00021	
9895	negative regulation of catabolic process	0	210	-05	17	291	43
GO:007	calcium ion import	19/171	86/232	1.2582E	0.00031	0.00021	19

0509		0	10	-05	17	291	
GO:001		22/171	109/23	1.2592E	0.00031	0.00021	
9363	pyridine nucleotide biosynthetic process	0	210	-05	17	291	22
GO:190		22/171	109/23	1.2592E	0.00031	0.00021	
3076	regulation of protein localization to plasma membrane	0	210	-05	17	291	22
GO:001		62/171	483/23	1.3568E	0.00033	0.00022	
9439	aromatic compound catabolic process	0	210	-05	404	817	62
GO:001		26/171	142/23	1.3609E	0.00033	0.00022	
6052	carbohydrate catabolic process	0	210	-05	404	817	26
GO:001		15/171	58/232	1.3848E	0.00033	0.00023	
0656	negative regulation of muscle cell apoptotic process	0	10	-05	82	101	15
GO:001		37/171	239/23	1.3894E	0.00033	0.00023	
0001	glial cell differentiation	0	210	-05	82	101	37
GO:190		13/171	45/232	1.4069E	0.00034	0.00023	
3580	positive regulation of ATP metabolic process	0	10	-05	104	296	13
GO:003		29/171	168/23	1.4535E	0.00034	0.00023	
0509	BMP signaling pathway	0	210	-05	943	869	29
GO:006		29/171	168/23	1.4535E	0.00034	0.00023	
1572	actin filament bundle organization	0	210	-05	943	869	29
GO:000		23/171	118/23	1.4715E	0.00035	0.00024	
6090	pyruvate metabolic process	0	210	-05	231	065	23
GO:000		63/171	495/23	1.5171E	0.00036	0.00024	
3013	circulatory system process	0	210	-05	175	71	63
GO:004		18/171	80/232	1.6208E	0.00038	0.00026	
6209	nitric oxide metabolic process	0	10	-05	491	292	18
GO:001	muscle cell apoptotic process	21/171	103/23	1.674E-	0.00039	0.00026	21

0657		0	210	05	276	828	
GO:003		21/171	103/23	1.674E-	0.00039	0.00026	
2231	regulation of actin filament bundle assembly	0	210	05	276	828	21
GO:190		21/171	103/23	1.674E-	0.00039	0.00026	
3426	regulation of reactive oxygen species biosynthetic process	0	210	05	276	828	21
GO:009		35/171	223/23	1.7386E	0.00040	0.00027	
0150	establishment of protein localization to membrane	0	210	-05	627	751	35
GO:004		34/171	214/23	1.7541E	0.00040	0.00027	
8639	positive regulation of developmental growth	0	210	-05	827	888	34
GO:000		52/171	386/23	1.7725E	0.00040	0.00027	
6914	autophagy	0	210	-05	928	957	52
GO:006		52/171	386/23	1.7725E	0.00040	0.00027	
1919	process utilizing autophagic mechanism	0	210	-05	928	957	52
GO:009		13/171	46/232	1.8296E	0.00042	0.00028	
0279	regulation of calcium ion import	0	10	-05	08	744	13
GO:000		25/171	136/23	1.8433E	0.00042	0.00028	
9166	nucleotide catabolic process	0	210	-05	097	755	25
GO:000		42/171	289/23	1.8447E	0.00042	0.00028	
6936	muscle contraction	0	210	-05	097	755	42
GO:000		30/171	179/23	1.8875E	0.00042	0.00029	
1659	temperature homeostasis	0	210	-05	739	194	30
GO:003		30/171	179/23	1.8875E	0.00042	0.00029	
4767	positive regulation of ion transmembrane transport	0	210	-05	739	194	30
GO:006		33/171	206/23	1.9481E	0.00043	0.00029	
0538	skeletal muscle organ development	0	210	-05	898	985	33
GO:004	cellular polysaccharide metabolic process	21/171	104/23	1.9537E	0.00043	0.00029	21

4264		0	210	-05	898	985	
GO:000		28/171	162/23	1.9779E	0.00044	0.00030	
9156	ribonucleoside monophosphate biosynthetic process	0	210	-05	272	241	28
GO:003		57/171	438/23	2.0004E	0.00044	0.00030	
2386	regulation of intracellular transport	0	210	-05	603	467	57
GO:006		14/171	53/232	2.053E-	0.00045	0.00031	
1900	glial cell activation	0	10	05	604	151	14
GO:190		19/171	89/232	2.1109E	0.00046	0.00031	
4035	regulation of epithelial cell apoptotic process	0	10	-05	711	907	19
GO:000		16/171	67/232	2.1497E	0.00047	0.00032	
6635	fatty acid beta-oxidation	0	10	-05	391	371	16
GO:000		23/171	121/23	2.2496E	0.00049	0.00033	
8286	insulin receptor signaling pathway	0	210	-05	221	621	23
GO:000		23/171	121/23	2.2496E	0.00049	0.00033	
9132	nucleoside diphosphate metabolic process	0	210	-05	221	621	23
GO:005		47/171	340/23	2.2781E	0.00049	0.00033	
0900	leukocyte migration	0	210	-05	553	848	47
GO:007		22/171	113/23	2.2817E	0.00049	0.00033	
2525	pyridine-containing compound biosynthetic process	0	210	-05	553	848	22
GO:006		27/171	155/23	2.3917E	0.00051	0.00035	
2013	positive regulation of small molecule metabolic process	0	210	-05	749	349	27
GO:007		43/171	302/23	2.4453E	0.00052	0.00036	
2001	renal system development	0	210	-05	714	008	43
GO:007		19/171	90/232	2.493E-	0.00053	0.00036	
2655	establishment of protein localization to mitochondrion	0	10	05	544	575	19
GO:000	purine ribonucleoside triphosphate biosynthetic process	26/171	147/23	2.5558E	0.00054	0.00037	26

9206		0	210	-05	691	358	
GO:001		14/171	54/232	2.585E-	0.00054	0.00037	
4009	glial cell proliferation	0	10	05	915	511	14
GO:004		12/171	41/232	2.5957E	0.00054	0.00037	
6326	positive regulation of glucose import	0	10	-05	915	511	12
GO:004		20/171	98/232	2.5984E	0.00054	0.00037	
6031	ADP metabolic process	0	10	-05	915	511	20
GO:007		34/171	218/23	2.6038E	0.00054	0.00037	
0374	positive regulation of ERK1 and ERK2 cascade	0	210	-05	915	511	34
GO:004		16/171	68/232	2.6206E	0.00054	0.00037	
5428	regulation of nitric oxide biosynthetic process	0	10	-05	929	52	16
GO:200		33/171	209/23	2.6354E	0.00054	0.00037	
0027	regulation of animal organ morphogenesis	0	210	-05	929	52	33
GO:001		21/171	106/23	2.6421E	0.00054	0.00037	
9359	nicotinamide nucleotide biosynthetic process	0	210	-05	929	52	21
GO:005		21/171	106/23	2.6421E	0.00054	0.00037	
5006	cardiac cell development	0	210	-05	929	52	21
GO:005		11/171	35/232	2.6787E	0.00055	0.00037	
1084	'de novo' posttranslational protein folding	0	10	-05	493	906	11
GO:004		41/171	284/23	2.7008E	0.00055	0.00038	
4262	cellular carbohydrate metabolic process	0	210	-05	753	084	41
GO:000		18/171	83/232	2.7586E	0.00056	0.00038	
6942	regulation of striated muscle contraction	0	10	-05	663	705	18
GO:000		61/171	484/23	2.7643E	0.00056	0.00038	
8015	blood circulation	0	210	-05	663	705	61
GO:003	negative regulation of cellular catabolic process	37/171	247/23	2.9145E	0.00059	0.00040	37

1330		0	210	-05	533	666	
GO:006		41/171	285/23	2.9345E	0.00059	0.00040	
1448	connective tissue development	0	210	-05	732	801	41
GO:007		23/171	123/23	2.9568E	0.00059	0.00040	
1621	granulocyte chemotaxis	0	210	-05	978	97	23
GO:000		17/171	76/232	2.997E-	0.00060	0.00041	
6809	nitric oxide biosynthetic process	0	10	05	583	382	17
GO:000		21/171	107/23	3.0619E	0.00060	0.00041	
3014	renal system process	0	210	-05	842	559	21
GO:000		21/171	107/23	3.0619E	0.00060	0.00041	
6140	regulation of nucleotide metabolic process	0	210	-05	842	559	21
GO:000		21/171	107/23	3.0619E	0.00060	0.00041	
9185	ribonucleoside diphosphate metabolic process	0	210	-05	842	559	21
GO:001		21/171	107/23	3.0619E	0.00060	0.00041	
4896	muscle hypertrophy	0	210	-05	842	559	21
GO:004		21/171	107/23	3.0619E	0.00060	0.00041	
8145	regulation of fibroblast proliferation	0	210	-05	842	559	21
GO:190		36/171	239/23	3.2932E	0.00065	0.00044	
3522	regulation of blood circulation	0	210	-05	216	548	36
GO:000		27/171	158/23	3.3983E	0.00066	0.00045	
9127	purine nucleoside monophosphate biosynthetic process	0	210	-05	845	66	27
GO:000		27/171	158/23	3.3983E	0.00066	0.00045	
9168	purine ribonucleoside monophosphate biosynthetic process	0	210	-05	845	66	27
GO:006		25/171	141/23	3.4755E	0.00068	0.00046	
1041	regulation of wound healing	0	210	-05	134	541	25
GO:011	regulation of actomyosin structure organization	20/171	100/23	3.5324E	0.00068	0.00047	20

0020		0	210	-05	944	094	
GO:003		21/171	108/23	3.5404E	0.00068	0.00047	
0048	actin filament-based movement	0	210	-05	944	094	21
GO:200		17/171	77/232	3.5848E	0.00069	0.00047	
1259	positive regulation of cation channel activity	0	10	-05	5	474	17
GO:000		11/171	36/232	3.6017E	0.00069	0.00047	
6458	'de novo' protein folding	0	10	-05	5	474	11
GO:004		36/171	240/23	3.6047E	0.00069	0.00047	
3393	regulation of protein binding	0	210	-05	5	474	36
GO:000		26/171	150/23	3.6658E	0.00070	0.00048	
9201	ribonucleoside triphosphate biosynthetic process	0	210	-05	446	12	26
GO:001		18/171	85/232	3.8681E	0.00073	0.00050	
4910	regulation of smooth muscle cell migration	0	10	-05	607	279	18
GO:004		18/171	85/232	3.8681E	0.00073	0.00050	
8708	astrocyte differentiation	0	10	-05	607	279	18
GO:190		18/171	85/232	3.8681E	0.00073	0.00050	
1863	positive regulation of muscle tissue development	0	10	-05	607	279	18
GO:010		25/171	142/23	3.9274E	0.00074	0.00050	
6106	cold-induced thermogenesis	0	210	-05	251	719	25
GO:012		25/171	142/23	3.9274E	0.00074	0.00050	
0161	regulation of cold-induced thermogenesis	0	210	-05	251	719	25
GO:005		32/171	204/23	3.9548E	0.00074	0.00050	
1224	negative regulation of protein transport	0	210	-05	529	909	32
GO:001		15/171	63/232	4.0066E	0.00075	0.00051	
0830	regulation of myotube differentiation	0	10	-05	263	41	15
GO:004	fibroblast proliferation	21/171	109/23	4.0848E	0.00076	0.00052	21

8144		0	210	-05	486	246	
GO:000		20/171	101/23	4.1034E	0.00076	0.00052	
3300	cardiac muscle hypertrophy	0	210	-05	588	315	20
GO:006		42/171	299/23	4.1675E	0.00077	0.00052	
0326	cell chemotaxis	0	210	-05	537	964	42
GO:001		40/171	280/23	4.2833E	0.00079	0.00054	
0631	epithelial cell migration	0	210	-05	439	263	40
GO:009		28/171	169/23	4.3646E	0.00080	0.00054	
8727	maintenance of cell number	0	210	-05	435	943	28
GO:200		28/171	169/23	4.3646E	0.00080	0.00054	
1242	regulation of intrinsic apoptotic signaling pathway	0	210	-05	435	943	28
GO:003		46/171	339/23	4.4092E	0.00081	0.00055	
1589	cell-substrate adhesion	0	210	-05	002	33	46
GO:004		18/171	86/232	4.5589E	0.00083	0.00057	
3648	dicarboxylic acid metabolic process	0	10	-05	49	03	18
GO:000		40/171	281/23	4.646E-	0.00084	0.00057	
1822	kidney development	0	210	05	818	937	40
GO:000		21/171	110/23	4.7028E	0.00085	0.00058	
1843	neural tube closure	0	210	-05	524	42	21
GO:004		19/171	94/232	4.7139E	0.00085	0.00058	
2866	pyruvate biosynthetic process	0	10	-05	524	42	19
GO:001		20/171	102/23	4.7552E	0.00085	0.00058	
0522	regulation of calcium ion transport into cytosol	0	210	-05	792	602	20
GO:001		49/171	370/23	4.7581E	0.00085	0.00058	
0876	lipid localization	0	210	-05	792	602	49
GO:003	regulation of polysaccharide biosynthetic process	11/171	37/232	4.7859E	0.00085	0.00058	11

2885		0	10	-05	838	634	
GO:000			20/232	4.8147E	0.00085	0.00058	
2577	regulation of antigen processing and presentation	8/1710	10	-05	838	634	8
GO:004			20/232	4.8147E	0.00085	0.00058	
5821	positive regulation of glycolytic process	8/1710	10	-05	838	634	8
GO:004		13/171	50/232	4.8341E	0.00085	0.00058	
5981	positive regulation of nucleotide metabolic process	0	10	-05	838	634	13
GO:190		13/171	50/232	4.8341E	0.00085	0.00058	
0544	positive regulation of purine nucleotide metabolic process	0	10	-05	838	634	13
GO:004		28/171	170/23	4.8639E	0.00086	0.00058	
6890	regulation of lipid biosynthetic process	0	210	-05	106	817	28
GO:000		29/171	179/23	4.9073E	0.00086	0.00058	
6839	mitochondrial transport	0	210	-05	351	984	29
GO:190		29/171	179/23	4.9073E	0.00086	0.00058	
1861	regulation of muscle tissue development	0	210	-05	351	984	29
GO:009		40/171	282/23	5.0364E	0.00088	0.00060	
0132	epithelium migration	0	210	-05	357	354	40
GO:000			11/232	5.2998E	0.00092	0.00063	
2579	positive regulation of antigen processing and presentation	6/1710	10	-05	423	132	6
GO:007			11/232	5.2998E	0.00092	0.00063	
0831	basement membrane assembly	6/1710	10	-05	423	132	6
GO:006		21/171	111/23	5.4029E	0.00093	0.00064	
0606	tube closure	0	210	-05	856	111	21
GO:004		28/171	171/23	5.4142E	0.00093	0.00064	
3409	negative regulation of MAPK cascade	0	210	-05	856	111	28
GO:007	protein localization to mitochondrion	19/171	95/232	5.4905E	0.00094	0.00064	19

0585		0	10	-05	186	336	
GO:000		20/171	103/23	5.4977E	0.00094	0.00064	
9135	purine nucleoside diphosphate metabolic process	0	210	-05	186	336	20
GO:000		20/171	103/23	5.4977E	0.00094	0.00064	
9179	purine ribonucleoside diphosphate metabolic process	0	210	-05	186	336	20
GO:190		20/171	103/23	5.4977E	0.00094	0.00064	
0542	regulation of purine nucleotide metabolic process	0	210	-05	186	336	20
GO:000		54/171	423/23	5.5251E	0.00094	0.00064	
6816	calcium ion transport	0	210	-05	379	468	54
GO:003		16/171	72/232	5.5449E	0.00094	0.00064	
0239	myofibril assembly	0	10	-05	44	51	16
GO:003		25/171	145/23	5.6173E	0.00095	0.00065	
1099	regeneration	0	210	-05	397	163	25
GO:009		40/171	284/23	5.9083E	0.00100	0.00068	
0130	tissue migration	0	210	-05	047	34	40
GO:005		31/171	199/23	5.9272E	0.00100	0.00068	
0679	positive regulation of epithelial cell proliferation	0	210	-05	077	36	31
GO:003		59/171	476/23	5.9885E	0.00100	0.00068	
3674	positive regulation of kinase activity	0	210	-05	821	869	59
GO:001		14/171	58/232	6.1153E	0.00102	0.00070	
0662	regulation of striated muscle cell apoptotic process	0	10	-05	659	124	14
GO:190			26/232	6.1829E	0.00103	0.00070	
3579	negative regulation of ATP metabolic process	9/1710	10	-05	497	696	9
GO:000		18/171	88/232	6.2763E	0.00104	0.00071	
6112	energy reserve metabolic process	0	10	-05	461	355	18
GO:005	regulation of stress fiber assembly	18/171	88/232	6.2763E	0.00104	0.00071	18

1492		0	10	-05	461	355	
GO:003		22/171	121/23	6.7897E	0.00112	0.00076	
2411	positive regulation of transporter activity	0	210	-05	366	754	22
GO:190		22/171	121/23	6.7897E	0.00112	0.00076	
3409	reactive oxygen species biosynthetic process	0	210	-05	366	754	22
GO:003		32/171	210/23	7.0185E	0.00115	0.00079	
3157	regulation of intracellular protein transport	0	210	-05	825	117	32
GO:004		25/171	147/23	7.0806E	0.00115	0.00079	
2133	neurotransmitter metabolic process	0	210	-05	981	224	25
GO:009	negative regulation of transmembrane receptor protein serine/threonine kinase	21/171	113/23	7.0875E	0.00115	0.00079	
0101	signaling pathway	0	210	-05	981	224	21
GO:190		21/171	113/23	7.0875E	0.00115	0.00079	
4019	epithelial cell apoptotic process	0	210	-05	981	224	21
GO:007		54/171	427/23	7.1113E	0.00116	0.00079	
1900	regulation of protein serine/threonine kinase activity	0	210	-05	045	268	54
GO:004		15/171	66/232	7.1397E	0.00116	0.00079	
6324	regulation of glucose import	0	10	-05	082	293	15
GO:000		12/171	45/232	7.1732E	0.00116	0.00079	
1774	microglial cell activation	0	10	-05	082	293	12
GO:000		12/171	45/232	7.1732E	0.00116	0.00079	
2269	leukocyte activation involved in inflammatory response	0	10	-05	082	293	12
GO:199		26/171	156/23	7.2706E	0.00116	0.00079	
0845	adaptive thermogenesis	0	210	-05	093	3	26
GO:003			21/232	7.2758E	0.00116	0.00079	
0813	positive regulation of nucleotide catabolic process	8/1710	10	-05	093	3	8
GO:003	positive regulation of superoxide anion generation	8/1710	21/232	7.2758E	0.00116	0.00079	8

2930			10	-05	093	3	
GO:005			21/232	7.2758E	0.00116	0.00079	
1197	positive regulation of coenzyme metabolic process	8/1710	10	-05	093	3	8
GO:001		20/171	105/23	7.2984E	0.00116	0.00079	
4897	striated muscle hypertrophy	0	210	-05	093	3	20
GO:190		18/171	89/232	7.3323E	0.00116	0.00079	
0076	regulation of cellular response to insulin stimulus	0	10	-05	093	3	18
GO:000			16/232	7.3527E	0.00116	0.00079	
6103	2-oxoglutarate metabolic process	7/1710	10	-05	093	3	7
GO:006			16/232	7.3527E	0.00116	0.00079	
0192	negative regulation of lipase activity	7/1710	10	-05	093	3	7
GO:007			16/232	7.3527E	0.00116	0.00079	
1498	cellular response to fluid shear stress	7/1710	10	-05	093	3	7
GO:001		27/171	165/23	7.3802E	0.00116	0.00079	
9827	stem cell population maintenance	0	210	-05	213	382	27
GO:006		13/171	52/232	7.5316E	0.00118	0.00080	
1077	chaperone-mediated protein folding	0	10	-05	279	793	13
GO:000		56/171	449/23	7.707E-	0.00120	0.00082	
1819	positive regulation of cytokine production	0	210	05	709	453	56
GO:002		47/171	357/23	7.7714E	0.00121	0.00082	
2411	cellular component disassembly	0	210	-05	391	919	47
GO:003		21/171	114/23	8.0934E	0.00126	0.00086	
2414	positive regulation of ion transmembrane transporter activity	0	210	-05	084	125	21
GO:190		11/171	39/232	8.181E-	0.00127	0.00086	
1998	toxin transport	0	10	05	11	826	11
GO:000	isoprenoid metabolic process	17/171	82/232	8.3329E	0.00129	0.00088	17

6720		0	10	-05	127	203	
GO:004		44/171	328/23	8.3766E	0.00129	0.00088	
5862	positive regulation of proteolysis	0	210	-05	191	247	44
GO:004		20/171	106/23	8.3812E	0.00129	0.00088	
5185	maintenance of protein location	0	210	-05	191	247	20
GO:000		18/171	90/232	8.5421E	0.00130	0.00089	
6096	glycolytic process	0	10	-05	979	468	18
GO:004		18/171	90/232	8.5421E	0.00130	0.00089	
2770	signal transduction in response to DNA damage	0	10	-05	979	468	18
GO:003			27/232	8.6701E	0.00132	0.00090	
3014	tetrapyrrole biosynthetic process	9/1710	10	-05	226	321	9
GO:190			27/232	8.6701E	0.00132	0.00090	
0543	negative regulation of purine nucleotide metabolic process	9/1710	10	-05	226	321	9
GO:005		56/171	451/23	8.6914E	0.00132	0.00090	
0808	synapse organization	0	210	-05	226	321	56
GO:000		26/171	158/23	9.0416E	0.00137	0.00093	
8654	phospholipid biosynthetic process	0	210	-05	197	716	26
GO:000		16/171	75/232	9.3347E	0.00140	0.00096	
0271	polysaccharide biosynthetic process	0	10	-05	913	254	16
GO:005		16/171	75/232	9.3347E	0.00140	0.00096	
1193	regulation of cofactor metabolic process	0	10	-05	913	254	16
GO:000		19/171	99/232	9.8529E	0.00147	0.00101	
6892	post-Golgi vesicle-mediated transport	0	10	-05	906	031	19
GO:009		19/171	99/232	9.8529E	0.00147	0.00101	
8876	vesicle-mediated transport to the plasma membrane	0	10	-05	906	031	19
GO:006	cardiac muscle contraction	22/171	124/23	9.9105E	0.00147	0.00101	22

0048		0	210	-05	906	031	
GO:000		18/171	91/232	9.9245E	0.00147	0.00101	
1938	positive regulation of endothelial cell proliferation	0	10	-05	906	031	18
GO:000		18/171	91/232	9.9245E	0.00147	0.00101	
6757	ATP generation from ADP	0	10	-05	906	031	18
GO:004		31/171	205/23	0.00010	0.00155	0.00105	
3112	receptor metabolic process	0	210	441	07	925	31
GO:190		21/171	116/23	0.00010	0.00155	0.00105	
3828	negative regulation of cellular protein localization	0	210	493	07	925	21
GO:000		11/171	40/232	0.00010	0.00155	0.00105	
0266	mitochondrial fission	0	10	538	07	925	11
GO:008		11/171	40/232	0.00010	0.00155	0.00105	
6004	regulation of cardiac muscle cell contraction	0	10	538	07	925	11
GO:190		11/171	40/232	0.00010	0.00155	0.00105	
3053	regulation of extracellular matrix organization	0	10	538	07	925	11
GO:004			22/232	0.00010	0.00157	0.00107	
6628	positive regulation of insulin receptor signaling pathway	8/1710	10	697	008	249	8
GO:001		51/171	403/23	0.00010	0.00160	0.00109	
0639	negative regulation of organelle organization	0	210	951	337	523	51
GO:004		14/171	61/232	0.00011	0.00160	0.00109	
5600	positive regulation of fat cell differentiation	0	10	018	923	922	14
GO:005		27/171	169/23	0.00011	0.00162	0.00111	
0821	protein stabilization	0	210	2	81	212	27
GO:003		22/171	125/23	0.00011	0.00162	0.00111	
2273	positive regulation of protein polymerization	0	210	203	81	212	22
GO:004	Golgi vesicle transport	37/171	263/23	0.00011	0.00162	0.00111	37

8193		0	210	239	92	287	
GO:003		57/171	466/23	0.00011	0.00164	0.00112	
1346	positive regulation of cell projection organization	0	210	352	149	126	57
GO:004		17/171	84/232	0.00011	0.00164	0.00112	
5844	positive regulation of striated muscle tissue development	0	10	417	204	164	17
GO:004		17/171	84/232	0.00011	0.00164	0.00112	
8636	positive regulation of muscle organ development	0	10	417	204	164	17
GO:003		31/171	206/23	0.00011	0.00164	0.00112	
4329	cell junction assembly	0	210	44	204	164	31
GO:004			17/232	0.00011	0.00167	0.00114	
5725	positive regulation of glycogen biosynthetic process	7/1710	10	705	596	481	7
GO:001		21/171	117/23	0.00011	0.00169	0.00115	
4020	primary neural tube formation	0	210	913	378	698	21
GO:000			28/232	0.00011	0.00169	0.00115	
5979	regulation of glycogen biosynthetic process	9/1710	10	945	378	698	9
GO:001			28/232	0.00011	0.00169	0.00115	
0962	regulation of glucan biosynthetic process	9/1710	10	945	378	698	9
GO:004			28/232	0.00011	0.00169	0.00115	
5980	negative regulation of nucleotide metabolic process	9/1710	10	945	378	698	9
GO:004		32/171	216/23	0.00012	0.00170	0.00116	
8545	response to steroid hormone	0	210	083	924	754	32
GO:001		24/171	143/23	0.00012	0.00170	0.00116	
0634	positive regulation of epithelial cell migration	0	210	113	924	754	24
GO:001		25/171	152/23	0.00012	0.00173	0.00118	
0675	regulation of cellular carbohydrate metabolic process	0	210	336	649	615	25
GO:006	cytosolic calcium ion transport	27/171	170/23	0.00012	0.00173	0.00118	27

0401		0	210	397	756	689	
GO:007		51/171	405/23	0.00012	0.00173	0.00118	
0997	neuron death	0	210	403	756	689	51
GO:190		19/171	101/23	0.00013	0.00181	0.00124	
1222	regulation of NIK/NF-kappaB signaling	0	210	011	845	214	19
GO:000		14/171	62/232	0.00013	0.00183	0.00125	
6721	terpenoid metabolic process	0	10	277	132	093	14
GO:001		14/171	62/232	0.00013	0.00183	0.00125	
0658	striated muscle cell apoptotic process	0	10	277	132	093	14
GO:007		14/171	62/232	0.00013	0.00183	0.00125	
0988	demethylation	0	10	277	132	093	14
GO:008		14/171	62/232	0.00013	0.00183	0.00125	
6003	cardiac muscle cell contraction	0	10	277	132	093	14
GO:000		18/171	93/232	0.00013	0.00183	0.00125	
9062	fatty acid catabolic process	0	10	291	132	093	18
GO:004		18/171	93/232	0.00013	0.00183	0.00125	
2116	macrophage activation	0	10	291	132	093	18
GO:003		46/171	355/23	0.00013	0.00184	0.00126	
1331	positive regulation of cellular catabolic process	0	210	427	563	071	46
GO:000		27/171	171/23	0.00013	0.00187	0.00128	
0082	G1/S transition of mitotic cell cycle	0	210	706	96	391	27
GO:004		54/171	438/23	0.00013	0.00189	0.00129	
5860	positive regulation of protein kinase activity	0	210	853	527	461	54
GO:005		12/171	48/232	0.00014	0.00191	0.00131	
0435	amyloid-beta metabolic process	0	10	135	935	106	12
GO:005	brown fat cell differentiation	12/171	48/232	0.00014	0.00191	0.00131	12

0873		0	10	135	935	106	
GO:190		12/171	48/232	0.00014	0.00191	0.00131	
3115	regulation of actin filament-based movement	0	10	135	935	106	12
GO:003		30/171	199/23	0.00014	0.00191	0.00131	
0522	intracellular receptor signaling pathway	0	210	16	935	106	30
GO:001		22/171	127/23	0.00014	0.00192	0.00131	
4065	phosphatidylinositol 3-kinase signaling	0	210	245	642	589	22
GO:004		32/171	218/23	0.00014	0.00194	0.00132	
2445	hormone metabolic process	0	210	39	147	617	32
GO:001		35/171	247/23	0.00014	0.00198	0.00135	
0506	regulation of autophagy	0	210	719	128	337	35
GO:001		23/171	136/23	0.00014	0.00198	0.00135	
0821	regulation of mitochondrion organization	0	210	775	425	539	23
GO:200		10/171	35/232	0.00015	0.00204	0.00139	
0249	regulation of actin cytoskeleton reorganization	0	10	261	477	673	10
GO:000		17/171	86/232	0.00015	0.00206	0.00141	
7044	cell-substrate junction assembly	0	10	459	664	167	17
GO:004		14/171	63/232	0.00015	0.00211	0.00144	
5454	cell redox homeostasis	0	10	926	451	437	14
GO:004		14/171	63/232	0.00015	0.00211	0.00144	
5661	regulation of myoblast differentiation	0	10	926	451	437	14
GO:007		57/171	472/23	0.00015	0.00211	0.00144	
0838	divalent metal ion transport	0	210	962	451	437	57
GO:190		57/171	472/23	0.00015	0.00211	0.00144	
3532	positive regulation of secretion by cell	0	210	962	451	437	57
GO:190	regulation of neuron death	47/171	368/23	0.00016	0.00212	0.00145	47

1214		0	210	106	874	409	
GO:009		33/171	229/23	0.00016	0.00214	0.00146	
7191	extrinsic apoptotic signaling pathway	0	210	305	536	544	33
GO:190		33/171	229/23	0.00016	0.00214	0.00146	
3320	regulation of protein modification by small protein conjugation or removal	0	210	305	536	544	33
GO:000		23/171	137/23	0.00016	0.00217	0.00148	
2221	pattern recognition receptor signaling pathway	0	210	538	115	306	23
GO:001		34/171	239/23	0.00016	0.00220	0.00150	
6485	protein processing	0	210	832	061	318	34
GO:009		24/171	146/23	0.00016	0.00220	0.00150	
7530	granulocyte migration	0	210	838	061	318	24
GO:000		19/171	103/23	0.00017	0.00220	0.00150	
6165	nucleoside diphosphate phosphorylation	0	210	028	572	667	19
GO:001		19/171	103/23	0.00017	0.00220	0.00150	
7015	regulation of transforming growth factor beta receptor signaling pathway	0	210	028	572	667	19
GO:003		19/171	103/23	0.00017	0.00220	0.00150	
0038	contractile actin filament bundle assembly	0	210	028	572	667	19
GO:004		19/171	103/23	0.00017	0.00220	0.00150	
3149	stress fiber assembly	0	210	028	572	667	19
GO:004		21/171	120/23	0.00017	0.00222	0.00152	
5727	positive regulation of translation	0	210	249	651	087	21
GO:005			13/232	0.00017	0.00222	0.00152	
5119	relaxation of cardiac muscle	6/1710	10	303	651	087	6
GO:006			13/232	0.00017	0.00222	0.00152	
0391	positive regulation of SMAD protein signal transduction	6/1710	10	303	651	087	6
GO:004	positive regulation of cell cycle	45/171	349/23	0.00017	0.00226	0.00155	45

5787		0	210	679	99	051	
GO:005		16/171	79/232	0.00017	0.00227	0.00155	
1279	regulation of release of sequestered calcium ion into cytosol	0	10	8	799	604	16
GO:005		31/171	211/23	0.00017	0.00227	0.00155	
1216	cartilage development	0	210	836	799	604	31
GO:001		17/171	87/232	0.00017	0.00227	0.00155	
0717	regulation of epithelial to mesenchymal transition	0	10	913	799	604	17
GO:007		17/171	87/232	0.00017	0.00227	0.00155	
0252	actin-mediated cell contraction	0	10	913	799	604	17
GO:006			18/232	0.00017	0.00227	0.00155	
0416	response to growth hormone	7/1710	10	937	799	604	7
GO:000		50/171	401/23	0.00018	0.00231	0.00158	
9991	response to extracellular stimulus	0	210	301	921	42	50
GO:000		52/171	422/23	0.00018	0.00233	0.00159	
9896	positive regulation of catabolic process	0	210	482	709	641	52
GO:190		24/171	147/23	0.00018	0.00236	0.00161	
2904	negative regulation of supramolecular fiber organization	0	210	742	479	533	24
GO:007		25/171	156/23	0.00018	0.00236	0.00161	
1383	cellular response to steroid hormone stimulus	0	210	795	637	641	25
GO:007		57/171	475/23	0.00018	0.00236	0.00161	
2511	divalent inorganic cation transport	0	210	854	877	805	57
GO:003		14/171	64/232	0.00019	0.00238	0.00162	
2233	positive regulation of actin filament bundle assembly	0	10	019	429	865	14
GO:003		31/171	212/23	0.00019	0.00243	0.00166	
5264	multicellular organism growth	0	210	446	257	163	31
GO:001	negative regulation of striated muscle cell apoptotic process	10/171	36/232	0.00019	0.00245	0.00168	10

0664		0	10	745	946		
GO:190		10/171	36/232	0.00019	0.00245		
4037	positive regulation of epithelial cell apoptotic process	0	10	745	946	0.00168	10
GO:001		18/171	96/232	0.00020	0.00250	0.00171	
4066	regulation of phosphatidylinositol 3-kinase signaling	0	10	216	744	277	18
GO:012		18/171	96/232	0.00020	0.00250	0.00171	
0162	positive regulation of cold-induced thermogenesis	0	10	216	744	277	18
GO:000		23/171	139/23	0.00020	0.00255	0.00174	
2758	innate immune response-activating signal transduction	0	210	632	369	437	23
GO:004		25/171	157/23	0.00020	0.00257	0.00175	
5834	positive regulation of lipid metabolic process	0	210	818	122	634	25
GO:000		40/171	301/23	0.00021	0.00258	0.00176	
8202	steroid metabolic process	0	210	011	964	892	40
GO:000		11/171	43/232	0.00021	0.00262	0.00179	
5978	glycogen biosynthetic process	0	10	36	157	073	11
GO:000		11/171	43/232	0.00021	0.00262	0.00179	
9250	glucan biosynthetic process	0	10	36	157	073	11
GO:001		12/171	50/232	0.00021	0.00262	0.00179	
0828	positive regulation of glucose transmembrane transport	0	10	468	932	602	12
GO:003			24/232	0.00021	0.00263	0.00179	
2928	regulation of superoxide anion generation	8/1710	10	538	228	804	8
GO:004		28/171	185/23	0.00021	0.00264	0.00180	
4843	cell cycle G1/S phase transition	0	210	704	158	44	28
GO:004		28/171	185/23	0.00021	0.00264	0.00180	
5766	positive regulation of angiogenesis	0	210	704	158	44	28
GO:004	gland development	56/171	467/23	0.00021	0.00264	0.00180	56

8732		0	210	764	339	563	
GO:004		19/171	105/23	0.00022	0.00266	0.00182	
6939	nucleotide phosphorylation	0	210	094	907	318	19
GO:190		19/171	105/23	0.00022	0.00266	0.00182	
3844	regulation of cellular response to transforming growth factor beta stimulus	0	210	094	907	318	19
GO:190		30/171	204/23	0.00022	0.00266	0.00182	
4018	positive regulation of vasculature development	0	210	113	907	318	30
GO:003		57/171	478/23	0.00022	0.00267	0.00182	
4765	regulation of ion transmembrane transport	0	210	214	581	778	57
GO:001		27/171	176/23	0.00022	0.00267	0.00182	
6202	regulation of striated muscle tissue development	0	210	286	891	99	27
GO:006		37/171	272/23	0.00022	0.00268	0.00183	
0485	mesenchyme development	0	210	382	491	399	37
GO:009		39/171	292/23	0.00022	0.00270	0.00184	
7193	intrinsic apoptotic signaling pathway	0	210	562	101	499	39
GO:006		14/171	65/232	0.00022	0.00270	0.00184	
0389	pathway-restricted SMAD protein phosphorylation	0	10	616	185	557	14
GO:006		15/171	73/232	0.00023	0.00283	0.00193	
0191	regulation of lipase activity	0	10	752	186	437	15
GO:005		30/171	205/23	0.00024	0.00286	0.00195	
1099	positive regulation of binding	0	210	114	908	98	30
GO:003		33/171	234/23	0.00024	0.00291	0.00198	
3002	muscle cell proliferation	0	210	523	185	901	33
GO:000		22/171	132/23	0.00025	0.00299	0.00204	
1841	neural tube formation	0	210	258	019	252	22
GO:004	superoxide anion generation	10/171	37/232	0.00025	0.00299	0.00204	10

2554		0	10	285	019	252	
GO:003			19/232	0.00026	0.00312	0.00213	
5994	response to muscle stretch	7/1710	10	599	064	163	7
GO:007			19/232	0.00026	0.00312	0.00213	
0875	positive regulation of glycogen metabolic process	7/1710	10	599	064	163	7
GO:009			19/232	0.00026	0.00312	0.00213	
0030	regulation of steroid hormone biosynthetic process	7/1710	10	599	064	163	7
GO:003		11/171	44/232	0.00026	0.00312	0.00213	
2881	regulation of polysaccharide metabolic process	0	10	602	064	163	11
GO:004		31/171	216/23	0.00027	0.00319	0.00217	
8762	mesenchymal cell differentiation	0	210	258	117	981	31
GO:004		17/171	90/232	0.00027	0.00320	0.00218	
3535	regulation of blood vessel endothelial cell migration	0	10	421	382	845	17
GO:000		50/171	408/23	0.00027	0.00323	0.00220	
1933	negative regulation of protein phosphorylation	0	210	724	276	822	50
GO:190		15/171	74/232	0.00027	0.00323	0.00220	
1224	positive regulation of NIK/NF-kappaB signaling	0	10	789	39	9	15
GO:000		16/171	82/232	0.00027	0.00323	0.00221	
6637	acyl-CoA metabolic process	0	10	936	811	187	16
GO:003		16/171	82/232	0.00027	0.00323	0.00221	
5383	thioester metabolic process	0	10	936	811	187	16
GO:001		22/171	133/23	0.00028	0.00326	0.00222	
6999	antibiotic metabolic process	0	210	195	17	799	22
GO:200			14/232	0.00028	0.00326	0.00223	
0052	positive regulation of non-canonical Wnt signaling pathway	6/1710	10	393	466	001	6
GO:003	NIK/NF-kappaB signaling	19/171	107/23	0.00028	0.00326	0.00223	19

8061		0	210	432	466	001	
GO:000			31/232	0.00028	0.00326	0.00223	
6506	GPI anchor biosynthetic process	9/1710	10	5	466	001	9
GO:009			31/232	0.00028	0.00326	0.00223	
0075	relaxation of muscle	9/1710	10	5	466	001	9
GO:190			31/232	0.00028	0.00326	0.00223	
5476	negative regulation of protein localization to membrane	9/1710	10	5	466	001	9
GO:001		13/171	59/232	0.00029	0.00335	0.00229	
0524	positive regulation of calcium ion transport into cytosol	0	10	596	829	396	13
GO:190		13/171	59/232	0.00029	0.00335	0.00229	
1016	regulation of potassium ion transmembrane transporter activity	0	10	596	829	396	13
GO:000			25/232	0.00029	0.00335	0.00229	
2478	antigen processing and presentation of exogenous peptide antigen	8/1710	10	638	829	396	8
GO:007			25/232	0.00029	0.00335	0.00229	
1711	basement membrane organization	8/1710	10	638	829	396	8
GO:190			25/232	0.00029	0.00335	0.00229	
4376	negative regulation of protein localization to cell periphery	8/1710	10	638	829	396	8
GO:001		40/171	306/23	0.00029	0.00335	0.00229	
6042	lipid catabolic process	0	210	662	829	396	40
GO:004		29/171	198/23	0.00029	0.00337	0.00230	
3542	endothelial cell migration	0	210	91	758	714	29
GO:003		18/171	99/232	0.00030	0.00337	0.00230	
3865	nucleoside bisphosphate metabolic process	0	10	103	758	714	18
GO:003		18/171	99/232	0.00030	0.00337	0.00230	
3875	ribonucleoside bisphosphate metabolic process	0	10	103	758	714	18
GO:003	purine nucleoside bisphosphate metabolic process	18/171	99/232	0.00030	0.00337	0.00230	18

4032		0	10	103	758	714	
GO:000		56/171	473/23	0.00030	0.00337	0.00230	
2683	negative regulation of immune system process	0	210	122	758	714	56
GO:005		43/171	337/23	0.00030	0.00341	0.00233	
2548	regulation of endopeptidase activity	0	210	551	913	553	43
GO:005		24/171	152/23	0.00031	0.00349	0.00238	
0709	negative regulation of protein secretion	0	210	413	531	756	24
GO:190	positive regulation of protein modification by small protein conjugation or	22/171	134/23	0.00031	0.00349	0.00238	
3322	removal	0	210	427	531	756	22
GO:004		17/171	91/232	0.00031	0.00349	0.00238	
6683	response to organophosphorus	0	10	44	531	756	17
GO:003		23/171	143/23	0.00031	0.00349	0.00238	
1333	negative regulation of protein complex assembly	0	210	583	531	756	23
GO:009		23/171	143/23	0.00031	0.00349	0.00238	
0288	negative regulation of cellular response to growth factor stimulus	0	210	583	531	756	23
GO:003		14/171	67/232	0.00031	0.00349	0.00238	
3692	cellular polysaccharide biosynthetic process	0	10	591	531	756	14
GO:004		12/171	52/232	0.00031	0.00351		
3268	positive regulation of potassium ion transport	0	10	815	352	0.0024	12
GO:003		31/171	218/23	0.00032	0.00354	0.00241	
0258	lipid modification	0	210	124	09	87	31
GO:007		16/171	83/232	0.00032	0.00354	0.00242	
1674	mononuclear cell migration	0	10	274	53	171	16
GO:004		27/171	180/23	0.00032	0.00354	0.00242	
8634	regulation of muscle organ development	0	210	285	53	171	27
GO:003	negative regulation of protein polymerization	15/171	75/232	0.00032	0.00355	0.00242	15

2272		0	10	402	148	593	
GO:003		29/171	199/23	0.00032	0.00355	0.00243	
1396	regulation of protein ubiquitination	0	210	597	943	136	29
GO:004		29/171	199/23	0.00032	0.00355	0.00243	
4242	cellular lipid catabolic process	0	210	597	943	136	29
GO:001		11/171	45/232	0.00032	0.00357	0.00243	
0257	NADH dehydrogenase complex assembly	0	10	888	206	999	11
GO:003		11/171	45/232	0.00032	0.00357	0.00243	
2981	mitochondrial respiratory chain complex I assembly	0	10	888	206	999	11
GO:004		50/171	411/23	0.00032	0.00357	0.00243	
5785	positive regulation of cell adhesion	0	210	957	206	999	50
GO:005		50/171	411/23	0.00032	0.00357	0.00243	
2547	regulation of peptidase activity	0	210	957	206	999	50
GO:000		33/171	238/23	0.00033	0.00363	0.00248	
6470	protein dephosphorylation	0	210	572	2	093	33
GO:001		30/171	209/23	0.00033	0.00365	0.00249	
7038	protein import	0	210	823	241	487	30
GO:000		25/171	162/23	0.00034	0.00366	0.00250	
2792	negative regulation of peptide secretion	0	210	109	297	209	25
GO:000		25/171	162/23	0.00034	0.00366	0.00250	
7272	ensheathment of neurons	0	210	109	297	209	25
GO:000		25/171	162/23	0.00034	0.00366	0.00250	
8366	axon ensheathment	0	210	109	297	209	25
GO:003		18/171	100/23	0.00034	0.00366	0.00250	
0593	neutrophil chemotaxis	0	210	223	846	584	18
GO:003	positive regulation of cellular amide metabolic process	23/171	144/23	0.00035	0.00374	0.00255	23

4250		0	210	014	635	904	
GO:007		13/171	60/232	0.00035	0.00376	0.00257	
1156	regulation of cell cycle arrest	0	10	265	632	268	13
GO:000		47/171	381/23	0.00035	0.00382	0.00261	
1503	ossification	0	210	859	282	127	47
GO:004		30/171	210/23	0.00036	0.00389	0.00265	
6434	organophosphate catabolic process	0	210	736	275	904	30
GO:000		38/171	289/23	0.00036	0.00389	0.00265	
6913	nucleocytoplasmic transport	0	210	892	275	904	38
GO:005		38/171	289/23	0.00036	0.00389	0.00265	
1169	nuclear transport	0	210	892	275	904	38
GO:000		35/171	259/23	0.00036	0.00389	0.00265	
1818	negative regulation of cytokine production	0	210	953	275	904	35
GO:000			32/232	0.00037	0.00389	0.00265	
6505	GPI anchor metabolic process	9/1710	10	08	275	904	9
GO:007		28/171	191/23	0.00037	0.00389	0.00265	
1804	cellular potassium ion transport	0	210	11	275	904	28
GO:007		28/171	191/23	0.00037	0.00389	0.00265	
1805	potassium ion transmembrane transport	0	210	11	275	904	28
GO:004		16/171	84/232	0.00037	0.00389	0.00265	
5913	positive regulation of carbohydrate metabolic process	0	10	182	275	904	16
GO:006		16/171	84/232	0.00037	0.00389	0.00265	
0420	regulation of heart growth	0	10	182	275	904	16
GO:190		16/171	84/232	0.00037	0.00389	0.00265	
1379	regulation of potassium ion transmembrane transport	0	10	182	275	904	16
GO:004	sarcomere organization	12/171	53/232	0.00038	0.00401	0.00274	12

5214		0	10	402	328	137	
GO:000		29/171	201/23	0.00038	0.00402	0.00274	
2685	regulation of leukocyte migration	0	210	619	312	809	29
GO:001		27/171	182/23	0.00038	0.00402	0.00274	
6331	morphogenesis of embryonic epithelium	0	210	634	312	809	27
GO:004		23/171	145/23	0.00038	0.00402	0.00275	
5216	cell-cell junction organization	0	210	767	852	178	23
GO:005		18/171	101/23	0.00038	0.00402	0.00275	
1651	maintenance of location in cell	0	210	823	852	178	18
GO:000		50/171	414/23	0.00039	0.00404	0.00276	
7265	Ras protein signal transduction	0	210	062	534	327	50
GO:004		33/171	240/23	0.00039	0.00404	0.00276	
6777	protein autophosphorylation	0	210	124	534	327	33
GO:001		43/171	341/23	0.00039	0.00405	0.00277	
9221	cytokine-mediated signaling pathway	0	210	344	67	103	43
GO:005		57/171	489/23	0.00039	0.00405	0.00277	
5074	calcium ion homeostasis	0	210	681	67	103	57
GO:004			10/232	0.00039	0.00405	0.00277	
2996	regulation of Golgi to plasma membrane protein transport	5/1710	10	717	67	103	5
GO:004			10/232	0.00039	0.00405	0.00277	
3497	regulation of protein heterodimerization activity	5/1710	10	717	67	103	5
GO:007			10/232	0.00039	0.00405	0.00277	
0391	response to lipoteichoic acid	5/1710	10	717	67	103	5
GO:007			10/232	0.00039	0.00405	0.00277	
1223	cellular response to lipoteichoic acid	5/1710	10	717	67	103	5
GO:000	heart morphogenesis	37/171	280/23	0.00039	0.00405	0.00277	37

3007		0	210	72	67	103	
GO:000			26/232	0.00040	0.00407	0.00278	
6779	porphyrin-containing compound biosynthetic process	8/1710	10	062	742	518	8
GO:190			26/232	0.00040	0.00407	0.00278	
0078	positive regulation of cellular response to insulin stimulus	8/1710	10	062	742	518	8
GO:001		10/171	39/232	0.00040	0.00409	0.00279	
0613	positive regulation of cardiac muscle hypertrophy	0	10	306	512	728	10
GO:006		31/171	221/23	0.00040	0.00414	0.00282	
1138	morphogenesis of a branching epithelium	0	210	871	103	864	31
GO:009	positive regulation of transmembrane receptor protein serine/threonine kinase	19/171	110/23	0.00040	0.00414	0.00282	
0100	signaling pathway	0	210	9	103	864	19
GO:003		25/171	164/23	0.00041	0.00416	0.00284	
2680	regulation of tumor necrosis factor production	0	210	233	751	673	25
GO:200		32/171	231/23	0.00041	0.00420	0.00286	
1234	negative regulation of apoptotic signaling pathway	0	210	641	151	995	32
GO:190		13/171	61/232	0.00041	0.00420	0.00287	
3428	positive regulation of reactive oxygen species biosynthetic process	0	10	832	621	316	13
GO:190		13/171	61/232	0.00041	0.00420	0.00287	
5207	regulation of cardiocyte differentiation	0	10	832	621	316	13
GO:005		24/171	155/23	0.00042	0.00423	0.00289	
1494	negative regulation of cytoskeleton organization	0	210	202	619	363	24
GO:003		22/171	137/23	0.00043	0.00432	0.00295	
4341	response to interferon-gamma	0	210	153	419	375	22
GO:000		14/171	69/232	0.00043	0.00433	0.00295	
6949	syncytium formation	0	10	454	205	912	14
GO:004	positive regulation of fibroblast proliferation	14/171	69/232	0.00043	0.00433	0.00295	14

8146		0	10	454	205	912	
GO:005		14/171	69/232	0.00043	0.00433	0.00295	
5117	regulation of cardiac muscle contraction	0	10	454	205	912	14
GO:003		15/171	77/232	0.00043	0.00434	0.00296	
3108	mitochondrial respiratory chain complex assembly	0	10	631	225	609	15
GO:190		18/171	102/23	0.00043	0.00436	0.00298	
5477	positive regulation of protein localization to membrane	0	210	949	448	127	18
GO:003		40/171	312/23	0.00044	0.00436	0.00298	
2103	positive regulation of response to external stimulus	0	210	159	448	127	40
GO:006			15/232	0.00044	0.00436	0.00298	
0396	growth hormone receptor signaling pathway	6/1710	10	377	448	127	6
GO:007			15/232	0.00044	0.00436	0.00298	
1378	cellular response to growth hormone stimulus	6/1710	10	377	448	127	6
GO:190			15/232	0.00044	0.00436	0.00298	
1077	regulation of relaxation of muscle	6/1710	10	377	448	127	6
GO:190			15/232	0.00044	0.00436	0.00298	
3818	positive regulation of voltage-gated potassium channel activity	6/1710	10	377	448	127	6
GO:200			15/232	0.00044	0.00436	0.00298	
0095	regulation of Wnt signaling pathway, planar cell polarity pathway	6/1710	10	377	448	127	6
GO:001		43/171	343/23	0.00044	0.00437	0.00298	
0769	regulation of cell morphogenesis involved in differentiation	0	210	536	279	694	43
GO:005		39/171	302/23	0.00044	0.00438	0.00299	
0714	positive regulation of protein secretion	0	210	751	652	632	39
GO:003		36/171	272/23	0.00045	0.00448	0.00306	
2409	regulation of transporter activity	0	210	819	147	118	36
GO:002	glial cell development	19/171	111/23	0.00045	0.00448	0.00306	19

1782		0	210	997	147	118	
GO:003		12/171	54/232	0.00046	0.00448	0.00306	
0199	collagen fibril organization	0	10	103	147	118	12
GO:005		12/171	54/232	0.00046	0.00448	0.00306	
5025	positive regulation of cardiac muscle tissue development	0	10	103	147	118	12
GO:008		12/171	54/232	0.00046	0.00448	0.00306	
6001	cardiac muscle cell action potential	0	10	103	147	118	12
GO:003		24/171	156/23	0.00046	0.00450	0.00307	
4249	negative regulation of cellular amide metabolic process	0	210	458	846	962	24
GO:001		30/171	213/23	0.00046	0.00453	0.00309	
6054	organic acid catabolic process	0	210	85	141	53	30
GO:004		30/171	213/23	0.00046	0.00453	0.00309	
6395	carboxylic acid catabolic process	0	210	85	141	53	30
GO:000		20/171	120/23	0.00047	0.00456	0.00311	
2062	chondrocyte differentiation	0	210	259	342	716	20
GO:006			33/232	0.00047	0.00459	0.00313	
1842	microtubule organizing center localization	9/1710	10	68	645	972	9
GO:001		26/171	175/23	0.00047	0.00460	0.00314	
0977	negative regulation of neuron projection development	0	210	933	676	676	26
GO:000		28/171	194/23	0.00047	0.00460	0.00314	
6006	glucose metabolic process	0	210	944	676	676	28
GO:004		11/171	47/232	0.00049	0.00470	0.00321	
5429	positive regulation of nitric oxide biosynthetic process	0	10	244	11	12	11
GO:006		13/171	62/232	0.00049	0.00470	0.00321	
0393	regulation of pathway-restricted SMAD protein phosphorylation	0	10	407	11	12	13
GO:007	protein trimerization	13/171	62/232	0.00049	0.00470	0.00321	13

0206		0	10	407	11	12	
GO:004		54/171	461/23	0.00049	0.00470	0.00321	
5666	positive regulation of neuron differentiation	0	210	476	11	12	54
GO:190		25/171	166/23	0.00049	0.00470	0.00321	
3555	regulation of tumor necrosis factor superfamily cytokine production	0	210	631	11	12	25
GO:000		18/171	103/23	0.00049	0.00470	0.00321	
2224	toll-like receptor signaling pathway	0	210	65	11	12	18
GO:000		18/171	103/23	0.00049	0.00470	0.00321	
9408	response to heat	0	210	65	11	12	18
GO:003		18/171	103/23	0.00049	0.00470	0.00321	
5601	protein deacylation	0	210	65	11	12	18
GO:009		18/171	103/23	0.00049	0.00470	0.00321	
8732	macromolecule deacylation	0	210	65	11	12	18
GO:001		27/171	185/23	0.00050	0.00474	0.00323	
0770	positive regulation of cell morphogenesis involved in differentiation	0	210	219	052	814	27
GO:001		10/171	40/232	0.00050	0.00474	0.00323	
4742	positive regulation of muscle hypertrophy	0	10	229	052	814	10
GO:004		15/171	78/232	0.00050	0.00474	0.00324	
6785	microtubule polymerization	0	10	395	847	356	15
GO:001		14/171	70/232	0.00050	0.00476	0.00325	
9915	lipid storage	0	10	687	834	714	14
GO:006		24/171	157/23	0.00051	0.00479	0.00327	
0402	calcium ion transport into cytosol	0	210	085	805	743	24
GO:003		19/171	112/23	0.00051	0.00483	0.00330	
1398	positive regulation of protein ubiquitination	0	210	634	411	206	19
GO:007	monocarboxylic acid catabolic process	19/171	112/23	0.00051	0.00483	0.00330	19

2329		0	210	634	411	206	
GO:003		26/171	176/23	0.00052	0.00489	0.00334	
5148	tube formation	0	210	379	593	429	26
GO:000		34/171	254/23	0.00052	0.00493	0.00337	
7626	locomotory behavior	0	210	894	622	181	34
GO:000			27/232	0.00053	0.00494	0.00337	
9394	2'-deoxyribonucleotide metabolic process	8/1710	10	28	067	485	8
GO:001			27/232	0.00053	0.00494	0.00337	
9692	deoxyribose phosphate metabolic process	8/1710	10	28	067	485	8
GO:190			27/232	0.00053	0.00494	0.00337	
1018	positive regulation of potassium ion transmembrane transporter activity	8/1710	10	28	067	485	8
GO:200			27/232	0.00053	0.00494	0.00337	
0727	positive regulation of cardiac muscle cell differentiation	8/1710	10	28	067	485	8
GO:000			21/232	0.00053	0.00497	0.00339	
6783	heme biosynthetic process	7/1710	10	855	03	509	7
GO:000			21/232	0.00053	0.00497	0.00339	
8053	mitochondrial fusion	7/1710	10	855	03	509	7
GO:004			21/232	0.00053	0.00497	0.00339	
6716	muscle cell cellular homeostasis	7/1710	10	855	03	509	7
GO:003		25/171	167/23	0.00054	0.00500	0.00342	
2640	tumor necrosis factor production	0	210	365	95	186	25
GO:000		12/171	55/232	0.00055	0.00505	0.00344	
7528	neuromuscular junction development	0	10	066	02	966	12
GO:001		12/171	55/232	0.00055	0.00505	0.00344	
0665	regulation of cardiac muscle cell apoptotic process	0	10	066	02	966	12
GO:001	negative regulation of mitochondrion organization	12/171	55/232	0.00055	0.00505	0.00344	12

0823		0	10	066	02	966	
GO:000		51/171	431/23	0.00055	0.00507	0.00346	
1558	regulation of cell growth	0	210	382	122	403	51
GO:005		41/171	326/23	0.00055	0.00511	0.00349	
0890	cognition	0	210	931	34	284	41
GO:003		13/171	63/232	0.00058	0.00530	0.00362	
0330	DNA damage response, signal transduction by p53 class mediator	0	10	113	459	343	13
GO:001		14/171	71/232	0.00058	0.00536	0.00366	
0611	regulation of cardiac muscle hypertrophy	0	10	919	89	737	14
GO:009		21/171	131/23	0.00059	0.00536	0.00366	
0316	positive regulation of intracellular protein transport	0	210	001	89	737	21
GO:200		25/171	168/23	0.00059	0.00539	0.00368	
1257	regulation of cation channel activity	0	210	49	72	67	25
GO:000		11/171	48/232	0.00059	0.00539	0.00368	
7520	myoblast fusion	0	10	681	72	67	11
GO:009		11/171	48/232	0.00059	0.00539	0.00368	
0659	walking behavior	0	10	681	72	67	11
GO:190		11/171	48/232	0.00059	0.00539	0.00368	
4407	positive regulation of nitric oxide metabolic process	0	10	681	72	67	11
GO:000			34/232	0.00060	0.00547	0.00374	
2931	response to ischemia	9/1710	10	645	583	04	9
GO:003		33/171	246/23	0.00060	0.00549	0.00375	
1334	positive regulation of protein complex assembly	0	210	973	701	487	33
GO:003		48/171	401/23	0.00061	0.00553	0.00377	
0099	myeloid cell differentiation	0	210	559	282	933	48
GO:004	myelination	24/171	159/23	0.00061	0.00553	0.00377	24

2552		0	210	56	282	933	
GO:001		53/171	455/23	0.00063	0.00567	0.00387	
5672	monovalent inorganic cation transport	0	210	257	662	756	53
GO:200		19/171	114/23	0.00064	0.00579	0.00396	
0045	regulation of G1/S transition of mitotic cell cycle	0	210	723	927	134	19
GO:003		32/171	237/23	0.00065	0.00584	0.00399	
4976	response to endoplasmic reticulum stress	0	210	348	632	347	32
GO:190		12/171	56/232	0.00065	0.00584	0.00399	
5517	macrophage migration	0	10	448	632	347	12
GO:006			16/232	0.00066	0.00593	0.00405	
0766	negative regulation of androgen receptor signaling pathway	6/1710	10	591	935	702	6
GO:001		17/171	97/232	0.00067	0.00604	0.00412	
0595	positive regulation of endothelial cell migration	0	10	966	346	814	17
GO:003		17/171	97/232	0.00067	0.00604	0.00412	
0510	regulation of BMP signaling pathway	0	10	966	346	814	17
GO:005		13/171	64/232	0.00068	0.00604	0.00412	
1926	negative regulation of calcium ion transport	0	10	078	422	865	13
GO:000			11/232	0.00068	0.00604	0.00412	
9265	2'-deoxyribonucleotide biosynthetic process	5/1710	10	397	503	921	5
GO:004			11/232	0.00068	0.00604	0.00412	
6385	deoxyribose phosphate biosynthetic process	5/1710	10	397	503	921	5
GO:009			11/232	0.00068	0.00604	0.00412	
0281	negative regulation of calcium ion import	5/1710	10	397	503	921	5
GO:005			28/232	0.00069	0.00616	0.00420	
1194	positive regulation of cofactor metabolic process	8/1710	10	814	093	838	8
GO:007	tumor necrosis factor superfamily cytokine production	25/171	170/23	0.00071	0.00625	0.00427	25

1706		0	210	018	775	452	
GO:000		21/171	133/23	0.00072	0.00636	0.00434	
1837	epithelial to mesenchymal transition	0	210	358	617	858	21
GO:000		16/171	89/232	0.00072	0.00637	0.00435	
7229	integrin-mediated signaling pathway	0	10	525	131	209	16
GO:004		37/171	289/23	0.00072	0.00637	0.00435	
5088	regulation of innate immune response	0	210	711	806	669	37
GO:001			22/232	0.00073	0.00646	0.00441	
4850	response to muscle activity	7/1710	10	995	578	662	7
GO:003			22/232	0.00073	0.00646	0.00441	
4405	response to fluid shear stress	7/1710	10	995	578	662	7
GO:001		44/171	362/23	0.00074	0.00646	0.00441	
0976	positive regulation of neuron projection development	0	210	043	578	662	44
GO:000		41/171	331/23	0.00075	0.00661	0.00452	
2793	positive regulation of peptide secretion	0	210	899	796	057	41
GO:001		10/171	42/232	0.00076	0.00662	0.00452	
0559	regulation of glycoprotein biosynthetic process	0	10	179	799	741	10
GO:004			35/232	0.00076	0.00662	0.00452	
5940	positive regulation of steroid metabolic process	9/1710	10	355	799	741	9
GO:007			35/232	0.00076	0.00662	0.00452	
0873	regulation of glycogen metabolic process	9/1710	10	355	799	741	9
GO:003		17/171	98/232	0.00076	0.00664	0.00454	
2760	positive regulation of tumor necrosis factor production	0	10	685	678	025	17
GO:001		12/171	57/232	0.00077	0.00669	0.00457	
6101	diterpenoid metabolic process	0	10	421	07	025	12
GO:004	positive regulation of organ growth	12/171	57/232	0.00077	0.00669	0.00457	12

6622		0	10	421	07	025	
GO:000		54/171	470/23	0.00078	0.00673	0.00460	
7409	axonogenesis	0	210	103	973	374	54
GO:003		28/171	200/23	0.00078	0.00674	0.00460	
2388	positive regulation of intracellular transport	0	210	229	065	437	28
GO:007		36/171	280/23	0.00078	0.00677	0.00462	
0588	calcium ion transmembrane transport	0	210	737	436	74	36
GO:001		18/171	107/23	0.00079	0.00681	0.00465	
4074	response to purine-containing compound	0	210	298	264	355	18
GO:005		21/171	134/23	0.00079	0.00684	0.00467	
1928	positive regulation of calcium ion transport	0	210	964	972	888	21
GO:005		21/171	134/23	0.00079	0.00684	0.00467	
5123	digestive system development	0	210	964	972	888	21
GO:004		34/171	260/23	0.00080	0.00688	0.00470	
5665	negative regulation of neuron differentiation	0	210	46	212	101	34
GO:001		24/171	162/23	0.00080	0.00689	0.00471	
0952	positive regulation of peptidase activity	0	210	772	871	234	24
GO:000		32/171	240/23	0.00081	0.00692	0.00472	
1763	morphogenesis of a branching structure	0	210	149	074	739	32
GO:005		47/171	396/23	0.00083	0.00712	0.00486	
1090	regulation of DNA-binding transcription factor activity	0	210	71	201	487	47
GO:003		23/171	153/23	0.00083	0.00712	0.00486	
2675	regulation of interleukin-6 production	0	210	752	201	487	23
GO:190		17/171	99/232	0.00086	0.00733	0.00500	
3557	positive regulation of tumor necrosis factor superfamily cytokine production	0	10	342	154	799	17
GO:000	learning or memory	37/171	292/23	0.00088	0.00747	0.00510	37

7611		0	210	144	098	324	
GO:004		24/171	163/23	0.00088	0.00747	0.00510	
8660	regulation of smooth muscle cell proliferation	0	210	24	098	324	24
GO:190		18/171	108/23	0.00088	0.00749	0.00511	
0006	positive regulation of dendrite development	0	210	726	044	654	18
GO:190		18/171	108/23	0.00088	0.00749	0.00511	
4659	glucose transmembrane transport	0	210	726	044	654	18
GO:005		19/171	117/23	0.00089		0.00516	
1701	interaction with host	0	210	68	0.00756	405	19
GO:000			29/232	0.00090	0.00759	0.00518	
2092	positive regulation of receptor internalization	8/1710	10	241	634	888	8
GO:000		54/171	473/23	0.00090	0.00760	0.00519	
6874	cellular calcium ion homeostasis	0	210	496	687	606	54
GO:001		14/171	74/232	0.00090	0.00761	0.00520	
4743	regulation of muscle hypertrophy	0	10	724	508	167	14
GO:006		12/171	58/232	0.00091	0.00764	0.00521	
1337	cardiac conduction	0	10	168	136	963	12
GO:000		23/171	154/23	0.00091	0.00767	0.00524	
1838	embryonic epithelial tube formation	0	210	734	78	452	23
GO:002		34/171	262/23	0.00092	0.00769	0.00525	
2898	regulation of transmembrane transporter activity	0	210	113	847	864	34
GO:004		10/171	43/232	0.00092	0.00773	0.00528	
0018	positive regulation of multicellular organism growth	0	10	791	3	222	10
GO:190		10/171	43/232	0.00092	0.00773	0.00528	
1381	positive regulation of potassium ion transmembrane transport	0	10	791	3	222	10
GO:000	receptor-mediated endocytosis	30/171	222/23	0.00093	0.00777	0.00530	30

6898		0	210	381	108	823	
GO:004		52/171	452/23	0.00093	0.00779	0.00532	
5786	negative regulation of cell cycle	0	210	851	914	74	52
GO:003			36/232	0.00095	0.00789	0.00539	
4205	amyloid-beta formation	9/1710	10	225	08	001	9
GO:190			36/232	0.00095	0.00789	0.00539	
5209	positive regulation of cardiocyte differentiation	9/1710	10	225	08	001	9
GO:000		24/171	164/23	0.00096	0.00796	0.00544	
2218	activation of innate immune response	0	210	298	506	074	24
GO:004			17/232	0.00096	0.00796	0.00544	
5989	positive regulation of striated muscle contraction	6/1710	10	53	506	074	6
GO:200			17/232	0.00096	0.00796	0.00544	
1170	negative regulation of ATP biosynthetic process	6/1710	10	53	506	074	6
GO:000		21/171	136/23	0.00097	0.00800	0.00546	
9791	post-embryonic development	0	210	267	326	683	21
GO:009		21/171	136/23	0.00097	0.00800	0.00546	
7553	calcium ion transmembrane import into cytosol	0	210	267	326	683	21
GO:000		18/171	109/23	0.00099	0.00814	0.00556	
8645	hexose transmembrane transport	0	210	096	233	183	18
GO:004		19/171	118/23	0.00099	0.00816	0.00557	
6620	regulation of organ growth	0	210	651	525	748	19
GO:190			23/232	0.00099	0.00816	0.00557	
3077	negative regulation of protein localization to plasma membrane	7/1710	10	655	525	748	7
GO:004		45/171	378/23	0.00100	0.00819	0.00559	
2176	regulation of protein catabolic process	0	210	169	591	842	45
GO:005	import into nucleus	23/171	155/23	0.00100	0.00819	0.00559	23

1170		0	210	365	702	918	
GO:001		30/171	223/23	0.00100	0.00819	0.00559	
9318	hexose metabolic process	0	210	463	702	918	30
GO:003		11/171	51/232	0.00102	0.00834	0.00570	
1103	axon regeneration	0	10	598	791	225	11
GO:003		11/171	51/232	0.00102	0.00834	0.00570	
2355	response to estradiol	0	10	598	791	225	11
GO:003		14/171	75/232	0.00104	0.00845	0.00577	
2507	maintenance of protein location in cell	0	10	109	906	818	14
GO:003		16/171	92/232	0.00105	0.00853	0.00582	
0838	positive regulation of actin filament polymerization	0	10	157	235	824	16
GO:003		33/171	254/23	0.00106	0.00862	0.00588	
2412	regulation of ion transmembrane transporter activity	0	210	458	114	889	33
GO:001		12/171	59/232	0.00106	0.00862	0.00588	
0659	cardiac muscle cell apoptotic process	0	10	888	114	889	12
GO:000		13/171	67/232	0.00106	0.00862	0.00588	
0768	syncytium formation by plasma membrane fusion	0	10	99	114	889	13
GO:004		13/171	67/232	0.00106	0.00862	0.00588	
8678	response to axon injury	0	10	99	114	889	13
GO:014		13/171	67/232	0.00106	0.00862	0.00588	
0253	cell-cell fusion	0	10	99	114	889	13
GO:005		17/171	101/23	0.00108	0.00875	0.00597	
5024	regulation of cardiac muscle tissue development	0	210	794	449	998	17
GO:190		20/171	128/23	0.00109	0.00878	0.00600	
2806	regulation of cell cycle G1/S phase transition	0	210	325	506	086	20
GO:000	steroid biosynthetic process	23/171	156/23	0.00109	0.00880	0.00601	23

6694		0	210	687	206	247	
GO:000			12/232	0.00110	0.00881	0.00602	
9219	pyrimidine deoxyribonucleotide metabolic process	5/1710	10	154	537	156	5
GO:003			12/232	0.00110	0.00881	0.00602	
2048	cardiolipin metabolic process	5/1710	10	154	537	156	5
GO:199		19/171	119/23	0.00110	0.00883	0.00603	
0266	neutrophil migration	0	210	556	54	525	19
GO:000		42/171	348/23	0.00110	0.00884	0.00604	
6644	phospholipid metabolic process	0	210	824	472	161	42
GO:004		34/171	265/23	0.00112	0.00895	0.00611	
2593	glucose homeostasis	0	210	363	534	717	34
GO:000		27/171	195/23	0.00113	0.00904	0.00618	
7266	Rho protein signal transduction	0	210	689	868	093	27
GO:000		22/171	147/23	0.00113	0.00905	0.00618	
8643	carbohydrate transport	0	210	983	967	844	22
GO:003		28/171	205/23	0.00115	0.00911	0.00622	
1345	negative regulation of cell projection organization	0	210	088	842	857	28
GO:005			30/232	0.00115	0.00911	0.00622	
1085	chaperone cofactor-dependent protein refolding	8/1710	10	19	842	857	8
GO:006			30/232	0.00115	0.00911	0.00622	
0143	positive regulation of syncytium formation by plasma membrane fusion	8/1710	10	19	842	857	8
GO:003		44/171	370/23	0.00115	0.00914	0.00624	
1349	positive regulation of defense response	0	210	702	653	777	44
GO:004		42/171	349/23	0.00117	0.00925	0.00632	
5861	negative regulation of proteolysis	0	210	233	51	193	42
GO:003	negative regulation of intracellular steroid hormone receptor signaling pathway	9/1710	37/232	0.00117	0.00927	0.00633	9

3144			10	705	98	88	
GO:004		16/171	93/232	0.00118	0.00930	0.00635	
5445	myoblast differentiation	0	10	482	342	494	16
GO:004		16/171	93/232	0.00118	0.00930	0.00635	
6889	positive regulation of lipid biosynthetic process	0	10	482	342	494	16
GO:005	modification of morphology or physiology of other organism involved in symbiotic	16/171	93/232	0.00118	0.00930	0.00635	
1817	interaction	0	10	482	342	494	16
GO:000		14/171	76/232	0.00119	0.00932	0.00637	
7045	cell-substrate adherens junction assembly	0	10	114	795	17	14
GO:004		14/171	76/232	0.00119	0.00932	0.00637	
8041	focal adhesion assembly	0	10	114	795	17	14
GO:003		34/171	266/23	0.00119	0.00937	0.00640	
3500	carbohydrate homeostasis	0	210	928	909	662	34
GO:004		20/171	129/23	0.00120	0.00941	0.00643	
6488	phosphatidylinositol metabolic process	0	210	607	958	428	20
GO:190		11/171	52/232	0.00121	0.00947	0.00647	
1570	fatty acid derivative biosynthetic process	0	10	589	191	003	11
GO:000		33/171	256/23	0.00121	0.00947	0.00647	
6605	protein targeting	0	210	662	191	003	33
GO:001		17/171	102/23	0.00121	0.00947	0.00647	
9218	regulation of steroid metabolic process	0	210	763	191	003	17
GO:003		44/171	371/23	0.00122	0.00948	0.00648	
1667	response to nutrient levels	0	210	142	876	154	44
GO:003		19/171	120/23	0.00122	0.00950	0.00648	
1109	microtubule polymerization or depolymerization	0	210	463	107	995	19
GO:000	regulation of leukocyte chemotaxis	18/171	111/23	0.00122	0.00950	0.00649	18

2688		0	210	963	202	059	
GO:001		18/171	111/23	0.00122	0.00950	0.00649	
5749	monosaccharide transmembrane transport	0	210	963	202	059	18
GO:004		18/171	111/23	0.00122	0.00950	0.00649	
3401	steroid hormone mediated signaling pathway	0	210	963	202	059	18
GO:000		29/171	216/23	0.00124	0.00958	0.00654	
2573	myeloid leukocyte differentiation	0	210	614	014	396	29
GO:003		12/171	60/232	0.00124	0.00958	0.00654	
0837	negative regulation of actin filament polymerization	0	10	794	014	396	12
GO:003		12/171	60/232	0.00124	0.00958	0.00654	
1102	neuron projection regeneration	0	10	794	014	396	12
GO:004		12/171	60/232	0.00124	0.00958	0.00654	
2982	amyloid precursor protein metabolic process	0	10	794	014	396	12
GO:200		12/171	60/232	0.00124	0.00958	0.00654	
0134	negative regulation of G1/S transition of mitotic cell cycle	0	10	794	014	396	12
GO:003		15/171	85/232	0.00127	0.00979	0.00668	
4333	adherens junction assembly	0	10	724	219	881	15
GO:005		45/171	383/23	0.00130	0.01002	0.00684	
1260	protein homooligomerization	0	210	942	574	834	45
GO:005		10/171	45/232	0.00134	0.01026	0.00701	
1646	mitochondrion localization	0	10	935	638	271	10
GO:006		10/171	45/232	0.00134	0.01026	0.00701	
0421	positive regulation of heart growth	0	10	935	638	271	10
GO:004		24/171	168/23	0.00135	0.01026	0.00701	
8659	smooth muscle cell proliferation	0	210	226	638	271	24
GO:190	regulation of protein localization to nucleus	19/171	121/23	0.00135	0.01026	0.00701	19

0180		0	210	446	638	271	
GO:000			18/232	0.00135	0.01026	0.00701	
5980	glycogen catabolic process	6/1710	10	826	638	271	6
GO:000			18/232	0.00135	0.01026	0.00701	
9251	glucan catabolic process	6/1710	10	826	638	271	6
GO:003			18/232	0.00135	0.01026	0.00701	
0809	negative regulation of nucleotide biosynthetic process	6/1710	10	826	638	271	6
GO:190			18/232	0.00135	0.01026	0.00701	
0372	negative regulation of purine nucleotide biosynthetic process	6/1710	10	826	638	271	6
GO:190			18/232	0.00135	0.01026	0.00701	
3909	regulation of receptor clustering	6/1710	10	826	638	271	6
GO:005		14/171	77/232	0.00135	0.01026	0.00701	
5021	regulation of cardiac muscle tissue growth	0	10	888	638	271	14
GO:004		17/171	103/23	0.00136	0.01026	0.00701	
3255	regulation of carbohydrate biosynthetic process	0	210	018	638	271	17
GO:001		18/171	112/23	0.00136	0.01028	0.00702	
6525	negative regulation of angiogenesis	0	210	622	54	57	18
GO:003		18/171	112/23	0.00136	0.01028	0.00702	
4219	carbohydrate transmembrane transport	0	210	622	54	57	18
GO:006		13/171	69/232	0.00142	0.01068	0.00729	
1035	regulation of cartilage development	0	10	087	307	734	13
GO:003		11/171	53/232	0.00143	0.01076	0.00735	
0850	prostate gland development	0	10	376	611	407	11
GO:001			38/232	0.00144	0.01078	0.00736	
0831	positive regulation of myotube differentiation	9/1710	10	282	506	701	9
GO:001	astrocyte development	9/1710	38/232	0.00144	0.01078	0.00736	9

4002			10	282	506	701	
GO:004			38/232	0.00144	0.01078	0.00736	
2149	cellular response to glucose starvation	9/1710	10	282	506	701	9
GO:001		15/171	86/232	0.00144	0.01078	0.00736	
0827	regulation of glucose transmembrane transport	0	10	367	506	701	15
GO:005		12/171	61/232	0.00145	0.01080	0.00737	
1591	response to cAMP	0	10	112	271	906	12
GO:001			31/232	0.00145	0.01080	0.00737	
9884	antigen processing and presentation of exogenous antigen	8/1710	10	343	271	906	8
GO:004			31/232	0.00145	0.01080	0.00737	
5662	negative regulation of myoblast differentiation	8/1710	10	343	271	906	8
GO:007			31/232	0.00145	0.01080	0.00737	
0670	response to interleukin-4	8/1710	10	343	271	906	8
GO:001		33/171	259/23	0.00148	0.01098	0.00750	
6050	vesicle organization	0	210	009	691	489	33
GO:001		22/171	150/23	0.00148	0.01103	0.00753	
4013	regulation of gliogenesis	0	210	98	092	495	22
GO:002		22/171	150/23	0.00148	0.01103	0.00753	
2612	gland morphogenesis	0	210	98	092	495	22
GO:003		17/171	104/23	0.00151	0.01120	0.00765	
0518	intracellular steroid hormone receptor signaling pathway	0	210	658	083	101	17
GO:003		17/171	104/23	0.00151	0.01120	0.00765	
2612	interleukin-1 production	0	210	658	083	101	17
GO:190		31/171	239/23	0.00152	0.01122	0.00766	
3362	regulation of cellular protein catabolic process	0	210	206	714	898	31
GO:003	cellular carbohydrate biosynthetic process	14/171	78/232	0.00154	0.01135	0.00775	14

4637		0	10	59	994	97	
GO:190		14/171	78/232	0.00154	0.01135	0.00775	
0046	regulation of hemostasis	0	10	59	994	97	14
GO:190		14/171	78/232	0.00154	0.01135	0.00775	
1862	negative regulation of muscle tissue development	0	10	59	994	97	14
GO:190		23/171	160/23	0.00154	0.01136	0.00776	
3169	regulation of calcium ion transmembrane transport	0	210	817	235	134	23
GO:004		36/171	291/23	0.00157	0.01151	0.00786	
8511	rhythmic process	0	210	145	872	815	36
GO:000		10/171	46/232	0.00161	0.01178	0.00805	
7628	adult walking behavior	0	10	202	647	105	10
GO:003		10/171	46/232	0.00161	0.01178	0.00805	
3013	tetrapyrrole metabolic process	0	10	202	647	105	10
GO:000		22/171	151/23	0.00162	0.01186	0.00810	
6606	protein import into nucleus	0	210	522	814	684	22
GO:007		55/171	497/23	0.00166	0.01212	0.00828	
2503	cellular divalent inorganic cation homeostasis	0	210	228	363	135	55
GO:006		18/171	114/23	0.00167	0.01221	0.00834	
0419	heart growth	0	210	825	453	344	18
GO:001		12/171	62/232	0.00168	0.01221	0.00834	
4911	positive regulation of smooth muscle cell migration	0	10	081	453	344	12
GO:004			13/232	0.00168	0.01221	0.00834	
6471	phosphatidylglycerol metabolic process	5/1710	10	187	453	344	5
GO:007		23/171	161/23	0.00168	0.01221	0.00834	
2175	epithelial tube formation	0	210	31	453	344	23
GO:004	regulation of potassium ion transport	17/171	105/23	0.00168	0.01223	0.00835	17

3266		0	210	787	393	67	
GO:005		21/171	142/23	0.00169	0.01228	0.00839	
0680	negative regulation of epithelial cell proliferation	0	210	746	818	375	21
GO:004			25/232	0.00171	0.01238	0.00846	
3171	peptide catabolic process	7/1710	10	558	866	239	7
GO:190			25/232	0.00171	0.01238	0.00846	
3747	regulation of establishment of protein localization to mitochondrion	7/1710	10	558	866	239	7
GO:003		14/171	79/232	0.00175	0.01264	0.00863	
3143	regulation of intracellular steroid hormone receptor signaling pathway	0	10	387	021	422	14
GO:003			39/232	0.00175	0.01264	0.00863	
0521	androgen receptor signaling pathway	9/1710	10	474	021	422	9
GO:006		30/171	231/23	0.00176	0.01266	0.00865	
0348	bone development	0	210	044	566	16	30
GO:002		26/171	191/23	0.00177	0.01276	0.00871	
1915	neural tube development	0	210	626	376	861	26
GO:004		27/171	201/23	0.00178	0.01280	0.00875	
3281	regulation of cysteine-type endopeptidase activity involved in apoptotic process	0	210	487	981	007	27
GO:000			32/232	0.00181	0.01297	0.00886	
6220	pyrimidine nucleotide metabolic process	8/1710	10	431	328	173	8
GO:000			32/232	0.00181	0.01297	0.00886	
9262	deoxyribonucleotide metabolic process	8/1710	10	431	328	173	8
GO:005			32/232	0.00181	0.01297	0.00886	
1642	centrosome localization	8/1710	10	431	328	173	8
GO:000		23/171	162/23	0.00182	0.01303	0.00890	
7033	vacuole organization	0	210	795	894	658	23
GO:003	interleukin-6 production	23/171	162/23	0.00182	0.01303	0.00890	23

2635		0	210	795	894	658	
GO:190		15/171	88/232	0.00183	0.01304	0.00891	
3035	negative regulation of response to wounding	0	10	134	715	219	15
GO:005		18/171	115/23	0.00185	0.01314	0.00897	
1209	release of sequestered calcium ion into cytosol	0	210	555	017	573	18
GO:007		18/171	115/23	0.00185	0.01314	0.00897	
1346	cellular response to interferon-gamma	0	210	555	017	573	18
GO:000			19/232	0.00186	0.01314	0.00897	
2495	antigen processing and presentation of peptide antigen via MHC class II	6/1710	10	239	017	573	6
GO:003			19/232	0.00186	0.01314	0.00897	
2011	ARF protein signal transduction	6/1710	10	239	017	573	6
GO:003			19/232	0.00186	0.01314	0.00897	
2012	regulation of ARF protein signal transduction	6/1710	10	239	017	573	6
GO:004			19/232	0.00186	0.01314	0.00897	
4766	multi-organism transport	6/1710	10	239	017	573	6
GO:190			19/232	0.00186	0.01314	0.00897	
2259	regulation of delayed rectifier potassium channel activity	6/1710	10	239	017	573	6
GO:190			19/232	0.00186	0.01314	0.00897	
2579	multi-organism localization	6/1710	10	239	017	573	6
GO:200		16/171	97/232	0.00186	0.01317	0.00899	
1243	negative regulation of intrinsic apoptotic signaling pathway	0	10	93	306	82	16
GO:004		17/171	106/23	0.00187	0.01318	0.00900	
5446	endothelial cell differentiation	0	210	515	247	462	17
GO:004		17/171	106/23	0.00187	0.01318	0.00900	
8661	positive regulation of smooth muscle cell proliferation	0	210	515	247	462	17
GO:190	regulation of glycoprotein metabolic process	10/171	47/232	0.00191	0.01344	0.00918	10

3018		0	10	46	365	302	
GO:006		12/171	63/232	0.00193	0.01360	0.00929	
0999	positive regulation of dendritic spine development	0	10	956	25	154	12
GO:000		11/171	55/232	0.00196	0.01373	0.00938	
1523	retinoid metabolic process	0	10	558	557	243	11
GO:000		11/171	55/232	0.00196	0.01373	0.00938	
2548	monocyte chemotaxis	0	10	558	557	243	11
GO:004		11/171	55/232	0.00196	0.01373	0.00938	
6676	negative regulation of insulin secretion	0	10	558	557	243	11
GO:005		46/171	402/23	0.00198	0.01388	0.00948	
1346	negative regulation of hydrolase activity	0	210	903	284	303	46
GO:000		19/171	125/23	0.00199	0.01392	0.00951	
2761	regulation of myeloid leukocyte differentiation	0	210	715	287	037	19
GO:003		30/171	233/23	0.00201	0.01401	0.00957	
0278	regulation of ossification	0	210	253	334	217	30
GO:000		21/171	144/23	0.00202	0.01407	0.00961	
2687	positive regulation of leukocyte migration	0	210	39	572	478	21
GO:200		18/171	116/23	0.00204	0.01422	0.00971	
0181	negative regulation of blood vessel morphogenesis	0	210	837	902	949	18
GO:001		15/171	89/232	0.00205	0.01426	0.00974	
4015	positive regulation of gliogenesis	0	10	554	185	192	15
GO:004		17/171	107/23	0.00207	0.01441	0.00984	
3487	regulation of RNA stability	0	210	956	084	369	17
GO:004		16/171	98/232	0.00208	0.01441	0.00984	
3488	regulation of mRNA stability	0	10	442	084	369	16
GO:200	negative regulation of extrinsic apoptotic signaling pathway	16/171	98/232	0.00208	0.01441	0.00984	16

1237		0	10	442	084	369	
GO:002		38/171	317/23	0.00209	0.01445	0.00987	
1700	developmental maturation	0	210	457	785	58	38
GO:000		22/171	154/23	0.00209	0.01445	0.00987	
6937	regulation of muscle contraction	0	210	617	785	58	22
GO:007		20/171	135/23	0.00211	0.01449	0.00989	
2331	signal transduction by p53 class mediator	0	210	35	116	856	20
GO:190		31/171	244/23	0.00211	0.01449	0.00989	
1215	negative regulation of neuron death	0	210	462	116	856	31
GO:000			40/232	0.00211	0.01449	0.00989	
6692	prostanoid metabolic process	9/1710	10	836	116	856	9
GO:000			40/232	0.00211	0.01449	0.00989	
6693	prostaglandin metabolic process	9/1710	10	836	116	856	9
GO:003			40/232	0.00211	0.01449	0.00989	
0225	macrophage differentiation	9/1710	10	836	116	856	9
GO:003			40/232	0.00211	0.01449	0.00989	
8179	neurotrophin signaling pathway	9/1710	10	836	116	856	9
GO:007			40/232	0.00211	0.01449	0.00989	
0207	protein homotrimerization	9/1710	10	836	116	856	9
GO:000		30/171	234/23	0.00214	0.01468	0.01003	
6813	potassium ion transport	0	210	981	908	375	30
GO:009			26/232	0.00220	0.01499	0.01024	
8901	regulation of cardiac muscle cell action potential	7/1710	10	004	724	424	7
GO:200			26/232	0.00220	0.01499	0.01024	
0050	regulation of non-canonical Wnt signaling pathway	7/1710	10	004	724	424	7
GO:004	branching morphogenesis of an epithelial tube	25/171	184/23	0.00220	0.01503	0.01027	25

8754		0	210	815	5	004	
GO:000		27/171	204/23	0.00221	0.01505	0.01028	
1666	response to hypoxia	0	210	358	439	328	27
GO:000		12/171	64/232	0.00223	0.01509	0.01031	
2763	positive regulation of myeloid leukocyte differentiation	0	10	004	608	176	12
GO:003	negative regulation of transforming growth factor beta receptor signaling	12/171	64/232	0.00223	0.01509	0.01031	
0512	pathway	0	10	004	608	176	12
GO:003		12/171	64/232	0.00223	0.01509	0.01031	
4605	cellular response to heat	0	10	004	608	176	12
GO:190		12/171	64/232	0.00223	0.01509	0.01031	
2807	negative regulation of cell cycle G1/S phase transition	0	10	004	608	176	12
GO:007		14/171	81/232	0.00223	0.01512	0.01033	
2384	organelle transport along microtubule	0	10	979	652	255	14
GO:001			33/232	0.00224	0.01512	0.01033	
0543	regulation of platelet activation	8/1710	10	231	652	255	8
GO:001			33/232	0.00224	0.01512	0.01033	
0667	negative regulation of cardiac muscle cell apoptotic process	8/1710	10	231	652	255	8
GO:005		18/171	117/23	0.00225	0.01520	0.01038	
1283	negative regulation of sequestering of calcium ion	0	210	775	258	451	18
GO:003		10/171	48/232	0.00226	0.01520	0.01038	
1648	protein destabilization	0	10	139	258	451	10
GO:200		10/171	48/232	0.00226	0.01520	0.01038	
0725	regulation of cardiac muscle cell differentiation	0	10	139	258	451	10
GO:004		11/171	56/232	0.00228	0.01535	0.01048	
3536	positive regulation of blood vessel endothelial cell migration	0	10	607	085	579	11
GO:004	regulation of multicellular organism growth	15/171	90/232	0.00230	0.01542	0.01053	15

0014		0	10	21	434	599	
GO:003		17/171	108/23	0.00230	0.01542	0.01053	
0316	osteoclast differentiation	0	210	23	434	599	17
GO:003		16/171	99/232	0.00231	0.01550	0.01059	
2755	positive regulation of interleukin-6 production	0	10	979	596	174	16
GO:006		16/171	99/232	0.00231	0.01550	0.01059	
2014	negative regulation of small molecule metabolic process	0	10	979	596	174	16
GO:000		28/171	215/23	0.00235	0.01573	0.01074	
2274	myeloid leukocyte activation	0	210	652	345	713	28
GO:004		24/171	175/23	0.00236	0.01574	0.01075	
5807	positive regulation of endocytosis	0	210	118	656	609	24
GO:009		31/171	246/23	0.00240	0.01600	0.01092	
7485	neuron projection guidance	0	210	201	06	962	31
GO:000		13/171	73/232	0.00241	0.01602	0.01094	
6626	protein targeting to mitochondrion	0	10	154	752	801	13
GO:005		13/171	73/232	0.00241	0.01602	0.01094	
1145	smooth muscle cell differentiation	0	10	154	752	801	13
GO:000			14/232	0.00245	0.01628	0.01112	
9263	deoxyribonucleotide biosynthetic process	5/1710	10	85	405	323	5
GO:001	antigen processing and presentation of exogenous peptide antigen via MHC class		14/232	0.00245	0.01628	0.01112	
9886	II	5/1710	10	85	405	323	5
GO:200			14/232	0.00245	0.01628	0.01112	
1044	regulation of integrin-mediated signaling pathway	5/1710	10	85	405	323	5
GO:200		29/171	226/23	0.00248	0.01634	0.01116	
0116	regulation of cysteine-type endopeptidase activity	0	210	9	9	76	29
GO:000	antigen processing and presentation of peptide or polysaccharide antigen via	6/1710	20/232	0.00249	0.01634	0.01116	6

2504	MHC class II		10	629	9	76	
GO:000			20/232	0.00249	0.01634	0.01116	
8090	retrograde axonal transport	6/1710	10	629	9	76	6
GO:000			20/232	0.00249	0.01634	0.01116	
9200	deoxyribonucleoside triphosphate metabolic process	6/1710	10	629	9	76	6
GO:001			20/232	0.00249	0.01634	0.01116	
6226	iron-sulfur cluster assembly	6/1710	10	629	9	76	6
GO:003			20/232	0.00249	0.01634	0.01116	
1163	metallo-sulfur cluster assembly	6/1710	10	629	9	76	6
GO:003			20/232	0.00249	0.01634	0.01116	
2288	myelin assembly	6/1710	10	629	9	76	6
GO:004			20/232	0.00249	0.01634	0.01116	
4247	cellular polysaccharide catabolic process	6/1710	10	629	9	76	6
GO:006			20/232	0.00249	0.01634	0.01116	
1323	cell proliferation involved in heart morphogenesis	6/1710	10	629	9	76	6
GO:200			20/232	0.00249	0.01634	0.01116	
0136	regulation of cell proliferation involved in heart morphogenesis	6/1710	10	629	9	76	6
GO:004		23/171	166/23	0.00251	0.01647	0.01125	
5931	positive regulation of mitotic cell cycle	0	210	838	524	383	23
GO:003			41/232	0.00253	0.01653	0.01129	
8083	peptidyl-tyrosine autophosphorylation	9/1710	10	951	937	764	9
GO:005			41/232	0.00253	0.01653	0.01129	
1281	positive regulation of release of sequestered calcium ion into cytosol	9/1710	10	951	937	764	9
GO:008			41/232	0.00253	0.01653	0.01129	
6009	membrane repolarization	9/1710	10	951	937	764	9
GO:190	positive regulation of sprouting angiogenesis	9/1710	41/232	0.00253	0.01653	0.01129	9

3672			10	951	937	764	
GO:006		12/171	65/232	0.00255	0.01662	0.01135	
0425	lung morphogenesis	0	10	509	23	428	12
GO:003		15/171	91/232	0.00257	0.01671	0.01141	
2652	regulation of interleukin-1 production	0	10	266	801	966	15
GO:003		33/171	268/23	0.00258	0.01680	0.01147	
4599	cellular response to oxidative stress	0	210	841	167	681	33
GO:000		53/171	485/23	0.00260	0.01691	0.01155	
7264	small GTPase mediated signal transduction	0	210	897	629	51	53
GO:005		19/171	128/23	0.00263	0.01703	0.01163	
1092	positive regulation of NF-kappaB transcription factor activity	0	210	326	598	686	19
GO:190		19/171	128/23	0.00263	0.01703	0.01163	
1343	negative regulation of vasculature development	0	210	326	598	686	19
GO:007		34/171	279/23	0.00266	0.01724	0.01177	
1902	positive regulation of protein serine/threonine kinase activity	0	210	822	31	834	34
GO:004		37/171	311/23	0.00270	0.01746	0.01193	
4282	small molecule catabolic process	0	210	567	579	045	37
GO:003		31/171	248/23	0.00272	0.01753	0.01198	
2147	activation of protein kinase activity	0	210	225	963	089	31
GO:000		18/171	119/23	0.00273	0.01753	0.01198	
3158	endothelium development	0	210	058	963	089	18
GO:005		18/171	119/23	0.00273	0.01753	0.01198	
1282	regulation of sequestering of calcium ion	0	210	058	963	089	18
GO:003		13/171	74/232	0.00273	0.01753	0.01198	
2651	regulation of interleukin-1 beta production	0	10	212	963	089	13
GO:200	positive regulation of DNA biosynthetic process	13/171	74/232	0.00273	0.01753	0.01198	13

0573		0	10	212	963	089	
GO:000			34/232	0.00274	0.01754	0.01198	
0002	mitochondrial genome maintenance	8/1710	10	565	933	752	8
GO:004			34/232	0.00274	0.01754	0.01198	
4275	cellular carbohydrate catabolic process	8/1710	10	565	933	752	8
GO:004			34/232	0.00274	0.01754	0.01198	
8679	regulation of axon regeneration	8/1710	10	565	933	752	8
GO:009			34/232	0.00274	0.01754	0.01198	
0322	regulation of superoxide metabolic process	8/1710	10	565	933	752	8
GO:003			27/232	0.00278	0.01777	0.01213	
2801	receptor catabolic process	7/1710	10	357	228	981	7
GO:190		14/171	83/232	0.00283	0.01806	0.01233	
1888	regulation of cell junction assembly	0	10	18	046	665	14
GO:000		15/171	92/232	0.00286	0.01825	0.01247	
6476	protein deacetylation	0	10	898	77	138	15
GO:006		15/171	92/232	0.00286	0.01825	0.01247	
0998	regulation of dendritic spine development	0	10	898	77	138	15
GO:004		19/171	129/23	0.00287	0.01828	0.01249	
2177	negative regulation of protein catabolic process	0	210	977	655	109	19
GO:004		19/171	129/23	0.00287	0.01828	0.01249	
5995	regulation of embryonic development	0	210	977	655	109	19
GO:003		31/171	249/23	0.00289	0.01836	0.01254	
1503	protein-containing complex localization	0	210	56	711	612	31
GO:003		12/171	66/232	0.00291	0.01846	0.01261	
2922	circadian regulation of gene expression	0	10	764	68	422	12
GO:190	negative regulation of cellular response to transforming growth factor beta	12/171	66/232	0.00291	0.01846	0.01261	12

3845	stimulus	0	10	764	68	422	
GO:005		18/171	120/23	0.00299	0.01894	0.01294	
1208	sequestering of calcium ion	0	210	633	434	041	18
GO:000			42/232	0.00302	0.01905	0.01301	
0959	mitochondrial RNA metabolic process	9/1710	10	434	956	912	9
GO:003			42/232	0.00302	0.01905	0.01301	
0866	cortical actin cytoskeleton organization	9/1710	10	434	956	912	9
GO:005			42/232	0.00302	0.01905	0.01301	
5023	positive regulation of cardiac muscle tissue growth	9/1710	10	434	956	912	9
GO:000		45/171	400/23	0.00306	0.01926	0.01315	
6979	response to oxidative stress	0	210	032	551	98	45
GO:004		13/171	75/232	0.00308	0.01940	0.01325	
5843	negative regulation of striated muscle tissue development	0	10	669	538	533	13
GO:000		17/171	111/23	0.00309	0.01940	0.01325	
8637	apoptotic mitochondrial changes	0	210	321	538	533	17
GO:007		17/171	111/23	0.00309	0.01940	0.01325	
1901	negative regulation of protein serine/threonine kinase activity	0	210	321	538	533	17
GO:003		10/171	50/232	0.00310	0.01940	0.01325	
0865	cortical cytoskeleton organization	0	10	579	538	533	10
GO:003		10/171	50/232	0.00310	0.01940	0.01325	
2330	regulation of chondrocyte differentiation	0	10	579	538	533	10
GO:005		10/171	50/232	0.00310	0.01940	0.01325	
1055	negative regulation of lipid biosynthetic process	0	10	579	538	533	10
GO:007		10/171	50/232	0.00310	0.01940	0.01325	
1675	regulation of mononuclear cell migration	0	10	579	538	533	10
GO:000	cellular glucose homeostasis	22/171	159/23	0.00313	0.01958	0.01337	22

1678		0	210	733	15	564	
GO:003		24/171	179/23	0.00318	0.01984	0.01355	
4612	response to tumor necrosis factor	0	210	352	859	808	24
GO:007		20/171	140/23	0.00325	0.02029	0.01386	
1333	cellular response to glucose stimulus	0	210	813	212	105	20
GO:001			21/232	0.00327	0.02038	0.01392	
0560	positive regulation of glycoprotein biosynthetic process	6/1710	10	938	102	177	6
GO:190			21/232	0.00327	0.02038	0.01392	
1626	regulation of postsynaptic membrane organization	6/1710	10	938	102	177	6
GO:000		18/171	121/23	0.00328	0.02038	0.01392	
7030	Golgi organization	0	210	326	343	342	18
GO:001			35/232	0.00333	0.02062	0.01408	
4823	response to activity	8/1710	10	294	61	918	8
GO:006			35/232	0.00333	0.02062	0.01408	
0142	regulation of syncytium formation by plasma membrane fusion	8/1710	10	294	61	918	8
GO:190			35/232	0.00333	0.02062	0.01408	
4707	positive regulation of vascular smooth muscle cell proliferation	8/1710	10	294	61	918	8
GO:000		17/171	112/23	0.00340	0.02098	0.01433	
8630	intrinsic apoptotic signaling pathway in response to DNA damage	0	210	225	833	661	17
GO:004		17/171	112/23	0.00340	0.02098	0.01433	
6822	regulation of nucleocytoplasmic transport	0	210	225	833	661	17
GO:190		17/171	112/23	0.00340	0.02098	0.01433	
5330	regulation of morphogenesis of an epithelium	0	210	225	833	661	17
GO:000			15/232	0.00346	0.02114	0.01444	
9143	nucleoside triphosphate catabolic process	5/1710	10	586	742	528	5
GO:001	regulation of macrophage cytokine production	5/1710	15/232	0.00346	0.02114	0.01444	5

0935			10	586	742	528	
GO:003			15/232	0.00346	0.02114	0.01444	
0730	sequestering of triglyceride	5/1710	10	586	742	528	5
GO:004			15/232	0.00346	0.02114	0.01444	
8681	negative regulation of axon regeneration	5/1710	10	586	742	528	5
GO:006			15/232	0.00346	0.02114	0.01444	
1051	positive regulation of cell growth involved in cardiac muscle cell development	5/1710	10	586	742	528	5
GO:007			15/232	0.00346	0.02114	0.01444	
0293	renal absorption	5/1710	10	586	742	528	5
GO:190	regulation of transcription from RNA polymerase II promoter involved in heart		15/232	0.00346	0.02114	0.01444	
1213	development	5/1710	10	586	742	528	5
GO:190			15/232	0.00346	0.02114	0.01444	
2430	negative regulation of amyloid-beta formation	5/1710	10	586	742	528	5
GO:190			15/232	0.00346	0.02114	0.01444	
3729	regulation of plasma membrane organization	5/1710	10	586	742	528	5
GO:003		13/171	76/232	0.00347	0.02114	0.01444	
0168	platelet activation	0	10	784	742	528	13
GO:001			28/232	0.00347	0.02114	0.01444	
0719	negative regulation of epithelial to mesenchymal transition	7/1710	10	872	742	528	7
GO:006			28/232	0.00347	0.02114	0.01444	
0765	regulation of androgen receptor signaling pathway	7/1710	10	872	742	528	7
GO:006			28/232	0.00347	0.02114	0.01444	
1050	regulation of cell growth involved in cardiac muscle cell development	7/1710	10	872	742	528	7
GO:006			28/232	0.00347	0.02114	0.01444	
1311	cell surface receptor signaling pathway involved in heart development	7/1710	10	872	742	528	7
GO:000	phosphatidylinositol biosynthetic process	11/171	59/232	0.00350	0.02126	0.01452	11

6661		0	10	878	382	479	
GO:000		11/171	59/232	0.00350	0.02126	0.01452	
7029	endoplasmic reticulum organization	0	10	878	382	479	11
GO:000		11/171	59/232	0.00350	0.02126	0.01452	
8347	glial cell migration	0	10	878	382	479	11
GO:006		44/171	392/23	0.00352	0.02132	0.01456	
0249	anatomical structure homeostasis	0	210	229	355	559	44
GO:004			43/232	0.00357	0.02162	0.01477	
8246	macrophage chemotaxis	9/1710	10	928	375	065	9
GO:008			43/232	0.00357	0.02162	0.01477	
6002	cardiac muscle cell action potential involved in contraction	9/1710	10	928	375	065	9
GO:004		18/171	122/23	0.00359	0.02165	0.01479	
8284	organelle fusion	0	210	263	957	512	18
GO:006		18/171	122/23	0.00359	0.02165	0.01479	
1013	regulation of mRNA catabolic process	0	210	263	957	512	18
GO:001		50/171	459/23	0.00360	0.02170	0.01482	
0498	proteasomal protein catabolic process	0	210	435	784	809	50
GO:003		10/171	51/232	0.00361	0.02171	0.01483	
0834	regulation of actin filament depolymerization	0	10	306	372	21	10
GO:004		26/171	201/23	0.00361	0.02171	0.01483	
8736	appendage development	0	210	849	372	21	26
GO:006		26/171	201/23	0.00361	0.02171	0.01483	
0173	limb development	0	210	849	372	21	26
GO:004		35/171	295/23	0.00362	0.02171	0.01483	
3405	regulation of MAP kinase activity	0	210	019	372	21	35
GO:004	positive regulation of innate immune response	29/171	232/23	0.00365	0.02189	0.01495	29

5089		0	210	537	93	887	
GO:200		22/171	161/23	0.00366	0.02189	0.01495	
1236	regulation of extrinsic apoptotic signaling pathway	0	210	094	93	887	22
GO:000		53/171	493/23	0.00366	0.02189	0.01495	
7346	regulation of mitotic cell cycle	0	210	238	93	887	53
GO:000		31/171	253/23	0.00368	0.02199	0.01502	
5996	monosaccharide metabolic process	0	210	639	784	618	31
GO:190		31/171	253/23	0.00368	0.02199	0.01502	
3311	regulation of mRNA metabolic process	0	210	639	784	618	31
GO:000		12/171	68/232	0.00376	0.02243	0.01532	
6855	drug transmembrane transport	0	10	773	739	643	12
GO:003		12/171	68/232	0.00376	0.02243	0.01532	
4109	homotypic cell-cell adhesion	0	10	773	739	643	12
GO:007		20/171	142/23	0.00384	0.02285	0.01561	
1331	cellular response to hexose stimulus	0	210	225	789	366	20
GO:000		16/171	104/23	0.00385	0.02289	0.01564	
6413	translational initiation	0	210	299	846	138	16
GO:003		13/171	77/232	0.00390	0.02317	0.01583	
0193	regulation of blood coagulation	0	10	825	971	349	13
GO:004		13/171	77/232	0.00390	0.02317	0.01583	
8635	negative regulation of muscle organ development	0	10	825	971	349	13
GO:004		18/171	123/23	0.00392	0.02325	0.01588	
2157	lipoprotein metabolic process	0	210	574	981	82	18
GO:004		15/171	95/232	0.00393	0.02326	0.01589	
2158	lipoprotein biosynthetic process	0	10	087	662	286	15
GO:003	interleukin-1 beta production	14/171	86/232	0.00395	0.02338	0.01597	14

2611		0	10	535	779	562	
GO:003		28/171	223/23	0.00398	0.02351	0.01606	
0073	insulin secretion	0	210	055	303	117	28
GO:004		48/171	439/23	0.00398	0.02351	0.01606	
2391	regulation of membrane potential	0	210	463	336	139	48
GO:001		47/171	428/23	0.00400	0.02360	0.01612	
6055	Wnt signaling pathway	0	210	783	498	398	47
GO:004			36/232	0.00401	0.02360	0.01612	
8009	insulin-like growth factor receptor signaling pathway	8/1710	10	314	498	398	8
GO:007			36/232	0.00401	0.02360	0.01612	
1320	cellular response to cAMP	8/1710	10	314	498	398	8
GO:000		11/171	60/232	0.00401	0.02360	0.01612	
6446	regulation of translational initiation	0	10	632	498	398	11
GO:000		42/171	373/23	0.00405	0.02378	0.01624	
9914	hormone transport	0	210	149	775	883	42
GO:004		17/171	114/23	0.00409	0.02403	0.01641	
3534	blood vessel endothelial cell migration	0	210	707	122	513	17
GO:007		20/171	143/23	0.00416	0.02428	0.01658	
1326	cellular response to monosaccharide stimulus	0	210	545	585	906	20
GO:007		20/171	143/23	0.00416	0.02428	0.01658	
2073	kidney epithelium development	0	210	545	585	906	20
GO:000		38/171	330/23	0.00417	0.02428	0.01658	
6869	lipid transport	0	210	871	585	906	38
GO:006		10/171	52/232	0.00418	0.02428	0.01658	
1614	pri-miRNA transcription by RNA polymerase II	0	10	381	585	906	10
GO:200	regulation of transcription regulatory region DNA binding	10/171	52/232	0.00418	0.02428	0.01658	10

0677		0	10	381	585	906	
GO:004		25/171	193/23	0.00419	0.02428	0.01658	
8771	tissue remodeling	0	210	327	585	906	25
GO:001			44/232	0.00421	0.02428	0.01658	
4904	myotube cell development	9/1710	10	101	585	906	9
GO:005			44/232	0.00421	0.02428	0.01658	
0918	positive chemotaxis	9/1710	10	101	585	906	9
GO:006			44/232	0.00421	0.02428	0.01658	
1098	positive regulation of protein tyrosine kinase activity	9/1710	10	101	585	906	9
GO:001		21/171	153/23	0.00422	0.02428	0.01658	
0950	positive regulation of endopeptidase activity	0	210	668	585	906	21
GO:003		21/171	153/23	0.00422	0.02428	0.01658	
1056	regulation of histone modification	0	210	668	585	906	21
GO:003			22/232	0.00423	0.02428	0.01658	
2331	negative regulation of chondrocyte differentiation	6/1710	10	167	585	906	6
GO:003			22/232	0.00423	0.02428	0.01658	
2897	negative regulation of viral transcription	6/1710	10	167	585	906	6
GO:010			22/232	0.00423	0.02428	0.01658	
1023	vascular endothelial cell proliferation	6/1710	10	167	585	906	6
GO:190			22/232	0.00423	0.02428	0.01658	
1741	positive regulation of myoblast fusion	6/1710	10	167	585	906	6
GO:190			22/232	0.00423	0.02428	0.01658	
3055	positive regulation of extracellular matrix organization	6/1710	10	167	585	906	6
GO:190			22/232	0.00423	0.02428	0.01658	
5562	regulation of vascular endothelial cell proliferation	6/1710	10	167	585	906	6
GO:005	protein tetramerization	24/171	183/23	0.00423	0.02428	0.01658	24

1262		0	210	503	585	906	
GO:003		16/171	105/23	0.00424	0.02428	0.01658	
4754	cellular hormone metabolic process	0	210	223	585	906	16
GO:001		12/171	69/232	0.00426	0.02428	0.01658	
0633	negative regulation of epithelial cell migration	0	10	181	585	906	12
GO:004		12/171	69/232	0.00426	0.02428	0.01658	
3407	negative regulation of MAP kinase activity	0	10	181	585	906	12
GO:190		12/171	69/232	0.00426	0.02428	0.01658	
3670	regulation of sprouting angiogenesis	0	10	181	585	906	12
GO:190		12/171	69/232	0.00426	0.02428	0.01658	
4589	regulation of protein import	0	10	181	585	906	12
GO:000		30/171	245/23	0.00427	0.02428	0.01658	
7411	axon guidance	0	210	232	585	906	30
GO:005		30/171	245/23	0.00427	0.02428	0.01658	
1054	positive regulation of DNA metabolic process	0	210	232	585	906	30
GO:003		18/171	124/23	0.00428	0.02428	0.01658	
3138	positive regulation of peptidyl-serine phosphorylation	0	210	395	585	906	18
GO:003			10/232	0.00429	0.02428	0.01658	
2908	regulation of transforming growth factor beta1 production	4/1710	10	746	585	906	4
GO:004			10/232	0.00429	0.02428	0.01658	
4154	histone H3-K14 acetylation	4/1710	10	746	585	906	4
GO:004			10/232	0.00429	0.02428	0.01658	
6543	development of secondary female sexual characteristics	4/1710	10	746	585	906	4
GO:190			10/232	0.00429	0.02428	0.01658	
3749	positive regulation of establishment of protein localization to mitochondrion	4/1710	10	746	585	906	4
GO:190	positive regulation of production of miRNAs involved in gene silencing by miRNA	4/1710	10/232	0.00429	0.02428	0.01658	4

3800			10	746	585	906	
GO:190			10/232	0.00429	0.02428	0.01658	
4851	positive regulation of establishment of protein localization to telomere	4/1710	10	746	585	906	4
GO:190			10/232	0.00429	0.02428	0.01658	
5097	regulation of guanyl-nucleotide exchange factor activity	4/1710	10	746	585	906	4
GO:190			10/232	0.00429	0.02428	0.01658	
5245	regulation of aspartic-type peptidase activity	4/1710	10	746	585	906	4
GO:000			29/232	0.00429	0.02428	0.01658	
7007	inner mitochondrial membrane organization	7/1710	10	845	585	906	7
GO:007			29/232	0.00429	0.02428	0.01658	
0296	sarcoplasmic reticulum calcium ion transport	7/1710	10	845	585	906	7
GO:007			29/232	0.00429	0.02428	0.01658	
1353	cellular response to interleukin-4	7/1710	10	845	585	906	7
GO:009			29/232	0.00429	0.02428	0.01658	
9622	cardiac muscle cell membrane repolarization	7/1710	10	845	585	906	7
GO:003		27/171	214/23	0.00433	0.02445	0.01670	
3673	negative regulation of kinase activity	0	210	23	343	354	27
GO:019		47/171	430/23	0.00437	0.02467	0.01685	
8738	cell-cell signaling by wnt	0	210	591	573	539	47
GO:005		13/171	78/232	0.00438	0.02467	0.01685	
1881	regulation of mitochondrial membrane potential	0	10	072	906	766	13
GO:000		26/171	204/23	0.00441	0.02485	0.01697	
7160	cell-matrix adhesion	0	210	575	128	53	26
GO:000		19/171	134/23	0.00441	0.02485	0.01697	
6633	fatty acid biosynthetic process	0	210	98	128	53	19
GO:001	macroautophagy	25/171	194/23	0.00448	0.02519	0.01721	25

6236		0	210	567	74	172	
GO:000		28/171	225/23	0.00450	0.02529	0.01727	
6650	glycerophospholipid metabolic process	0	210	92	028	517	28
GO:000		20/171	144/23	0.00451	0.02529	0.01727	
7613	memory	0	210	086	028	517	20
GO:000		11/171	61/232	0.00458	0.02563	0.01750	
6493	protein O-linked glycosylation	0	10	067	242	888	11
GO:004		11/171	61/232	0.00458	0.02563	0.01750	
8286	lung alveolus development	0	10	067	242	888	11
GO:003		27/171	215/23	0.00461	0.02580	0.01762	
5265	organ growth	0	210	606	575	727	27
GO:004		39/171	343/23	0.00464	0.02593	0.01771	
4403	symbiont process	0	210	384	624	64	39
GO:005		40/171	354/23	0.00465	0.02596	0.01773	
0727	regulation of inflammatory response	0	210	893	901	879	40
GO:004		16/171	106/23	0.00466	0.02596	0.01773	
6474	glycerophospholipid biosynthetic process	0	210	304	901	879	16
GO:006		16/171	106/23	0.00466	0.02596	0.01773	
0840	artery development	0	210	304	901	879	16
GO:001		18/171	125/23	0.00466	0.02597	0.01774	
8107	peptidyl-threonine phosphorylation	0	210	864	543	318	18
GO:004		26/171	205/23	0.00471	0.02619	0.01789	
2098	T cell proliferation	0	210	22	285	169	26
GO:000			16/232	0.00473	0.02621	0.01790	
2689	negative regulation of leukocyte chemotaxis	5/1710	10	856	465	658	5
GO:000	activation of NF-kappaB-inducing kinase activity	5/1710	16/232	0.00473	0.02621	0.01790	5

7250			10	856	465	658	
GO:003			16/232	0.00473	0.02621	0.01790	
4134	toll-like receptor 2 signaling pathway	5/1710	10	856	465	658	5
GO:004			16/232	0.00473	0.02621	0.01790	
2407	cristae formation	5/1710	10	856	465	658	5
GO:004			16/232	0.00473	0.02621	0.01790	
5947	negative regulation of translational initiation	5/1710	10	856	465	658	5
GO:001		28/171	226/23	0.00479	0.02642	0.01805	
0948	negative regulation of cell cycle process	0	210	488	959	34	28
GO:004		25/171	195/23	0.00479	0.02642	0.01805	
2180	cellular ketone metabolic process	0	210	492	959	34	25
GO:008			37/232	0.00479	0.02642	0.01805	
6065	cell communication involved in cardiac conduction	8/1710	10	551	959	34	8
GO:190			37/232	0.00479	0.02642	0.01805	
2229	regulation of intrinsic apoptotic signaling pathway in response to DNA damage	8/1710	10	551	959	34	8
GO:001		10/171	53/232	0.00482	0.02648	0.01808	
0517	regulation of phospholipase activity	0	10	336	316	999	10
GO:004		10/171	53/232	0.00482	0.02648	0.01808	
8662	negative regulation of smooth muscle cell proliferation	0	10	336	316	999	10
GO:190		10/171	53/232	0.00482	0.02648	0.01808	
4705	regulation of vascular smooth muscle cell proliferation	0	10	336	316	999	10
GO:199		10/171	53/232	0.00482	0.02648	0.01808	
0874	vascular smooth muscle cell proliferation	0	10	336	316	999	10
GO:005		24/171	185/23	0.00486	0.02666	0.01821	
1668	localization within membrane	0	210	108	521	435	24
GO:003	skeletal muscle cell differentiation	14/171	88/232	0.00488	0.02677	0.01829	14

5914		0	10	8	924	224	
GO:001		41/171	366/23	0.00489	0.02677	0.01829	
6311	dephosphorylation	0	210	104	924	224	41
GO:005		13/171	79/232	0.00489	0.02679	0.01830	
1781	positive regulation of cell division	0	10	813	298	163	13
GO:004			45/232	0.00492	0.02688	0.01836	
2987	amyloid precursor protein catabolic process	9/1710	10	642	486	439	9
GO:190			45/232	0.00492	0.02688	0.01836	
2893	regulation of pri-miRNA transcription by RNA polymerase II	9/1710	10	642	486	439	9
GO:004		22/171	165/23	0.00492	0.02688	0.01836	
3122	regulation of I-kappaB kinase/NF-kappaB signaling	0	210	874	486	439	22
GO:001		48/171	444/23	0.00493	0.02692	0.01838	
6569	covalent chromatin modification	0	210	981	013	848	48
GO:001		32/171	269/23	0.00502	0.02738	0.01870	
0038	response to metal ion	0	210	967	429	553	32
GO:003		18/171	126/23	0.00508	0.02763	0.01887	
1623	receptor internalization	0	210	126	938	978	18
GO:004			30/232	0.00525	0.02846	0.01944	
2168	heme metabolic process	7/1710	10	61	221	183	7
GO:006			30/232	0.00525	0.02846	0.01944	
1037	negative regulation of cartilage development	7/1710	10	61	221	183	7
GO:006			30/232	0.00525	0.02846	0.01944	
1082	myeloid leukocyte cytokine production	7/1710	10	61	221	183	7
GO:190			30/232	0.00525	0.02846	0.01944	
2003	regulation of amyloid-beta formation	7/1710	10	61	221	183	7
GO:000	pattern specification process	50/171	468/23	0.00525	0.02846	0.01944	50

7389		0	210	858	221	183	
GO:004		23/171	176/23	0.00526	0.02846	0.01944	
8592	eye morphogenesis	0	210	176	221	183	23
GO:000		21/171	156/23	0.00530	0.02863	0.01955	
7596	blood coagulation	0	210	26	01	651	21
GO:005		21/171	156/23	0.00530	0.02863	0.01955	
1302	regulation of cell division	0	210	26	01	651	21
GO:000		26/171	207/23	0.00535	0.02888	0.01973	
6402	mRNA catabolic process	0	210	518	731	221	26
GO:000			23/232	0.00537	0.02890	0.01974	
0272	polysaccharide catabolic process	6/1710	10	348	594	494	6
GO:006			23/232	0.00537	0.02890	0.01974	
0390	regulation of SMAD protein signal transduction	6/1710	10	348	594	494	6
GO:190			23/232	0.00537	0.02890	0.01974	
3020	positive regulation of glycoprotein metabolic process	6/1710	10	348	594	494	6
GO:002		30/171	249/23	0.00539	0.02895	0.01977	
1537	telencephalon development	0	210	247	481	832	30
GO:009		30/171	249/23	0.00539	0.02895	0.01977	
0068	positive regulation of cell cycle process	0	210	247	481	832	30
GO:001		12/171	71/232	0.00540	0.02899	0.01980	
4068	positive regulation of phosphatidylinositol 3-kinase signaling	0	10	523	667	691	12
GO:003		14/171	89/232	0.00541	0.02903	0.01983	
1016	pancreas development	0	10	672	164	08	14
GO:004	negative regulation of cysteine-type endopeptidase activity involved in apoptotic	13/171	80/232	0.00546	0.02922	0.01996	
3154	process	0	10	346	85	527	13
GO:190	positive regulation of calcium ion transmembrane transport	13/171	80/232	0.00546	0.02922	0.01996	13

4427		0	10	346	85	527	
GO:003		10/171	54/232	0.00553	0.02956	0.02019	
2964	collagen biosynthetic process	0	10	717	87	765	10
GO:005		10/171	54/232	0.00553	0.02956	0.02019	
1496	positive regulation of stress fiber assembly	0	10	717	87	765	10
GO:004		43/171	391/23	0.00558	0.02977	0.02034	
3900	regulation of multi-organism process	0	210	163	888	122	43
GO:003		38/171	336/23	0.00562	0.02996	0.02046	
2102	negative regulation of response to external stimulus	0	210	193	655	941	38
GO:004		19/171	137/23	0.00563	0.02999	0.02049	
8593	camera-type eye morphogenesis	0	210	292	773	071	19
GO:000			38/232	0.00568	0.03024	0.02065	
6778	porphyrin-containing compound metabolic process	8/1710	10	956	423	909	8
GO:007			38/232	0.00568	0.03024	0.02065	
0570	regulation of neuron projection regeneration	8/1710	10	956	423	909	8
GO:000		26/171	208/23	0.00570	0.03026	0.02067	
7623	circadian rhythm	0	210	31	113	063	26
GO:190		26/171	208/23	0.00570	0.03026	0.02067	
3050	regulation of proteolysis involved in cellular protein catabolic process	0	210	31	113	063	26
GO:001			46/232	0.00573	0.03036	0.02073	
9674	NAD metabolic process	9/1710	10	263	265	998	9
GO:004			46/232	0.00573	0.03036	0.02073	
5010	actin nucleation	9/1710	10	263	265	998	9
GO:003		34/171	293/23	0.00576	0.03047	0.02081	
0100	regulation of endocytosis	0	210	356	125	416	34
GO:005	neuron apoptotic process	34/171	293/23	0.00576	0.03047	0.02081	34

1402		0	210	356	125	416	
GO:004		15/171	99/232	0.00582	0.03074	0.02099	
5639	positive regulation of myeloid cell differentiation	0	10	49	187	901	15
GO:000		31/171	261/23	0.00582	0.03074	0.02099	
6520	cellular amino acid metabolic process	0	210	527	187	901	31
GO:004		36/171	315/23	0.00585	0.03087	0.02109	
8872	homeostasis of number of cells	0	210	648	866	245	36
GO:004		39/171	348/23	0.00590	0.03110	0.02124	
3010	camera-type eye development	0	210	428	264	545	39
GO:006		14/171	90/232	0.00599	0.03152	0.02153	
0395	SMAD protein signal transduction	0	10	047	821	614	14
GO:001		23/171	178/23	0.00603	0.03173	0.02167	
0951	negative regulation of endopeptidase activity	0	210	5	397	67	23
GO:190		34/171	294/23	0.00606	0.03187	0.02177	
2105	regulation of leukocyte differentiation	0	210	912	137	055	34
GO:000		13/171	81/232	0.00607	0.03187	0.02177	
6664	glycolipid metabolic process	0	10	975	137	055	13
GO:005		13/171	81/232	0.00607	0.03187	0.02177	
0818	regulation of coagulation	0	10	975	137	055	13
GO:004		48/171	449/23	0.00608	0.03187	0.02177	
8608	reproductive structure development	0	210	374	137	055	48
GO:000		19/171	138/23	0.00609	0.03187	0.02177	
7050	cell cycle arrest	0	210	32	137	055	19
GO:003		28/171	230/23	0.00609	0.03187	0.02177	
6293	response to decreased oxygen levels	0	210	386	137	055	28
GO:000	adult locomotory behavior	16/171	109/23	0.00613	0.03198	0.02185	16

8344		0	210	368	893	085	
GO:003		16/171	109/23	0.00613	0.03198	0.02185	
2963	collagen metabolic process	0	210	368	893	085	16
GO:000		20/171	148/23	0.00613	0.03198	0.02185	
3231	cardiac ventricle development	0	210	779	893	085	20
GO:000		21/171	158/23	0.00613	0.03198	0.02185	
7599	hemostasis	0	210	824	893	085	21
GO:000		37/171	327/23	0.00617	0.03216	0.02196	
7204	positive regulation of cytosolic calcium ion concentration	0	210	7	223	923	37
GO:001		46/171	427/23	0.00621	0.03232	0.02208	
6570	histone modification	0	210	445	843	275	46
GO:000			17/232	0.00631	0.03250	0.02220	
1820	serotonin secretion	5/1710	10	076	487	328	5
GO:000			17/232	0.00631	0.03250	0.02220	
7096	regulation of exit from mitosis	5/1710	10	076	487	328	5
GO:001			17/232	0.00631	0.03250	0.02220	
0934	macrophage cytokine production	5/1710	10	076	487	328	5
GO:003			17/232	0.00631	0.03250	0.02220	
5269	protein O-linked mannosylation	5/1710	10	076	487	328	5
GO:007			17/232	0.00631	0.03250	0.02220	
0571	negative regulation of neuron projection regeneration	5/1710	10	076	487	328	5
GO:190			17/232	0.00631	0.03250	0.02220	
2992	negative regulation of amyloid precursor protein catabolic process	5/1710	10	076	487	328	5
GO:000		10/171	55/232	0.00633	0.03250	0.02220	
2833	positive regulation of response to biotic stimulus	0	10	086	487	328	10
GO:003	actin filament depolymerization	10/171	55/232	0.00633	0.03250	0.02220	10

0042		0	10	086	487	328	
GO:200		17/171	119/23	0.00635	0.03250	0.02220	
0278	regulation of DNA biosynthetic process	0	210	523	487	328	17
GO:000			11/232	0.00636	0.03250	0.02220	
2468	dendritic cell antigen processing and presentation	4/1710	10	231	487	328	4
GO:000			11/232	0.00636	0.03250	0.02220	
3263	cardioblast proliferation	4/1710	10	231	487	328	4
GO:000			11/232	0.00636	0.03250	0.02220	
3266	regulation of secondary heart field cardioblast proliferation	4/1710	10	231	487	328	4
GO:003			11/232	0.00636	0.03250	0.02220	
1077	post-embryonic camera-type eye development	4/1710	10	231	487	328	4
GO:003			11/232	0.00636	0.03250	0.02220	
2905	transforming growth factor beta1 production	4/1710	10	231	487	328	4
GO:004			11/232	0.00636	0.03250	0.02220	
8312	intracellular distribution of mitochondria	4/1710	10	231	487	328	4
GO:007			11/232	0.00636	0.03250	0.02220	
0203	regulation of establishment of protein localization to telomere	4/1710	10	231	487	328	4
GO:008			11/232	0.00636	0.03250	0.02220	
6103	G protein-coupled receptor signaling pathway involved in heart process	4/1710	10	231	487	328	4
GO:009			11/232	0.00636	0.03250	0.02220	
7084	vascular smooth muscle cell development	4/1710	10	231	487	328	4
GO:190			11/232	0.00636	0.03250	0.02220	
1163	regulation of trophoblast cell migration	4/1710	10	231	487	328	4
GO:000			31/232	0.00636	0.03250	0.02220	
2026	regulation of the force of heart contraction	7/1710	10	521	487	328	7
GO:004	neurotrophin TRK receptor signaling pathway	7/1710	31/232	0.00636	0.03250	0.02220	7

8011			10	521	487	328	
GO:000		41/171	372/23	0.00644	0.03288	0.02246	
1505	regulation of neurotransmitter levels	0	210	445	078	005	41
GO:001		21/171	159/23	0.00659	0.03356	0.02292	
0970	transport along microtubule	0	210	49	045	432	21
GO:005		21/171	159/23	0.00659	0.03356	0.02292	
0817	coagulation	0	210	49	045	432	21
GO:009		21/171	159/23	0.00659	0.03356	0.02292	
9111	microtubule-based transport	0	210	49	045	432	21
GO:000			47/232	0.00663	0.03374	0.02305	
3179	heart valve morphogenesis	9/1710	10	689	476	022	9
GO:004		11/171	64/232	0.00665	0.03376	0.02306	
2267	natural killer cell mediated cytotoxicity	0	10	822	505	408	11
GO:004		11/171	64/232	0.00665	0.03376	0.02306	
2306	regulation of protein import into nucleus	0	10	822	505	408	11
GO:190		11/171	64/232	0.00665	0.03376	0.02306	
3391	regulation of adherens junction organization	0	10	822	505	408	11
GO:000		16/171	110/23	0.00669	0.03381	0.02309	
6986	response to unfolded protein	0	210	979	138	572	16
GO:004			39/232	0.00670	0.03381	0.02309	
6885	regulation of hormone biosynthetic process	8/1710	10	5	138	572	8
GO:006			39/232	0.00670	0.03381	0.02309	
0251	regulation of glial cell proliferation	8/1710	10	5	138	572	8
GO:009			39/232	0.00670	0.03381	0.02309	
0049	regulation of cell migration involved in sprouting angiogenesis	8/1710	10	5	138	572	8
GO:000	atrioventricular valve morphogenesis	6/1710	24/232	0.00672	0.03381	0.02309	6

3181			10	523	138	572	
GO:003			24/232	0.00672	0.03381	0.02309	
2352	positive regulation of hormone metabolic process	6/1710	10	523	138	572	6
GO:004			24/232	0.00672	0.03381	0.02309	
6685	response to arsenic-containing substance	6/1710	10	523	138	572	6
GO:007			24/232	0.00672	0.03381	0.02309	
0584	mitochondrion morphogenesis	6/1710	10	523	138	572	6
GO:190			24/232	0.00672	0.03381	0.02309	
2683	regulation of receptor localization to synapse	6/1710	10	523	138	572	6
GO:200			24/232	0.00672	0.03381	0.02309	
0637	positive regulation of gene silencing by miRNA	6/1710	10	523	138	572	6
GO:190		13/171	82/232	0.00675	0.03390	0.02316	
3509	liposaccharide metabolic process	0	10	014	74	131	13
GO:005		12/171	73/232	0.00677	0.03396	0.02320	
0810	regulation of steroid biosynthetic process	0	10	987	914	349	12
GO:005		12/171	73/232	0.00677	0.03396	0.02320	
1851	modification by host of symbiont morphology or physiology	0	10	987	914	349	12
GO:190		12/171	73/232	0.00677	0.03396	0.02320	
3036	positive regulation of response to wounding	0	10	987	914	349	12
GO:001		36/171	318/23	0.00678	0.03398	0.02321	
8105	peptidyl-serine phosphorylation	0	210	791	029	11	36
GO:004		31/171	264/23	0.00685	0.03428	0.02342	
3523	regulation of neuron apoptotic process	0	210	825	889	19	31
GO:001		26/171	211/23	0.00686	0.03428	0.02342	
0810	regulation of cell-substrate adhesion	0	210	13	889	19	26
GO:190	regulation of hemopoiesis	44/171	407/23	0.00689	0.03440	0.02350	44

3706		0	210	104	806	33	
GO:004		22/171	170/23	0.00700	0.03494	0.02387	
2129	regulation of T cell proliferation	0	210	469	571	055	22
GO:005		15/171	101/23	0.00701	0.03497	0.02388	
0848	regulation of calcium-mediated signaling	0	210	569	068	761	15
GO:005		41/171	374/23	0.00704	0.03509	0.02397	
1480	regulation of cytosolic calcium ion concentration	0	210	679	578	306	41
GO:004		21/171	160/23	0.00707	0.03522	0.02406	
3433	negative regulation of DNA-binding transcription factor activity	0	210	899	611	209	21
GO:005		19/171	140/23	0.00710	0.03533	0.02413	
0921	positive regulation of chemotaxis	0	210	594	013	314	19
GO:007		20/171	150/23	0.00711	0.03534	0.02414	
1322	cellular response to carbohydrate stimulus	0	210	547	742	495	20
GO:006		48/171	453/23	0.00715	0.03550	0.02425	
1458	reproductive system development	0	210	366	694	392	48
GO:002		10/171	56/232	0.00721	0.03572	0.02440	
1575	hindbrain morphogenesis	0	10	016	668	401	10
GO:009		10/171	56/232	0.00721	0.03572	0.02440	
0303	positive regulation of wound healing	0	10	016	668	401	10
GO:005		24/171	191/23	0.00721	0.03573	0.02440	
0773	regulation of dendrite development	0	210	723	138	723	24
GO:000		23/171	181/23	0.00736	0.03644	0.02489	
9749	response to glucose	0	210	861	997	808	23
GO:005		38/171	342/23	0.00746	0.03689	0.02520	
0878	regulation of body fluid levels	0	210	463	369	117	38
GO:000	autophagy of mitochondrion	11/171	65/232	0.00749	0.03694	0.02523	11

0422		0	10	405	534	645	
GO:006		11/171	65/232	0.00749	0.03694	0.02523	
1726	mitochondrion disassembly	0	10	405	534	645	11
GO:009		11/171	65/232	0.00749	0.03694	0.02523	
0559	regulation of membrane permeability	0	10	405	534	645	11
GO:001		17/171	121/23	0.00750	0.03695	0.02524	
0906	regulation of glucose metabolic process	0	210	238	525	322	17
GO:001		12/171	74/232	0.00756	0.03719	0.02540	
0507	negative regulation of autophagy	0	10	331	264	537	12
GO:004		12/171	74/232	0.00756	0.03719	0.02540	
5453	bone resorption	0	10	331	264	537	12
GO:000			32/232	0.00763	0.03747	0.02559	
7031	peroxisome organization	7/1710	10	947	616	904	7
GO:001			48/232	0.00764	0.03747	0.02559	
0862	positive regulation of pathway-restricted SMAD protein phosphorylation	9/1710	10	663	616	904	9
GO:003			48/232	0.00764	0.03747	0.02559	
8066	p38MAPK cascade	9/1710	10	663	616	904	9
GO:009			48/232	0.00764	0.03747	0.02559	
0311	regulation of protein deacetylation	9/1710	10	663	616	904	9
GO:004		19/171	141/23	0.00766	0.03751	0.02562	
5017	glycerolipid biosynthetic process	0	210	126	644	655	19
GO:004		15/171	102/23	0.00768	0.03757	0.02566	
6849	bone remodeling	0	210	009	712	801	15
GO:005		26/171	213/23	0.00773	0.03778	0.02581	
0707	regulation of cytokine secretion	0	210	638	924	29	26
GO:005	regulation of chemotaxis	26/171	213/23	0.00773	0.03778	0.02581	26

0920		0	210	638	924	29	
GO:001		38/171	343/23	0.00781	0.03814	0.02605	
8209	peptidyl-serine modification	0	210	607	659	7	38
GO:000			40/232	0.00785	0.03819	0.02608	
9409	response to cold	8/1710	10	166	265	846	8
GO:001			40/232	0.00785	0.03819	0.02608	
0470	regulation of gastrulation	8/1710	10	166	265	846	8
GO:004			40/232	0.00785	0.03819	0.02608	
6456	icosanoid biosynthetic process	8/1710	10	166	265	846	8
GO:004			40/232	0.00785	0.03819	0.02608	
8741	skeletal muscle fiber development	8/1710	10	166	265	846	8
GO:000		14/171	93/232	0.00800	0.03892	0.02658	
0045	autophagosome assembly	0	10	865	39	796	14
GO:000		41/171	377/23	0.00803	0.03903	0.02666	
2237	response to molecule of bacterial origin	0	210	841	607	458	41
GO:003		32/171	278/23	0.00811	0.03935	0.02687	
0072	peptide hormone secretion	0	210	332	034	925	32
GO:003		28/171	235/23	0.00811	0.03935	0.02687	
2886	regulation of microtubule-based process	0	210	66	034	925	28
GO:006		17/171	122/23	0.00813	0.03938	0.02690	
0996	dendritic spine development	0	210	539	815	507	17
GO:000		10/171	57/232	0.00818	0.03938	0.02690	
3170	heart valve development	0	10	092	815	507	10
GO:001		10/171	57/232	0.00818	0.03938	0.02690	
0463	mesenchymal cell proliferation	0	10	092	815	507	10
GO:004	positive regulation of receptor-mediated endocytosis	10/171	57/232	0.00818	0.03938	0.02690	10

8260		0	10	092	815	507	
GO:005		10/171	57/232	0.00818	0.03938	0.02690	
0879	multicellular organismal movement	0	10	092	815	507	10
GO:005		10/171	57/232	0.00818	0.03938	0.02690	
0881	musculoskeletal movement	0	10	092	815	507	10
GO:005		10/171	57/232	0.00818	0.03938	0.02690	
1893	regulation of focal adhesion assembly	0	10	092	815	507	10
GO:009		10/171	57/232	0.00818	0.03938	0.02690	
0109	regulation of cell-substrate junction assembly	0	10	092	815	507	10
GO:000		24/171	193/23	0.00818	0.03938	0.02690	
7249	I-kappaB kinase/NF-kappaB signaling	0	210	508	815	507	24
GO:003			18/232	0.00821	0.03943	0.02693	
4389	lipid droplet organization	5/1710	10	556	739	871	5
GO:003	regulation of viral-induced cytoplasmic pattern recognition receptor signaling		18/232	0.00821	0.03943	0.02693	
9531	pathway	5/1710	10	556	739	871	5
GO:007			18/232	0.00821	0.03943	0.02693	
0131	positive regulation of mitochondrial translation	5/1710	10	556	739	871	5
GO:015		43/171	400/23	0.00823	0.03945	0.02695	
0063	visual system development	0	210	851	597	14	43
GO:005		29/171	246/23	0.00826	0.03945	0.02695	
1348	negative regulation of transferase activity	0	210	067	597	14	29
GO:003			25/232	0.00830	0.03945	0.02695	
2469	endoplasmic reticulum calcium ion homeostasis	6/1710	10	723	597	14	6
GO:005			25/232	0.00830	0.03945	0.02695	
1443	positive regulation of ubiquitin-protein transferase activity	6/1710	10	723	597	14	6
GO:006	positive regulation of posttranscriptional gene silencing	6/1710	25/232	0.00830	0.03945	0.02695	6

0148			10	723	597	14	
GO:0086019	cell-cell signaling involved in cardiac conduction	6/1710	25/23210	0.00830723	0.03945597	0.0269514	6
GO:0090280	positive regulation of calcium ion import	6/1710	25/23210	0.00830723	0.03945597	0.0269514	6
GO:0150077	regulation of neuroinflammatory response	6/1710	25/23210	0.00830723	0.03945597	0.0269514	6
GO:1901739	regulation of myoblast fusion	6/1710	25/23210	0.00830723	0.03945597	0.0269514	6
GO:1902230	negative regulation of intrinsic apoptotic signaling pathway in response to DNA damage	6/1710	25/23210	0.00830723	0.03945597	0.0269514	6
GO:1903649	regulation of cytoplasmic transport	6/1710	25/23210	0.00830723	0.03945597	0.0269514	6
GO:2000679	positive regulation of transcription regulatory region DNA binding	6/1710	25/23210	0.00830723	0.03945597	0.0269514	6
GO:2000737	negative regulation of stem cell differentiation	6/1710	25/23210	0.00830723	0.03945597	0.0269514	6
GO:0002228	natural killer cell mediated immunity	11/1710	66/23210	0.00840933	0.03987605	0.02723835	11
GO:0090278	negative regulation of peptide hormone secretion	11/1710	66/23210	0.00840933	0.03987605	0.02723835	11
GO:0045637	regulation of myeloid cell differentiation	26/1710	215/23210	0.00870117	0.04122647	0.02816079	26
GO:0007568	aging	29/1710	247/23210	0.00872036	0.04128391	0.02820002	29
GO:001	striated muscle adaptation	9/1710	49/232	0.00876	0.04144	0.02831	9

4888			10	932	851	246	
GO:190			49/232	0.00876	0.04144	0.02831	
4036	negative regulation of epithelial cell apoptotic process	9/1710	10	932	851	246	9
GO:004		30/171	258/23	0.00883	0.04170	0.02848	
5926	negative regulation of growth	0	210	131	779	956	30
GO:001		18/171	133/23	0.00887	0.04179	0.02854	
8210	peptidyl-threonine modification	0	210	97	42	859	18
GO:190		18/171	133/23	0.00887	0.04179	0.02854	
1568	fatty acid derivative metabolic process	0	210	97	42	859	18
GO:000		23/171	184/23	0.00893	0.04179	0.02854	
9746	response to hexose	0	210	437	42	859	23
GO:005		25/171	205/23	0.00898	0.04179	0.02854	
0770	regulation of axonogenesis	0	210	436	42	859	25
GO:000			12/232	0.00899	0.04179	0.02854	
2016	regulation of blood volume by renin-angiotensin	4/1710	10	269	42	859	4
GO:000			12/232	0.00899	0.04179	0.02854	
3091	renal water homeostasis	4/1710	10	269	42	859	4
GO:000			12/232	0.00899	0.04179	0.02854	
3183	mitral valve morphogenesis	4/1710	10	269	42	859	4
GO:000			12/232	0.00899	0.04179	0.02854	
3264	regulation of cardioblast proliferation	4/1710	10	269	42	859	4
GO:001			12/232	0.00899	0.04179	0.02854	
0889	regulation of sequestering of triglyceride	4/1710	10	269	42	859	4
GO:001			12/232	0.00899	0.04179	0.02854	
4842	regulation of skeletal muscle satellite cell proliferation	4/1710	10	269	42	859	4
GO:001	glycine transport	4/1710	12/232	0.00899	0.04179	0.02854	4

5816			10	269	42	859	
GO:003			12/232	0.00899	0.04179	0.02854	
0836	positive regulation of actin filament depolymerization	4/1710	10	269	42	859	4
GO:004			12/232	0.00899	0.04179	0.02854	
3649	dicarboxylic acid catabolic process	4/1710	10	269	42	859	4
GO:006			12/232	0.00899	0.04179	0.02854	
1450	trophoblast cell migration	4/1710	10	269	42	859	4
GO:007			12/232	0.00899	0.04179	0.02854	
0202	regulation of establishment of protein localization to chromosome	4/1710	10	269	42	859	4
GO:008			12/232	0.00899	0.04179	0.02854	
6013	membrane repolarization during cardiac muscle cell action potential	4/1710	10	269	42	859	4
GO:009			12/232	0.00899	0.04179	0.02854	
7428	protein maturation by iron-sulfur cluster transfer	4/1710	10	269	42	859	4
GO:190			12/232	0.00899	0.04179	0.02854	
2946	protein localization to early endosome	4/1710	10	269	42	859	4
GO:190			12/232	0.00899	0.04179	0.02854	
5668	positive regulation of protein localization to endosome	4/1710	10	269	42	859	4
GO:200			12/232	0.00899	0.04179	0.02854	
0674	regulation of type B pancreatic cell apoptotic process	4/1710	10	269	42	859	4
GO:004			33/232	0.00909	0.04210	0.02876	
5047	protein targeting to ER	7/1710	10	255	778	278	7
GO:006			33/232	0.00909	0.04210	0.02876	
0740	prostate gland epithelium morphogenesis	7/1710	10	255	778	278	7
GO:008			33/232	0.00909	0.04210	0.02876	
6091	regulation of heart rate by cardiac conduction	7/1710	10	255	778	278	7
GO:000	lens development in camera-type eye	13/171	85/232	0.00911	0.04210	0.02876	13

2088		0	10	824	778	278	
GO:000		22/171	174/23	0.00913	0.04210	0.02876	
6575	cellular modified amino acid metabolic process	0	210	877	778	278	22
GO:000			41/232	0.00913	0.04210	0.02876	
3298	physiological muscle hypertrophy	8/1710	10	945	778	278	8
GO:000			41/232	0.00913	0.04210	0.02876	
3301	physiological cardiac muscle hypertrophy	8/1710	10	945	778	278	8
GO:000			41/232	0.00913	0.04210	0.02876	
9066	aspartate family amino acid metabolic process	8/1710	10	945	778	278	8
GO:006			41/232	0.00913	0.04210	0.02876	
1049	cell growth involved in cardiac muscle cell development	8/1710	10	945	778	278	8
GO:190			41/232	0.00913	0.04210	0.02876	
0744	regulation of p38MAPK cascade	8/1710	10	945	778	278	8
GO:190			41/232	0.00913	0.04210	0.02876	
5521	regulation of macrophage migration	8/1710	10	945	778	278	8
GO:006		15/171	104/23	0.00915	0.04216	0.02880	
1326	renal tubule development	0	210	899	457	158	15
GO:000		26/171	216/23	0.00921	0.04240	0.02896	
7163	establishment or maintenance of cell polarity	0	210	925	854	823	26
GO:000		24/171	195/23	0.00925	0.04251	0.02903	
3205	cardiac chamber development	0	210	646	275	941	24
GO:003		24/171	195/23	0.00925	0.04251	0.02903	
0307	positive regulation of cell growth	0	210	646	275	941	24
GO:190		30/171	259/23	0.00930	0.04270	0.02917	
1990	regulation of mitotic cell cycle phase transition	0	210	551	446	036	30
GO:004	sensory system development	43/171	403/23	0.00933	0.04278	0.02922	43

8880		0	210	092	746	706	
GO:000		11/171	67/232	0.00940	0.04303	0.02939	
6801	superoxide metabolic process	0	10	886	896	886	11
GO:004		11/171	67/232	0.00940	0.04303	0.02939	
3113	receptor clustering	0	10	886	896	886	11
GO:006		11/171	67/232	0.00940	0.04303	0.02939	
0688	regulation of morphogenesis of a branching structure	0	10	886	896	886	11
GO:003		27/171	227/23	0.00941	0.04303	0.02939	
2944	regulation of mononuclear cell proliferation	0	210	523	896	886	27
GO:004		36/171	325/23	0.00945	0.04316	0.02948	
6486	glycerolipid metabolic process	0	210	116	943	797	36
GO:003		23/171	185/23	0.00951	0.04338	0.02963	
4284	response to monosaccharide	0	210	268	257	357	23
GO:005		23/171	185/23	0.00951	0.04338	0.02963	
0796	regulation of insulin secretion	0	210	268	257	357	23
GO:000		28/171	238/23	0.00957	0.04360	0.02978	
6401	RNA catabolic process	0	210	545	075	26	28
GO:007		28/171	238/23	0.00957	0.04360	0.02978	
0663	regulation of leukocyte proliferation	0	210	545	075	26	28
GO:004		22/171	175/23	0.00974	0.04434	0.03029	
3902	positive regulation of multi-organism process	0	210	725	847	335	22
GO:001		45/171	427/23	0.00985	0.04478	0.03059	
9932	second-messenger-mediated signaling	0	210	042	299	015	45
GO:001		15/171	105/23	0.00997	0.04525	0.03091	
6241	regulation of macroautophagy	0	210	845	942	559	15
GO:003	segmentation	15/171	105/23	0.00997	0.04525	0.03091	15

5282		0	210	845	942	559	
GO:005		15/171	105/23	0.00997	0.04525	0.03091	
5017	cardiac muscle tissue growth	0	210	845	942	559	15
GO:001			50/232	0.01001	0.04534	0.03097	
0596	negative regulation of endothelial cell migration	9/1710	10	253	358	308	9
GO:003			50/232	0.01001	0.04534	0.03097	
1663	lipopolysaccharide-mediated signaling pathway	9/1710	10	253	358	308	9
GO:006		13/171	86/232	0.01003	0.04542	0.03102	
1333	renal tubule morphogenesis	0	10	766	217	676	13
GO:002		46/171	439/23	0.01009	0.04563	0.03117	
2613	ribonucleoprotein complex biogenesis	0	210	207	302	079	46
GO:005			26/232	0.01013	0.04574	0.03124	
1125	regulation of actin nucleation	6/1710	10	939	077	439	6
GO:007			26/232	0.01013	0.04574	0.03124	
0129	regulation of mitochondrial translation	6/1710	10	939	077	439	6
GO:007			26/232	0.01013	0.04574	0.03124	
1549	cellular response to dexamethasone stimulus	6/1710	10	939	077	439	6
GO:190		19/171	145/23	0.01024	0.04617	0.03154	
5952	regulation of lipid localization	0	210	382	622	184	19
GO:005		30/171	261/23	0.01031	0.04646	0.03174	
1048	negative regulation of secretion	0	210	665	869	162	30
GO:006		12/171	77/232	0.01034	0.04656	0.03180	
1180	mammary gland epithelium development	0	10	504	064	442	12
GO:000		22/171	176/23	0.01038	0.04668	0.03188	
2262	myeloid cell homeostasis	0	210	804	223	748	22
GO:000	response to mechanical stimulus	22/171	176/23	0.01038	0.04668	0.03188	22

9612		0	210	804	223	748	
GO:000		10/171	59/232	0.01042	0.04672	0.03191	
6749	glutathione metabolic process	0	10	053	063	371	10
GO:190		10/171	59/232	0.01042	0.04672	0.03191	
3078	positive regulation of protein localization to plasma membrane	0	10	053	063	371	10
GO:000			19/232	0.01048	0.04672	0.03191	
9435	NAD biosynthetic process	5/1710	10	455	063	371	5
GO:001			19/232	0.01048	0.04672	0.03191	
0544	negative regulation of platelet activation	5/1710	10	455	063	371	5
GO:003			19/232	0.01048	0.04672	0.03191	
1571	mitotic G1 DNA damage checkpoint	5/1710	10	455	063	371	5
GO:004			19/232	0.01048	0.04672	0.03191	
4794	positive regulation by host of viral process	5/1710	10	455	063	371	5
GO:004			19/232	0.01048	0.04672	0.03191	
5953	negative regulation of natural killer cell mediated cytotoxicity	5/1710	10	455	063	371	5
GO:004			19/232	0.01048	0.04672	0.03191	
6886	positive regulation of hormone biosynthetic process	5/1710	10	455	063	371	5
GO:006	positive regulation of myeloid leukocyte cytokine production involved in immune		19/232	0.01048	0.04672	0.03191	
1081	response	5/1710	10	455	063	371	5
GO:190			19/232	0.01048	0.04672	0.03191	
4385	cellular response to angiotensin	5/1710	10	455	063	371	5
GO:200			19/232	0.01048	0.04672	0.03191	
0251	positive regulation of actin cytoskeleton reorganization	5/1710	10	455	063	371	5
GO:004		11/171	68/232	0.01049	0.04674	0.03192	
8002	antigen processing and presentation of peptide antigen	0	10	75	27	878	11
GO:007	cellular response to hypoxia	14/171	96/232	0.01053	0.04685	0.03200	14

1456		0	10	089	564	593	
GO:001			42/232	0.01057	0.04695	0.03207	
0883	regulation of lipid storage	8/1710	10	83	928	673	8
GO:004			42/232	0.01057	0.04695	0.03207	
5923	positive regulation of fatty acid metabolic process	8/1710	10	83	928	673	8
GO:009			42/232	0.01057	0.04695	0.03207	
8927	vesicle-mediated transport between endosomal compartments	8/1710	10	83	928	673	8
GO:004		21/171	166/23	0.01062	0.04714	0.03220	
2594	response to starvation	0	210	753	203	156	21
GO:002		42/171	395/23	0.01068	0.04733	0.03233	
2407	regulation of cell-cell adhesion	0	210	001	884	6	42
GO:000			34/232	0.01073	0.04748	0.03243	
6984	ER-nucleus signaling pathway	7/1710	10	804	799	788	7
GO:003			34/232	0.01073	0.04748	0.03243	
3032	regulation of myeloid cell apoptotic process	7/1710	10	804	799	788	7
GO:006			34/232	0.01073	0.04748	0.03243	
0512	prostate gland morphogenesis	7/1710	10	804	799	788	7
GO:005		23/171	187/23	0.01076	0.04755	0.03248	
0870	positive regulation of T cell activation	0	210	027	03	044	23
GO:004		33/171	295/23	0.01093	0.04829	0.03299	
3087	regulation of GTPase activity	0	210	735	63	001	33
GO:190		13/171	87/232	0.01102	0.04862	0.03321	
1879	regulation of protein depolymerization	0	10	776	202	251	13
GO:200		13/171	87/232	0.01102	0.04862	0.03321	
0117	negative regulation of cysteine-type endopeptidase activity	0	10	776	202	251	13
GO:009	import across plasma membrane	16/171	116/23	0.01103	0.04863	0.03321	16

8739		0	210	846	248	965	
GO:200		25/171	209/23	0.01133	0.04987	0.03406	
1020	regulation of response to DNA damage stimulus	0	210	615	722	99	25
GO:000		21/171	167/23	0.01133	0.04987	0.03406	
6399	tRNA metabolic process	0	210	806	722	99	21
GO:004			51/232	0.01138	0.04996	0.03413	
5912	negative regulation of carbohydrate metabolic process	9/1710	10	385	577	039	9
GO:005			51/232	0.01138	0.04996	0.03413	
1954	positive regulation of amine transport	9/1710	10	385	577	039	9
GO:007			51/232	0.01138	0.04996	0.03413	
0542	response to fatty acid	9/1710	10	385	577	039	9
GO:007		32/171	285/23	0.01147	0.05032	0.03437	
1496	cellular response to external stimulus	0	210	386	305	444	32
GO:007		14/171	97/232	0.01149	0.05034	0.03439	
2676	lymphocyte migration	0	10	711	941	244	14
GO:190		14/171	97/232	0.01149	0.05034	0.03439	
5037	autophagosome organization	0	10	711	941	244	14
GO:003		20/171	157/23	0.01157	0.05063	0.03458	
3135	regulation of peptidyl-serine phosphorylation	0	210	871	695	886	20
GO:000		42/171	397/23	0.01158	0.05063	0.03458	
1654	eye development	0	210	01	695	886	42
GO:004		10/171	60/232	0.01170	0.05109	0.03489	
5824	negative regulation of innate immune response	0	10	137	073	882	10
GO:006		10/171	60/232	0.01170	0.05109	0.03489	
0324	face development	0	10	137	073	882	10
GO:007	regulation of microtubule cytoskeleton organization	24/171	199/23	0.01174	0.05122	0.03499	24

0507		0	210	114	61	129	
GO:004		22/171	178/23	0.01177	0.05131	0.03505	
3524	negative regulation of neuron apoptotic process	0	210	13	934	498	22
GO:000		15/171	107/23	0.01179	0.05136	0.03508	
1823	mesonephros development	0	210	006	28	466	15
GO:001		17/171	127/23	0.01197	0.05207	0.03557	
0565	regulation of cellular ketone metabolic process	0	210	109	381	034	17
GO:001		17/171	127/23	0.01197	0.05207	0.03557	
0811	positive regulation of cell-substrate adhesion	0	210	109	381	034	17
GO:003		13/171	88/232	0.01209	0.05227	0.03571	
1110	regulation of microtubule polymerization or depolymerization	0	10	199	885	04	13
GO:004			43/232	0.01217	0.05227	0.03571	
3403	skeletal muscle tissue regeneration	8/1710	10	807	885	04	8
GO:000			27/232	0.01224	0.05227	0.03571	
3171	atrioventricular valve development	6/1710	10	105	885	04	6
GO:001			27/232	0.01224	0.05227	0.03571	
0893	positive regulation of steroid biosynthetic process	6/1710	10	105	885	04	6
GO:001			27/232	0.01224	0.05227	0.03571	
6577	histone demethylation	6/1710	10	105	885	04	6
GO:004			27/232	0.01224	0.05227	0.03571	
4342	type B pancreatic cell proliferation	6/1710	10	105	885	04	6
GO:007			27/232	0.01224	0.05227	0.03571	
0076	histone lysine demethylation	6/1710	10	105	885	04	6
GO:000			13/232	0.01224	0.05227	0.03571	
1553	luteinization	4/1710	10	195	885	04	4
GO:000	mitral valve development	4/1710	13/232	0.01224	0.05227	0.03571	4

3174			10	195	885	04	
GO:000			13/232	0.01224	0.05227	0.03571	
6983	ER overload response	4/1710	10	195	885	04	4
GO:000			13/232	0.01224	0.05227	0.03571	
7614	short-term memory	4/1710	10	195	885	04	4
GO:001			13/232	0.01224	0.05227	0.03571	
4841	skeletal muscle satellite cell proliferation	4/1710	10	195	885	04	4
GO:002			13/232	0.01224	0.05227	0.03571	
1683	cerebellar granular layer morphogenesis	4/1710	10	195	885	04	4
GO:003			13/232	0.01224	0.05227	0.03571	
0240	skeletal muscle thin filament assembly	4/1710	10	195	885	04	4
GO:003			13/232	0.01224	0.05227	0.03571	
2060	bleb assembly	4/1710	10	195	885	04	4
GO:004			13/232	0.01224	0.05227	0.03571	
2983	amyloid precursor protein biosynthetic process	4/1710	10	195	885	04	4
GO:004			13/232	0.01224	0.05227	0.03571	
2984	regulation of amyloid precursor protein biosynthetic process	4/1710	10	195	885	04	4
GO:004			13/232	0.01224	0.05227	0.03571	
5136	development of secondary sexual characteristics	4/1710	10	195	885	04	4
GO:004			13/232	0.01224	0.05227	0.03571	
5820	negative regulation of glycolytic process	4/1710	10	195	885	04	4
GO:004			13/232	0.01224	0.05227	0.03571	
8742	regulation of skeletal muscle fiber development	4/1710	10	195	885	04	4
GO:006			13/232	0.01224	0.05227	0.03571	
0452	positive regulation of cardiac muscle contraction	4/1710	10	195	885	04	4
GO:190	regulation of neuromuscular junction development	4/1710	13/232	0.01224	0.05227	0.03571	4

4396			10	195	885	04	
GO:190			13/232	0.01224	0.05227	0.03571	
4816	positive regulation of protein localization to chromosome, telomeric region	4/1710	10	195	885	04	4
GO:190			13/232	0.01224	0.05227	0.03571	
5048	regulation of metallopeptidase activity	4/1710	10	195	885	04	4
GO:190			13/232	0.01224	0.05227	0.03571	
5666	regulation of protein localization to endosome	4/1710	10	195	885	04	4
GO:000		27/171	232/23	0.01235	0.05274	0.03602	
1894	tissue homeostasis	0	210	984	375	795	27
GO:004		14/171	98/232	0.01253	0.05340	0.03648	
2102	positive regulation of T cell proliferation	0	10	099	896	234	14
GO:004		39/171	365/23	0.01253	0.05340	0.03648	
6879	hormone secretion	0	210	401	896	234	39
GO:003			35/232	0.01258	0.05341	0.03648	
4122	negative regulation of toll-like receptor signaling pathway	7/1710	10	932	422	594	7
GO:004			35/232	0.01258	0.05341	0.03648	
8147	negative regulation of fibroblast proliferation	7/1710	10	932	422	594	7
GO:004			35/232	0.01258	0.05341	0.03648	
8710	regulation of astrocyte differentiation	7/1710	10	932	422	594	7
GO:190			35/232	0.01258	0.05341	0.03648	
2895	positive regulation of pri-miRNA transcription by RNA polymerase II	7/1710	10	932	422	594	7
GO:003		12/171	79/232	0.01259	0.05341	0.03648	
8034	signal transduction in absence of ligand	0	10	925	422	594	12
GO:005		12/171	79/232	0.01259	0.05341	0.03648	
1702	interaction with symbiont	0	10	925	422	594	12
GO:009	extrinsic apoptotic signaling pathway in absence of ligand	12/171	79/232	0.01259	0.05341	0.03648	12

7192		0	10	925	422	594	
GO:190		32/171	287/23	0.01261	0.05345	0.03651	
3037	regulation of leukocyte cell-cell adhesion	0	210	914	977	705	32
GO:003		18/171	138/23	0.01278	0.05409	0.03695	
2946	positive regulation of mononuclear cell proliferation	0	210	208	391	022	18
GO:005		15/171	108/23	0.01278	0.05409	0.03695	
1028	mRNA transport	0	210	735	391	022	15
GO:004		23/171	190/23	0.01287	0.05435	0.03712	
4706	multi-multicellular organism process	0	210	56	671	973	23
GO:004			52/232	0.01289	0.05435	0.03712	
6148	pigment biosynthetic process	9/1710	10	083	671	973	9
GO:190			52/232	0.01289	0.05435	0.03712	
0271	regulation of long-term synaptic potentiation	9/1710	10	083	671	973	9
GO:006		16/171	118/23	0.01289	0.05435	0.03712	
0349	bone morphogenesis	0	210	6	671	973	16
GO:006		16/171	118/23	0.01289	0.05435	0.03712	
0759	regulation of response to cytokine stimulus	0	210	6	671	973	16
GO:190		11/171	70/232	0.01296	0.05451	0.03723	
1796	regulation of signal transduction by p53 class mediator	0	10	167	553	822	11
GO:199		11/171	70/232	0.01296	0.05451	0.03723	
0868	response to chemokine	0	10	167	553	822	11
GO:199		11/171	70/232	0.01296	0.05451	0.03723	
0869	cellular response to chemokine	0	10	167	553	822	11
GO:005		28/171	244/23	0.01313	0.05482	0.03744	
0663	cytokine secretion	0	210	657	267	802	28
GO:005	positive regulation of DNA-binding transcription factor activity	28/171	244/23	0.01313	0.05482	0.03744	28

1091		0	210	657	267	802	
GO:000			20/232	0.01314	0.05482	0.03744	
1946	lymphangiogenesis	5/1710	10	731	267	802	5
GO:000			20/232	0.01314	0.05482	0.03744	
2716	negative regulation of natural killer cell mediated immunity	5/1710	10	731	267	802	5
GO:003			20/232	0.01314	0.05482	0.03744	
2148	activation of protein kinase B activity	5/1710	10	731	267	802	5
GO:004			20/232	0.01314	0.05482	0.03744	
4783	G1 DNA damage checkpoint	5/1710	10	731	267	802	5
GO:004			20/232	0.01314	0.05482	0.03744	
4819	mitotic G1/S transition checkpoint	5/1710	10	731	267	802	5
GO:004			20/232	0.01314	0.05482	0.03744	
5649	regulation of macrophage differentiation	5/1710	10	731	267	802	5
GO:004			20/232	0.01314	0.05482	0.03744	
8143	astrocyte activation	5/1710	10	731	267	802	5
GO:007			20/232	0.01314	0.05482	0.03744	
1157	negative regulation of cell cycle arrest	5/1710	10	731	267	802	5
GO:008			20/232	0.01314	0.05482	0.03744	
0111	DNA demethylation	5/1710	10	731	267	802	5
GO:199			20/232	0.01314	0.05482	0.03744	
0776	response to angiotensin	5/1710	10	731	267	802	5
GO:190		13/171	89/232	0.01323	0.05514	0.03766	
3008	organelle disassembly	0	10	381	402	752	13
GO:004		50/171	493/23	0.01355	0.05645	0.03855	
8568	embryonic organ development	0	210	699	043	99	50
GO:003	cellular response to extracellular stimulus	26/171	223/23	0.01359	0.05655	0.03863	26

1668		0	210	158	413	073	
GO:000		21/171	170/23	0.01370	0.05696	0.03891	
7369	gastrulation	0	210	004	486	129	21
GO:007		49/171	482/23	0.01384	0.05748	0.03926	
1407	cellular response to organic cyclic compound	0	210	059	755	833	49
GO:001		15/171	109/23	0.01384	0.05748	0.03926	
5844	monoamine transport	0	210	91	755	833	15
GO:005		17/171	129/23	0.01385	0.05748	0.03926	
1101	regulation of DNA binding	0	210	527	755	833	17
GO:004			44/232	0.01394	0.05779	0.03947	
2181	ketone biosynthetic process	8/1710	10	853	242	658	8
GO:007			44/232	0.01394	0.05779	0.03947	
1385	cellular response to glucocorticoid stimulus	8/1710	10	853	242	658	8
GO:000		20/171	160/23	0.01407	0.05826	0.03979	
7565	female pregnancy	0	210	184	199	733	20
GO:000		13/171	90/232	0.01445	0.05972	0.04079	
6497	protein lipidation	0	10	671	851	907	13
GO:001		13/171	90/232	0.01445	0.05972	0.04079	
4031	mesenchymal cell development	0	10	671	851	907	13
GO:004		13/171	90/232	0.01445	0.05972	0.04079	
5833	negative regulation of lipid metabolic process	0	10	671	851	907	13
GO:003			53/232	0.01454	0.05988	0.04090	
1113	regulation of microtubule polymerization	9/1710	10	1	837	827	9
GO:004			53/232	0.01454	0.05988	0.04090	
6902	regulation of mitochondrial membrane permeability	9/1710	10	1	837	827	9
GO:003	cytoskeleton-dependent intracellular transport	21/171	171/23	0.01456	0.05988	0.04090	21

0705		0	210	867	837	827	
GO:004		21/171	171/23	0.01456	0.05988	0.04090	
3271	negative regulation of ion transport	0	210	867	837	827	21
GO:005		29/171	257/23	0.01457	0.05988	0.04090	
1403	stress-activated MAPK cascade	0	210	81	837	827	29
GO:001		10/171	62/232	0.01461	0.05988	0.04090	
0171	body morphogenesis	0	10	517	837	827	10
GO:001			28/232	0.01463	0.05988	0.04090	
0458	exit from mitosis	6/1710	10	077	837	827	6
GO:003			28/232	0.01463	0.05988	0.04090	
1063	regulation of histone deacetylation	6/1710	10	077	837	827	6
GO:004			28/232	0.01463	0.05988	0.04090	
4788	modulation by host of viral process	6/1710	10	077	837	827	6
GO:004			28/232	0.01463	0.05988	0.04090	
5822	negative regulation of heart contraction	6/1710	10	077	837	827	6
GO:008			28/232	0.01463	0.05988	0.04090	
6005	ventricular cardiac muscle cell action potential	6/1710	10	077	837	827	6
GO:009			28/232	0.01463	0.05988	0.04090	
0312	positive regulation of protein deacetylation	6/1710	10	077	837	827	6
GO:190			28/232	0.01463	0.05988	0.04090	
0745	positive regulation of p38MAPK cascade	6/1710	10	077	837	827	6
GO:003			36/232	0.01465	0.05988	0.04090	
3363	secretory granule organization	7/1710	10	943	837	827	7
GO:007			36/232	0.01465	0.05988	0.04090	
1398	cellular response to fatty acid	7/1710	10	943	837	827	7
GO:190	regulation of amyloid precursor protein catabolic process	7/1710	36/232	0.01465	0.05988	0.04090	7

2991			10	943	837	827	
GO:000		24/171	203/23	0.01473	0.06011	0.04106	
3018	vascular process in circulatory system	0	210	613	765	489	24
GO:190		24/171	203/23	0.01473	0.06011	0.04106	
3039	positive regulation of leukocyte cell-cell adhesion	0	210	613	765	489	24
GO:000		15/171	110/23	0.01497	0.06097	0.04165	
1776	leukocyte homeostasis	0	210	794	641	149	15
GO:001		15/171	110/23	0.01497	0.06097	0.04165	
9882	antigen processing and presentation	0	210	794	641	149	15
GO:190		15/171	110/23	0.01497	0.06097	0.04165	
1657	glycosyl compound metabolic process	0	210	794	641	149	15
GO:005		26/171	225/23	0.01510	0.06145	0.04198	
0670	regulation of lymphocyte proliferation	0	210	715	961	154	26
GO:000		21/171	172/23	0.01548	0.06293	0.04298	
6403	RNA localization	0	210	024	359	839	21
GO:004		13/171	91/232	0.01576	0.06404	0.04374	
5685	regulation of glial cell differentiation	0	10	418	336	644	13
GO:001		11/171	72/232	0.01584	0.06431	0.04392	
6575	histone deacetylation	0	10	107	102	927	11
GO:005		22/171	183/23	0.01587	0.06442	0.04400	
0792	regulation of viral process	0	210	984	363	619	22
GO:001			45/232	0.01589	0.06445	0.04402	
0464	regulation of mesenchymal cell proliferation	8/1710	10	926	764	943	8
GO:003		14/171	101/23	0.01606	0.06470	0.04420	
4446	substrate adhesion-dependent cell spreading	0	210	857	806	048	14
GO:005	regulation of DNA metabolic process	45/171	440/23	0.01611	0.06470	0.04420	45

1052		0	210	539	806	048	
GO:001			14/232	0.01615	0.06470	0.04420	
4819	regulation of skeletal muscle contraction	4/1710	10	533	806	048	4
GO:001			14/232	0.01615	0.06470	0.04420	
4866	skeletal myofibril assembly	4/1710	10	533	806	048	4
GO:003			14/232	0.01615	0.06470	0.04420	
0812	negative regulation of nucleotide catabolic process	4/1710	10	533	806	048	4
GO:003			14/232	0.01615	0.06470	0.04420	
5791	platelet-derived growth factor receptor-beta signaling pathway	4/1710	10	533	806	048	4
GO:004			14/232	0.01615	0.06470	0.04420	
2574	retinal metabolic process	4/1710	10	533	806	048	4
GO:004			14/232	0.01615	0.06470	0.04420	
5651	positive regulation of macrophage differentiation	4/1710	10	533	806	048	4
GO:004			14/232	0.01615	0.06470	0.04420	
6479	glycosphingolipid catabolic process	4/1710	10	533	806	048	4
GO:004			14/232	0.01615	0.06470	0.04420	
8711	positive regulation of astrocyte differentiation	4/1710	10	533	806	048	4
GO:005			14/232	0.01615	0.06470	0.04420	
1198	negative regulation of coenzyme metabolic process	4/1710	10	533	806	048	4
GO:006			14/232	0.01615	0.06470	0.04420	
1469	regulation of type B pancreatic cell proliferation	4/1710	10	533	806	048	4
GO:007			14/232	0.01615	0.06470	0.04420	
1340	skeletal muscle acetylcholine-gated channel clustering	4/1710	10	533	806	048	4
GO:009			14/232	0.01615	0.06470	0.04420	
8911	regulation of ventricular cardiac muscle cell action potential	4/1710	10	533	806	048	4
GO:190	positive regulation of TORC1 signaling	4/1710	14/232	0.01615	0.06470	0.04420	4

4263			10	533	806	048	
GO:000<br>6469	negative regulation of protein kinase activity	23/171<br>0	194/23<br>210	0.01619<br>896	0.06470<br>806	0.04420<br>048	23
GO:003<br>0308	negative regulation of cell growth	23/171<br>0	194/23<br>210	0.01619<br>896	0.06470<br>806	0.04420<br>048	23
GO:000<br>1976	neurological system process involved in regulation of systemic arterial blood pressure	5/1710	21/232<br>10	0.01623<br>103	0.06470<br>806	0.04420<br>048	5
GO:003<br>1065	positive regulation of histone deacetylation	5/1710	21/232<br>10	0.01623<br>103	0.06470<br>806	0.04420<br>048	5
GO:003<br>6303	lymph vessel morphogenesis	5/1710	21/232<br>10	0.01623<br>103	0.06470<br>806	0.04420<br>048	5
GO:009<br>0050	positive regulation of cell migration involved in sprouting angiogenesis	5/1710	21/232<br>10	0.01623<br>103	0.06470<br>806	0.04420<br>048	5
GO:190<br>1522	positive regulation of transcription from RNA polymerase II promoter involved in cellular response to chemical stimulus	5/1710	21/232<br>10	0.01623<br>103	0.06470<br>806	0.04420<br>048	5
GO:000<br>8652	cellular amino acid biosynthetic process	10/171<br>0	63/232<br>10	0.01626<br>009	0.06470<br>806	0.04420<br>048	10
GO:003<br>4121	regulation of toll-like receptor signaling pathway	10/171<br>0	63/232<br>10	0.01626<br>009	0.06470<br>806	0.04420<br>048	10
GO:004<br>2246	tissue regeneration	10/171<br>0	63/232<br>10	0.01626<br>009	0.06470<br>806	0.04420<br>048	10
GO:004<br>3388	positive regulation of DNA binding	10/171<br>0	63/232<br>10	0.01626<br>009	0.06470<br>806	0.04420<br>048	10
GO:007<br>0059	intrinsic apoptotic signaling pathway in response to endoplasmic reticulum stress	10/171<br>0	63/232<br>10	0.01626<br>009	0.06470<br>806	0.04420<br>048	10
GO:003	regulation of hormone metabolic process	9/1710	54/232	0.01634	0.06485	0.04430	9

2350			10	176	636	178	
GO:003			54/232	0.01634	0.06485	0.04430	
5272	exocrine system development	9/1710	10	176	636	178	9
GO:004			54/232	0.01634	0.06485	0.04430	
5687	positive regulation of glial cell differentiation	9/1710	10	176	636	178	9
GO:004			54/232	0.01634	0.06485	0.04430	
6718	viral entry into host cell	9/1710	10	176	636	178	9
GO:190		12/171	82/232	0.01666	0.06608	0.04513	
3317	regulation of protein maturation	0	10	182	171	879	12
GO:000		41/171	395/23	0.01672	0.06630	0.04529	
9314	response to radiation	0	210	947	501	132	41
GO:004		29/171	260/23	0.01684	0.06673	0.04558	
8588	developmental cell growth	0	210	856	173	28	29
GO:001			37/232	0.01696	0.06689	0.04569	
5695	organic cation transport	7/1710	10	101	754	606	7
GO:003			37/232	0.01696	0.06689	0.04569	
3028	myeloid cell apoptotic process	7/1710	10	101	754	606	7
GO:005			37/232	0.01696	0.06689	0.04569	
1647	nucleus localization	7/1710	10	101	754	606	7
GO:006			37/232	0.01696	0.06689	0.04569	
0071	Wnt signaling pathway, planar cell polarity pathway	7/1710	10	101	754	606	7
GO:007			37/232	0.01696	0.06689	0.04569	
1158	positive regulation of cell cycle arrest	7/1710	10	101	754	606	7
GO:007			37/232	0.01696	0.06689	0.04569	
2599	establishment of protein localization to endoplasmic reticulum	7/1710	10	101	754	606	7
GO:003	mammary gland development	20/171	163/23	0.01697	0.06689	0.04569	20

0879		0	210	058	754	606	
GO:000			29/232	0.01732	0.06806	0.04649	
6734	NADH metabolic process	6/1710	10	616	956	664	6
GO:003			29/232	0.01732	0.06806	0.04649	
4629	cellular protein-containing complex localization	6/1710	10	616	956	664	6
GO:004			29/232	0.01732	0.06806	0.04649	
3090	amino acid import	6/1710	10	616	956	664	6
GO:006			29/232	0.01732	0.06806	0.04649	
0914	heart formation	6/1710	10	616	956	664	6
GO:007			29/232	0.01732	0.06806	0.04649	
1548	response to dexamethasone	6/1710	10	616	956	664	6
GO:004		14/171	102/23	0.01740	0.06832	0.04667	
6427	positive regulation of JAK-STAT cascade	0	210	31	588	173	14
GO:004		15/171	112/23	0.01744	0.06841	0.04673	
3123	positive regulation of I-kappaB kinase/NF-kappaB signaling	0	210	739	298	122	15
GO:004		11/171	73/232	0.01744	0.06841	0.04673	
5670	regulation of osteoclast differentiation	0	10	871	298	122	11
GO:001		31/171	283/23	0.01750	0.06857	0.04684	
6032	viral process	0	210	248	779	38	31
GO:000		22/171	185/23	0.01780	0.06973	0.04763	
1764	neuron migration	0	210	973	487	417	22
GO:000		18/171	143/23	0.01792	0.07014	0.04791	
9267	cellular response to starvation	0	210	528	032	112	18
GO:003		20/171	164/23	0.01803	0.07035	0.04805	
0902	hindbrain development	0	210	404	204	574	20
GO:001	positive regulation of cellular carbohydrate metabolic process	10/171	64/232	0.01803	0.07035	0.04805	10

0676		0	10	821	204	574	
GO:001			46/232	0.01803	0.07035	0.04805	
0677	negative regulation of cellular carbohydrate metabolic process	8/1710	10	96	204	574	8
GO:004			46/232	0.01803	0.07035	0.04805	
3029	T cell homeostasis	8/1710	10	96	204	574	8
GO:007			46/232	0.01803	0.07035	0.04805	
1384	cellular response to corticosteroid stimulus	8/1710	10	96	204	574	8
GO:190		12/171	83/232	0.01821	0.07098	0.04848	
3363	negative regulation of cellular protein catabolic process	0	10	405	496	808	12
GO:000		31/171	284/23	0.01830	0.07127	0.04868	
7162	negative regulation of cell adhesion	0	210	733	541	648	31
GO:003		17/171	133/23	0.01831	0.07127	0.04868	
5966	response to topologically incorrect protein	0	210	297	541	648	17
GO:003		40/171	386/23	0.01837	0.07146	0.04881	
0900	forebrain development	0	210	277	053	293	40
GO:003		47/171	467/23	0.01843	0.07165	0.04894	
4660	ncRNA metabolic process	0	210	569	754	75	47
GO:003		21/171	175/23	0.01848	0.07175	0.04901	
5107	appendage morphogenesis	0	210	643	929	701	21
GO:003		21/171	175/23	0.01848	0.07175	0.04901	
5108	limb morphogenesis	0	210	643	929	701	21
GO:000		13/171	93/232	0.01864	0.07233	0.04940	
2027	regulation of heart rate	0	10	675	355	927	13
GO:000		15/171	113/23	0.01879	0.07285	0.04976	
1952	regulation of cell-matrix adhesion	0	210	326	35	443	15
GO:005	protein homotetramerization	14/171	103/23	0.01882	0.07290	0.04980	14

1289		0	210	031	997	301	
GO:190		23/171	197/23	0.01910	0.07398	0.05053	
2275	regulation of chromatin organization	0	210	928	039	418	23
GO:001		11/171	74/232	0.01917	0.07418	0.05067	
0822	positive regulation of mitochondrion organization	0	10	473	461	368	11
GO:000		34/171	319/23	0.01936	0.07487	0.05114	
7159	leukocyte cell-cell adhesion	0	210	513	166	299	34
GO:004			38/232	0.01950	0.07531	0.05144	
0019	positive regulation of embryonic development	7/1710	10	617	729	739	7
GO:190			38/232	0.01950	0.07531	0.05144	
0026	positive regulation of substrate adhesion-dependent cell spreading	7/1710	10	617	729	739	7
GO:000		17/171	134/23	0.01958	0.07556	0.05161	
2698	negative regulation of immune effector process	0	210	307	425	608	17
GO:000			22/232	0.01976	0.07564	0.05167	
2183	cytoplasmic translational initiation	5/1710	10	026	838	354	5
GO:000			22/232	0.01976	0.07564	0.05167	
9067	aspartate family amino acid biosynthetic process	5/1710	10	026	838	354	5
GO:001			22/232	0.01976	0.07564	0.05167	
5874	norepinephrine transport	5/1710	10	026	838	354	5
GO:003			22/232	0.01976	0.07564	0.05167	
2042	mitochondrial DNA metabolic process	5/1710	10	026	838	354	5
GO:003			22/232	0.01976	0.07564	0.05167	
2691	negative regulation of interleukin-1 beta production	5/1710	10	026	838	354	5
GO:003			22/232	0.01976	0.07564	0.05167	
5268	protein mannosylation	5/1710	10	026	838	354	5
GO:003	DNA dealkylation	5/1710	22/232	0.01976	0.07564	0.05167	5

5510			10	026	838	354	
GO:005			22/232	0.01976	0.07564	0.05167	
1654	establishment of mitochondrion localization	5/1710	10	026	838	354	5
GO:006			22/232	0.01976	0.07564	0.05167	
0749	mammary gland alveolus development	5/1710	10	026	838	354	5
GO:006			22/232	0.01976	0.07564	0.05167	
1377	mammary gland lobule development	5/1710	10	026	838	354	5
GO:009			22/232	0.01976	0.07564	0.05167	
0382	phagosome maturation	5/1710	10	026	838	354	5
GO:009			22/232	0.01976	0.07564	0.05167	
7062	dendritic spine maintenance	5/1710	10	026	838	354	5
GO:007		35/171	331/23	0.01991	0.07610	0.05198	
0661	leukocyte proliferation	0	210	122	494	541	35
GO:000		22/171	187/23	0.01991	0.07610	0.05198	
6473	protein acetylation	0	210	86	494	541	22
GO:003		22/171	187/23	0.01991	0.07610	0.05198	
0534	adult behavior	0	210	86	494	541	22
GO:004		10/171	65/232	0.01995	0.07619	0.05204	
2440	pigment metabolic process	0	10	533	546	724	10
GO:001		49/171	493/23	0.02014	0.07684	0.05249	
0035	response to inorganic substance	0	210	782	631	182	49
GO:000		23/171	198/23	0.02016	0.07684	0.05249	
9743	response to carbohydrate	0	210	525	631	182	23
GO:004		23/171	198/23	0.02016	0.07684	0.05249	
3903	regulation of symbiosis, encompassing mutualism through parasitism	0	210	525	631	182	23
GO:000	ureteric bud development	14/171	104/23	0.02032	0.07692	0.05254	14

1657		0	210	318	412	497	
GO:007		14/171	104/23	0.02032	0.07692	0.05254	
2163	mesonephric epithelium development	0	210	318	412	497	14
GO:007		14/171	104/23	0.02032	0.07692	0.05254	
2164	mesonephric tubule development	0	210	318	412	497	14
GO:001		24/171	209/23	0.02033	0.07692	0.05254	
9722	calcium-mediated signaling	0	210	408	412	497	24
GO:000			30/232	0.02034	0.07692	0.05254	
6482	protein demethylation	6/1710	10	368	412	497	6
GO:000			30/232	0.02034	0.07692	0.05254	
8214	protein dealkylation	6/1710	10	368	412	497	6
GO:001			30/232	0.02034	0.07692	0.05254	
0043	response to zinc ion	6/1710	10	368	412	497	6
GO:003			30/232	0.02034	0.07692	0.05254	
4260	negative regulation of GTPase activity	6/1710	10	368	412	497	6
GO:004			30/232	0.02034	0.07692	0.05254	
4062	regulation of excretion	6/1710	10	368	412	497	6
GO:005			30/232	0.02034	0.07692	0.05254	
5022	negative regulation of cardiac muscle tissue growth	6/1710	10	368	412	497	6
GO:006			30/232	0.02034	0.07692	0.05254	
1117	negative regulation of heart growth	6/1710	10	368	412	497	6
GO:190			30/232	0.02034	0.07692	0.05254	
2253	regulation of intrinsic apoptotic signaling pathway by p53 class mediator	6/1710	10	368	412	497	6
GO:007		18/171	145/23	0.02038	0.07701	0.05260	
0665	positive regulation of leukocyte proliferation	0	210	152	736	866	18
GO:000	excretion	9/1710	56/232	0.02042	0.07702	0.05261	9

7588			10	408	872	642	
GO:003			56/232	0.02042	0.07702	0.05261	
5567	non-canonical Wnt signaling pathway	9/1710	10	408	872	642	9
GO:004			56/232	0.02042	0.07702	0.05261	
2743	hydrogen peroxide metabolic process	9/1710	10	408	872	642	9
GO:001		27/171	242/23	0.02046	0.07707	0.05264	
0466	negative regulation of peptidase activity	0	210	217	03	482	27
GO:000		26/171	231/23	0.02047	0.07707	0.05264	
9952	anterior/posterior pattern specification	0	210	468	03	482	26
GO:004		26/171	231/23	0.02047	0.07707	0.05264	
3543	protein acylation	0	210	468	03	482	26
GO:000			15/232	0.02076	0.07723	0.05275	
6704	glucocorticoid biosynthetic process	4/1710	10	968	52	746	4
GO:000	DNA damage response, signal transduction by p53 class mediator resulting in		15/232	0.02076	0.07723	0.05275	
6978	transcription of p21 class mediator	4/1710	10	968	52	746	4
GO:000			15/232	0.02076	0.07723	0.05275	
9083	branched-chain amino acid catabolic process	4/1710	10	968	52	746	4
GO:001			15/232	0.02076	0.07723	0.05275	
0172	embryonic body morphogenesis	4/1710	10	968	52	746	4
GO:001			15/232	0.02076	0.07723	0.05275	
0523	negative regulation of calcium ion transport into cytosol	4/1710	10	968	52	746	4
GO:001			15/232	0.02076	0.07723	0.05275	
0715	regulation of extracellular matrix disassembly	4/1710	10	968	52	746	4
GO:001			15/232	0.02076	0.07723	0.05275	
4857	regulation of skeletal muscle cell proliferation	4/1710	10	968	52	746	4
GO:001	glycolipid catabolic process	4/1710	15/232	0.02076	0.07723	0.05275	4

9377			10	968	52	746	
GO:003			15/232	0.02076	0.07723	0.05275	
9535	regulation of RIG-I signaling pathway	4/1710	10	968	52	746	4
GO:004			15/232	0.02076	0.07723	0.05275	
3129	surfactant homeostasis	4/1710	10	968	52	746	4
GO:005			15/232	0.02076	0.07723	0.05275	
0930	induction of positive chemotaxis	4/1710	10	968	52	746	4
GO:005			15/232	0.02076	0.07723	0.05275	
5089	fatty acid homeostasis	4/1710	10	968	52	746	4
GO:006			15/232	0.02076	0.07723	0.05275	
1430	bone trabecula morphogenesis	4/1710	10	968	52	746	4
GO:007			15/232	0.02076	0.07723	0.05275	
2567	chemokine (C-X-C motif) ligand 2 production	4/1710	10	968	52	746	4
GO:008			15/232	0.02076	0.07723	0.05275	
6014	atrial cardiac muscle cell action potential	4/1710	10	968	52	746	4
GO:008			15/232	0.02076	0.07723	0.05275	
6026	atrial cardiac muscle cell to AV node cell signaling	4/1710	10	968	52	746	4
GO:008			15/232	0.02076	0.07723	0.05275	
6066	atrial cardiac muscle cell to AV node cell communication	4/1710	10	968	52	746	4
GO:009			15/232	0.02076	0.07723	0.05275	
7050	type B pancreatic cell apoptotic process	4/1710	10	968	52	746	4
GO:190			15/232	0.02076	0.07723	0.05275	
4814	regulation of protein localization to chromosome, telomeric region	4/1710	10	968	52	746	4
GO:000		11/171	75/232	0.02102	0.07798	0.05326	
8625	extrinsic apoptotic signaling pathway via death domain receptors	0	10	388	193	753	11
GO:004	hormone biosynthetic process	11/171	75/232	0.02102	0.07798	0.05326	11

2446		0	10	388	193	753	
GO:006		11/171	75/232	0.02102	0.07798	0.05326	
1097	regulation of protein tyrosine kinase activity	0	10	388	193	753	11
GO:007		11/171	75/232	0.02102	0.07798	0.05326	
0373	negative regulation of ERK1 and ERK2 cascade	0	10	388	193	753	11
GO:001		23/171	199/23	0.02126	0.07883	0.05384	
9058	viral life cycle	0	210	613	041	711	23
GO:190		26/171	232/23	0.02149	0.07964	0.05440	
3531	negative regulation of secretion by cell	0	210	889	267	195	26
GO:007		20/171	167/23	0.02153	0.07973	0.05446	
1897	DNA biosynthetic process	0	210	739	475	484	20
GO:005		12/171	85/232	0.02163	0.08006	0.05468	
1384	response to glucocorticoid	0	10	979	309	913	12
GO:004		15/171	115/23	0.02172	0.08027	0.05483	
3244	regulation of protein complex disassembly	0	210	037	519	4	15
GO:004		37/171	356/23	0.02172	0.08027	0.05483	
4772	mitotic cell cycle phase transition	0	210	459	519	4	37
GO:005		14/171	105/23	0.02191	0.08092	0.05527	
0764	regulation of phagocytosis	0	210	468	638	882	14
GO:006		21/171	178/23	0.02192	0.08092	0.05528	
1136	regulation of proteasomal protein catabolic process	0	210	913	86	034	21
GO:003		10/171	66/232	0.02201	0.08115	0.05543	
4394	protein localization to cell surface	0	10	712	079	211	10
GO:190		10/171	66/232	0.02201	0.08115	0.05543	
4377	positive regulation of protein localization to cell periphery	0	10	712	079	211	10
GO:001	peptidyl-lysine modification	36/171	345/23	0.02209	0.08139	0.05560	36

8205		0	210	815	81	103	
GO:003			39/232	0.02230	0.08181	0.05588	
2008	positive regulation of TOR signaling	7/1710	10	643	916	865	7
GO:009			39/232	0.02230	0.08181	0.05588	
0175	regulation of establishment of planar polarity	7/1710	10	643	916	865	7
GO:009			39/232	0.02230	0.08181	0.05588	
7009	energy homeostasis	7/1710	10	643	916	865	7
GO:190			39/232	0.02230	0.08181	0.05588	
1021	positive regulation of calcium ion transmembrane transporter activity	7/1710	10	643	916	865	7
GO:001		17/171	136/23	0.02232	0.08181	0.05588	
7148	negative regulation of translation	0	210	45	916	865	17
GO:004		17/171	136/23	0.02232	0.08181	0.05588	
3624	cellular protein complex disassembly	0	210	45	916	865	17
GO:005		17/171	136/23	0.02232	0.08181	0.05588	
0671	positive regulation of lymphocyte proliferation	0	210	45	916	865	17
GO:200		17/171	136/23	0.02232	0.08181	0.05588	
1056	positive regulation of cysteine-type endopeptidase activity	0	210	45	916	865	17
GO:000			57/232	0.02271	0.08311	0.05677	
1736	establishment of planar polarity	9/1710	10	968	105	111	9
GO:000			57/232	0.02271	0.08311	0.05677	
9247	glycolipid biosynthetic process	9/1710	10	968	105	111	9
GO:004			57/232	0.02271	0.08311	0.05677	
2551	neuron maturation	9/1710	10	968	105	111	9
GO:190		20/171	168/23	0.02281	0.08340	0.05697	
2107	positive regulation of leukocyte differentiation	0	210	459	604	261	20
GO:003	adherens junction organization	16/171	126/23	0.02286	0.08354	0.05706	16

4332		0	210	933	831	98	
GO:001			48/232	0.02292	0.08354	0.05706	
0107	potassium ion import	8/1710	10	502	831	98	8
GO:004			48/232	0.02292	0.08354	0.05706	
7496	vesicle transport along microtubule	8/1710	10	502	831	98	8
GO:005			48/232	0.02292	0.08354	0.05706	
0850	positive regulation of calcium-mediated signaling	8/1710	10	502	831	98	8
GO:190			48/232	0.02292	0.08354	0.05706	
4591	positive regulation of protein import	8/1710	10	502	831	98	8
GO:004		11/171	76/232	0.02300	0.08372	0.05718	
8844	artery morphogenesis	0	10	084	017	719	11
GO:006		11/171	76/232	0.02300	0.08372	0.05718	
0350	endochondral bone morphogenesis	0	10	084	017	719	11
GO:001		18/171	147/23	0.02308	0.08398	0.05736	
6482	cytosolic transport	0	210	8	51	815	18
GO:005		15/171	116/23	0.02330	0.08472	0.05787	
1261	protein depolymerization	0	210	679	822	576	15
GO:007		22/171	190/23	0.02343	0.08515	0.05817	
1695	anatomical structure maturation	0	210	997	938	028	22
GO:001		12/171	86/232	0.02352	0.08540	0.05833	
5908	fatty acid transport	0	10	121	144	562	12
GO:190		14/171	106/23	0.02359	0.08540	0.05833	
4894	positive regulation of STAT cascade	0	210	773	675	925	14
GO:000		19/171	158/23	0.02368	0.08540	0.05833	
2244	hematopoietic progenitor cell differentiation	0	210	932	675	925	19
GO:005	negative regulation of immune response	19/171	158/23	0.02368	0.08540	0.05833	19

0777		0	210	932	675	925	
GO:000			31/232	0.02369	0.08540	0.05833	
1832	blastocyst growth	6/1710	10	851	675	925	6
GO:003			31/232	0.02369	0.08540	0.05833	
2967	positive regulation of collagen biosynthetic process	6/1710	10	851	675	925	6
GO:005			31/232	0.02369	0.08540	0.05833	
1491	positive regulation of filopodium assembly	6/1710	10	851	675	925	6
GO:190			31/232	0.02369	0.08540	0.05833	
3393	positive regulation of adherens junction organization	6/1710	10	851	675	925	6
GO:002		25/171	223/23	0.02371	0.08540	0.05833	
2618	ribonucleoprotein complex assembly	0	210	104	675	925	25
GO:000			23/232	0.02375	0.08540	0.05833	
1911	negative regulation of leukocyte mediated cytotoxicity	5/1710	10	659	675	925	5
GO:000			23/232	0.02375	0.08540	0.05833	
2320	lymphoid progenitor cell differentiation	5/1710	10	659	675	925	5
GO:000			23/232	0.02375	0.08540	0.05833	
6837	serotonin transport	5/1710	10	659	675	925	5
GO:000			23/232	0.02375	0.08540	0.05833	
7020	microtubule nucleation	5/1710	10	659	675	925	5
GO:003			23/232	0.02375	0.08540	0.05833	
9528	cytoplasmic pattern recognition receptor signaling pathway in response to virus	5/1710	10	659	675	925	5
GO:004			23/232	0.02375	0.08540	0.05833	
8333	mesodermal cell differentiation	5/1710	10	659	675	925	5
GO:009			23/232	0.02375	0.08540	0.05833	
0190	positive regulation of branching involved in ureteric bud morphogenesis	5/1710	10	659	675	925	5
GO:009	mannosylation	5/1710	23/232	0.02375	0.08540	0.05833	5

7502			10	659	675	925	
GO:005		10/171	67/232	0.02422	0.08705	0.05946	
0710	negative regulation of cytokine secretion	0	10	909	188	3	10
GO:003		37/171	359/23	0.02435	0.08745	0.05974	
2496	response to lipopolysaccharide	0	210	712	81	047	37
GO:005		18/171	148/23	0.02453	0.08800	0.06011	
0657	nucleic acid transport	0	210	968	539	432	18
GO:005		18/171	148/23	0.02453	0.08800	0.06011	
0658	RNA transport	0	210	968	539	432	18
GO:000		15/171	117/23	0.02497	0.08936	0.06104	
7498	mesoderm development	0	210	854	406	239	15
GO:000		33/171	314/23	0.02510	0.08936	0.06104	
6066	alcohol metabolic process	0	210	926	406	239	33
GO:001		11/171	77/232	0.02511	0.08936	0.06104	
4855	striated muscle cell proliferation	0	10	013	406	239	11
GO:007		11/171	77/232	0.02511	0.08936	0.06104	
1347	cellular response to interleukin-1	0	10	013	406	239	11
GO:007		11/171	77/232	0.02511	0.08936	0.06104	
2332	intrinsic apoptotic signaling pathway by p53 class mediator	0	10	013	406	239	11
GO:190		11/171	77/232	0.02511	0.08936	0.06104	
5897	regulation of response to endoplasmic reticulum stress	0	10	013	406	239	11
GO:000			58/232	0.02519	0.08936	0.06104	
7164	establishment of tissue polarity	9/1710	10	387	406	239	9
GO:001			58/232	0.02519	0.08936	0.06104	
5909	long-chain fatty acid transport	9/1710	10	387	406	239	9
GO:003	entry into host cell	9/1710	58/232	0.02519	0.08936	0.06104	9

0260			10	387	406	239	
GO:003			58/232	0.02519	0.08936	0.06104	
2835	glomerulus development	9/1710	10	387	406	239	9
GO:004			58/232	0.02519	0.08936	0.06104	
3954	cellular component maintenance	9/1710	10	387	406	239	9
GO:004			58/232	0.02519	0.08936	0.06104	
4409	entry into host	9/1710	10	387	406	239	9
GO:005			58/232	0.02519	0.08936	0.06104	
1187	cofactor catabolic process	9/1710	10	387	406	239	9
GO:005			58/232	0.02519	0.08936	0.06104	
1806	entry into cell of other organism involved in symbiotic interaction	9/1710	10	387	406	239	9
GO:005			58/232	0.02519	0.08936	0.06104	
1828	entry into other organism involved in symbiotic interaction	9/1710	10	387	406	239	9
GO:007			58/232	0.02519	0.08936	0.06104	
2678	T cell migration	9/1710	10	387	406	239	9
GO:009			58/232	0.02519	0.08936	0.06104	
8900	regulation of action potential	9/1710	10	387	406	239	9
GO:009			58/232	0.02519	0.08936	0.06104	
8930	axonal transport	9/1710	10	387	406	239	9
GO:000			40/232	0.02537	0.08946	0.06111	
1881	receptor recycling	7/1710	10	26	414	075	7
GO:000			40/232	0.02537	0.08946	0.06111	
2686	negative regulation of leukocyte migration	7/1710	10	26	414	075	7
GO:000			40/232	0.02537	0.08946	0.06111	
3009	skeletal muscle contraction	7/1710	10	26	414	075	7
GO:004	multi-organism cellular process	7/1710	40/232	0.02537	0.08946	0.06111	7

4764			10	26	414	075	
GO:007			40/232	0.02537	0.08946	0.06111	
0884	regulation of calcineurin-NFAT signaling cascade	7/1710	10	26	414	075	7
GO:009			40/232	0.02537	0.08946	0.06111	
8801	regulation of renal system process	7/1710	10	26	414	075	7
GO:010			40/232	0.02537	0.08946	0.06111	
6056	regulation of calcineurin-mediated signaling	7/1710	10	26	414	075	7
GO:011			40/232	0.02537	0.08946	0.06111	
0111	negative regulation of animal organ morphogenesis	7/1710	10	26	414	075	7
GO:190			40/232	0.02537	0.08946	0.06111	
5332	positive regulation of morphogenesis of an epithelium	7/1710	10	26	414	075	7
GO:000		14/171	107/23	0.02537	0.08946	0.06111	
7043	cell-cell junction assembly	0	210	523	414	075	14
GO:000		12/171	87/232	0.02552	0.08992	0.06142	
2690	positive regulation of leukocyte chemotaxis	0	10	016	086	273	12
GO:000		27/171	247/23	0.02583	0.09069	0.06195	
2064	epithelial cell development	0	210	773	824	373	27
GO:001		21/171	181/23	0.02584	0.09069	0.06195	
5931	nucleobase-containing compound transport	0	210	601	824	373	21
GO:003		31/171	292/23	0.02585	0.09069	0.06195	
0111	regulation of Wnt signaling pathway	0	210	433	824	373	31
GO:007		26/171	236/23	0.02600	0.09069	0.06195	
1826	ribonucleoprotein complex subunit organization	0	210	091	824	373	26
GO:000		18/171	149/23	0.02605	0.09069	0.06195	
1824	blastocyst development	0	210	941	824	373	18
GO:000	macrophage activation involved in immune response	4/1710	16/232	0.02611	0.09069	0.06195	4

2281			10	339	824	373	
GO:000			16/232	0.02611	0.09069	0.06195	
9081	branched-chain amino acid metabolic process	4/1710	10	339	824	373	4
GO:000			16/232	0.02611	0.09069	0.06195	
9147	pyrimidine nucleoside triphosphate metabolic process	4/1710	10	339	824	373	4
GO:001			16/232	0.02611	0.09069	0.06195	
4067	negative regulation of phosphatidylinositol 3-kinase signaling	4/1710	10	339	824	373	4
GO:001			16/232	0.02611	0.09069	0.06195	
4733	regulation of skeletal muscle adaptation	4/1710	10	339	824	373	4
GO:001			16/232	0.02611	0.09069	0.06195	
4856	skeletal muscle cell proliferation	4/1710	10	339	824	373	4
GO:004			16/232	0.02611	0.09069	0.06195	
2228	interleukin-8 biosynthetic process	4/1710	10	339	824	373	4
GO:004			16/232	0.02611	0.09069	0.06195	
2772	DNA damage response, signal transduction resulting in transcription	4/1710	10	339	824	373	4
GO:005			16/232	0.02611	0.09069	0.06195	
1386	regulation of neurotrophin TRK receptor signaling pathway	4/1710	10	339	824	373	4
GO:006			16/232	0.02611	0.09069	0.06195	
0100	positive regulation of phagocytosis, engulfment	4/1710	10	339	824	373	4
GO:006			16/232	0.02611	0.09069	0.06195	
0314	regulation of ryanodine-sensitive calcium-release channel activity	4/1710	10	339	824	373	4
GO:007			16/232	0.02611	0.09069	0.06195	
0200	establishment of protein localization to telomere	4/1710	10	339	824	373	4
GO:007			16/232	0.02611	0.09069	0.06195	
1379	cellular response to prostaglandin stimulus	4/1710	10	339	824	373	4
GO:009	regulation of brown fat cell differentiation	4/1710	16/232	0.02611	0.09069	0.06195	4

0335			10	339	824	373	
GO:009	regulation of neurotransmitter receptor localization to postsynaptic specialization		16/232	0.02611	0.09069	0.06195	
8696	membrane	4/1710	10	339	824	373	4
GO:190			16/232	0.02611	0.09069	0.06195	
0119	positive regulation of execution phase of apoptosis	4/1710	10	339	824	373	4
GO:190			16/232	0.02611	0.09069	0.06195	
1881	positive regulation of protein depolymerization	4/1710	10	339	824	373	4
GO:190			16/232	0.02611	0.09069	0.06195	
5155	positive regulation of membrane invagination	4/1710	10	339	824	373	4
GO:190			16/232	0.02611	0.09069	0.06195	
5564	positive regulation of vascular endothelial cell proliferation	4/1710	10	339	824	373	4
GO:000		35/171	338/23	0.02625	0.09114	0.06225	
9100	glycoprotein metabolic process	0	210	701	287	745	35
GO:000		29/171	270/23	0.02650	0.09195	0.06281	
9615	response to virus	0	210	804	96	534	29
GO:005		10/171	68/232	0.02659	0.09215	0.06295	
5081	anion homeostasis	0	10	661	741	046	10
GO:200		10/171	68/232	0.02659	0.09215	0.06295	
1244	positive regulation of intrinsic apoptotic signaling pathway	0	10	661	741	046	10
GO:000		38/171	373/23	0.02668	0.09239	0.06311	
3002	regionalization	0	210	137	629	363	38
GO:004		41/171	408/23	0.02683	0.09287	0.06344	
4419	interspecies interaction between organisms	0	210	537	454	031	41
GO:000		42/171	420/23	0.02714	0.09388	0.06413	
2697	regulation of immune effector process	0	210	34	5	053	42
GO:000	regulation of cytokine-mediated signaling pathway	14/171	108/23	0.02725	0.09417	0.06432	14

1959		0	210	002	456	832	
GO:000		11/171	78/232	0.02735	0.09417	0.06432	
2260	lymphocyte homeostasis	0	10	622	456	832	11
GO:000			32/232	0.02740	0.09417	0.06432	
3016	respiratory system process	6/1710	10	444	456	832	6
GO:000			32/232	0.02740	0.09417	0.06432	
7097	nuclear migration	6/1710	10	444	456	832	6
GO:004			32/232	0.02740	0.09417	0.06432	
3243	positive regulation of protein complex disassembly	6/1710	10	444	456	832	6
GO:004			32/232	0.02740	0.09417	0.06432	
5672	positive regulation of osteoclast differentiation	6/1710	10	444	456	832	6
GO:004			32/232	0.02740	0.09417	0.06432	
6466	membrane lipid catabolic process	6/1710	10	444	456	832	6
GO:004			32/232	0.02740	0.09417	0.06432	
8048	embryonic eye morphogenesis	6/1710	10	444	456	832	6
GO:005			32/232	0.02740	0.09417	0.06432	
1150	regulation of smooth muscle cell differentiation	6/1710	10	444	456	832	6
GO:006			32/232	0.02740	0.09417	0.06432	
0306	regulation of membrane repolarization	6/1710	10	444	456	832	6
GO:190			32/232	0.02740	0.09417	0.06432	
0181	negative regulation of protein localization to nucleus	6/1710	10	444	456	832	6
GO:007		13/171	98/232	0.02757	0.09470	0.06468	
2080	nephron tubule development	0	10	4	152	827	13
GO:000		12/171	88/232	0.02764	0.09473	0.06471	
9116	nucleoside metabolic process	0	10	04	638	209	12
GO:003	regulation of chemokine production	12/171	88/232	0.02764	0.09473	0.06471	12

2642		0	10	04	638	209	
GO:003		18/171	150/23	0.02764	0.09473	0.06471	
4614	cellular response to reactive oxygen species	0	210	901	638	209	18
GO:005		18/171	150/23	0.02764	0.09473	0.06471	
0715	positive regulation of cytokine secretion	0	210	901	638	209	18
GO:001			59/232	0.02785	0.09532	0.06511	
5807	L-amino acid transport	9/1710	10	307	376	331	9
GO:005			59/232	0.02785	0.09532	0.06511	
1148	negative regulation of muscle cell differentiation	9/1710	10	307	376	331	9
GO:004		31/171	294/23	0.02807	0.09602	0.06559	
2254	ribosome biogenesis	0	210	456	553	267	31
GO:000			24/232	0.02823	0.09624	0.06574	
6221	pyrimidine nucleotide biosynthetic process	5/1710	10	852	821	478	5
GO:003			24/232	0.02823	0.09624	0.06574	
2878	regulation of establishment or maintenance of cell polarity	5/1710	10	852	821	478	5
GO:004			24/232	0.02823	0.09624	0.06574	
2572	retinol metabolic process	5/1710	10	852	821	478	5
GO:004			24/232	0.02823	0.09624	0.06574	
3496	regulation of protein homodimerization activity	5/1710	10	852	821	478	5
GO:004			24/232	0.02823	0.09624	0.06574	
5723	positive regulation of fatty acid biosynthetic process	5/1710	10	852	821	478	5
GO:009			24/232	0.02823	0.09624	0.06574	
9623	regulation of cardiac muscle cell membrane repolarization	5/1710	10	852	821	478	5
GO:004	positive regulation of cysteine-type endopeptidase activity involved in apoptotic	15/171	119/23	0.02858	0.09738	0.06651	
3280	process	0	210	805	272	974	15
GO:006	adipose tissue development	8/1710	50/232	0.02867	0.09747	0.06658	8

0612			10	274	319	154	
GO:190			50/232	0.02867	0.09747	0.06658	
0047	negative regulation of hemostasis	8/1710	10	274	319	154	8
GO:000			41/232	0.02871	0.09747	0.06658	
1937	negative regulation of endothelial cell proliferation	7/1710	10	471	319	154	7
GO:005			41/232	0.02871	0.09747	0.06658	
0856	regulation of T cell receptor signaling pathway	7/1710	10	471	319	154	7
GO:005			41/232	0.02871	0.09747	0.06658	
1154	negative regulation of striated muscle cell differentiation	7/1710	10	471	319	154	7
GO:007			41/232	0.02871	0.09747	0.06658	
1364	cellular response to epidermal growth factor stimulus	7/1710	10	471	319	154	7
GO:200		21/171	183/23	0.02873	0.09749	0.06659	
1235	positive regulation of apoptotic signaling pathway	0	210	912	939	943	21
GO:001		22/171	194/23	0.02885	0.09784	0.06683	
6051	carbohydrate biosynthetic process	0	210	719	311	422	22
GO:003		10/171	69/232	0.02912	0.09869	0.06741	
2637	interleukin-8 production	0	10	482	329	495	10
GO:190		47/171	481/23	0.02919	0.09885	0.06752	
1615	organic hydroxy compound metabolic process	0	210	055	865	791	47
GO:003		14/171	109/23	0.02922	0.09886	0.06752	
2092	positive regulation of protein binding	0	210	489	033	906	14
GO:007		14/171	109/23	0.02922	0.09886	0.06752	
2009	nephron epithelium development	0	210	489	033	906	14
GO:005		18/171	151/23	0.02931	0.09909	0.06768	
1236	establishment of RNA localization	0	210	025	171	711	18
GO:000	mitochondrial matrix	73/173	264/23	1.479E-	1.0441E	7.8774E	73

5759		7	436	23	-20	-21	
GO:000		91/173	433/23	5.7622E	2.0341E	1.5346E	
5743	mitochondrial inner membrane	7	436	-20	-17	-17	91
GO:001		95/173	475/23	3.1029E	7.3021E	5.5089E	
9866	organelle inner membrane	7	436	-19	-17	-17	95
GO:009		63/173	263/23	4.2384E	7.4809E	5.6438E	
8798	mitochondrial protein complex	7	436	-17	-15	-15	63
GO:004		53/173	226/23	3.3571E	4.7402E	3.5762E	
3292	contractile fiber	7	436	-14	-12	-12	53
GO:001		86/173	490/23	4.8611E	5.7199E	4.3153E	
5629	actin cytoskeleton	7	436	-14	-12	-12	86
GO:006		70/173	359/23	6.1456E	6.1982E	4.6762E	
2023	collagen-containing extracellular matrix	7	436	-14	-12	-12	70
GO:004		49/173	203/23	9.5752E	8.4501E	6.3751E	
4449	contractile fiber part	7	436	-14	-12	-12	49
GO:003		50/173	212/23	1.4065E	1.1033E	8.3238E	
0016	myofibril	7	436	-13	-11	-12	50
GO:003		46/173	189/23	3.9533E	2.791E-	2.1056E	
0017	sarcomere	7	436	-13	11	-11	46
GO:003		38/173	142/23	1.9084E	1.2248E	9.2407E	
1674	I band	7	436	-12	-10	-11	38
GO:000		28/173	86/234	9.1306E	4.5573E	3.4382E	
1725	stress fiber	7	36	-12	-10	-10	28
GO:009		28/173	86/234	9.1306E	4.5573E	3.4382E	
7517	contractile actin filament bundle	7	36	-12	-10	-10	28
GO:000	organellar ribosome	29/173	92/234	9.6827E	4.5573E	3.4382E	29

0313		7	36	-12	-10	-10	
GO:000		29/173	92/234	9.6827E	4.5573E	3.4382E	
5761	mitochondrial ribosome	7	36	-12	-10	-10	29
GO:003		78/173	475/23	2.3122E	1.0203E	7.6973E	
1012	extracellular matrix	7	436	-11	-09	-10	78
GO:004		29/173	97/234	4.1585E	1.727E-	1.3029E	
2641	actomyosin	7	36	-11	09	-09	29
GO:003		28/173	95/234	1.3031E	5.1112E	3.8561E	
2432	actin filament bundle	7	36	-10	-09	-09	28
GO:004		38/173	163/23	1.6694E	6.2032E	4.6799E	
2383	sarcolemma	7	436	-10	-09	-09	38
GO:003		33/173	130/23	2.4598E	8.6831E	6.5509E	
0018	Z disc	7	436	-10	-09	-09	33
GO:004		43/173	216/23	2.172E-	7.3019E	5.5088E	
4455	mitochondrial membrane part	7	436	09	-08	-08	43
GO:000		32/173	143/23	1.3121E	4.0277E	3.0386E	
5777	peroxisome	7	436	-08	-07	-07	32
GO:004		32/173	143/23	1.3121E	4.0277E	3.0386E	
2579	microbody	7	436	-08	-07	-07	32
GO:004		41/173	213/23	1.4075E	4.1403E	3.1236E	
3209	myelin sheath	7	436	-08	-07	-07	41
GO:000		17/173	47/234	1.8249E	4.9552E	3.7384E	
9295	nucleoid	7	36	-08	-07	-07	17
GO:004		17/173	47/234	1.8249E	4.9552E	3.7384E	
2645	mitochondrial nucleoid	7	36	-08	-07	-07	17
GO:000	actin filament	29/173	124/23	2.1919E	5.7313E	4.3239E	29

5884		7	436	-08	-07	-07	
GO:000		19/173	60/234	3.2909E	8.0118E	6.0444E	
0315	organellar large ribosomal subunit	7	36	-08	-07	-07	19
GO:000		19/173	60/234	3.2909E	8.0118E	6.0444E	
5762	mitochondrial large ribosomal subunit	7	36	-08	-07	-07	19
GO:000		45/173	265/23	1.4248E	3.353E-	2.5296E	
5840	ribosome	7	436	-07	06	-06	45
GO:003		22/173	88/234	3.1094E	7.0813E	5.3424E	
1970	organelle envelope lumen	7	36	-07	-06	-06	22
GO:000		32/173	168/23	6.7192E	1.4824E	1.1184E	
5741	mitochondrial outer membrane	7	436	-07	-05	-05	32
GO:199		24/173	108/23	9.4963E	2.0316E	1.5327E	
0204	oxidoreductase complex	7	436	-07	-05	-05	24
GO:009		19/173	73/234	1.0099E	2.097E-	1.5821E	
8573	intrinsic component of mitochondrial membrane	7	36	-06	05	-05	19
GO:001		33/173	185/23	2.0937E	4.106E-	3.0977E	
9867	outer membrane	7	436	-06	05	-05	33
GO:003		33/173	185/23	2.0937E	4.106E-	3.0977E	
1968	organelle outer membrane	7	436	-06	05	-05	33
GO:001		20/173	84/234	2.4161E	4.6102E	3.4781E	
6528	sarcoplasm	7	36	-06	-05	-05	20
GO:004		35/173	208/23	4.1337E	7.6801E	5.7941E	
4448	cell cortex part	7	436	-06	-05	-05	35
GO:004		37/173	230/23	6.5293E	0.00011	8.8431E	
4391	ribosomal subunit	7	436	-06	722	-05	37
GO:000	mitochondrial intermembrane space	18/173	75/234	6.7517E	0.00011	8.8431E	18

5758		7	36	-06	722	-05	
GO:009		25/173	128/23	6.8071E	0.00011	8.8431E	
8800	inner mitochondrial membrane protein complex	7	436	-06	722	-05	25
GO:004		64/173	491/23	7.318E-	0.00012	9.214E-	
4431	Golgi apparatus part	7	436	06	213	05	64
GO:003		47/173	324/23	7.4386E	0.00012	9.214E-	
0133	transport vesicle	7	436	-06	213	05	47
GO:004		19/173	83/234	7.9401E	0.00012	9.49E-0	
4291	cell-cell contact zone	7	36	-06	579	5	19
GO:001		16/173	62/234	8.0177E	0.00012	9.49E-0	
4704	intercalated disc	7	36	-06	579	5	16
GO:000		55/173	407/23	1.1153E	0.00017	0.00012	
5635	nuclear envelope	7	436	-05	118	914	55
GO:003		34/173	209/23	1.1912E	0.00017	0.00013	
5770	ribonucleoprotein granule	7	436	-05	893	499	34
GO:003		17/173	71/234	1.2494E	0.00018	0.00013	
2592	integral component of mitochondrial membrane	7	36	-05	377	864	17
GO:003		38/173	249/23	1.7083E	0.00024	0.00018	
1300	intrinsic component of organelle membrane	7	436	-05	613	569	38
GO:004			14/234	2.6165E	0.00036	0.00027	
5239	tricarboxylic acid cycle enzyme complex	7/1737	36	-05	945	873	7
GO:001		17/173	75/234	2.6957E	0.00037	0.00028	
6529	sarcoplasmic reticulum	7	36	-05	317	153	17
GO:003		24/173	131/23	3.1358E	0.00042	0.00032	
0863	cortical cytoskeleton	7	436	-05	574	12	24
GO:000	basement membrane	21/173	107/23	3.3448E	0.00044	0.00033	21

5604		7	436	-05	556	614	
GO:000		27/173	158/23	3.7716E	0.00048	0.00036	
5924	cell-substrate adherens junction	7	436	-05	867	867	27
GO:001		11/173	36/234	3.8069E	0.00048	0.00036	
6592	mediator complex	7	36	-05	867	867	11
GO:000		42/173	297/23	4.0841E	0.00051	0.00038	
5912	adherens junction	7	436	-05	489	845	42
GO:003		12/173	43/234	4.657E-	0.00057	0.00043	
1304	intrinsic component of mitochondrial inner membrane	7	36	05	681	517	12
GO:000		26/173	152/23	5.1196E	0.00062	0.00047	
5925	focal adhesion	7	436	-05	318	015	26
GO:004		14/173	57/234	5.3103E	0.00062	0.00047	
4438	microbody part	7	36	-05	484	14	14
GO:004		14/173	57/234	5.3103E	0.00062	0.00047	
4439	peroxisomal part	7	36	-05	484	14	14
GO:007		43/173	311/23	5.6929E	0.00065	0.00049	
0161	anchoring junction	7	436	-05	888	709	43
GO:000		43/173	312/23	6.1392E	0.00069	0.00052	
5938	cell cortex	7	436	-05	907	74	43
GO:001			16/234	7.6398E	0.00085	0.00064	
6460	myosin II complex	7/1737	36	-05	615	591	7
GO:003		27/173	165/23	8.1654E	0.00090	0.00067	
0055	cell-substrate junction	7	436	-05	075	955	27
GO:009		55/173	438/23	8.6874E	0.00094	0.00071	
8791	Golgi subcompartment	7	436	-05	359	188	55
GO:003	integral component of organelle membrane	33/173	222/23	0.00010	0.00107	0.00080	33

1301		7	436	011	088	791	
GO:000		54/173	431/23	0.00010	0.00112	0.00084	
5764	lysosome	7	436	632	029	518	54
GO:000		54/173	432/23	0.00011	0.00117	0.00088	
0323	lytic vacuole	7	436	294	263	467	54
GO:003		30/173	198/23	0.00014	0.00146	0.00110	
6464	cytoplasmic ribonucleoprotein granule	7	436	358	913	837	30
GO:003		15/173	70/234	0.00015	0.00156	0.00118	
0315	T-tubule	7	36	517	501	07	15
GO:000		11/173	42/234	0.00017	0.00173	0.00130	
5778	peroxisomal membrane	7	36	944	536	922	11
GO:003		11/173	42/234	0.00017	0.00173	0.00130	
1305	integral component of mitochondrial inner membrane	7	36	944	536	922	11
GO:003		11/173	42/234	0.00017	0.00173	0.00130	
1903	microbody membrane	7	36	944	536	922	11
GO:000		52/173	421/23	0.00020	0.00192	0.00145	
5667	transcription factor complex	7	436	176	493	224	52
GO:003			24/234	0.00022	0.00207	0.00156	
3017	sarcoplasmic reticulum membrane	8/1737	36	448	505	549	8
GO:000			30/234	0.00022	0.00207	0.00156	
0314	organellar small ribosomal subunit	9/1737	36	632	505	549	9
GO:000			30/234	0.00022	0.00207	0.00156	
5763	mitochondrial small ribosomal subunit	9/1737	36	632	505	549	9
GO:004		17/173	89/234	0.00025	0.00231	0.00174	
5335	phagocytic vesicle	7	36	605	755	844	17
GO:004	plasma membrane raft	21/173	123/23	0.00026	0.00238	0.00179	21

4853		7	436	673	37	835	
GO:001		24/173	150/23	0.00028	0.00247	0.00186	
5934	large ribosomal subunit	7	436	023	299	571	24
GO:000		31/173	219/23	0.00038	0.00336	0.00253	
5802	trans-Golgi network	7	436	602	459	836	31
GO:000		15/173	77/234	0.00046	0.00400	0.00301	
5746	mitochondrial respiratory chain	7	36	482	202	926	15
GO:000		17/173	94/234	0.00050	0.00425	0.00321	
5901	caveola	7	36	025	512	021	17
GO:003		32/173	233/23	0.00053	0.00451	0.00340	
1965	nuclear membrane	7	436	776	975	986	32
GO:009		15/173	81/234	0.00081	0.00674	0.00508	
8803	respiratory chain complex	7	36	153	05	528	15
GO:001		31/173	229/23	0.00083	0.00681	0.00514	
6323	basolateral plasma membrane	7	436	067	922	467	31
GO:000		11/173	50/234	0.00090	0.00717	0.00541	
5747	mitochondrial respiratory chain complex I	7	36	497	877	592	11
GO:003		11/173	50/234	0.00090	0.00717	0.00541	
0964	NADH dehydrogenase complex	7	36	497	877	592	11
GO:004		11/173	50/234	0.00090	0.00717	0.00541	
5271	respiratory chain complex I	7	36	497	877	592	11
GO:003			29/234	0.00093	0.00736	0.00555	
0140	trans-Golgi network transport vesicle	8/1737	36	863	302	492	8
GO:007			23/234	0.00103	0.00801	0.00604	
1782	endoplasmic reticulum tubular network	7/1737	36	255	078	362	7
GO:000	Golgi-associated vesicle	21/173	136/23	0.00104	0.00804	0.00606	21

5798		7	436	923	443	9	
GO:001		39/173	314/23	0.00106	0.00804	0.00606	
9898	extrinsic component of membrane	7	436	826	443	9	39
GO:009		45/173	377/23	0.00107	0.00804	0.00606	
8589	membrane region	7	436	107	443	9	45
GO:190		17/173	101/23	0.00116	0.00863	0.00651	
2911	protein kinase complex	7	436	178	389	372	17
GO:003		38/173	306/23	0.00123	0.00906	0.00683	
0659	cytoplasmic vesicle membrane	7	436	212	122	611	38
GO:001		12/173	60/234	0.00131	0.00956	0.00721	
6459	myosin complex	7	36	41	446	577	12
GO:001		40/173	332/23	0.00165	0.01190	0.00898	
2506	vesicle membrane	7	436	306	876	439	40
GO:000			13/234	0.00172	0.01232	0.00929	
2199	zona pellucida receptor complex	5/1737	36	766	045	499	5
GO:003			32/234	0.00188	0.01327	0.01001	
6379	myofilament	8/1737	36	485	786	729	8
GO:009		53/173	475/23	0.00189	0.01327	0.01001	
9568	cytoplasmic region	7	436	952	786	729	53
GO:000		33/173	263/23	0.00209	0.01453	0.01096	
5769	early endosome	7	436	982	402	498	33
GO:007		15/173	89/234	0.00217	0.01493	0.01126	
0469	respiratory chain	7	36	885	463	722	15
GO:009			26/234	0.00227	0.01545	0.01166	
7038	perinuclear endoplasmic reticulum	7/1737	36	682	608	061	7
GO:001	nuclear speck	38/173	317/23	0.00232	0.01561	0.01178	38

6607		7	436	377	447	011	
GO:001		12/173	64/234	0.00234	0.01561	0.01178	
0494	cytoplasmic stress granule	7	36	438	447	011	12
GO:003		16/173	99/234	0.00246	0.01625	0.01226	
0864	cortical actin cytoskeleton	7	36	394	738	515	16
GO:004		36/173	299/23	0.00276	0.01792	0.01351	
4445	cytosolic part	7	436	647	049	985	36
GO:004		27/173	206/23	0.00276	0.01792	0.01351	
4798	nuclear transcription factor complex	7	436	676	049	985	27
GO:007		31/173	247/23	0.00280	0.01797	0.01355	
0382	exocytic vesicle	7	436	005	12	811	31
GO:004		42/173	364/23	0.00290	0.01847	0.01393	
5121	membrane raft	7	436	435	268	645	42
GO:009		42/173	365/23	0.00305	0.01924	0.01451	
8857	membrane microdomain	7	436	311	551	95	42
GO:000		26/173	198/23	0.00319	0.01994	0.01504	
5770	late endosome	7	436	281	803	951	26
GO:000		17/173	112/23	0.00361	0.02239	0.01689	
5913	cell-cell adherens junction	7	436	641	634	66	17
GO:001		14/173	85/234	0.00374	0.02295	0.01731	
5935	small ribosomal subunit	7	36	222	409	739	14
GO:004		10/173	51/234	0.00377	0.02295	0.01731	
4420	extracellular matrix component	7	36	149	409	739	10
GO:000			10/234	0.00439	0.02605	0.01965	
5832	chaperonin-containing T-complex	4/1737	36	186	588	749	4
GO:004	laminin complex	4/1737	10/234	0.00439	0.02605	0.01965	4

3256			36	186	588	749	
GO:004			10/234	0.00439	0.02605	0.01965	
5240	dihydrolipoyl dehydrogenase complex	4/1737	36	186	588	749	4
GO:009		22/173	163/23	0.00456	0.02686	0.02026	
0575	RNA polymerase II transcription factor complex	7	436	659	679	927	22
GO:006		30/173	245/23	0.00465	0.02714	0.02047	
1695	transferase complex, transferring phosphorus-containing groups	7	436	192	258	733	30
GO:003			37/234	0.00497	0.02877	0.02170	
1941	filamentous actin	8/1737	36	209	291	731	8
GO:003		15/173	97/234	0.00507	0.02912	0.02197	
1594	neuromuscular junction	7	36	382	289	135	15
GO:000			30/234	0.00543	0.03019	0.02277	
5865	striated muscle thin filament	7/1737	36	101	126	736	7
GO:004			30/234	0.00543	0.03019	0.02277	
4215	other organism	7/1737	36	101	126	736	7
GO:004			30/234	0.00543	0.03019	0.02277	
4216	other organism cell	7/1737	36	101	126	736	7
GO:004			30/234	0.00543	0.03019	0.02277	
4217	other organism part	7/1737	36	101	126	736	7
GO:000		28/173	227/23	0.00552	0.03045	0.02297	
8021	synaptic vesicle	7	436	239	942	967	28
GO:003			17/234	0.00647	0.03529	0.02663	
2982	myosin filament	5/1737	36	279	836	034	5
GO:004			11/234	0.00649	0.03529	0.02663	
4666	MLL3/4 complex	4/1737	36	97	836	034	4
GO:003	coated vesicle	23/173	181/23	0.00789	0.04255	0.03210	23

0135		7	436	584	315	361	
GO:003		19/173	141/23	0.00814	0.04356	0.03286	
0176	integral component of endoplasmic reticulum membrane	7	436	547	591	768	19
GO:000			18/234	0.00842	0.04437	0.03348	
5782	peroxisomal matrix	5/1737	36	336	978	169	5
GO:003			18/234	0.00842	0.04437	0.03348	
1907	microbody lumen	5/1737	36	336	978	169	5
GO:004		11/173	66/234	0.00877	0.04591	0.03463	
5178	basal part of cell	7	36	912	153	73	11
GO:003		22/173	173/23	0.00914	0.04732	0.03570	
0139	endocytic vesicle	7	436	857	551	405	22
GO:003			12/234	0.00918	0.04732	0.03570	
2009	early phagosome	4/1737	36	356	551	405	4
GO:003		41/173	383/23	0.01139	0.05829	0.04398	
0141	secretory granule	7	436	504	636	085	41
GO:000			13/234	0.01249	0.06307	0.04758	
5641	nuclear envelope lumen	4/1737	36	73	404	531	4
GO:000			27/234	0.01258	0.06307	0.04758	
2102	podosome	6/1737	36	542	404	531	6
GO:000			43/234	0.01259	0.06307	0.04758	
9925	basal plasma membrane	8/1737	36	694	404	531	8
GO:000		44/173	420/23	0.01288	0.06406	0.04833	
5874	microtubule	7	436	496	186	055	44
GO:003		14/173	98/234	0.01315	0.06425	0.04847	
0136	clathrin-coated vesicle	7	36	285	493	621	14
GO:000	gamma-tubulin complex	5/1737	20/234	0.01346	0.06425	0.04847	5

0930			36	987	493	621	
GO:001			20/234	0.01346	0.06425	0.04847	
6010	dystrophin-associated glycoprotein complex	5/1737	36	987	493	621	5
GO:003			20/234	0.01346	0.06425	0.04847	
3646	host intracellular part	5/1737	36	987	493	621	5
GO:004			20/234	0.01346	0.06425	0.04847	
3656	intracellular region of host	5/1737	36	987	493	621	5
GO:009			20/234	0.01346	0.06425	0.04847	
0665	glycoprotein complex	5/1737	36	987	493	621	5
GO:003		19/173	149/23	0.01428	0.06768	0.05106	
1227	intrinsic component of endoplasmic reticulum membrane	7	436	43	264	22	19
GO:004		23/173	193/23	0.01632	0.07660	0.05779	
3197	dendritic spine	7	436	5	558	398	23
GO:007			14/234	0.01648	0.07660	0.05779	
1437	invadopodium	4/1737	36	643	558	398	4
GO:000		13/173	91/234	0.01649	0.07660	0.05779	
5902	microvillus	7	36	299	558	398	13
GO:010			21/234	0.01662	0.07670	0.05786	
1031	chaperone complex	5/1737	36	316	554	94	5
GO:190		12/173	82/234	0.01738	0.07972	0.06014	
2554	serine/threonine protein kinase complex	7	36	938	015	372	12
GO:000			46/234	0.01863	0.08489	0.06404	
0791	euchromatin	8/1737	36	857	567	832	8
GO:003			22/234	0.02023	0.09155	0.06907	
3643	host cell part	5/1737	36	026	489	227	5
GO:000	phagophore assembly site	6/1737	30/234	0.02089	0.09395	0.07087	6

0407			36	257	004	926	
GO:004		23/173	198/23	0.02145	0.09587	0.07233	
4309	neuron spine	7	436	718	828	4	23
GO:009		24/173	209/23	0.02166	0.09621	0.07259	
8858	actin-based cell projection	7	436	95	804	032	24
GO:005		45/168	200/22	1.4458E	1.023E-	8.4477E	
1015	actin filament binding	9	710	-11	08	-09	45
GO:000		73/168	428/22	1.9085E	1.023E-	8.4477E	
3779	actin binding	9	710	-11	08	-09	73
GO:005		49/168	288/22	4.1871E	1.4962E	1.2356E	
0662	coenzyme binding	9	710	-08	-05	-05	49
GO:001		21/168	80/227	2.4951E	6.6869E	5.5221E	
6651	oxidoreductase activity, acting on NAD(P)H	9	10	-07	-05	-05	21
GO:001		30/168	147/22	3.4249E	6.8705E	5.6737E	
9838	growth factor binding	9	710	-07	-05	-05	30
GO:000		29/168	140/22	3.8454E	6.8705E	5.6737E	
5201	extracellular matrix structural constituent	9	710	-07	-05	-05	29
GO:001		16/168	55/227	1.4974E	0.00022	0.00018	
6836	hydro-lyase activity	9	10	-06	931	937	16
GO:000		62/168	454/22	2.477E-	0.00033	0.00027	
3712	transcription coregulator activity	9	710	06	191	41	62
GO:001		32/168	184/22	5.5381E	0.00065	0.00054	
6829	lyase activity	9	710	-06	964	474	32
GO:000		17/168	70/227	1.0659E	0.00114	0.00094	
9055	electron transfer activity	9	10	-05	268	363	17
GO:009	scaffold protein binding	17/168	71/227	1.3052E	0.00127	0.00105	17

7110		9	10	-05	2	043	
GO:003		51/168	371/22	1.5646E	0.00139	0.00115	
3218	amide binding	9	710	-05	772	424	51
GO:190		38/168	250/22	2.0137E	0.00157	0.00130	
1681	sulfur compound binding	9	710	-05	465	035	38
GO:005		37/168	241/22	2.0564E	0.00157	0.00130	
0839	cell adhesion molecule binding	9	710	-05	465	035	37
GO:001		16/168	67/227	2.4121E	0.00172	0.00142	
6835	carbon-oxygen lyase activity	9	10	-05	387	358	16
GO:000		30/168	182/22	3.1197E	0.00209	0.00172	
3735	structural constituent of ribosome	9	710	-05	018	608	30
GO:004		24/168	136/22	6.192E-	0.00389	0.00321	
4325	ion channel binding	9	710	05	777	88	24
GO:000		23/168	128/22	6.5448E	0.00389	0.00321	
5178	integrin binding	9	710	-05	777	88	23
GO:005			21/227	7.778E-	0.00412	0.00340	
1371	muscle alpha-actinin binding	8/1689	10	05	573	705	8
GO:005		14/168	59/227	8.2731E	0.00412	0.00340	
0840	extracellular matrix binding	9	10	-05	573	705	14
GO:005		19/168	97/227	8.3711E	0.00412	0.00340	
1082	unfolded protein binding	9	10	-05	573	705	19
GO:003		42/168	306/22	8.7789E	0.00412	0.00340	
1625	ubiquitin protein ligase binding	9	710	-05	573	705	42
GO:003		24/168	139/22	8.8518E	0.00412	0.00340	
1072	heat shock protein binding	9	710	-05	573	705	24
GO:001	transferase activity, transferring alkyl or aryl (other than methyl) groups	14/168	61/227	0.00012	0.00543	0.00448	14

6765		9	10	172	678	972	
GO:000		34/168	234/22	0.00013	0.00559	0.00462	
8022	protein C-terminus binding	9	710	284	709	21	34
GO:000		15/168	69/227	0.00013	0.00559	0.00462	
5518	collagen binding	9	10	575	709	21	15
GO:005		14/168	62/227	0.00014	0.00581	0.00480	
1287	NAD binding	9	10	658	959	585	14
GO:004		43/168	325/22	0.00016	0.00630	0.00520	
4389	ubiquitin-like protein ligase binding	9	710	82	306	51	43
GO:000		29/168	190/22	0.00017	0.00630	0.00520	
5516	calmodulin binding	9	710	08	306	51	29
GO:000			13/227	0.00018	0.00630	0.00520	
4659	prenyltransferase activity	6/1689	10	227	306	51	6
GO:004			13/227	0.00018	0.00630	0.00520	
4548	S100 protein binding	6/1689	10	227	306	51	6
GO:001	oxidoreductase activity, acting on NAD(P)H, quinone or similar compound as	10/168	36/227	0.00021	0.00714	0.00589	
6655	acceptor	9	10	32	222	808	10
GO:003		37/168	270/22	0.00023	0.00752	0.00621	
0246	carbohydrate binding	9	710	15	04	039	37
GO:005		14/168	65/227	0.00024	0.00763	0.00630	
1536	iron-sulfur cluster binding	9	10	918	19	246	14
GO:005		14/168	65/227	0.00024	0.00763	0.00630	
1540	metal cluster binding	9	10	918	19	246	14
GO:001		24/168	149/22	0.00026	0.00790	0.00652	
6614	oxidoreductase activity, acting on CH-OH group of donors	9	710	56	52	816	24
GO:005	alpha-actinin binding	10/168	37/227	0.00027	0.00790	0.00652	10

1393		9	10	285	52	816	
GO:000			14/227	0.00029	0.00843	0.00696	
5159	insulin-like growth factor receptor binding	6/1689	10	891	242	353	6
GO:005		18/168	99/227	0.00033	0.00926	0.00765	
1087	chaperone binding	9	10	718	815	369	18
GO:003		45/168	361/22	0.00045	0.01231	0.01017	
2550	purine ribonucleoside binding	9	710	955	606	067	45
GO:000		24/168	155/22	0.00048	0.01262	0.01042	
8201	heparin binding	9	710	268	027	188	24
GO:000		13/168	62/227	0.00054	0.01378	0.01138	
5080	protein kinase C binding	9	10	015	662	506	13
GO:000		45/168	365/22	0.00058	0.01412	0.01166	
1883	purine nucleoside binding	9	710	244	707	621	45
GO:001		12/168	55/227	0.00059	0.01412	0.01166	
6667	oxidoreductase activity, acting on a sulfur group of donors	9	10	891	707	621	12
GO:003		45/168	366/22	0.00061	0.01412	0.01166	
2549	ribonucleoside binding	9	710	74	707	621	45
GO:000		13/168	63/227	0.00063	0.01412	0.01166	
5200	structural constituent of cytoskeleton	9	10	49	707	621	13
GO:001		46/168	377/22	0.00063	0.01412	0.01166	
9001	guanyl nucleotide binding	9	710	765	707	621	46
GO:003		46/168	377/22	0.00063	0.01412	0.01166	
2561	guanyl ribonucleotide binding	9	710	765	707	621	46
GO:004		11/168	48/227	0.00064	0.01412	0.01166	
2805	actinin binding	9	10	573	707	621	11
GO:000	enzyme activator activity	53/168	452/22	0.00067	0.01438	0.01187	53

8047		9	710	097	558	969	
GO:000			28/227	0.00074	0.01561	0.01289	
3954	NADH dehydrogenase activity	8/1689	10	301	788	733	8
GO:003			22/227	0.00078	0.01567	0.01294	
4237	protein kinase A regulatory subunit binding	7/1689	10	279	351	326	7
GO:000		29/168	208/22	0.00078	0.01567	0.01294	
5539	glycosaminoglycan binding	9	710	952	351	326	29
GO:003		29/168	208/22	0.00078	0.01567	0.01294	
1406	carboxylic acid binding	9	710	952	351	326	29
GO:000		34/168	258/22	0.00082	0.01606	0.01326	
3713	transcription coactivator activity	9	710	443	892	98	34
GO:004		13/168	65/227	0.00086	0.01659	0.01370	
3621	protein self-association	9	10	682	345	295	13
GO:001		23/168	153/22	0.00094	0.01786	0.01475	
6853	isomerase activity	9	710	996	59	375	23
GO:000			17/227	0.00101	0.01868	0.01543	
5086	ARF guanyl-nucleotide exchange factor activity	6/1689	10	435	958	395	6
GO:000		43/168	354/22	0.00102	0.01868	0.01543	
5525	GTP binding	9	710	862	958	395	43
GO:000		45/168	376/22	0.00108	0.01936	0.01599	
1882	nucleoside binding	9	710	381	401	09	45
GO:003			12/227	0.00114	0.01979	0.01634	
2036	myosin heavy chain binding	5/1689	10	97	74	88	5
GO:005		16/168	92/227	0.00116	0.01979	0.01634	
1213	dioxygenase activity	9	10	059	74	88	16
GO:004	organic acid binding	30/168	223/22	0.00116	0.01979	0.01634	30

3177		9	710	347	74	88	
GO:000		10/168	44/227	0.00120	0.02019	0.01668	
8009	chemokine activity	9	10	593	939	076	10
GO:004		15/168	84/227	0.00123	0.02042	0.01686	
6332	SMAD binding	9	10	859	717	886	15
GO:004		15/168	85/227	0.00140	0.02278	0.01881	
8029	monosaccharide binding	9	10	25	008	191	15
GO:001			38/227	0.00154	0.02465	0.02035	
9205	nucleobase-containing compound kinase activity	9/1689	10	083	334	886	9
GO:001		15/168	86/227	0.00158	0.02468	0.02038	
6209	antioxidant activity	9	10	422	566	555	15
GO:001	oxidoreductase activity, acting on the CH-OH group of donors, NAD or NADP as	21/168	140/22	0.00159	0.02468	0.02038	
6616	acceptor	9	710	01	566	555	21
GO:000		26/168	188/22	0.00161	0.02468	0.02038	
3714	transcription corepressor activity	9	710	194	566	555	26
GO:000			13/227	0.00175	0.02509	0.02072	
2161	aminoacyl-tRNA editing activity	5/1689	10	434	512	368	5
GO:001			13/227	0.00175	0.02509	0.02072	
6840	carbon-nitrogen lyase activity	5/1689	10	434	512	368	5
GO:001		20/168	132/22	0.00179	0.02509	0.02072	
9842	vitamin binding	9	710	454	512	368	20
GO:000			25/227	0.00181	0.02509	0.02072	
1671	ATPase activator activity	7/1689	10	152	512	368	7
GO:000			25/227	0.00181	0.02509	0.02072	
8137	NADH dehydrogenase (ubiquinone) activity	7/1689	10	152	512	368	7
GO:000	structural constituent of muscle	7/1689	25/227	0.00181	0.02509	0.02072	7

8307			10	152	512	368	
GO:005			25/227	0.00181	0.02509	0.02072	
0136	NADH dehydrogenase (quinone) activity	7/1689	10	152	512	368	7
GO:000		36/168	291/22	0.00184	0.02509	0.02072	
3924	GTPase activity	9	710	227	512	368	36
GO:005			39/227	0.00187	0.02509	0.02072	
1539	4 iron, 4 sulfur cluster binding	9/1689	10	277	512	368	9
GO:006			39/227	0.00187	0.02509	0.02072	
0590	ATPase regulator activity	9/1689	10	277	512	368	9
GO:000			26/227	0.00232	0.03023	0.02496	
5158	insulin receptor binding	7/1689	10	164	644	941	7
GO:000			26/227	0.00232	0.03023	0.02496	
5537	mannose binding	7/1689	10	164	644	941	7
GO:001		28/168	213/22	0.00234	0.03023	0.02496	
9902	phosphatase binding	9	710	845	644	941	28
GO:001			33/227	0.00237	0.03023	0.02496	
6504	peptidase activator activity	8/1689	10	898	644	941	8
GO:004		37/168	306/22	0.00239	0.03023	0.02496	
2277	peptide binding	9	710	748	644	941	37
GO:001		33/168	265/22	0.00250	0.03118	0.02575	
6791	phosphatase activity	9	710	195	71	447	33
GO:003			14/227	0.00256	0.03157	0.02607	
0021	extracellular matrix structural constituent conferring compression resistance	5/1689	10	291	974	871	5
GO:003			41/227	0.00270	0.03297	0.02723	
2451	demethylase activity	9/1689	10	692	52	109	9
GO:003	Hsp70 protein binding	10/168	49/227	0.00284	0.03426	0.02829	10

0544		9	10	513	942	986	
GO:005			34/227	0.00291	0.03467	0.02863	
1019	mitogen-activated protein kinase binding	8/1689	10	119	549	52	8
GO:001		13/168	74/227	0.00296	0.03491	0.02883	
7022	myosin binding	9	10	386	489	29	13
GO:000		47/168	419/22	0.00318	0.03707	0.03062	
5543	phospholipid binding	9	710	224	998	084	47
GO:004		41/168	355/22	0.00336	0.03882	0.03206	
2578	phosphoric ester hydrolase activity	9	710	844	766	409	41
GO:000		16/168	102/22	0.00346	0.03954	0.03265	
8238	exopeptidase activity	9	710	743	342	516	16
GO:003		21/168	150/22	0.00372	0.04200	0.03468	
5257	nuclear hormone receptor binding	9	710	235	374	691	21
GO:000		11/168	59/227	0.00376	0.04209	0.03476	
1540	amyloid-beta binding	9	10	983	646	347	11
GO:000		23/168	171/22	0.00412	0.04512	0.03726	
1085	RNA polymerase II transcription factor binding	9	710	556	855	739	23
GO:000		23/168	171/22	0.00412	0.04512	0.03726	
4721	phosphoprotein phosphatase activity	9	710	556	855	739	23
GO:000			36/227	0.00424	0.04601	0.03800	
3755	peptidyl-prolyl cis-trans isomerase activity	8/1689	10	984	842	225	8
GO:009			22/227	0.00443	0.04699	0.03881	
0079	translation regulator activity, nucleic acid binding	6/1689	10	325	798	118	6
GO:004			10/227	0.00444	0.04699	0.03881	
7429	nucleoside-triphosphate diphosphatase activity	4/1689	10	678	798	118	4
GO:005	protein kinase A binding	10/168	52/227	0.00447	0.04699	0.03881	10

1018		9	10	182	798	118	
GO:000		15/168	96/227	0.00474	0.04935	0.04075	
4725	protein tyrosine phosphatase activity	9	10	178	133	459	15
GO:003			23/227	0.00562	0.05743	0.04743	
0506	ankyrin binding	6/1689	10	607	95	384	6
GO:003			23/227	0.00562	0.05743	0.04743	
1681	G-protein beta-subunit binding	6/1689	10	607	95	384	6
GO:000		20/168	146/22	0.00583	0.05849	0.04830	
2020	protease binding	9	710	836	275	361	20
GO:001		20/168	146/22	0.00583	0.05849	0.04830	
6874	ligase activity	9	710	836	275	361	20
GO:000		10/168	54/227	0.00591	0.05867	0.04845	
4601	peroxidase activity	9	10	096	173	142	10
GO:000			38/227	0.00601	0.05918	0.04887	
8138	protein tyrosine/serine/threonine phosphatase activity	8/1689	10	773	351	405	8
GO:005			46/227	0.00609	0.05936	0.04902	
1879	Hsp90 protein binding	9/1689	10	155	491	385	9
GO:001		38/168	336/22	0.00652	0.06170	0.05095	
5631	tubulin binding	9	710	034	05	259	38
GO:001		12/168	72/227	0.00652	0.06170	0.05095	
9843	rRNA binding	9	10	168	05	259	12
GO:000			11/227	0.00657	0.06170	0.05095	
2162	dystroglycan binding	4/1689	10	962	05	259	4
GO:001			11/227	0.00657	0.06170	0.05095	
6846	carbon-sulfur lyase activity	4/1689	10	962	05	259	4
GO:000	fibronectin binding	7/1689	31/227	0.00669	0.06170	0.05095	7

1968			10	656	05	259	
GO:004			31/227	0.00669	0.06170	0.05095	
6875	ephrin receptor binding	7/1689	10	656	05	259	7
GO:005		23/168	178/22	0.00673	0.06170	0.05095	
1427	hormone receptor binding	9	710	41	05	259	23
GO:199		15/168	100/22	0.00695	0.06321	0.05220	
0782	protein tyrosine kinase binding	9	710	821	353	206	15
GO:003		11/168	64/227	0.00713	0.06424	0.05305	
0374	nuclear receptor transcription coactivator activity	9	10	117	045	009	11
GO:006		29/168	242/22	0.00749	0.06696	0.05529	
1659	ubiquitin-like protein ligase activity	9	710	6	423	941	29
GO:000		10/168	56/227	0.00768	0.06810	0.05624	
8375	acetylglucosaminyltransferase activity	9	10	732	588	219	10
GO:000			32/227	0.00803	0.07057	0.05828	
4364	glutathione transferase activity	7/1689	10	23	886	439	7
GO:001			40/227	0.00829	0.07170	0.05921	
5036	disulfide oxidoreductase activity	8/1689	10	439	63	543	8
GO:001			40/227	0.00829	0.07170	0.05921	
6859	cis-trans isomerase activity	8/1689	10	439	63	543	8
GO:001			18/227	0.00854	0.07269	0.06003	
6208	AMP binding	5/1689	10	435	482	176	5
GO:007			18/227	0.00854	0.07269	0.06003	
0628	proteasome binding	5/1689	10	435	482	176	5
GO:006		28/168	234/22	0.00865	0.07306	0.06033	
1630	ubiquitin protein ligase activity	9	710	592	413	674	28
GO:004	receptor ligand activity	51/168	489/22	0.00911	0.07637	0.06306	51

8018		9	710	903	184	826	
GO:004			12/227	0.00929	0.07723	0.06378	
3121	neurotrophin binding	4/1689	10	458	872	413	4
GO:014		16/168	113/22	0.00942	0.07770	0.06417	
0101	catalytic activity, acting on a tRNA	9	710	352	776	147	16
GO:001		10/168	58/227	0.00984	0.08059	0.06655	
6684	oxidoreductase activity, acting on peroxide as acceptor	9	10	896	609	667	10
GO:004		11/168	67/227	0.01005	0.08168	0.06745	
2379	chemokine receptor binding	9	10	841	647	711	11
GO:001		41/168	379/22	0.01016	0.08191	0.06764	
6887	ATPase activity	9	710	336	821	849	41
GO:003			26/227	0.01059	0.08427	0.06959	
2452	histone demethylase activity	6/1689	10	709	744	675	6
GO:000			50/227	0.01061	0.08427	0.06959	
1221	transcription cofactor binding	9/1689	10	33	744	675	9
GO:001		13/168	86/227	0.01080	0.08518	0.07034	
5238	drug transmembrane transporter activity	9	10	678	287	446	13
GO:004			42/227	0.01116	0.08733	0.07212	
2887	amide transmembrane transporter activity	8/1689	10	121	444	123	8
GO:004		32/168	282/22	0.01125	0.08746	0.07222	
2623	ATPase activity, coupled	9	710	913	223	677	32
GO:001		21/168	166/22	0.01172	0.09005	0.07436	
9903	protein phosphatase binding	9	710	748	024	395	21
GO:004		15/168	106/22	0.01176	0.09005	0.07436	
2562	hormone binding	9	710	029	024	395	15
GO:003	pyridoxal phosphate binding	9/1689	51/227	0.01205	0.09168	0.07571	9

0170			10	953	666	532	
GO:000		14/168	97/227	0.01240	0.09368	0.07736	
4197	cysteine-type endopeptidase activity	9	10	959	369	448	14
GO:000			27/227	0.01278	0.09585	0.07915	
8574	ATP-dependent microtubule motor activity, plus-end-directed	6/1689	10	608	088	415	6
GO:000		19/168	147/22	0.01290	0.09607	0.07933	
8083	growth factor activity	9	710	571	584	993	19
GO:000			35/227	0.01321	0.09701	0.08011	
1965	G-protein alpha-subunit binding	7/1689	10	288	51	557	7
GO:190			35/227	0.01321	0.09701	0.08011	
1567	fatty acid derivative binding	7/1689	10	288	51	557	7
GO:007			52/227	0.01364	0.09826	0.08114	
0279	vitamin B6 binding	9/1689	10	766	17	503	9
GO:000			20/227	0.01365	0.09826	0.08114	
4602	glutathione peroxidase activity	5/1689	10	764	17	503	5
GO:005			20/227	0.01365	0.09826	0.08114	
1861	glycolipid binding	5/1689	10	764	17	503	5
GO:001		11/168	70/227	0.01383	0.09884	0.08162	
6597	amino acid binding	9	10	085	444	625	11
mmu04			86/891	1.9864E	0.00402	0.00337	
146	Peroxisome	21/810	0	-05	232	356	21
mmu05			94/891	2.595E-	0.00402	0.00337	
414	Dilated cardiomyopathy	22/810	0	05	232	356	22
mmu00			32/891	7.0763E	0.00731	0.00613	
020	Citrate cycle (TCA cycle)	11/810	0	-05	219	28	11
mmu00	Propanoate metabolism	11/810	34/891	0.00013	0.00943	0.00791	11

640			0	246	331	181	
mmu05			91/891	0.00015	0.00943	0.00791	
410	Hypertrophic cardiomyopathy	20/810	0	215	331	181	20
mmu05			146/89	0.00026	0.01357	0.01138	
418	Fluid shear stress and atherosclerosis	27/810	10	271	332	407	27
mmu04			230/89	0.00040	0.01775	0.01488	
714	Thermogenesis	37/810	10	943	034	738	37
mmu04			151/89	0.00045	0.01775	0.01488	
932	Non-alcoholic fatty liver disease	27/810	10	993	034	738	27
mmu04			114/89	0.00051	0.01775	0.01488	
066	HIF-1 signaling pathway	22/810	10	874	034	738	22
mmu00			28/891	0.00057	0.01775	0.01488	
650	Butanoate metabolism	9/810	0	259	034	738	9
mmu05			248/89	0.00090	0.02540	0.02131	
012	Parkinson disease	38/810	10	161	895	073	38
mmu05			70/891	0.00130	0.03365	0.02823	
140	Leishmaniasis	15/810	0	295	941	048	15
mmu00			57/891	0.00147	0.03383	0.02837	
280	Valine, leucine and isoleucine degradation	13/810	0	743	667	914	13
mmu04			44/891	0.00152	0.03383	0.02837	
962	Vasopressin-regulated water reabsorption	11/810	0	811	667	914	11
mmu04			101/89	0.00166	0.03448	0.02892	
933	AGE-RAGE signaling pathway in diabetic complications	19/810	10	883	905	63	19
mmu05			110/89	0.00195	0.03777	0.03168	
145	Toxoplasmosis	20/810	10	982	572	287	20
mmu05	Prion disease	39/810	268/89	0.00207	0.03777	0.03168	39

020			10	157	572	287	
mmu05			370/89	0.00267	0.04601	0.03859	
014	Amyotrophic lateral sclerosis	50/810	10	212	977	723	50
mmu04			131/89	0.00338	0.05524	0.04633	
142	Lysosome	22/810	10	596	466	423	22
mmu01			124/89	0.00374	0.05799	0.04864	
200	Carbon metabolism	21/810	10	182	825	369	21
mmu05			369/89	0.00417	0.06157	0.05164	
010	Alzheimer disease	49/810	10	143	819	623	49
mmu04			110/89	0.00454	0.06402	0.05369	
931	Insulin resistance	19/810	10	382	661	974	19
mmu04			120/89	0.00553	0.07214	0.06050	
919	Thyroid hormone signaling pathway	20/810	10	234	297	701	20
mmu00			25/891	0.00558	0.07214	0.06050	
563	Glycosylphosphatidylinositol (GPI)-anchor biosynthesis	7/810	0	526	297	701	7
mmu05			180/89	0.00633	0.07636	0.06404	
152	Tuberculosis	27/810	10	567	57	865	27
mmu05			304/89	0.00643	0.07636	0.06404	
016	Huntington disease	41/810	10	205	57	865	41
mmu04			90/891	0.00665	0.07636	0.06404	
211	Longevity regulating pathway	16/810	0	121	57	865	16

Table S8. Primer sequences

Name	Seguence
mm_Wnk2_qRT_F2	GGCTCTGTGAAGATTGGTGACTTGG
mm_Wnk2_qRT_R2	GAAGGCGTAGACATCAACCGACTC
mm_Ces1d_qRT_F	ACAACAGATGTAAAGCCCATTGC
mm_Ces1d_qRT_R	GCCTTTGGCAGAACTACTCCAT
mm_Whrn_qRT_F	TCAAGGTGGGCCACGTAATTC
mm_Whrn_qRT_R	ACCAGAAAGTCGATGTAGTCTCT
mm_qRT_actb_F2	GGCTGTATTCCCCTCCATCG
mm_qRT_actb_R2	CCAGTTGGTAACAATGCCATGT
mm_nppb_qRT_F	GAGGTCACTCCTATCCTCTGG
mm_nppb_qRT_R	GCCATTTCCTCCGACTTTTCTC
